# Genetic Predisposition To Acquire a Polybasic Cleavage Site for Highly Pathogenic Avian Influenza Virus Hemagglutinin

**DOI:** 10.1128/mBio.02298-16

**Published:** 2017-02-14

**Authors:** Naganori Nao, Junya Yamagishi, Hiroko Miyamoto, Manabu Igarashi, Rashid Manzoor, Aiko Ohnuma, Yoshimi Tsuda, Wakako Furuyama, Asako Shigeno, Masahiro Kajihara, Noriko Kishida, Reiko Yoshida, Ayato Takada

**Affiliations:** a Division of Global Epidemiology, Research Center for Zoonosis Control, Hokkaido University, Sapporo, Japan; b Division of Collaboration and Education, Research Center for Zoonosis Control, Hokkaido University, Sapporo, Japan; c Administration Office, Research Center for Zoonosis Control, Hokkaido University, Sapporo, Japan; d Department of Microbiology, Graduate School of Medicine, Hokkaido University, Sapporo, Japan; e Global Institution for Collaborative Research and Education, Hokkaido University, Sapporo, Japan; f Influenza Virus Research Center, National Institute of Infectious Diseases, Musashimurayama, Japan; g School of Veterinary Medicine, the University of Zambia, Lusaka, Zambia; Baylor College of Medicine

## Abstract

Highly pathogenic avian influenza viruses with H5 and H7 hemagglutinin (HA) subtypes evolve from low-pathogenic precursors through the acquisition of multiple basic amino acid residues at the HA cleavage site. Although this mechanism has been observed to occur naturally only in these HA subtypes, little is known about the genetic basis for the acquisition of the polybasic HA cleavage site. Here we show that consecutive adenine residues and a stem-loop structure, which are frequently found in the viral RNA region encoding amino acids around the cleavage site of low-pathogenic H5 and H7 viruses isolated from waterfowl reservoirs, are important for nucleotide insertions into this RNA region. A reporter assay to detect nontemplated nucleotide insertions and deep-sequencing analysis of viral RNAs revealed that an increased number of adenine residues and enlarged stem-loop structure in the RNA region accelerated the multiple adenine and/or guanine insertions required to create codons for basic amino acids. Interestingly, nucleotide insertions associated with the HA cleavage site motif were not observed principally in the viral RNA of other subtypes tested (H1, H2, H3, and H4). Our findings suggest that the RNA editing-like activity is the key mechanism for nucleotide insertions, providing a clue as to why the acquisition of the polybasic HA cleavage site is restricted to the particular HA subtypes.

## IMPORTANCE

Influenza A viruses are divided into subtypes based on the antigenicity of the viral surface glycoproteins hemagglutinin (HA) and neuraminidase. Of the 16 HA subtypes (H1 to -16) maintained in waterfowl reservoirs of influenza A viruses, H5 and H7 viruses often become highly pathogenic through the acquisition of multiple basic amino acid residues at the HA cleavage site. Although this mechanism has been known since the 1980s, the genetic basis for nucleotide insertions has remained unclear. This study shows the potential role of the viral RNA secondary structure for nucleotide insertions and demonstrates a key mechanism explaining why the acquisition of the polybasic HA cleavage site is restricted to particular HA subtypes in nature. Our findings will contribute to better understanding of the ecology of influenza A viruses and will also be useful for the development of genetically modified vaccines against H5 and H7 influenza A viruses with increased stability.

## INTRODUCTION

Influenza A viruses (IAVs) are widely distributed in avian and mammalian species, including humans. IAVs have eight-segment negative-sense RNA genomes and are divided into subtypes on the basis of combination of two viral surface glycoproteins, hemagglutinin (HA) and neuraminidase (NA). IAVs with H1 to -16 HA and N1 to -9 NA subtypes have been isolated from wild aquatic birds, especially migratory ducks, which serve as a natural reservoir of IAVs in nature (1–3). Upon transmission to other host animals, IAVs evolve rapidly due to the high error rate of the viral RNA (vRNA) polymerase and strong immune-driven natural selection. It is also well known that human pandemic viruses emerge through reassortment among RNA segments between avian and human viruses (3, 4).

Highly pathogenic avian influenza (HPAI) viruses with the H5 and H7 HA subtypes produce high mortality in poultry and have caused devastating losses in poultry production worldwide. H5N1 HPAI viruses, first reported in Hong Kong in 1997, have been circulating in poultry for almost two decades (5) and have spread to more than 60 countries in Eurasia and Africa. In addition to infection of avian species, it has been reported that H5N1 HPAI viruses are occasionally transmitted to humans and cause severe pneumonia with high case fatality rates (6). Since the first fatal human infection caused by H5N1 HPAI viruses was recognized in 1997 (7, 8), 874 human cases, with 458 deaths, have been reported (as of 3 October 2016 [http://www.who.int/en/]). Some reassortant H5 viruses with different NA subtypes (e.g., H5N2, H5N8, and 5N6), originating from the same ancestral H5N1 virus, have recently emerged in China and spread to other countries in Eurasia and North America (9–14).

It is known that HPAI viruses evolve from low-pathogenic H5 and H7 viruses maintained in the natural reservoirs and that the key determinant for the different pathogenicities is the proteolytic cleavage of HA, which is required for infectivity of IAVs (15–19). Low-pathogenic IAVs contain a single arginine residue at the cleavage site of HA, which is cleaved only by trypsin-like proteases and therefore produces localized infection of the respiratory and/or intestinal tracts, causing asymptomatic or mild infection. After introduction into domestic poultry, low-pathogenic viruses often acquire multiple basic amino acids at the HA cleavage site, which is recognized by ubiquitous cellular proteases such as furin and PC6 (20–23), thereby rendering the virus capable of causing systemic infection with a fatal outcome in terrestrial poultry. The polybasic HA cleavage site is known to be generated by multiple nucleotide insertions/substitutions to create codons for basic amino acids (17, 24–26) or by recombination with cellular or viral RNAs (27–29) and is considered to be the primary virulence marker of HPAI viruses (16, 30).

Most outbreaks of HPAI are caused only by IAVs with the H5 and H7 subtypes. However, it was reported that an H6 virus with an artificially introduced polybasic HA cleavage site acquired high HA cleavability without trypsin and had a typical HPAI phenotype in experimentally infected chickens (31). An H9 virus also acquired high pathogenicity for chickens via the introduction of a pair of dibasic amino acid residues at the HA cleavage site and subsequent passages in chickens (32). Furthermore, other HA subtypes (i.e., H2, H4, H8, and H14) similarly supported a highly pathogenic phenotype after artificial introduction of the polybasic HA cleavage site in the appropriate genetic background (33). These studies strongly suggest that the restriction of naturally occurring nucleotide insertions or substitutions to produce multiple basic amino acids in the cleavage sites of H5 and H7 HAs is primarily due to the unique genetic predisposition of these HA subtypes, not a structural or functional limitation of the other HA subtypes.

**FIG 1:**
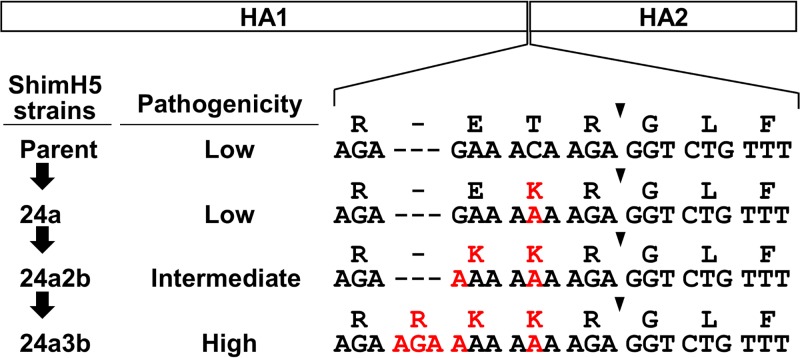
HA cleavage site sequences of ShimH5 and its variants passaged in chickens. HA was synthesized as a single polypeptide and then cleaved into HA1 and HA2 subunits at the cleavage site (indicated by arrowheads). ShimH5 and its variants (parent, 24a, 24a2b, and 24a3b) strains have different nucleotide and amino acid sequences at their HA cleavage sites and different pathogenicities for chickens (26). Nucleotide sequences at positions 1043 to 1063 (ShimH5 parent, 24a, and 24a2b) and 1043 to 1066 (24a3b) and the corresponding amino acid sequences are shown. Dashes are included to adjust the sequence alignment, and nucleotides and amino acids different from those of the parental ShimH5 sequence are shown in red.

In this study, we focused on the A/whistling swan/Shimane/499/83 (H5N3) (ShimH5) strain, which was originally isolated as a low-pathogenic strain and shown to become highly pathogenic after passaging through experimentally infected chickens (26). During serial passages through chickens, the ShimH5 strain first underwent two point mutations at nucleotide positions 1050 (C to A [ShimH5 24a]) and 1046 (G to A [ShimH5 24a2b]) in the RNA sequence encoding the HA cleavage site motif (R-E/K-T/K-R) and then acquired 5 consecutive basic amino acids, R-R-K-K-R, via the insertion of a codon for an arginine residue at the cleavage site (ShimH5 24a3b) (26) (Fig. 1). We found that this RNA sequence was involved in a stem-loop structure in the predicted secondary RNA structure and that the primary point mutations at positions 1046 and 1050 resulted in the creation of 8 consecutive adenine residues, which enlarged the loop structure and accelerated the frequency of nucleotide insertions. Interestingly, consecutive adenine residues and large stem-loop structures in this RNA region were frequently found only in particular HA subtypes (e.g., H5). While the importance of the RNA secondary structure of H5 HA genes has been previously proposed for the emergence of HPAI (25), we experimentally tested the hypothesis using a reverse-genetics approach and found the direct link between the frequency of nucleotide insertions and predicted stem-loop structures of the viral RNA. Our findings suggest that the RNA sequence determining the HA cleavage site amino acid motif has a key role in inducing viral polymerase slippage, which increases the frequency of nucleotide insertions, and that this mechanism contributes to the acquisition of additional codons for basic amino acids to create the polybasic HA cleavage site.

## RESULTS

### Reporter gene expression resulting from nontemplated nucleotide insertions into the ShimH5 HA sequence.

**FIG 2:**
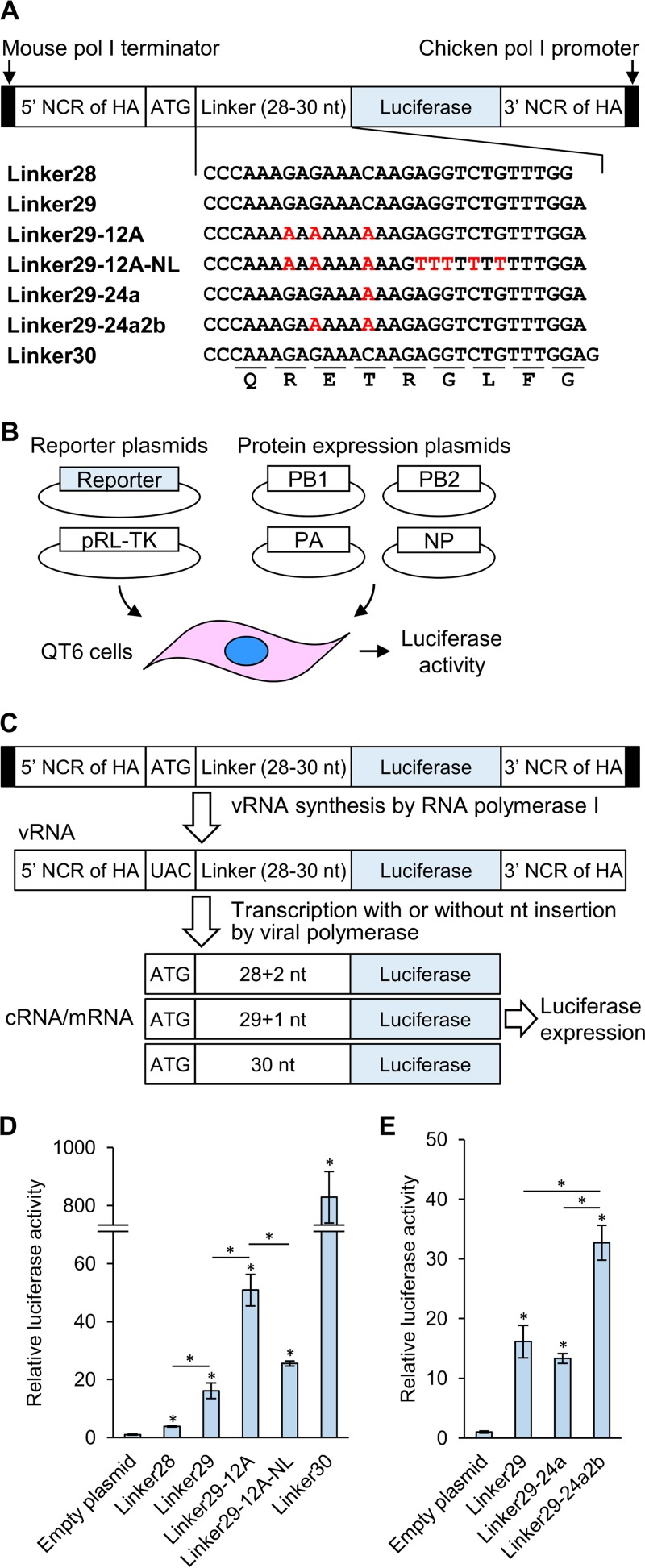
Schematic overview of the reporter assay system and luciferase activity in QT6 cells. (A) The reporter plasmids contained the chicken RNA polymerase I promoter, mouse RNA polymerase I terminator, PR8 HA segment-derived NCR, and firefly luciferase gene. Linkers (28, 29, or 30 polynucleotides) that originated from the RNA sequence encoding amino acids across the HA cleavage site of ShimH5 were inserted between a start codon and the firefly luciferase gene lacking its start codon. The nucleotide positions different from those of the parental ShimH5 sequence are indicated in red. (B) QT6 cells were transfected with the reporter plasmid, pRL-TK *Renilla* luciferase transfection control reporter plasmid, and a mixture of PB2-, PB1-, PA-, and NP-expressing plasmids, and luciferase (firefly and *Renilla* luciferase) activities were measured. (C) In this assay, negative-sense vRNA templates are transcribed from the reporter plasmid by cellular RNA polymerase I, and then mRNA and cRNA are produced by the PR8 polymerases and NP, which are provided by the cotransfected protein expression plasmids. The transcripts containing 28- or 29-polynucleotide linkers produce mRNAs that are not in frame with the ORF of the reporter gene. Therefore, the firefly luciferase is expected to be expressed when nucleotides are inserted into the linker region of mRNA, cRNA, and/or vRNA to make the linker sequence in frame with the ORF of the reporter gene. The firefly luciferase activities were standardized using the values given by the activities of the transfection control, *Renilla* luciferase. (D and E) Luciferase activities were expressed relative to the empty plasmid and compared among the reporter plasmids containing the indicated linkers. Representative data from three independent experiments are shown. Relative luciferase activities are presented as the averages and standard deviations from triplicate wells. Statistical significance was calculated using Student’s *t* test (*, *P* < 0.05). Asterisks placed directly above bars indicate significant differences compared to the empty plasmid, and asterisks placed between bars show significant differences between the indicated bars.

We established a reporter assay to detect nucleotide insertions into the RNA sequence encoding amino acids across the HA cleavage site (29 nucleotides, CCCAAAGAGAAACAAGAGGTCTGTTTGGA [designated RNAseqHAclv]) of the strain ShimH5 (Fig. 2A to C). In the reporter plasmid, the firefly luciferase gene lacking its start codon was inserted downstream of the start codon/linker region (e.g., 29 polynucleotides corresponding to RNAseqHAclv [designated Linker29]). Since the firefly luciferase gene that followed the 28- or 29-nucleotide linkers was out of frame, the luciferase was expected to be expressed only when nucleotides were inserted into the linker region of mRNA, cRNA, and/or viral RNA (vRNA) to make the linker sequence in frame with the open reading frame (ORF) of the reporter gene.

To evaluate this system, we first tested the plasmids containing the linkers (Linker28, Linker29, and Linker30), all of which had the sequence derived from the parent ShimH5 strain (Fig. 2A). QT6 cells (34) were transfected with these plasmids, and luciferase activities of cell lysates were measured (Fig. 2D). As expected, a high level of luciferase activity was detected in the cells transfected with the construct with Linker30, which had the sequence in frame with the ORF of the reporter gene. Interestingly, we found that the plasmids containing Linker28 and Linker29 expressed slightly but significantly higher levels of luciferase than the empty plasmid. Significantly higher luciferase expression was observed in cells transfected with the plasmid containing Linker29 than with Linker28. These results suggested that nontemplated nucleotide insertions into these linker regions occurred during the synthesis of mRNA, cRNA, and/or vRNA.

### Accelerated nucleotide insertions in the presence of consecutive adenine residues.

We then hypothesized that a stretch of adenine residues in the RNAseqHAclv region might affect the frequency of nucleotide insertions, as has been suggested with the RNA editing mechanisms of some RNA viruses (35–39). Thus, to test this hypothesis, we constructed another plasmid containing a modified version of Linker29 including runs of 12 adenines with G-to-A or C-to-A substitutions (Linker29-12A) (Fig. 2A) and compared the luciferase activities. We detected much higher levels of luciferase expression in QT6 cells transfected with this plasmid (Fig. 2D). Next, we tested the impact of the two nucleotide substitutions (C-to-A or G-to-A) that had actually been found in the ShimH5 24a2b HA gene prior to the nucleotide insertion to create a codon for an arginine residue at the HA cleavage site motif (26). We constructed plasmids containing Linker29-24a and Linker29-24a2b with the sequences of strains ShimH5 24a and 24a2b, which had 6 and 8 consecutive adenines in the RNAseqHAclv region, respectively (Fig. 1 and 2A). We found that the amount of luciferase expression from the cells transfected with the plasmid containing a single substitution (Linker29-24a) was similar to that of cells transfected with the plasmid with Linker29. Interestingly, however, the additional substitution (Linker29-24a2b) significantly enhanced the luciferase expression (Fig. 2E). These results suggested that these two nucleotide substitutions, resulting in the presence of 8 consecutive adenine residues, accelerated the nucleotide insertions into the linker sequence.

### Comparison of secondary structures of the linker sequences.

**FIG 3:**
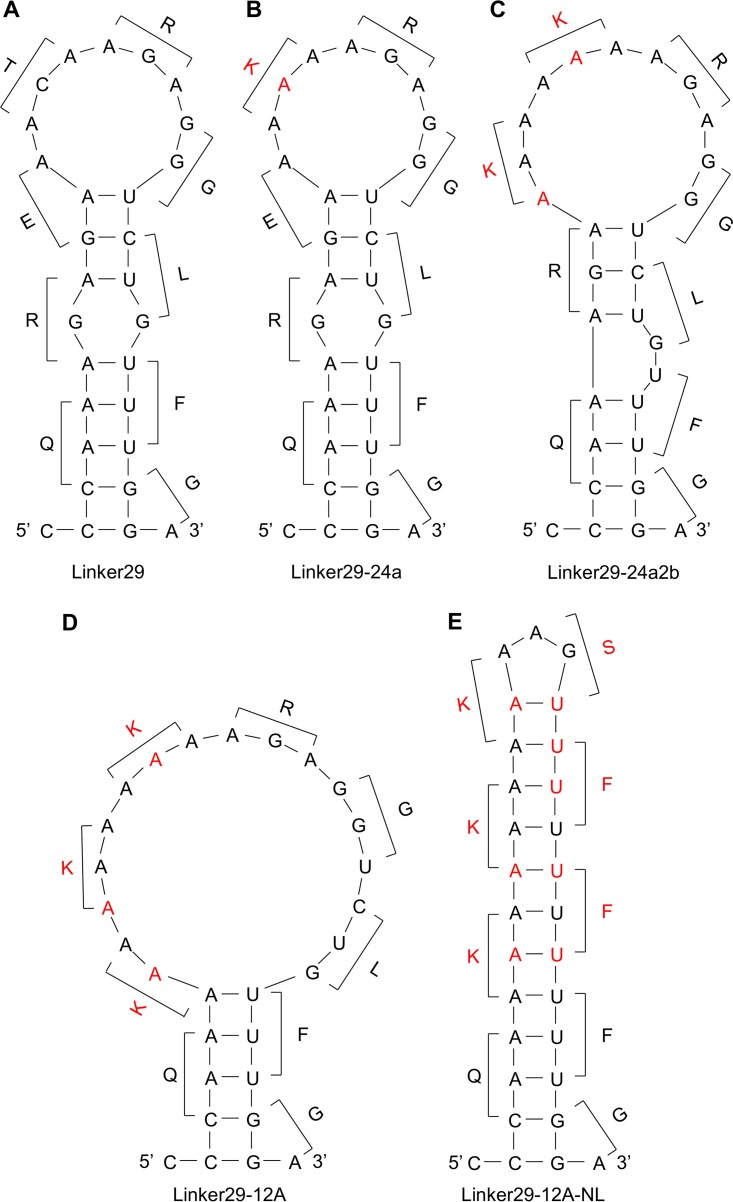
Quickfold-predicted RNA structures of the linker sequences. The predicted RNA (positive-sense) secondary structures of Linker29 (A), Linker29-24a (B), Linker29-24a2b (C), Linker29-12A (D), and Linker29-12A-NL (E) and amino acids corresponding to each codon are shown. Nucleotides and amino acids different from the parental ShimH5 sequence are shown in red.

We then compared the predicted secondary structures of RNAs corresponding to the Linker29, Linker29-24a, Linker29-24a2b, and Linker29-12A sequences (Fig. 3; see Fig. S1 in the supplemental material). Interestingly, all linker RNAs potentially formed stem-loop structures mainly consisting of adenine and guanine residues. These stem-loop structures were maintained even when longer sequences (i.e., 39 and 49 nucleotides, but not 69 nucleotides or more) including the RNAseqHAclv region in the middle were used for the analysis (see Fig. S2 in the supplemental material). It was noted that the loop magnitude was correlated with the efficiency of luciferase expression. To confirm the importance of the stem-loop structure, we tested an additional reporter plasmid containing Linker29-12A-NL, which was artificially designed to have 12 consecutive adenines but to minimize the loop structure (Fig. 2A and 3E). As expected, significantly lower luciferase expression was observed in the cells transfected with this plasmid than in those transfected with the plasmid containing Linker29-12A (Fig. 2D).

10.1128/mBio.02298-16.3 FIG S1  RNAfold-predicted RNA structures of the linker sequences. The predicted RNA (positive-sense) secondary structures of Linker29 (A), Linker29-24a (B), Linker29-24a2b (C), Linker29-12A (D), and Linker29-12A-NL (E) are shown. The structures are colored by base-pairing probabilities indicated with color bars. For unpaired regions, the color denotes the probability of being unpaired. Download FIG S1, PDF file, 0.2 MB. Copyright © 2017 Nao et al. 2017 Nao et al. This content is distributed under the terms of the Creative Commons Attribution 4.0 International license.

10.1128/mBio.02298-16.4 FIG S2  Predicted RNA structures of longer sequences, including the linker sequences. The RNA (positive-sense) secondary structures were generated by the Quickfold (A) and RNAfold (B) programs for Linker29, Linker29-24a, Linker29-24a2b, Linker29-12A, and Linker29-12A-NL. The structures are colored by base-pairing probabilities indicated with color bars (B). For unpaired regions, the color denotes the probability of being unpaired. Download FIG S2, PDF file, 0.5 MB. Copyright © 2017 Nao et al. 2017 Nao et al. This content is distributed under the terms of the Creative Commons Attribution 4.0 International license.

### Nontemplated nucleotide insertions found in the vRNA of purified virions.

**FIG 4:**
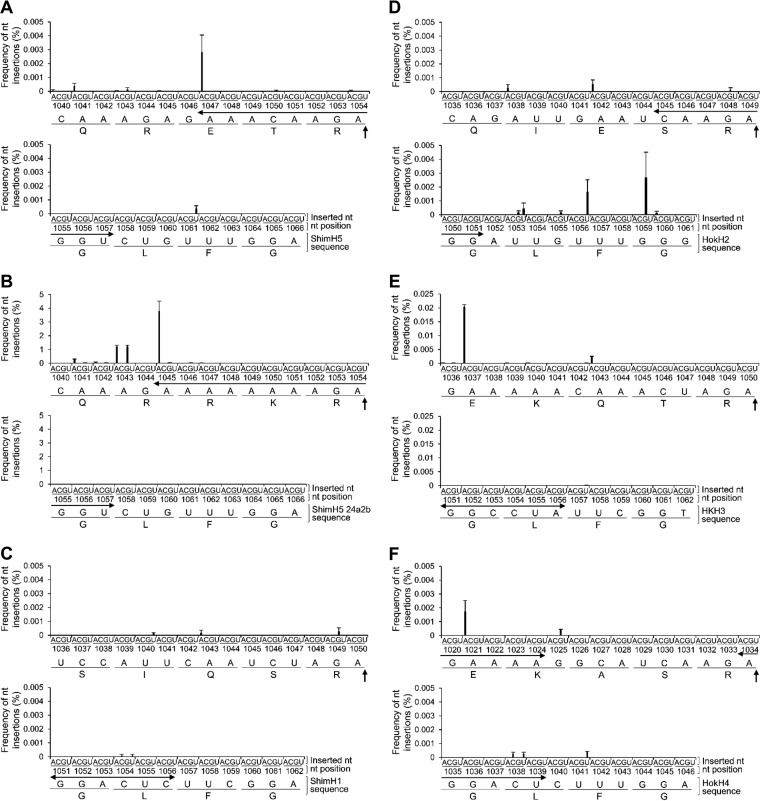
Nucleotide insertions detected by deep-sequencing analysis of vRNAs in virus particles. Total frequencies of nucleotide (nt) insertions into 9 codons in the RNAseqHAclv region of ShimH5 (A), ShimH5 24a2b (B), ShimH1 (C), HokH2 (D), HKH3 (E), and HokH4 (F) HA genes are shown. In case multiple nucleotide insertions were observed at the same position, inserted nucleotides were counted individually. (For example, if an AGA insertion was observed at nucleotide position 1045, it was counted as two adenine insertions and one guanine insertion at position 1045.) The HA cleavage site is indicated by vertical arrows. Horizontal arrows indicate the sequence corresponding to predicted loop structures (1047 to 1057, 1045 to 1057, 1051 to 1056, 1045 to 1051, 1051 to 1056, and 1020 to 1024/1035 to 1039 for the ShimH5, ShimH5 24a2b, ShimH1, HokH2, HKH3, and HokH4 HA genes, respectively). Frequencies of nucleotide insertions are presented as the averages and standard deviations from two or three independent experiments. Since which nucleotide position allowed the insertion into the consecutive adenines was not distinguishable, total frequencies for positions 1047 to 1049 (A), 1045 to 1052 (B), 1037 to 1041/1043 to 1045 (E), and 1021 to 1024 (F) are collectively shown at positions 1047, 1045, 1037/1043, and 1021, respectively. Similarly, total frequencies of uracil and guanine insertions are collectively shown at positions 1056 and 1059, respectively (D).

**FIG 5:**
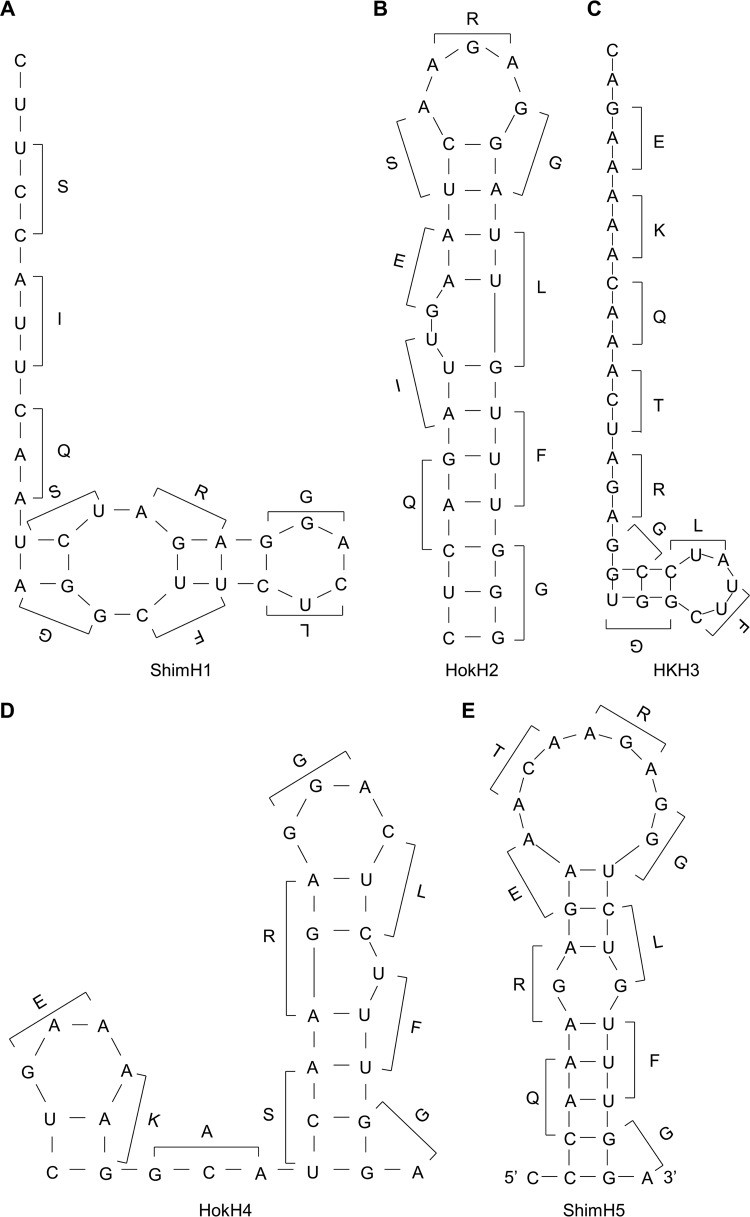
Comparison of Quickfold-predicted secondary structures of the RNAseqHAclv regions of ShimH1, HokH2, HKH3, HokH4, and ShimH5. The predicted RNA (positive-sense) secondary structures of the RNAseqHAcly region of ShimH1 (A), HokH2 (B), HKH3 (C), HokH4 (D), and ShimH5 (E) and amino acids corresponding to each codon are shown.

To verify the presence of vRNA containing the expected nucleotide insertions, we analyzed the ShimH5 HA vRNA incorporated into purified virions using a deep-sequencing approach. Reassortant viruses between A/Puerto Rico/8/1934 (H1N1) (PR8) and ShimH5 strains (rgPR8/ShimH5-HA [PR8 whose HA gene was replaced with that of ShimH5] and rgPR8/ShimH5 24a2b-HA [PR8 whose HA gene was replaced with that of ShimH5 24a2b]) were generated from cloned plasmids to standardize the viral polymerase activity and minimize the background genetic heterogeneity in the original population of the ShimH5 strains. As expected, we found single-nucleotide insertions of adenine residues into the 3 consecutive adenines at nucleotide positions 1047 to 1049 in rgPR8/ShimH5-HA (Fig. 4A) at a comparatively high frequency (0.0028%). No reads had multiple nucleotide insertions (i.e., double, triple, or more) in these positions. Surprisingly, adenine insertions into the 8 consecutive adenines at nucleotide positions 1045 to 1052 in rgPR8/ShimH5 24a2b-HA were found at a much higher frequency (3.8%) (Fig. 4B). Among the sequence reads in which the insertions were detected, 61.0%, 34.5%, 3.0%, and 1.5% had single, double, triple, and quadruple or more nucleotide insertions, respectively. Double insertions (i.e., AG and AA at nucleotide positions 1043 and 1045 to 1052, respectively), which could be part of the codon for arginine or lysine, were found in 1.2% and 0.35% of the total reads. Furthermore, 0.035% of the total reads contained the AGA insertion, which had in fact been observed in the RNAseqHAclv region of the ShimH5 24a3b HA gene. These findings indicated that virus particles carrying the HA gene segment containing such nucleotide insertions were indeed produced by infected cells. To investigate nucleotide insertions in other HA subtypes, we then cloned HA genes of A/pintail/Shimane/324/1998 (H1N9) (ShimH1), A/duck/Hokkaido/95/2001 (H2N2) (HokH2), A/duck/Hong Kong/836/1980 (H3N1) (HKH3), and A/duck/Hokkaido/138/2007 (H4N6) (HokH4) and similarly produced reassortant viruses carrying HA segments of these 4 viruses in the PR8 background (rgPR8/ShimH1-HA, rgPR8/HokH2-HA, rgPR8/HKH3-HA, and rgPR8/HokH4-HA, respectively). Nucleotide insertions into the HA vRNAs of in purified virus particles were analyzed by deep sequencing (Fig. 4C to F). Interestingly, we found that no remarkable nucleotide insertion was observed in the RNA sequences corresponding to the RNAseqHAclv region of rgPR8/ShimH1-HA and that single-nucleotide insertions that were not associated with the HA cleavage site motif were only observed in rgPR8/HokH2-HA, rgPR8/HKH3-HA, and rgPR8/HokH4-HA. It was also noted that the predicted secondary RNA structures of this region are remarkably different among these HAs (Fig. 5; see Fig. S3 in the supplemental material).

10.1128/mBio.02298-16.5 FIG S3  Comparison of RNAfold-predicted secondary structures of the RNAseqHAclv regions of ShimH1, HokH2, HKH3, HokH4, and ShimH5. The predicted RNA (positive-sense) secondary structures of the RNAseqHAcly region of ShimH1 (A), HokH2 (B), HKH3 (C), HokH4 (D), and ShimH5 (E) are shown. The structures are colored by base-pairing probabilities indicated with color bars. For unpaired regions, the color denotes the probability of being unpaired. Download FIG S3, PDF file, 0.2 MB. Copyright © 2017 Nao et al. 2017 Nao et al. This content is distributed under the terms of the Creative Commons Attribution 4.0 International license.

### Differences in the predicted RNA structures among HA subtypes.

**FIG 6:**
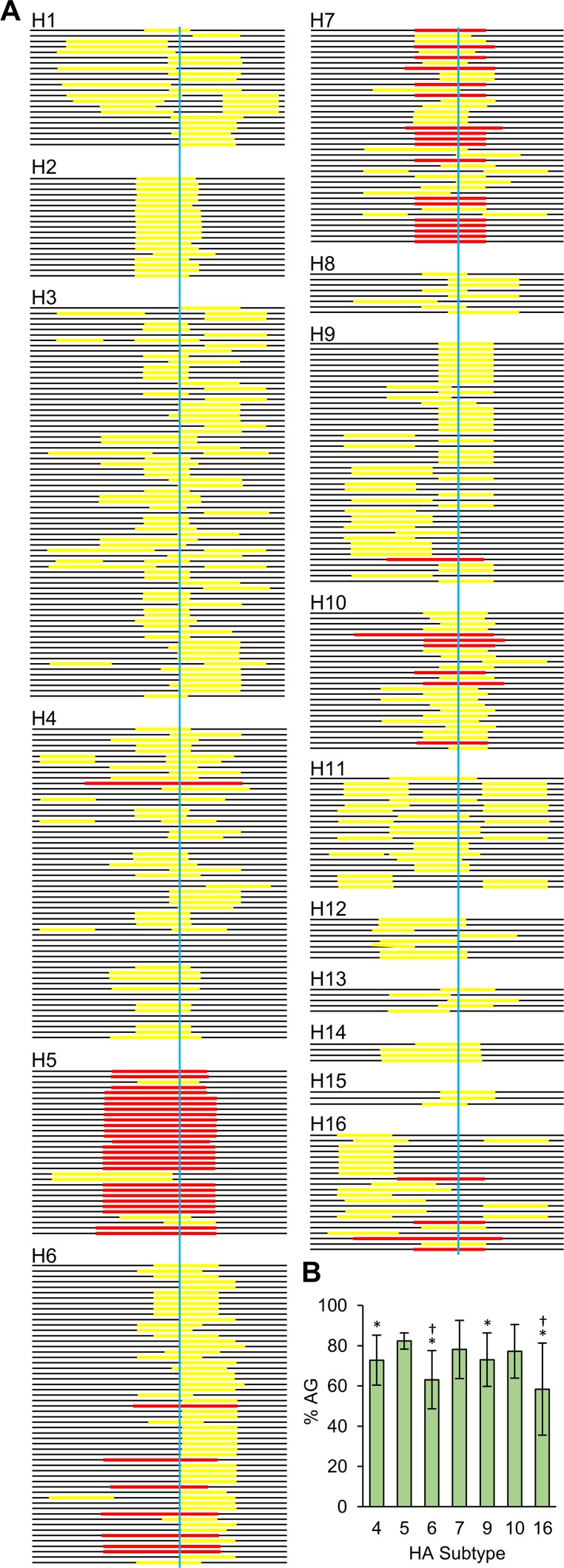
Putative loop regions found in the RNAseqHAclv region of low-pathogenic IAVs isolated from ducks. The RNA (positive-sense) secondary structures were generated by the Quickfold program. (A) Horizontal bars represent the RNAseqHAclv region, and colored (yellow and red) lines on each bar show nucleotide regions corresponding to the predicted loops in each stem-loop structure. Red lines indicate the loop structures consisting of more than 8 nucleotides that are fully included the codons for arginine and glycine at the HA cleavage site, and yellow lines show the others. The HA cleavage sites are indicated by blue vertical lines. (B) The AG ratios of the loop sequences in each RNAseqHAclv region were calculated, and averages and standard deviations of each HA subtype are shown. Statistical significance compared to H5 (*) and H7 (†) was calculated using Student’s *t* test (*P* < 0.05). There was no significant difference between H5 and H7.

Finally, we compared the predicted secondary structures of the RNAseqHAclv region among HA subtypes using database sequences of low-pathogenic IAVs isolated from ducks (Fig. 6; see Fig. S4 in the supplemental material). This comprehensive analysis revealed that the stem-loop structure was found in most of the viruses regardless of the HA subtype. Importantly, however, its size and location varied drastically. Most (26/31) of the RNAseqHAclv region of the H5 subtype contained large loop structures consisting of 8 to 14 nucleotides and fully included the codons for arginine and glycine at the HA cleavage site. Although the loop structure tended to be a little smaller than those found in H5 viruses, 45% (18/40) of the H7 sequences had loops (8 to 11 nucleotides) involving the codons encoding the cleavage site. In contrast, such long and well-positioned loop structures in the RNAseqHAclv region were not found in the H1, H2, H3, H8, H11, H12, H13, H14, or H15 subtypes and were found more infrequently in the H4, H6, H9, H10, and H16 subtypes (18/58, 7/56, 1/45, 6/26, and 4/22, respectively) than in the H5 and H7 subtypes. Two different programs used for the prediction showed a similar tendency of the presence of the stem-loop structure (see Data Set S2 in the supplemental material). It was also noted that the loop regions of the H5 and H7 RNAs generally had AG-rich sequences compared to the H4, H6, H9, and H16 subtypes (Fig. 6B; see Data Set S1 in the supplemental material).

10.1128/mBio.02298-16.1 DATA SET S1  Strain names and nucleotide sequences of 29 nucleotides encoding the amino acid around the HA cleavage sites of all analyzed viruses. Nucleotides included in loop structures are indicated in red. Codons for basic amino acids at the cleavage site are underlined (only for the earliest isolates). Download DATA SET S1, XLSX file, 0.04 MB. Copyright © 2017 Nao et al. 2017 Nao et al. This content is distributed under the terms of the Creative Commons Attribution 4.0 International license.

10.1128/mBio.02298-16.2 DATA SET S2  Comparison of RNA secondary structures predicted by Quickfold and RNAfold. The secondary structures of the RNAseqHAclv region of low-pathogenic IAVs isolated from waterfowl (441 strains used in Fig. 6; Data Set S1) were predicted using Quickfold and RNAfold programs and shown in the “dot-parenthesis” format. Briefly a structure on a sequence of 29 nucleotides is represented by a string of 29 letters consisting of matching parentheses and dots. A base pair between bases X and Y is represented by “(” at position X and “)” at position Y. Unpaired bases are represented by dots. Underlined positions (only for the earliest isolates) represent codons of basic amino acids at the cleavage site. Similarities of the secondary structures predicted by Quickfold and RNAfold (similarity score) are shown as follows: 2, exactly the same; 1, not exactly the same but containing the same loop structures at least partly including the codons for arginine and/or glycine at the HA cleavage site; and 0, other than score 1 or 2. Download DATA SET S2, XLSX file, 0.03 MB. Copyright © 2017 Nao et al. 2017 Nao et al. This content is distributed under the terms of the Creative Commons Attribution 4.0 International license.

10.1128/mBio.02298-16.6 FIG S4  Secondary structures of the RNAseqHAclv region of low-pathogenic IAVs isolated from waterfowl reservoirs. Secondary structures of the RNAseqHAclv region were generated by the Quickfold program according to the database sequences (NCBI Influenza Virus Resource Database) of low-pathogenic IAVs isolated from waterfowl (441 strains). All structures are shown as positive-sense RNA sequences. Download FIG S4, PDF file, 2.3 MB. Copyright © 2017 Nao et al. 2017 Nao et al. This content is distributed under the terms of the Creative Commons Attribution 4.0 International license.

## DISCUSSION

In this study, we demonstrated that nontemplated nucleotide insertions frequently occurred in the RNAseqHAclv region of ShimH5. The nucleotide insertions were indeed found in the vRNA incorporated into virions, and consecutive adenine residues, known as a key factor for the RNA editing mechanism of some RNA viruses (35–39), accelerated the frequency of the nucleotide insertions. Runs of adenines are present in the RNA editing site of ebolaviruses (38) and paramyxoviruses (39), where the virus polymerase inserts nontemplated nucleotides during mRNA synthesis to create alternative reading frames. The increase in the number of adenine residues in the editing site of paramyxoviruses enhances the frequency of additional nucleotide insertions at the editing site (39). In addition to viral polymerases, it has been reported that transcriptional slippage by *Escherichia coli* RNA polymerase during RNA elongation at runs of 10 or more adenines or thymines results in the addition of nontemplated uracil or adenine residues, leading to the restoration of the normal reading frame from out-of-frame *lacZ* constructs (40). Consistent with these previous studies, our data demonstrated that insertion of additional nucleotides into the RNA sequence determining the H5 HA cleavage site motif frequently occurs, most likely due to viral polymerase slippage induced by the presence of consecutive adenine residues.

Secondary structures of RNA molecules are also thought to be important for the RNA editing mechanism (41). The editing site of simian virus 5 has a stem-loop structure that is proposed to be essential for the RNA editing activity of the viral polymerase (42). Similarly, a hairpin-like structure of the ebolavirus glycoprotein (GP) gene is important for RNA editing (35). It was recently reported that the nucleotide mutations destabilizing the predicted stem-loop structure just upstream from the editing site of the ebolavirus GP gene dramatically reduced the RNA editing efficiency, whereas other mutations irrelevant to the destabilization of the stem-loop structure did not affect the editing (37). In this study, we found that almost all the H5 viruses examined had potential stem-loop structures in the RNAseqHAclv region. It was also noted that ShimH5 24a2b had a larger loop than the parent ShimH5 and 24a strains and that the nucleotide insertion-dependent reporter gene expression and actual frequency of the nucleotide insertions detected by deep sequencing of vRNA were correlated with the loop size and the number of consecutive adenines. Our data suggested that the enlarged stem-loop structure consisting of consecutive adenine and guanine residues was important to accelerate the nucleotide insertion. Accordingly, adenines were inserted into nucleotide positions 1047 to 1049 (ShimH5) and 1045 to 1052 (ShimH5 24a2b) in the HA genes, both of which were included in the loop structure of their predicted secondary RNA structures. These nucleotide insertions might enlarge the loop size and further accelerate the additional nucleotide insertions. Nucleotide insertions into the RNAseqHAclv region were also detected in H2, H3, and H4 HAs with comparatively high frequency (approximately 0.002 to 0.02%), although detailed mechanisms of these nucleotide insertions are unclear. However, importantly, the positions of these inserted nucleotides were apart from the loop structures and/or the sequences encoding the HA cleavage motif, suggesting that these nucleotide insertions might not directly contribute to the additional nucleotide insertions to create basic amino acid(s) in the HA cleavage site (Fig. 4 and 5).

This study provides the first experimental evidence for the potential contribution of the RNA secondary structure (i.e., stem-loop) to create the polybasic HA cleavage sites of IAVs, which has only been discussed hypothetically (25, 43). Taken together, our data suggest that the RNA editing-like mechanism plays a key role in insertion of additional nucleotides into the vRNA sequence determining the HA cleavage site motifs of HPAI viruses with the H5 and possibly H7 subtypes. The loop sequence consisting of consecutive adenines/guanines may also be favorable to create codons for lysine and/or arginine residues (e.g., AAA, AAG, AGA, and AGG). We assume that this genetic predisposition, which is found in H5 and partly in H7 subtypes, explains why the acquisition of basic amino acids at the HA cleavage site is restricted to these HA subtypes in nature. Interestingly, however, some viruses with H4, H6, H9, H10, and H16 subtypes seem to have such genetic backgrounds, requiring further studies to clarify whether these subtypes also have the potential to naturally acquire the polybasic HA cleavage site and become highly pathogenic.

Although we detected nucleotide insertions during the vRNA synthesis of the parent ShimH5 strain, the frequency did not seem to be high compared to the RNA editing observed in other viruses (35, 36, 39). It is important to note that an HA gene containing only a single- or double-nucleotide insertion into its ORF does not express complete HA molecules due to frameshift and that multiples of three nucleotide insertions (i.e., codons such as AAA or AGA) are required to generate a functional HA gene. Thus, further studies are needed to clarify the mechanisms by which HA segments carrying a nonfunctional (i.e., frameshifted) HA gene can be maintained in the virus population until nucleotide insertions into vRNA of the HA gene are accumulated to create basic amino acid codons during circulation in terrestrial poultry.

## MATERIALS AND METHODS

### Viruses and cells.

IAV strain ShimH5 was kindly provided by T. Ito (Tottori University), and ShimH1, HokH2, HKH3, and HokH4 were kindly provided by H. Kida (Hokkaido University). These viruses were propagated in the allantoic cavities of 10-day-old embryonated chicken eggs at 35°C for 48 h and stored at −80°C until use. MDCK cells (44) were grown in Dulbecco’s modified Eagle’s medium (DMEM) supplemented with 10% calf serum, 100 U/ml penicillin, and 0.1 mg/ml streptomycin. 293T cells (45) were grown in DMEM supplemented with 10% fetal calf serum and antibiotics as described above. QT6 cells were maintained in Kaighn’s modification of Ham’s F-12 medium supplemented with 5% calf serum and 10% tryptose phosphate broth. All cells were incubated at 37°C in a 5% CO_2_ incubator.

### Reporter assay.

We used the modified pHW72 plasmid (46), pHW72-LUC-CKpolI, containing the chicken RNA polymerase I promoter, mouse RNA polymerase I terminator, PR8 HA segment-derived noncoding region (NCR), and firefly luciferase gene. The reporter plasmid was constructed by inserting 28-, 29-, or 30-polynucleotide linkers whose sequences were derived from those encoding the HA cleavage site (i.e., RNAseqHAclv, including nucleotides at positions 1038 to 1065, 1066, or 1067) of the ShimH5 strains, between a start codon and the remaining ORF of the firefly luciferase gene. This construct was flanked by the PR8 HA-NCR at both the 5′ and 3′ ends (Fig. 2A). Eukaryotic expression plasmid pCAGGS/MCS (controlled by the chicken β-actin promoter) encoding the PR8 virus polymerases (PB2, PB1, and PA) and NP were kindly provided by Y. Kawaoka (University of Tokyo). Seventy percent-confluent QT6 cell monolayers in 24-well tissue culture plates were transfected with 150 ng of pHW72-LUC-CKpolI plasmids containing the respective linkers, 5 ng of a pRL-TK *Renilla* luciferase transfection control reporter plasmid (Promega), and a mixture of PB2-, PB1-, PA-, and NP-expressing pCAGGS in quantities of 150, 150, 150, and 300 ng, respectively, using FuGENE HD (Promega) according to the manufacturer’s protocol. At 24 h posttransfection, luciferase (firefly and *Renilla* luciferase) activities of cell lysates were measured with a GloMax96 Microplate luminometer (Promega) using the dual-luciferase assay system (Promega) according to the manufacturer’s protocol. Firefly luciferase activities were standardized to the transfection control *Renilla* luciferase activities (i.e., firefly luciferase activities were divided by *Renilla* luciferase activities).

### Generation of infectious viruses from plasmids.

The vRNAs of ShimH1, HokH2, HKH3, HokH4, and ShimH5 were extracted from infectious allantoic fluids using a QIAamp viral RNA minikit (Qiagen) and reverse transcribed with Moloney murine leukemia virus reverse transcriptase (Invitrogen) using the uni12 primer (5′-AGCAAAAGCAGG) (47). For the expression of vRNAs, cDNAs of the HA gene segments of these 4 viruses were cloned into the pHH21 plasmid, which contains the human RNA polymerase I promoter and the mouse RNA polymerase I terminator separated by BsmBI sites (48). The pHH21-based plasmid for the expression of the ShimH5 24a2b HA was generated by PCR-based mutagenesis using primers containing the desired nucleotide substitutions. Reassortant viruses (rgPR8/ShimH1-HA, rgPR8/HokH2-HA, rgPR8/HKH3-HA, rgPR8/HokH4-HA, rgPR8/ShimH5-HA, and rgPR8/ShimH5-24a2b-HA) were generated by a reverse-genetics system as described previously (48) with slight modification. Briefly, 293T cells were transfected with 1 μg of each of the 12 plasmids (8 pHH21-based plasmids for vRNA expression and 4 pCAGGS-based plasmids for viral polymerase/NP expression) carrying the ShimH1, HokH2, HKH3, HokH4, ShimH5, or ShimH5 24a2b HA and PR8 background genes using TransIT-LT1 (Mirus Bio) according to the manufacturer’s protocol. Forty-eight hours after transfection, the supernatant of transfected 293T cells was collected, diluted at 1:10, and transferred into confluent monolayers of MDCK cells. Rescued viruses propagated once in MDCK cells were stored at −80°C until use. Virus titers were determined as PFU using MDCK cells.

### Virus purification and RNA extraction for deep sequencing.

Cultured MDCK cells maintained in Eagle’s minimal essential medium (MEM) containing bovine serum albumin (0.3%), penicillin (100 U/ml), and streptomycin (0.1 mg/ml) were infected with rgPR8/ShimH1-HA, rgPR8/HokH2-HA, rgPR8/HKH3-HA, rgPR8/HokH4-HA, rgPR8/ShimH5-HA, or rgPR8/ShimH5-24a2b-HA at a multiplicity of infection of 0.001 and incubated at 37°C for 48 h. Then the infectious supernatant was collected. Virus particles were concentrated and purified by high-speed centrifugation (28,000 rpm for 2 h at 4°C) of the supernatant through a 10 to 50% sucrose density gradient. vRNAs were extracted from purified virus particles using TRIzol LS reagent (Sigma) according to the manufacturer’s protocol.

### Library preparation and deep sequencing.

cDNA libraries were prepared from vRNA without any amplification procedures to minimize the potential errors during the sequencing reaction, and the high depth of coverage of sequencing (more than 100,000 reads) with a high-quality score (no less than 30) enabled us to analyze the vRNA quasispecies, including infrequent nucleotide insertions that might not be detectable by Sanger sequencing. Briefly, extracted vRNA (10 μg) was used for the synthesis of double-stranded cDNA of the partial HA gene containing the sequence encoding the HA cleavage site with a PrimeScript double-strand cDNA synthesis kit (TaKaRa) using the HA gene-specific primers H1-996F (5′-GGAGAATGCCCTAAATATGTTAAAAGC), H2-1000F (5′-GCCCCAAATATGTAAAATCGGAGAG), H3-1001F (5′-GCCCCAAGTATGTTAAGCAAAACAC), H4-985F (5′-GCCCCAAATATGTTAAACAGGGCTC), and H5-963F (5′-GTATGCCTTTCCACAATATTCATCC). The synthesized double-stranded cDNAs (approximately 750 bp) were tagged with sequencing adapters having indexes by using a TruSeq DNA PCR-free sample prep kit (Illumina). The cDNA libraries were verified on a high-sensitivity DNA chip on a Bioanalyzer (Agilent Technologies) and quantified with real-time PCR using an Illumina compatible kit and standards (KAPA) before loading on the sequencing chip. Then the indexed libraries were sequenced using a MiSeq v3 600-cycle kit (Illumina) to perform 300-bp paired-end sequencing on a MiSeq instrument (Illumina), according to the manufacturer’s instructions. After the sequencing run, reads with the same index sequences were grouped.

### Sequence data analysis.

The sequencing reads were aligned with the reference HA sequences (ShimH1, HokH2, HKH3, HokH4, ShimH5, and ShimH5 24a2b) determined by Sanger sequencing using Bowtie 2 (49) with default settings. Then the positive-sense reads containing any of 30 nucleotides at the 5′ end of cDNAs were selected from the derived alignments and parsed to count insertions and deletion in the analyzed regions (nucleotide positions from 1036 to 1062 for ShimH1 and HKH3, from 1035 to 1061 for HokH2, from 1020 to 1046 for HokH4, and from 1040 to 1066 for ShimH5 and ShimH5 24a2b) together with filtering the reads containing the nucleotides with low-quality scores (<30) using in-house scripts.

### Prediction of RNA secondary structure.

The Quickfold program (http://unafold.rna.albany.edu/?q=DINAMelt/Quickfold) (50) and RNAfold program from the ViennaRNA Web Services (http://rna.tbi.univie.ac.at/) and the ViennaRNA package (51) were used to predict the secondary structures of the RNAseqHAclv region. The script (ct2b.pl) in the ViennaRNA package was employed to convert RNA structures from “connect” to “dot-parenthesis” format. The ShimH1, HokH2, HKH3, HokH4, and ShimH5 sequences and all full-length HA sequences (limited to low-pathogenic avian influenza viruses isolated from ducks [H1 to H13] and all avian species [H14 to H16]) available in The NCBI Influenza Virus Resource Database were used for the analysis. In case 2 or more viruses had identical sequences in the analyzed region, the HA sequence of the earliest isolate was used as a representative.
