# Supplementary figures and images for: Genetic Predisposition To Acquire a Polybasic Cleavage Site for Highly Pathogenic Avian Influenza Virus Hemagglutinin
Source: mBio. 2017 Feb 14;8(1):e02298-16. doi: 10.1128/mBio.02298-16 (PMC5312086; doi:10.1128/mBio.02298-16)

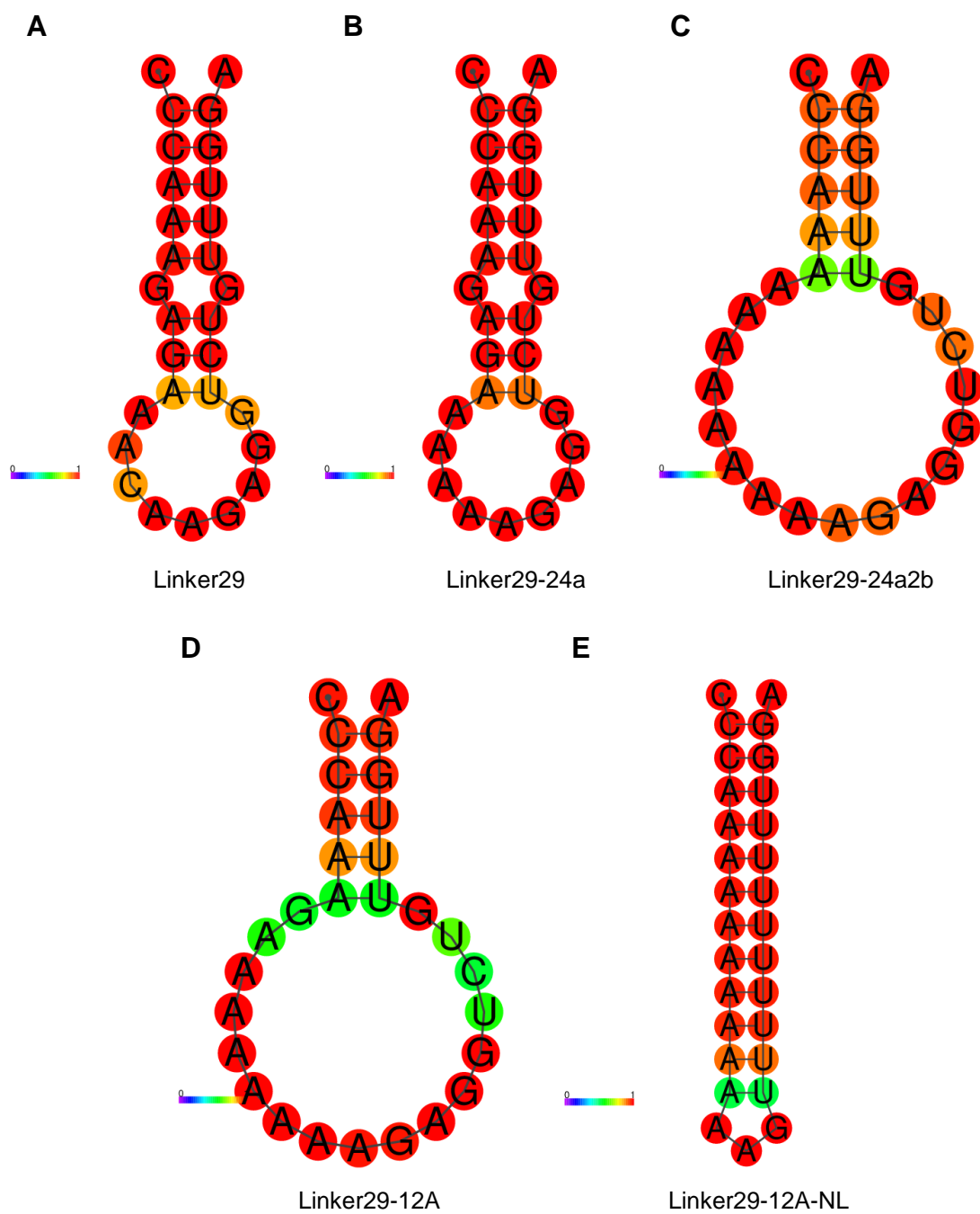

Supplement: FIG S1 [file mbo001173195sf1.pdf]

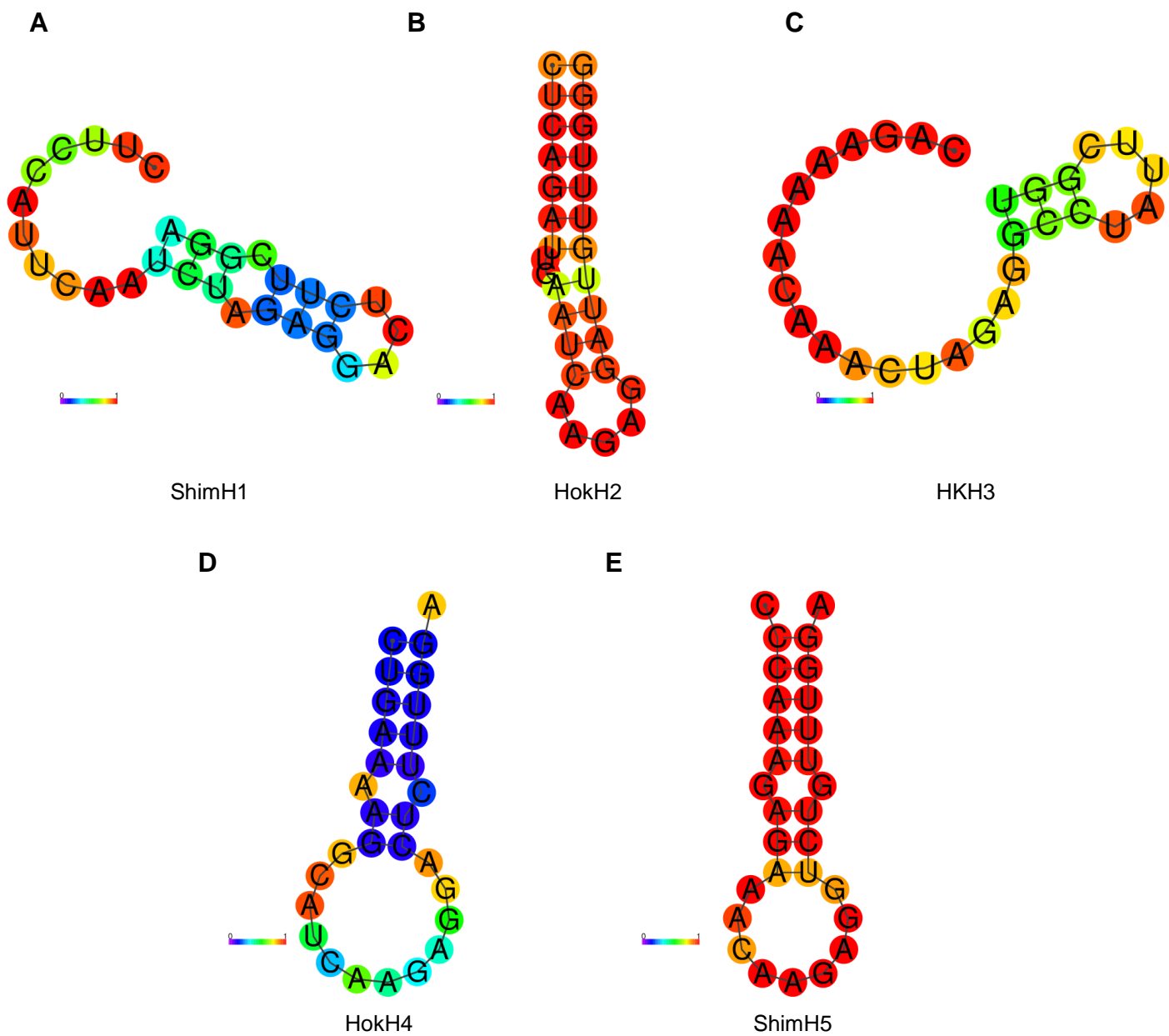

Supplement: FIG S3 [file mbo001173195sf3.pdf]
