# Supplementary material for: Genetic Predisposition To Acquire a Polybasic Cleavage Site for Highly Pathogenic Avian Influenza Virus Hemagglutinin
Source: mBio. 2017 Feb 14;8(1):e02298-16. doi: 10.1128/mBio.02298-16 (PMC5312086; doi:10.1128/mBio.02298-16)
Supplement: FIG S2 [file mbo001173195sf2.pdf]

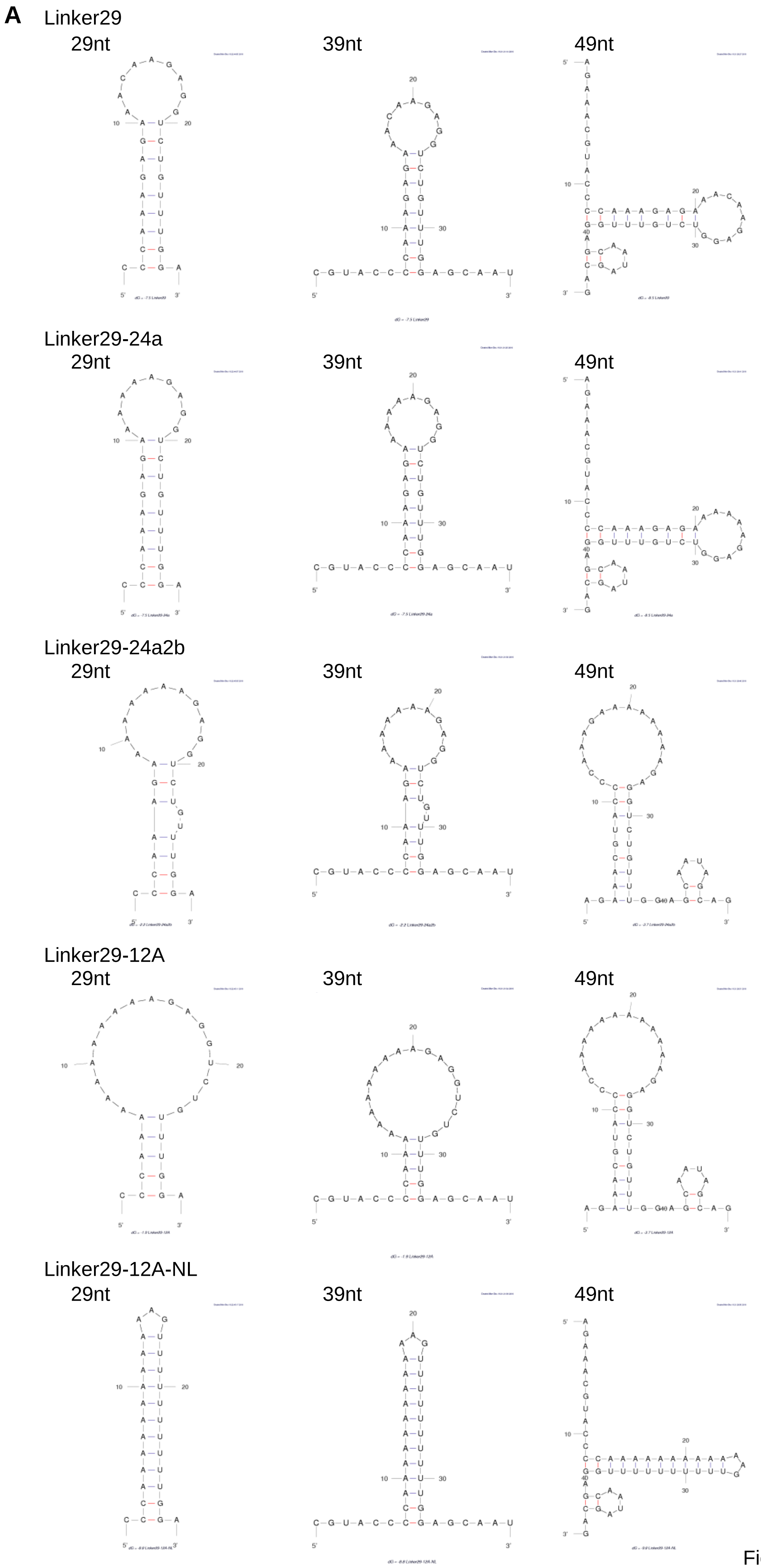

Fig. S2. Nao *et al.*

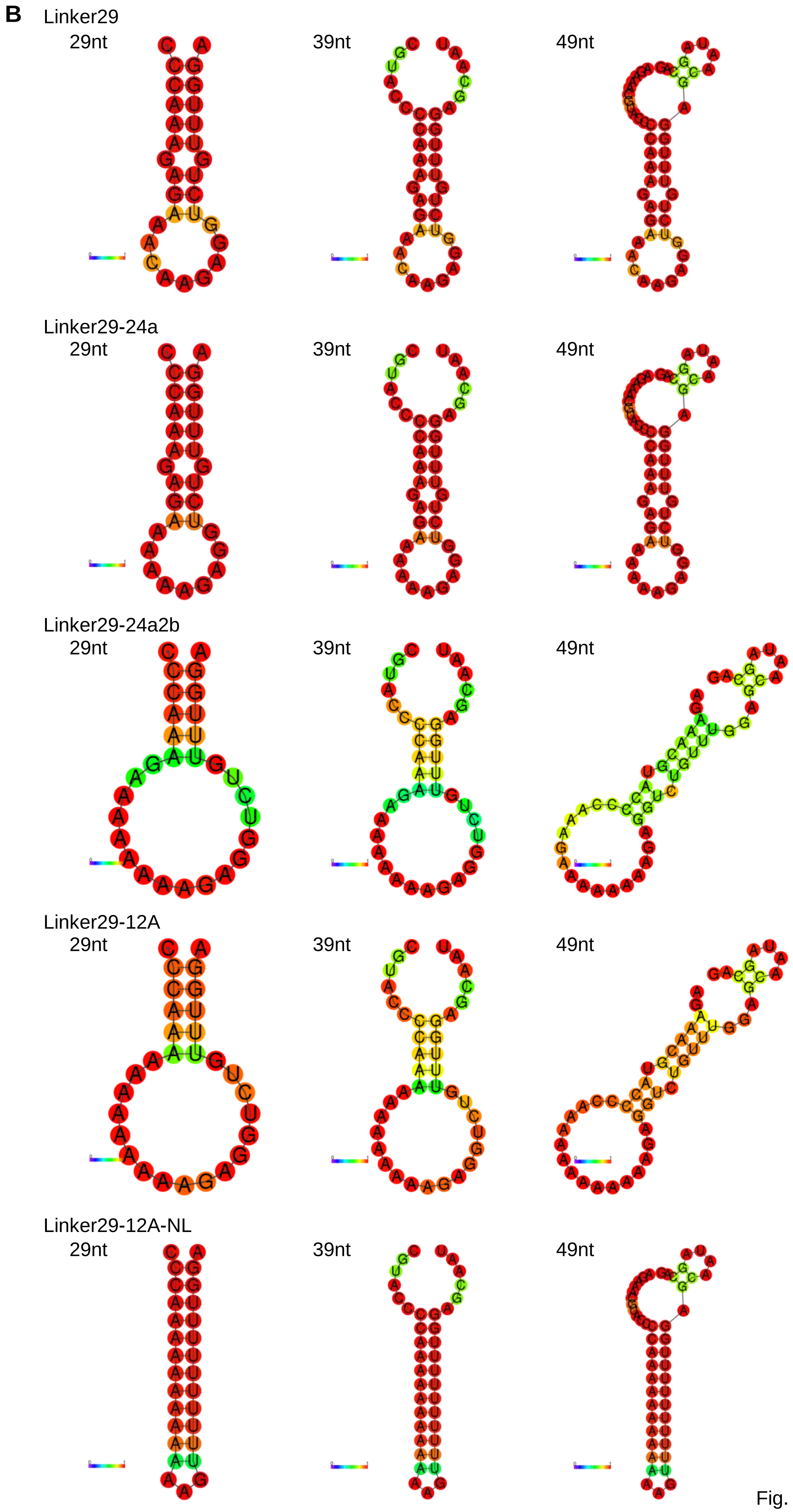

Fig. S2. Nao *et al*.
