# Supplementary material for: Genetic Predisposition To Acquire a Polybasic Cleavage Site for Highly Pathogenic Avian Influenza Virus Hemagglutinin
Source: mBio. 2017 Feb 14;8(1):e02298-16. doi: 10.1128/mBio.02298-16 (PMC5312086; doi:10.1128/mBio.02298-16)
Supplement: FIG S4 [file mbo001173195sf4.pdf]

Output of sir_graph (©)
mfold_util 4.7

Created Thu Mar 17 08:09:38 2016

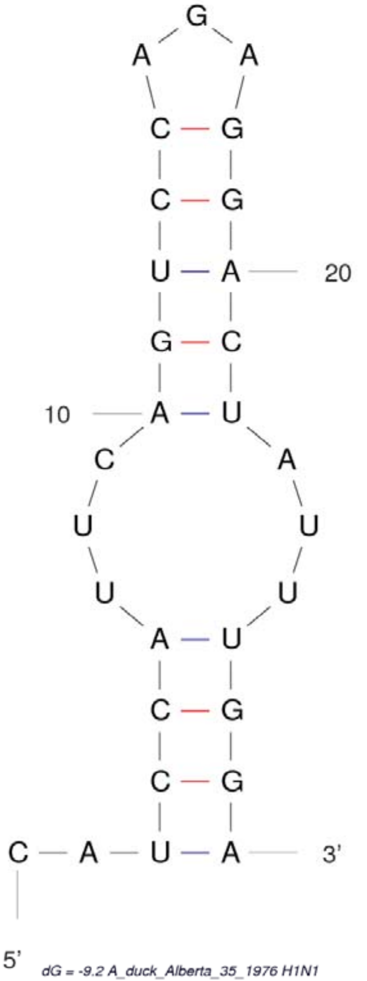

dG = -9.2 A_duck_Alberta_35_1976 H1N1

Output of sir_graph (©)
mfold_util 4.7

Created Thu Mar 17 08:09:38 2016

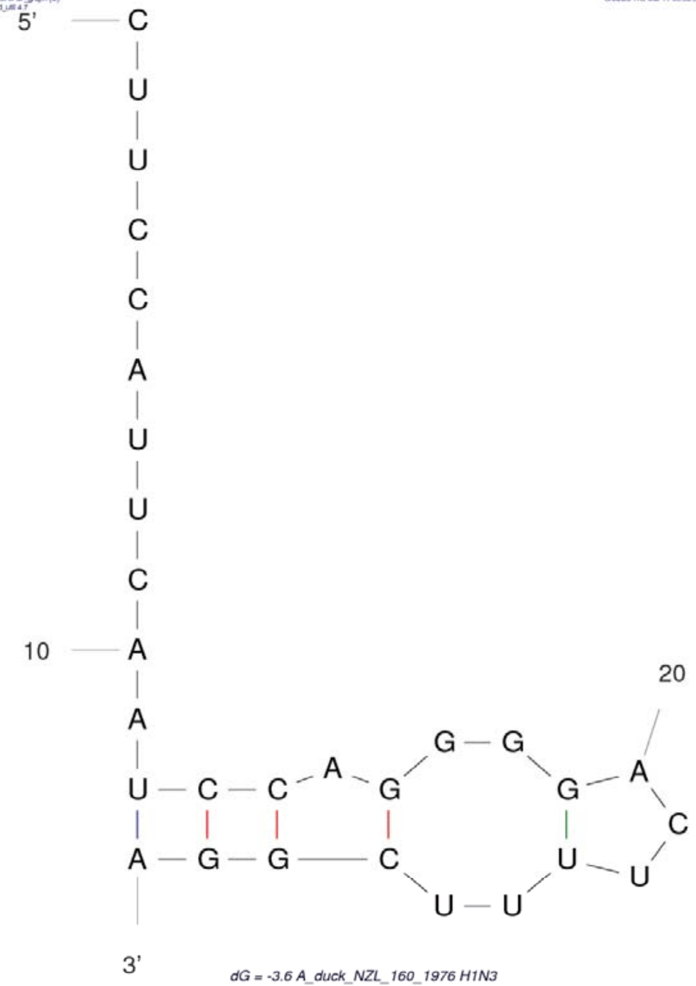

dG = -3.6 A_duck_NZL_160_1976 H1N3

Output of sir_graph (©)
mfold_util 4.7

Created Thu Mar 17 08:09:38 2016

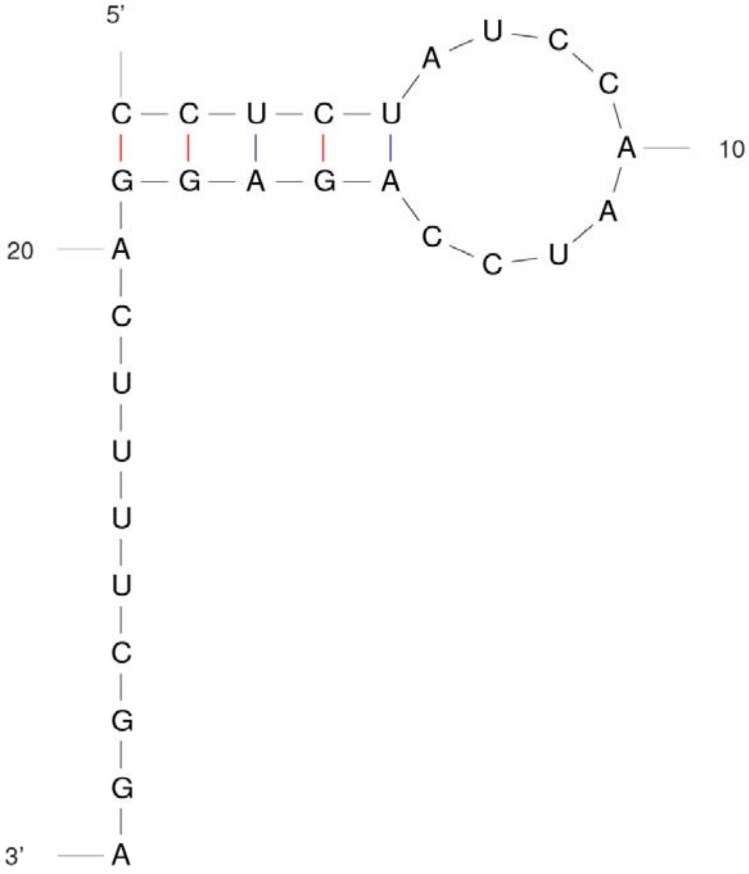

dG = -4.9 A_duck_Bavaria_1_1977 H1N1

Output of sir_graph (©)
mfold_util 4.7

Created Thu Mar 17 08:09:39 2016

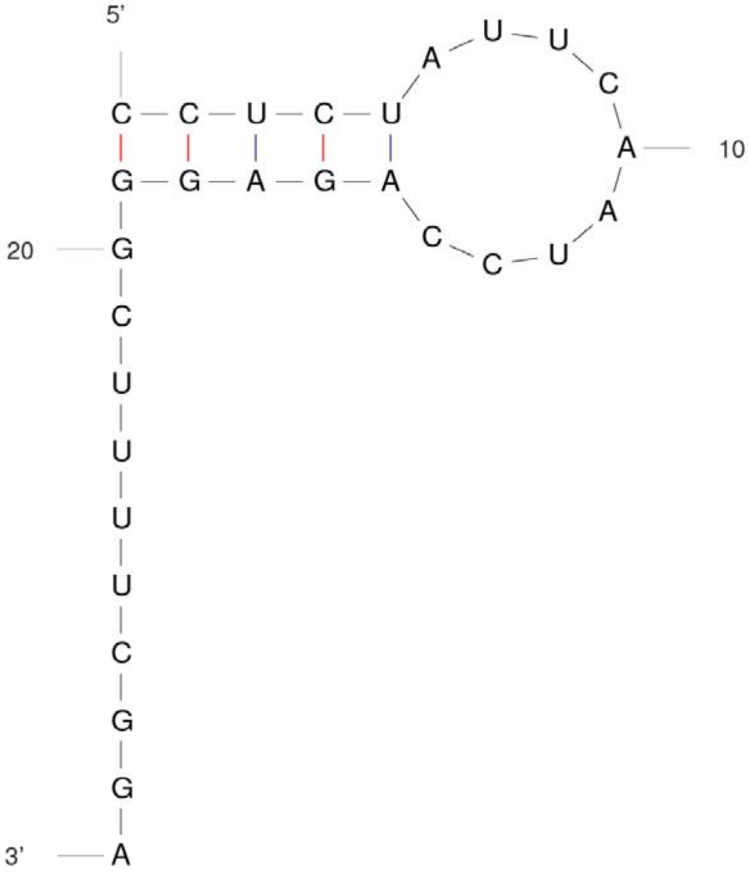

dG = -5.1 A_duck_HK_196_1977 H1

Output of sir_graph (©)
mfold_util 4.7

Created Thu Mar 17 08:09:39 2016

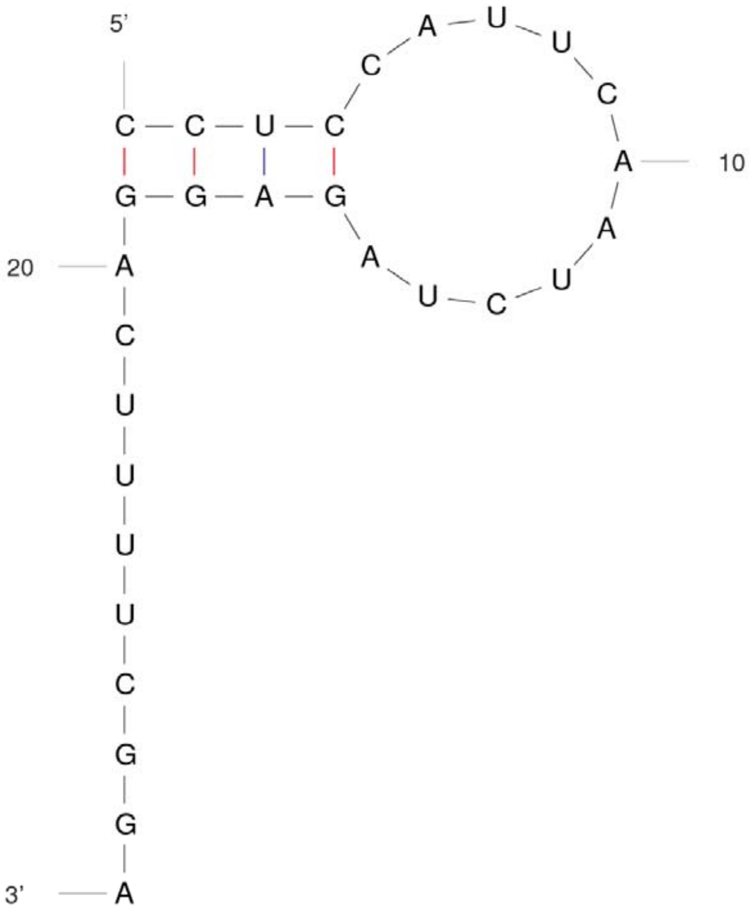

dG = -3.3 A_duck_Miyagi_66_1977 H1N1

Output of sir_graph (©)
mfold_util 4.7

Created Thu Mar 17 08:09:39 2016

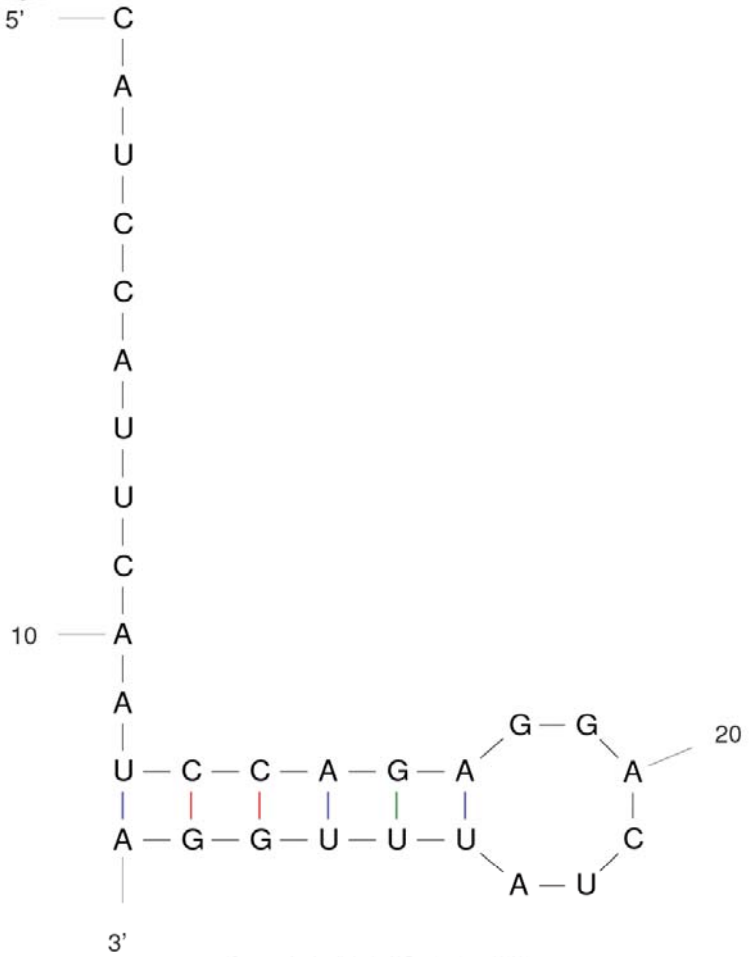

dG = -5.2 A_pintail duck_ALB_219_1977 H1N1

Output of sir_graph (©)
mfold_util 4.7

Created Thu Mar 17 08:09:39 2016

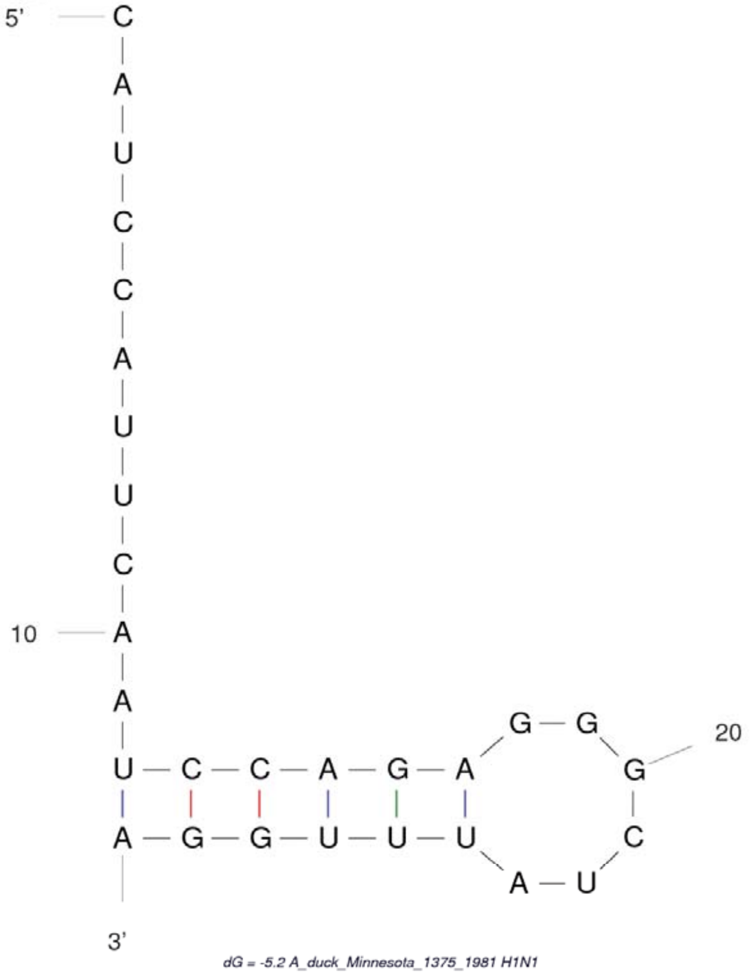

dG = -5.2 A_duck_Minnesota_1375_1981 H1N1

Output of sir_graph (©)
mfold_util 4.7

Created Thu Mar 17 08:09:39 2016

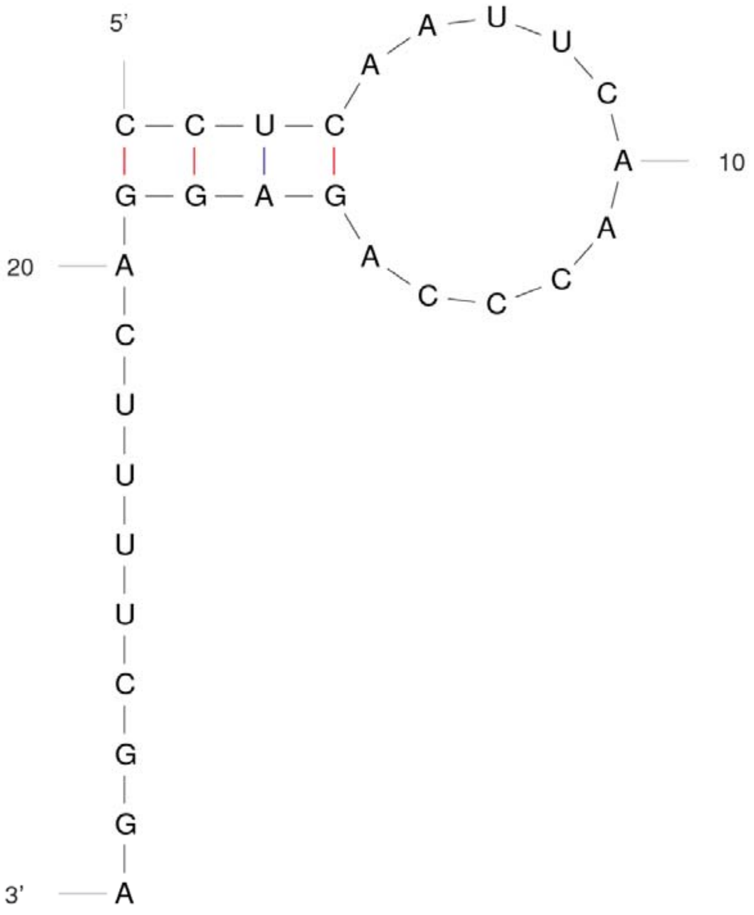

dG = -3.8 A_duck_Victoria_23_1981 H1N1

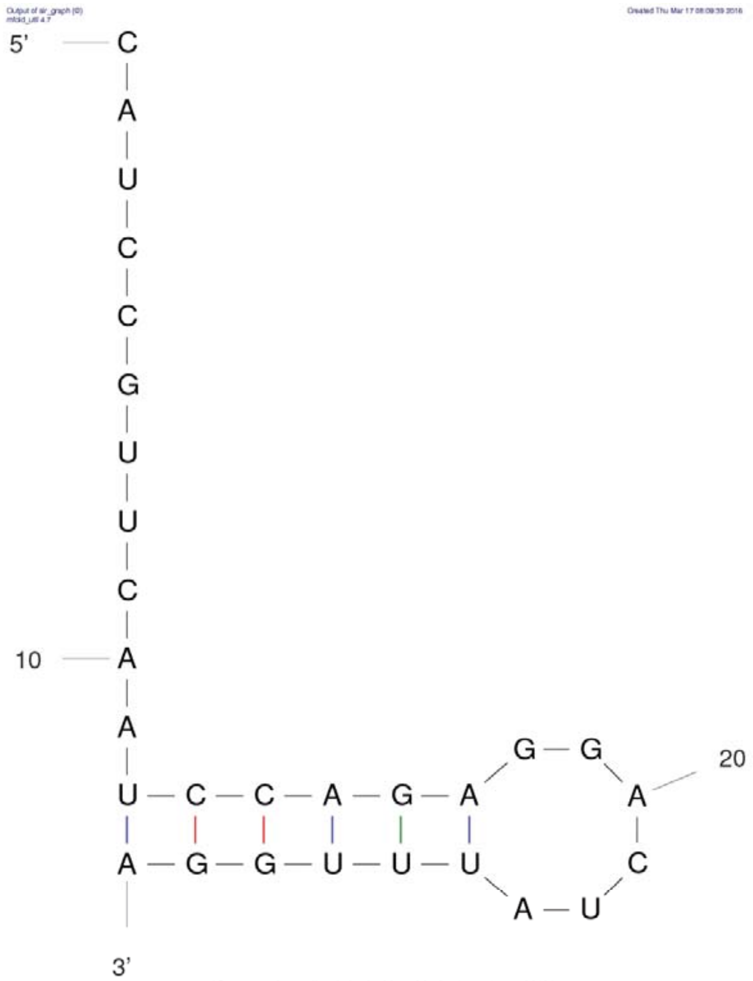

dG = -5.2 A_mallard duck_New York_170_1982 H1N2

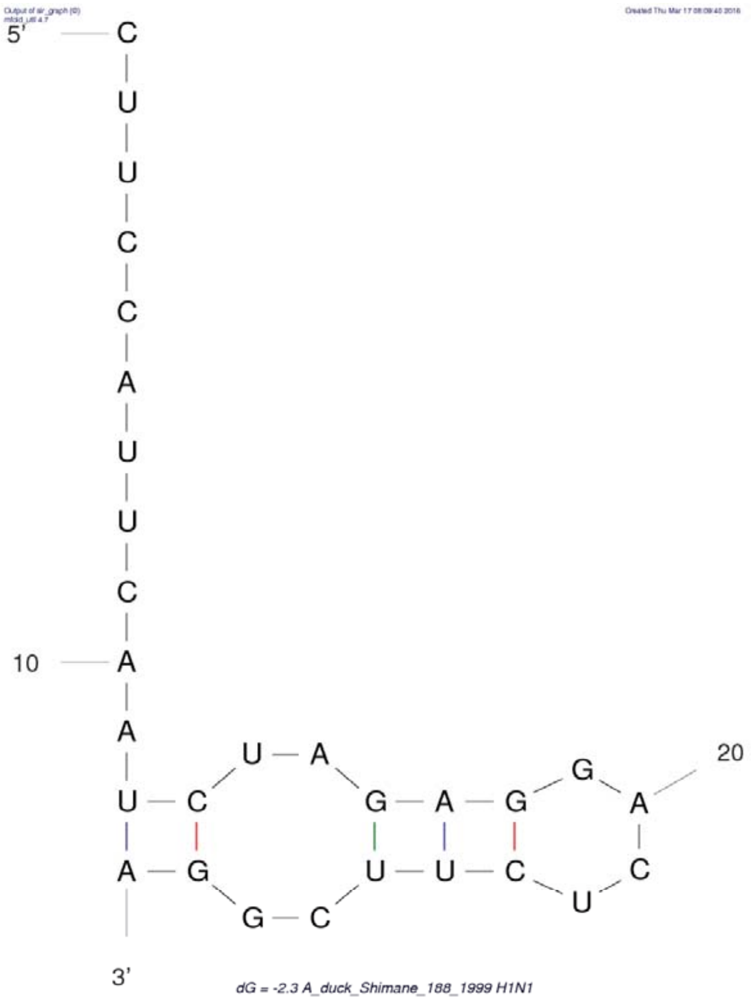

dG = -2.3 A_duck_Shimane_188_1999 H1N1

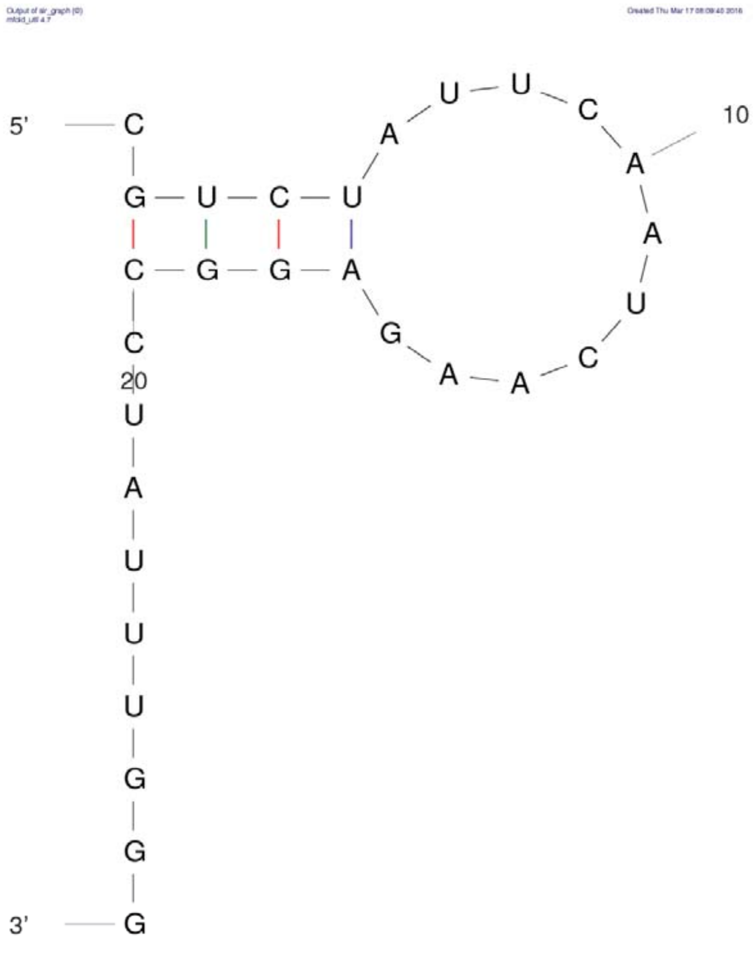

dG = -1.2 A_duck_NC_91347_01 H1N2

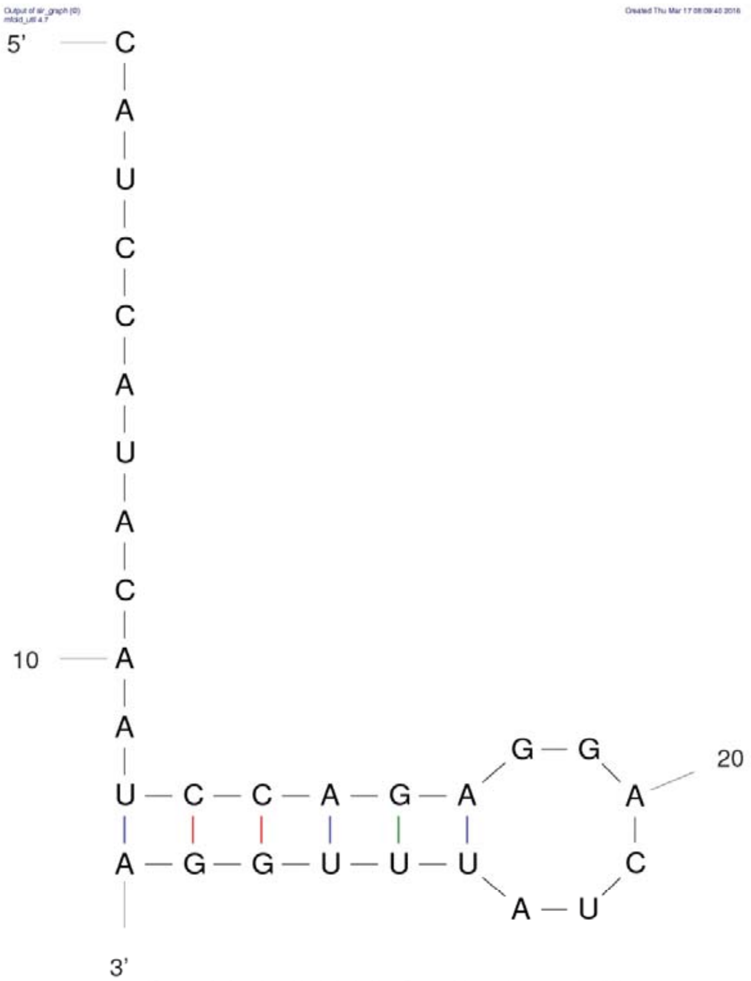

dG = -5.2 A_American black duck_New Brunswick_02715_2007 mixedH1

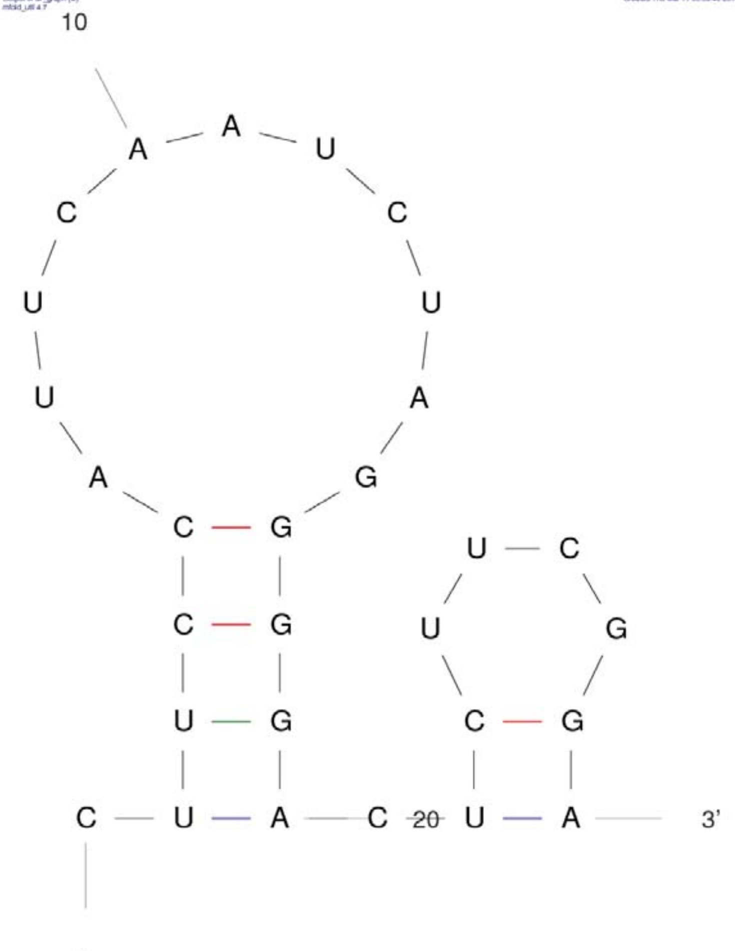

dG = -2.2 A_duck_Korea_U11-1_2007 H1N2

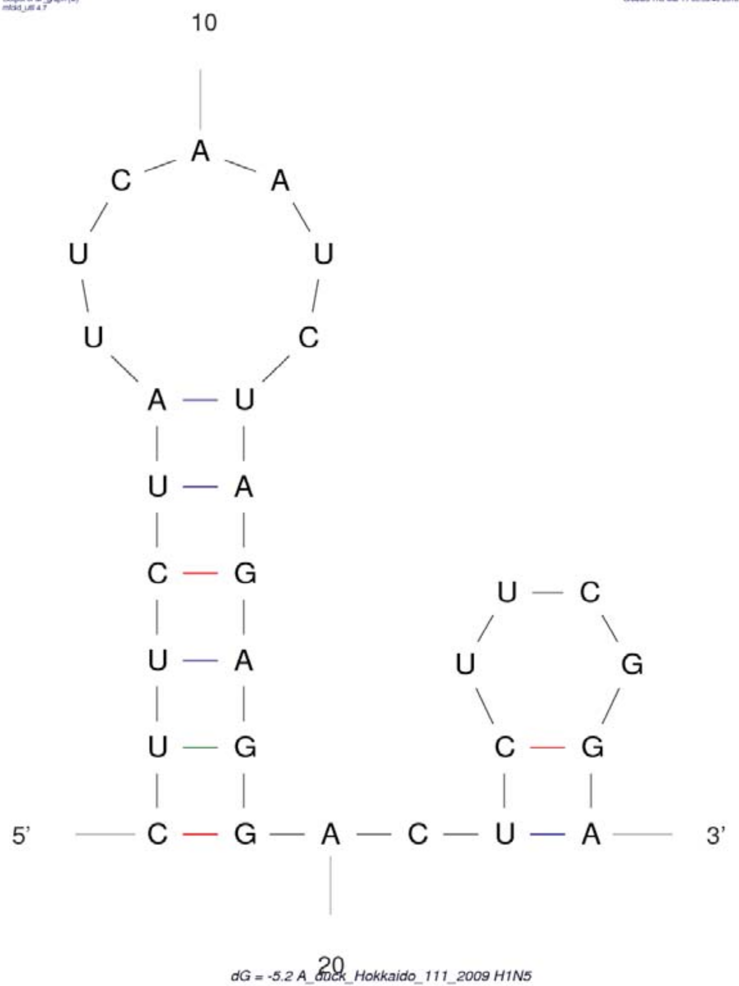

dG = -5.2 A_duck_Hokkaido_111_2009 H1N5

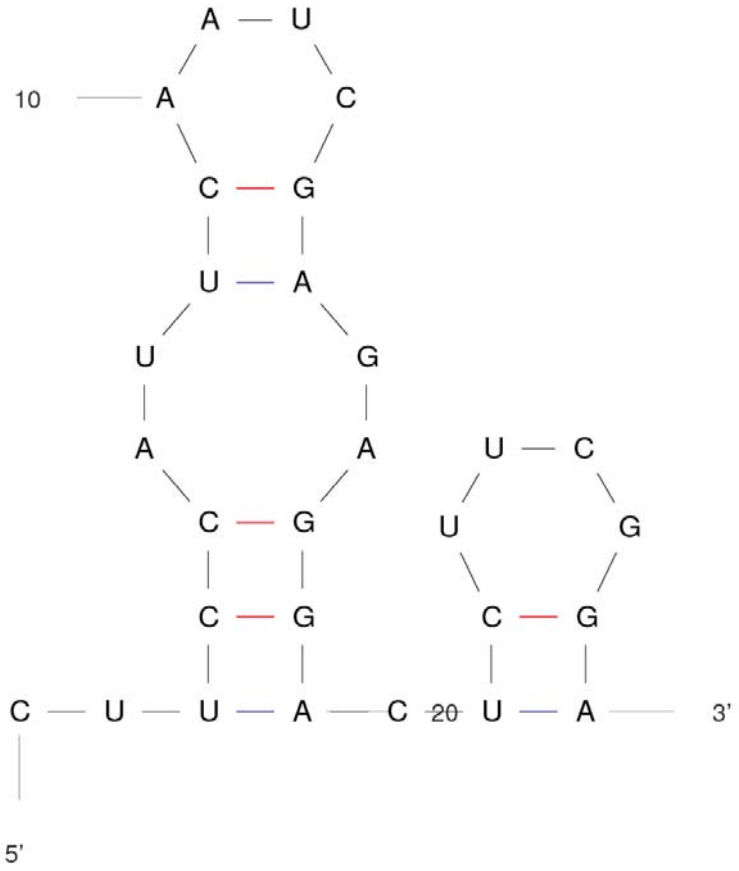

dG = -3.7 A_duck_Korea_372_2009 H1N1

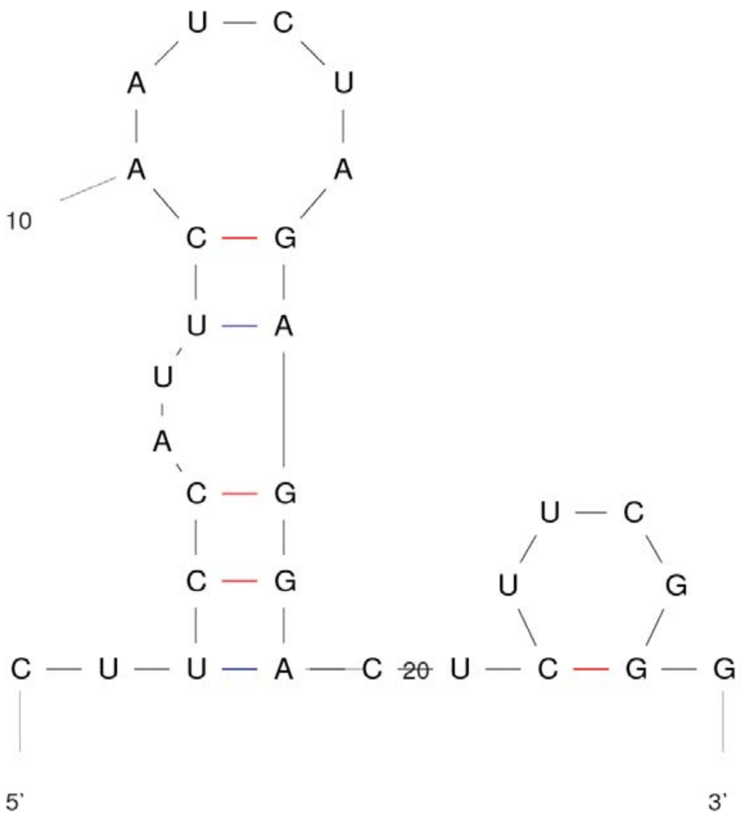

dG = -1.6 A_duck_Nanjing_19_2010 H1N3

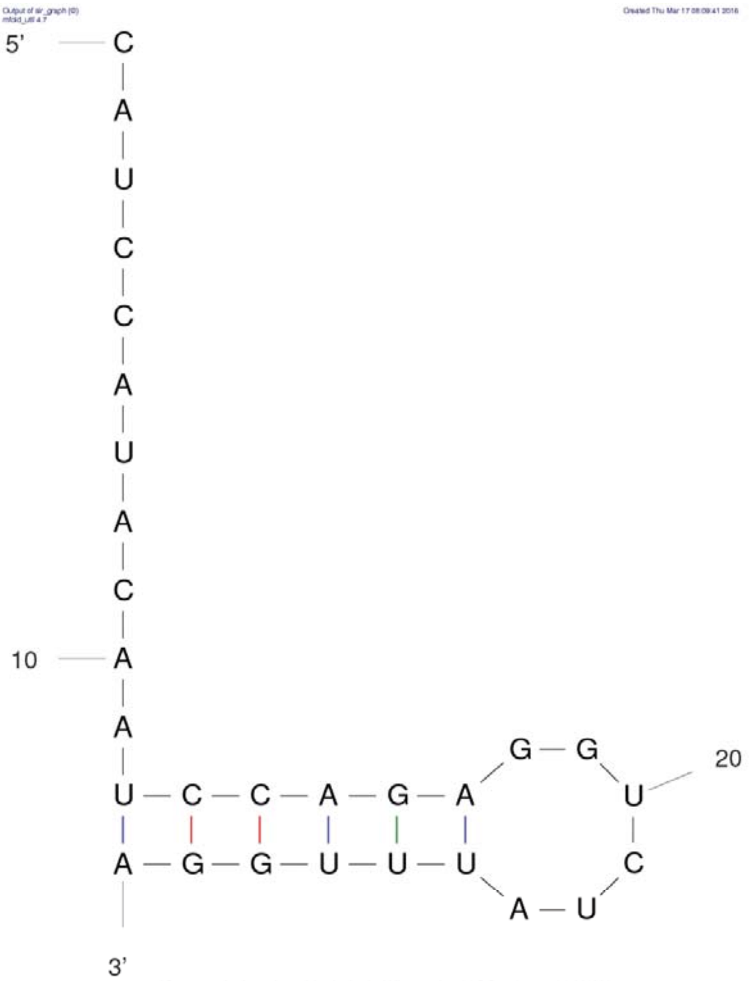

dG = -5.2 A_American black duck_Wisconsin_11OS3016_2011 H1N1

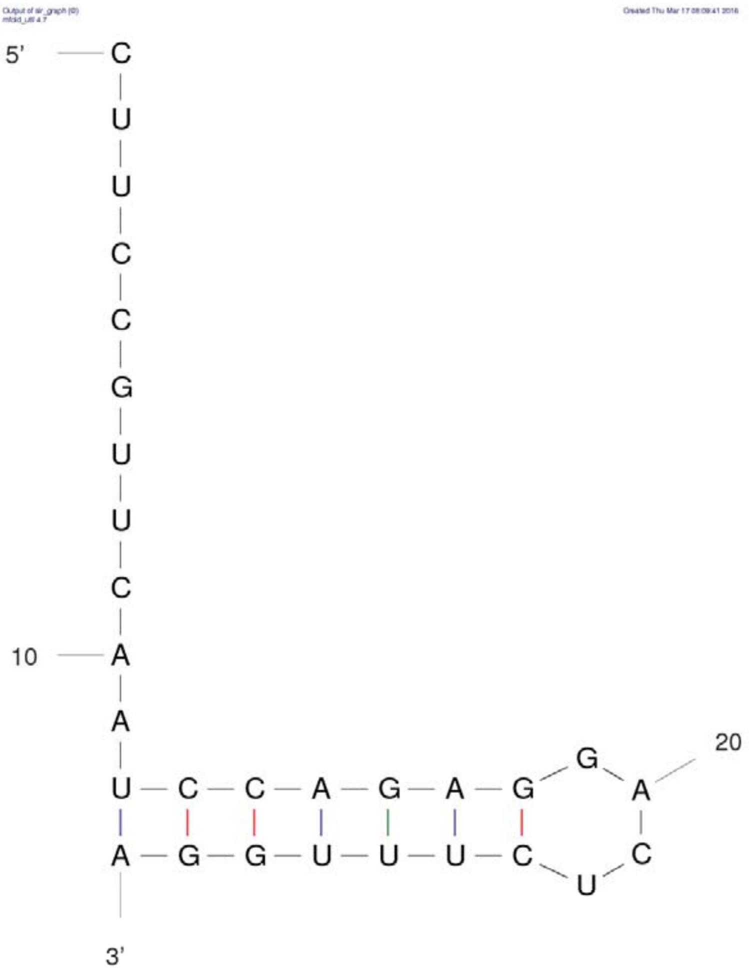

dG = -7.2 A_duck_Zhejiang_0607-13_2011 H1N2

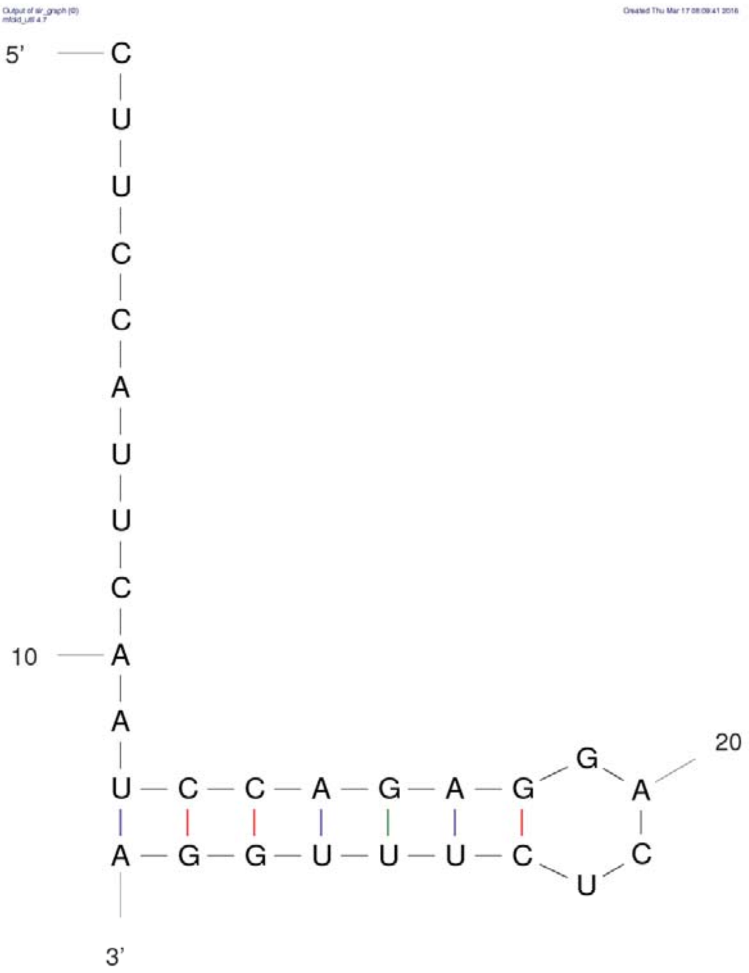

dG = -7.2 A_duck_Zhejiang_0611-8_2011 H1N3

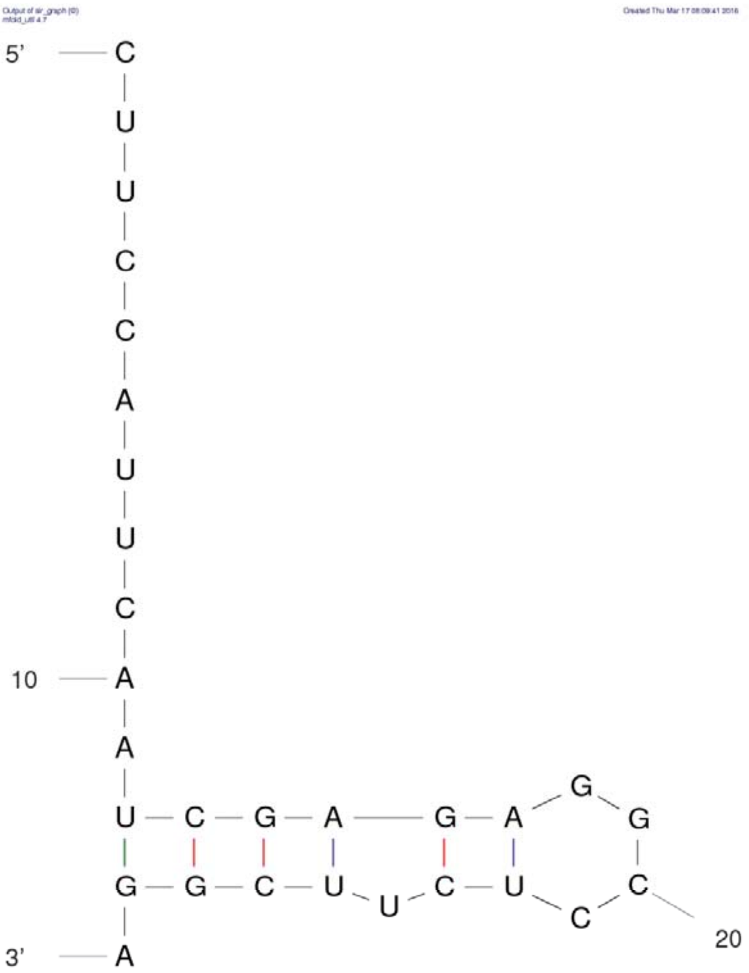

dG = -2.9 A_shelduck_Yasnopolyanske_4-08-02_2011 H1N1

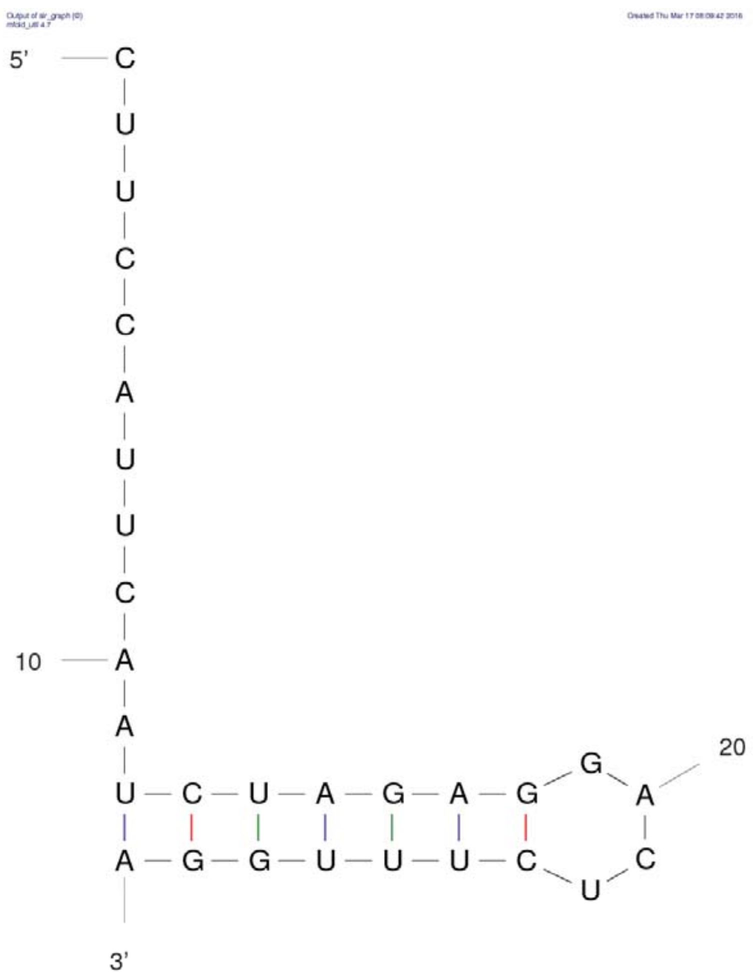

dG = -4.9 A_duck_Hunan_HN2203_2013 H1N3

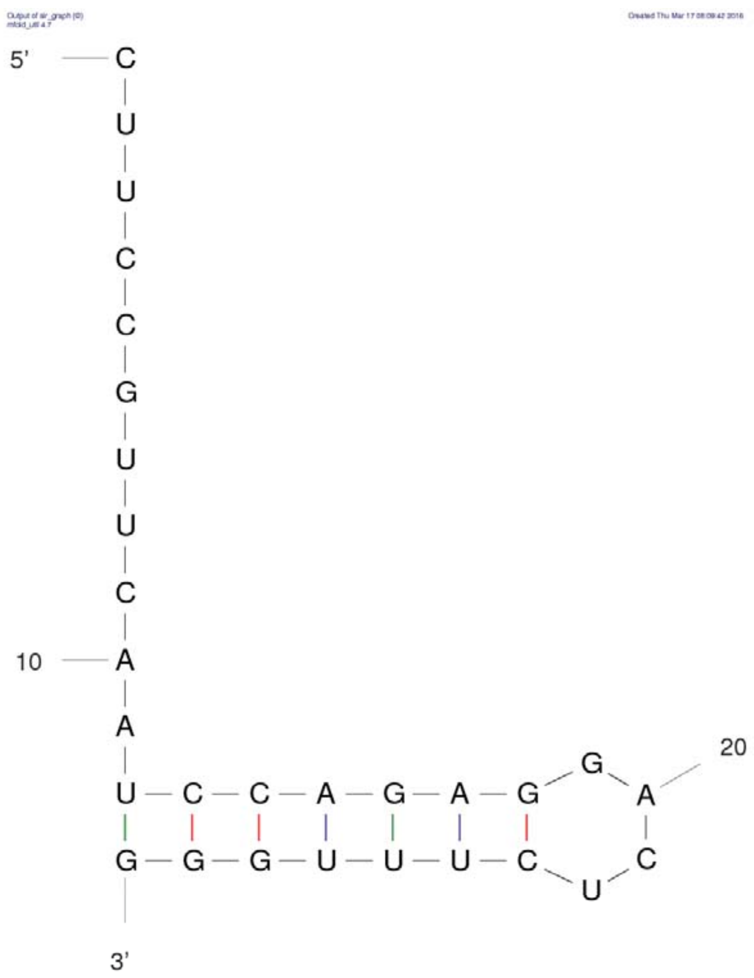

dG = -6.3 A_duck_Wenzhou_YJYF78_2015 H1N4

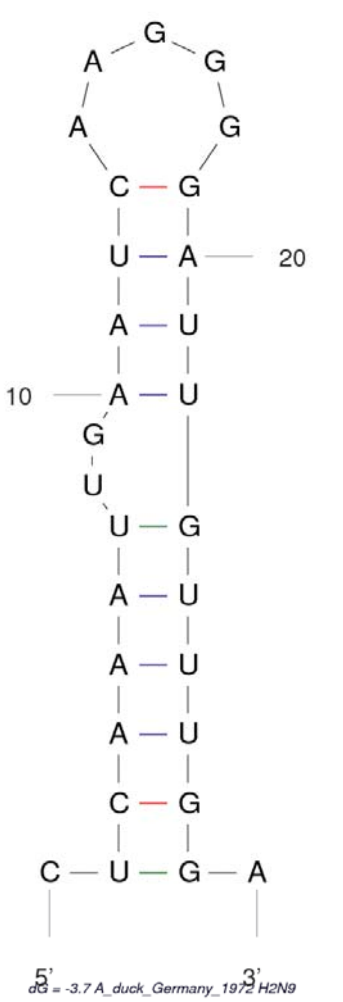

dG = -3.7 A_duck_Germany_1972 H2N9

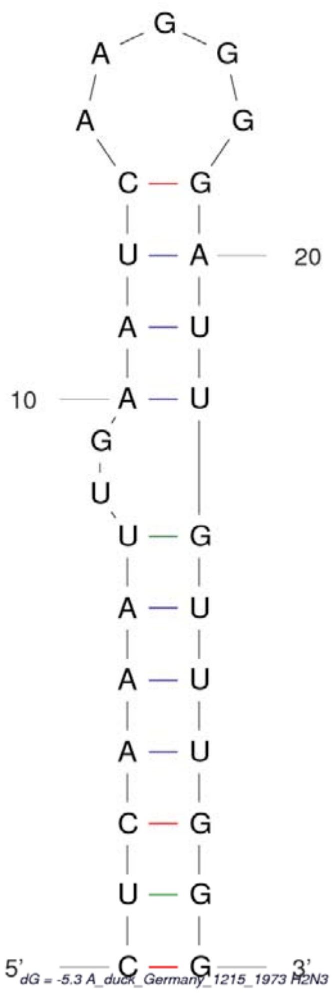

dG = -5.3 A_duck_Germany_1215_1973 H2N3

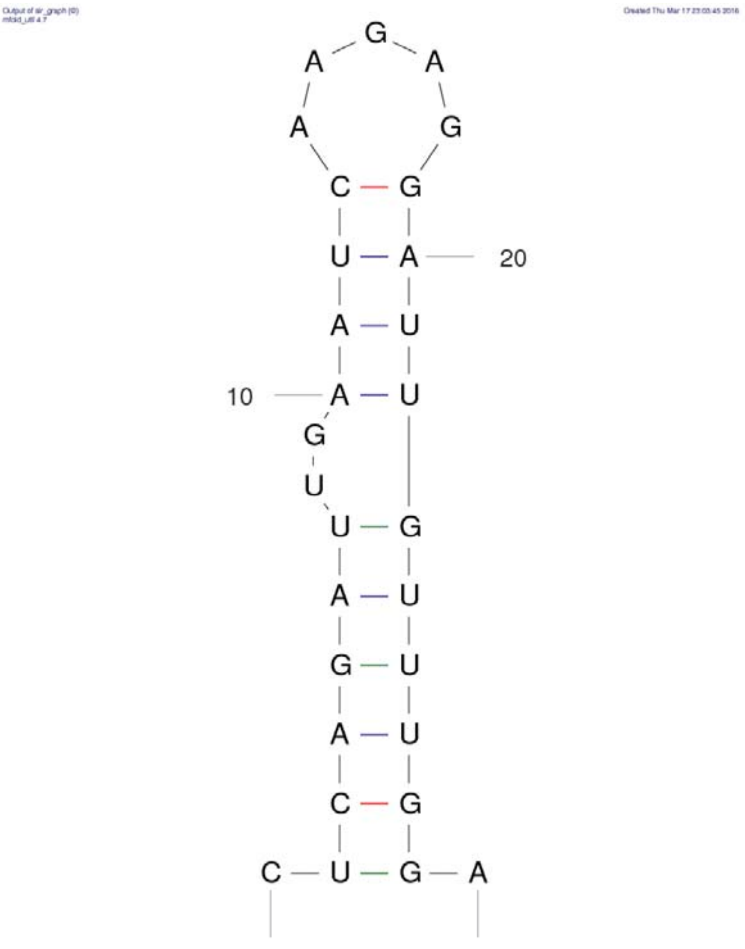

dG = -3.8 A_black duck_New Jersey_1580_1978 H2N3

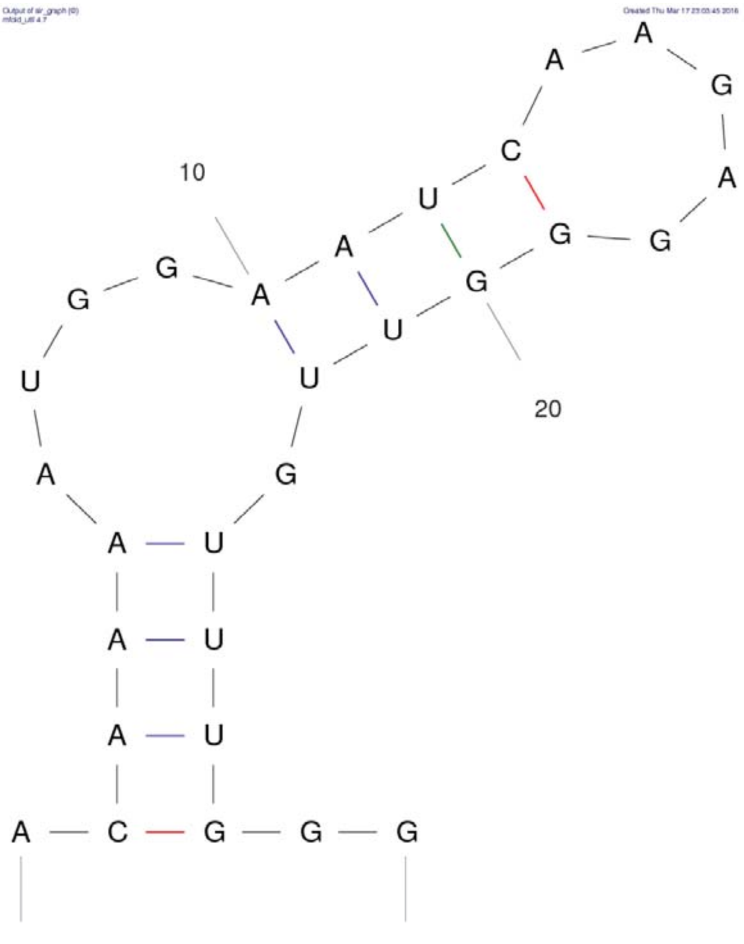

dG = -0.4 A_duck_Hong Kong_273_1978 H2N2

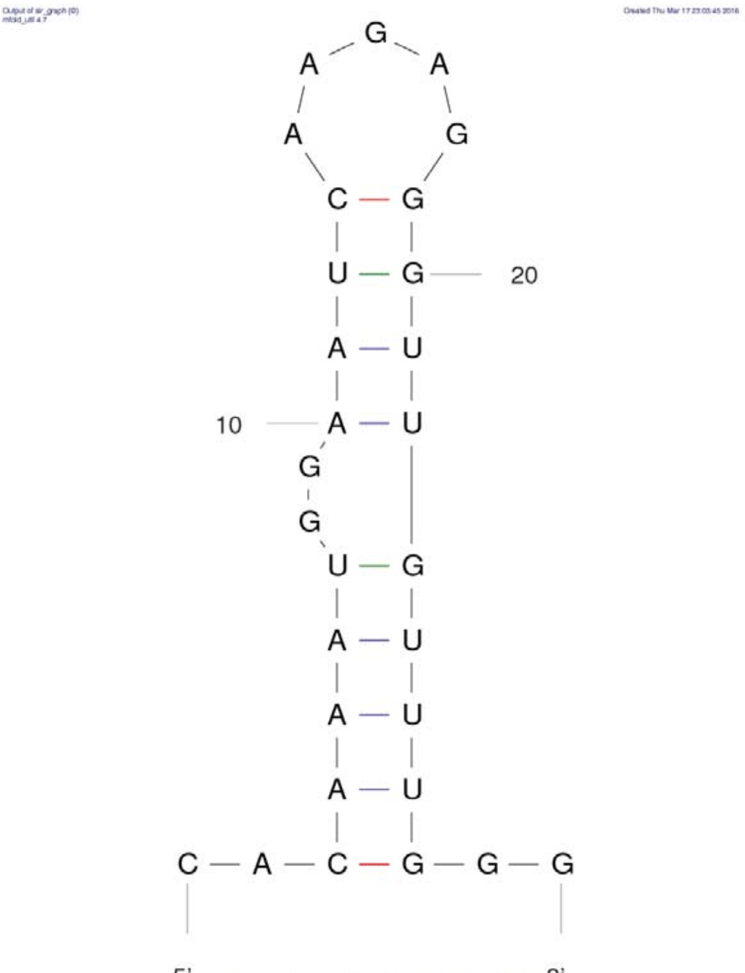

dG = -2.7 A_duck_Hong Kong_273_1978 H2N22

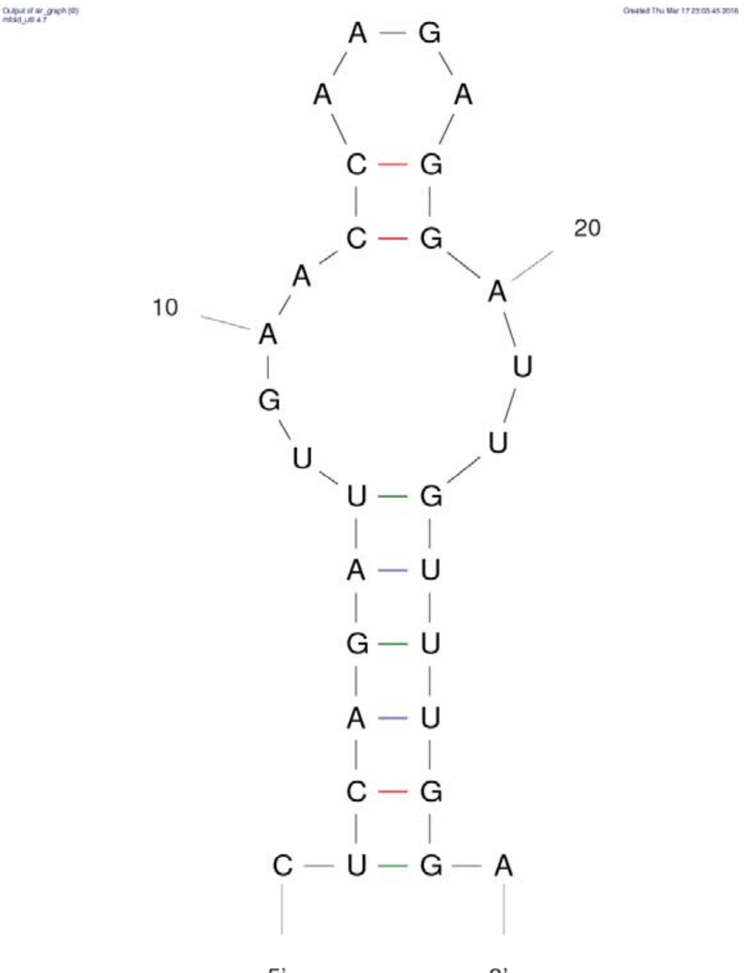

dG = -3.9 A_pintail duck_Alberta_211_1980 H2N3

Output of sir_graph (©)
mfold_util 4.7

Created Thu Mar 17 23:03:45 2016

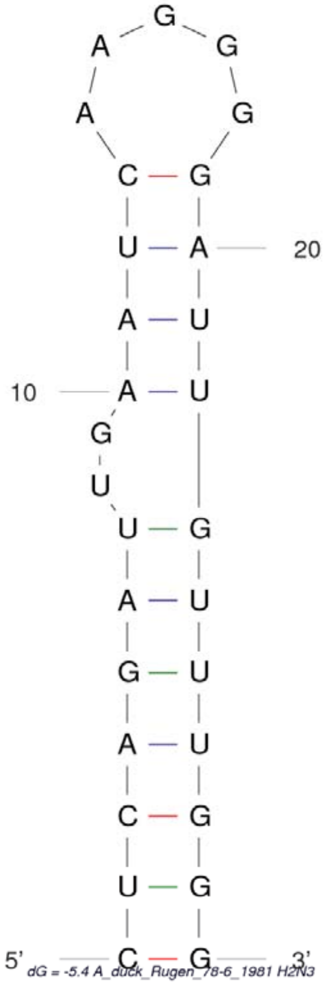

dG = -5.4 A_duck_Rugen_78-6_1981 H2N3

Output of sir_graph (©)
mfold_util 4.7

Created Thu Mar 17 23:03:45 2016

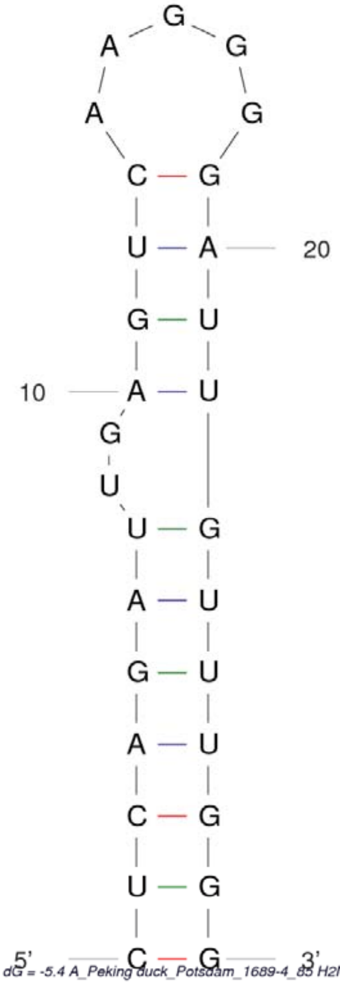

dG = -5.4 A_Peking duck_Potsdam_1689-4_85 H2N3

Output of sir_graph (©)
mfold_util 4.7

Created Thu Mar 17 23:03:46 2016

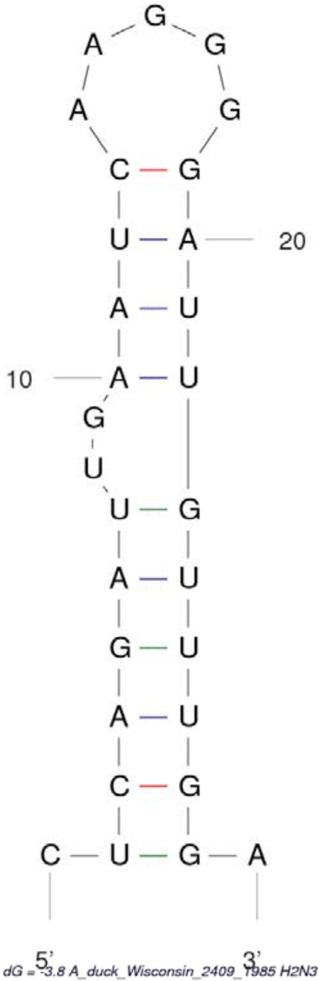

dG = -3.8 A_duck_Wisconsin_2409_1985 H2N3

Output of sir_graph (©)
mfold_util 4.7

Created Thu Mar 17 23:03:46 2016

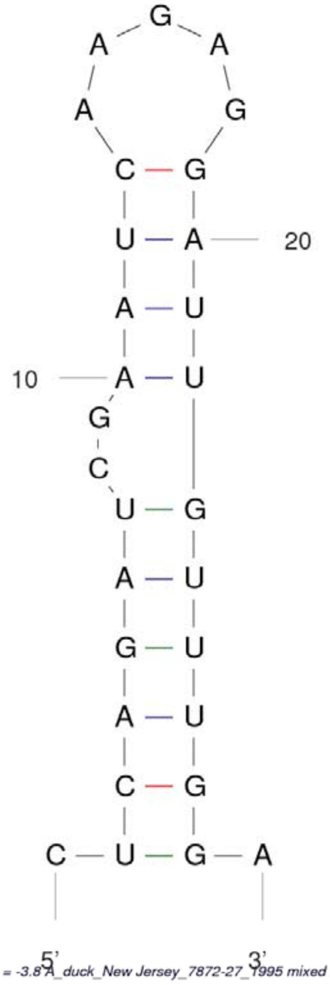

dG = -3.8 A_duck_New Jersey_7872-27_1995 mixed H2

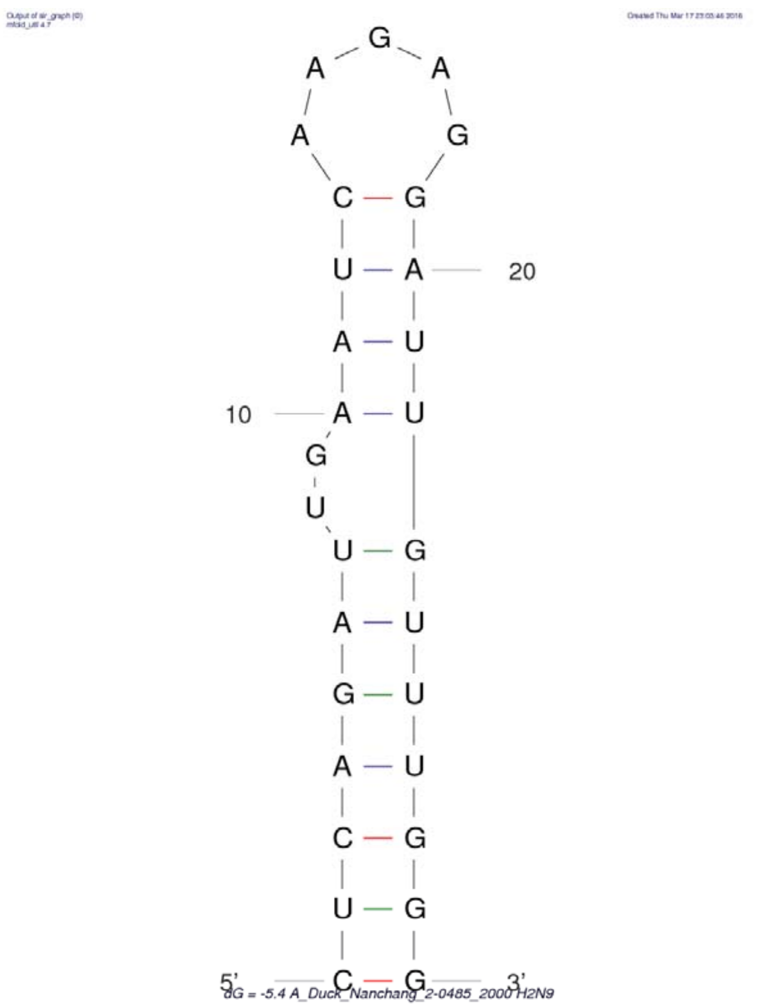
dG = -5.4 A_Duck_Nanchang_2-0485_2000 H2N9

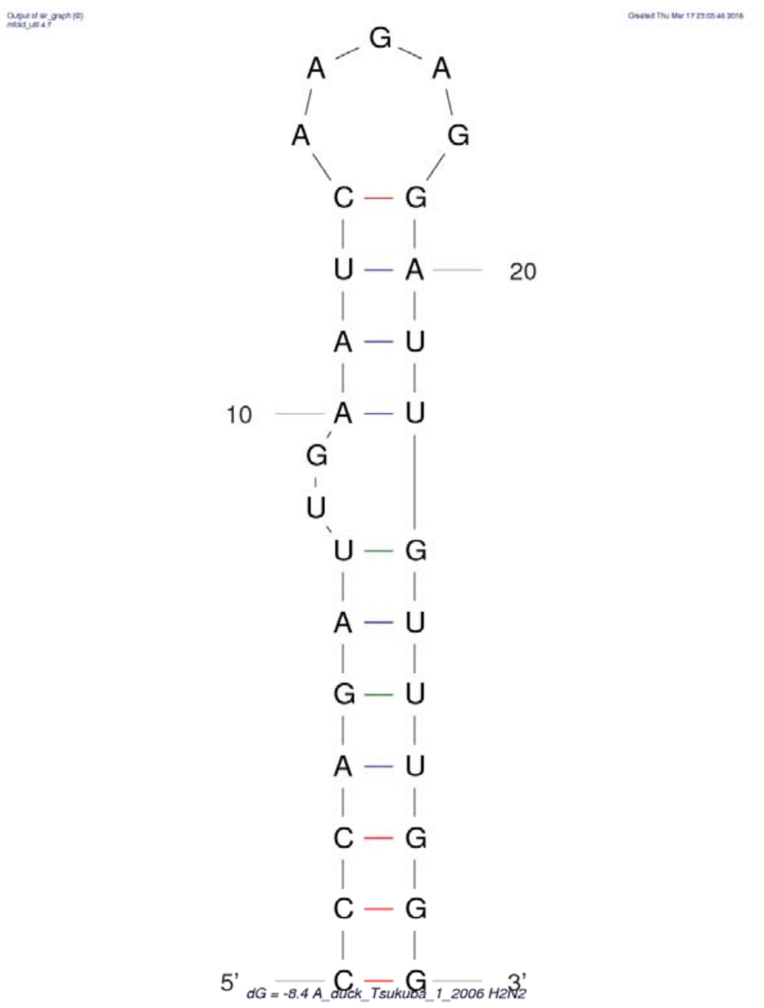
dG = -8.4 A_duck_Tsukuba_1_2006 H2N2

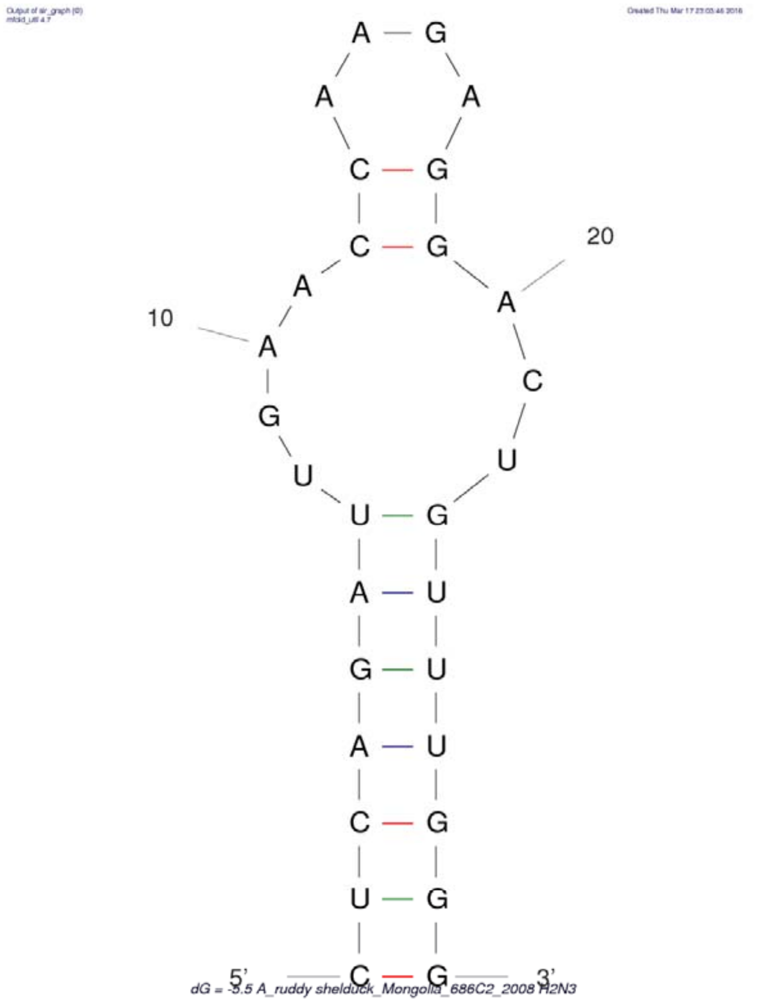
dG = -5.5 A_ruddy shelduck_Mongolia_686C2_2008 H2N3

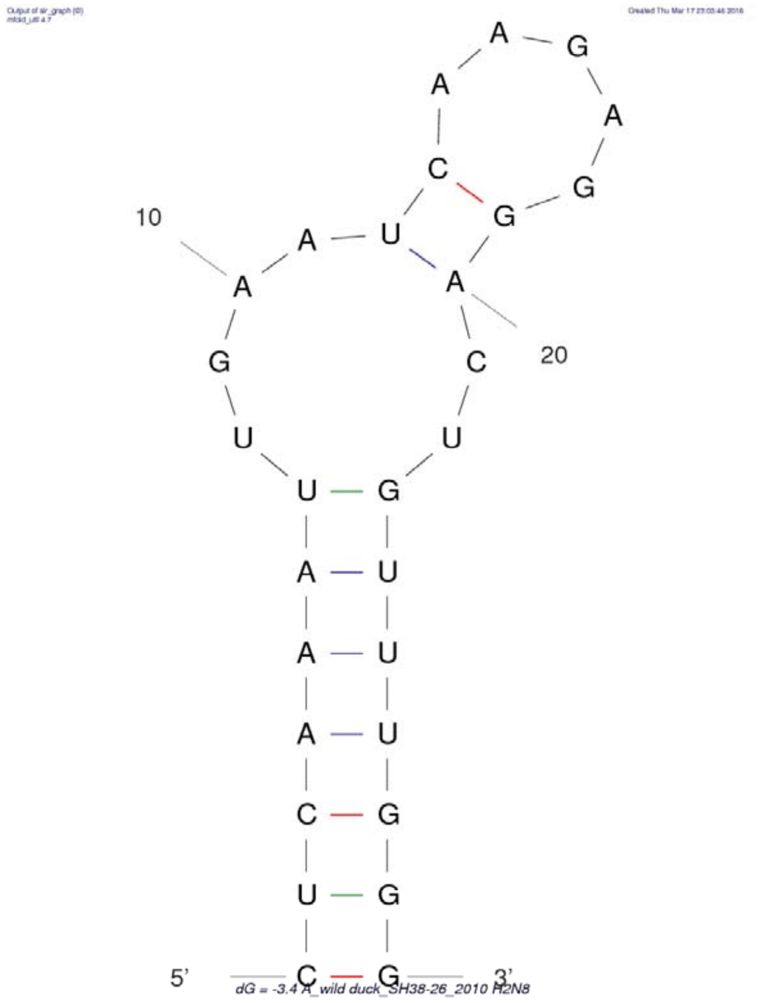
dG = -3.4 A_wild duck_SH38-26_2010 H2N8

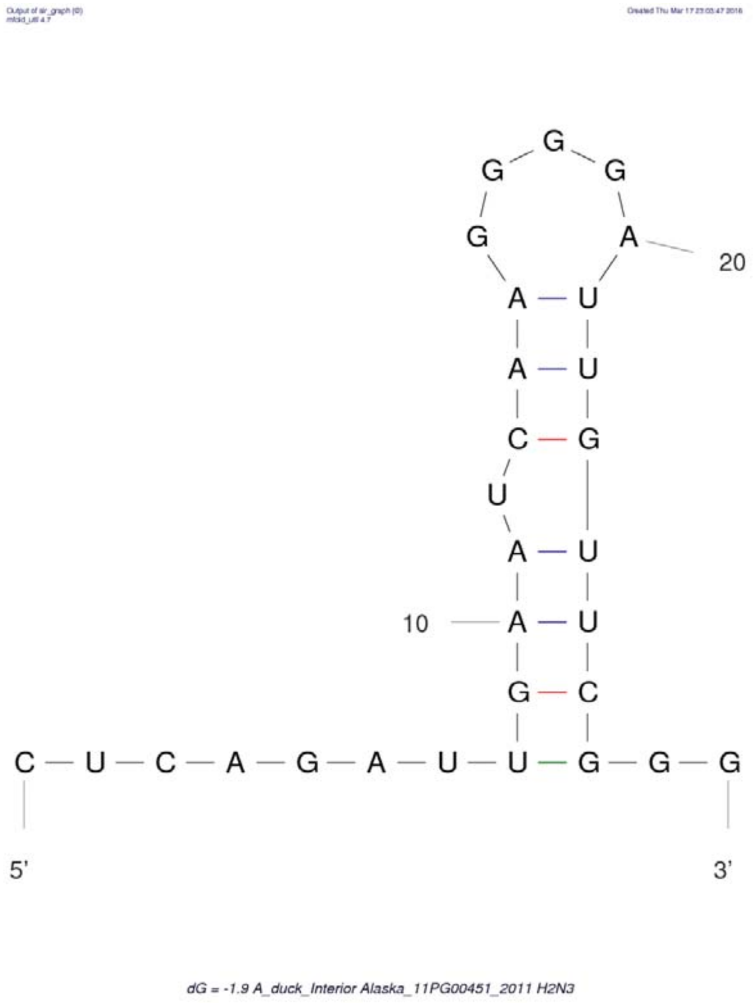
Output of sir_graph (©)
mfold_util 4.7
Created Thu Mar 17 23:03:47 2016
dG = -1.9 A_duck_Interior Alaska_11PG00451_2011 H2N3

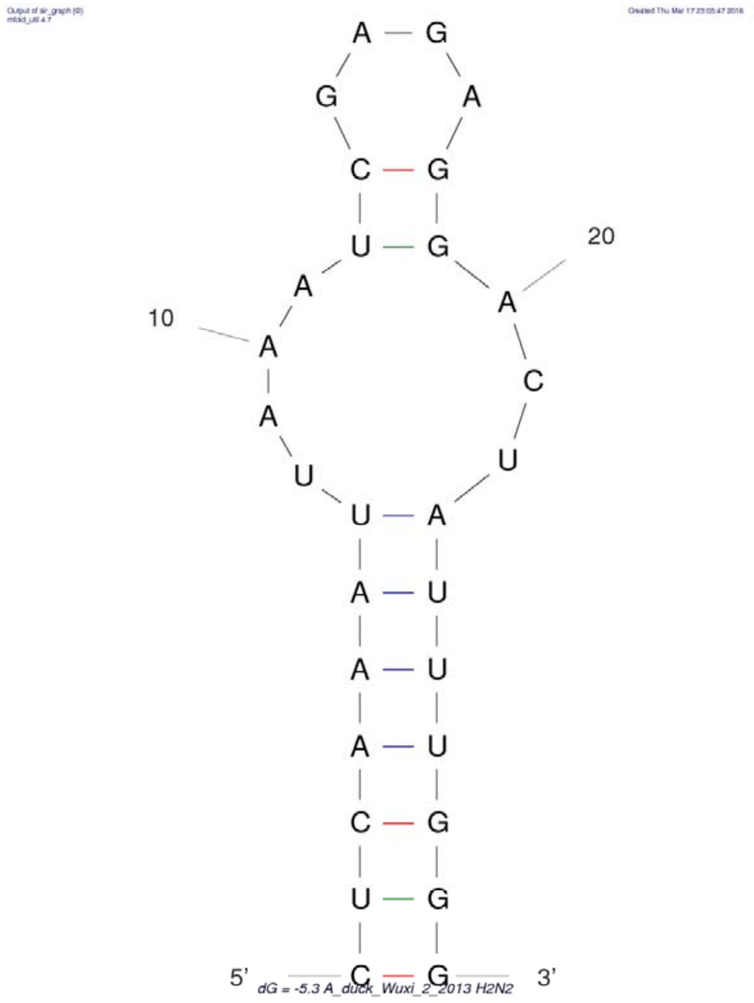
Output of sir_graph (©)
mfold_util 4.7
Created Thu Mar 17 23:03:47 2016
dG = -5.3 A_duck_Wuxi_2_2013 H2N2

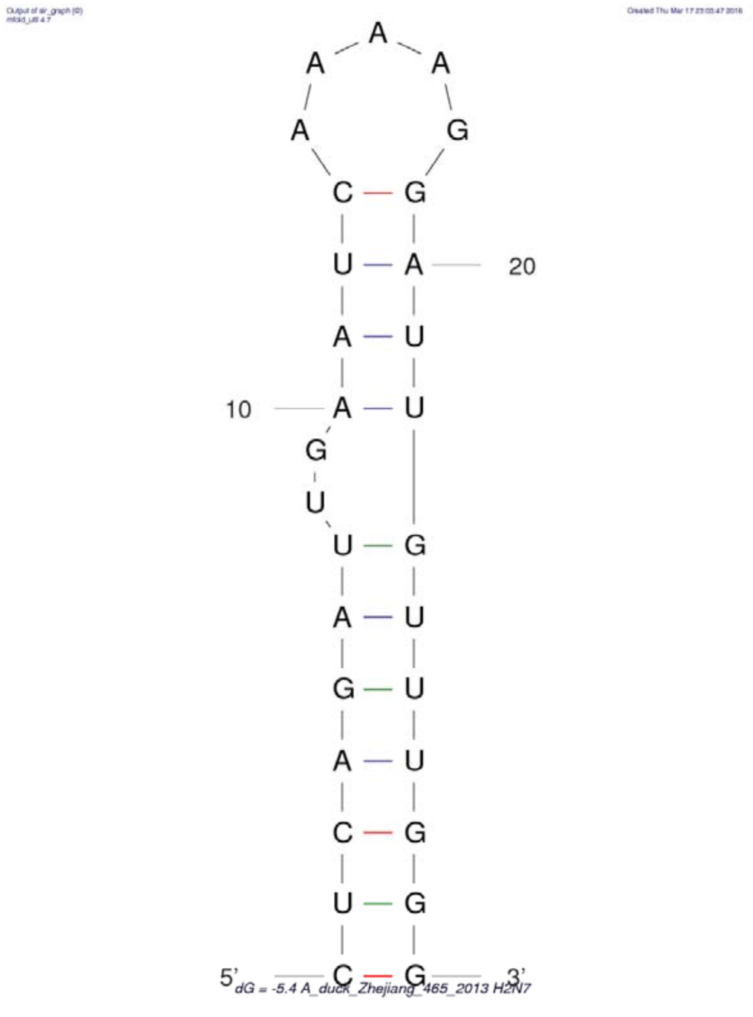
Output of sir_graph (©)
mfold_util 4.7
Created Thu Mar 17 23:03:47 2016
dG = -5.4 A_duck_Zhejiang_465_2013 H2N7

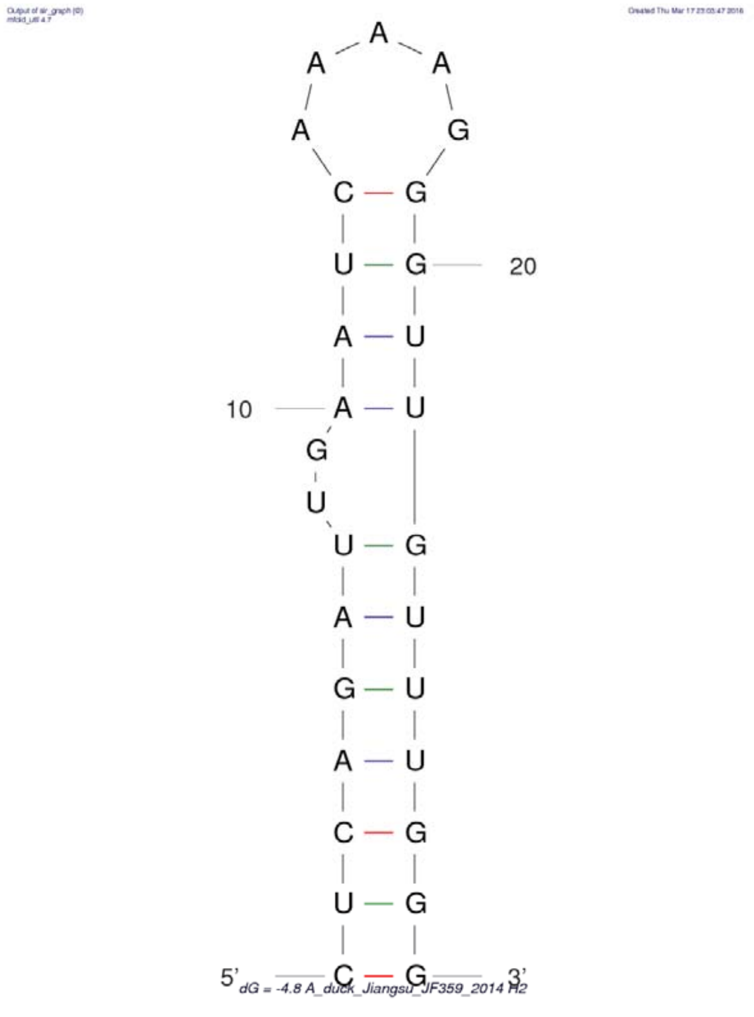
Output of sir_graph (©)
mfold_util 4.7
Created Thu Mar 17 23:03:47 2016
dG = -4.8 A_duck_Jiangsu_JF359_2014 H2

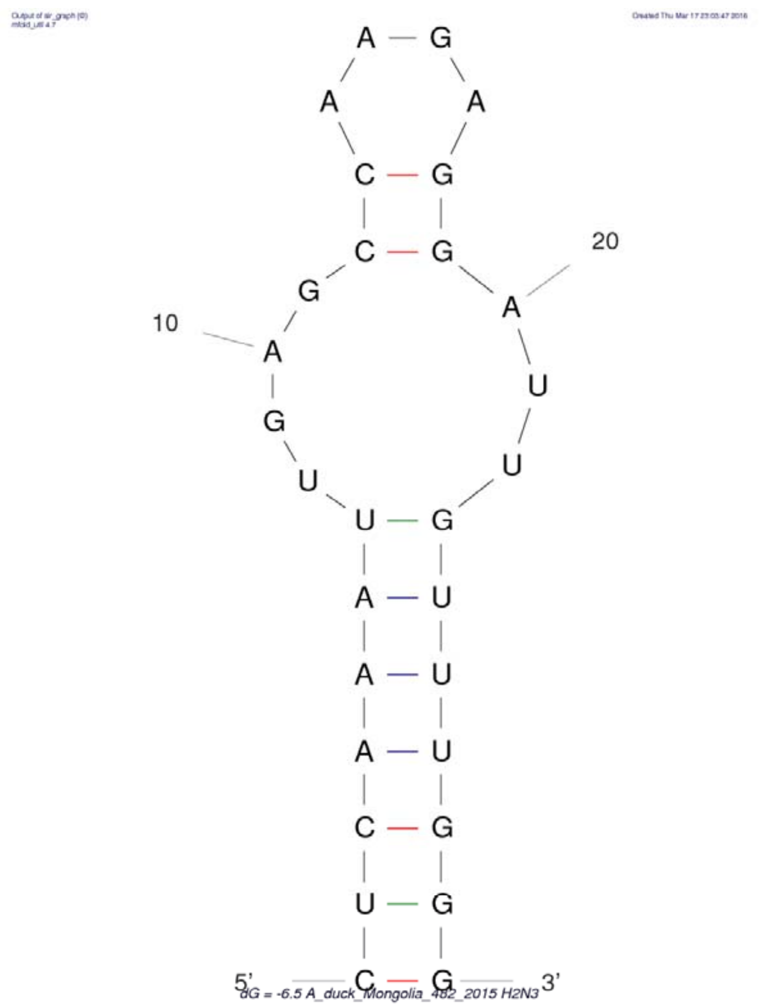
Output of sir_graph (©)
mfold_util 4.7
Created Thu Mar 17 23:03:47 2016
10
20
5'
3'
dG = -6.5 A_duck_Mongolia_482_2015 H2N3

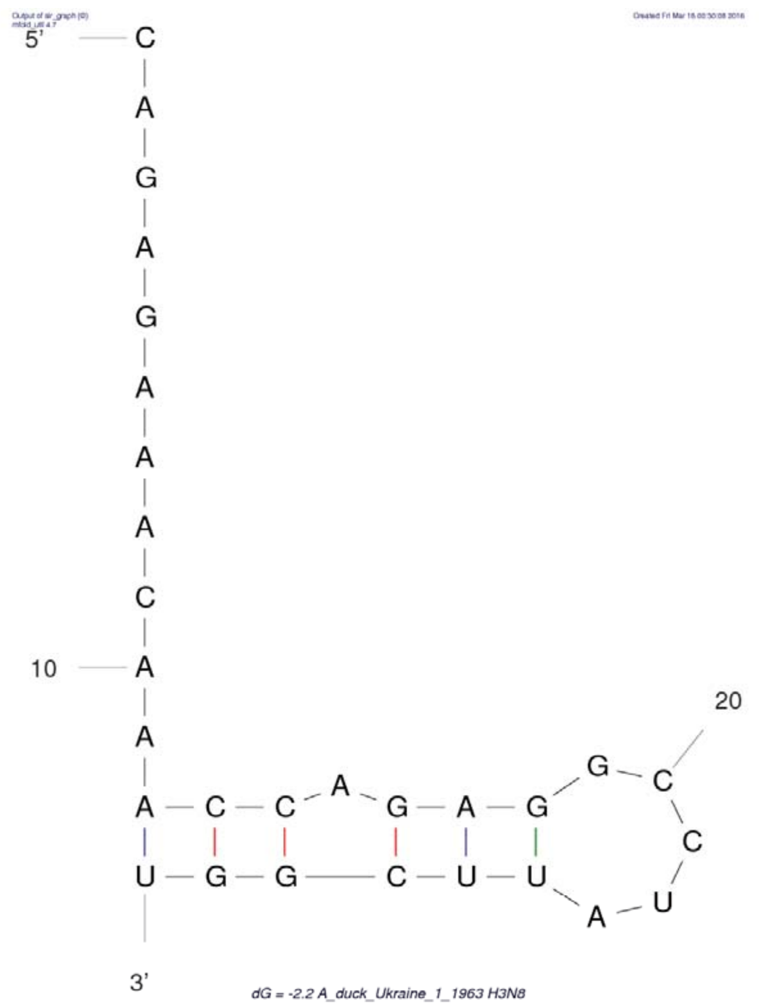
Output of sir_graph (©)
mfold_util 4.7
Created Fri Mar 18 00:30:08 2016
5'
10
20
3'
dG = -2.2 A_duck_Ukraine_1_1963 H3N8

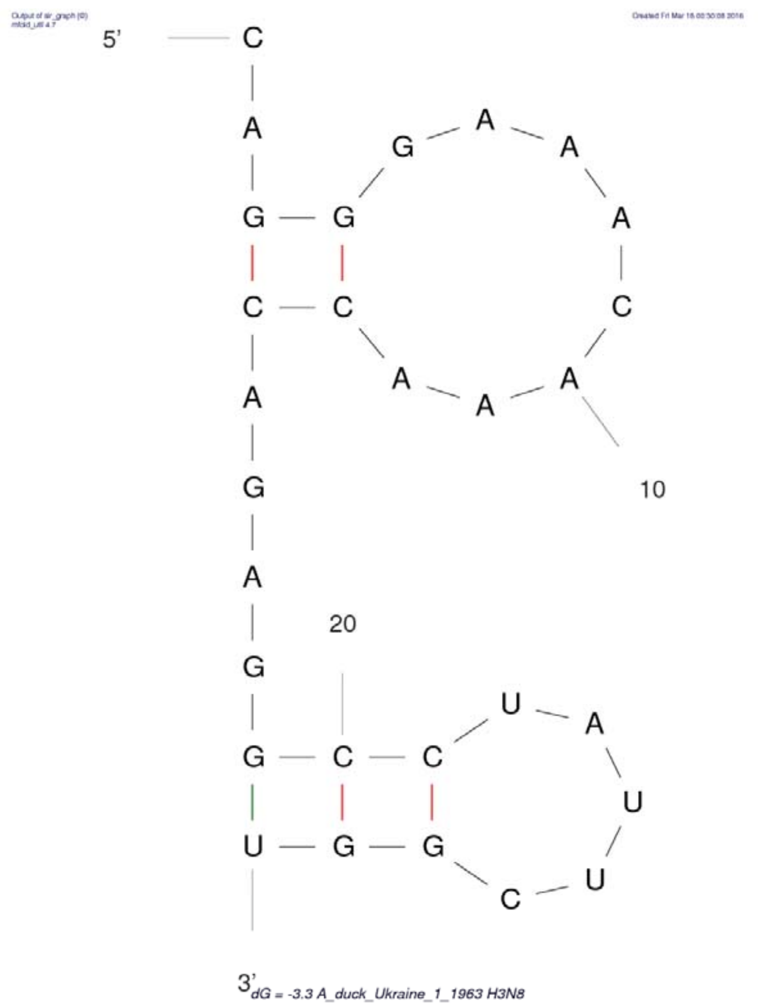
Output of sir_graph (©)
mfold_util 4.7
Created Fri Mar 18 00:30:08 2016
5'
10
20
3'
dG = -3.3 A_duck_Ukraine_1_1963 H3N8

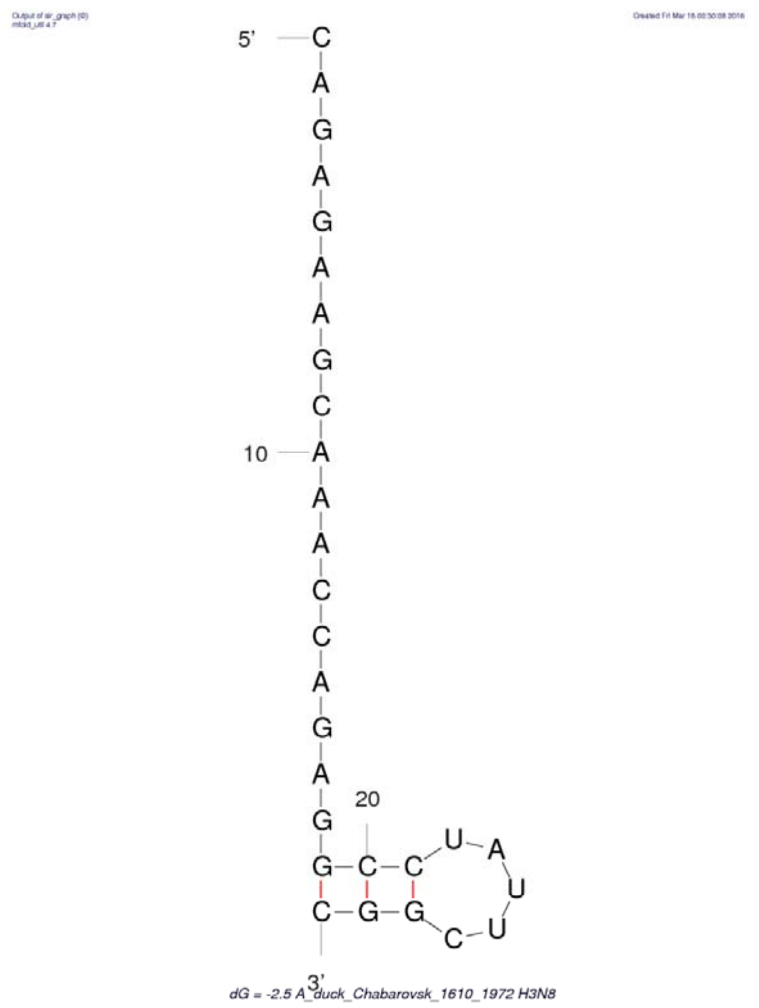
Output of sir_graph (©)
mfold_util 4.7
Created Fri Mar 18 00:30:08 2016
5'
10
20
3'
dG = -2.5 A_duck_Chabarovsk_1610_1972 H3N8

Output of sir_graph (©)
mfold_util 4.7

Created Fri Mar 18 00:30:08 2016

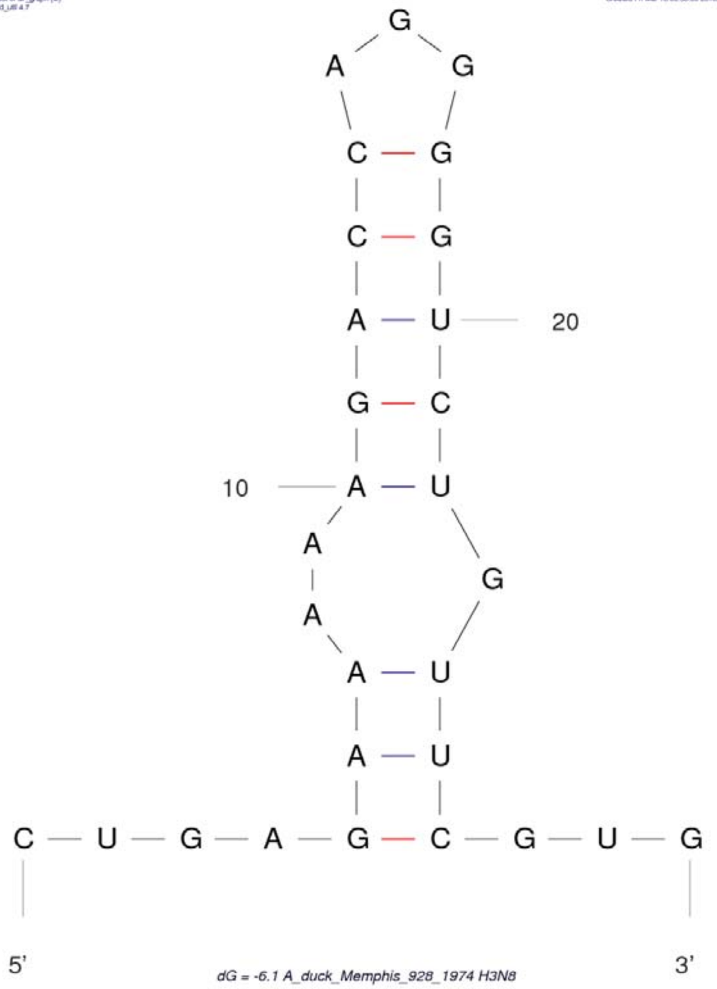

dG = -6.1 A_duck_Memphis_928_1974 H3N8

Output of sir_graph (©)
mfold_util 4.7

Created Fri Mar 18 00:30:09 2016

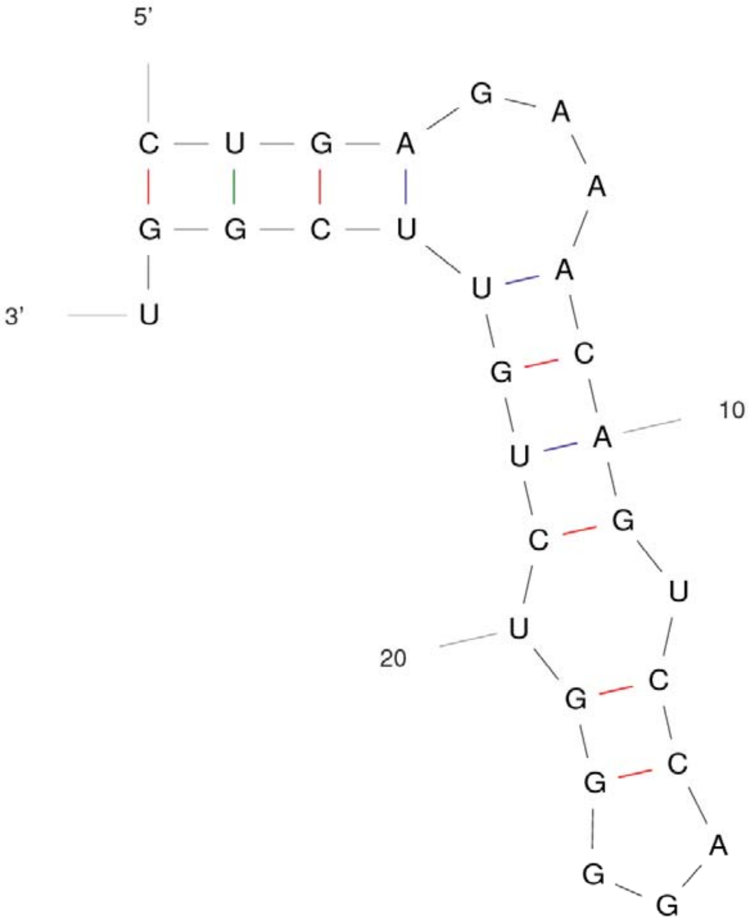

dG = -7 A_duck_Minnesota_53_1974 H3N8

Output of sir_graph (©)
mfold_util 4.7

Created Fri Mar 18 00:30:09 2016

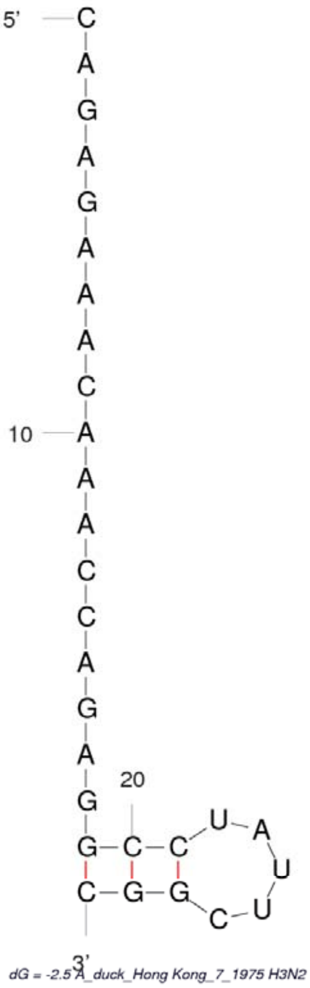

dG = -2.5 A_duck_Hong Kong_7_1975 H3N2

Output of sir_graph (©)
mfold_util 4.7

Created Fri Mar 18 00:30:09 2016

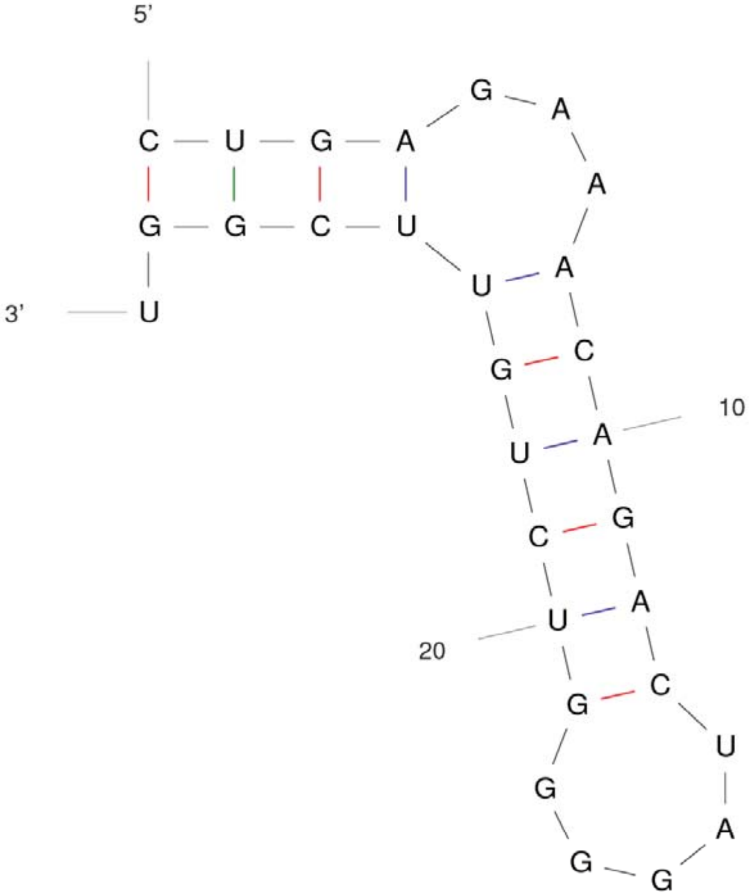

dG = -9.5 A_duck_Alberta_78_1976 H3N2

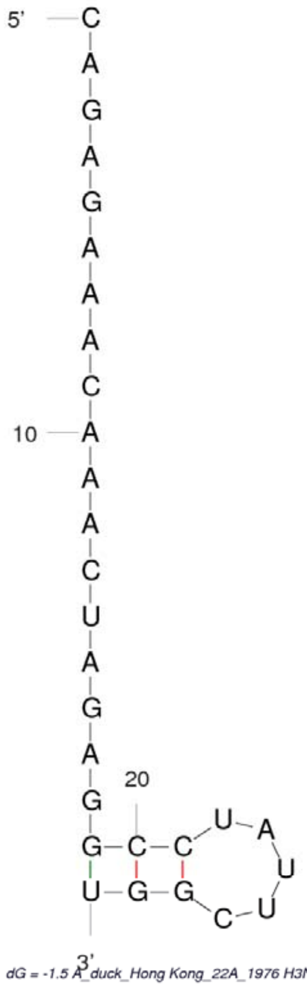

dG = -1.5 A_duck_Hong Kong_22A_1976 H3N3

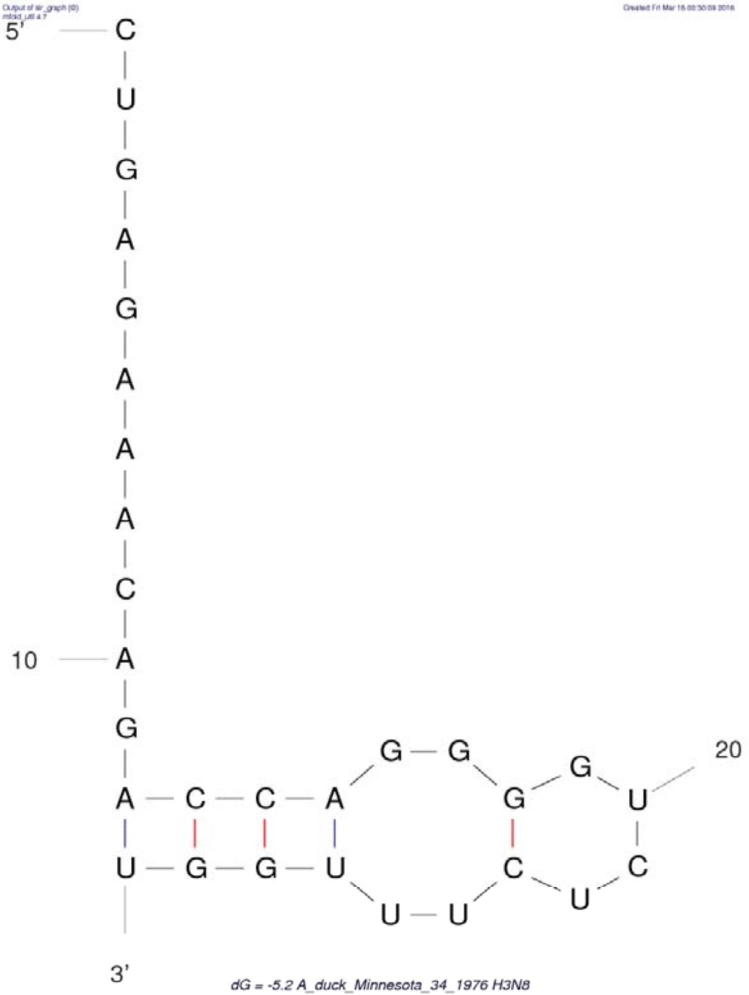

dG = -5.2 A_duck_Minnesota_34_1976 H3N8

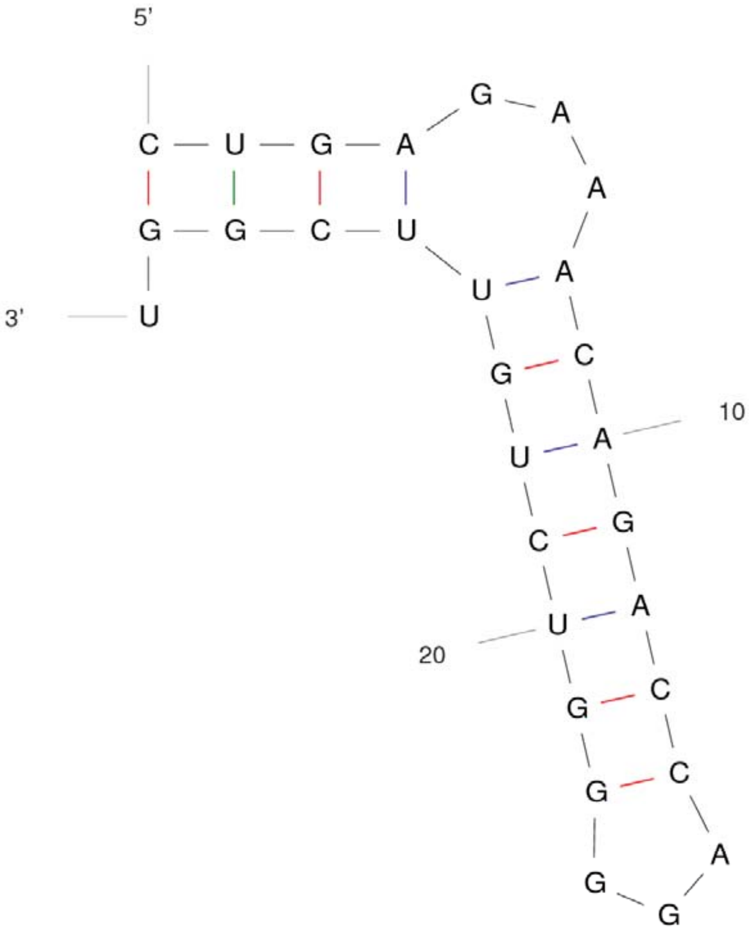

dG = -10.9 A_mallard duck_ALB_31_1976 H3N8

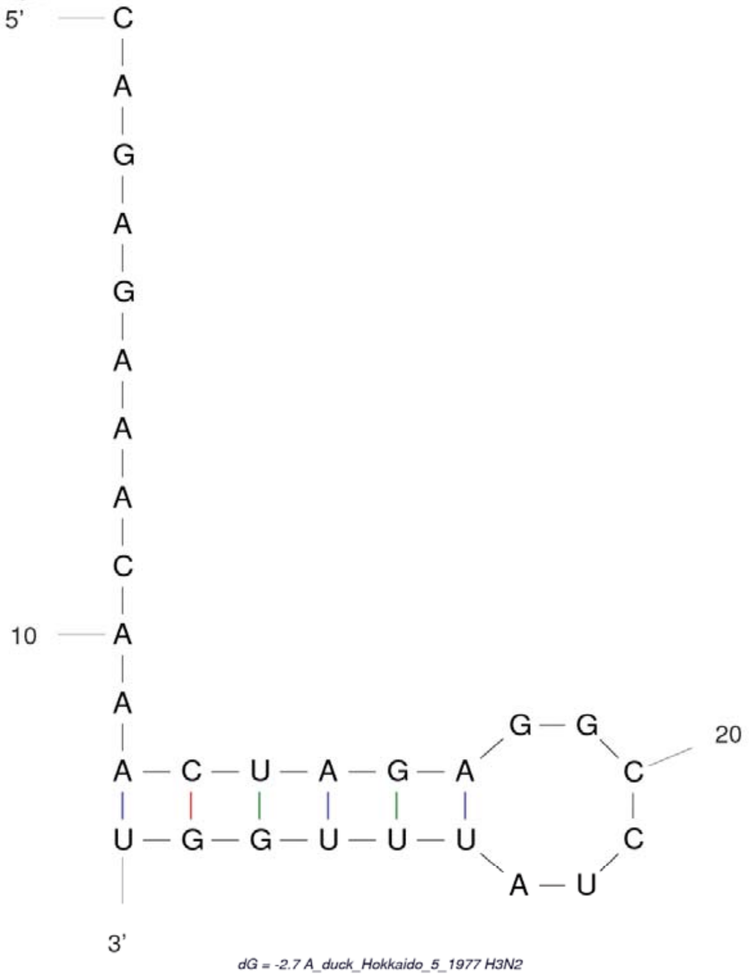

dG = -2.7 A_duck_Hokkaido_5_1977 H3N2

Output of sir_graph (©)
mfold_util 4.7

Created Fri Mar 18 00:30:10 2016

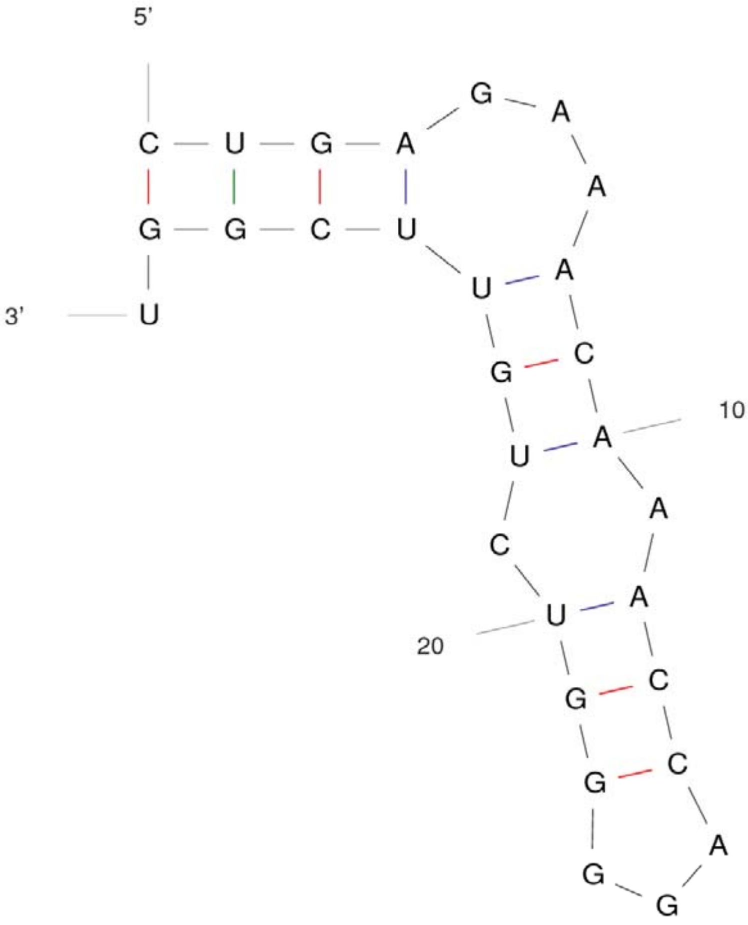

dG = -4.7 A_duck_NY_6874_1978 H3N2

Output of sir_graph (©)
mfold_util 4.7

Created Fri Mar 18 00:30:10 2016

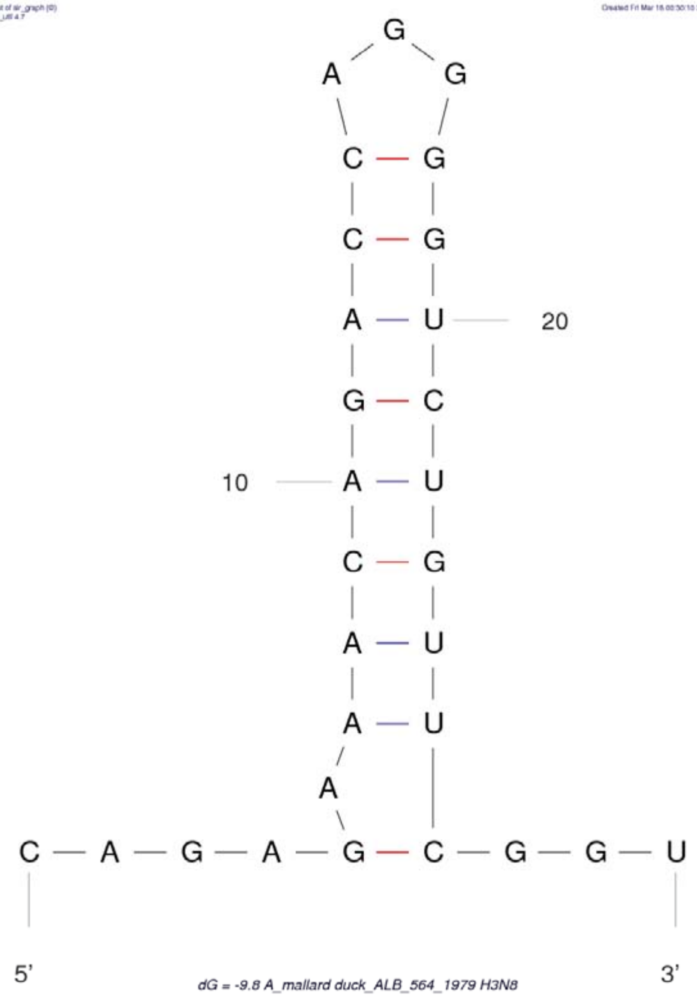

dG = -9.8 A_mallard duck_ALB_564_1979 H3N8

Output of sir_graph (©)
mfold_util 4.7

Created Fri Mar 18 00:30:11 2016

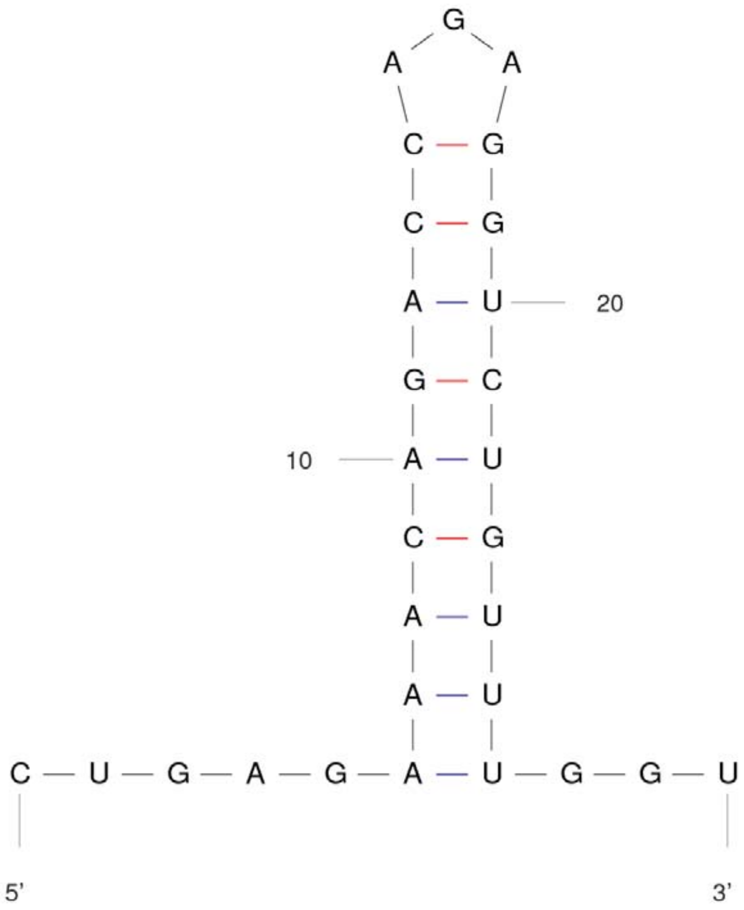

dG = -11.1 A_pintail duck_ALB_462_1979 H3N6

Output of sir_graph (©)
mfold_util 4.7

Created Fri Mar 18 00:30:11 2016

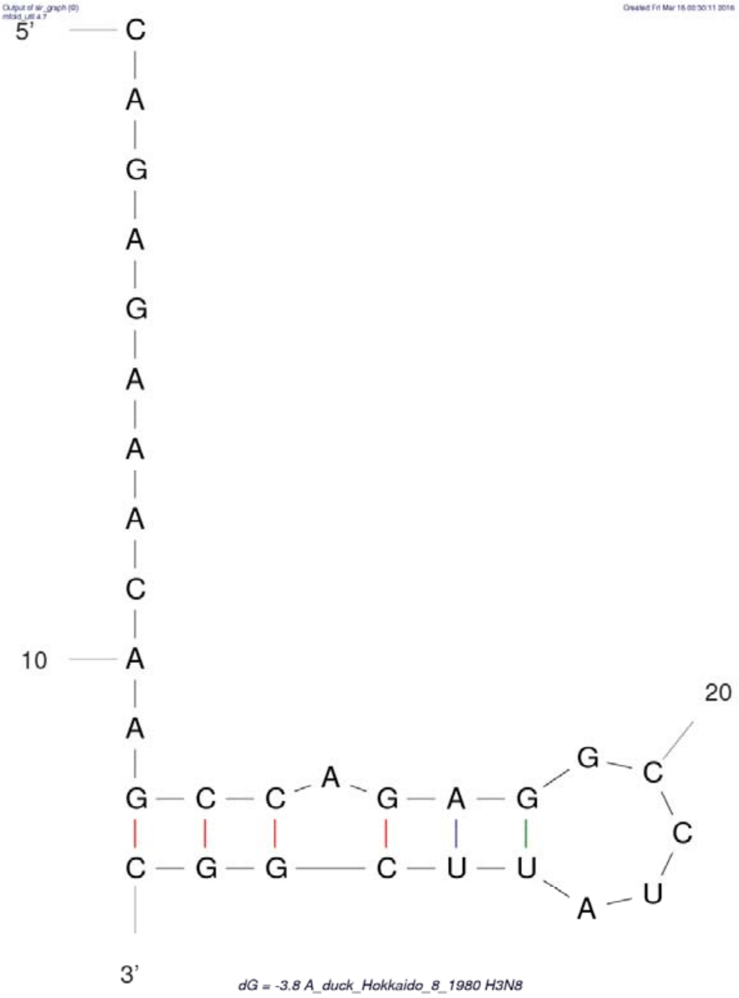

dG = -3.8 A_duck_Hokkaido_8_1980 H3N8

Output of sir_graph (©)
mfold_util 4.7

Created Fri Mar 18 00:30:11 2016

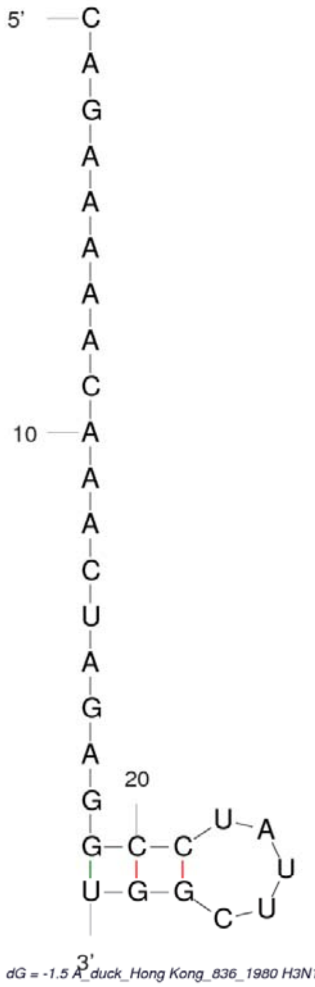

dG = -1.5 A_duck_Hong Kong_836_1980 H3N1

Output of sir_graph (©)
mfold_util 4.7

Created Fri Mar 18 00:30:11 2016

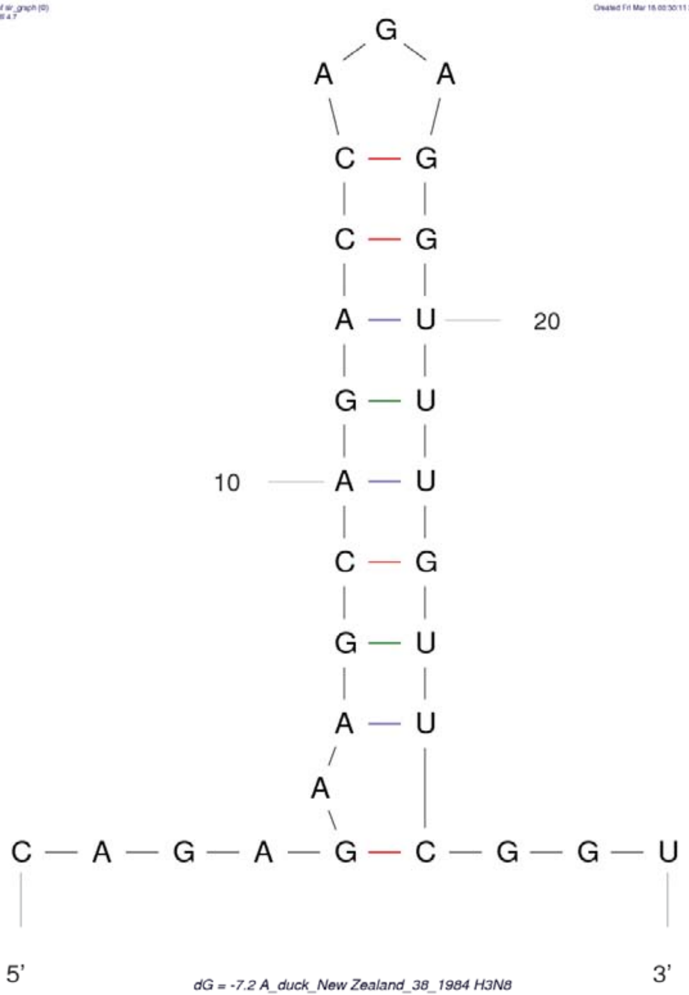

dG = -7.2 A_duck_New Zealand_38_1984 H3N8

Output of sir_graph (©)
mfold_util 4.7

Created Fri Mar 18 00:30:11 2016

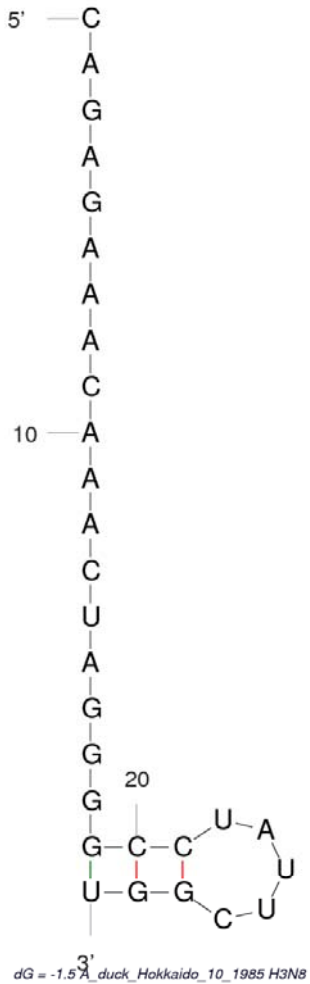

dG = -1.5 A_duck_Hokkaido_10_1985 H3N8

Output of sir_graph (©)
mfold_util 4.7

Created Fri Mar 18 00:30:11 2016

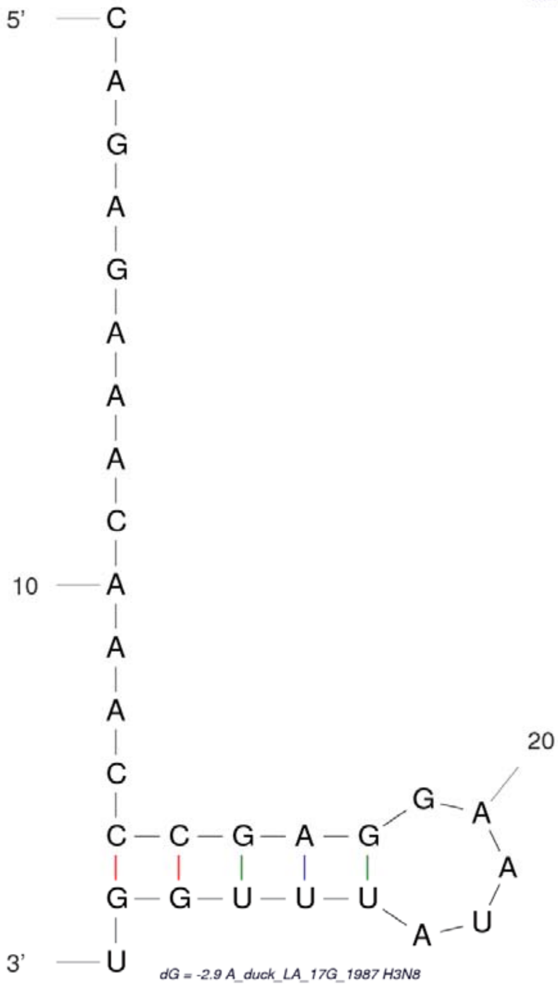

dG = -2.9 A_duck_LA_17G_1987 H3N8

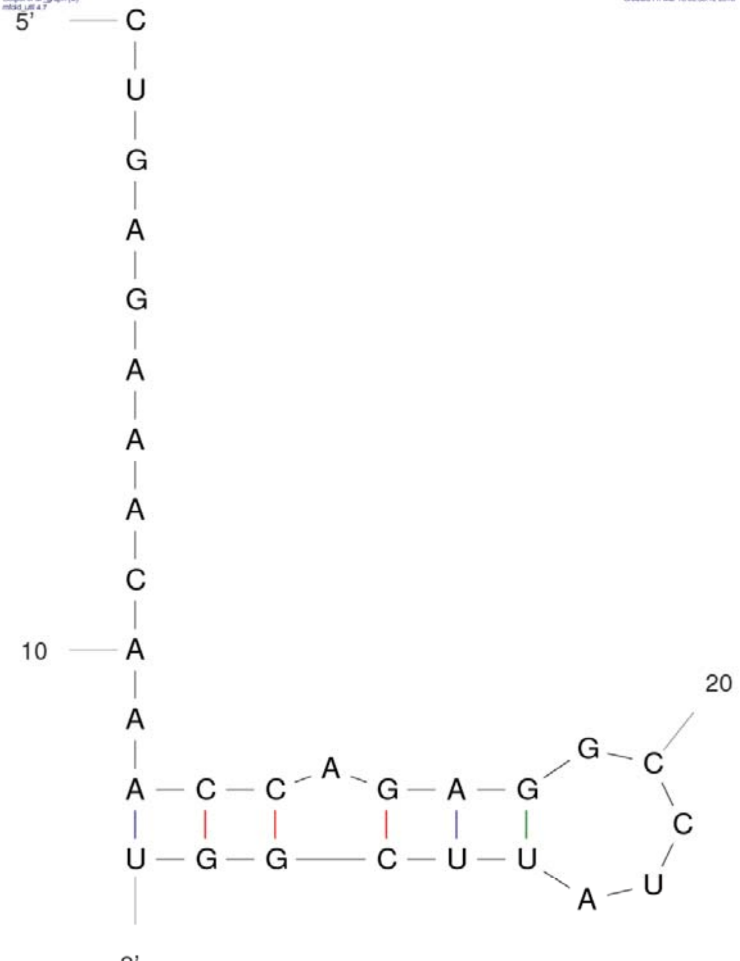

dG = -2.2 A_duck_Nanchang_1681_1992 H3N8

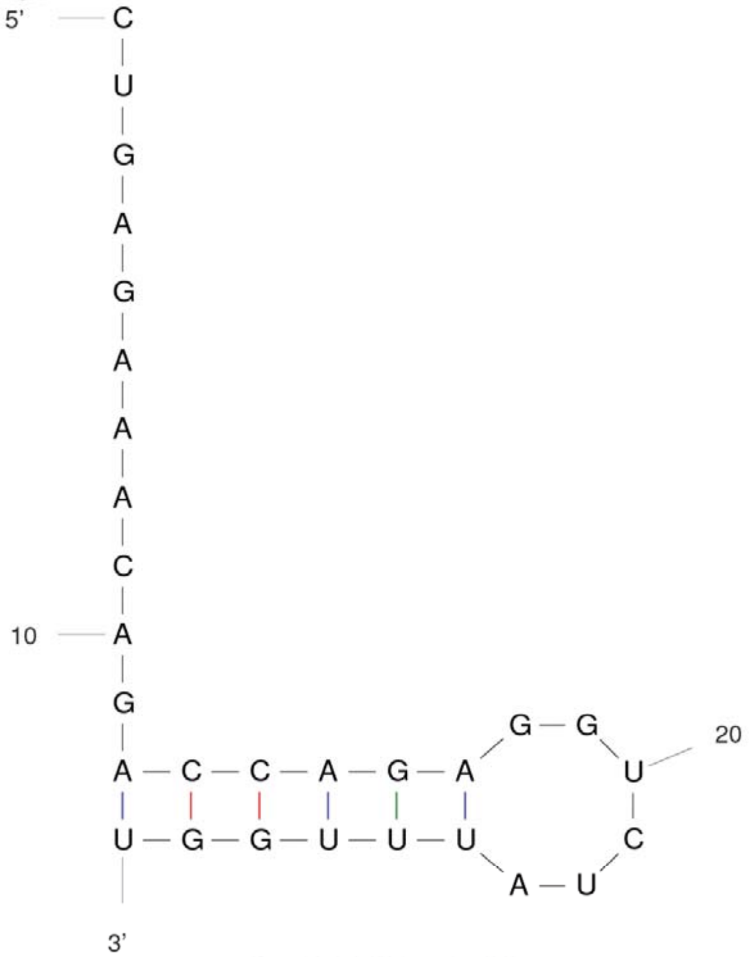

dG = -5.1 A_duck_NY_13822_1995 H3N8

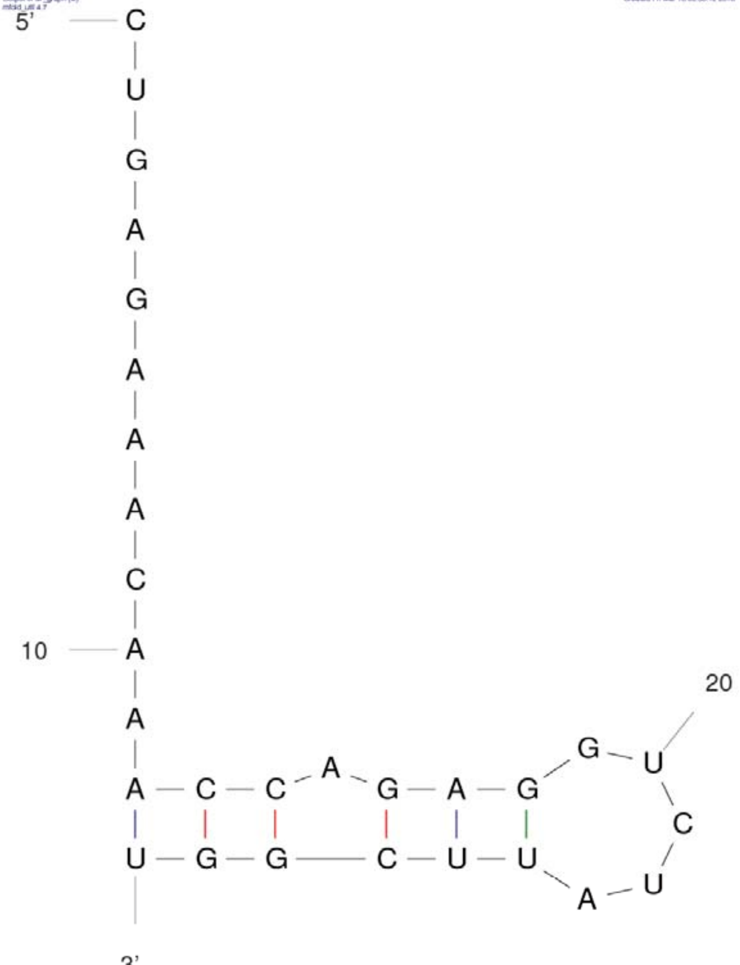

dG = -2.2 A_duck_Guangdong_455_2000 H3N9

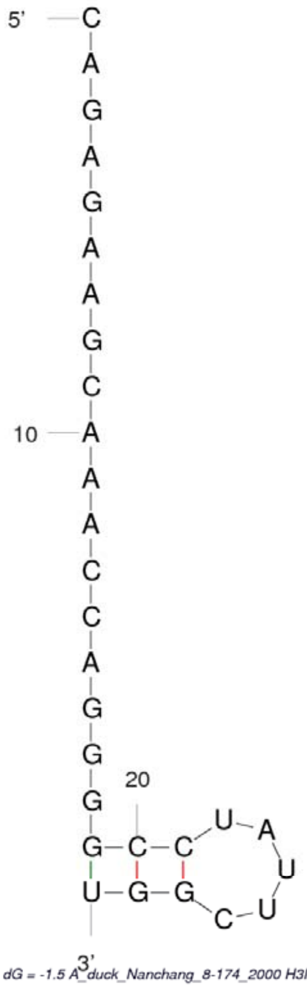

dG = -1.5 A_duck_Nanchang_8-174_2000 H3N6

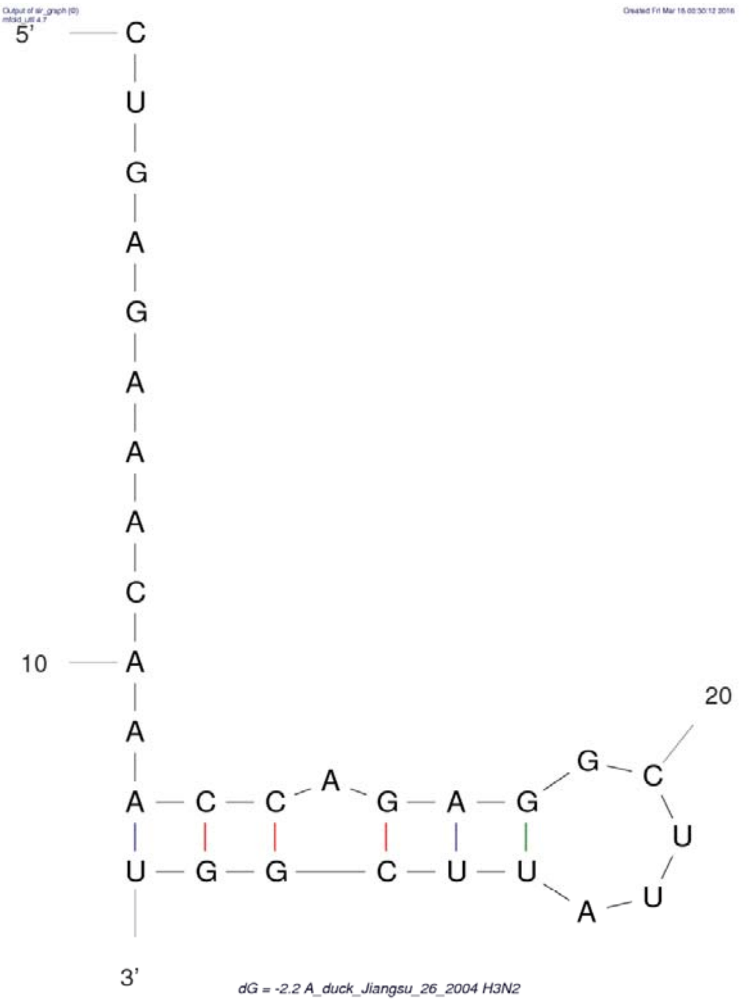

dG = -2.2 A_duck_Jiangsu_26_2004 H3N2

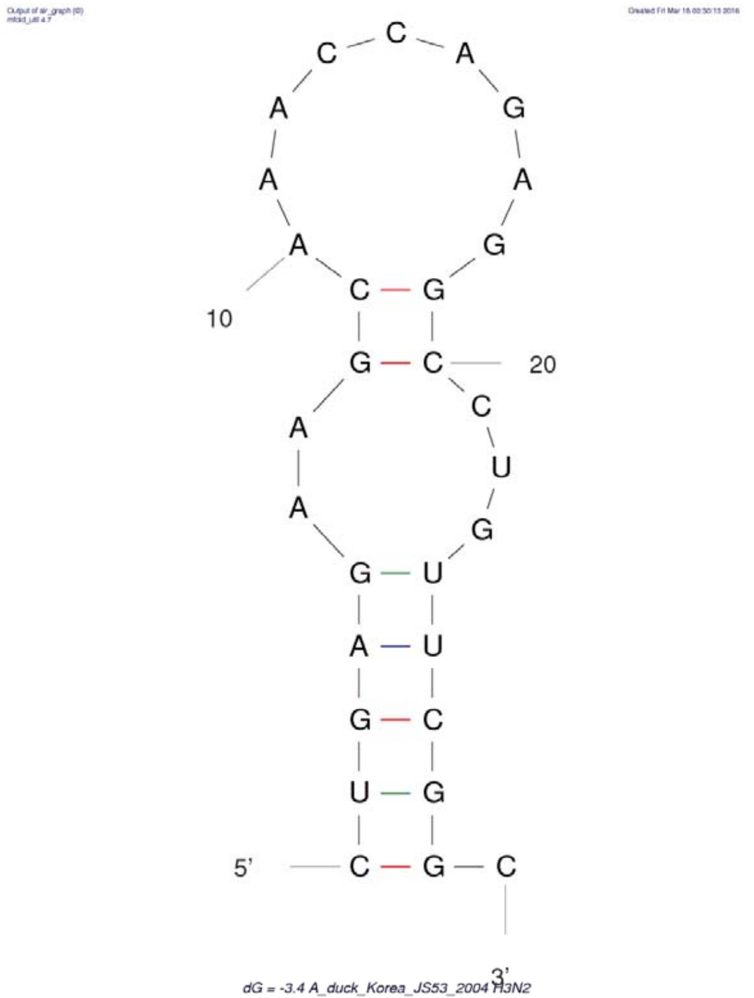

dG = -3.4 A_duck_Korea_JS53_2004 H3N2

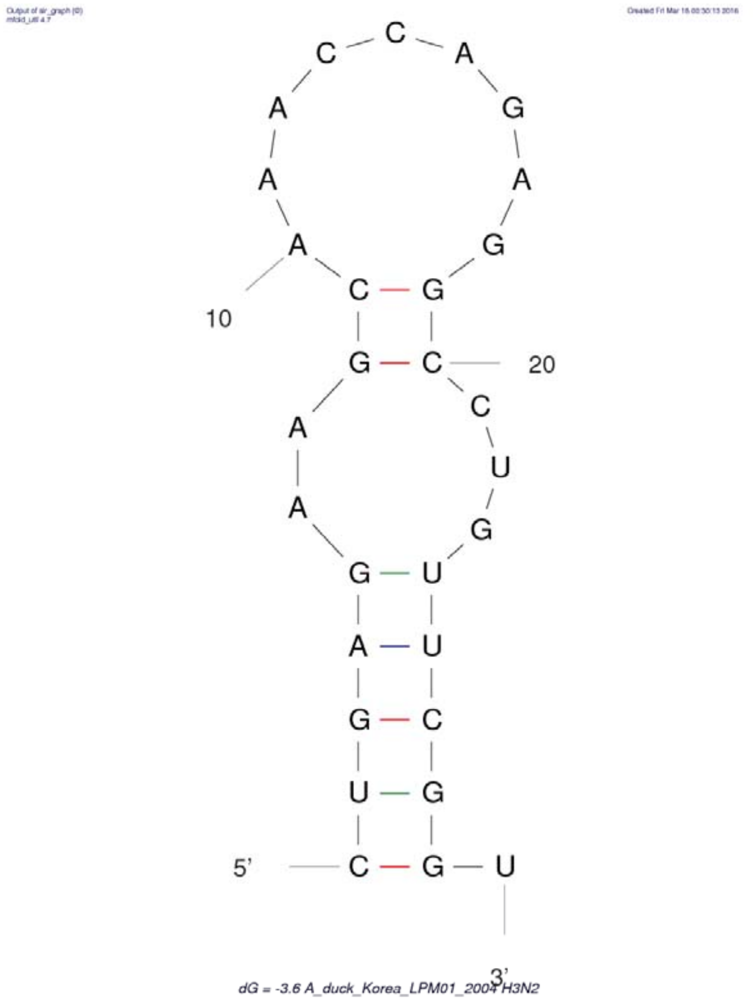

dG = -3.6 A_duck_Korea_LPM01_2004 H3N2

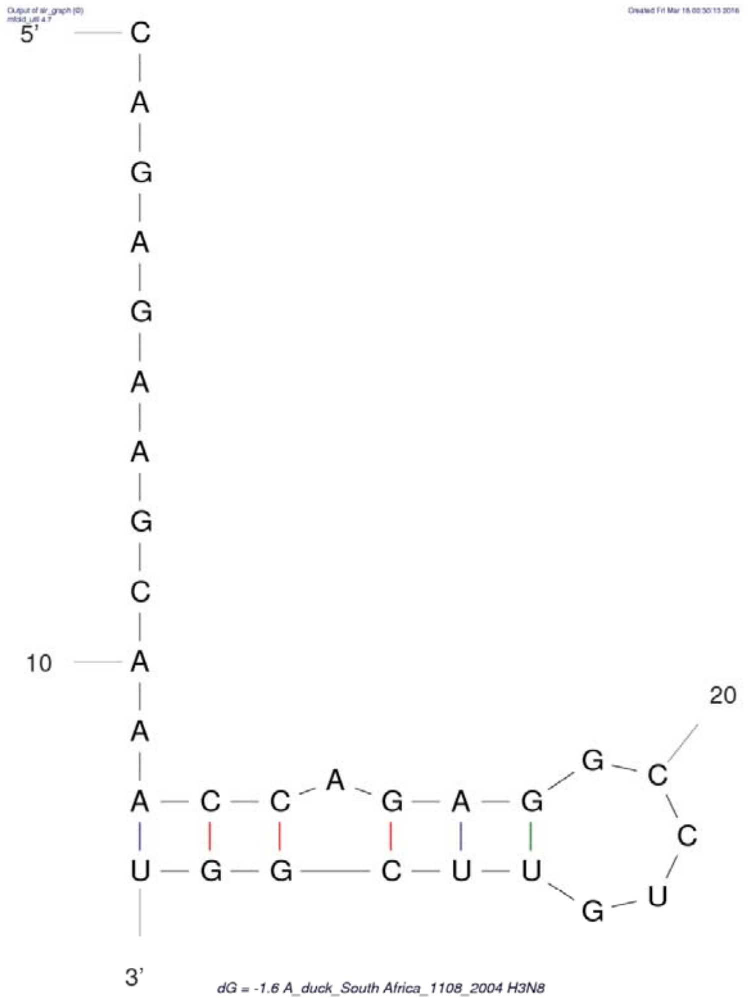

dG = -1.6 A_duck_South Africa_1108_2004 H3N8

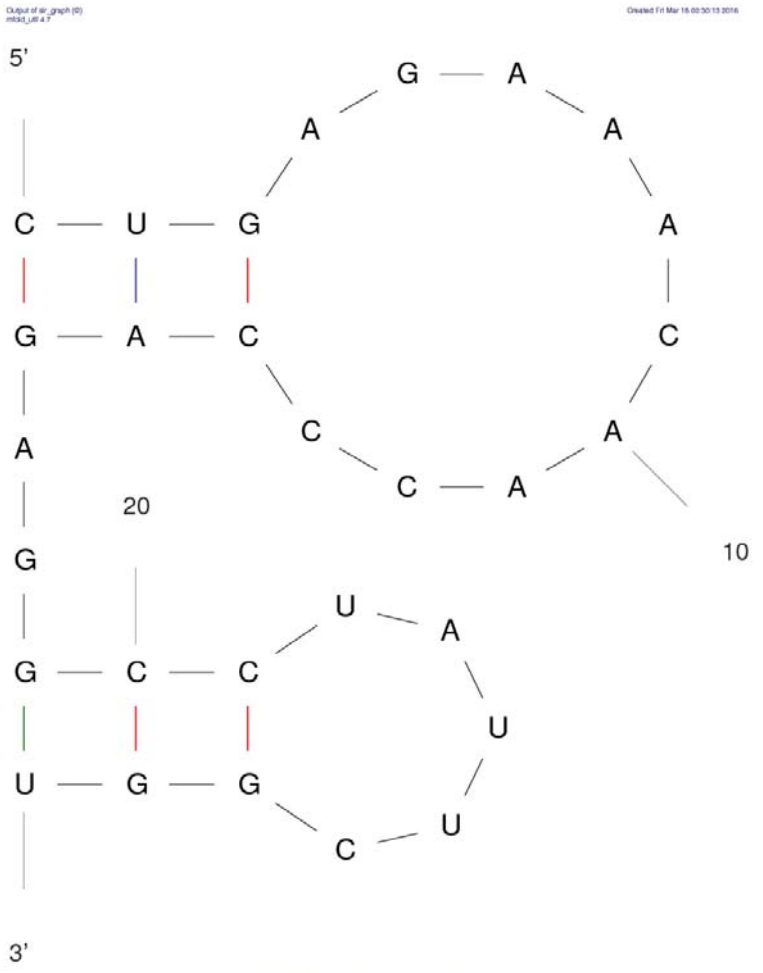

dG = -1.8 A_duck_Beijing_61_05 H3N8

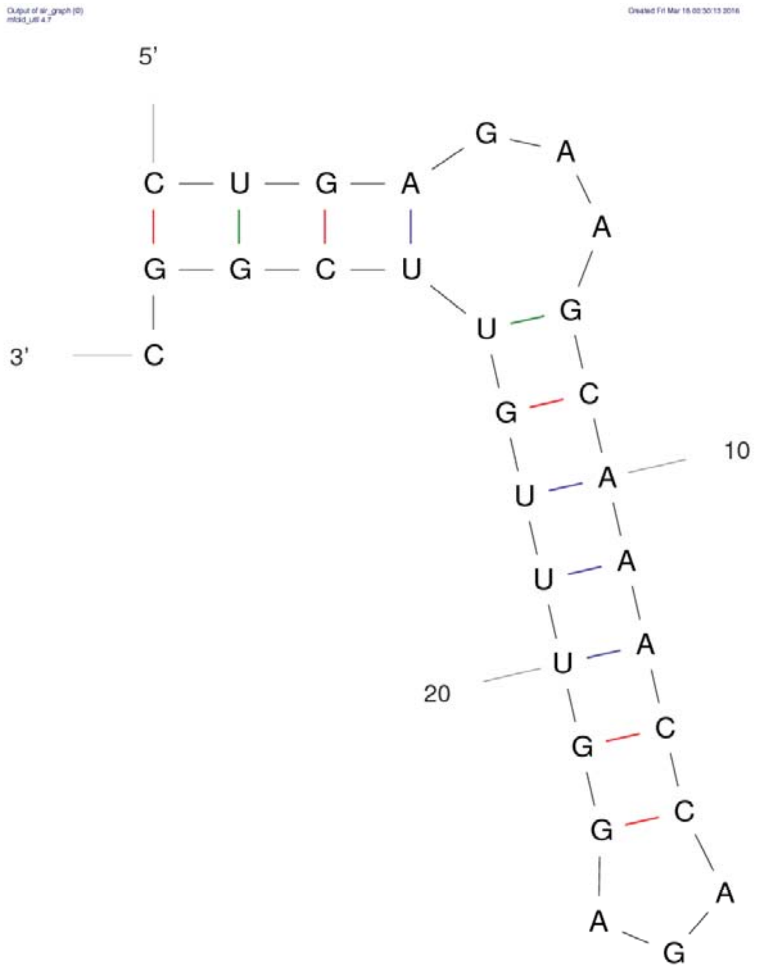

dG = -8.3 A_duck_Korea_LPM36_2005 H3N6

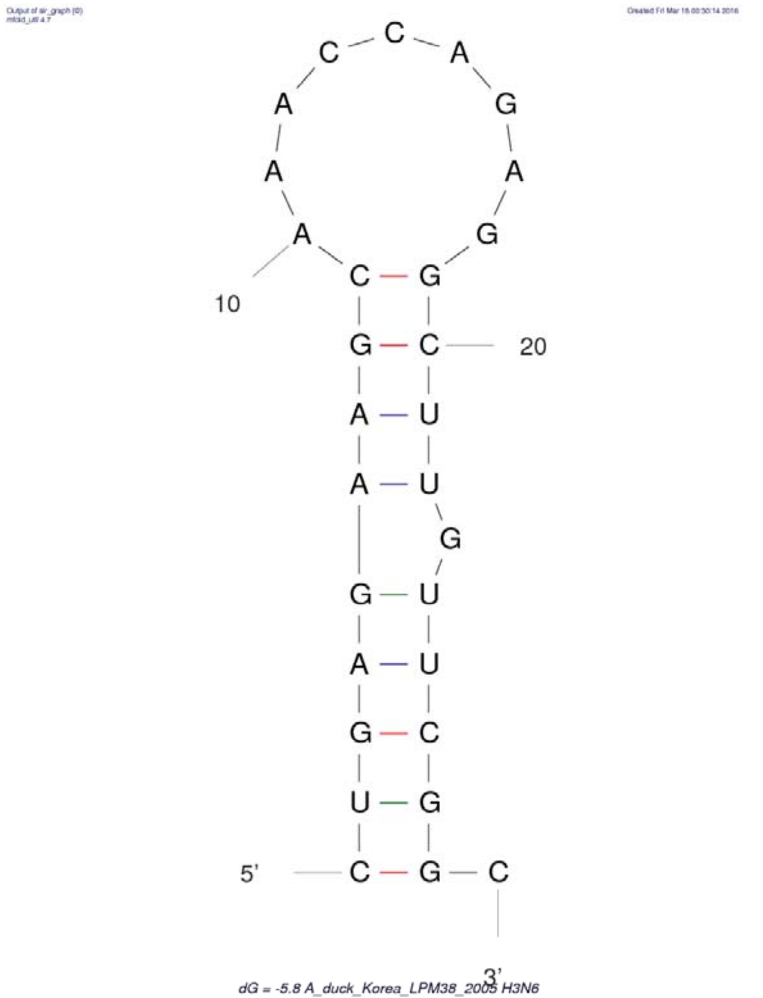

dG = -5.8 A_duck_Korea_LPM38_2005 H3N6

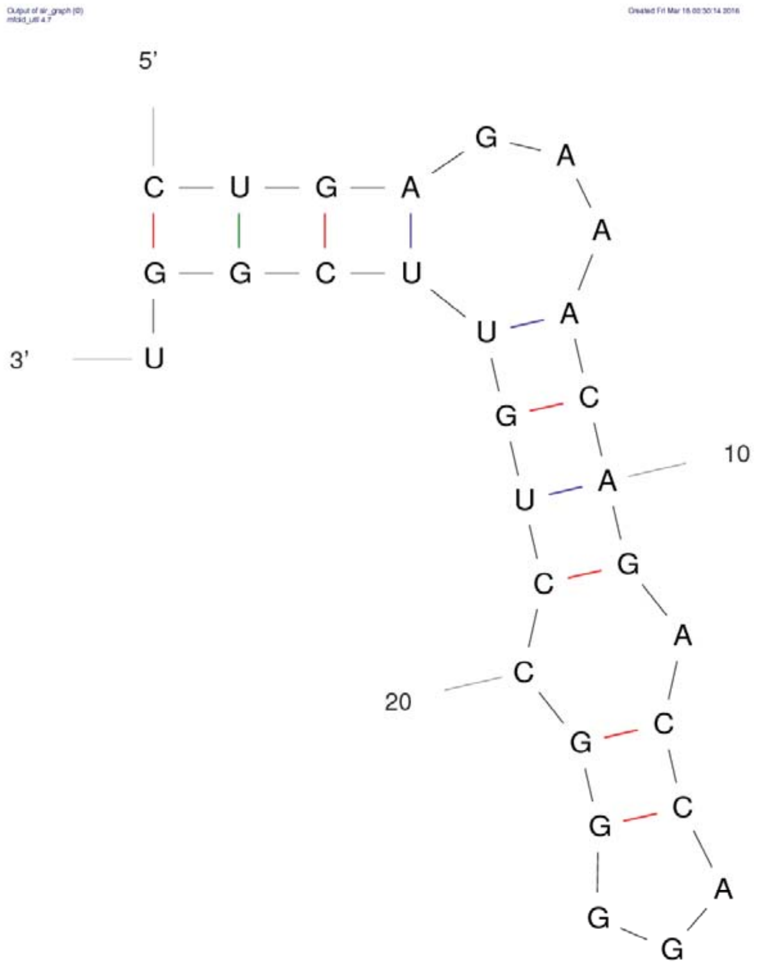

dG = -5.9 A_longtail duck_Maryland_291_2005 H3N8

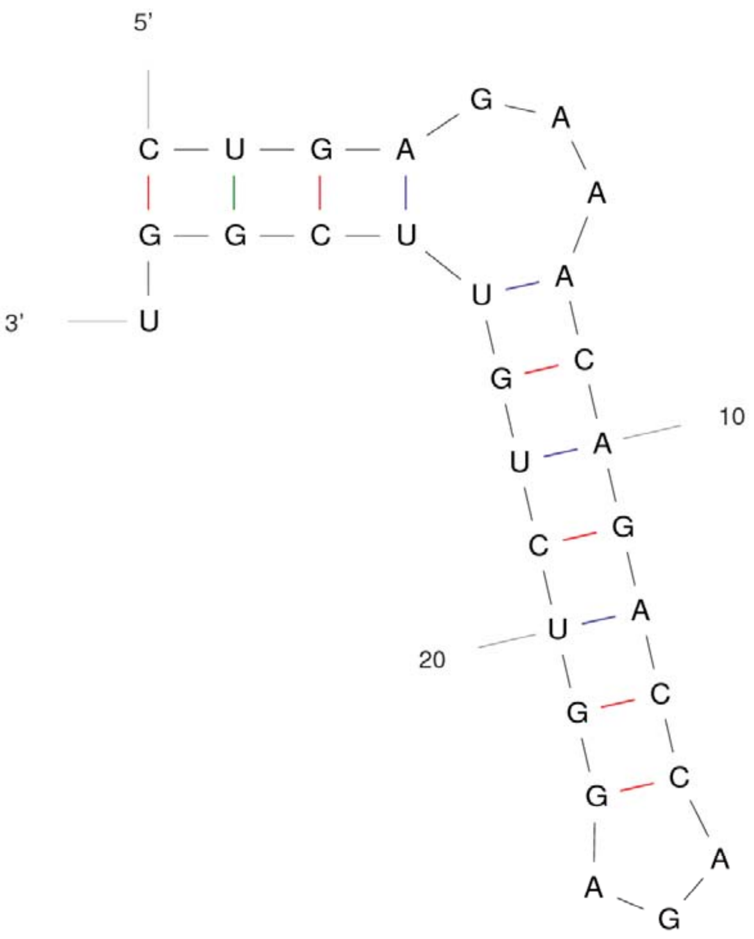

dG = -10.9 A_American black duck_Quebec_11235_2006 H3N2

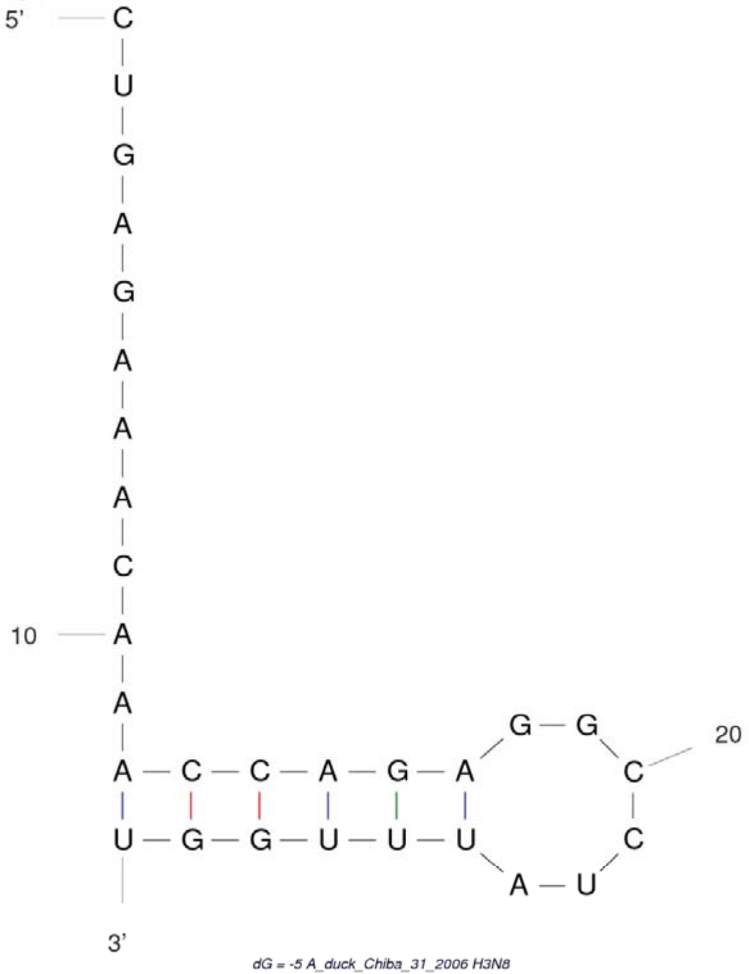

dG = -5 A_duck_Chiba_31_2006 H3N8

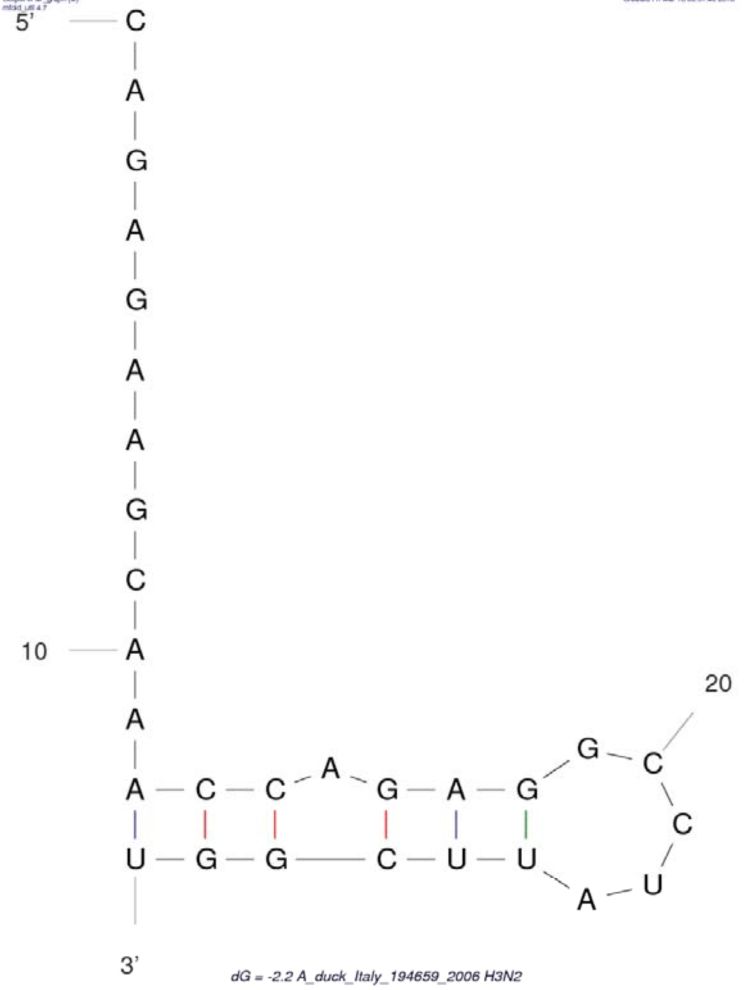

dG = -2.2 A_duck_Italy_194659_2006 H3N2

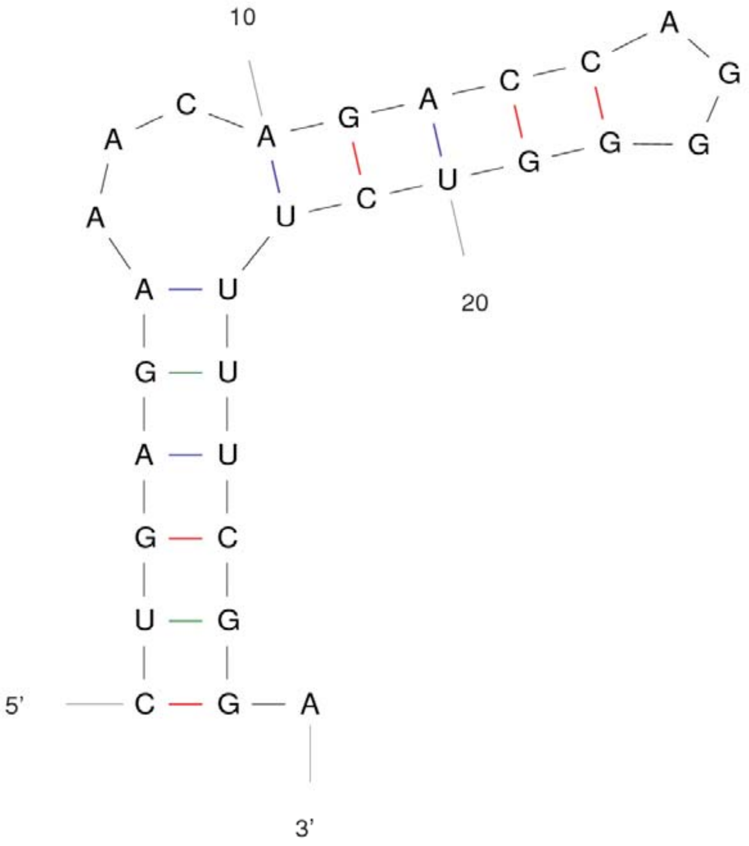

dG = -9 A_American black duck_New Brunswick_02656_2007 H3N8

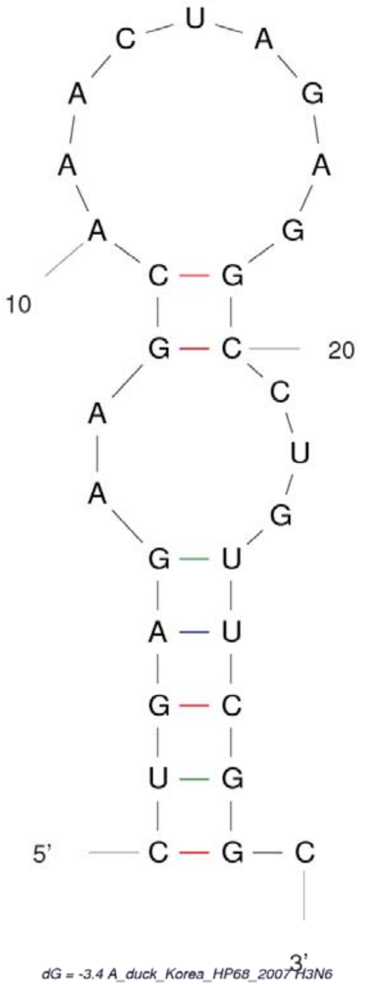

dG = -3.4 A_duck_Korea_HP68_2007 H3N6

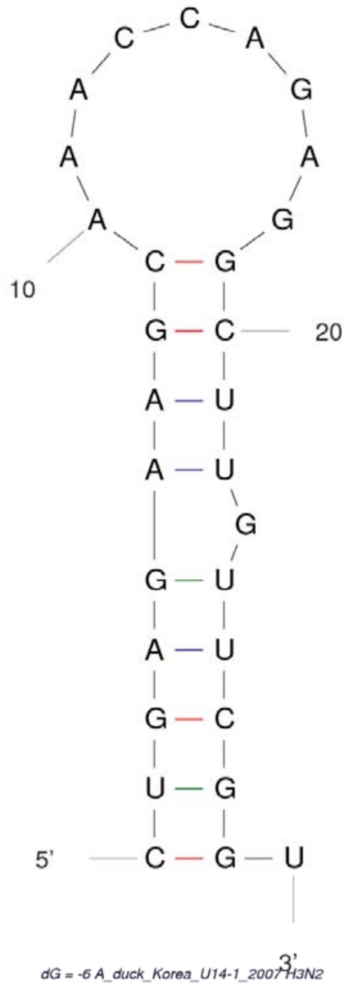

dG = -6 A_duck_Korea_U14-1_2007 H3N2

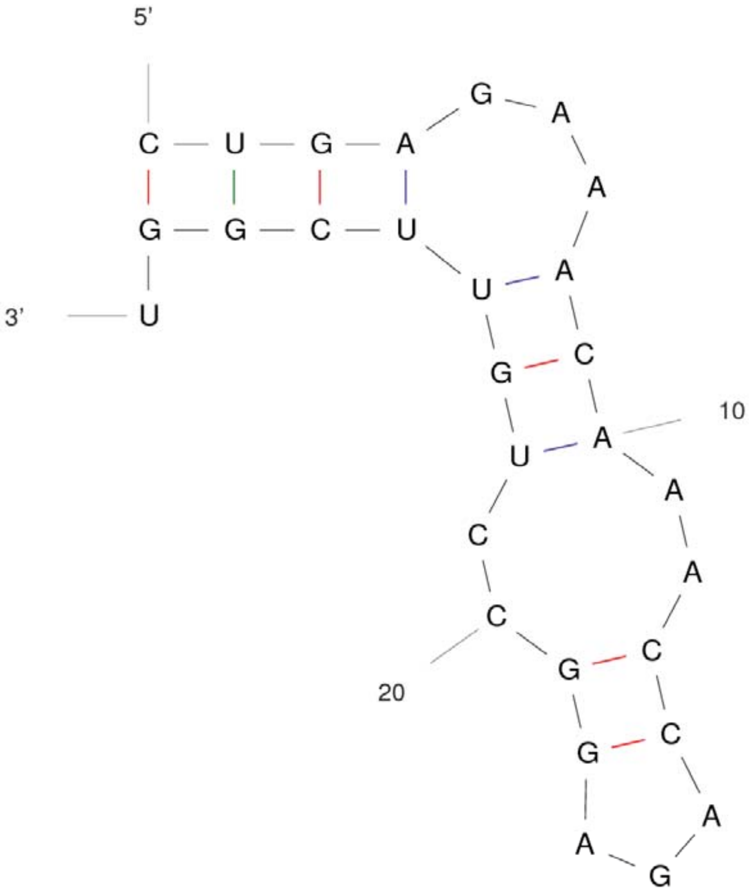

dG = -2.5 A_duck_Korea_U2-5_2007 H3N8

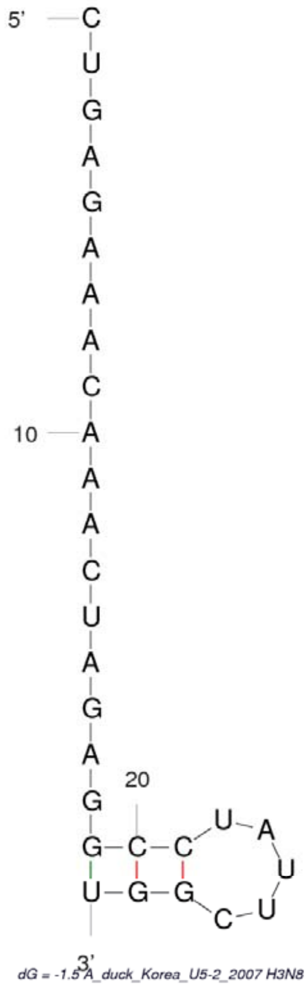

dG = -1.5 A_duck_Korea_U5-2_2007 H3N8

Output of sir_graph (©)
mfold_util 4.7

Created Fri Mar 18 00:31:48 2016

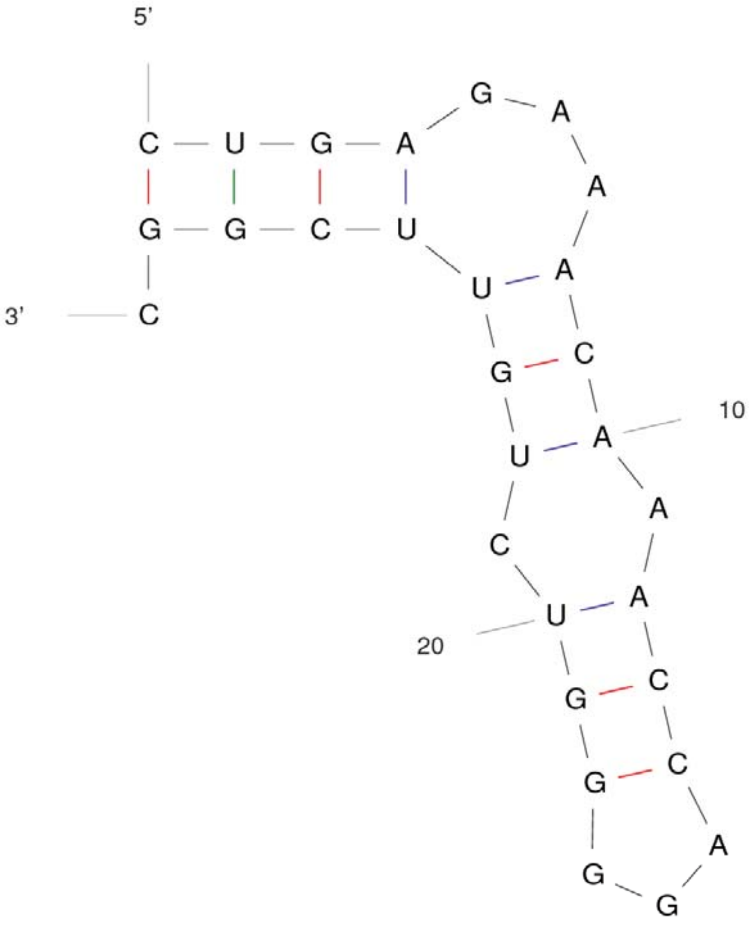

dG = -4.5 A_ring-necked duck_Minnesota_Sg-00066_2007 MixedH3

Output of sir_graph (©)
mfold_util 4.7

Created Fri Mar 18 00:31:48 2016

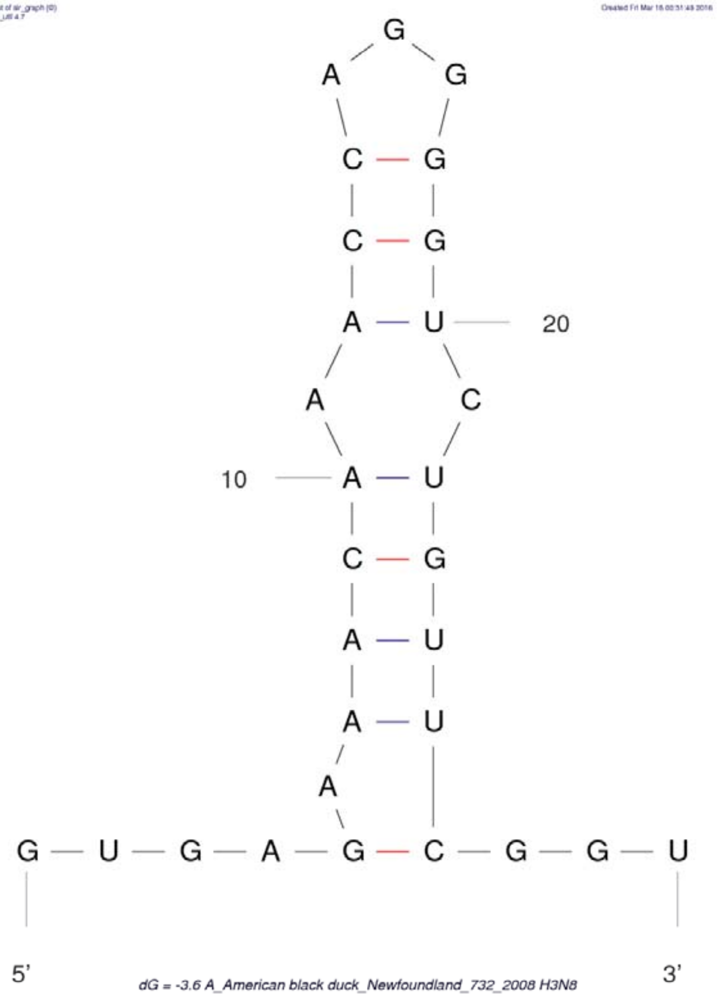

dG = -3.6 A_American black duck_Newfoundland_732_2008 H3N8

Output of sir_graph (©)
mfold_util 4.7

Created Fri Mar 18 00:31:50 2016

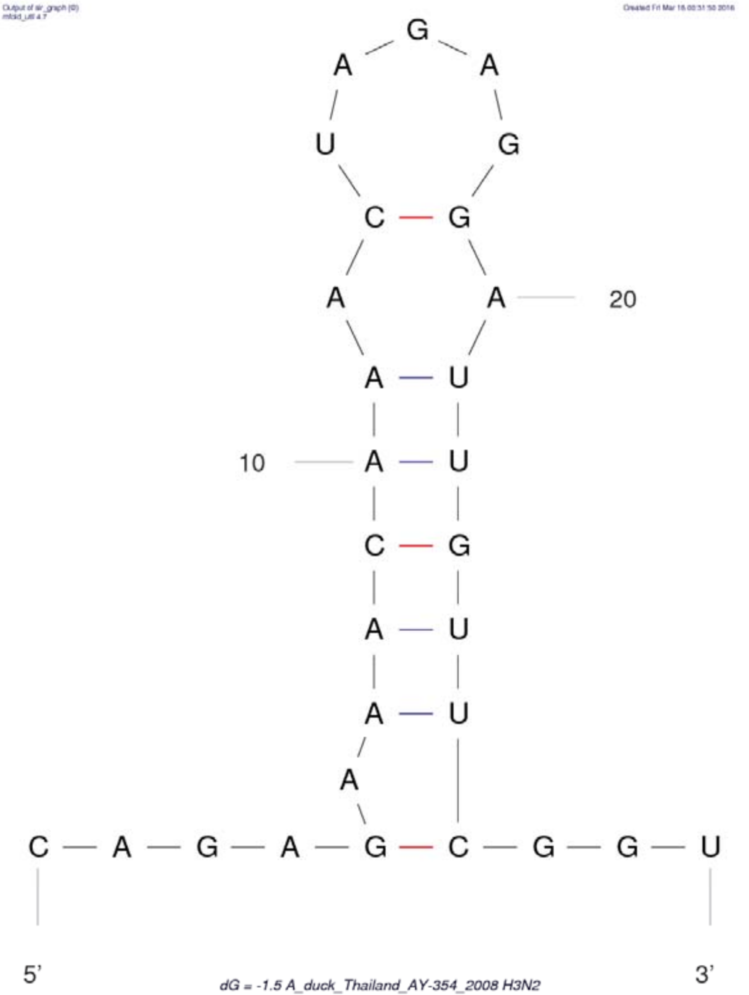

dG = -1.5 A_duck_Thailand_AY-354_2008 H3N2

Output of sir_graph (©)
mfold_util 4.7

Created Fri Mar 18 00:31:50 2016

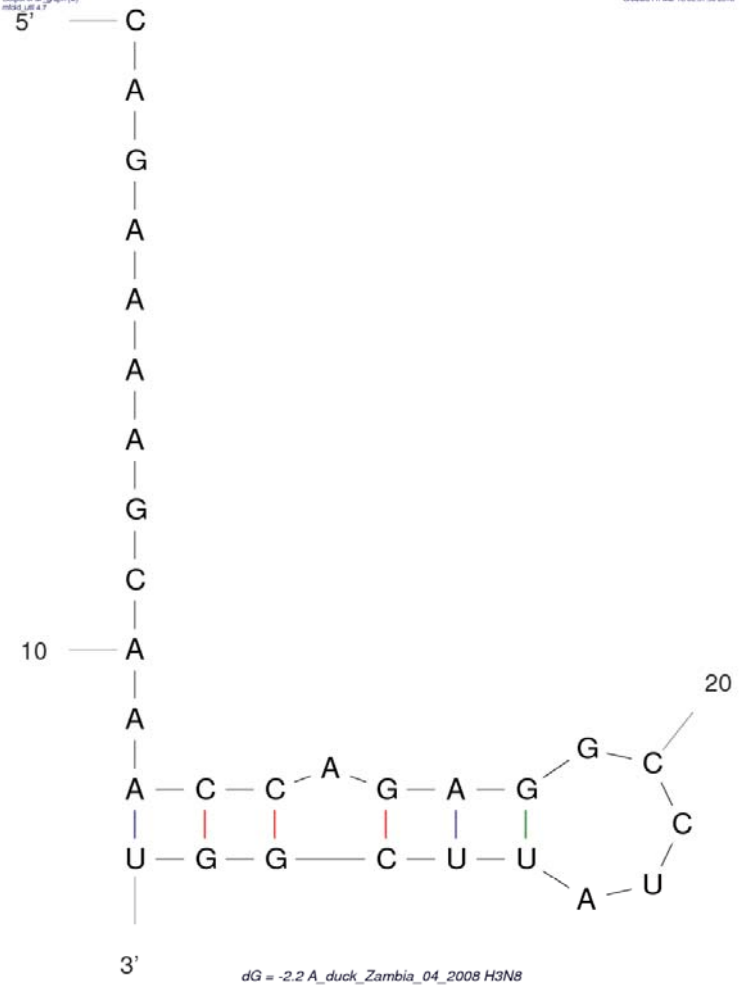

dG = -2.2 A_duck_Zambia_04_2008 H3N8

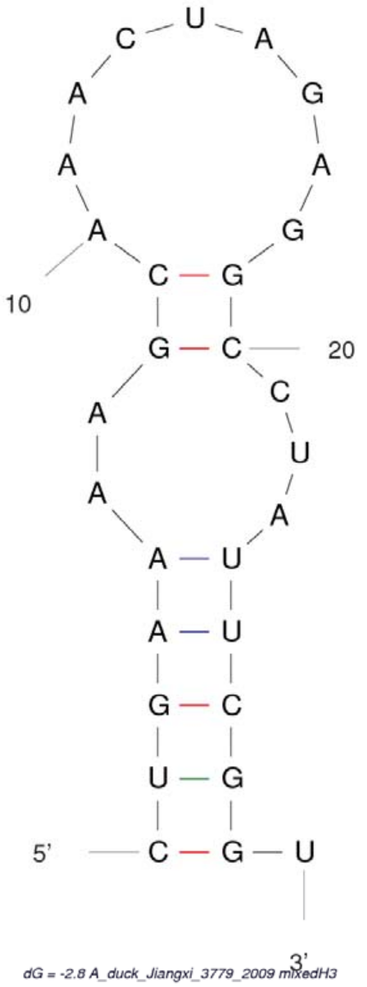

dG = -2.8 A_duck_Jiangxi_3779_2009 mixedH3

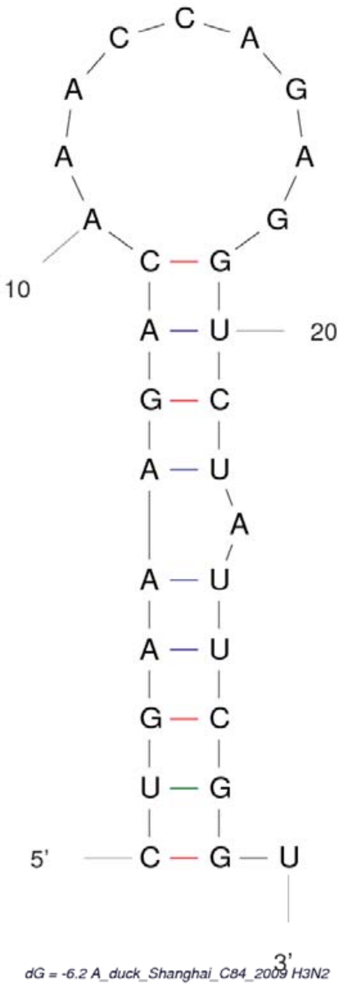

dG = -6.2 A_duck_Shanghai_C84_2009 H3N2

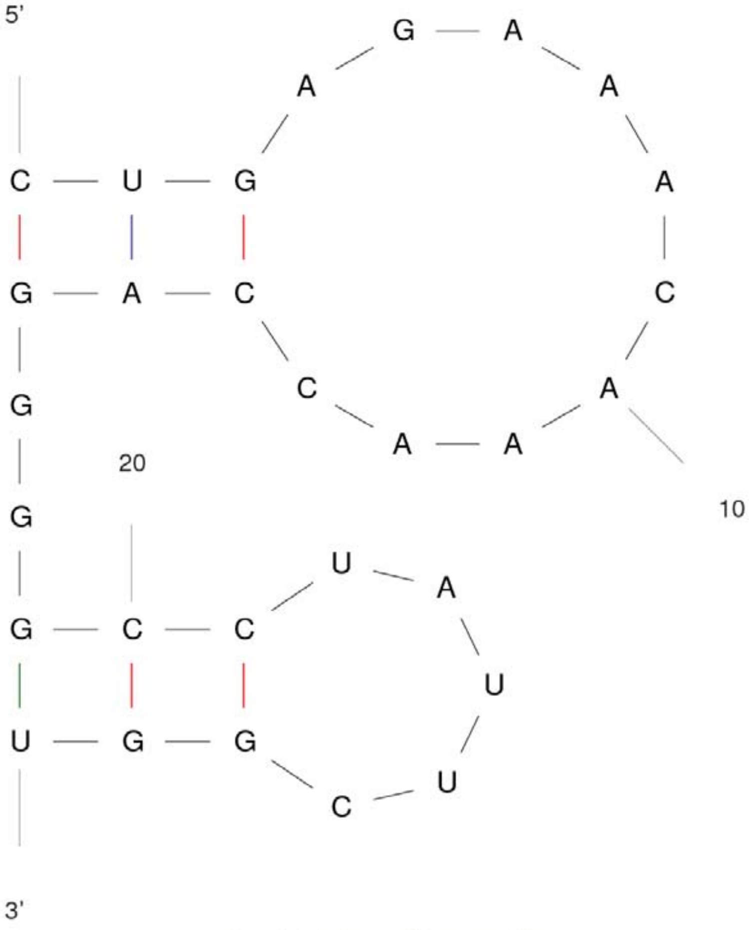

dG = -2 A_duck_Vietnam_OIE-2382_2009 H3N2

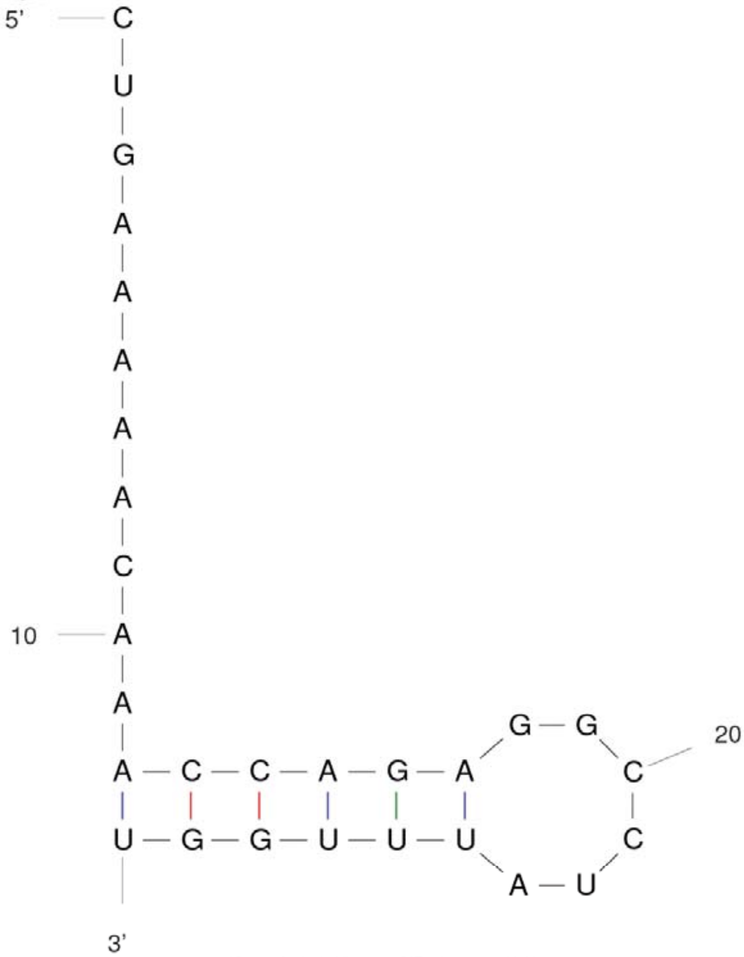

dG = -5 A_duck_Vietnam_OIE-2403_2009 H3N8

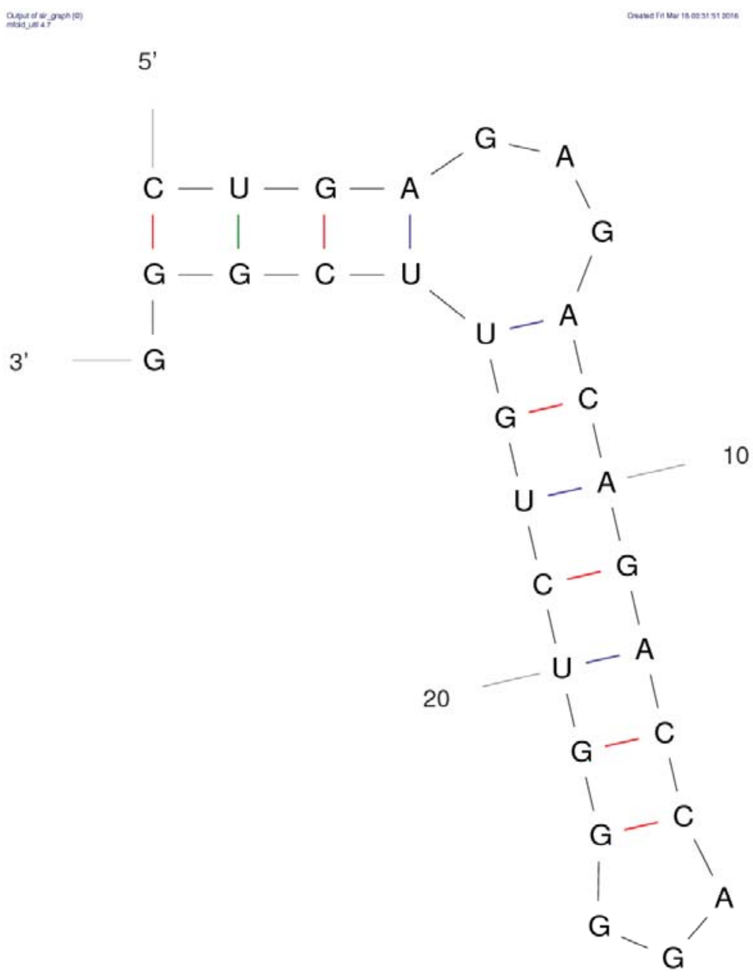

dG = -11.6 A_ruddy duck_Illinois_3471_2009 H3N8

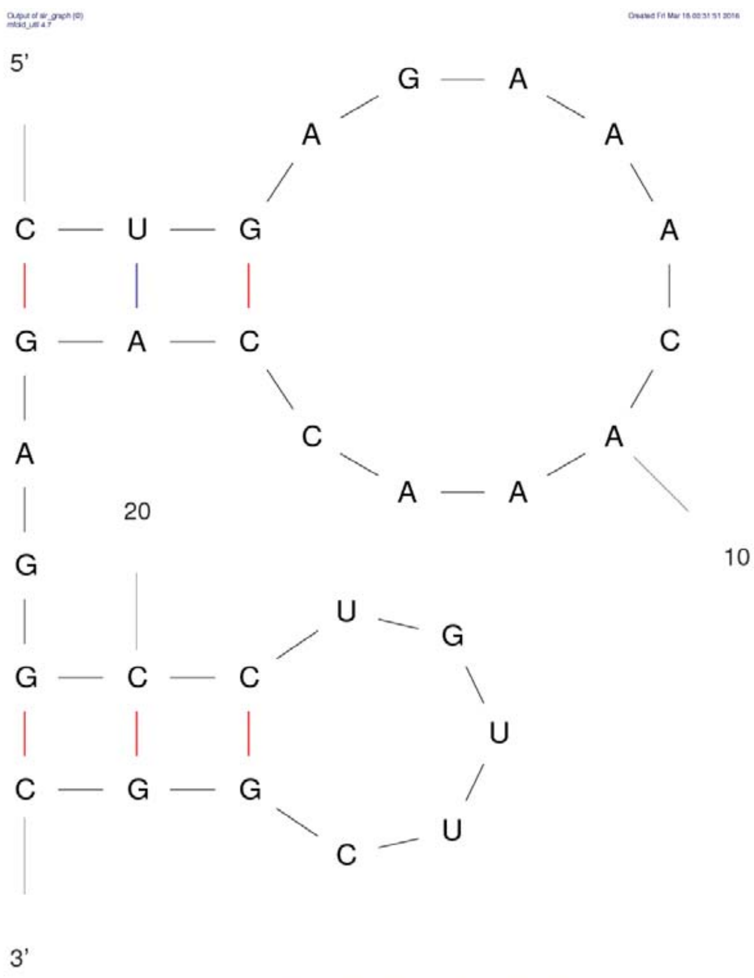

dG = -2.8 A_ruddy shelduck_Mongolia_963V_2009 H3N8

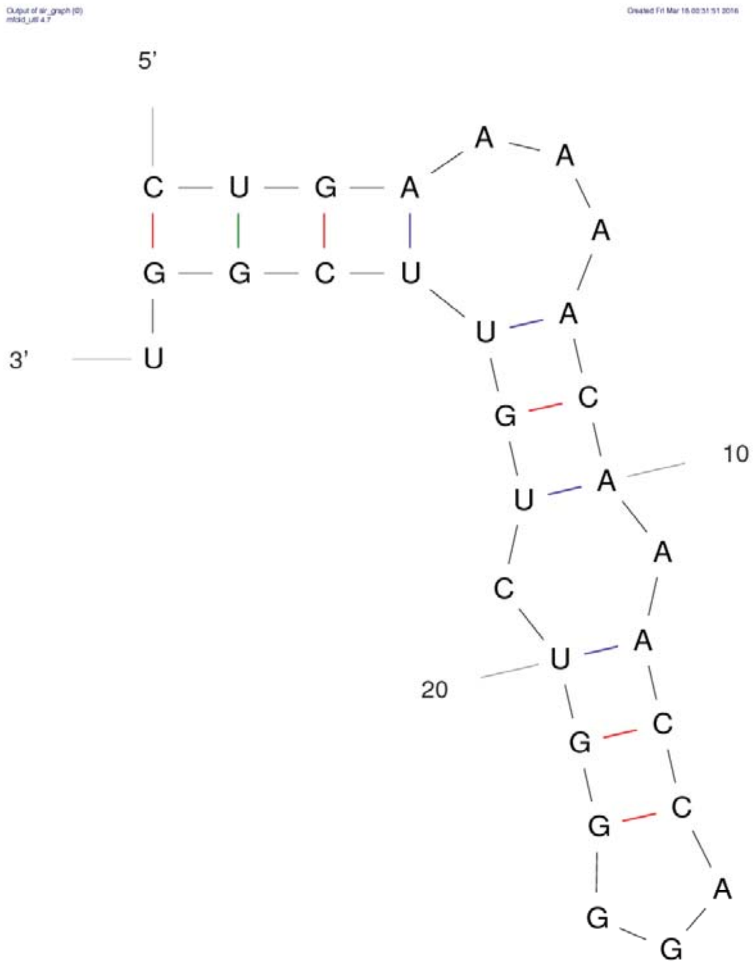

dG = -4.7 A_American black duck_New Brunswick_00322_2010 H3N8

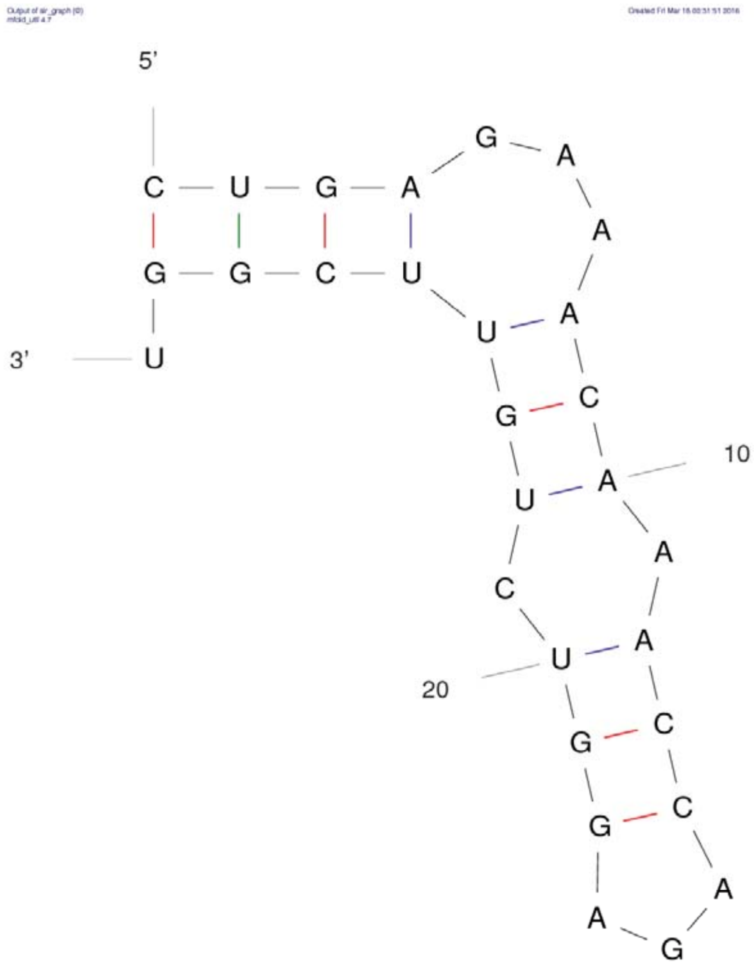

dG = -4.7 A_ruddy duck_Wisconsin_10OS3191_2010 H3N8

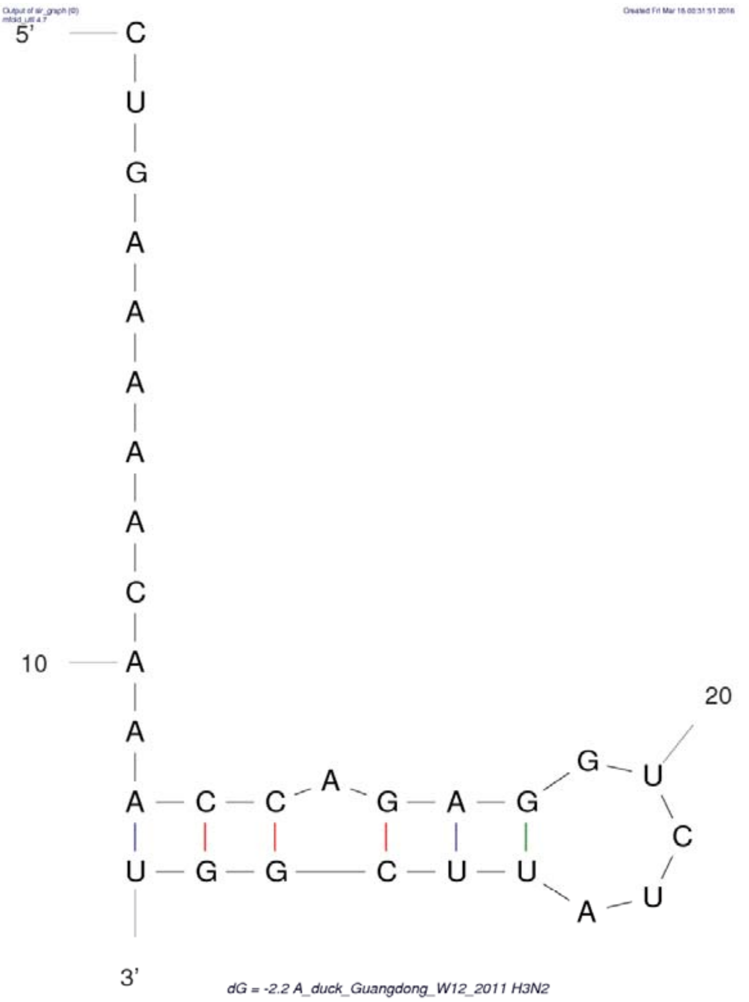

dG = -2.2 A_duck_Guangdong_W12_2011 H3N2

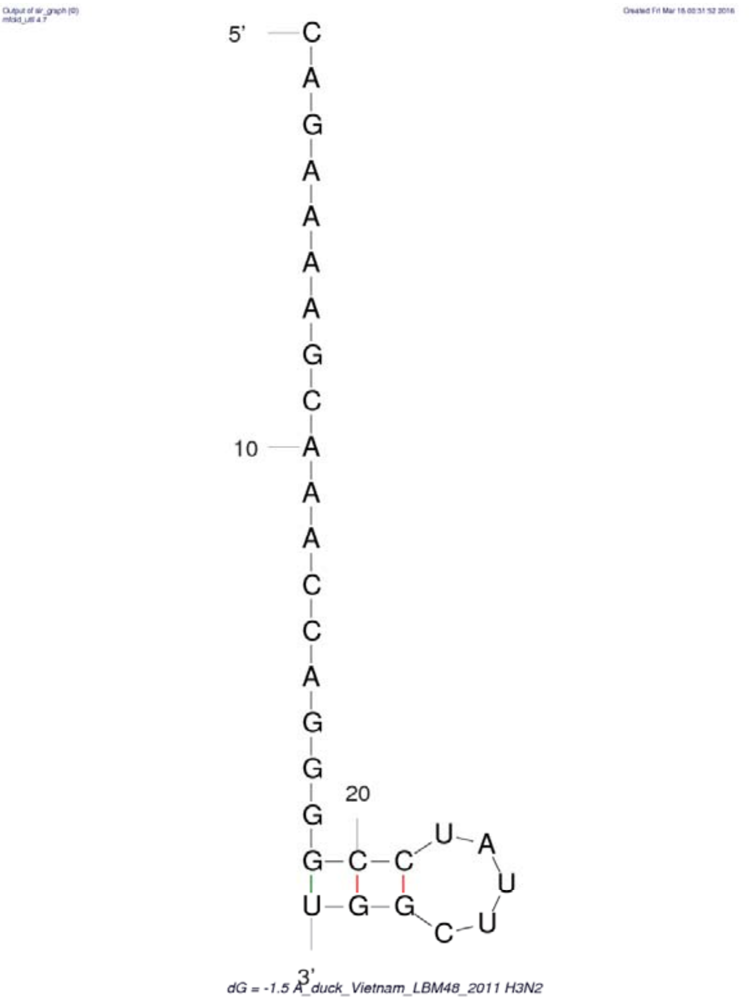

dG = -1.5 A_duck_Vietnam_LBM48_2011 H3N2

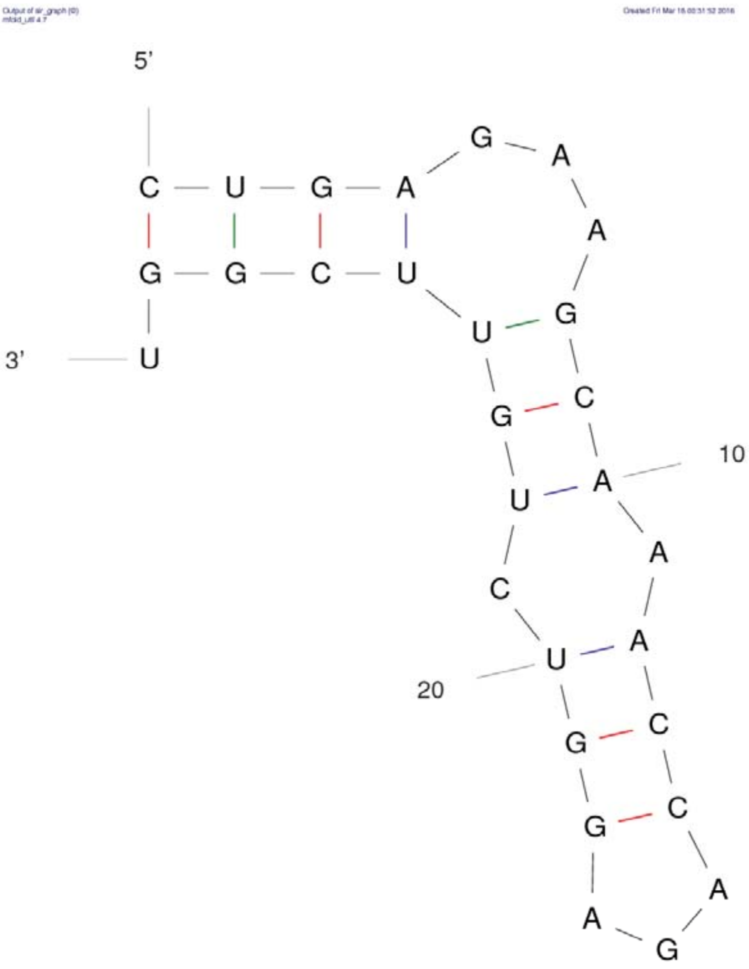

dG = -5 A_duck_Zhejiang_5_2011 H3N3

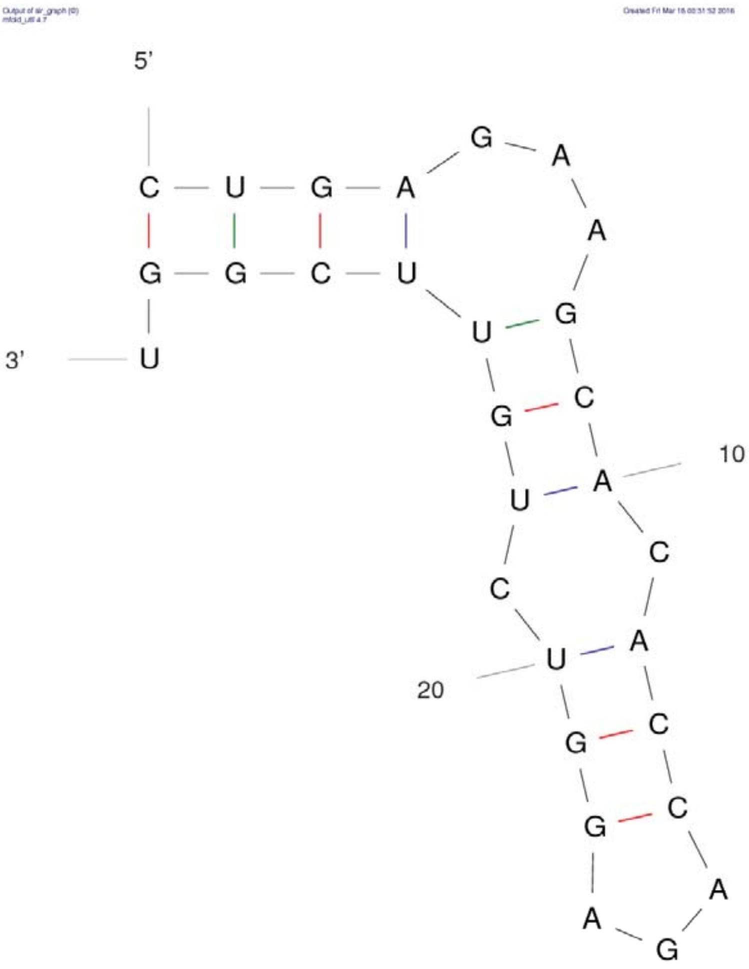

dG = -5 A_duck_Hunan_S1824_2012 H3N8

Output of sir_graph (©)
mfold_util 4.7

Created Fri Mar 18 00:31:52 2016

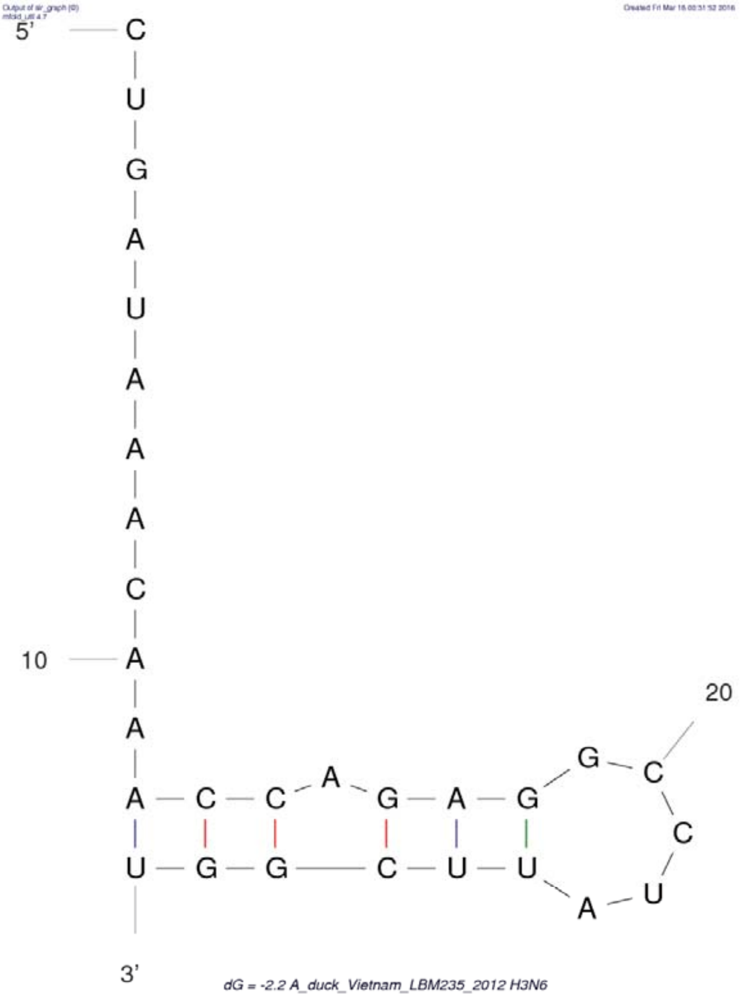

dG = -2.2 A_duck_Vietnam_LBM235_2012 H3N6

Output of sir_graph (©)
mfold_util 4.7

Created Fri Mar 18 00:31:52 2016

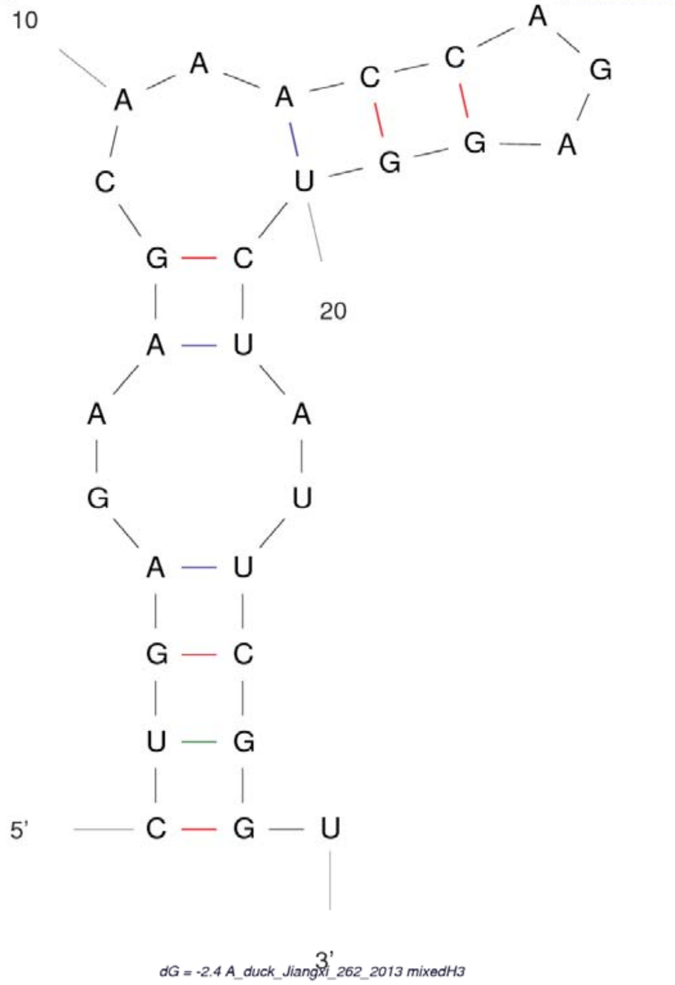

dG = -2.4 A_duck_Jiangxi_262_2013 mixedH3

Output of sir_graph (©)
mfold_util 4.7

Created Fri Mar 18 00:31:52 2016

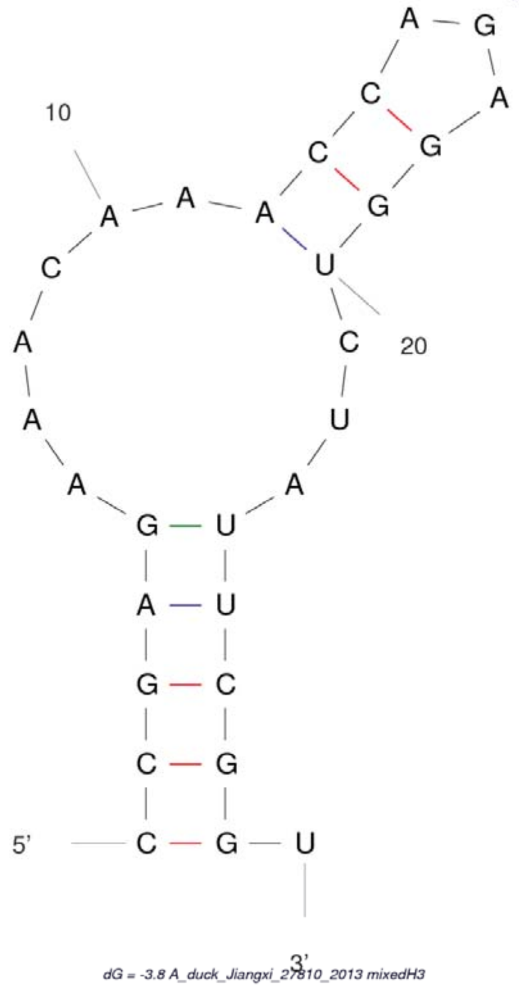

dG = -3.8 A_duck_Jiangxi_27810_2013 mixedH3

Output of sir_graph (©)
mfold_util 4.7

Created Fri Mar 18 00:31:53 2016

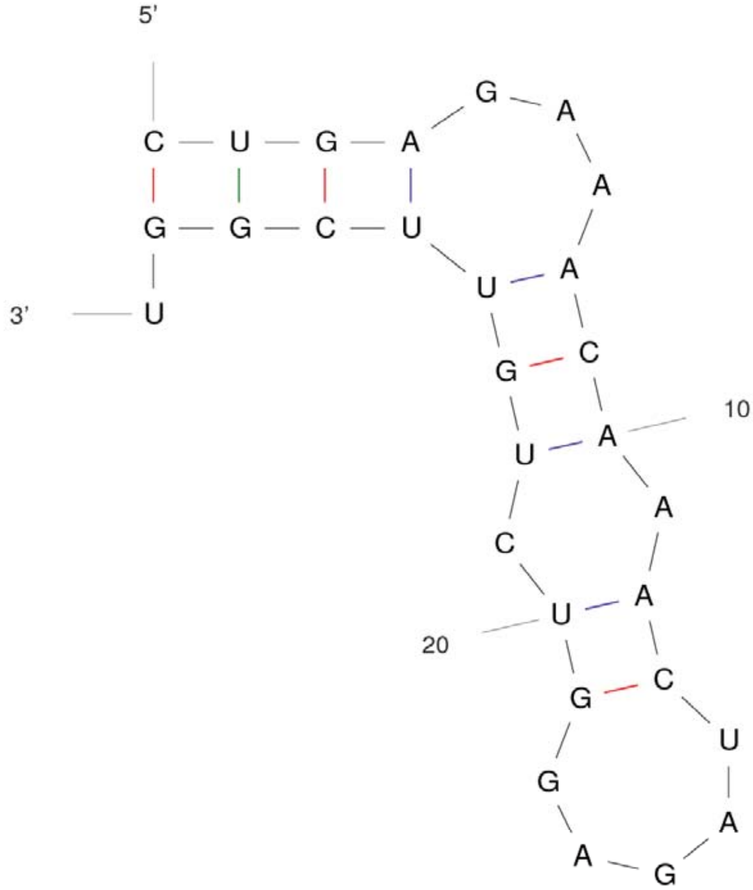

dG = -3.3 A_duck_Jiangxi_34864_2013 mixedH3

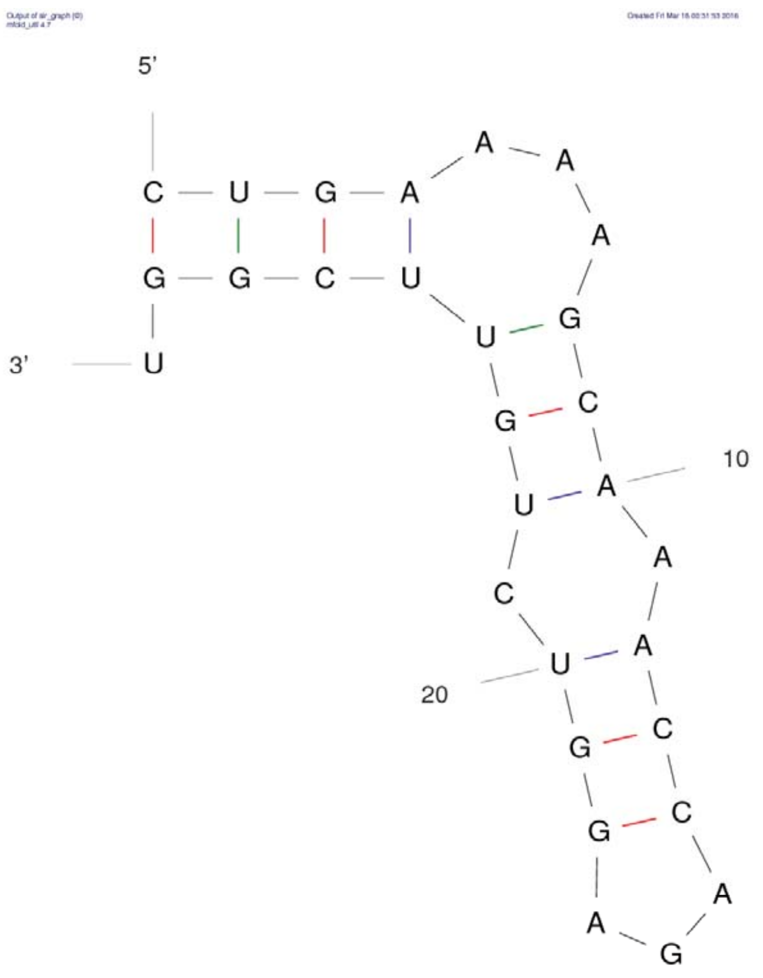

dG = -5 A_duck_Shanghai_SH1_2013 H3N2

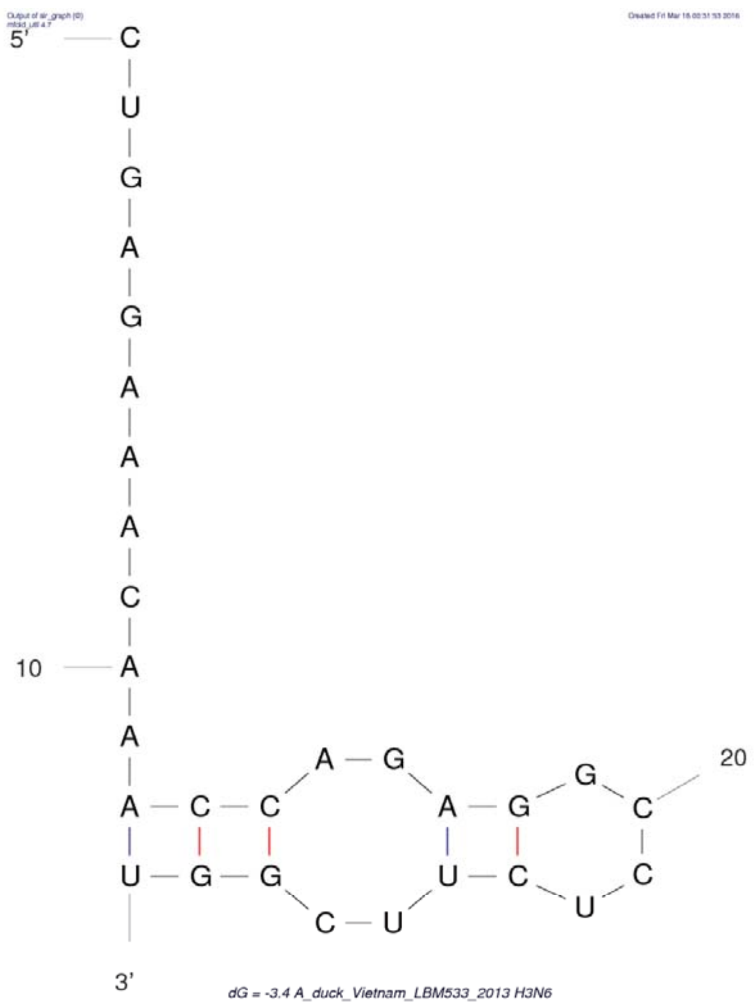

dG = -3.4 A_duck_Vietnam_LBM533_2013 H3N6

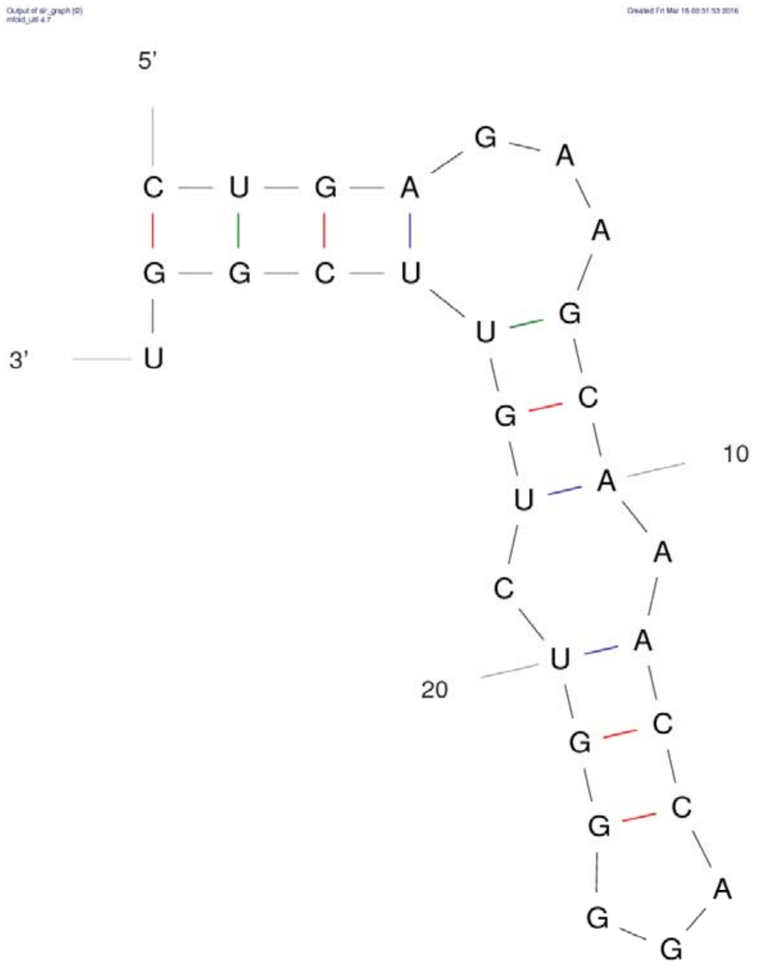

dG = -5 A_wood duck_Ohio_13OS3300_2013 H3N8

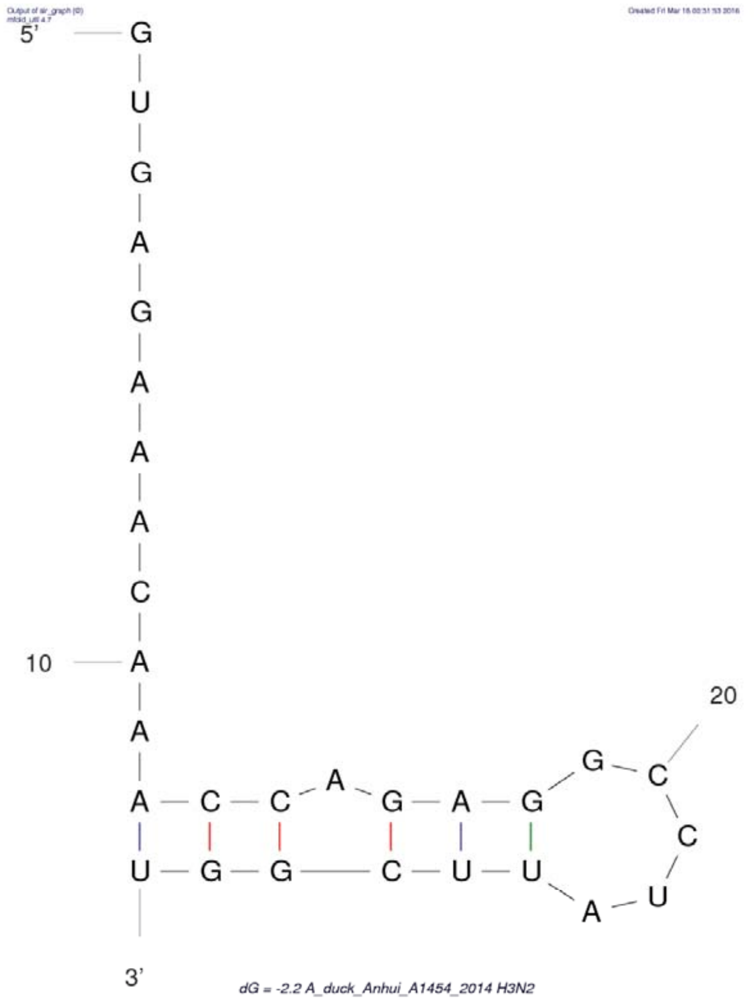

dG = -2.2 A_duck_Anhui_A1454_2014 H3N2

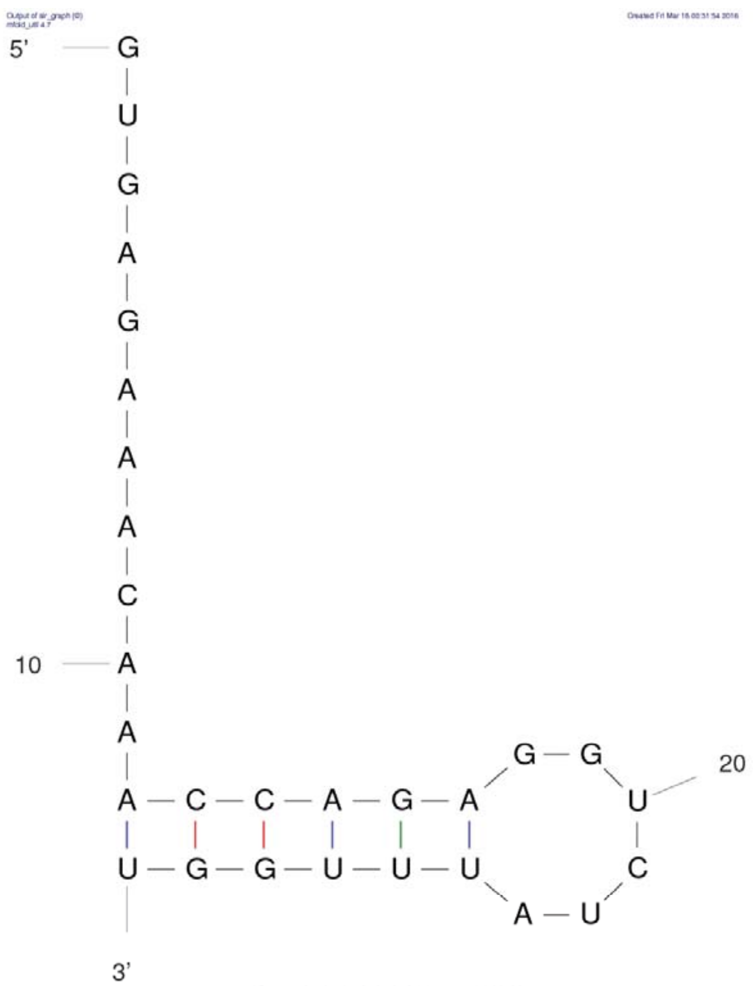

dG = -5 A_duck_Anhui_A1458_2014 H3N2

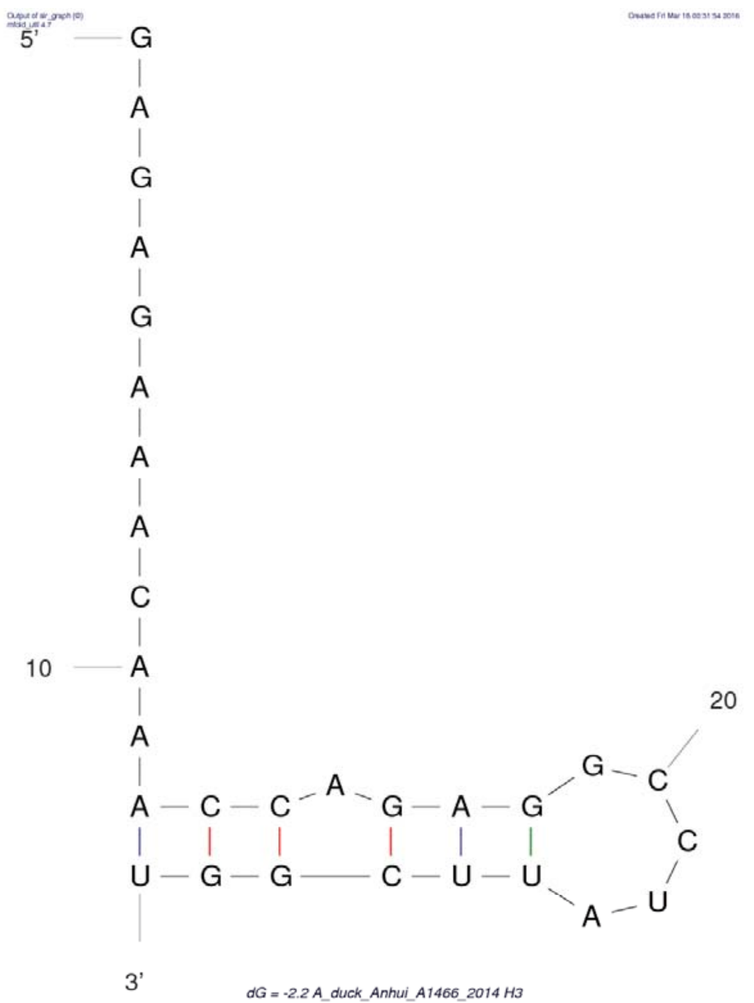

dG = -2.2 A_duck_Anhui_A1466_2014 H3

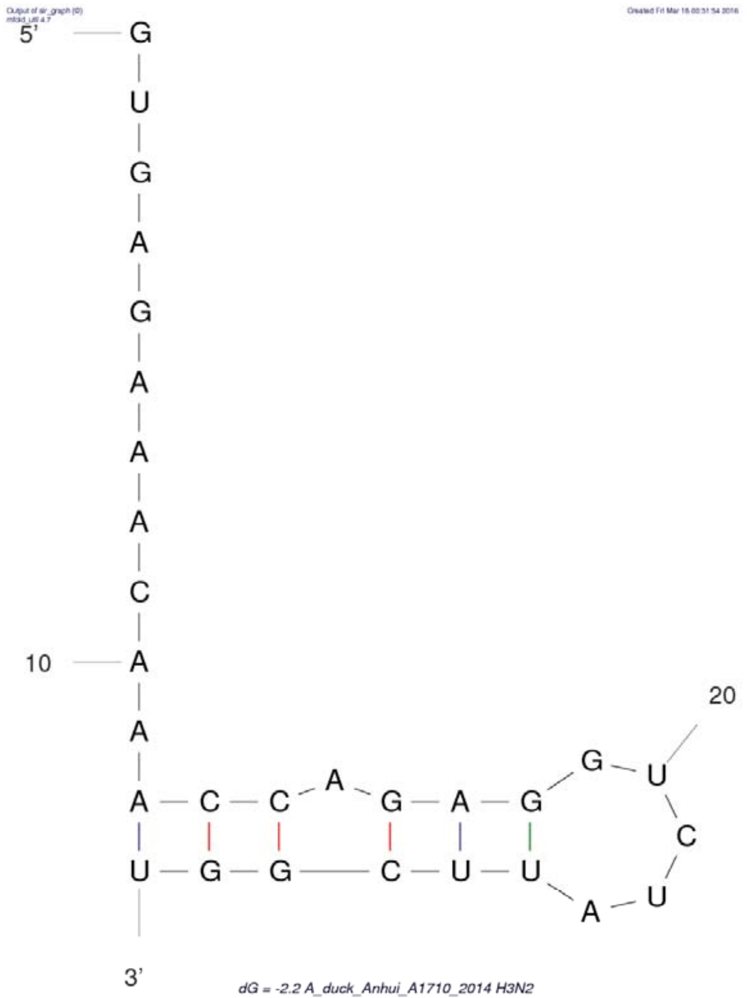

dG = -2.2 A_duck_Anhui_A1710_2014 H3N2

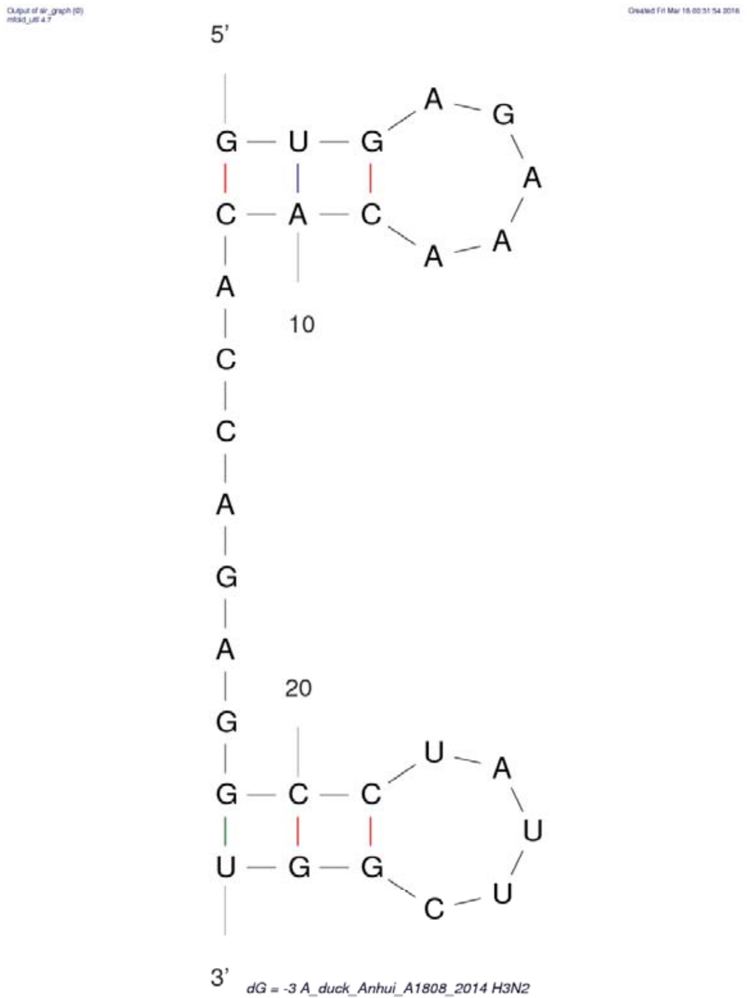

dG = -3 A_duck_Anhui_A1808_2014 H3N2

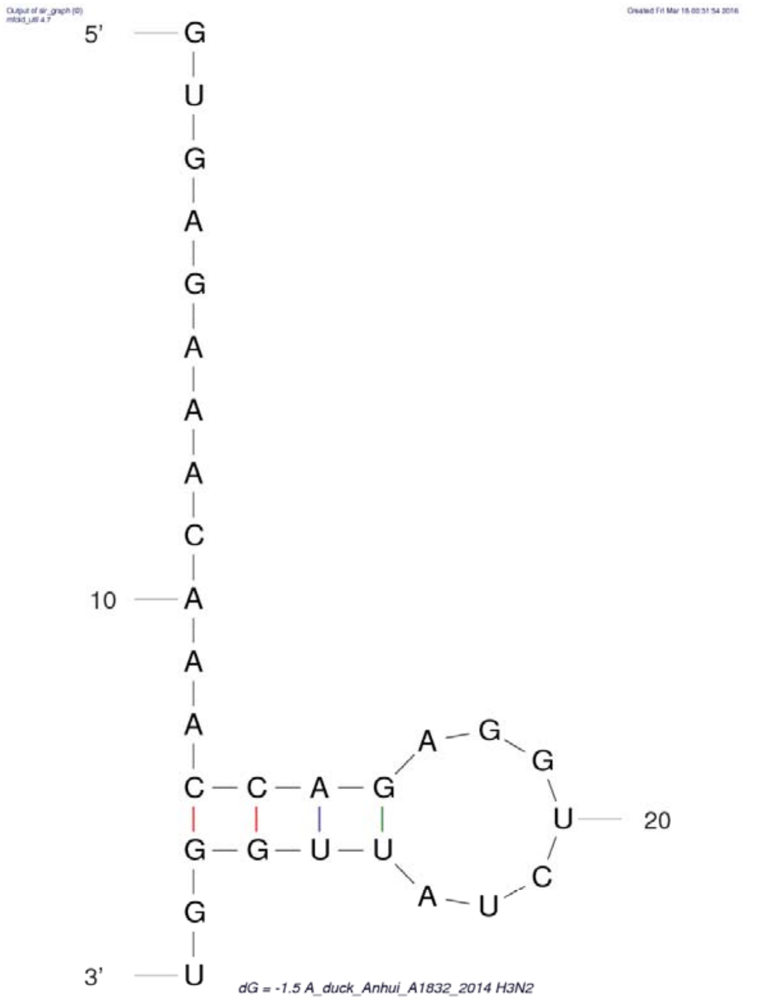

dG = -1.5 A_duck_Anhui_A1832_2014 H3N2

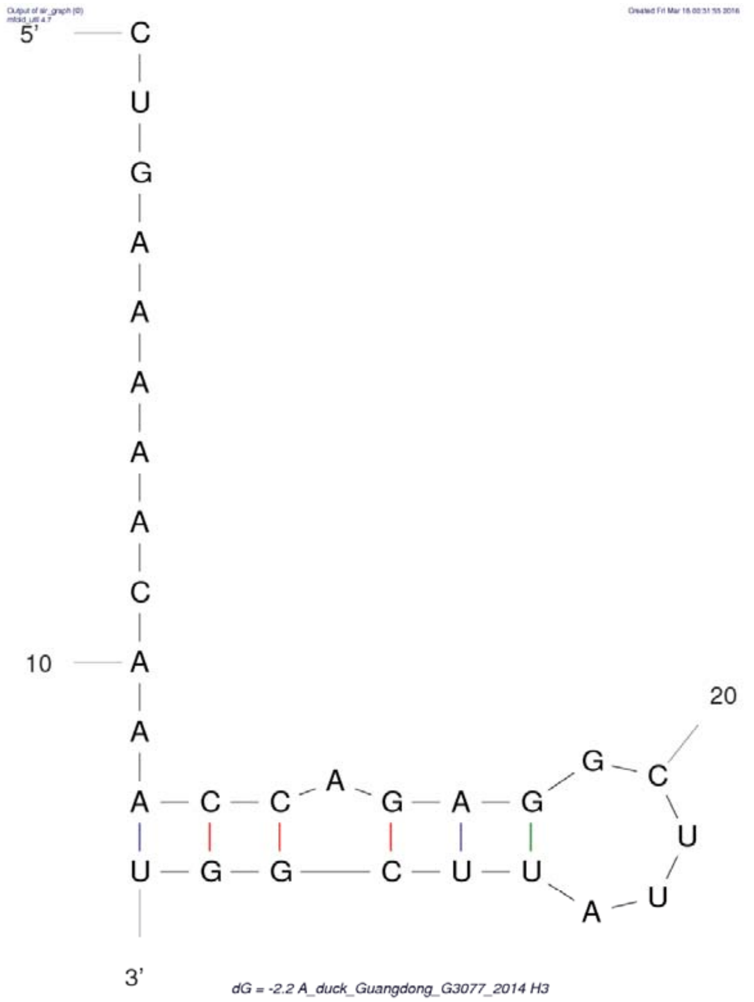

dG = -2.2 A_duck_Guangdong_G3077_2014 H3

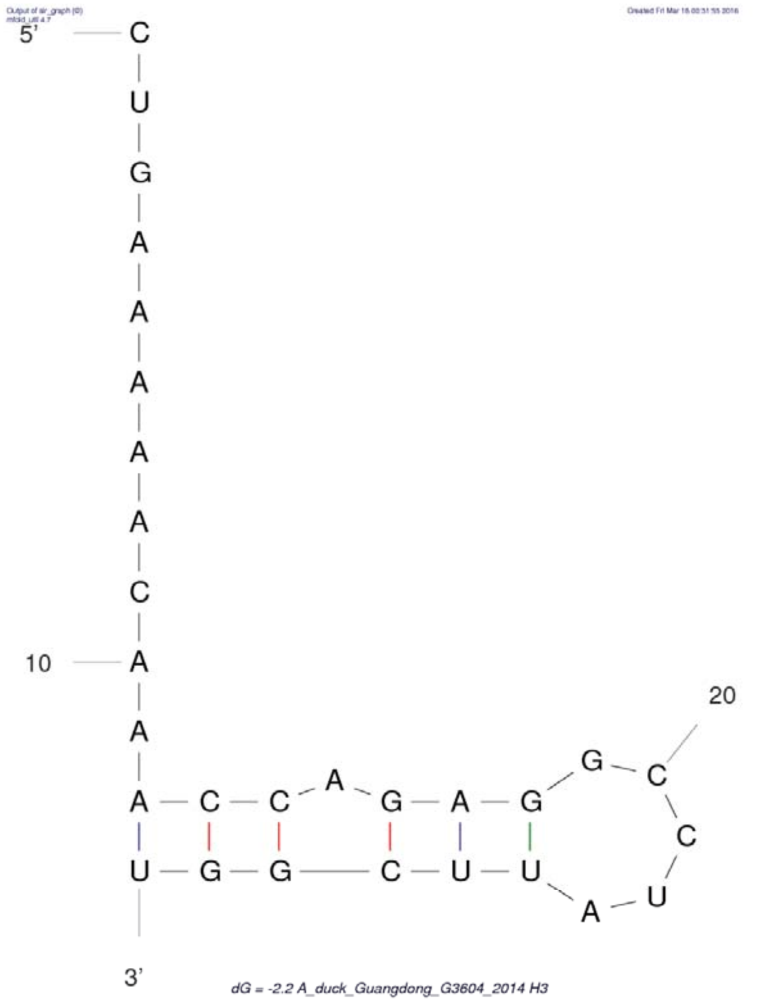

dG = -2.2 A_duck_Guangdong_G3604_2014 H3

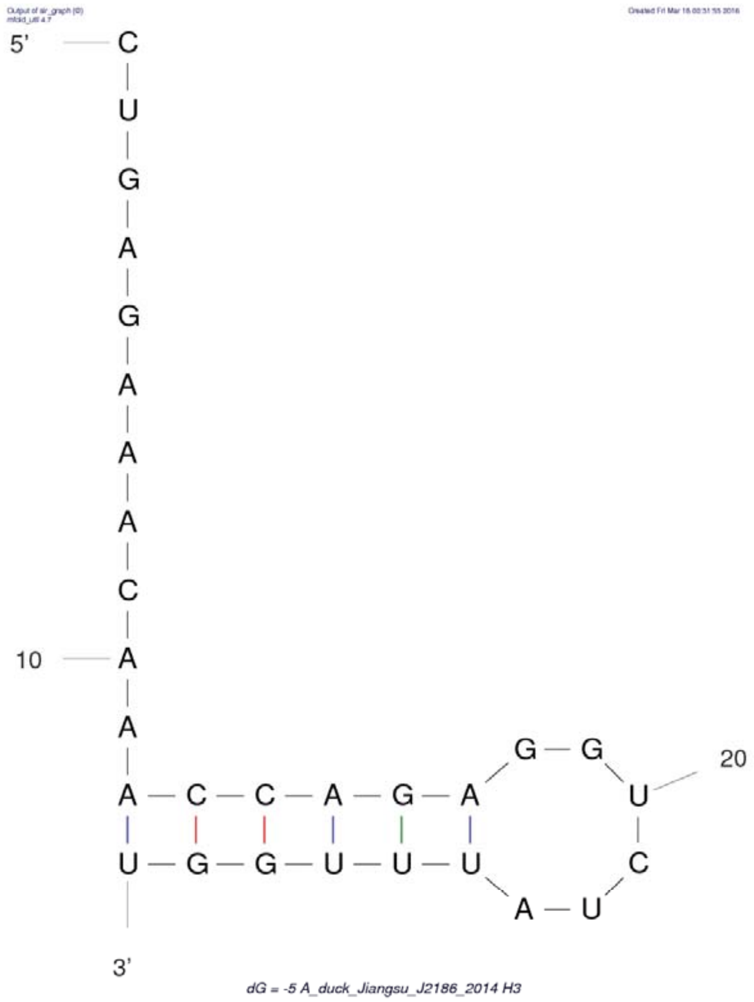

dG = -5 A_duck_Jiangsu_J2186_2014 H3

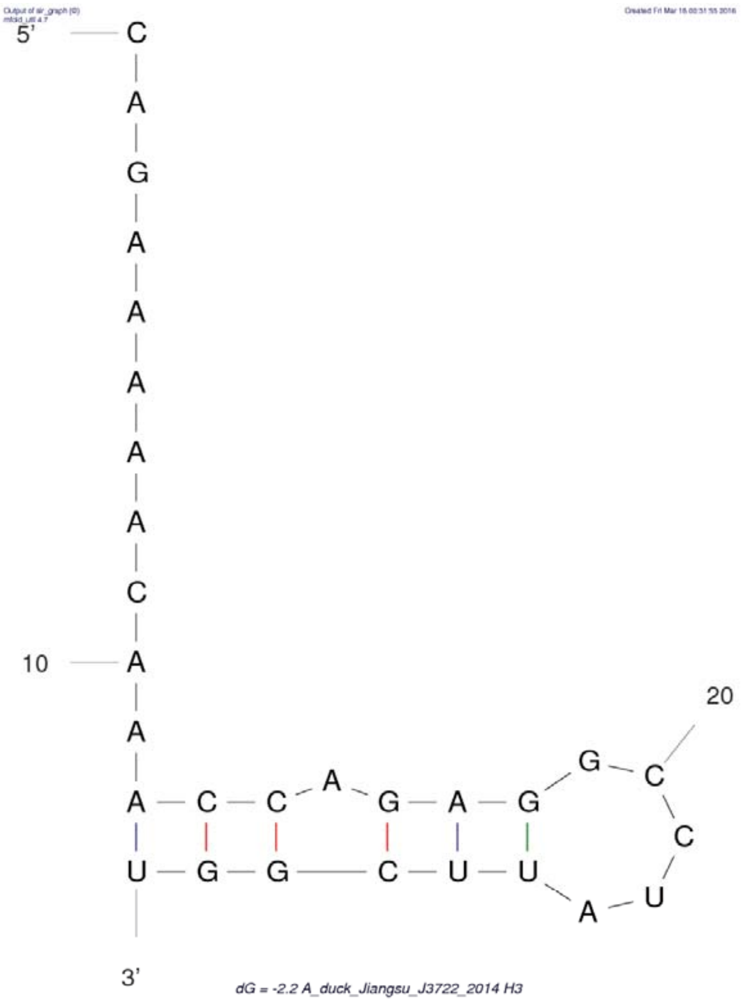

dG = -2.2 A_duck_Jiangsu_J3722_2014 H3

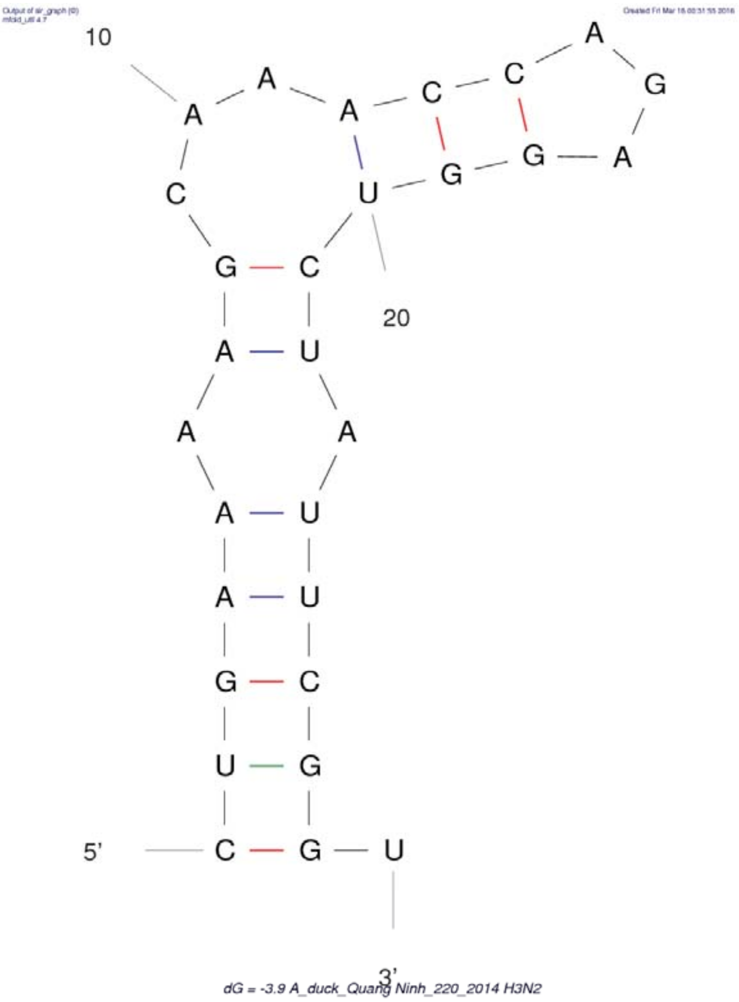

dG = -3.9 A_duck_Quang Ninh_220_2014 H3N2

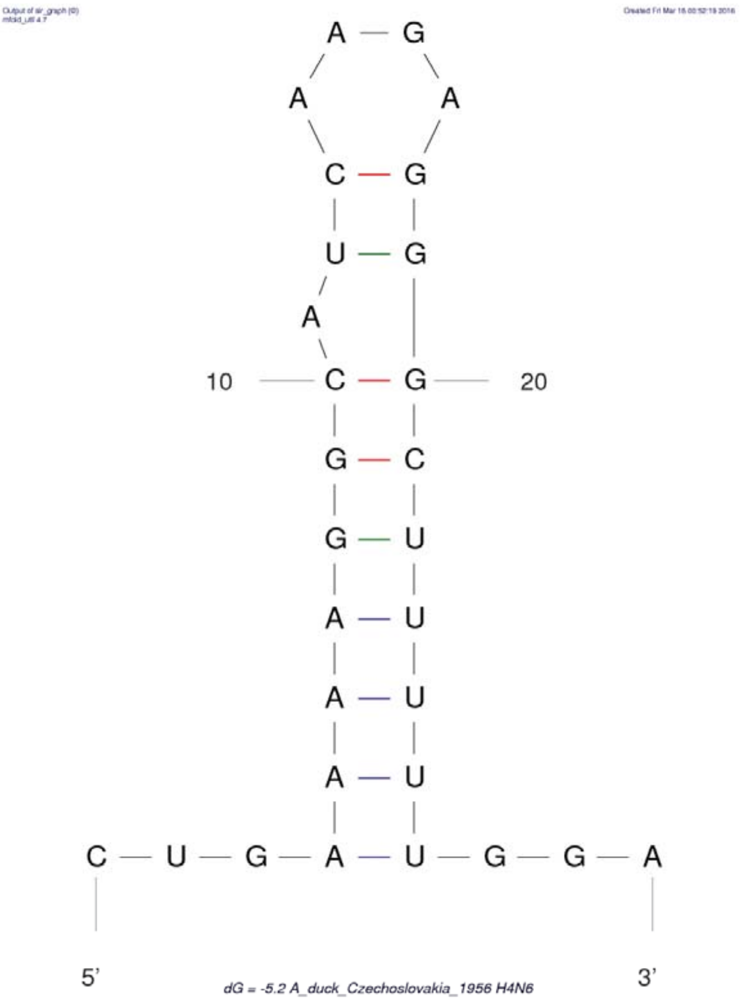

dG = -5.2 A_duck_Czechoslovakia_1956 H4N6

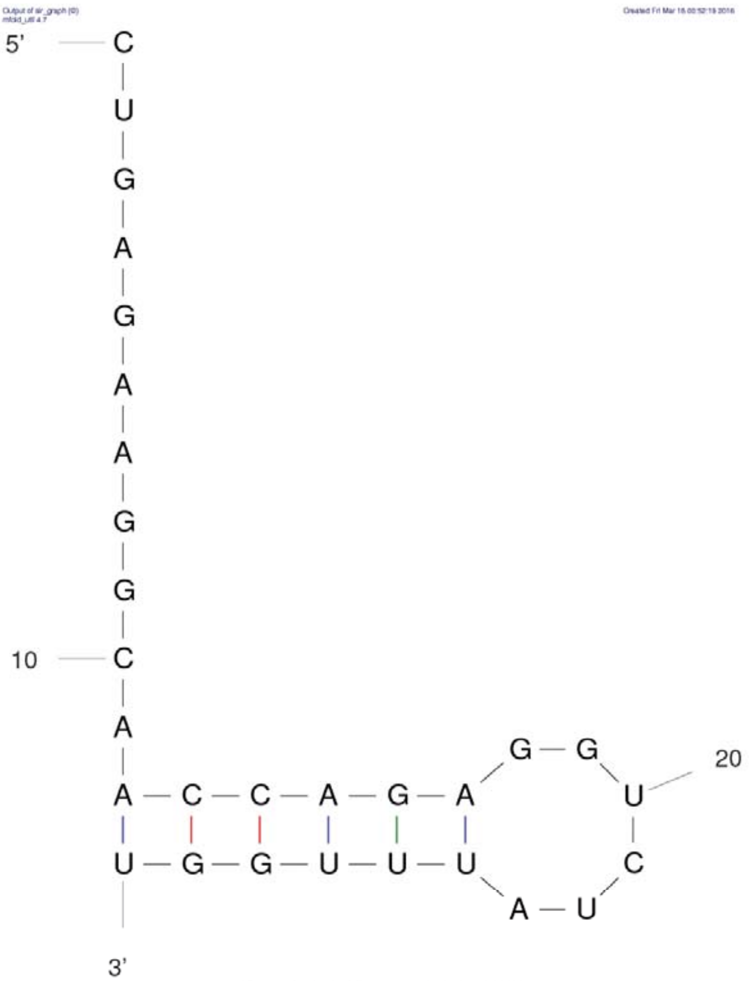

dG = -5 A_duck_Minnesota_104_1974 H4N6

Output of sir_graph (©)
mfold_util 4.7

Created Fri Mar 18 00:52:19 2016

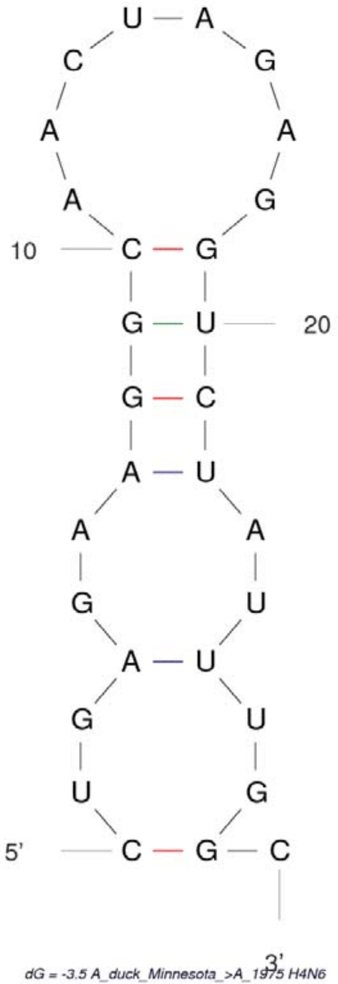

dG = -3.5 A_duck_Minnesota_>A_1975 H4N6

Output of sir_graph (©)
mfold_util 4.7

Created Fri Mar 18 00:52:19 2016

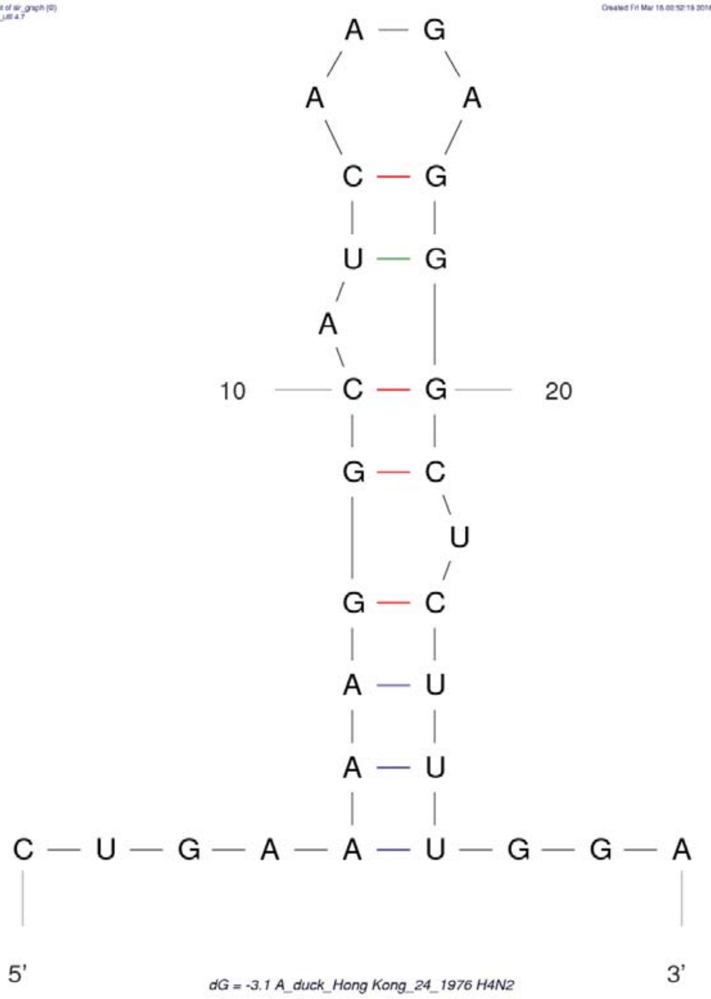

dG = -3.1 A_duck_Hong Kong_24_1976 H4N2

Output of sir_graph (©)
mfold_util 4.7

Created Fri Mar 18 00:52:19 2016

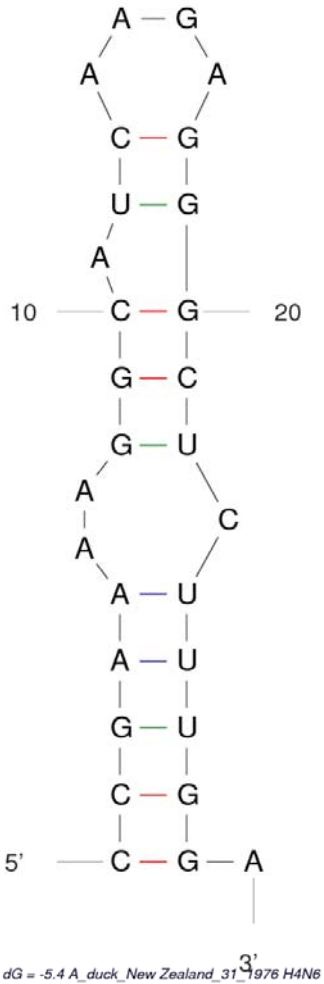

dG = -5.4 A_duck_New Zealand_31_1976 H4N6

Output of sir_graph (©)
mfold_util 4.7

Created Fri Mar 18 00:52:20 2016

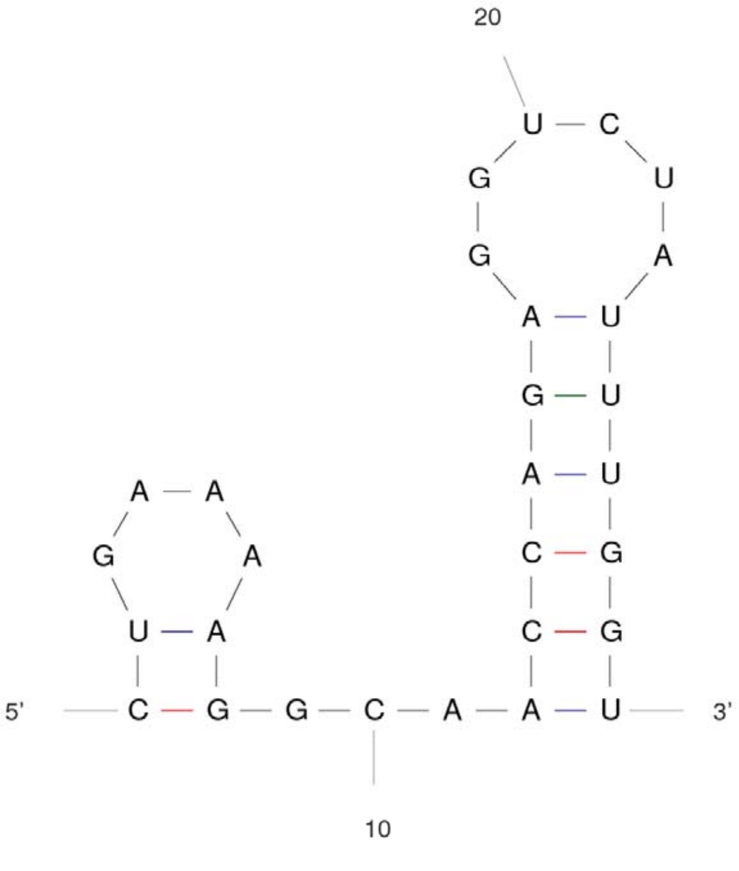

dG = -5.7 A_canvasback duck_ALB_274_1977 H4N6

Output of sir_graph (©)
mfold_util 4.7

Created Fri Mar 18 00:52:20 2016

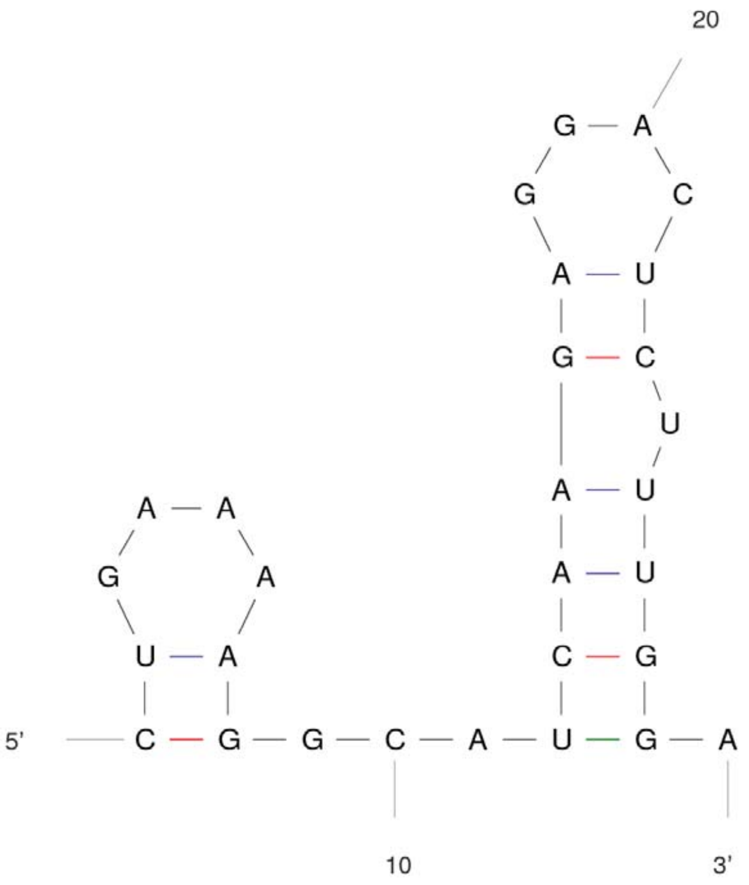

dG = -1.8 A_duck_Hong Kong_438_1977 H4N8

Output of sir_graph (©)
mfold_util 4.7

Created Fri Mar 18 00:52:20 2016

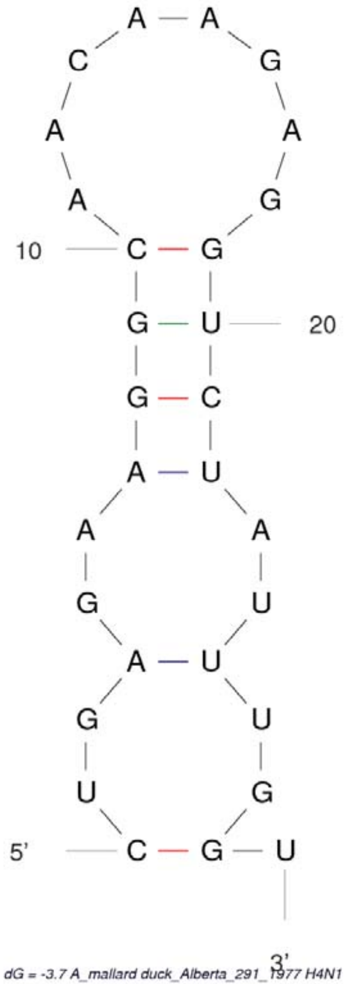

dG = -3.7 A_mallard duck_Alberta_291_1977 H4N1

Output of sir_graph (©)
mfold_util 4.7

Created Fri Mar 18 00:52:20 2016

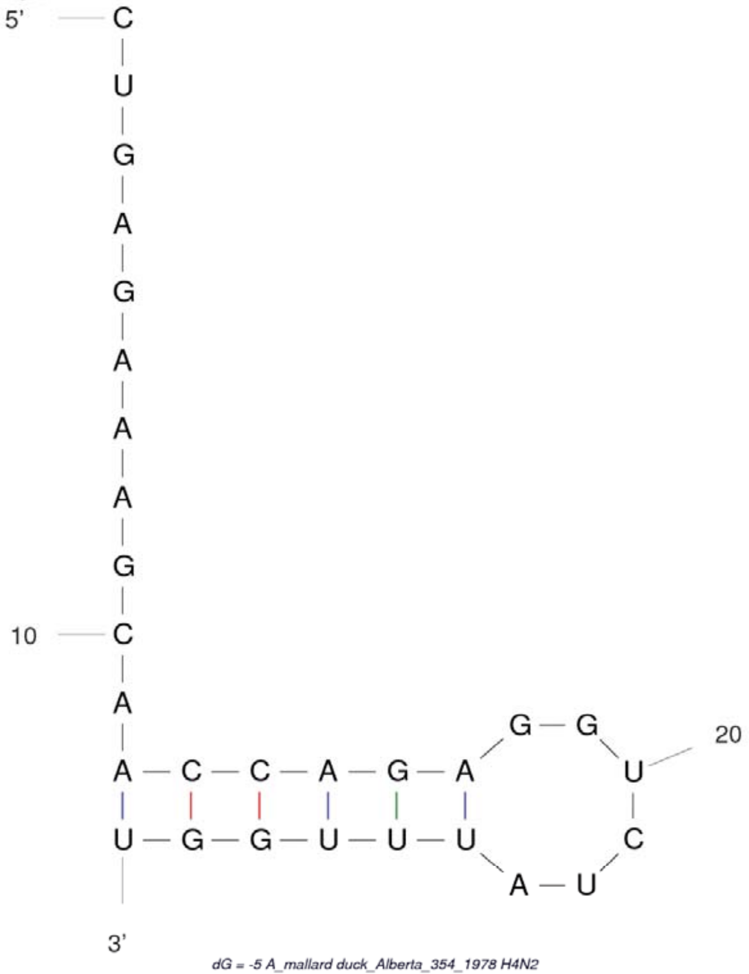

dG = -5 A_mallard duck_Alberta_354_1978 H4N2

Output of sir_graph (©)
mfold_util 4.7

Created Fri Mar 18 00:52:20 2016

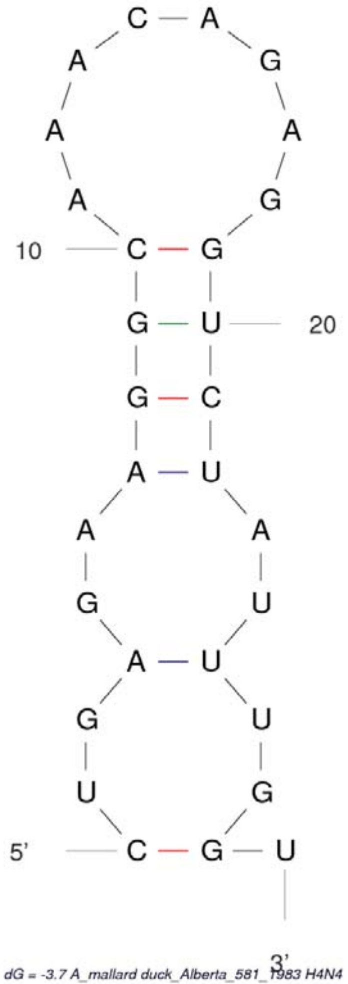

dG = -3.7 A_mallard duck_Alberta_581_1983 H4N4

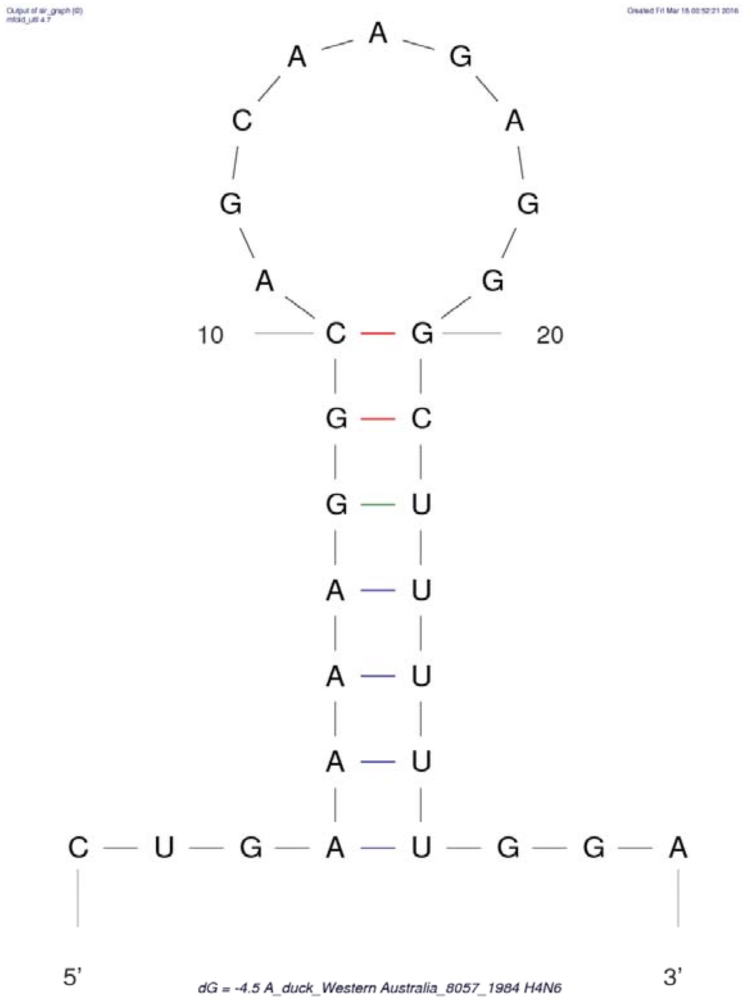

dG = -4.5 A_duck_Western Australia_8057_1984 H4N6

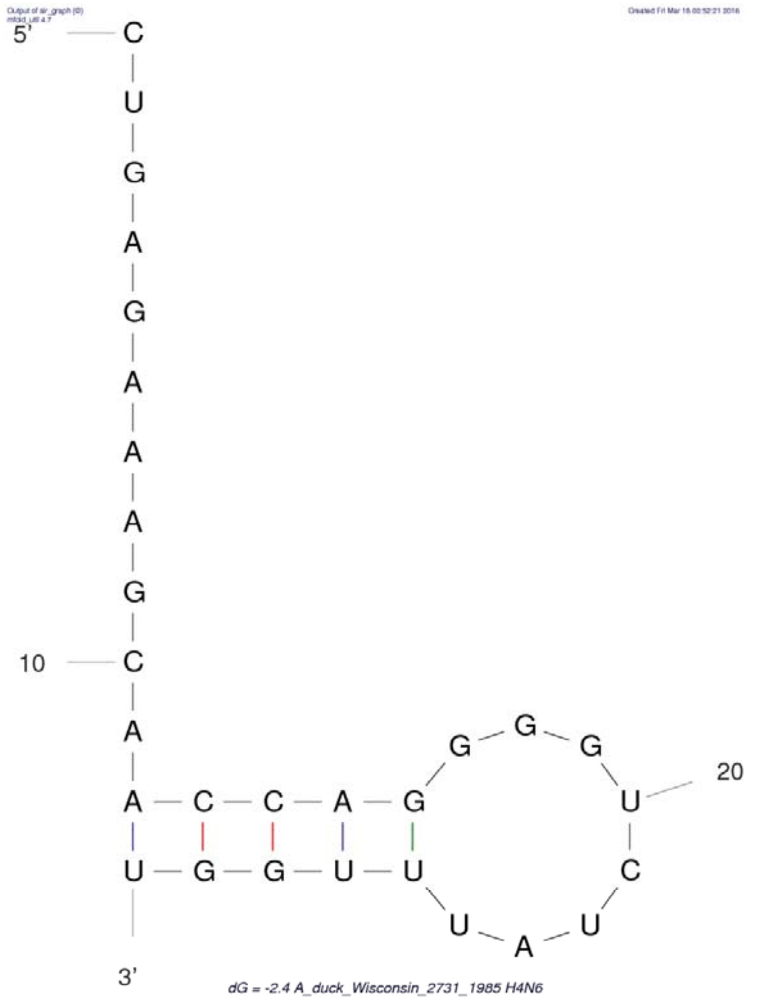

dG = -2.4 A_duck_Wisconsin_2731_1985 H4N6

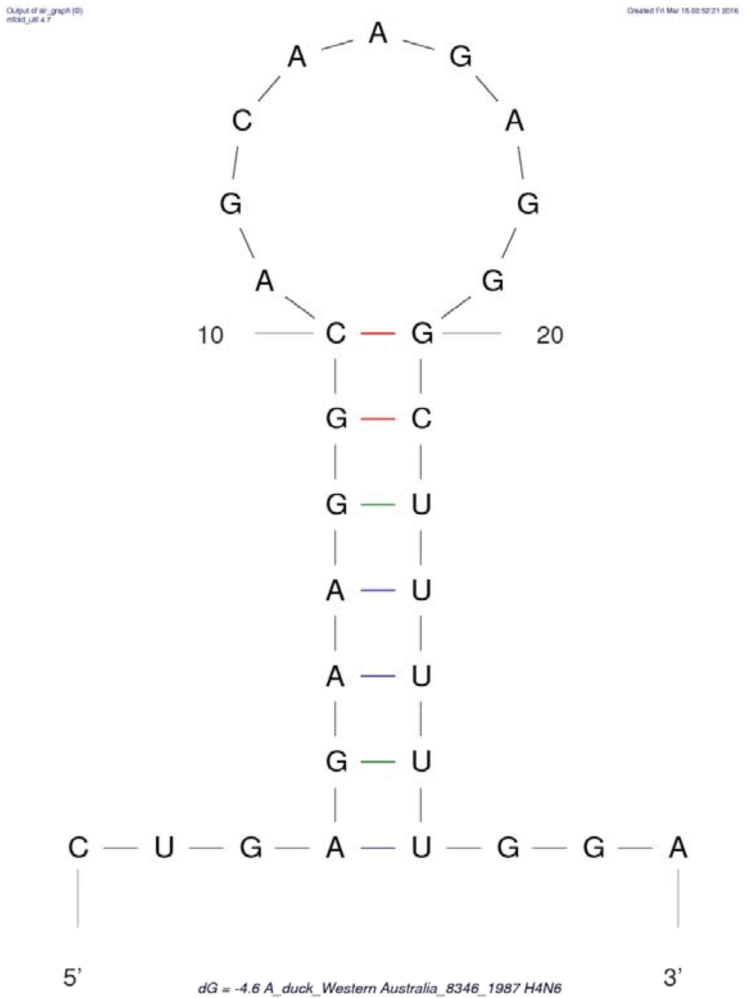

dG = -4.6 A_duck_Western Australia_8346_1987 H4N6

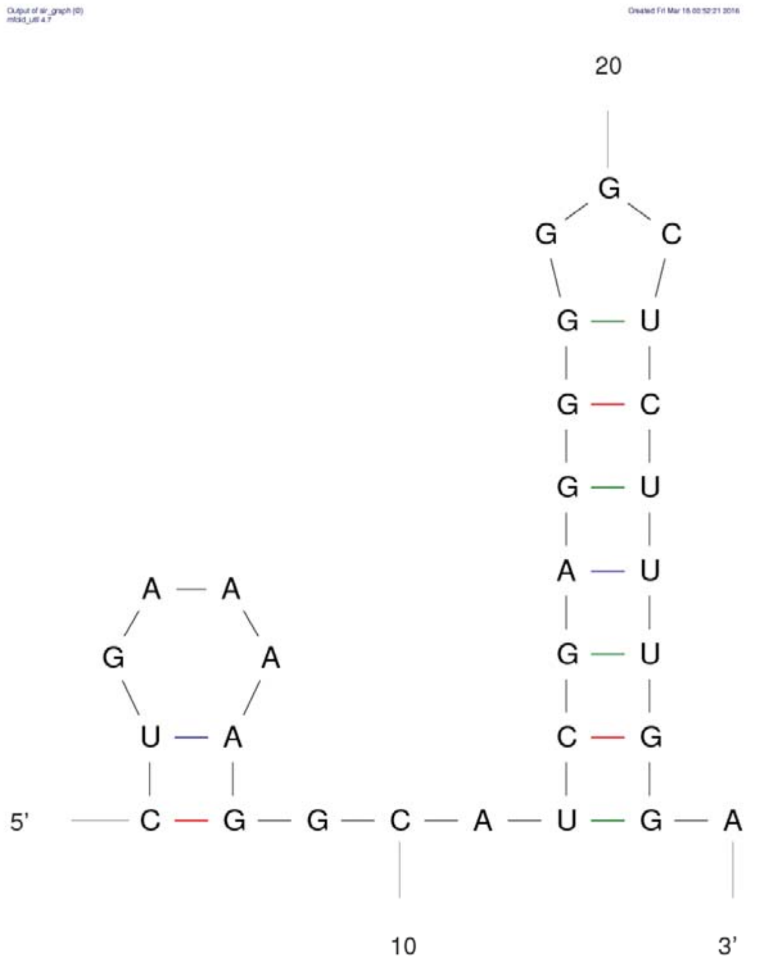

dG = -5.4 A_duck_Taiwan_wb1_1998 H4N2

Output of sir_graph (©)
mfold_util 4.7

Created Fri Mar 18 00:52:21 2016

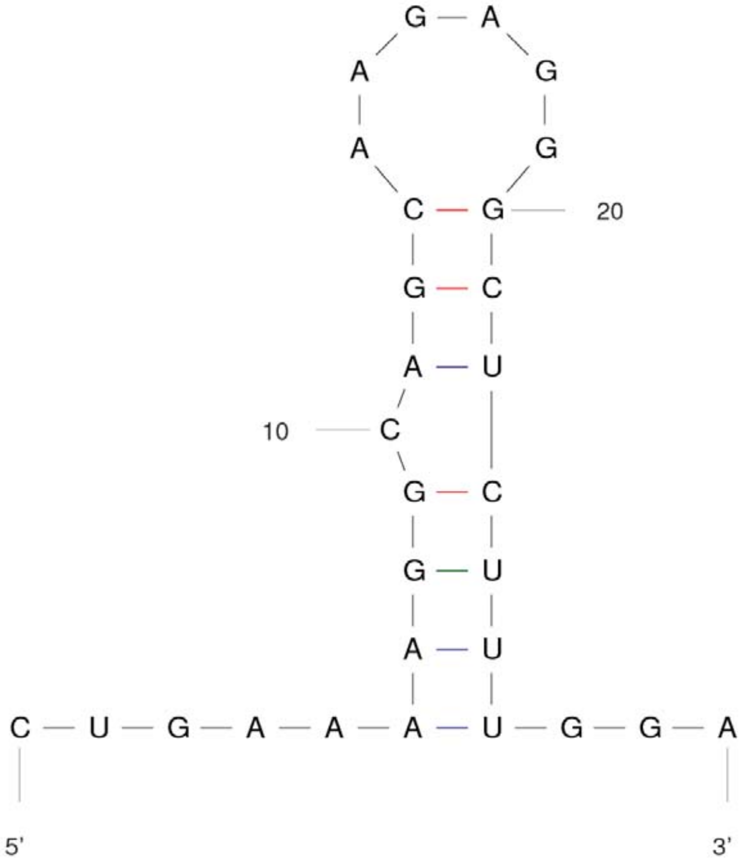

dG = -4.3 A_duck_Taiwan_wb14_1998 H4N6

Output of sir_graph (©)
mfold_util 4.7

Created Fri Mar 18 00:52:22 2016

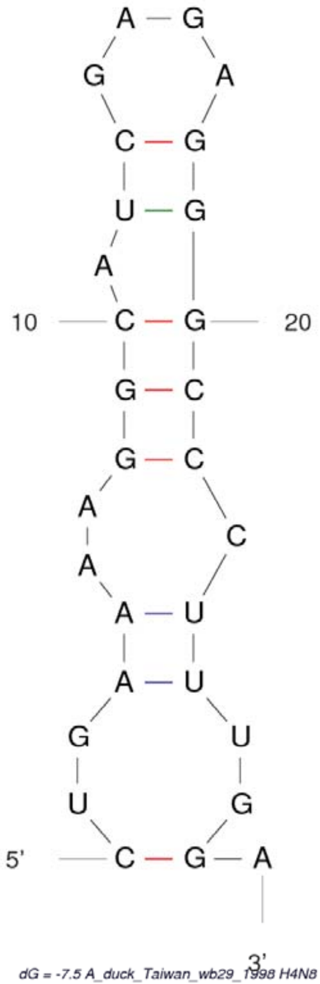

dG = -7.5 A_duck_Taiwan_wb29_1998 H4N8

Output of sir_graph (©)
mfold_util 4.7

Created Fri Mar 18 00:52:22 2016

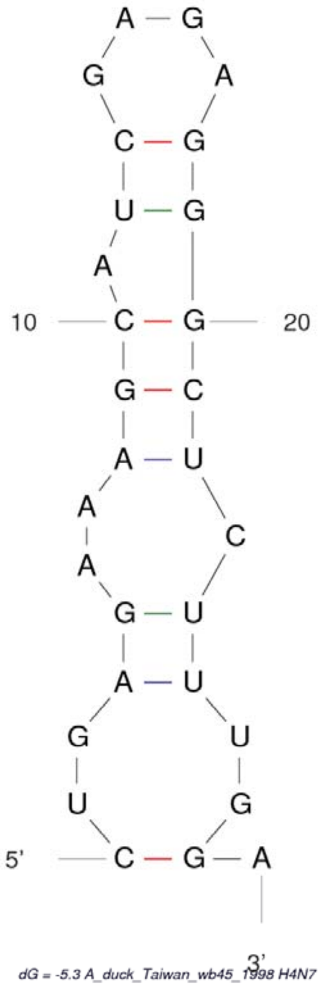

dG = -5.3 A_duck_Taiwan_wb45_1998 H4N7

Output of sir_graph (©)
mfold_util 4.7

Created Fri Mar 18 00:52:22 2016

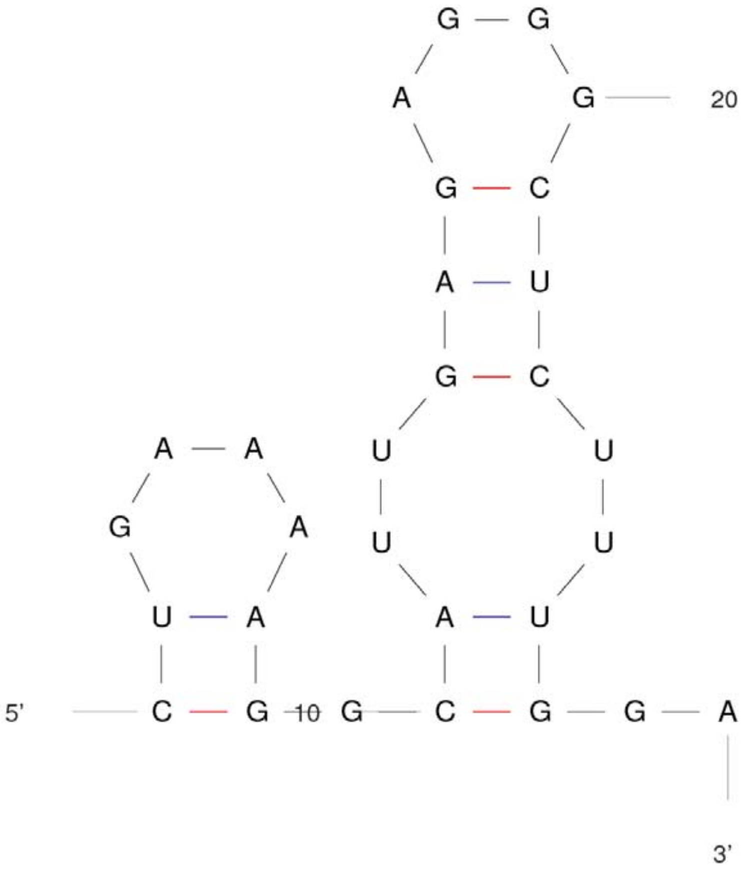

dG = -4.2 A_duck_Hokkaido_1058_2001 H4N5

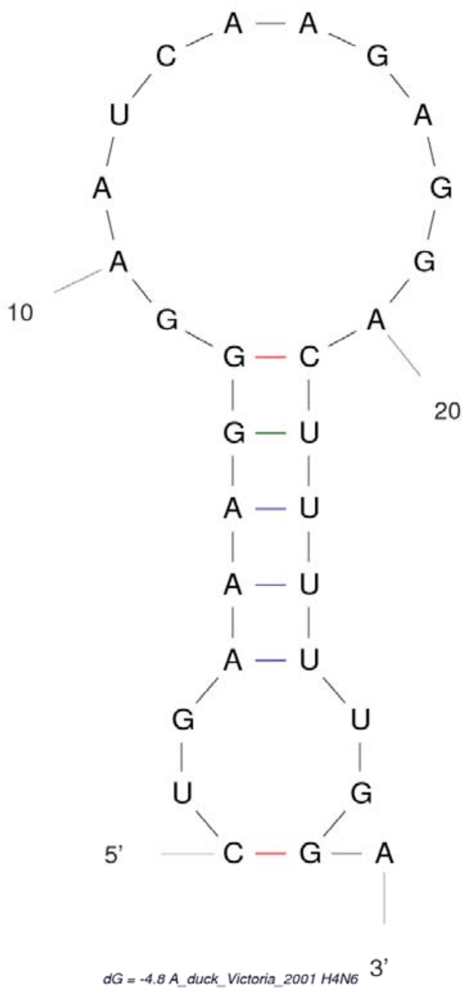

dG = -4.8 A_duck_Victoria_2001 H4N6

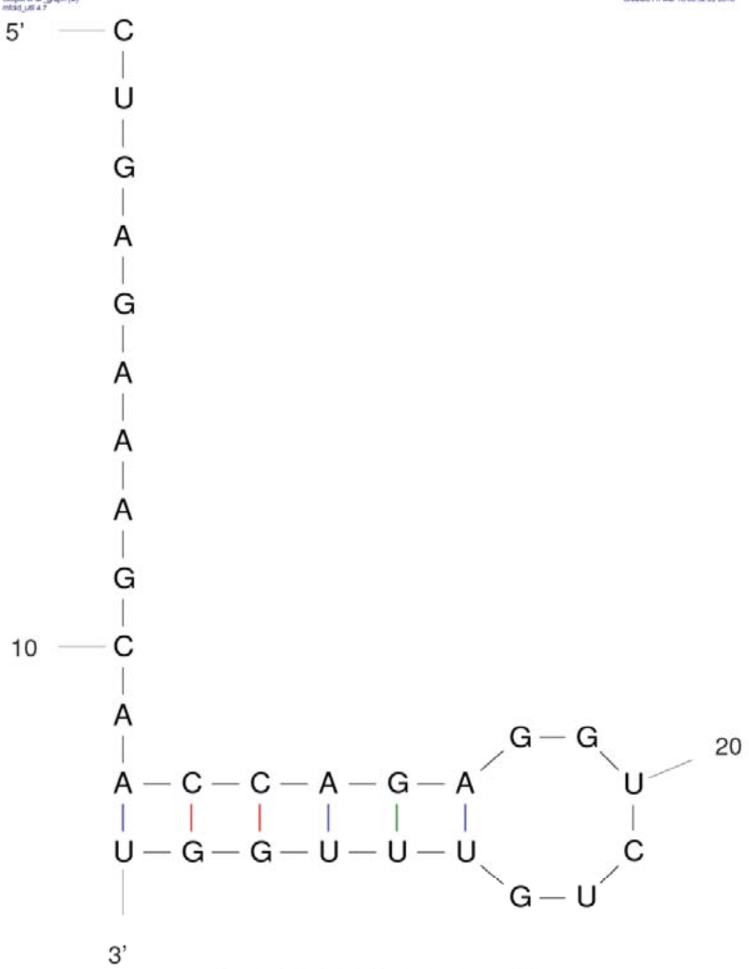

dG = -4.1 A_black duck_Maryland_834_2002 H4N8

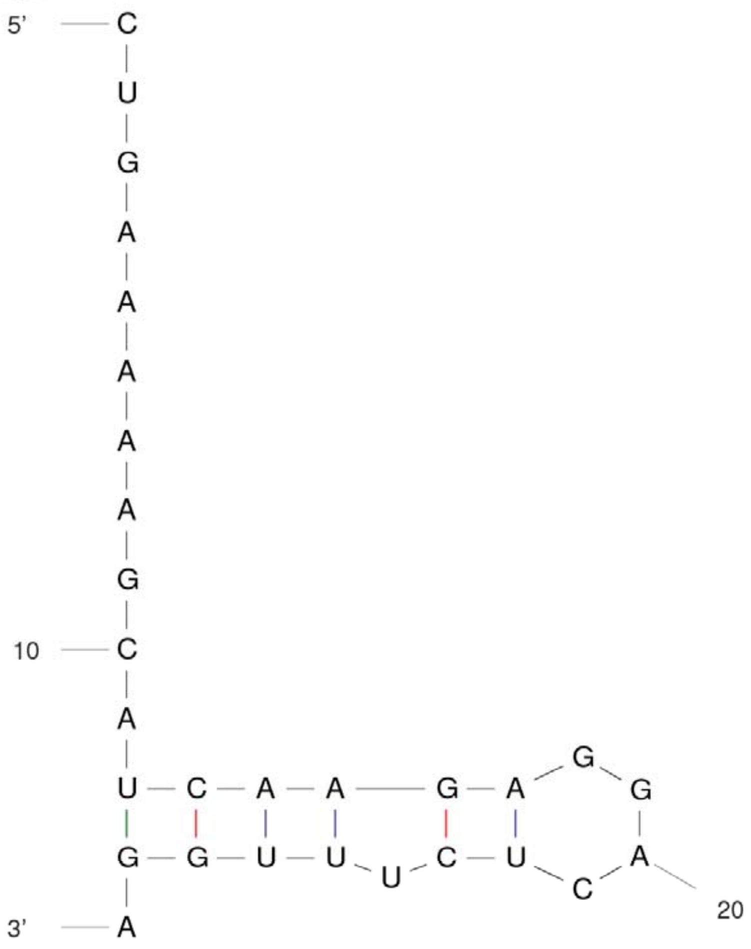

dG = -1.1 A_duck_Taiwan_wb281_2002 H4N6

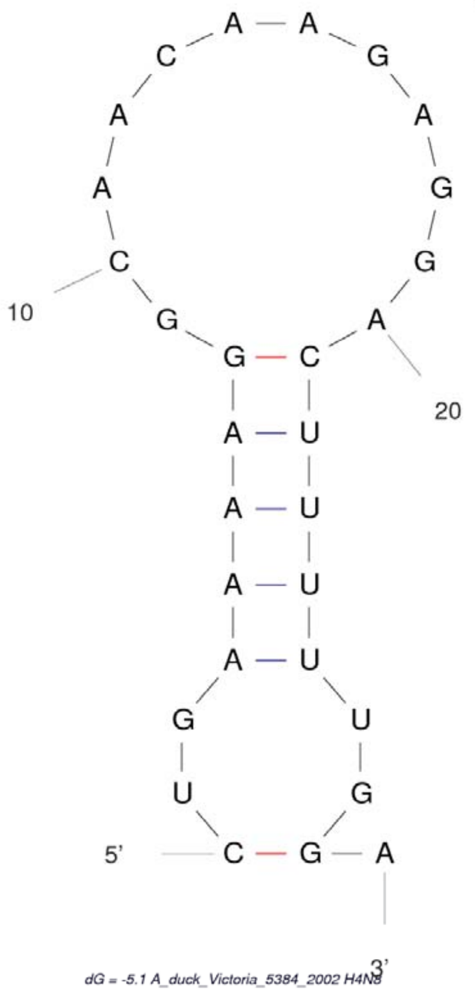

dG = -5.1 A_duck_Victoria_5384_2002 H4N8

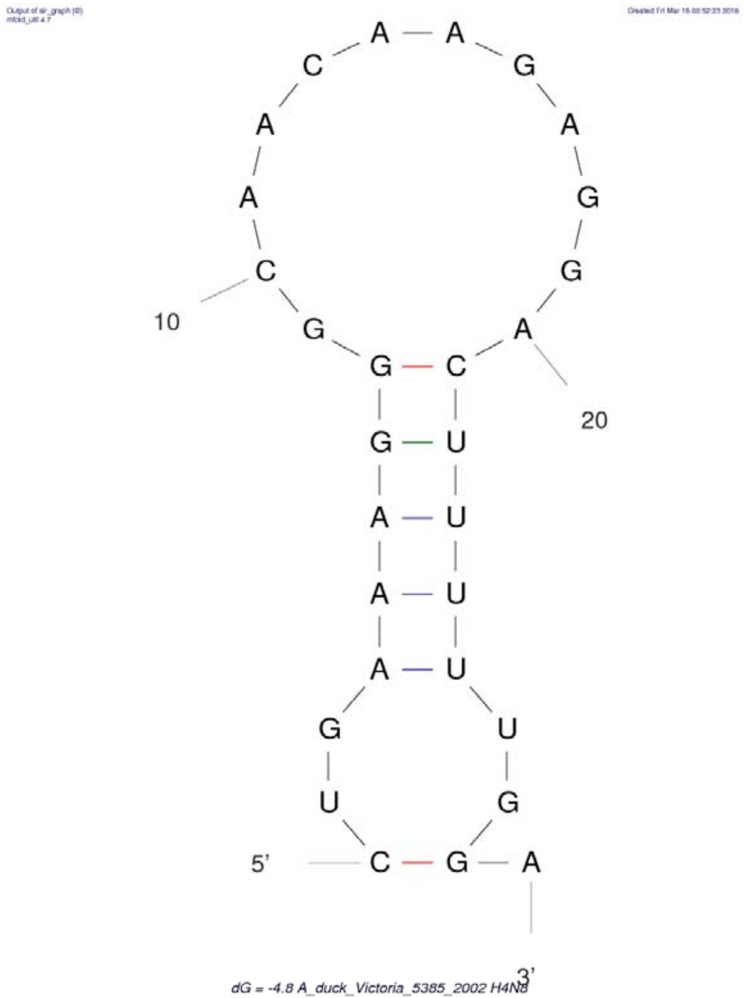

dG = -4.8 A_duck_Victoria_5385_2002 H4N8

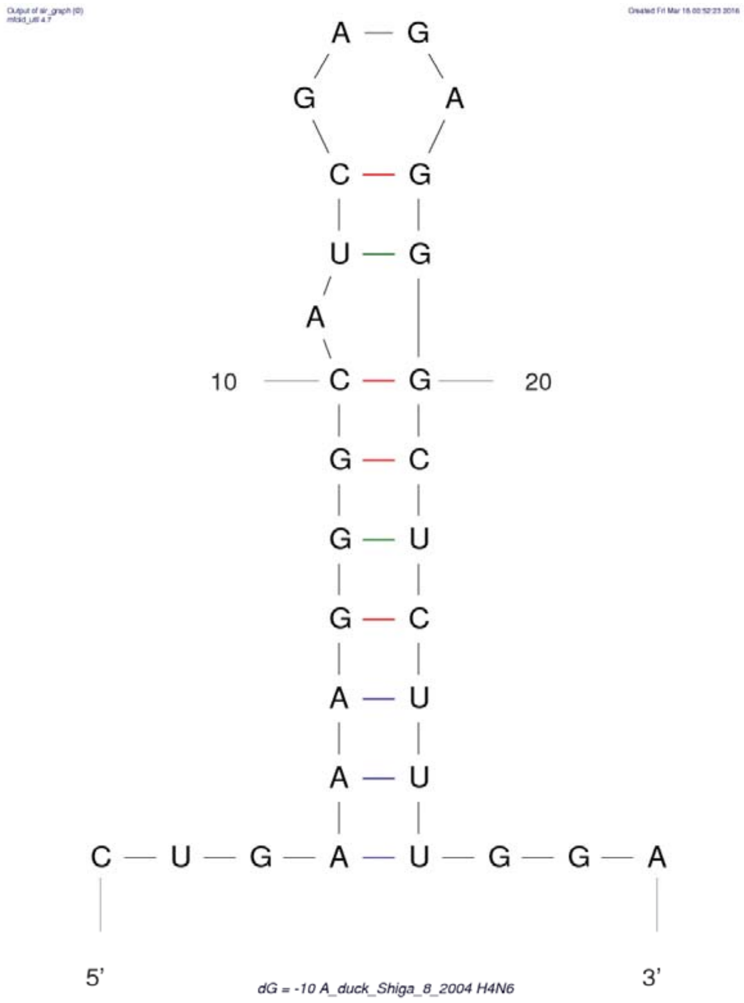

dG = -10 A_duck_Shiga_8_2004 H4N6

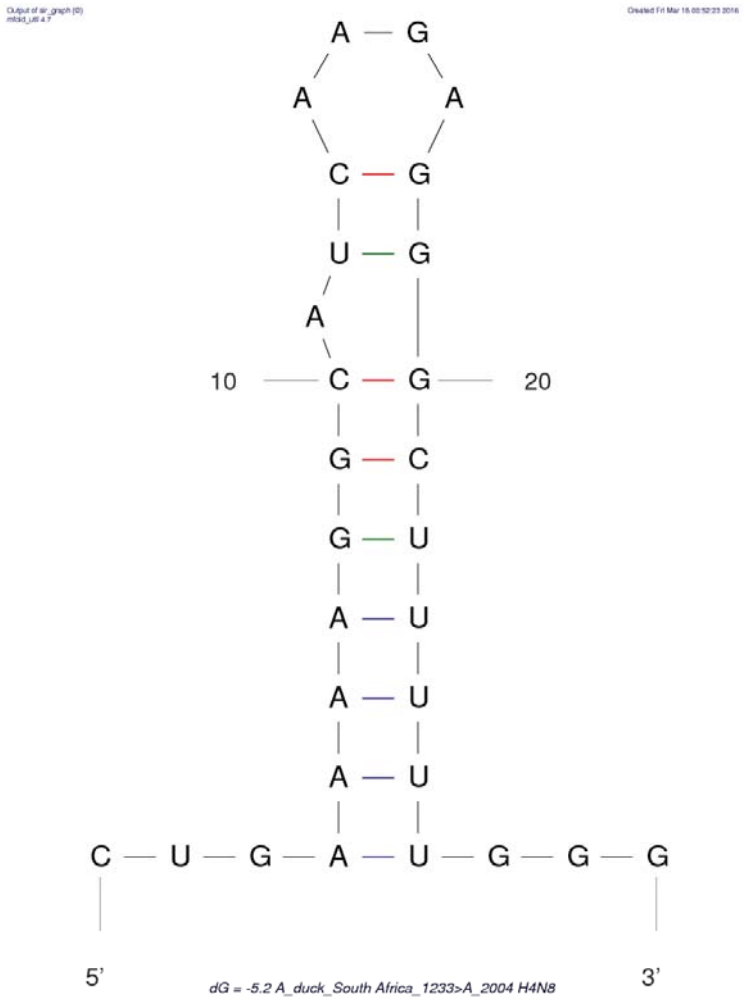

dG = -5.2 A_duck_South Africa_1233>A_2004 H4N8

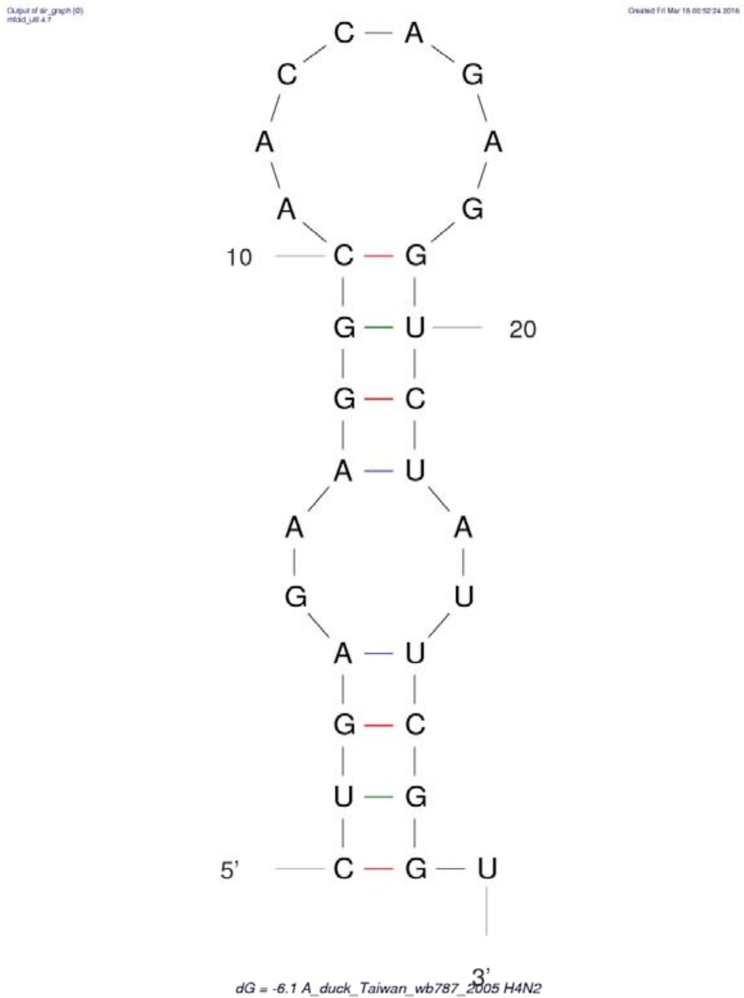

dG = -6.1 A_duck_Taiwan_wb787_2005 H4N2

Output of sir_graph (©)
mfold_util 4.7

Created Fri Mar 18 00:53:35 2016

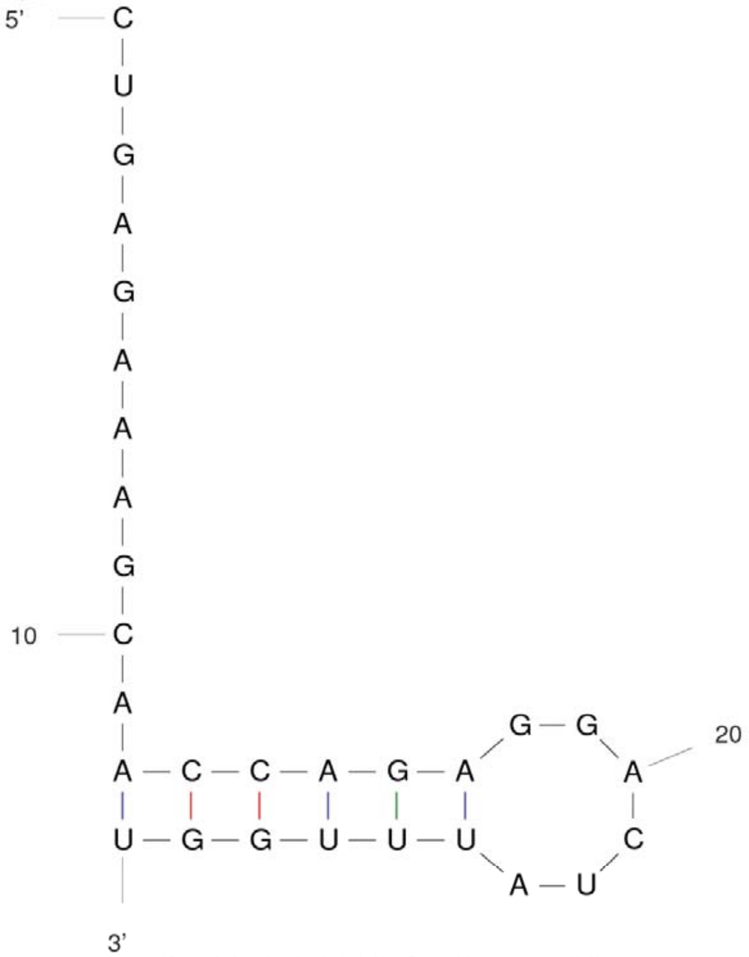

dG = -5 A_American black duck_New Brunswick_19347_2006 H4N6

Output of sir_graph (©)
mfold_util 4.7

Created Fri Mar 18 00:53:35 2016

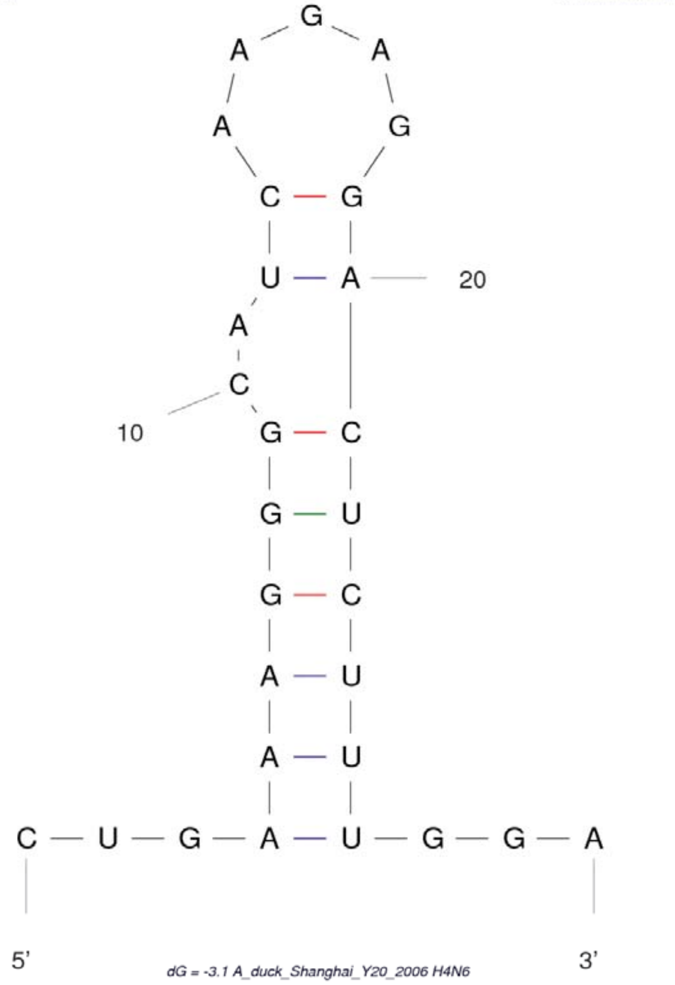

dG = -3.1 A_duck_Shanghai_Y20_2006 H4N6

Output of sir_graph (©)
mfold_util 4.7

Created Fri Mar 18 00:53:35 2016

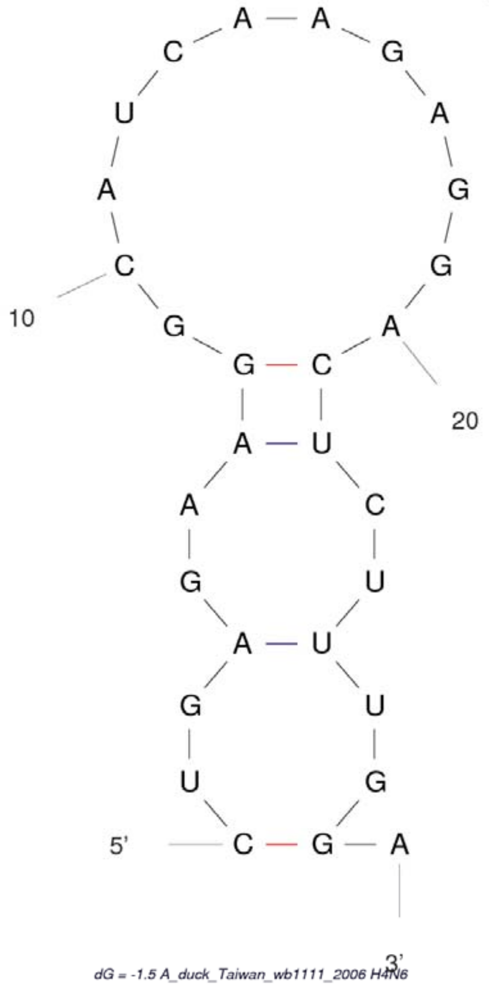

dG = -1.5 A_duck_Taiwan_wb1111_2006 H4N6

Output of sir_graph (©)
mfold_util 4.7

Created Fri Mar 18 00:53:35 2016

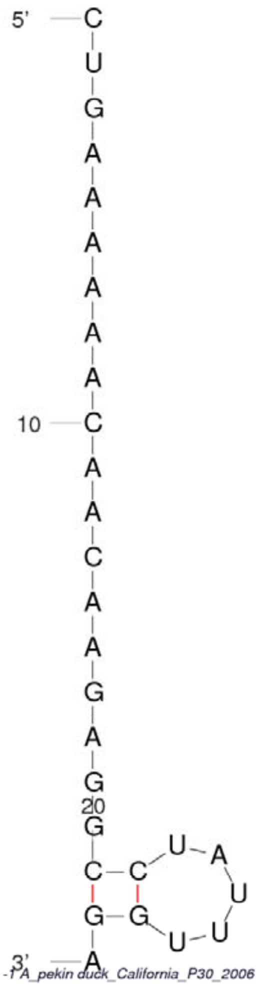

dG = -1 A_pekin duck_California_P30_2006 H4N2

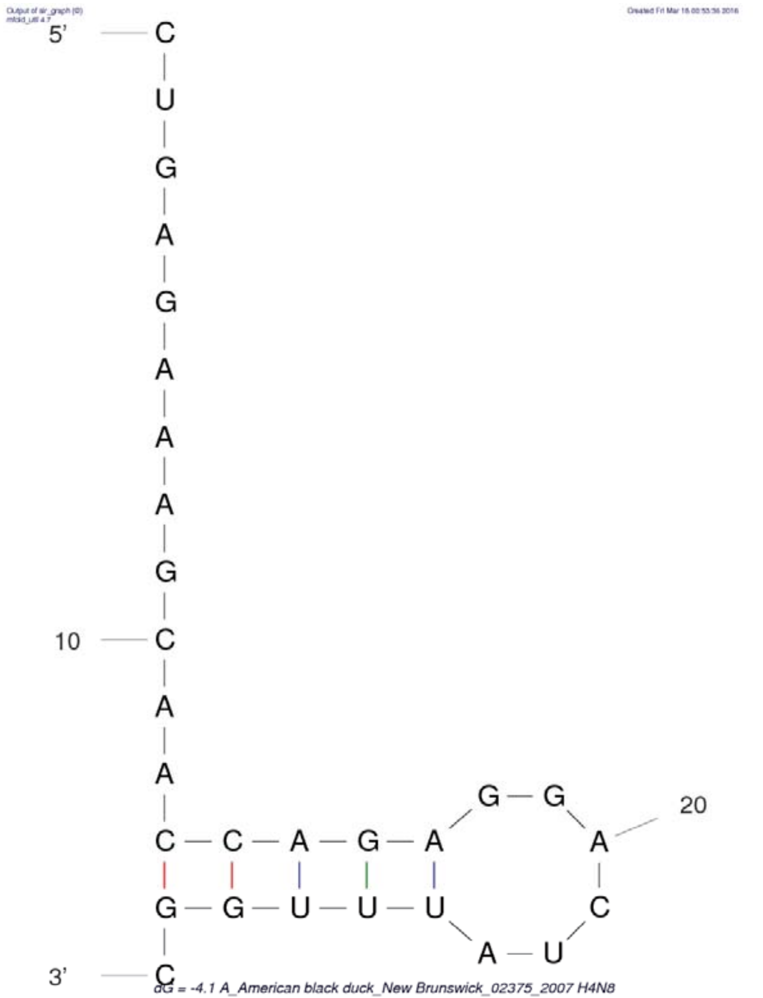

dG = -4.1 A_American black duck_New Brunswick_02375_2007 H4N8

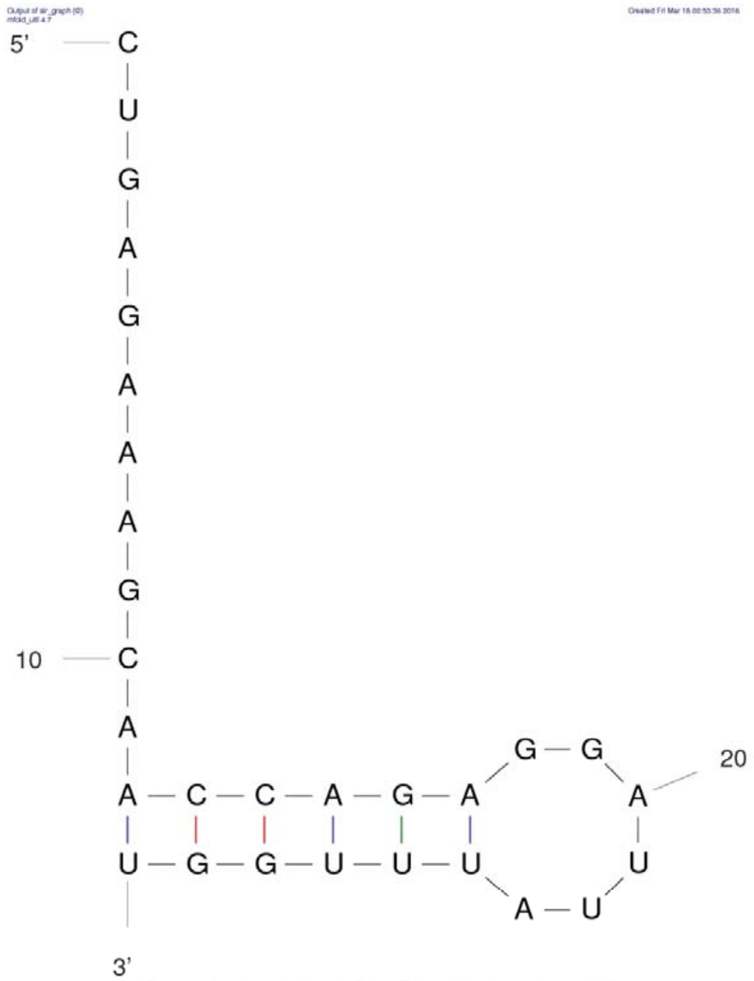

dG = -5 A_American black duck_Prince Edward Island_02661_2007 H4N6

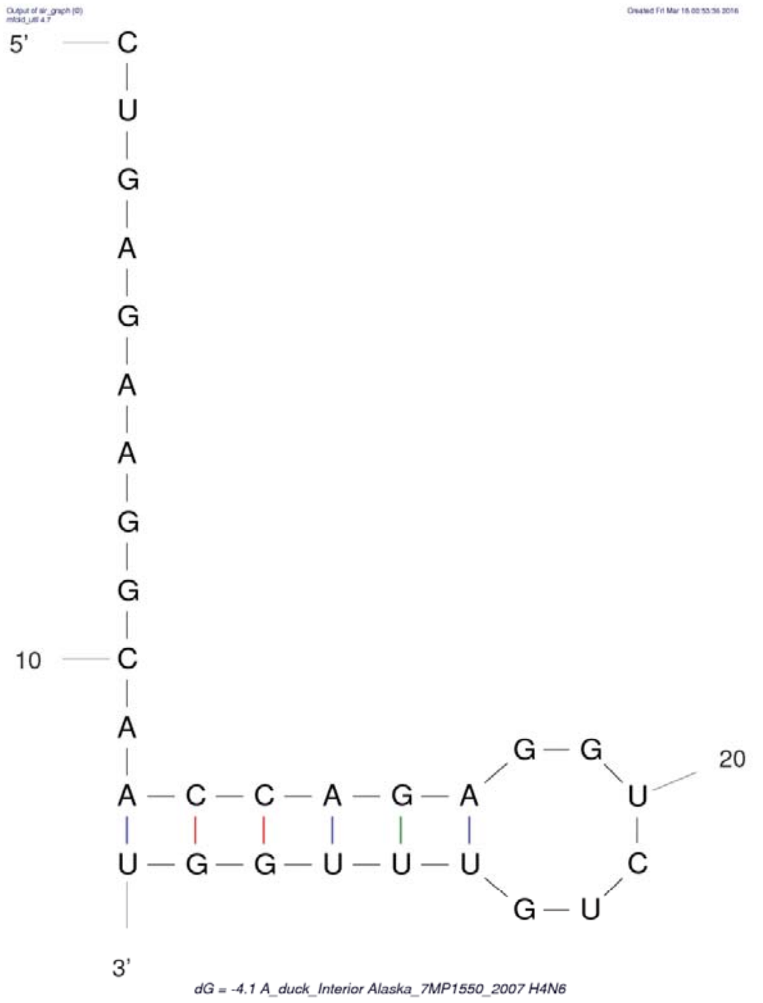

dG = -4.1 A_duck_Interior Alaska_7MP1550_2007 H4N6

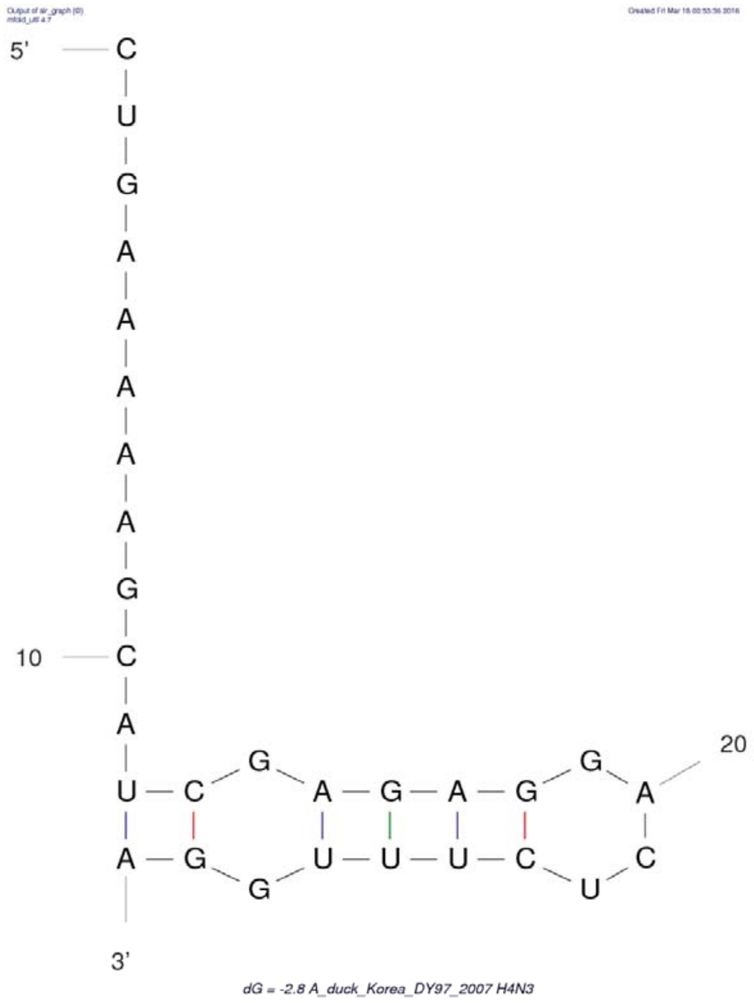

dG = -2.8 A_duck_Korea_DY97_2007 H4N3

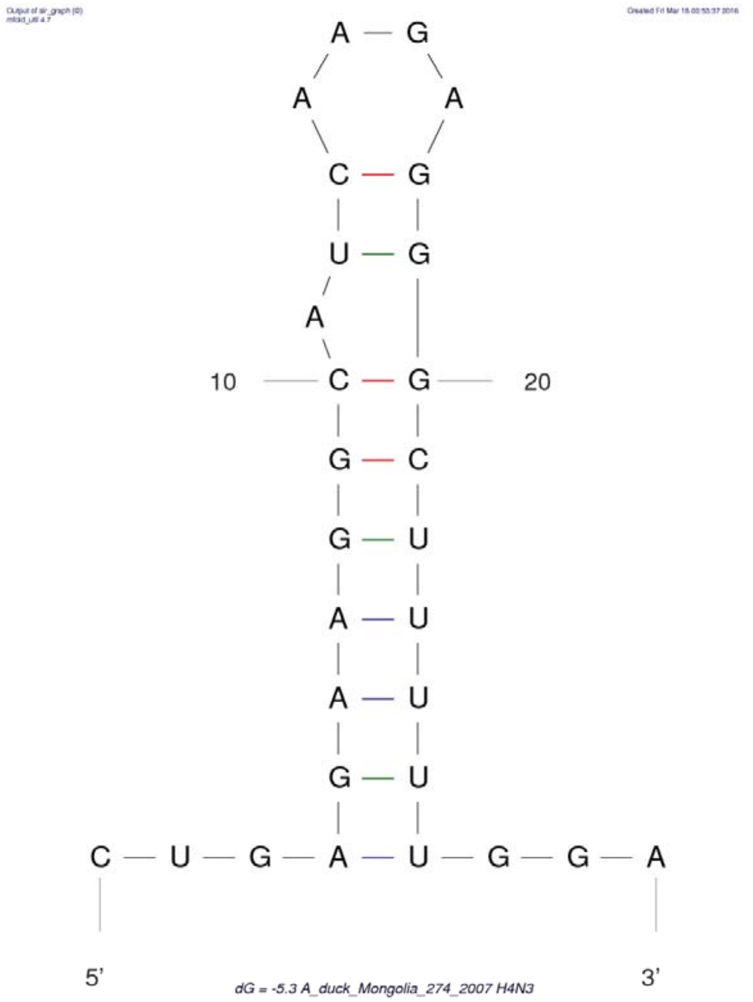

dG = -5.3 A_duck_Mongolia_274_2007 H4N3

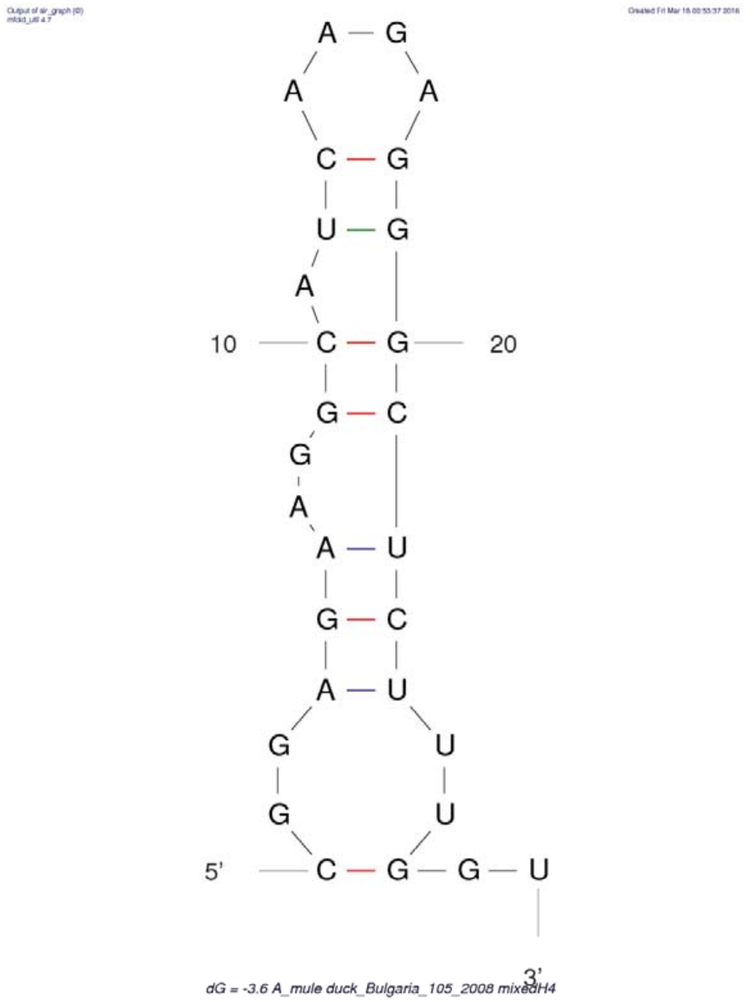

dG = -3.6 A_mule duck_Bulgaria_105_2008 mixedH4

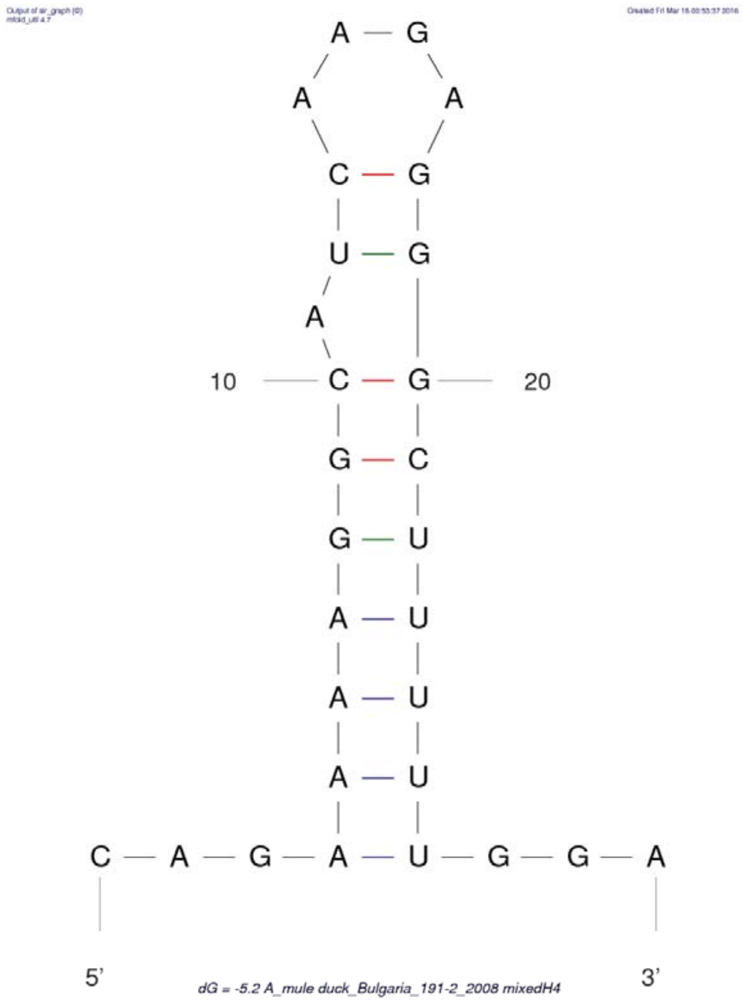

dG = -5.2 A_mule duck_Bulgaria_191-2_2008 mixedH4

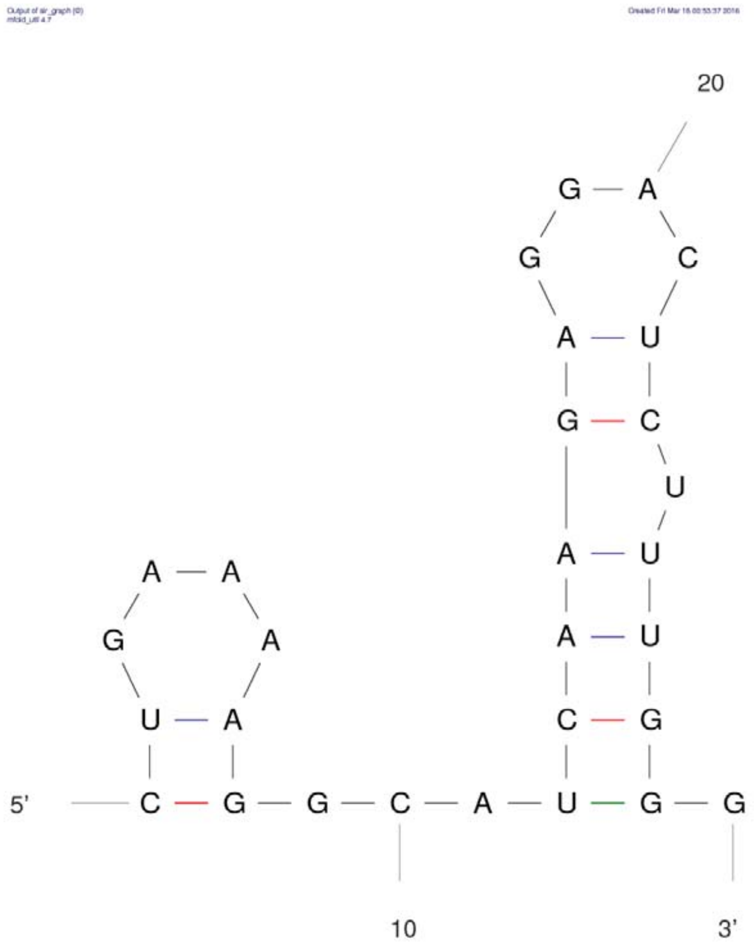

dG = -1.8 A_wild duck_Korea_SH5-60_2008 H4N6

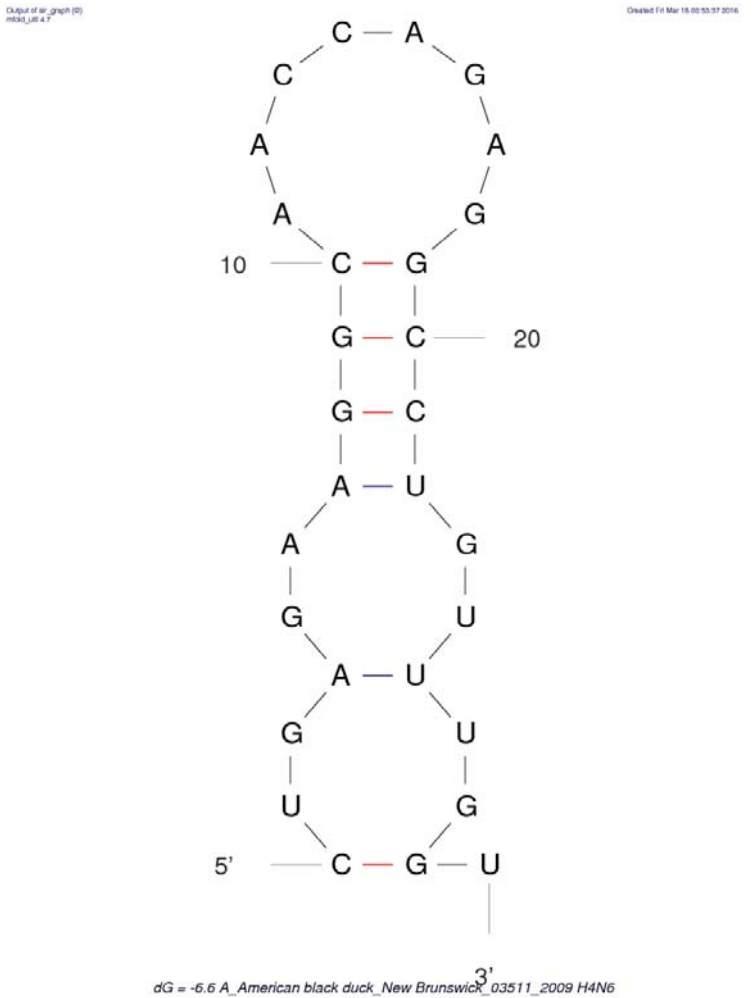

dG = -6.6 A_American black duck_New Brunswick_03511_2009 H4N6

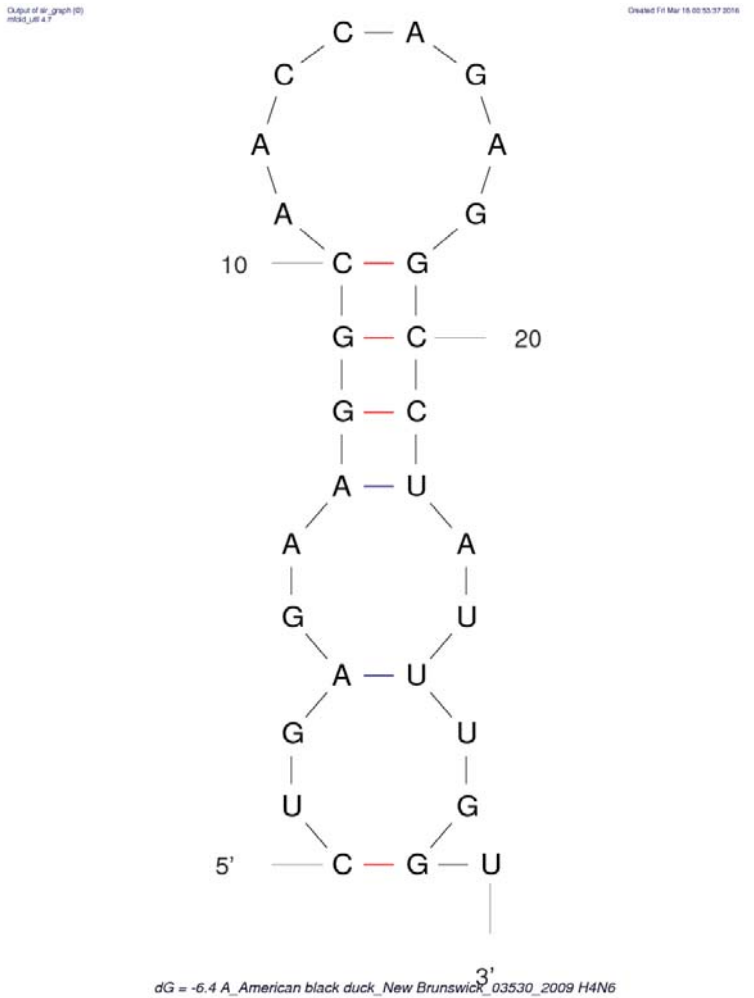

dG = -6.4 A_American black duck_New Brunswick_03530_2009 H4N6

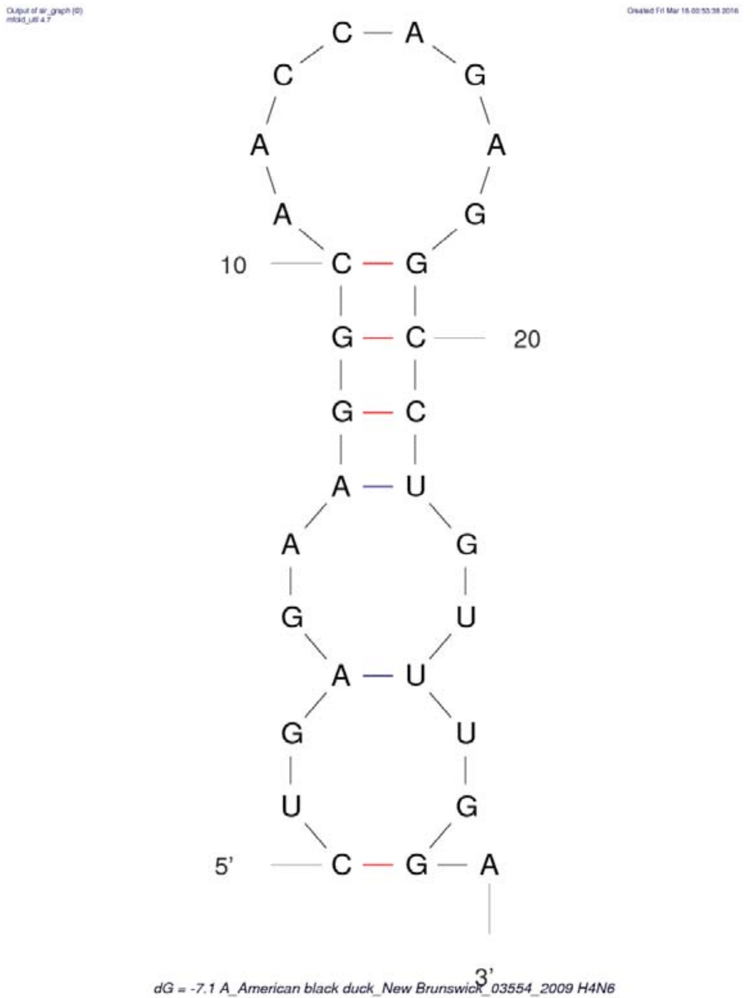

dG = -7.1 A_American black duck_New Brunswick_03554_2009 H4N6

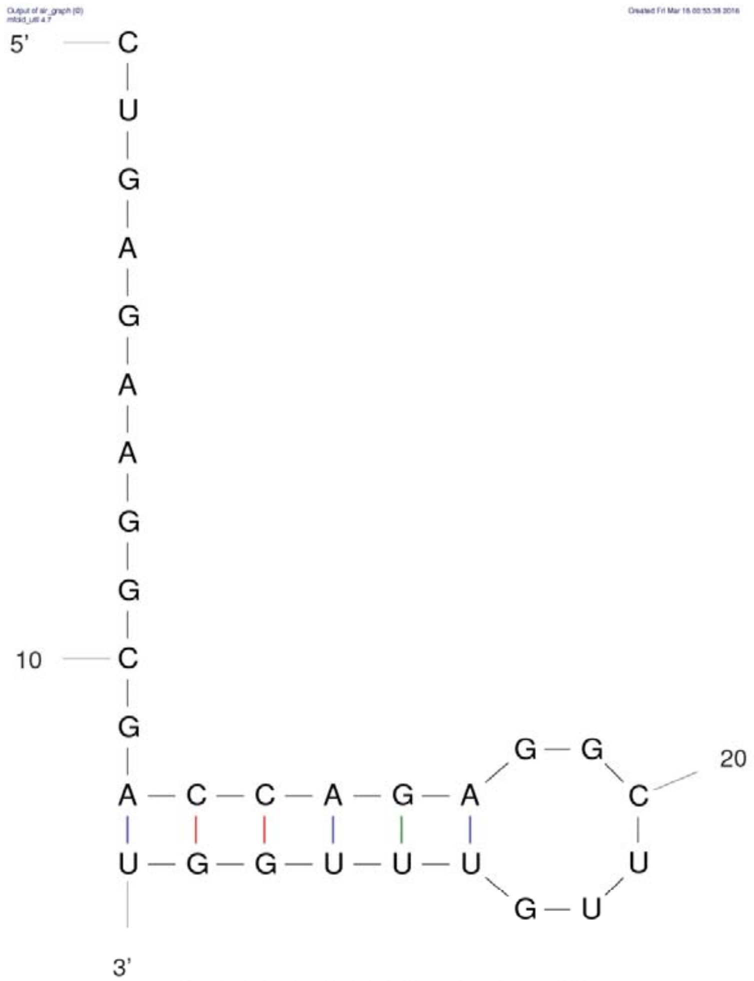

dG = -4.2 A_American black duck_Wisconsin_2542_2009 H4N2

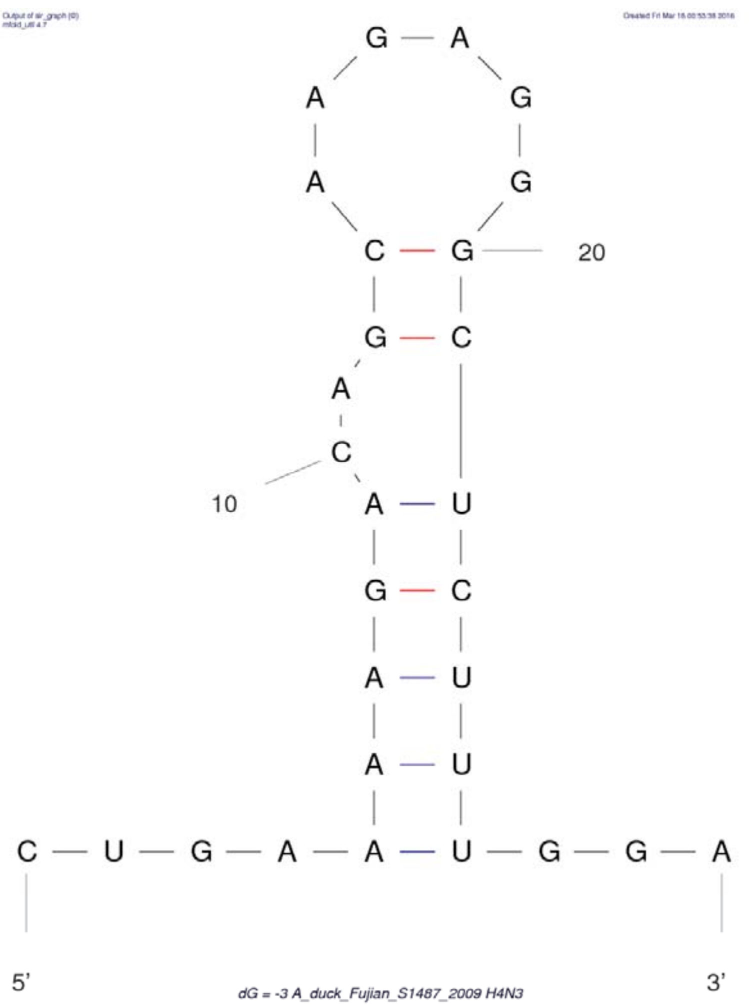

dG = -3 A_duck_Fujian_S1487_2009 H4N3

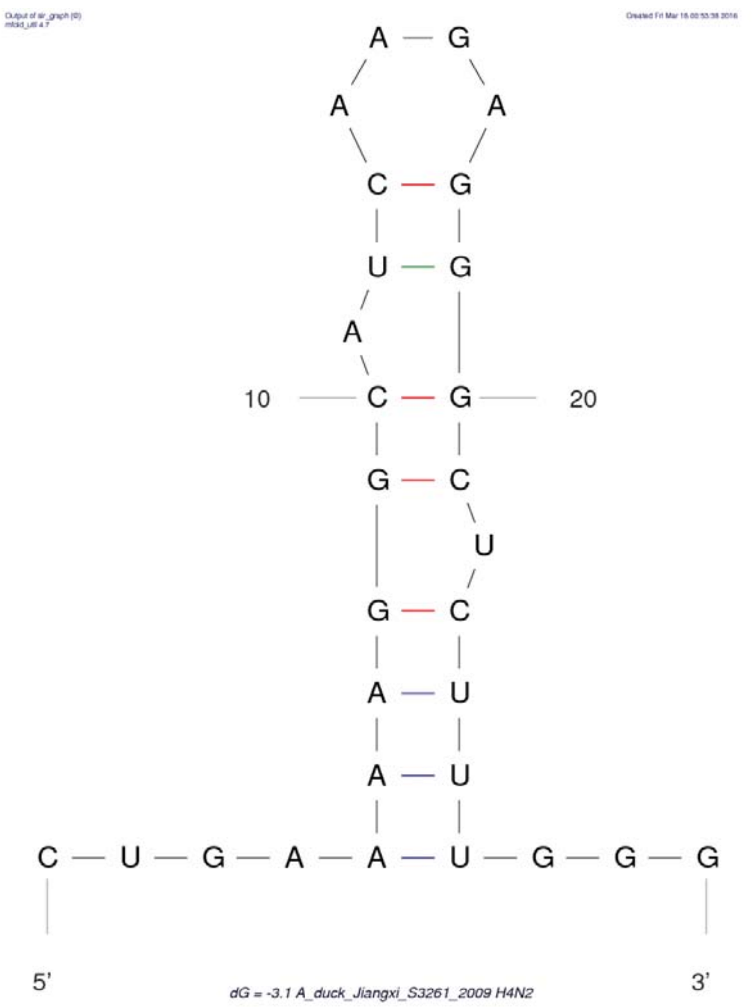

dG = -3.1 A_duck_Jiangxi_S3261_2009 H4N2

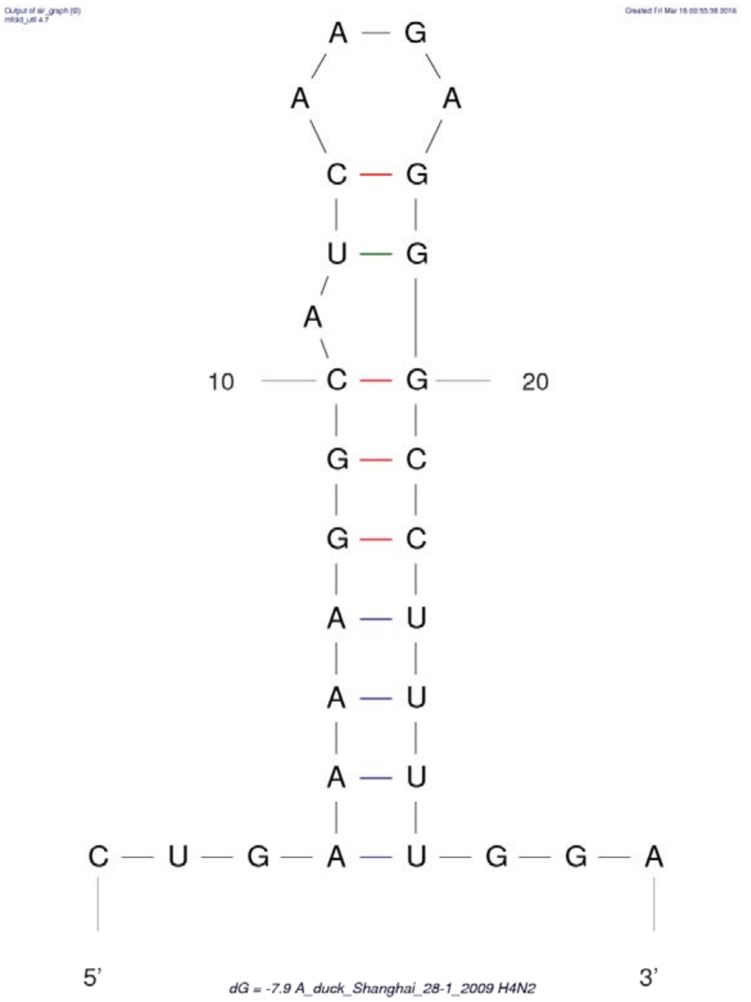

dG = -7.9 A_duck_Shanghai_28-1_2009 H4N2

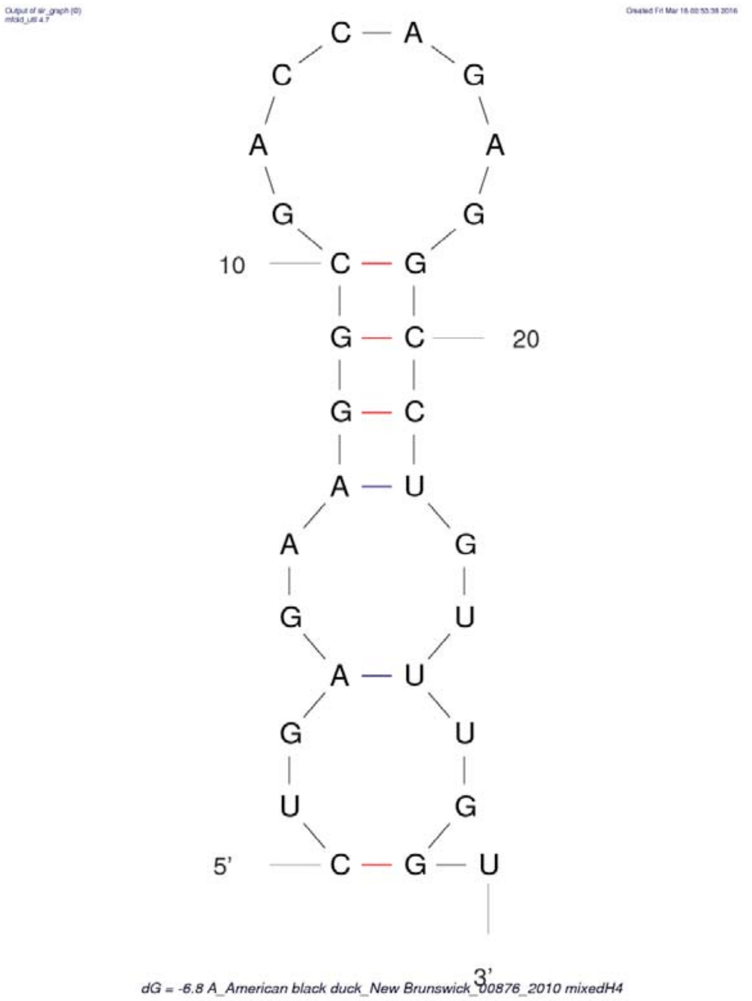

dG = -6.8 A_American black duck_New Brunswick_00876_2010 mixedH4

Output of sir_graph (©)
mfold_util 4.7

Created Fri Mar 18 00:53:38 2016

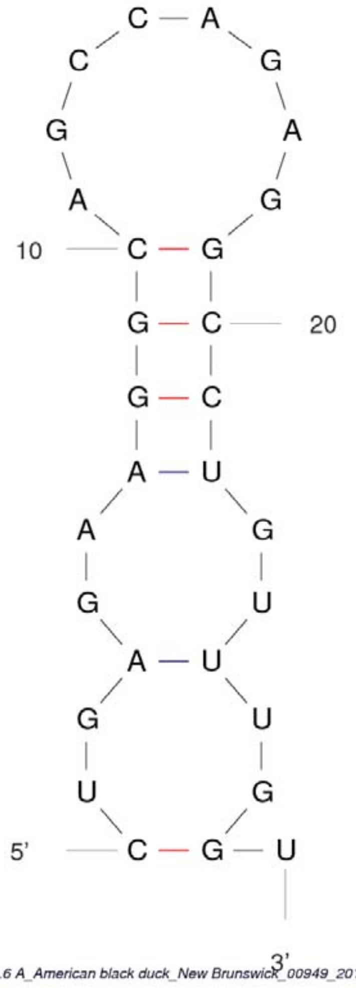

dG = -6.6 A_American black duck_New Brunswick_00949_2010 H4N6

Output of sir_graph (©)
mfold_util 4.7

Created Fri Mar 18 00:53:39 2016

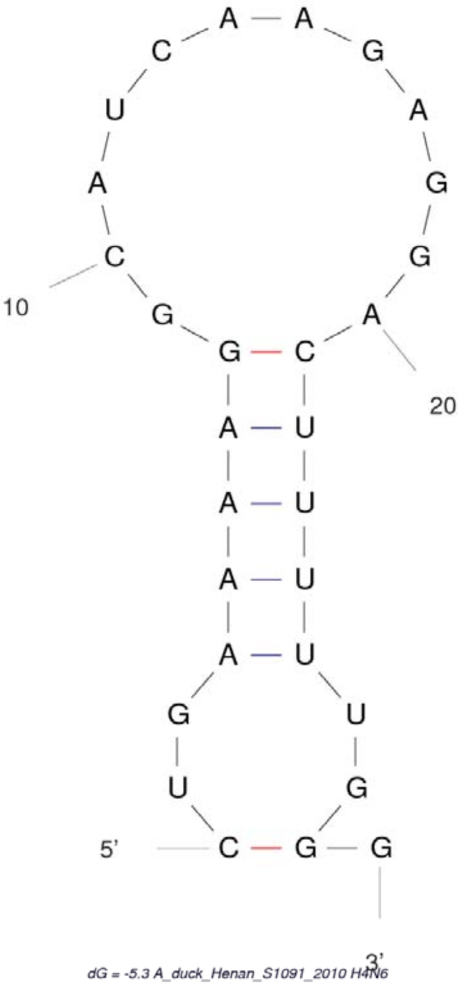

dG = -5.3 A_duck_Henan_S1091_2010 H4N6

Output of sir_graph (©)
mfold_util 4.7

Created Fri Mar 18 00:53:39 2016

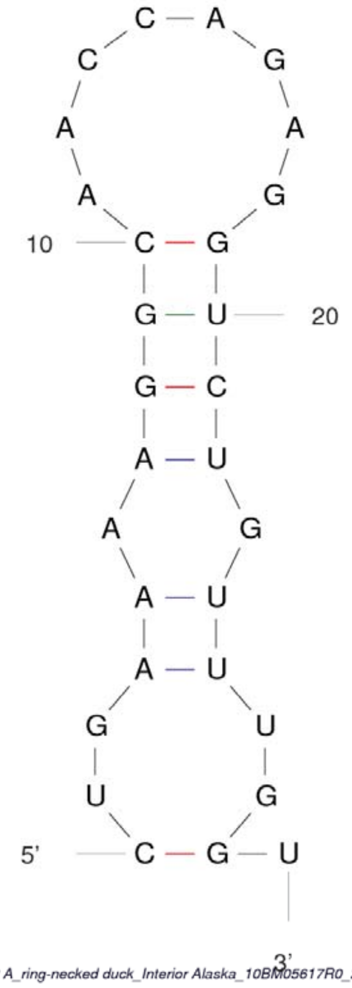

dG = -5.2 A_ring-necked duck_Interior Alaska_10BM05617R0_2010 H4N6

Output of sir_graph (©)
mfold_util 4.7

Created Fri Mar 18 00:53:39 2016

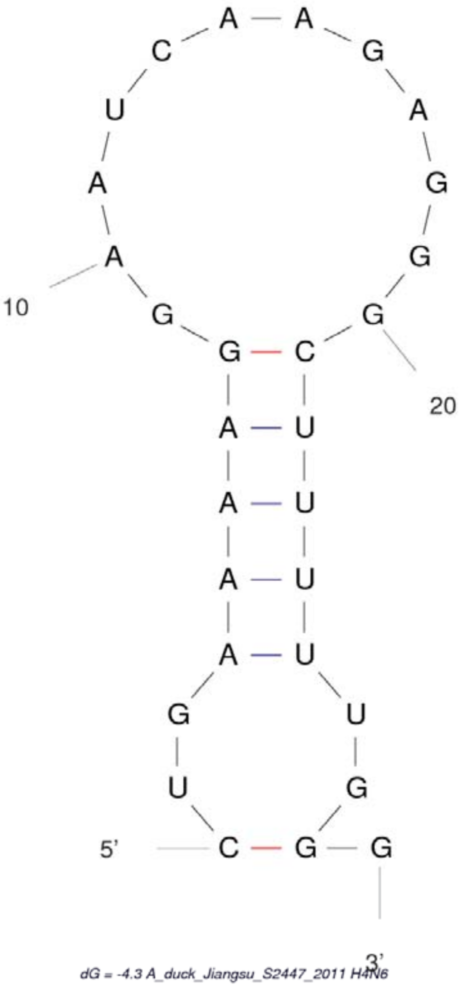

dG = -4.3 A_duck_Jiangsu_S2447_2011 H4N6

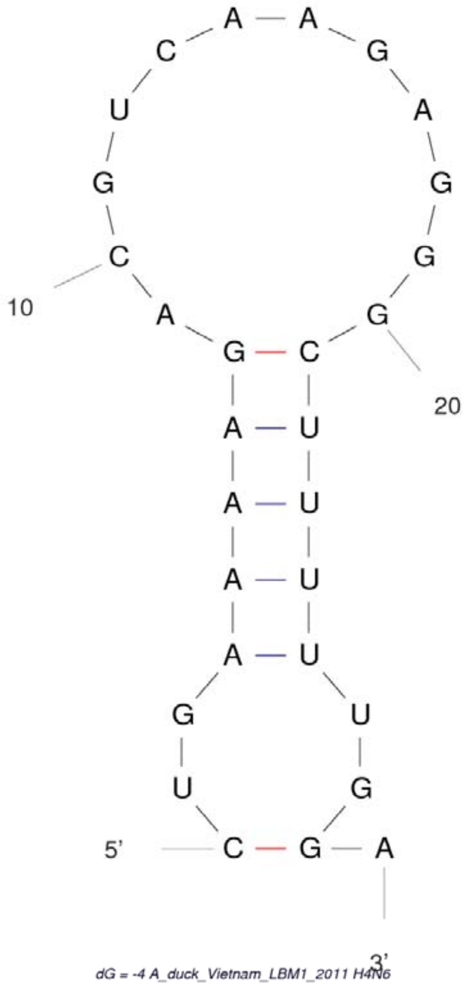

dG = -4 A_duck_Vietnam_LBM1_2011 H4N6

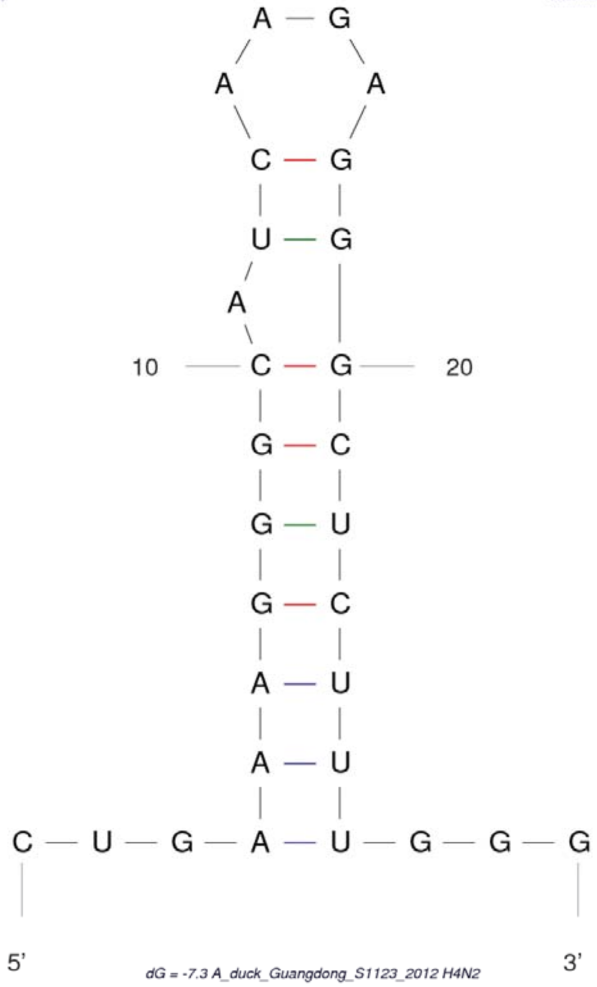

dG = -7.3 A_duck_Guangdong_S1123_2012 H4N2

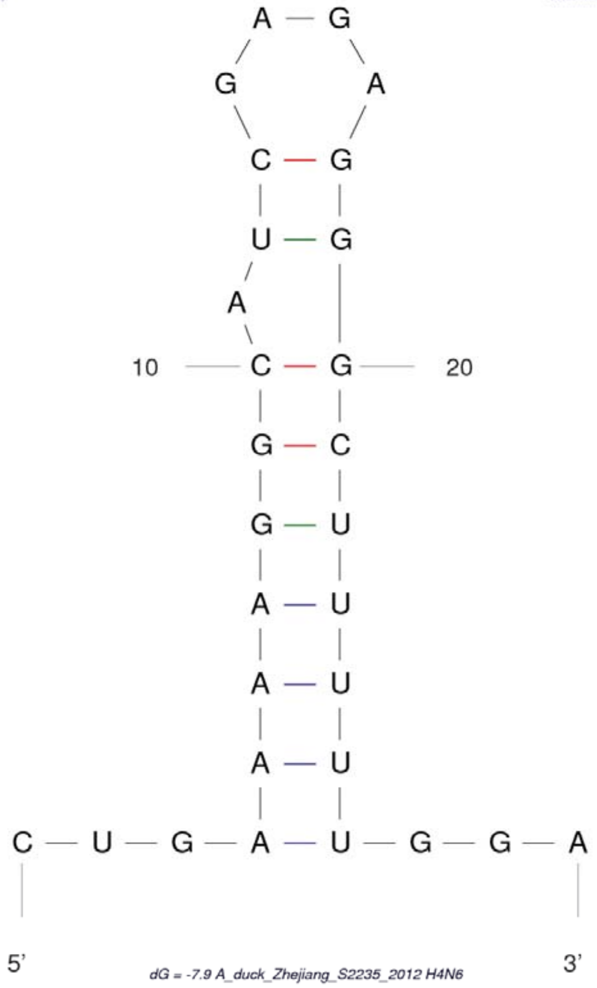

dG = -7.9 A_duck_Zhejiang_S2235_2012 H4N6

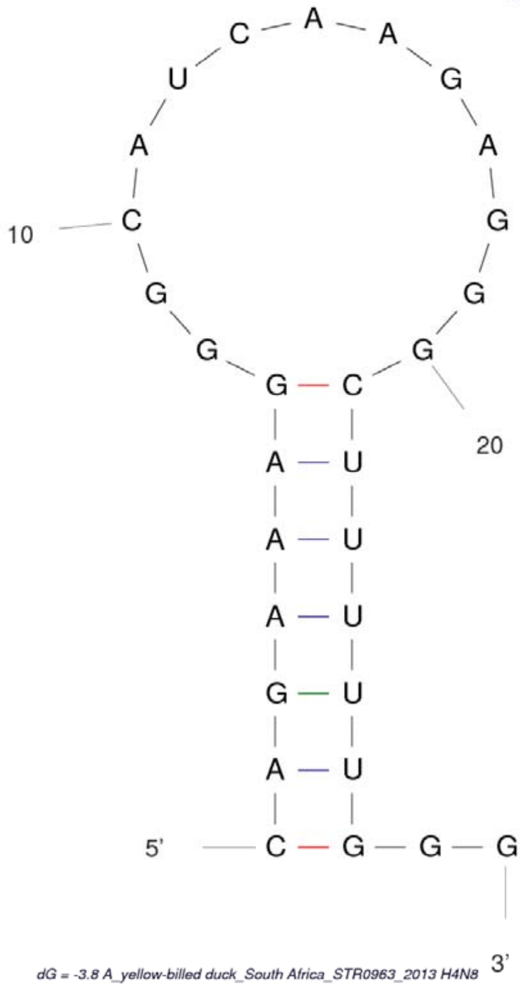

dG = -3.8 A_yellow-billed duck_South Africa_STR0963_2013 H4N8

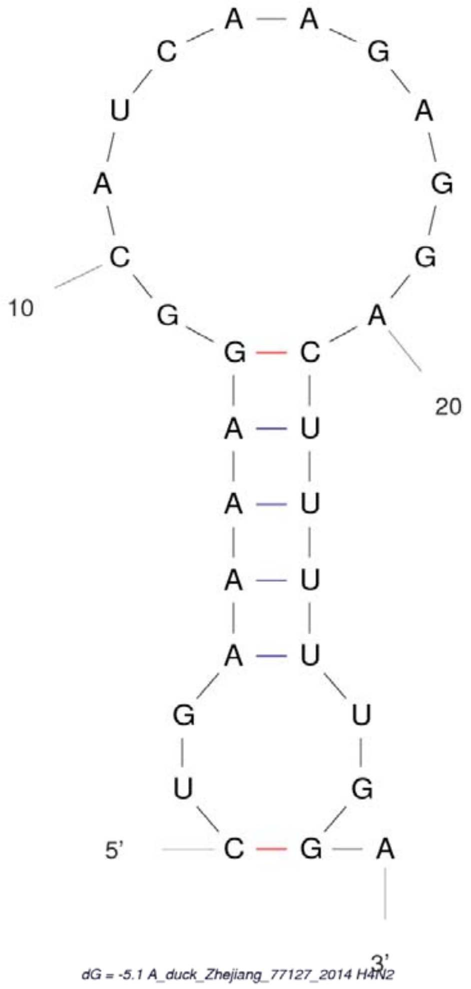

dG = -5.1 A_duck_Zhejiang_77127_2014 H4N2

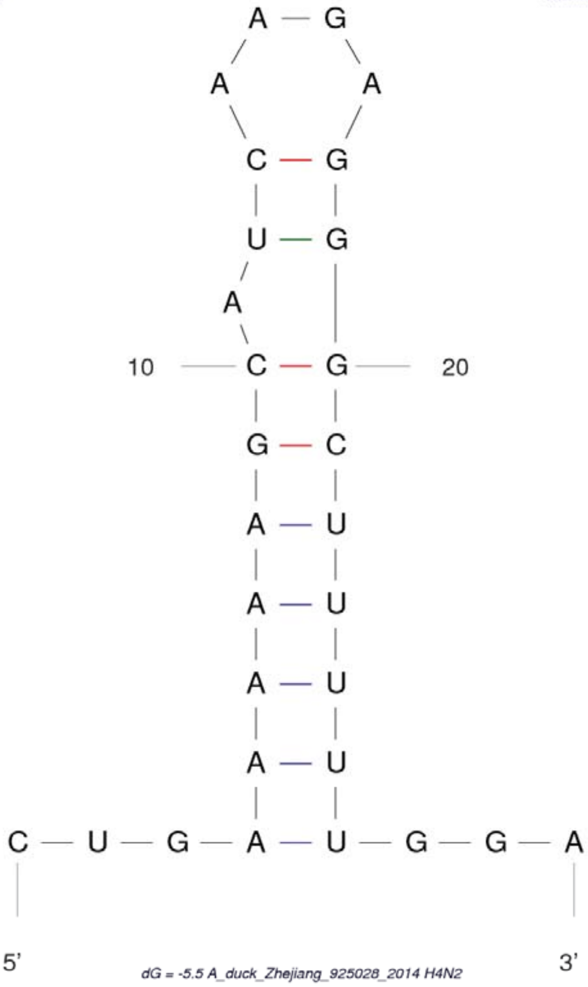

dG = -5.5 A_duck_Zhejiang_925028_2014 H4N2

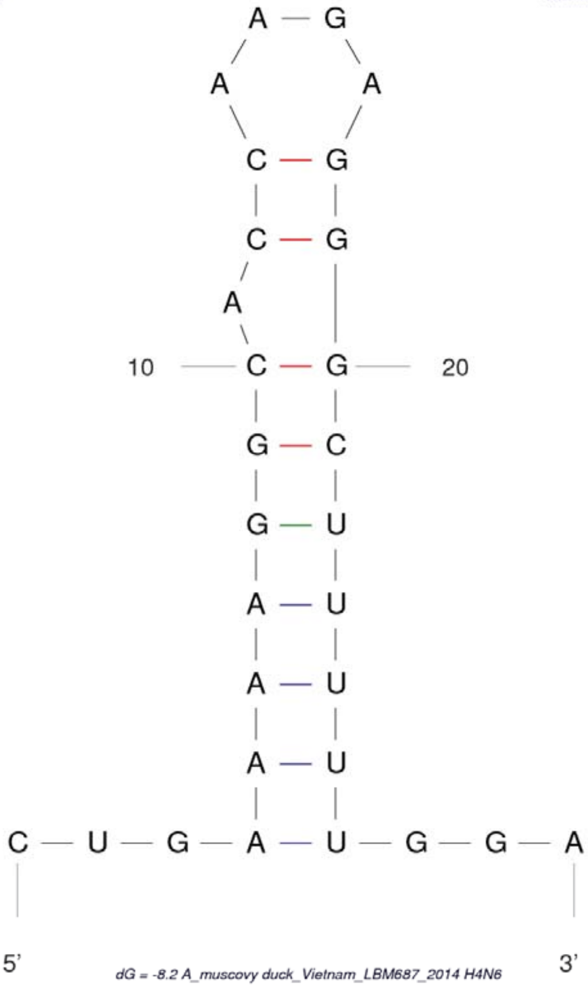

dG = -8.2 A_muscovy duck_Vietnam_LBM687_2014 H4N6

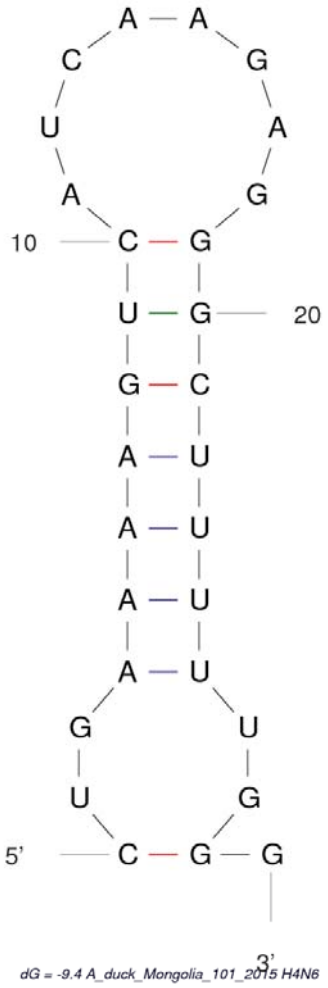

dG = -9.4 A_duck_Mongolia_101_2015 H4N6

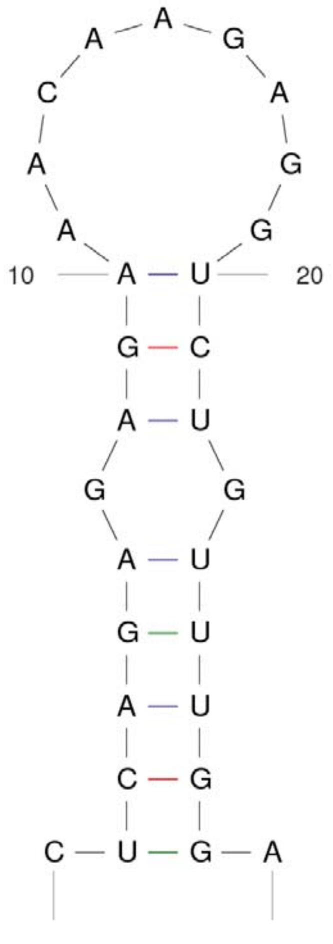

dG = -4.8 A_duck_Minnesota_63_1976 H5N2

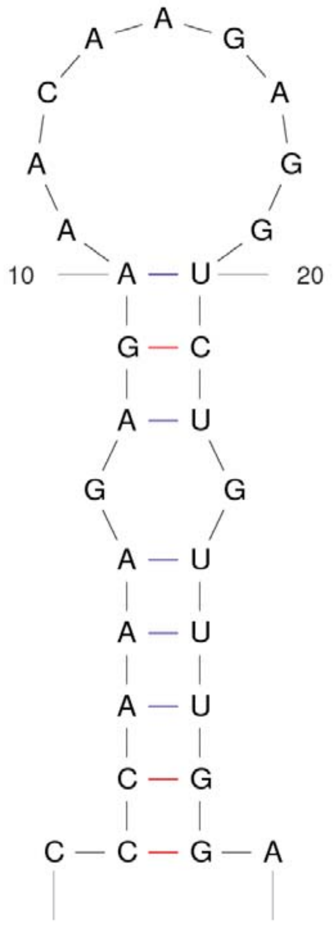

dG = -7.5 A_mallard duck_ALB_57_1976 H5N2

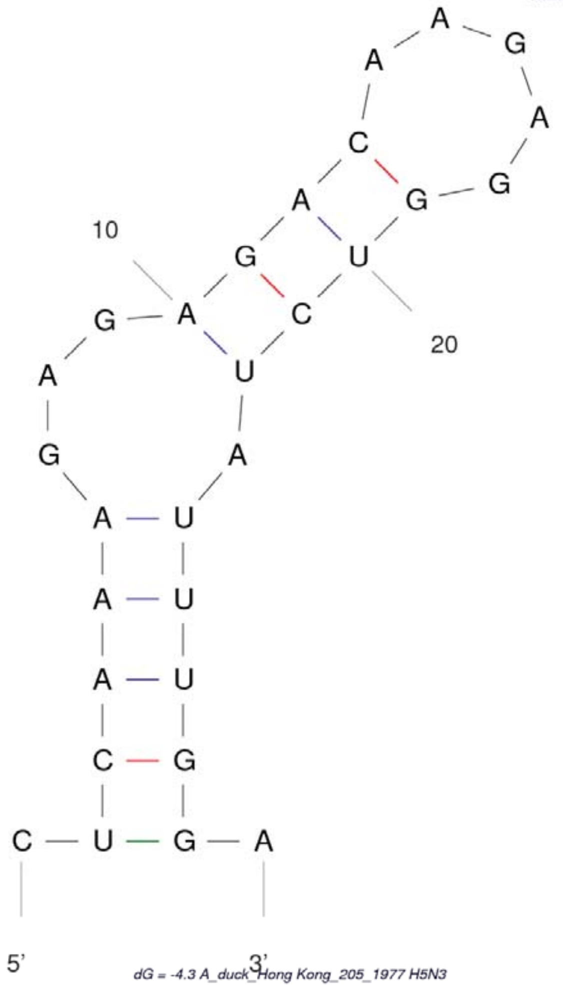

dG = -4.3 A_duck_Hong Kong_205_1977 H5N3

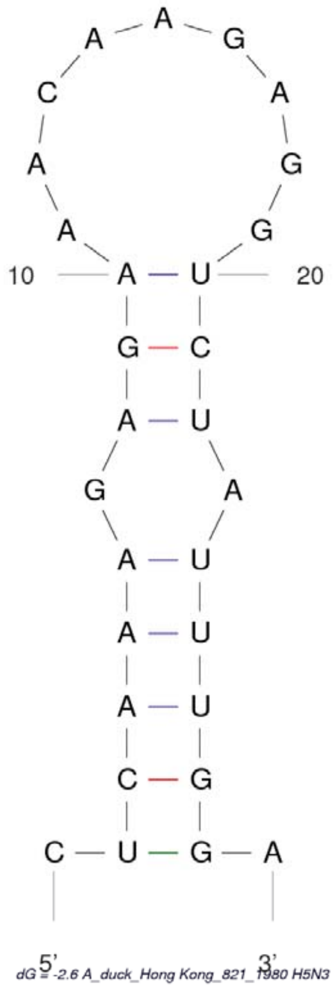

dG = -2.6 A_duck_Hong Kong_821_1980 H5N3

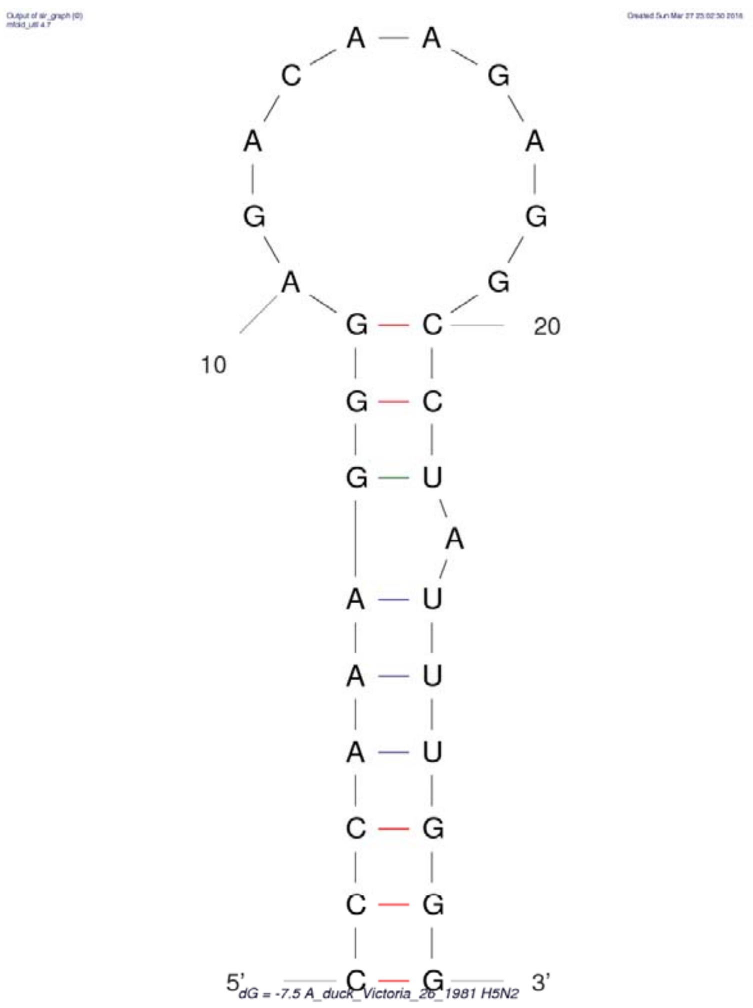
Output of sir_graph (©)
mfold_util 4.7
Created Sun Mar 27 23:02:30 2016
dG = -7.5 A_duck_Victoria_26_1981 H5N2

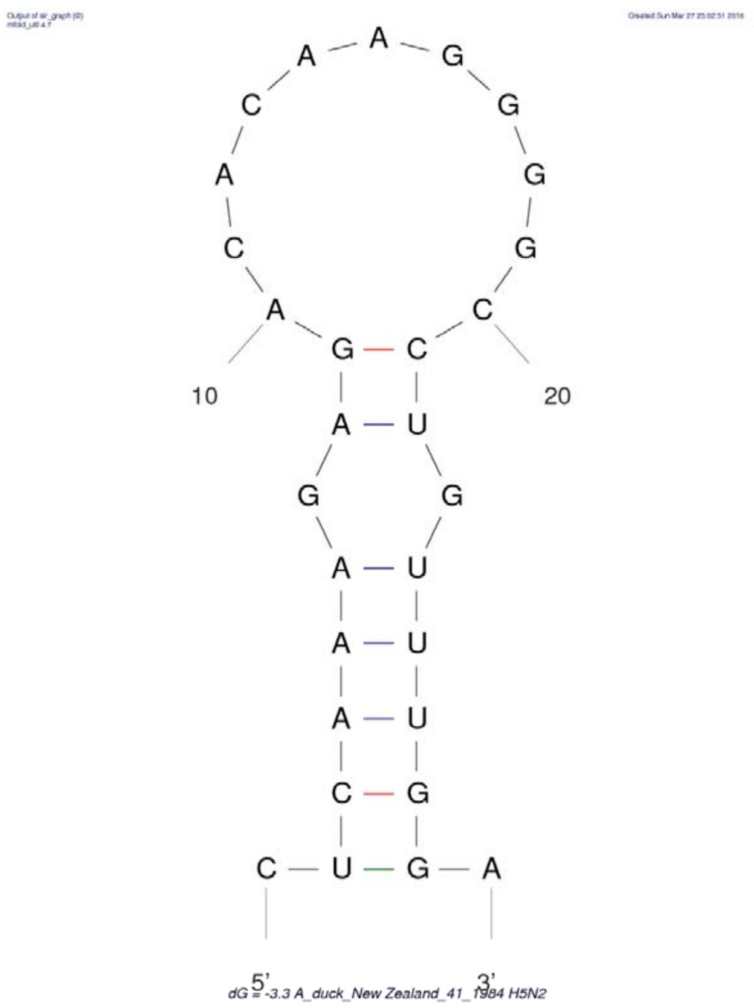
Output of sir_graph (©)
mfold_util 4.7
Created Sun Mar 27 23:02:31 2016
dG = -3.3 A_duck_New Zealand_41_1984 H5N2

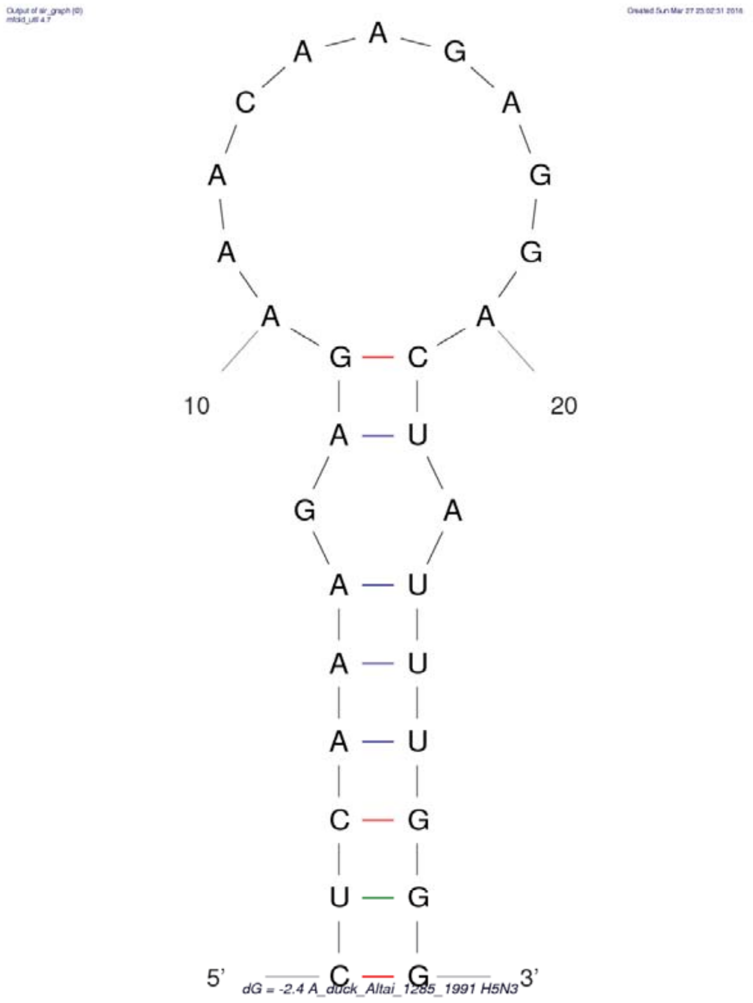
Output of sir_graph (©)
mfold_util 4.7
Created Sun Mar 27 23:02:31 2016
dG = -2.4 A_duck_Altai_1285_1991 H5N3

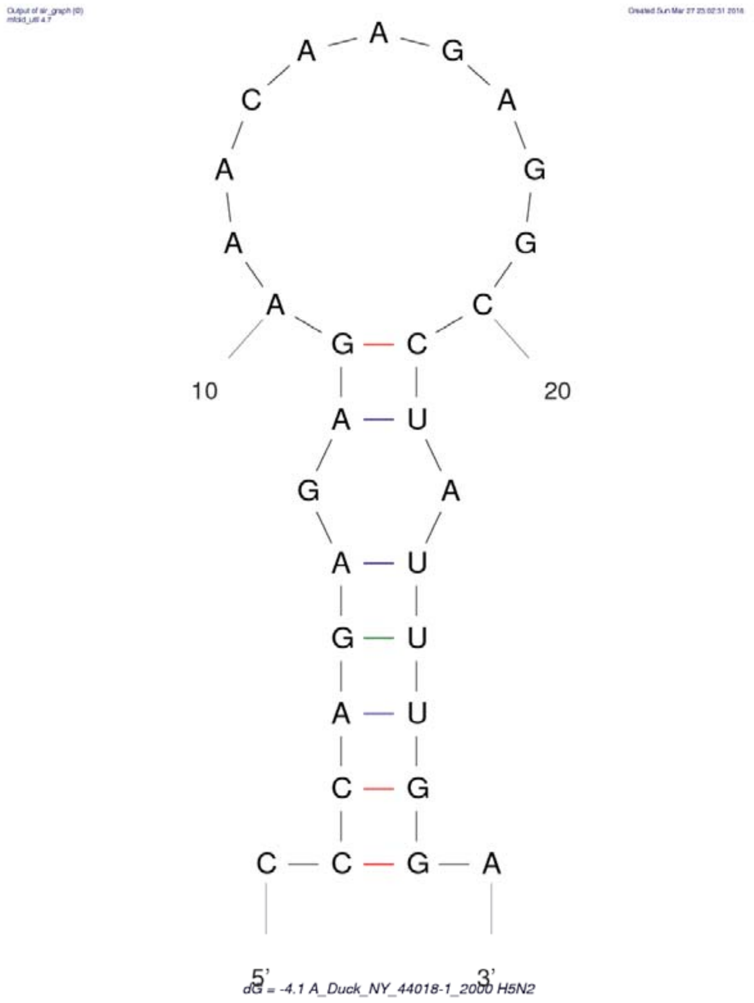
Output of sir_graph (©)
mfold_util 4.7
Created Sun Mar 27 23:02:31 2016
dG = -4.1 A_Duck_NY_44018-1_2000 H5N2

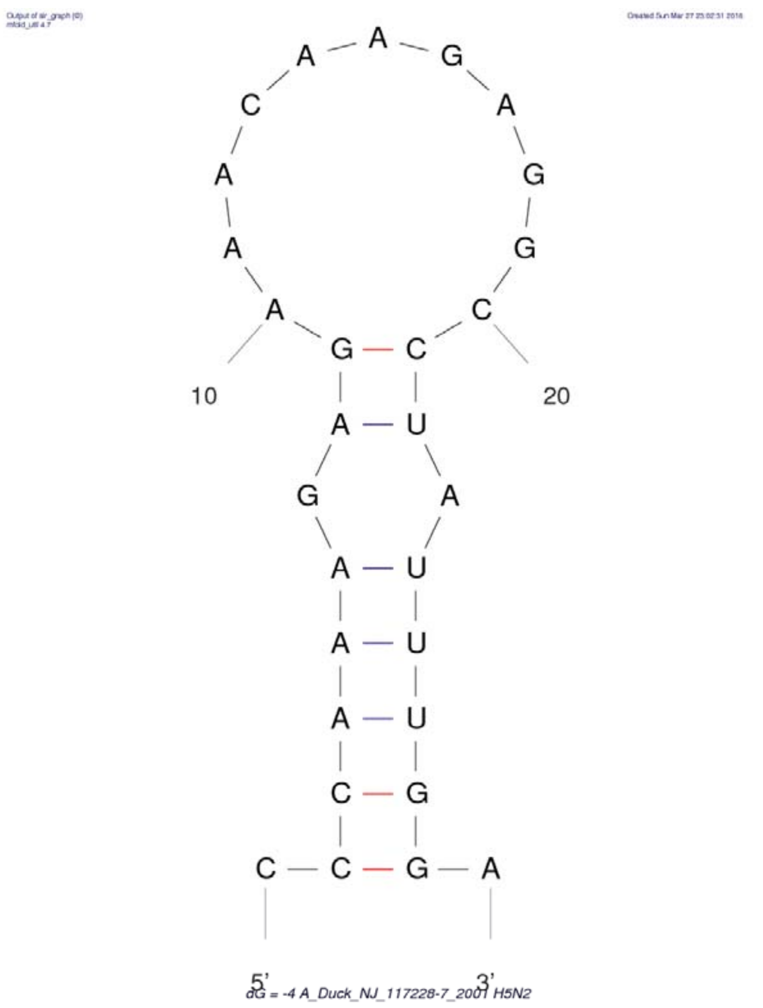

Output of sir_graph (©)
mfold_util 4.7
Created Sun Mar 27 23:02:31 2016
10
20
5'
3'
dG = -4 A_Duck_NJ_117228-7_2001 H5N2

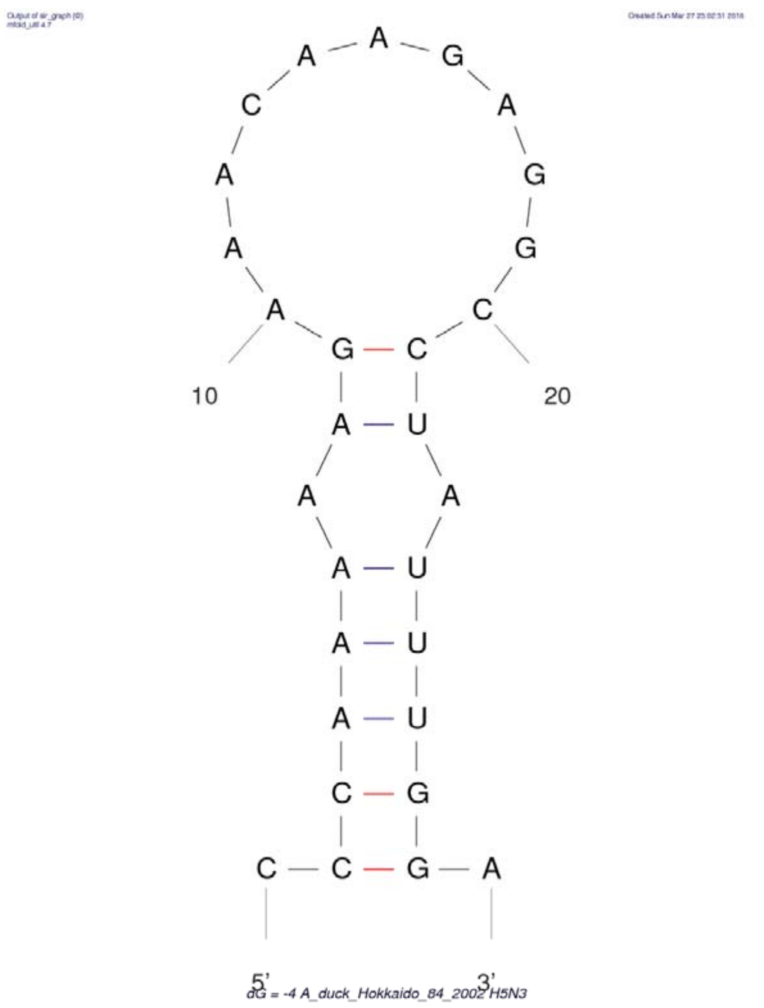

Output of sir_graph (©)
mfold_util 4.7
Created Sun Mar 27 23:02:31 2016
10
20
5'
3'
dG = -4 A_duck_Hokkaido_84_2002 H5N3

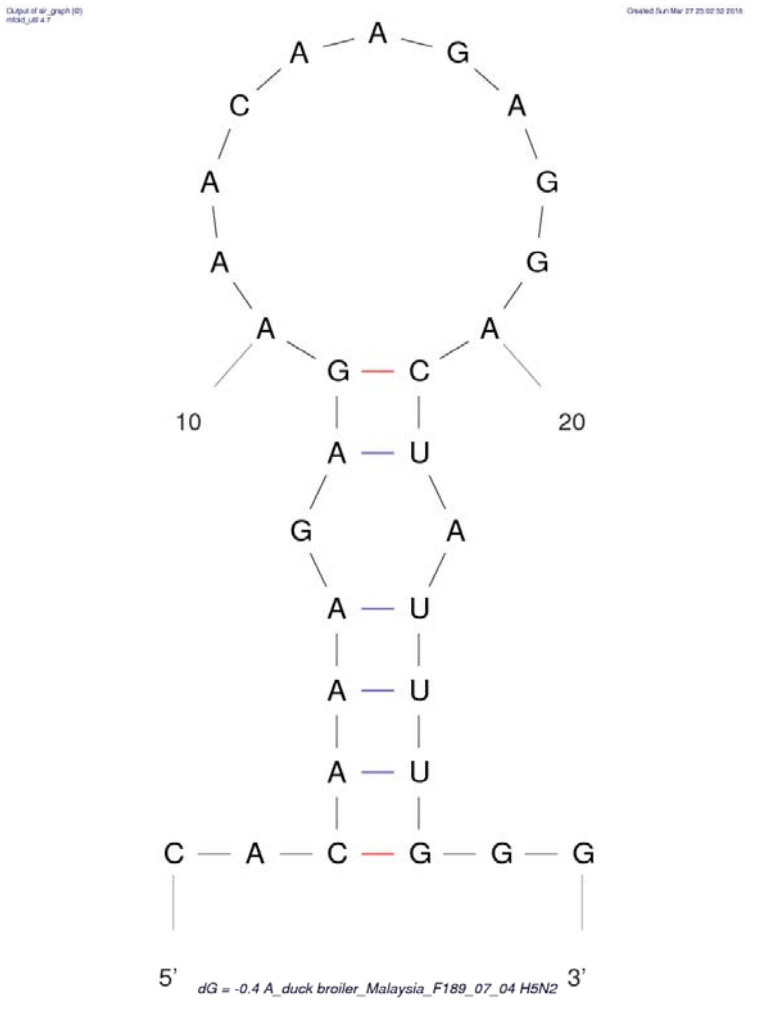

Output of sir_graph (©)
mfold_util 4.7
Created Sun Mar 27 23:02:32 2016
10
20
5'
3'
dG = -0.4 A_duck broiler_Malaysia_F189_07_04 H5N2

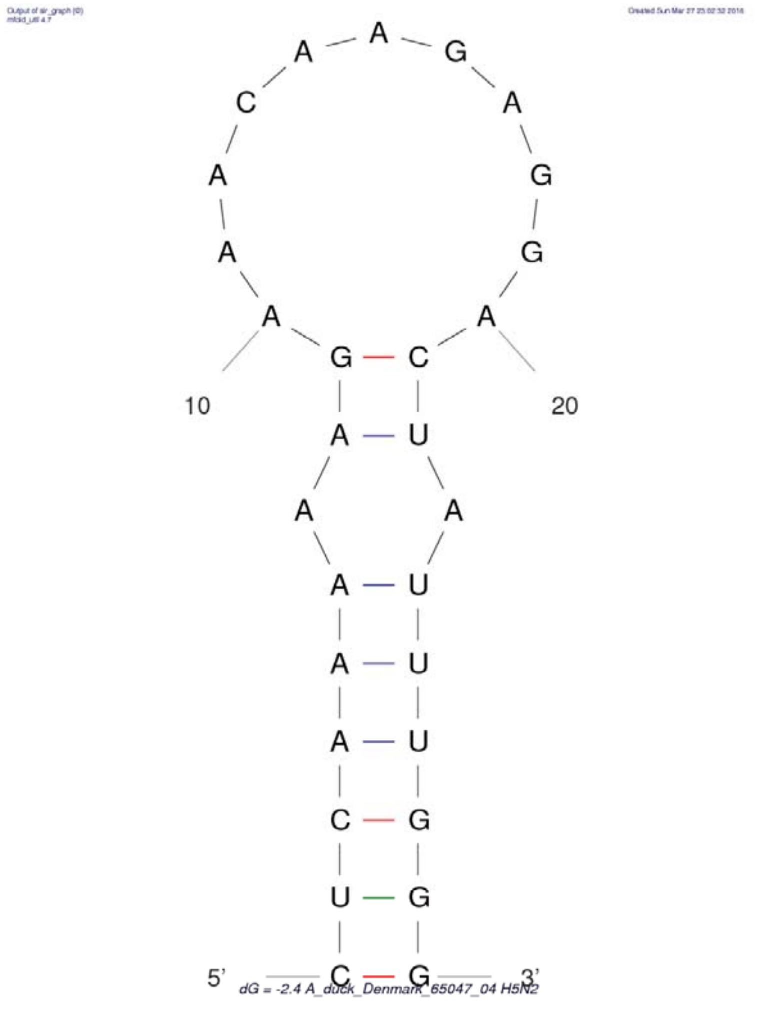

Output of sir_graph (©)
mfold_util 4.7
Created Sun Mar 27 23:02:32 2016
10
20
5'
3'
dG = -2.4 A_duck_Denmark_65047_04 H5N2

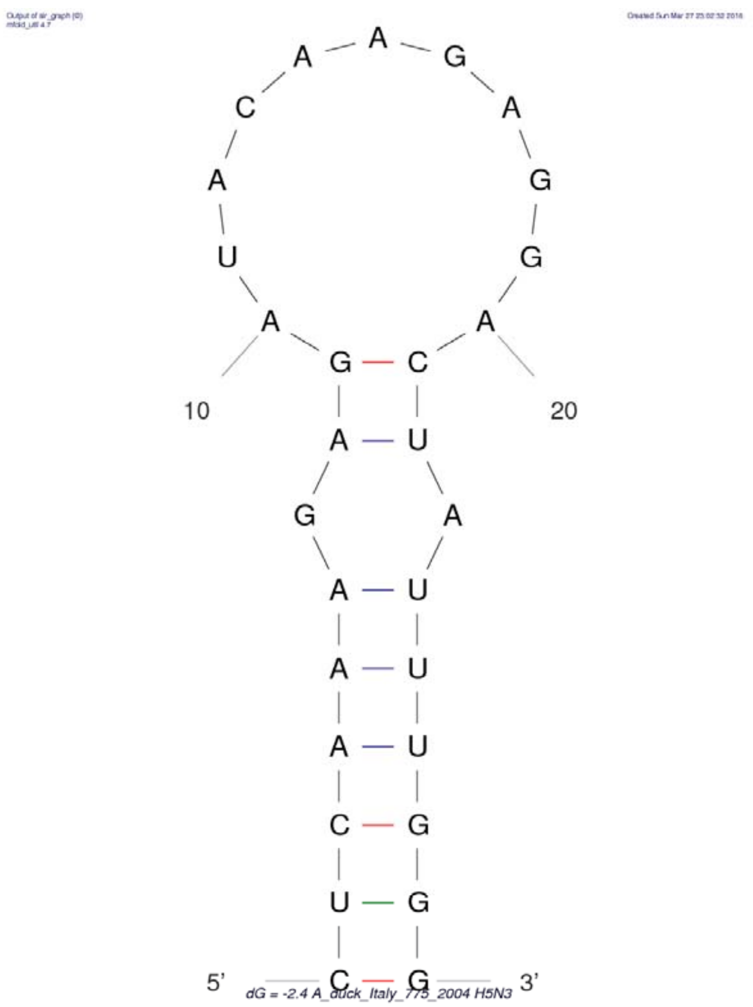

dG = -2.4 A_duck_Italy_775_2004 H5N3

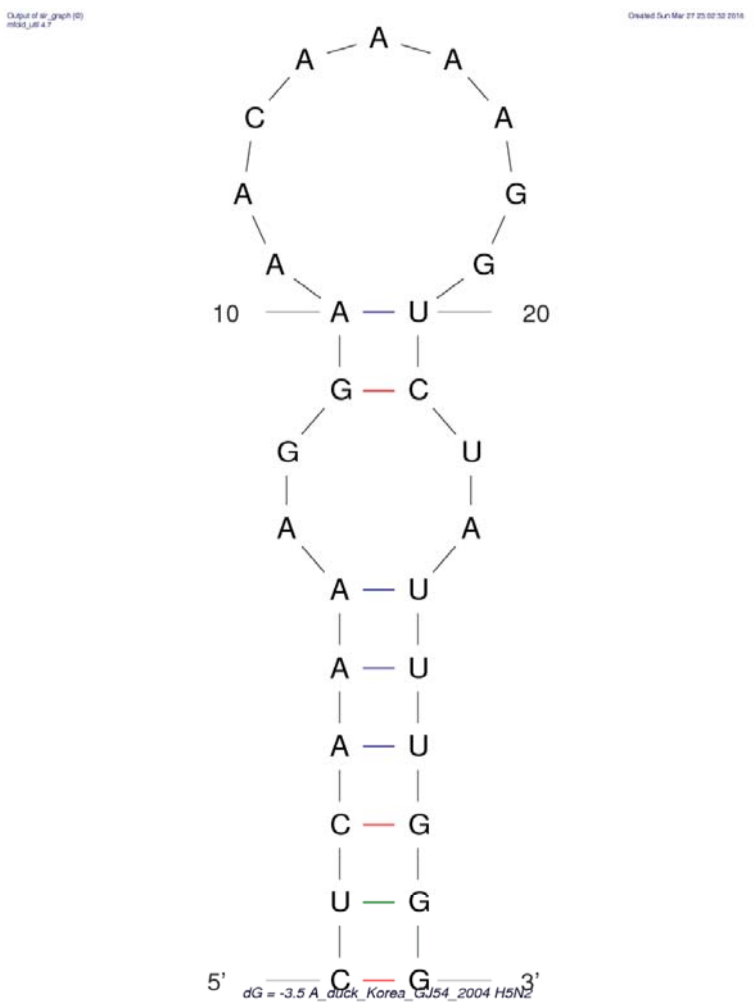

dG = -3.5 A_duck_Korea_GJ54_2004 H5N2

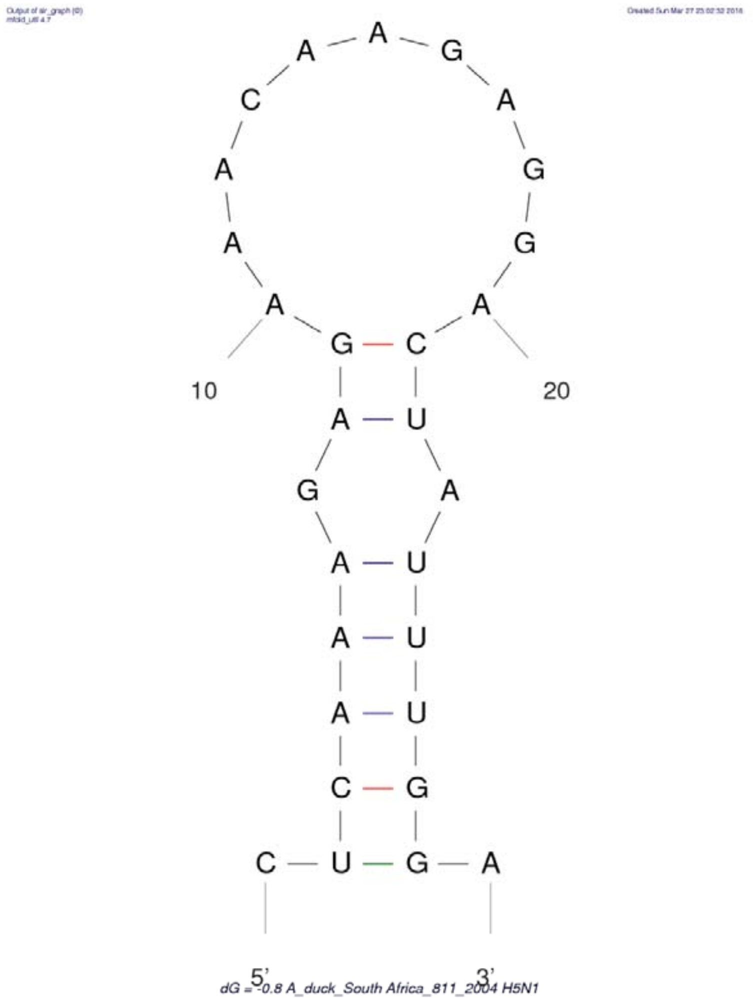

dG = -0.8 A_duck_South Africa_811_2004 H5N1

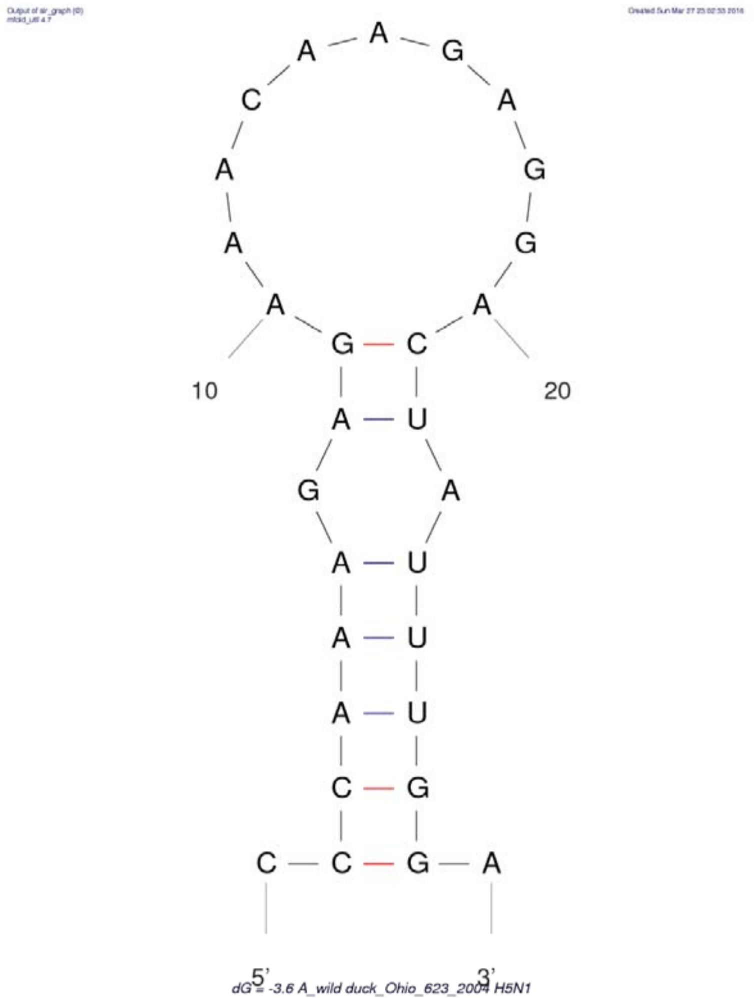

dG = -3.6 A_wild duck_Ohio_623_2004 H5N1

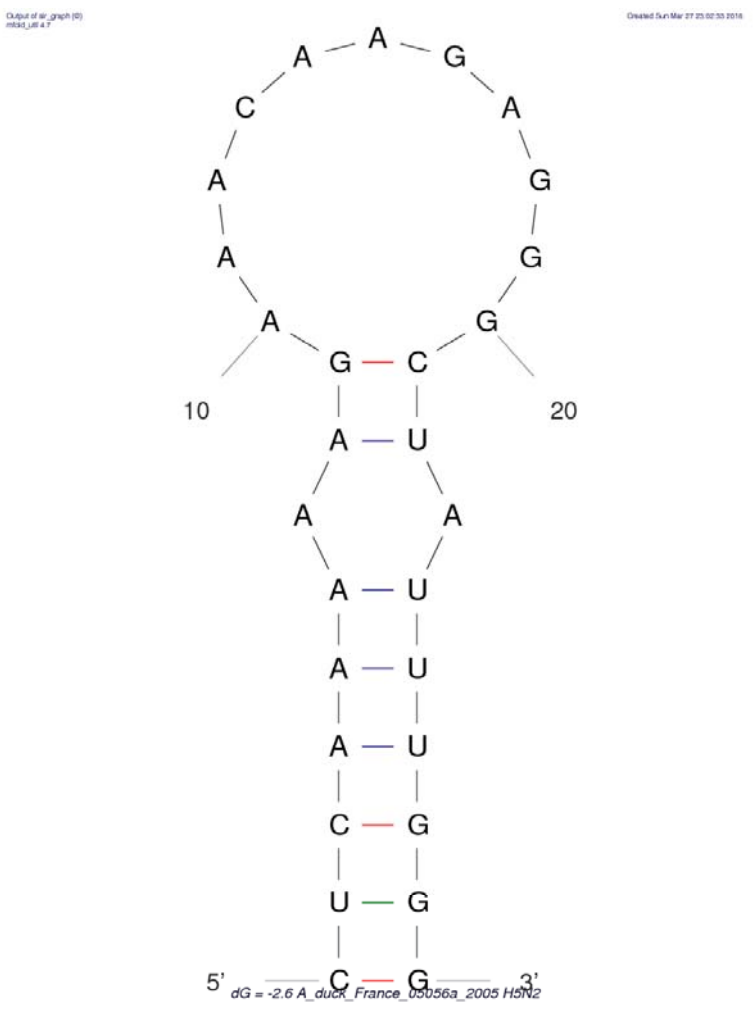
Output of sir_graph (©)
mfold_util 4.7
Created Sun Mar 27 23:02:33 2016
10
20
5'
3'
dG = -2.6 A_duck_France_05056a_2005 H5N2

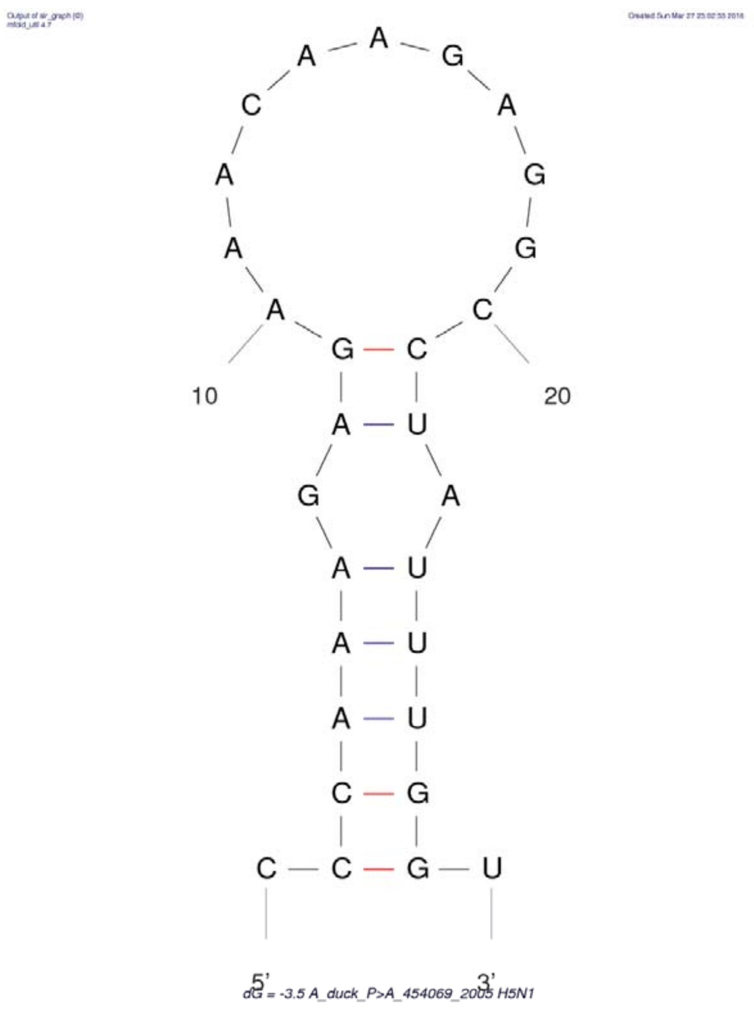
Output of sir_graph (©)
mfold_util 4.7
Created Sun Mar 27 23:02:33 2016
10
20
5'
3'
dG = -3.5 A_duck_P>A_454069_2005 H5N1

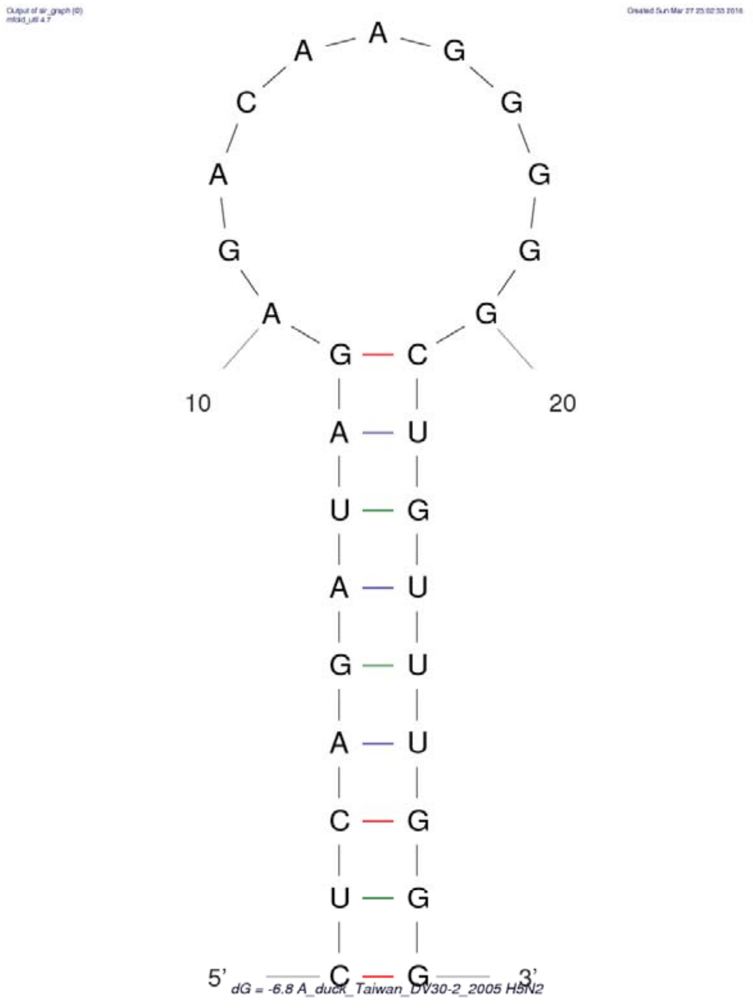
Output of sir_graph (©)
mfold_util 4.7
Created Sun Mar 27 23:02:33 2016
10
20
5'
3'
dG = -6.8 A_duck_Taiwan_DV30-2_2005 H5N2

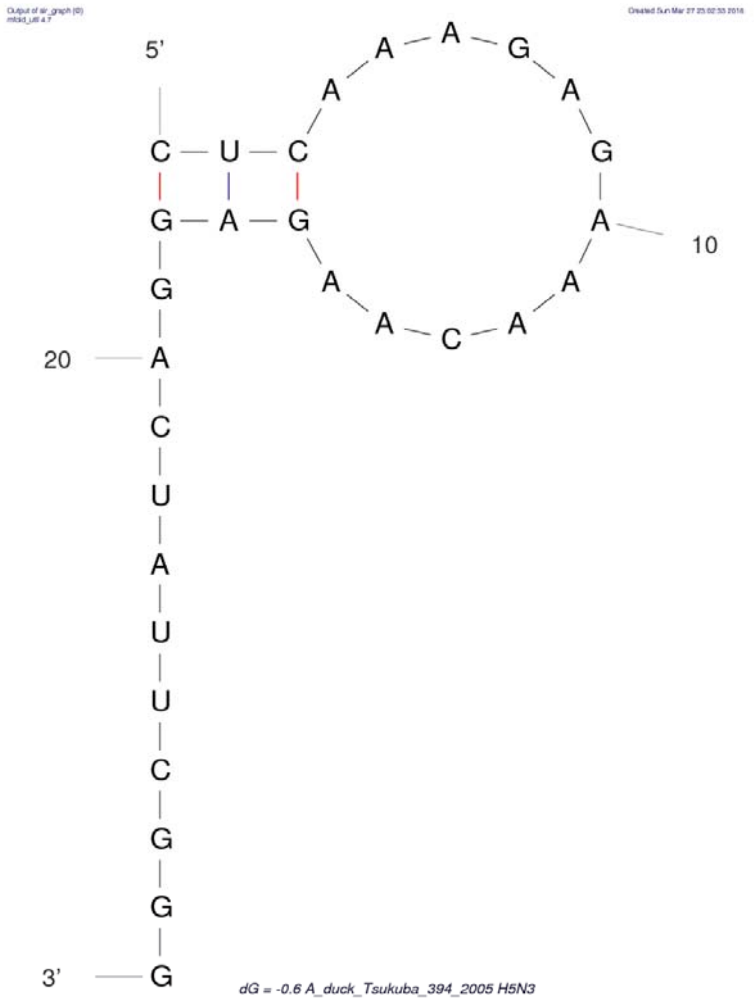
Output of sir_graph (©)
mfold_util 4.7
Created Sun Mar 27 23:02:33 2016
5'
10
20
3'
dG = -0.6 A_duck_Tsukuba_394_2005 H5N3

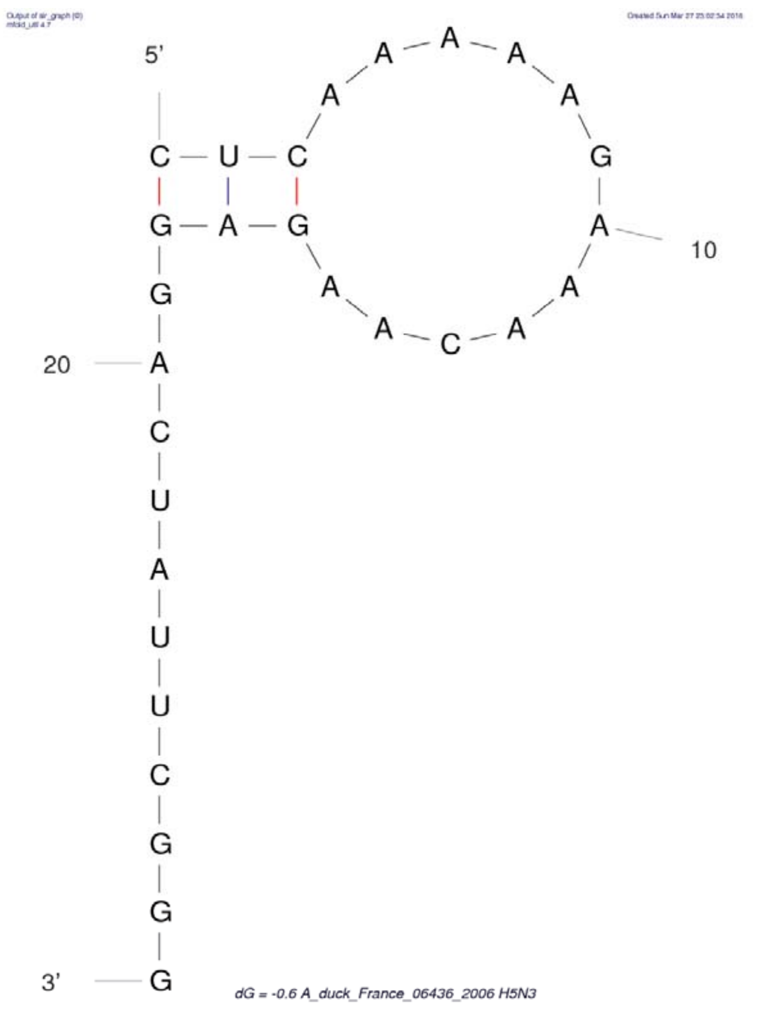
5'
10
20
3'
dG = -0.6 A_duck_France_06436_2006 H5N3

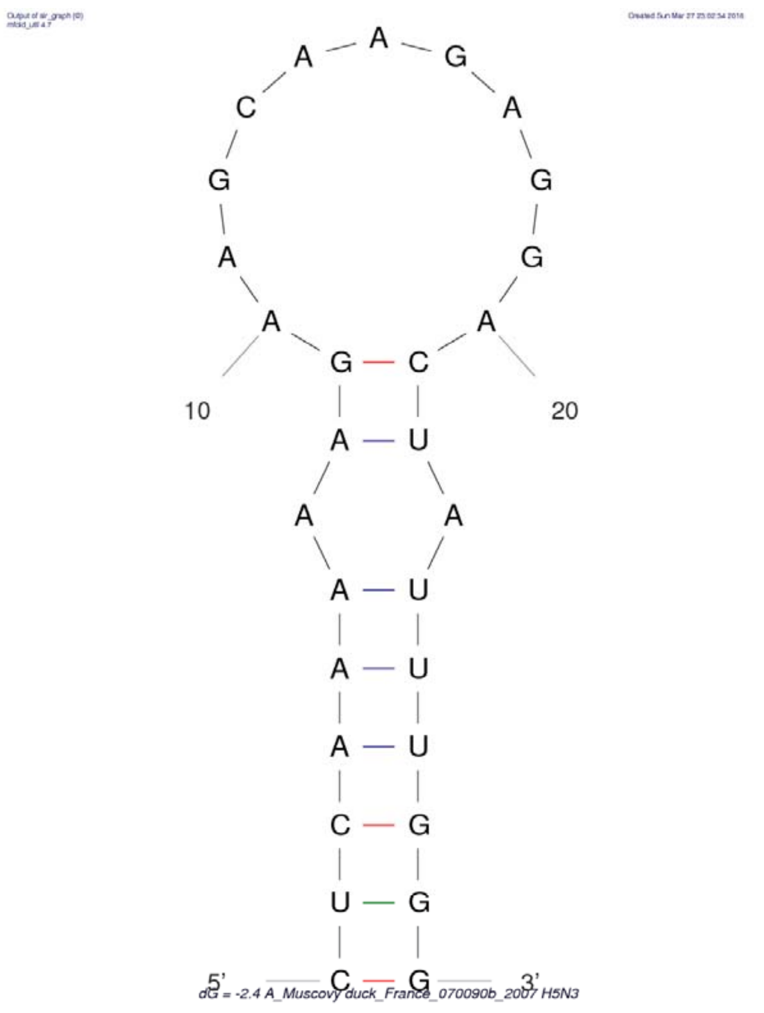
10
20
5'
3'
dG = -2.4 A_Muscovy duck_France_070090b_2007 H5N3

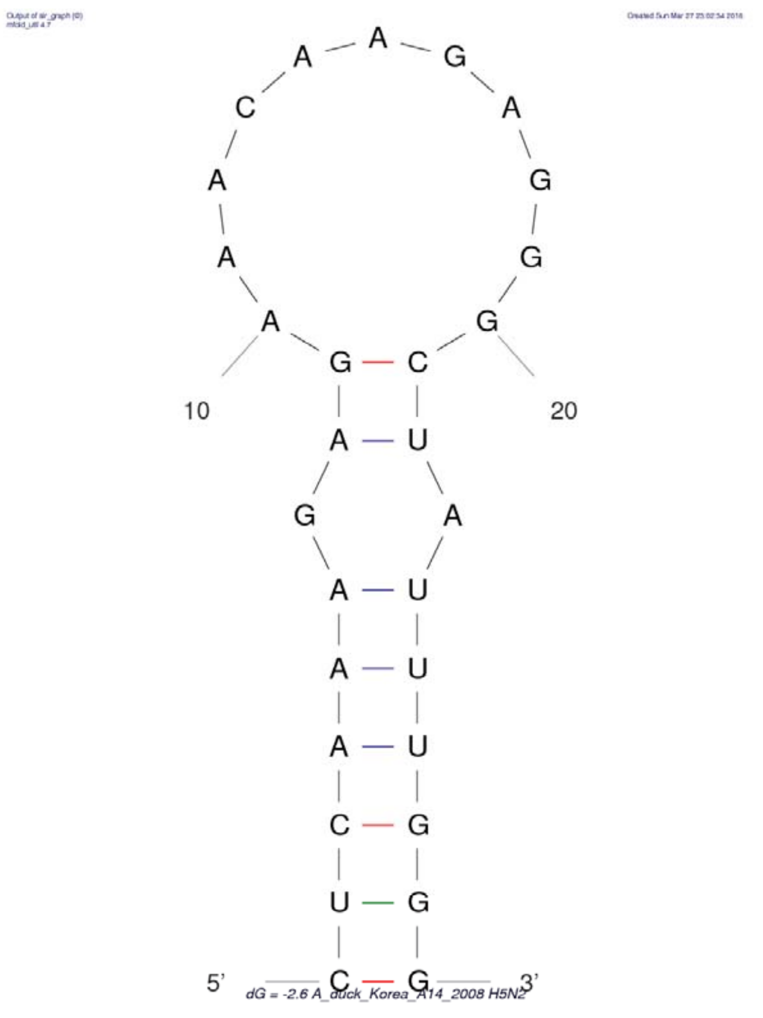
10
20
5'
3'
dG = -2.6 A_duck_Korea_A14_2008 H5N2

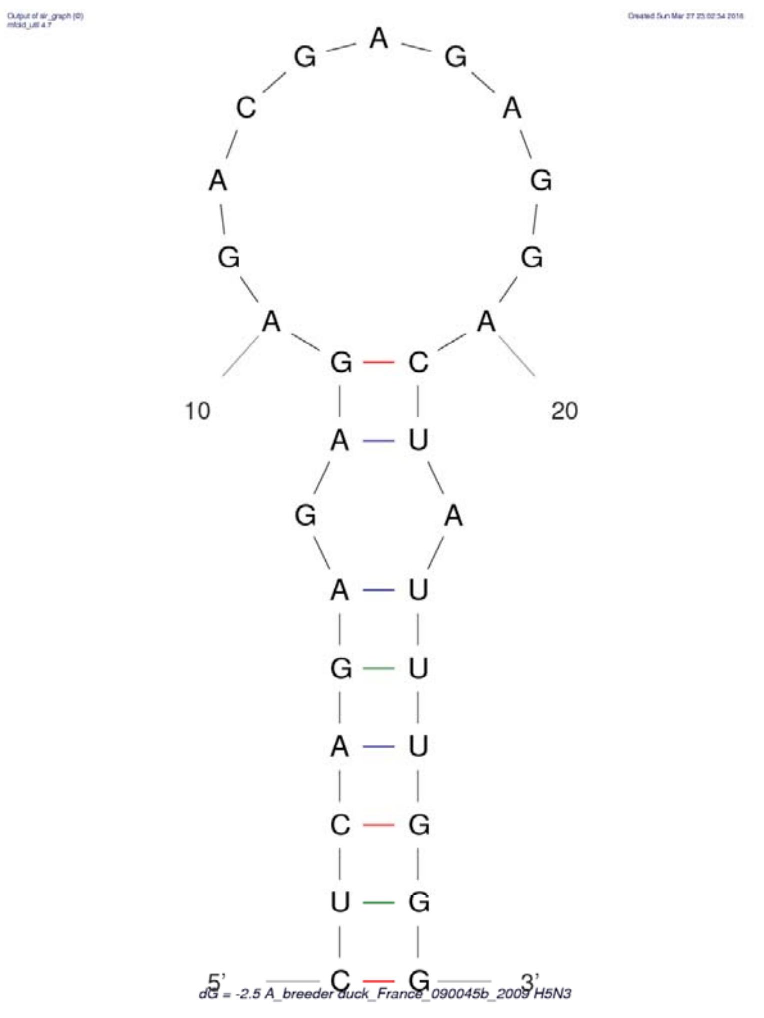
10
20
5'
3'
dG = -2.5 A_breeder duck_France_090045b_2009 H5N3

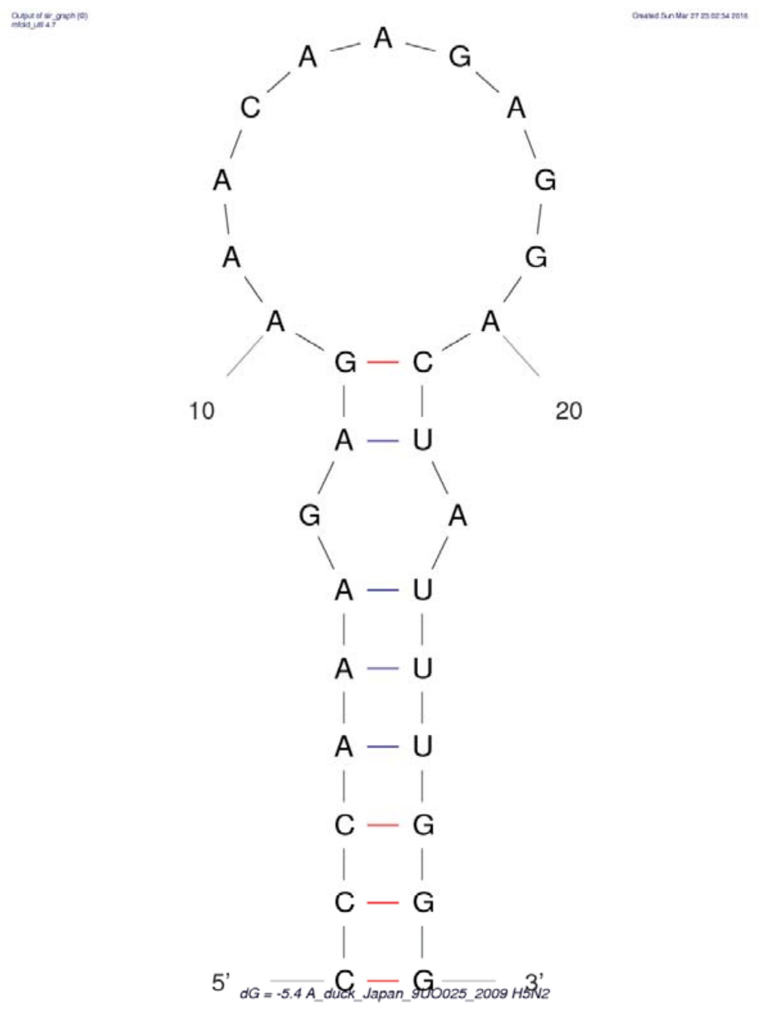

Output of sir_graph (©)
mfold_util 4.7
Created Sun Mar 27 23:02:54 2016
10
20
5'
3'
dG = -5.4 A_duck_Japan_9UO025_2009 H5N2

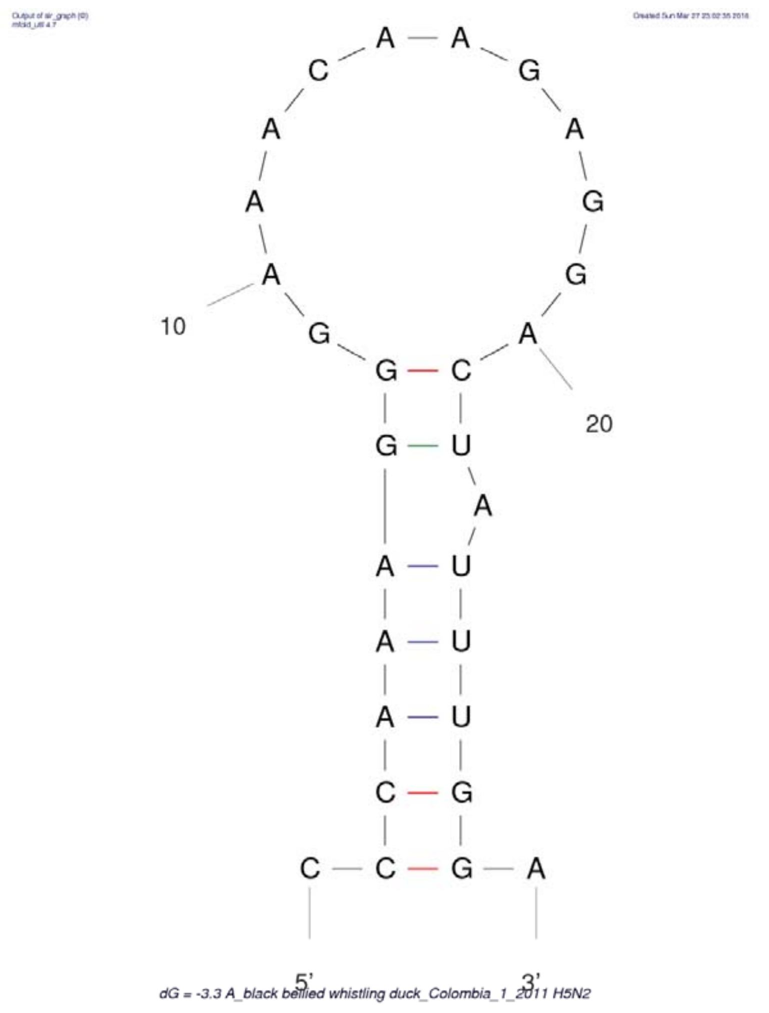

Output of sir_graph (©)
mfold_util 4.7
Created Sun Mar 27 23:02:55 2016
10
20
5'
3'
dG = -3.3 A_black bellied whistling duck_Colombia_1_2011 H5N2

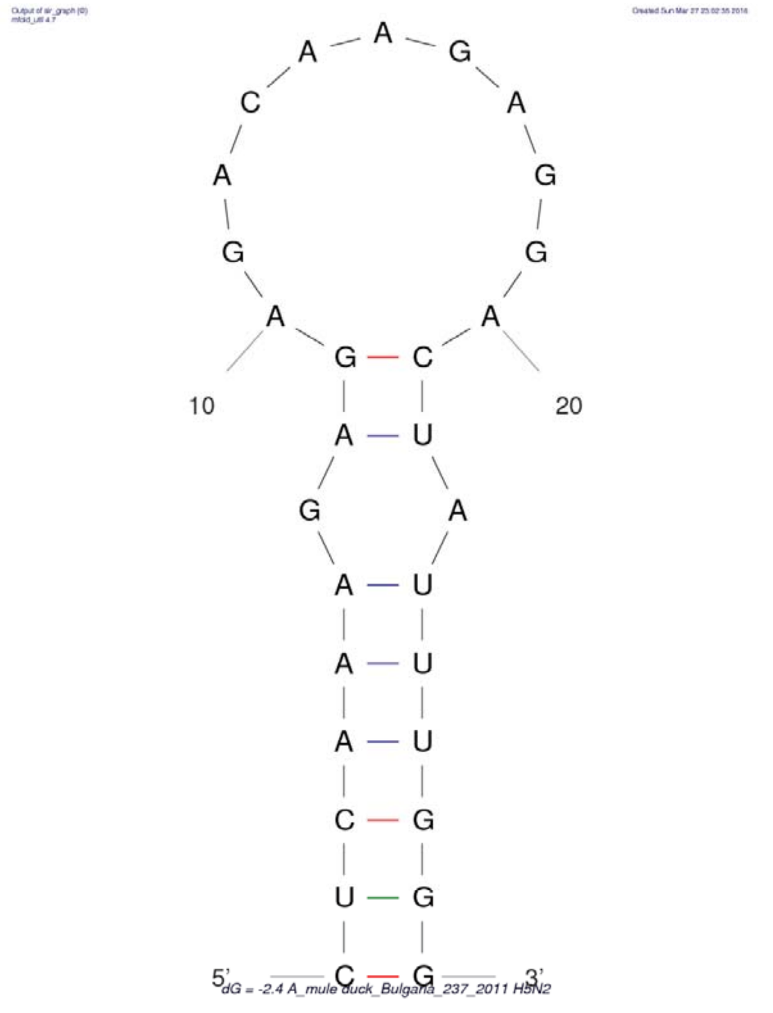

Output of sir_graph (©)
mfold_util 4.7
Created Sun Mar 27 23:02:55 2016
10
20
5'
3'
dG = -2.4 A_mule duck_Bulgaria_237_2011 H5N2

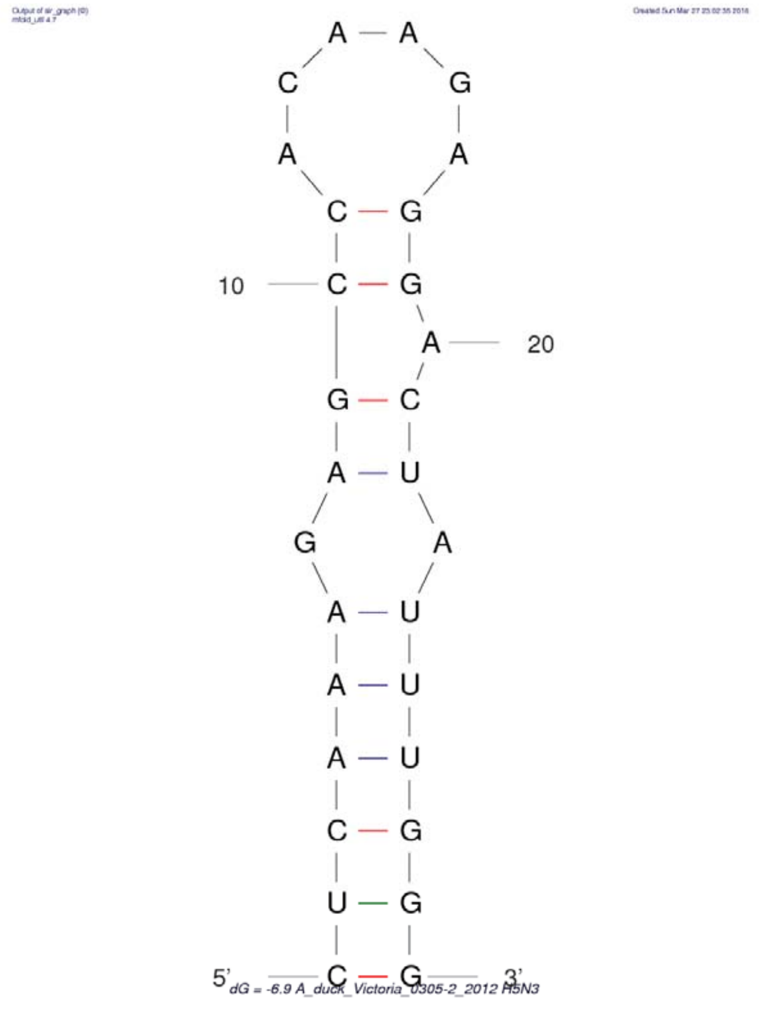

Output of sir_graph (©)
mfold_util 4.7
Created Sun Mar 27 23:02:55 2016
10
20
5'
3'
dG = -6.9 A_duck_Victoria_0305-2_2012 H5N3

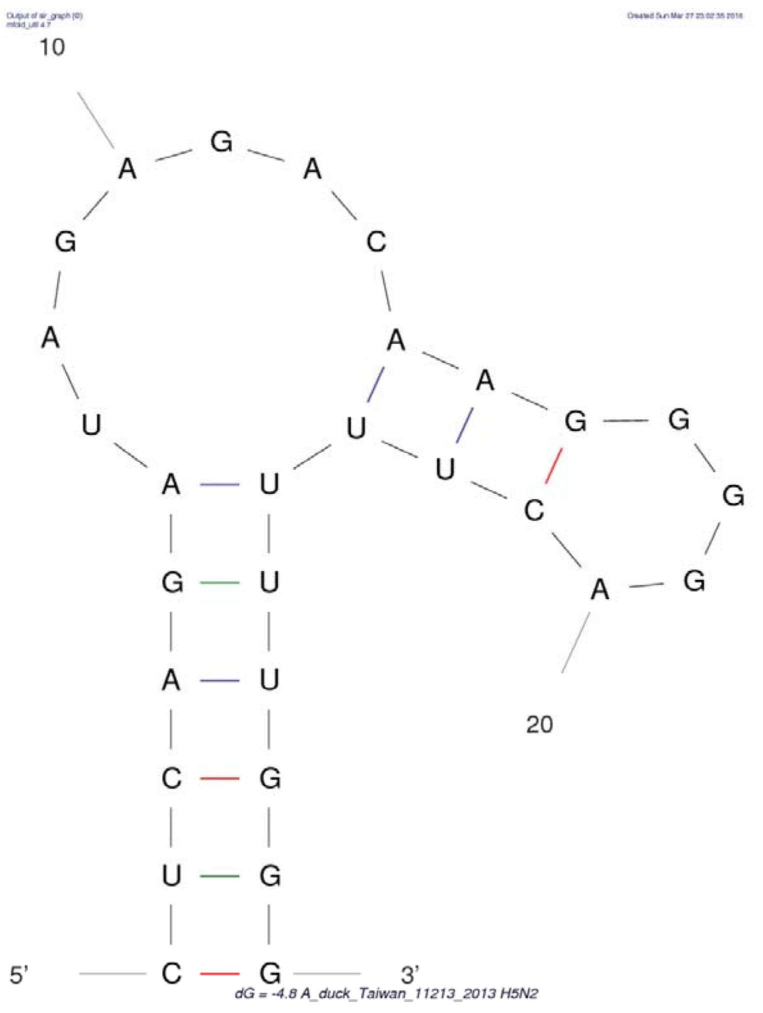
5'
3'
10
20
dG = -4.8 A_duck_Taiwan_11213_2013 H5N2

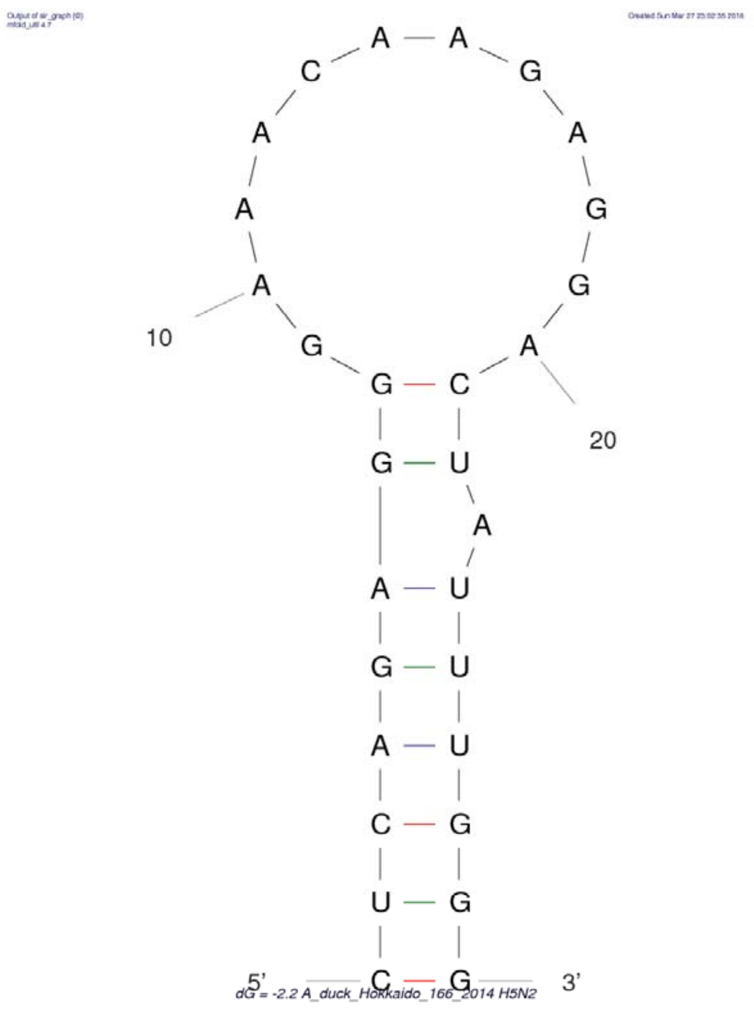
10
20
5'
3'
dG = -2.2 A_duck_Hokkaido_166_2014 H5N2

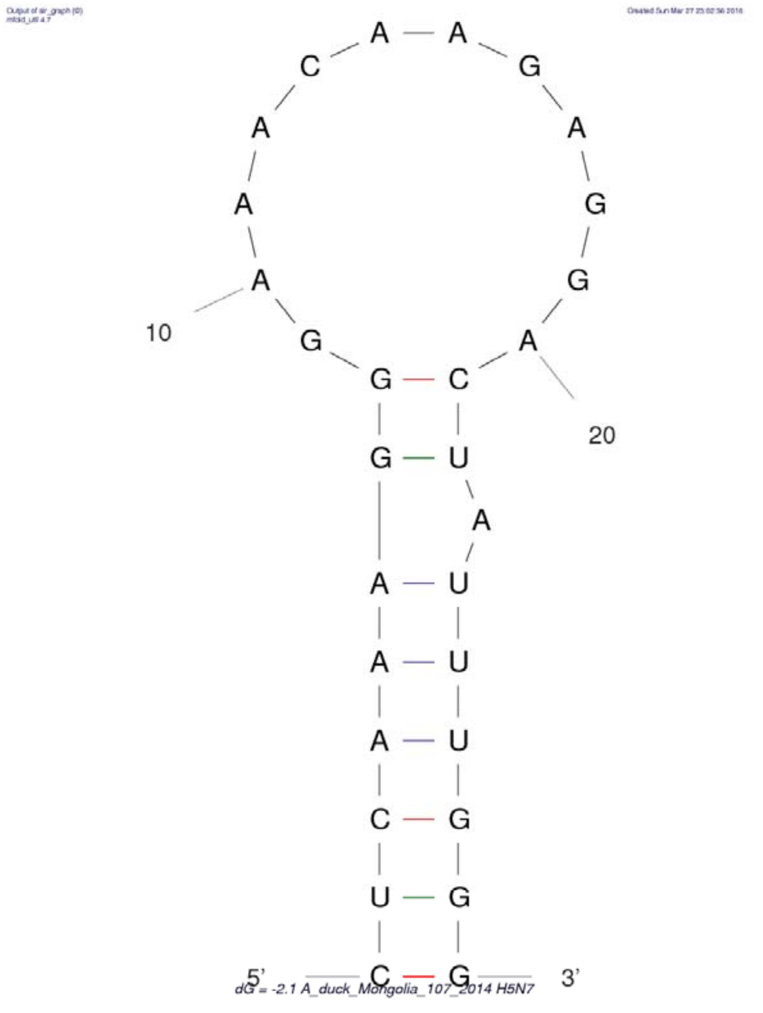
10
20
5'
3'
dG = -2.1 A_duck_Mongolia_107_2014 H5N7

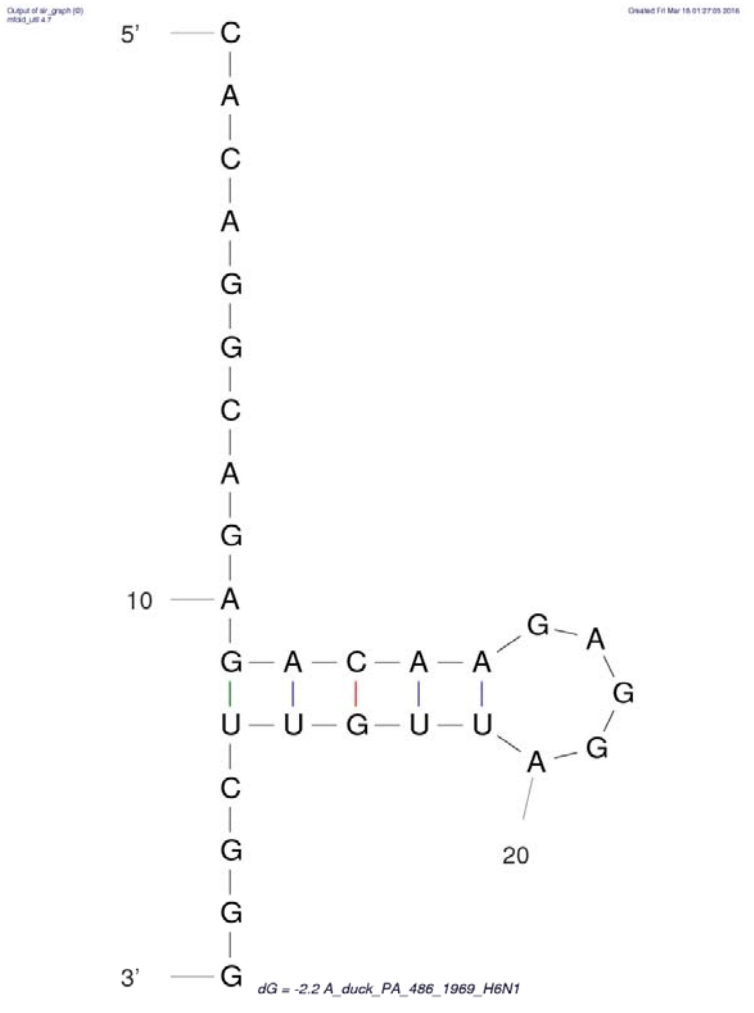
5'
10
20
3'
dG = -2.2 A_duck_PA_486_1969_H6N1

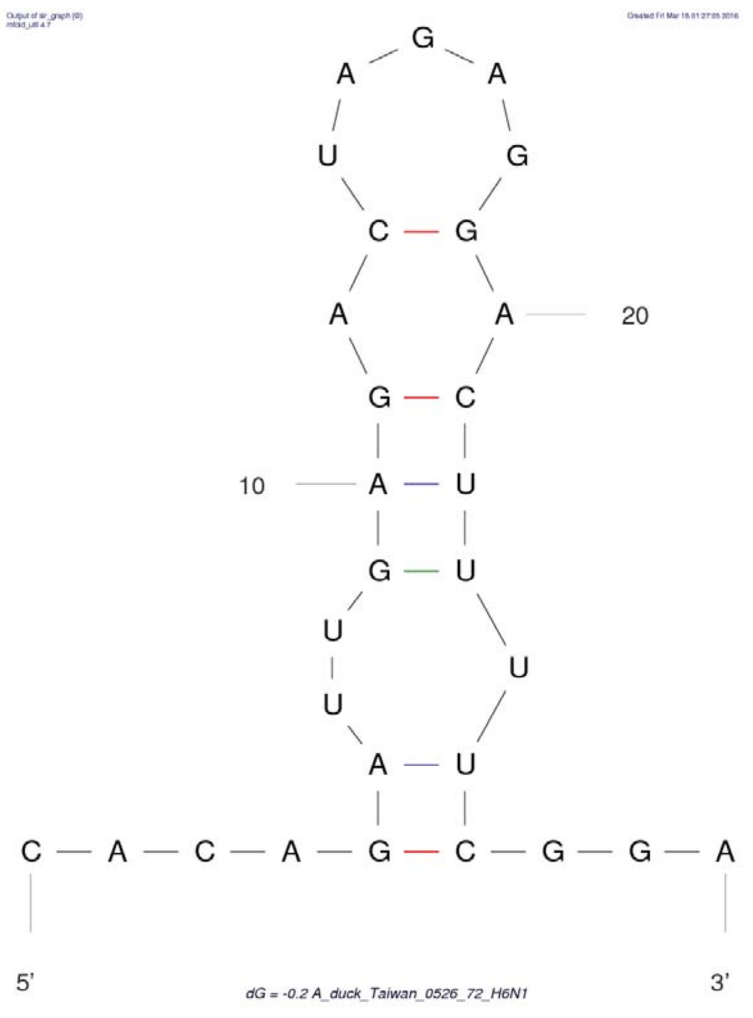
20
10
5'
3'
dG = -0.2 A_duck_Taiwan_0526_72_H6N1

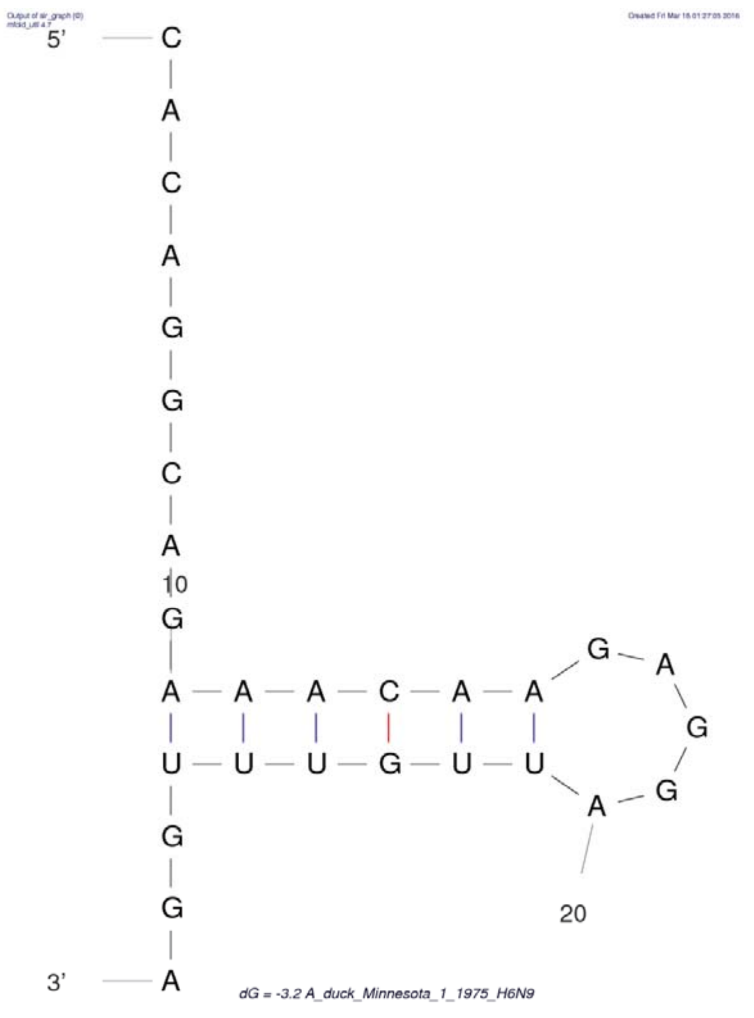
5'
10
20
3'
dG = -3.2 A_duck_Minnesota_1_1975_H6N9

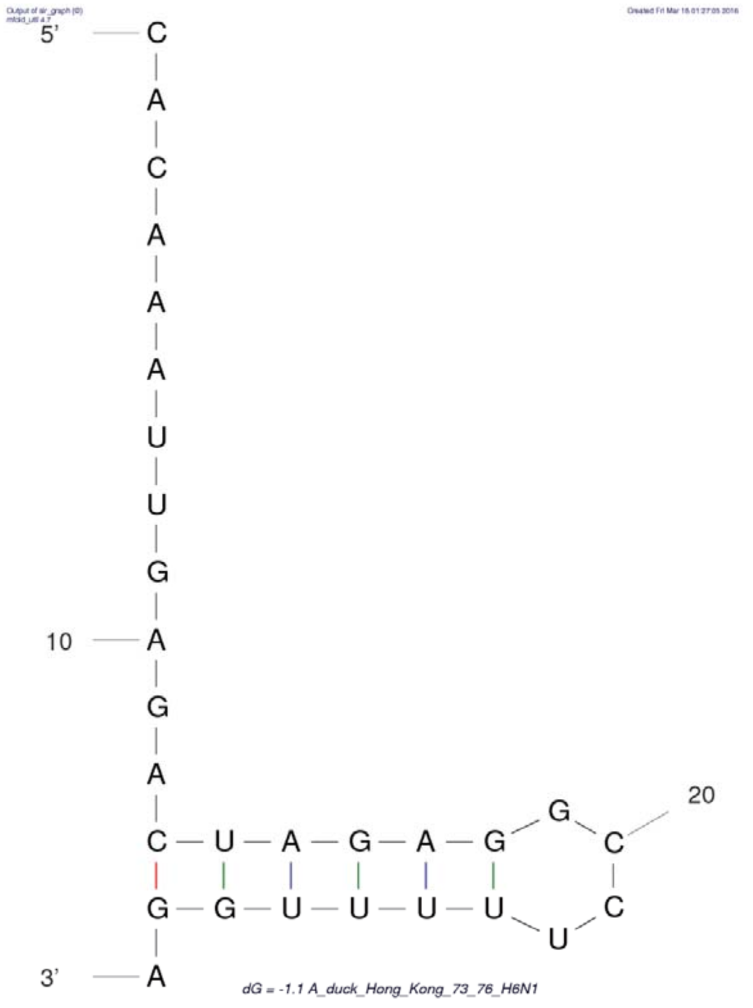
5'
10
20
3'
dG = -1.1 A_duck_Hong_Kong_73_76_H6N1

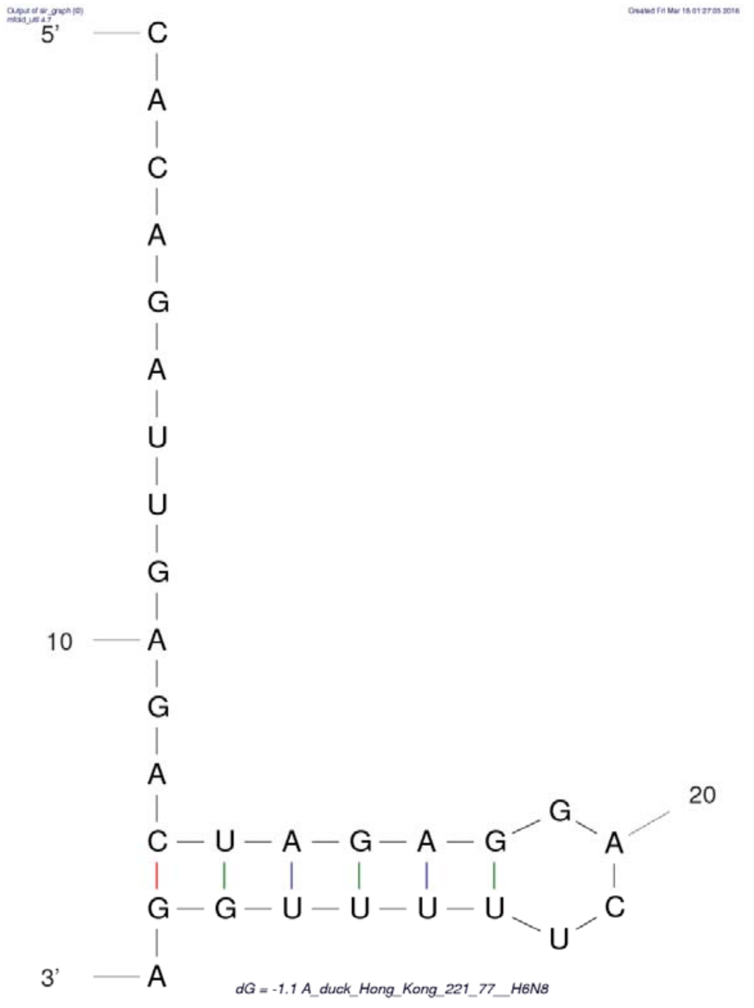
5'
10
20
3'
dG = -1.1 A_duck_Hong_Kong_221_77__H6N8

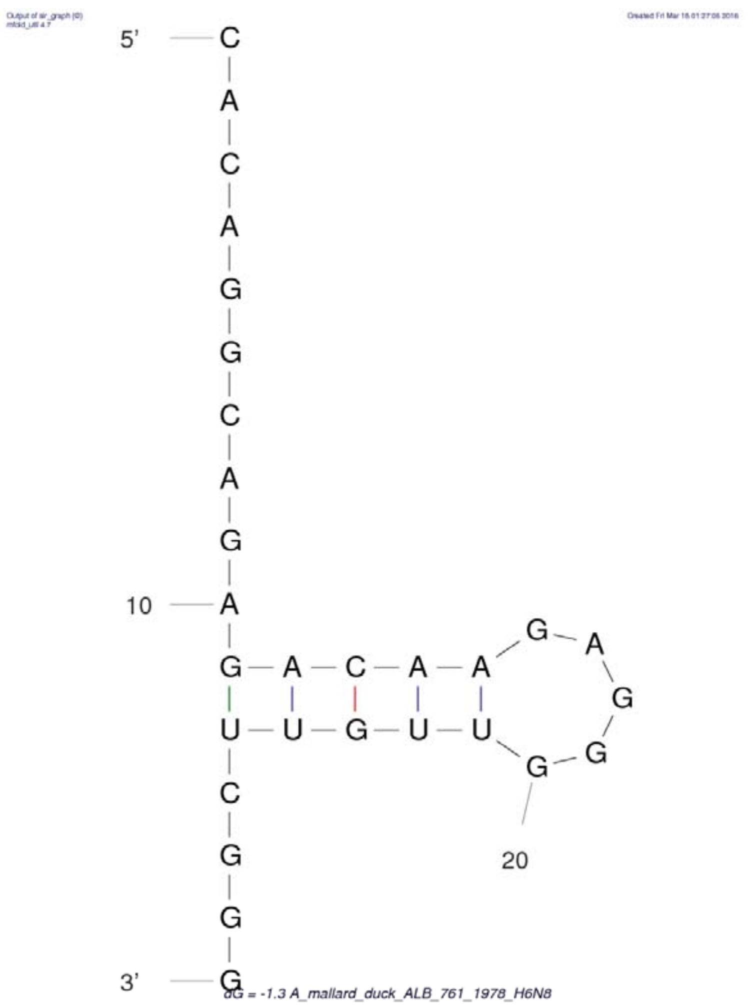
Output of sir_graph (©)
mfold_util 4.7
Created Fri Mar 18 01:27:06 2016
5'
3'
10
20
dG = -1.3 A_mallard_duck_ALB_761_1978_H6N8

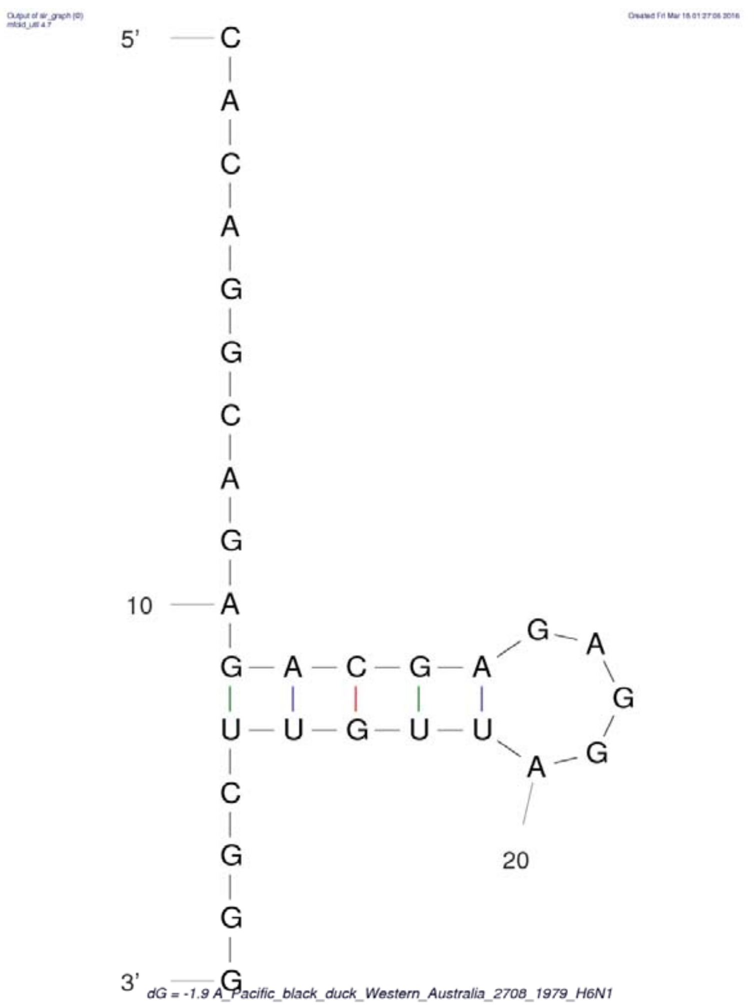
Output of sir_graph (©)
mfold_util 4.7
Created Fri Mar 18 01:27:06 2016
5'
3'
10
20
dG = -1.9 A_Pacific_black_duck_Western_Australia_2708_1979_H6N1

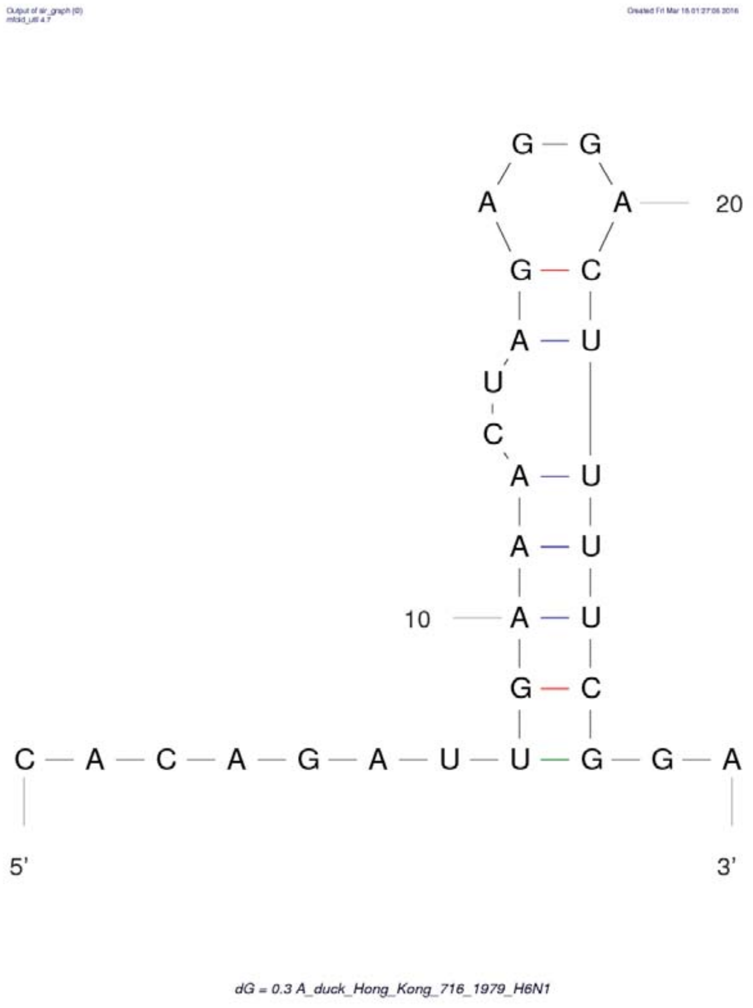
Output of sir_graph (©)
mfold_util 4.7
Created Fri Mar 18 01:27:06 2016
20
10
5'
3'
dG = 0.3 A_duck_Hong_Kong_716_1979_H6N1

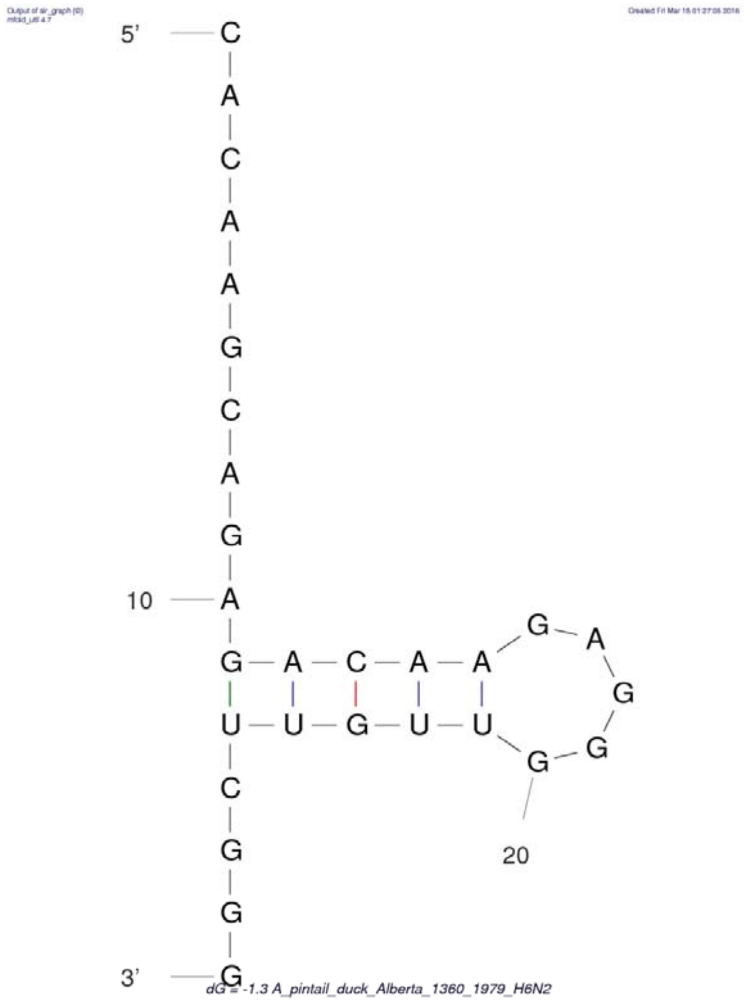
Output of sir_graph (©)
mfold_util 4.7
Created Fri Mar 18 01:27:06 2016
5'
10
20
3'
dG = -1.3 A_pintail_duck_Alberta_1360_1979_H6N2

Output of sir_graph (©)
mfold_util 4.7

Created Fri Mar 18 01:27:06 2016

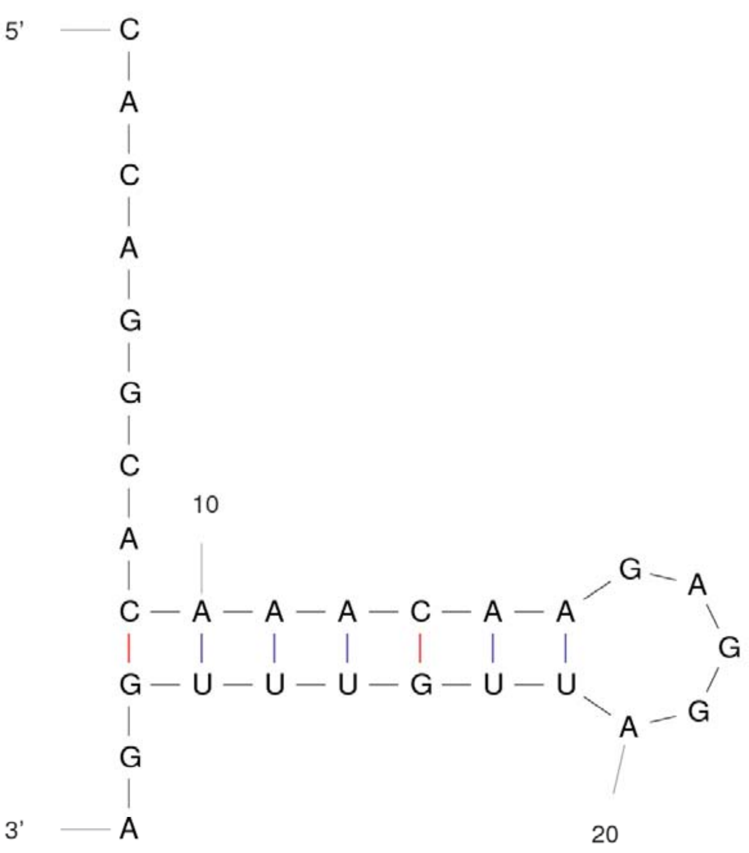

dG = -6.2 A_black_duck_Ohio_101_1986_H6N2

Output of sir_graph (©)
mfold_util 4.7

Created Fri Mar 18 01:27:07 2016

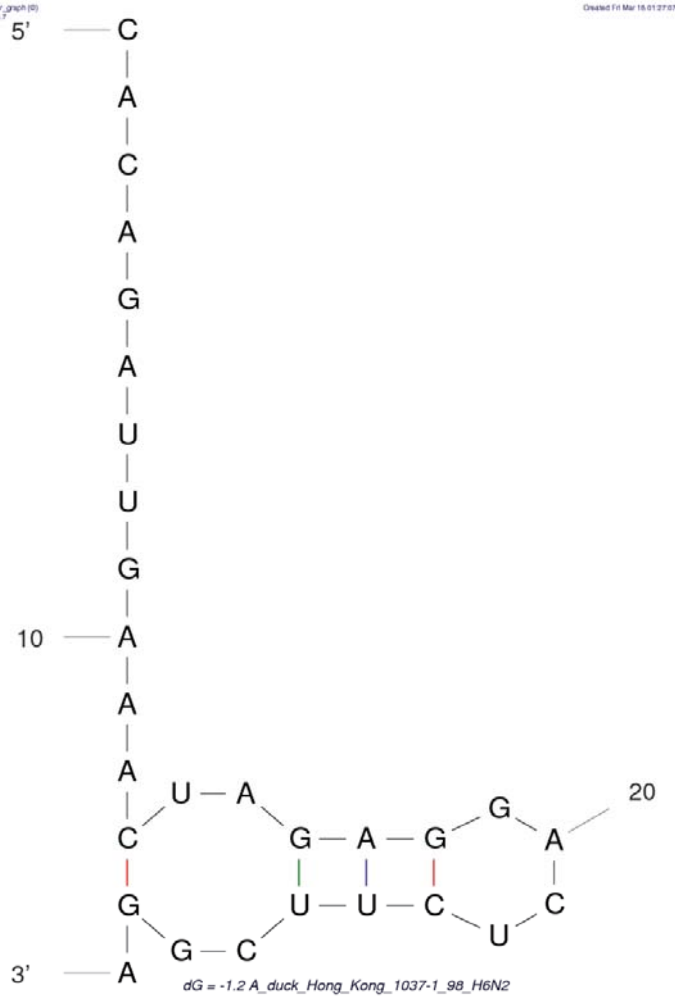

dG = -1.2 A_duck_Hong_Kong_1037-1_98_H6N2

Output of sir_graph (©)
mfold_util 4.7

Created Fri Mar 18 01:27:07 2016

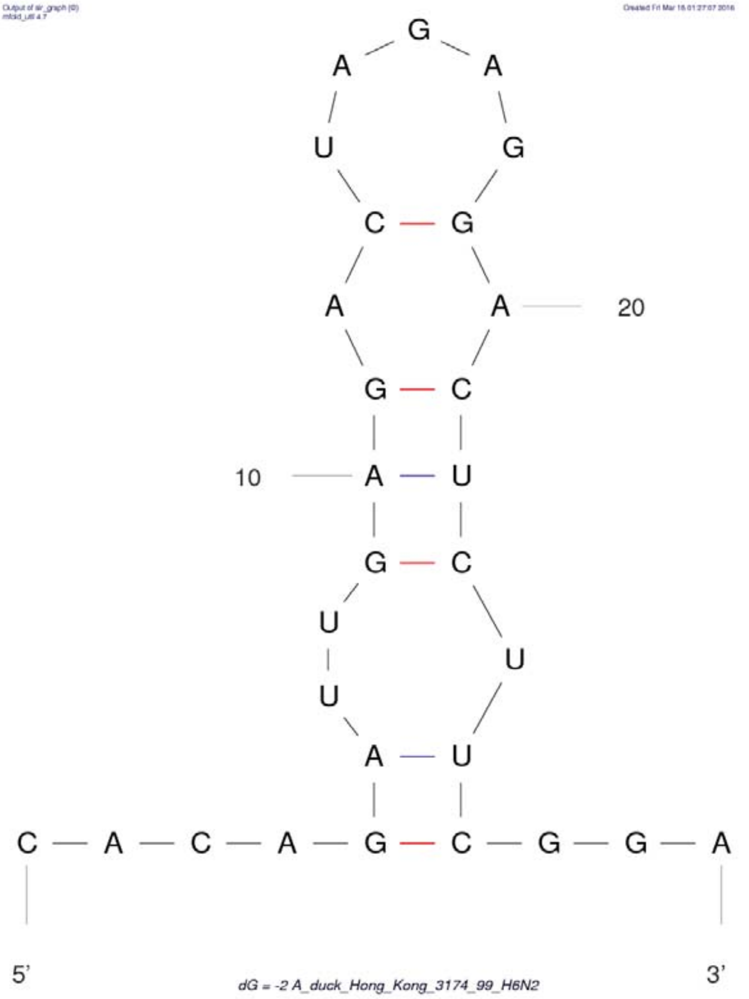

dG = -2 A_duck_Hong_Kong_3174_99_H6N2

Output of sir_graph (©)
mfold_util 4.7

Created Fri Mar 18 01:27:07 2016

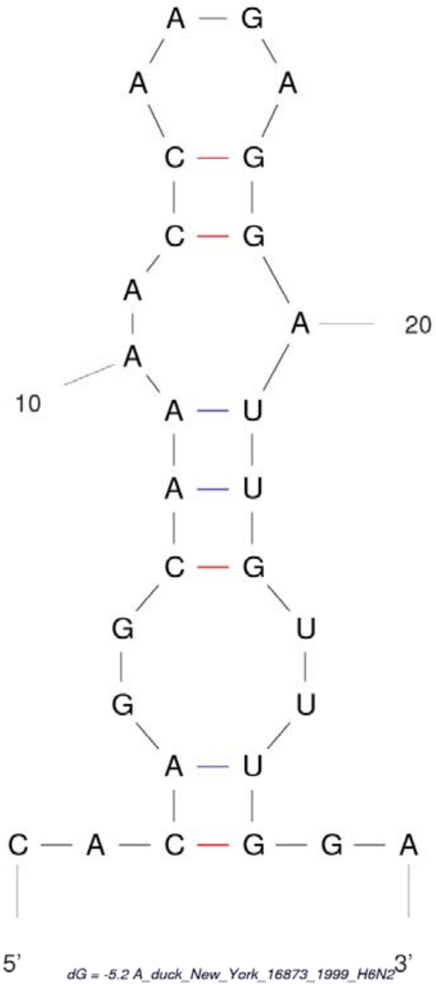

dG = -5.2 A_duck_New_York_16873_1999_H6N2

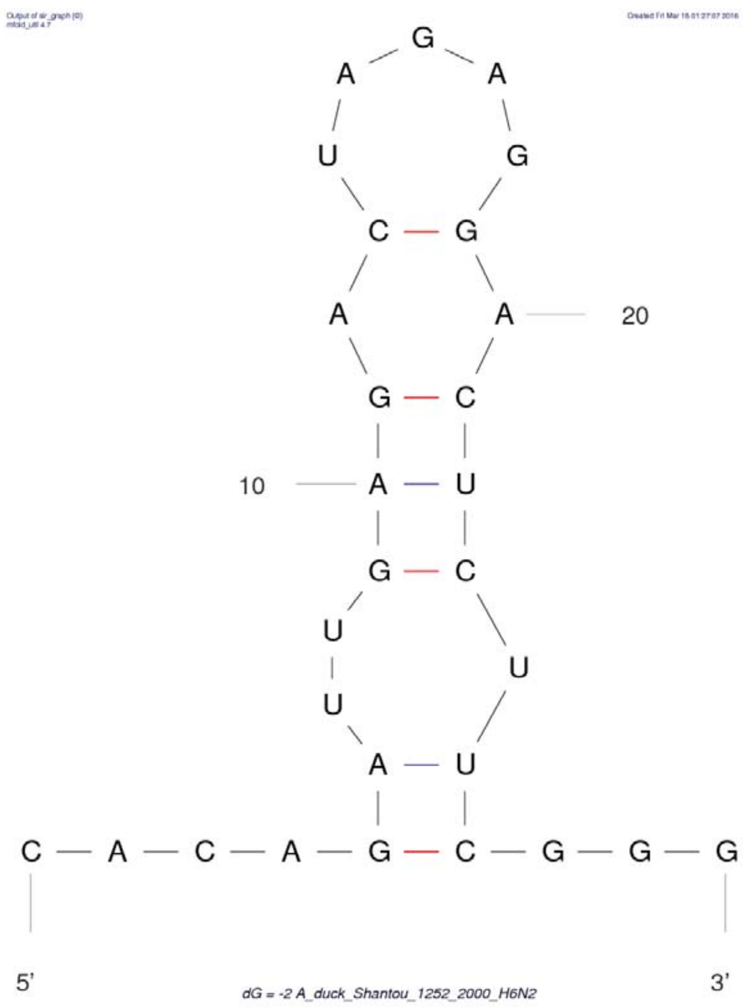

20
10
5’
3’
dG = -2 A_duck_Shantou_1252_2000_H6N2

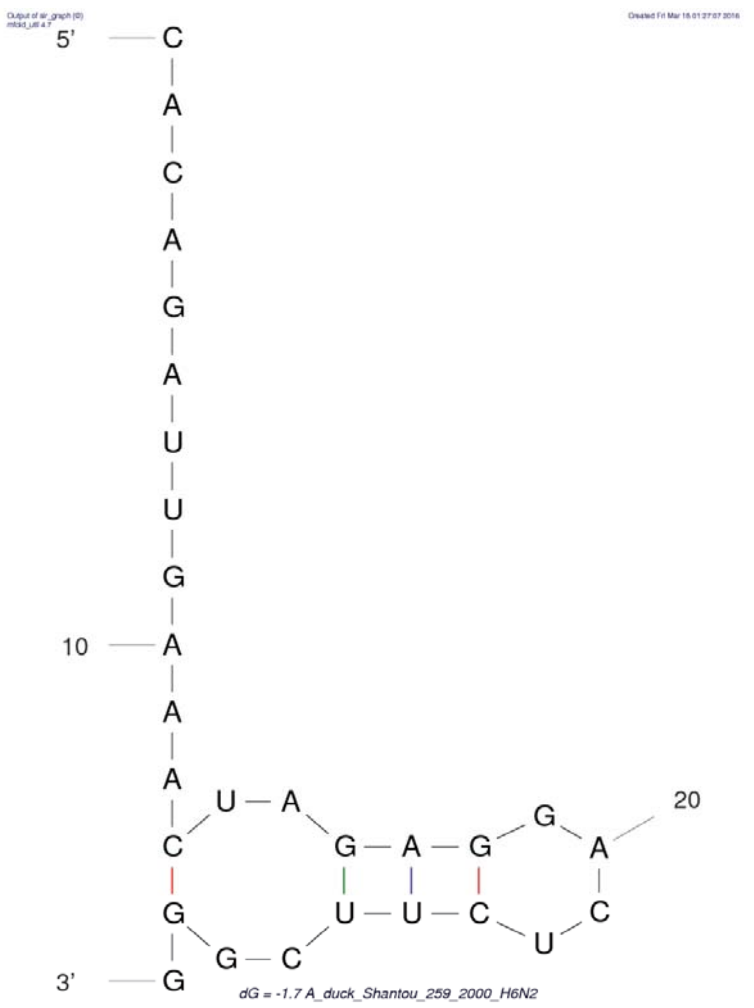

5’
10
20
3’
dG = -1.7 A_duck_Shantou_259_2000_H6N2

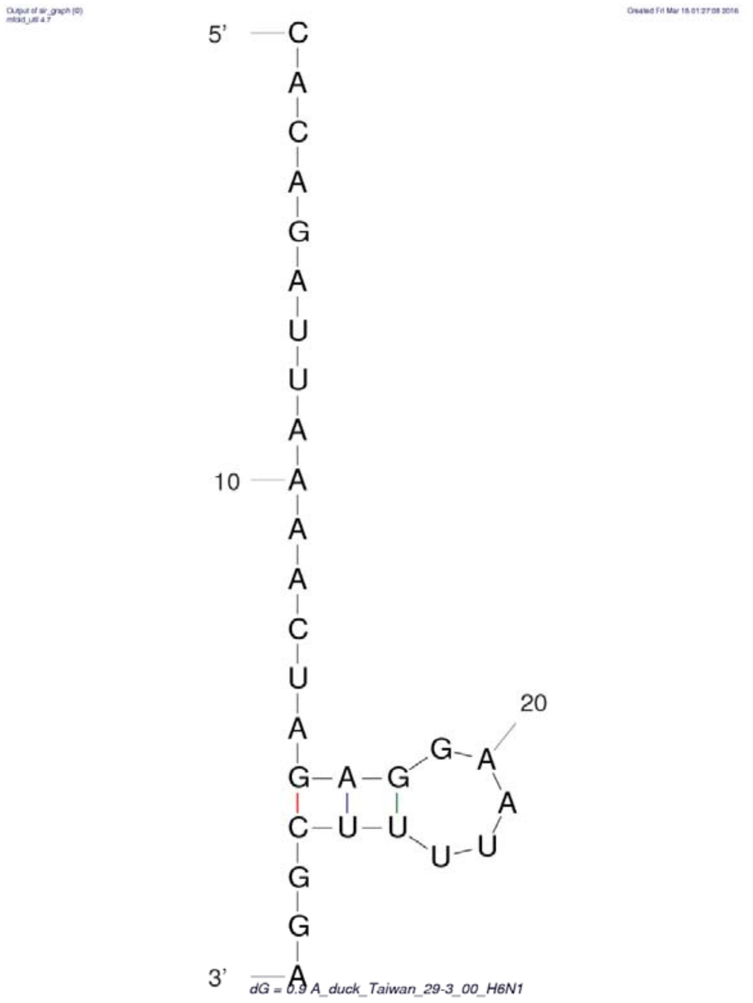

5’
10
20
3’
dG = 0.9 A_duck_Taiwan_29-3_00_H6N1

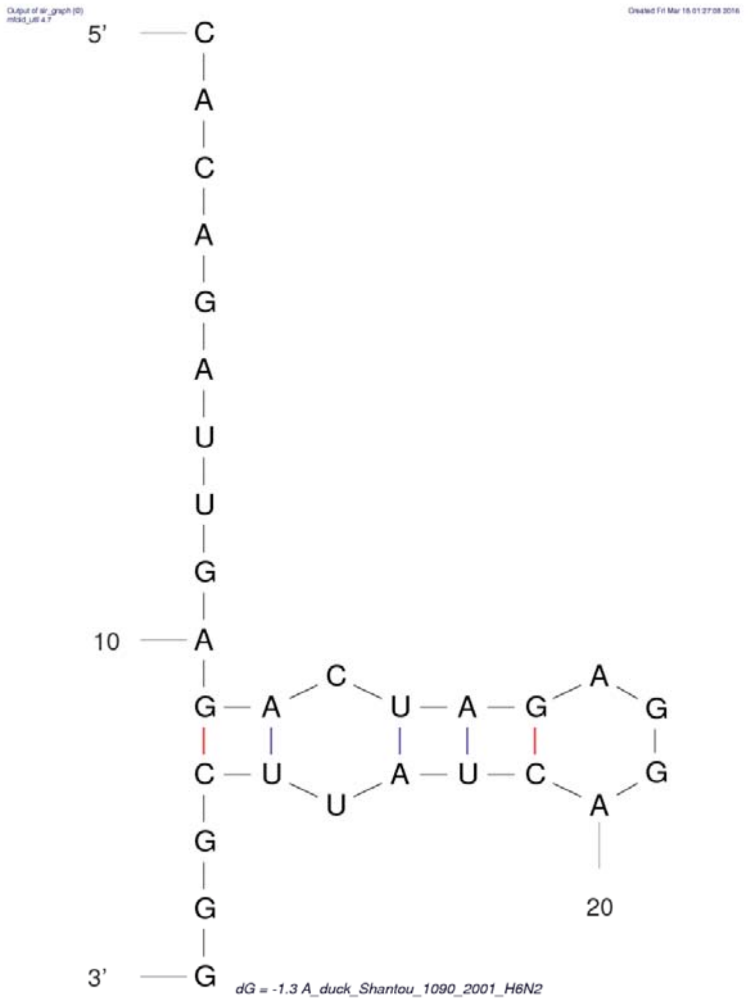

5’
10
20
3’
dG = -1.3 A_duck_Shantou_1090_2001_H6N2

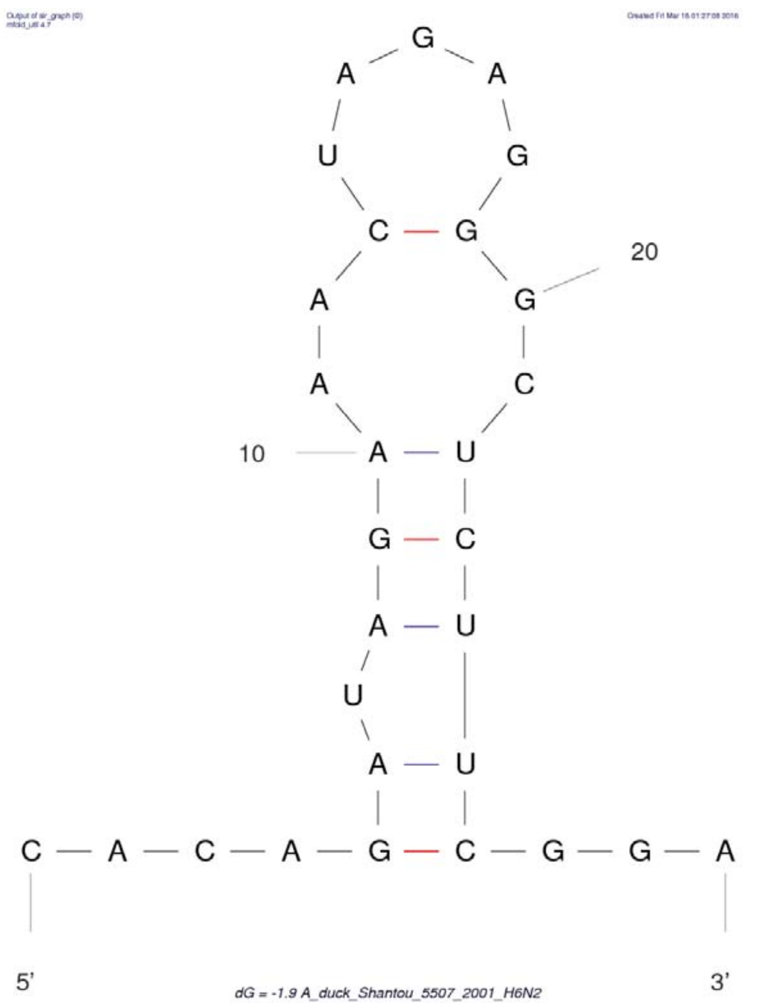

dG = -1.9 A_duck_Shantou_5507_2001_H6N2

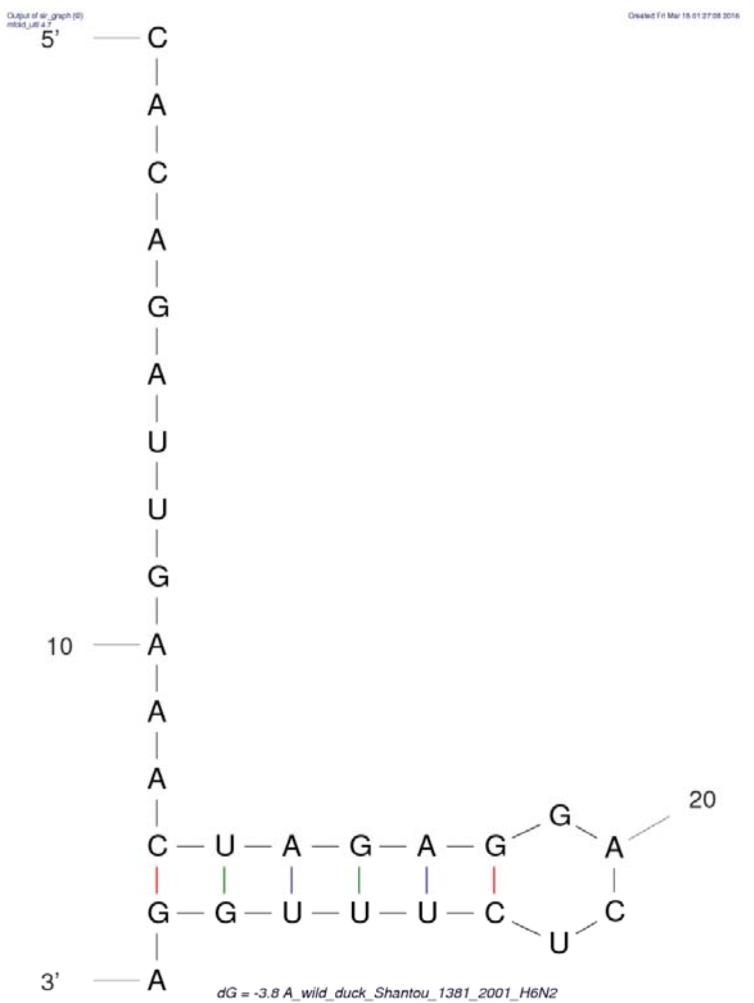

dG = -3.8 A_wild_duck_Shantou_1381_2001_H6N2

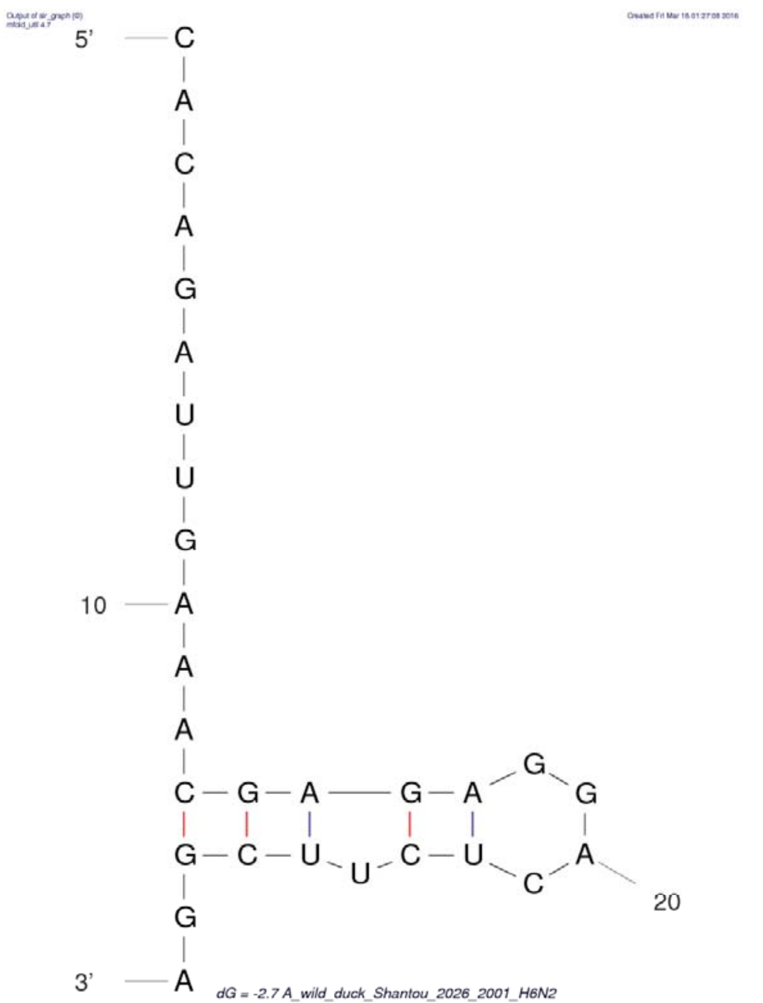

dG = -2.7 A_wild_duck_Shantou_2026_2001_H6N2

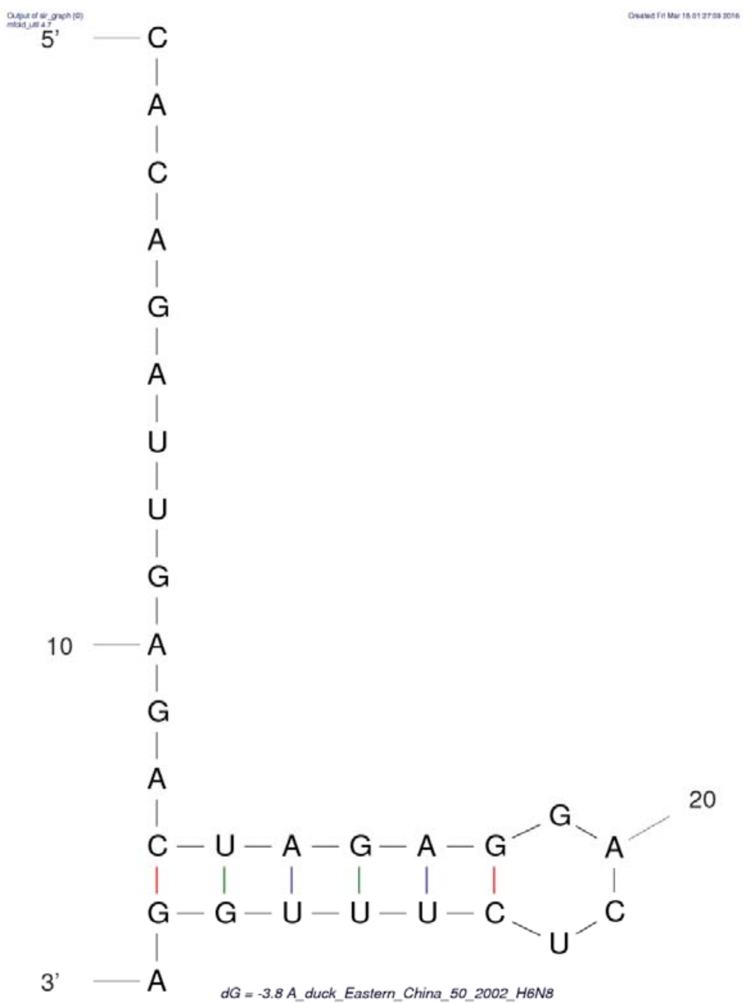

dG = -3.8 A_duck_Eastern_China_50_2002_H6N8

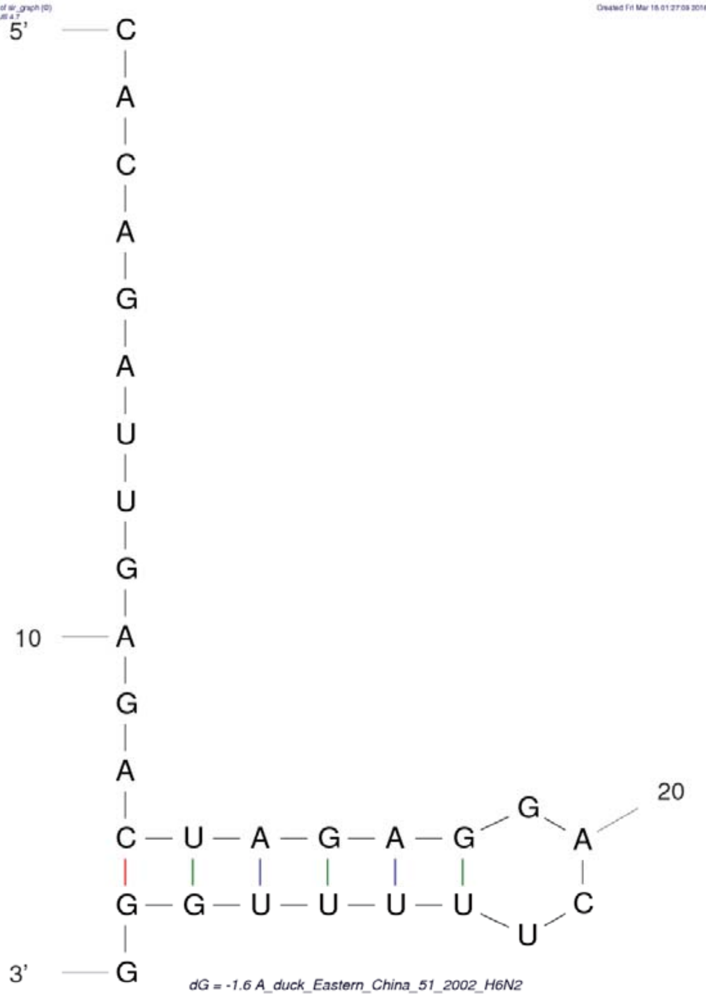

dG = -1.6 A_duck_Eastern_China_51_2002_H6N2

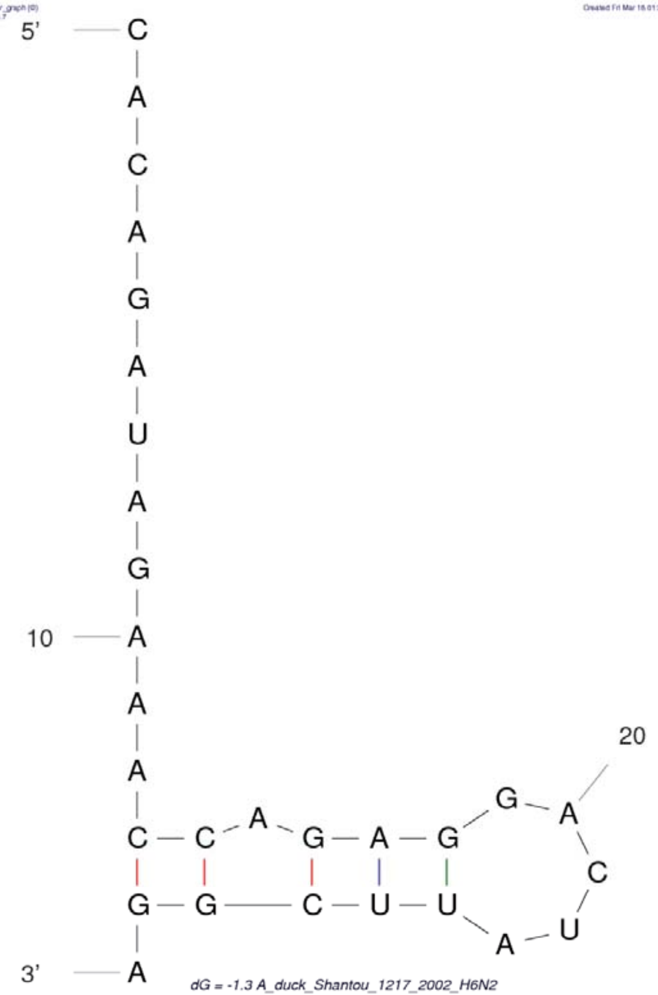

dG = -1.3 A_duck_Shantou_1217_2002_H6N2

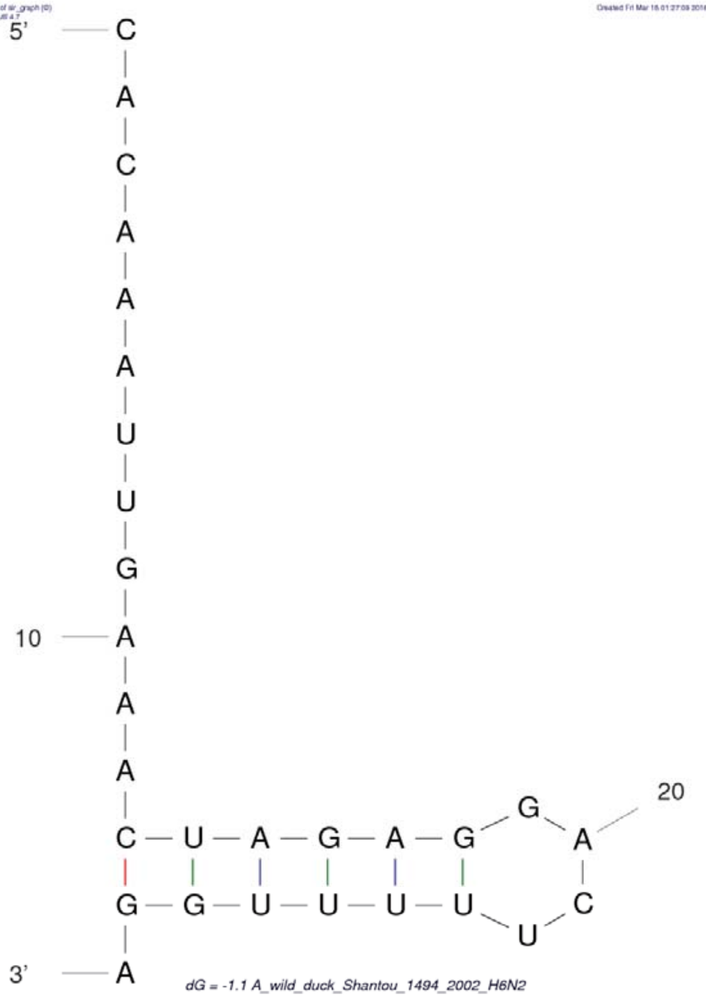

dG = -1.1 A_wild_duck_Shantou_1494_2002_H6N2

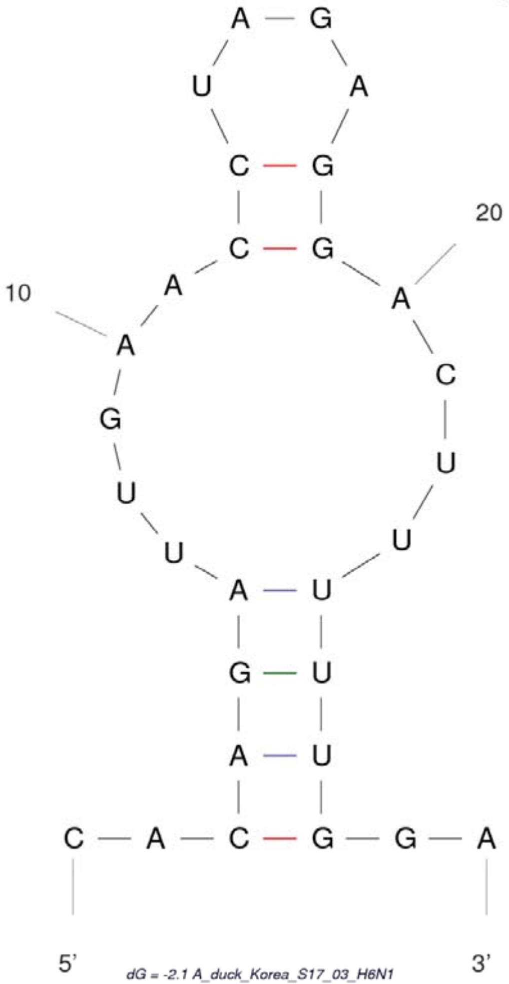

dG = -2.1 A_duck_Korea_S17_03_H6N1

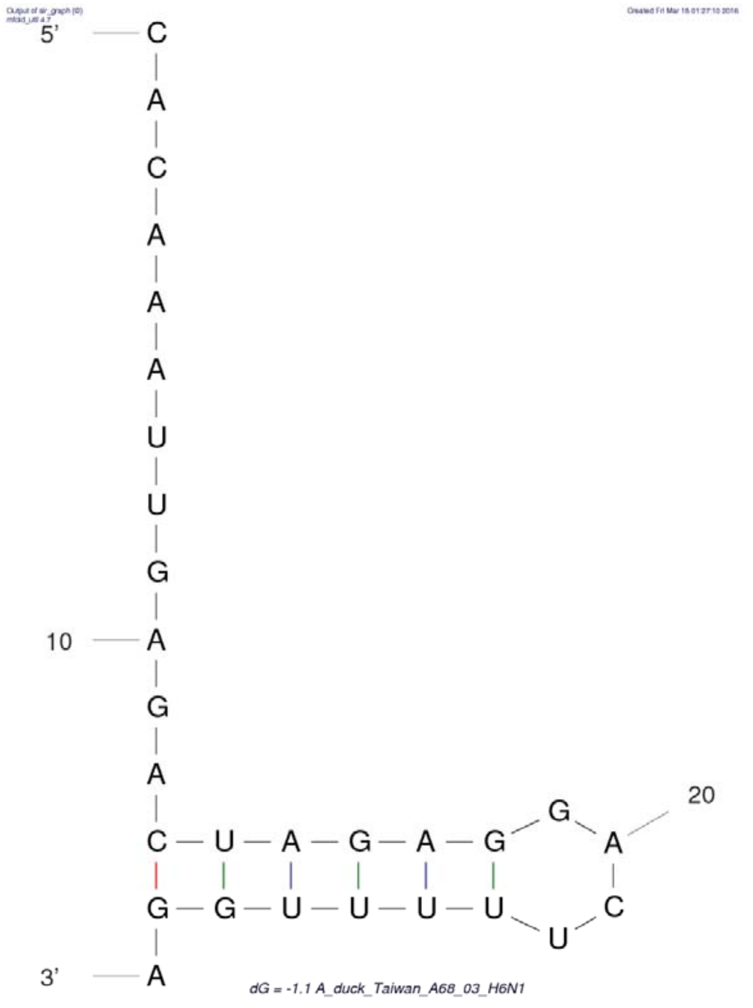
dG = -1.1 A_duck_Taiwan_A68_03_H6N1

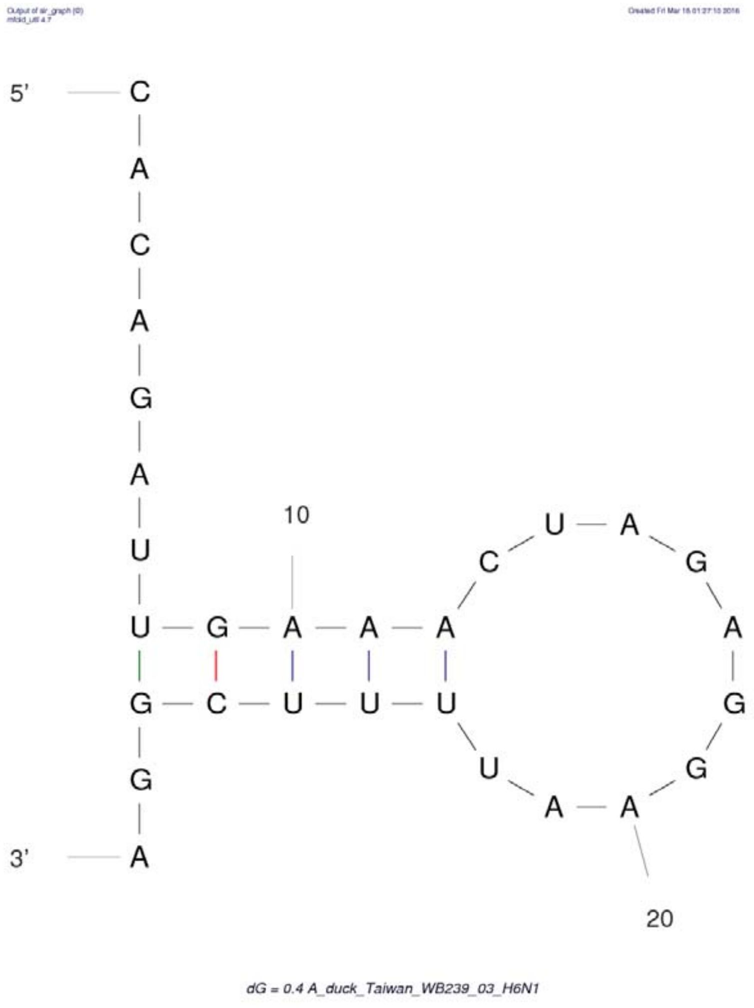
dG = 0.4 A_duck_Taiwan_WB239_03_H6N1

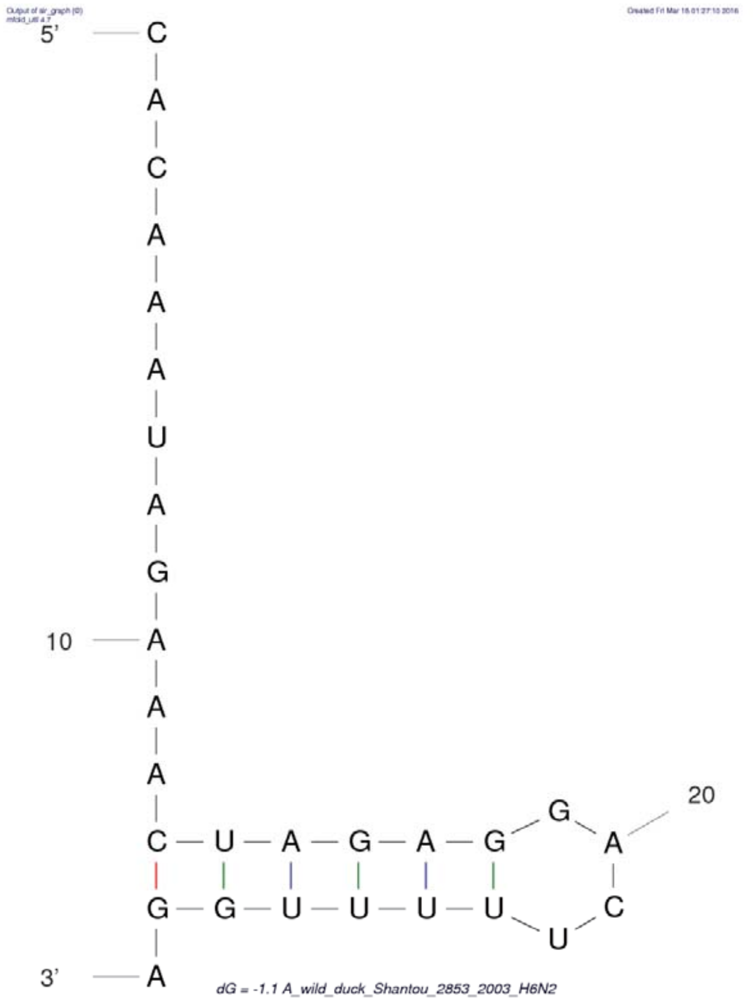
dG = -1.1 A_wild_duck_Shantou_2853_2003_H6N2

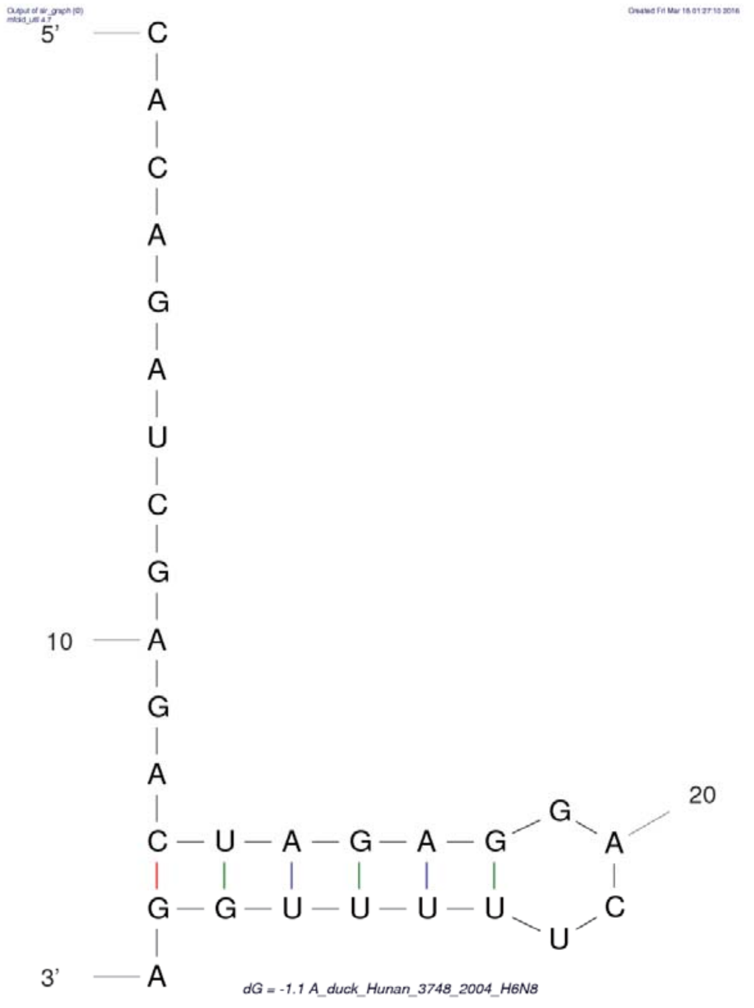
dG = -1.1 A_duck_Hunan_3748_2004_H6N8

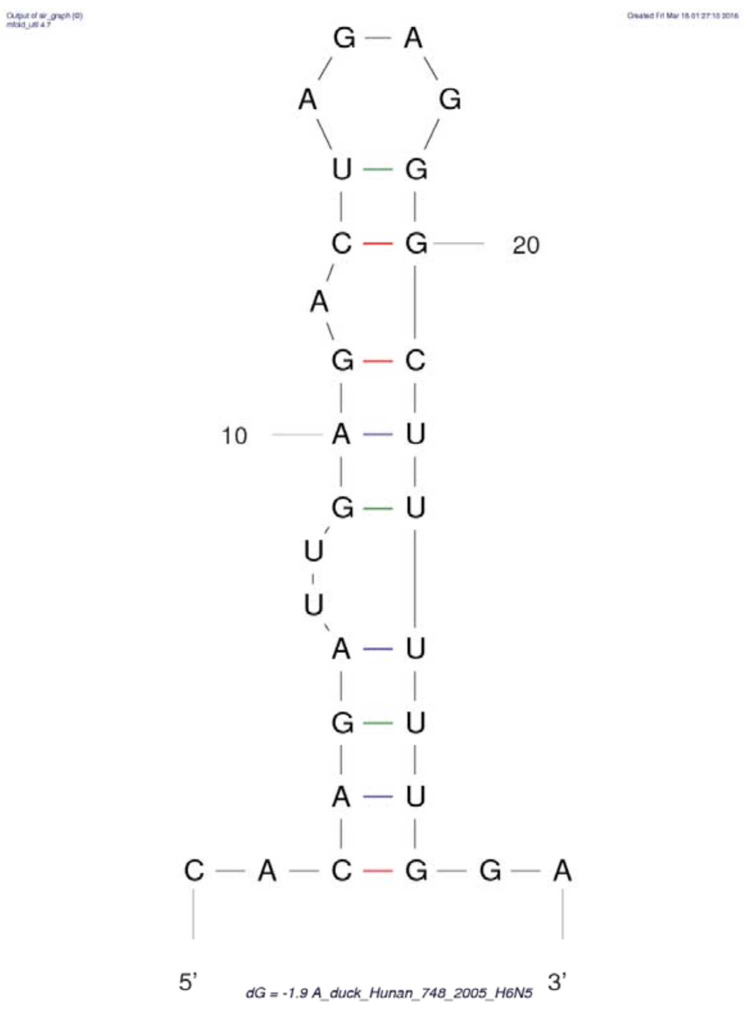

5'
3'
10
20
dG = -1.9 A_duck_Hunan_748_2005_H6N5

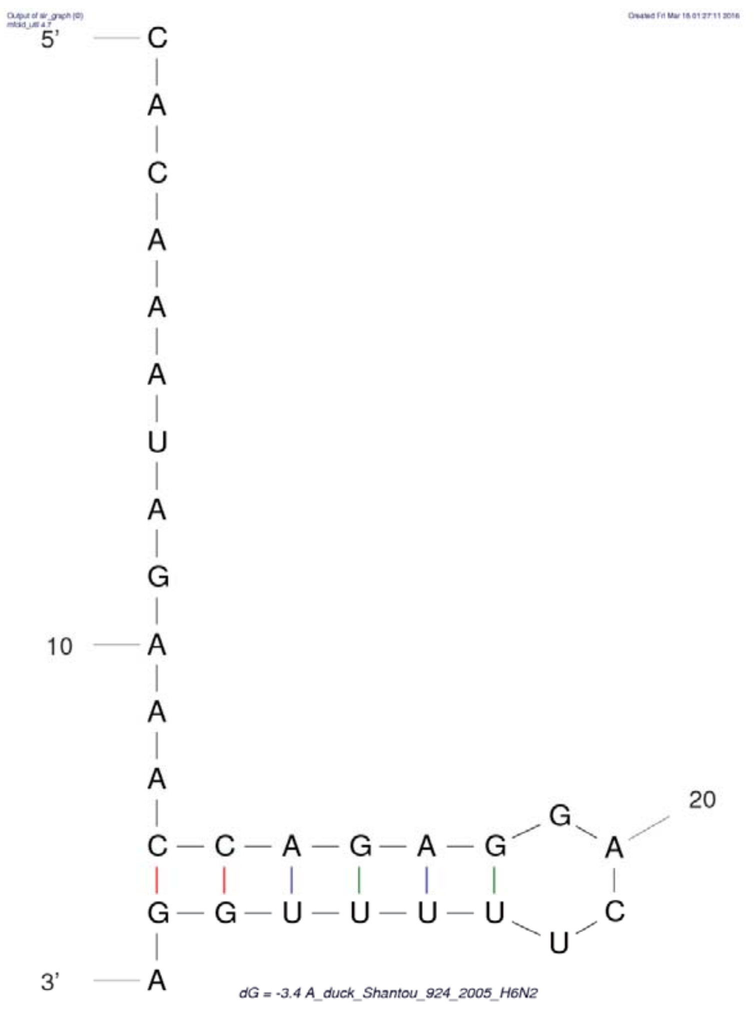

5'
3'
10
20
dG = -3.4 A_duck_Shantou_924_2005_H6N2

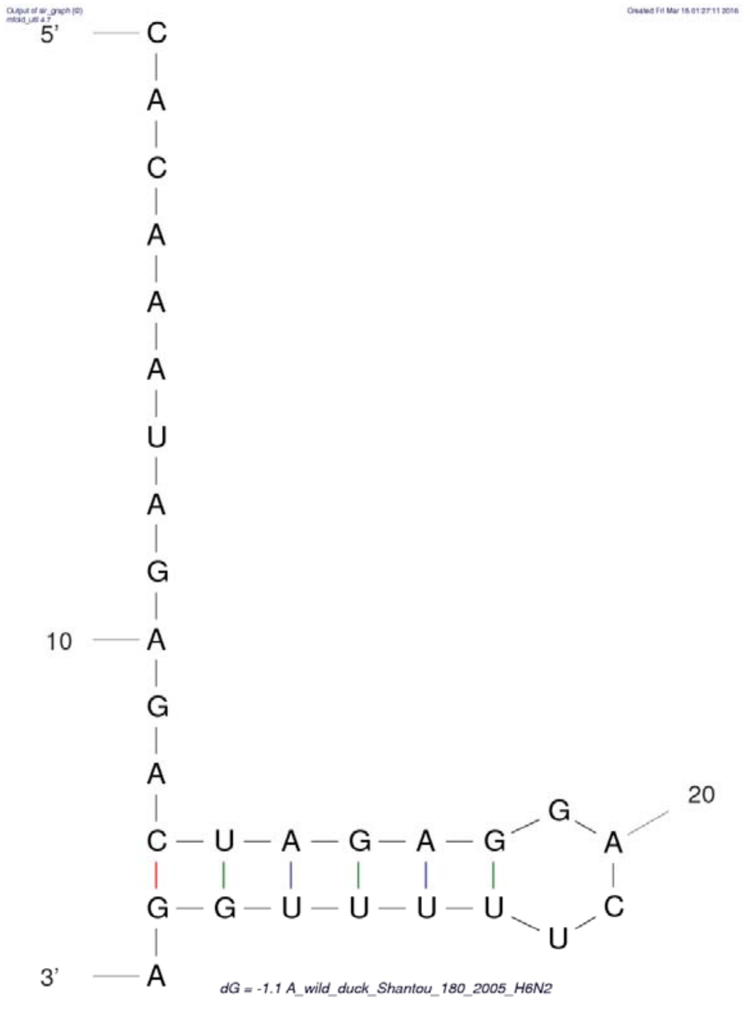

5'
3'
10
20
dG = -1.1 A_wild_duck_Shantou_180_2005_H6N2

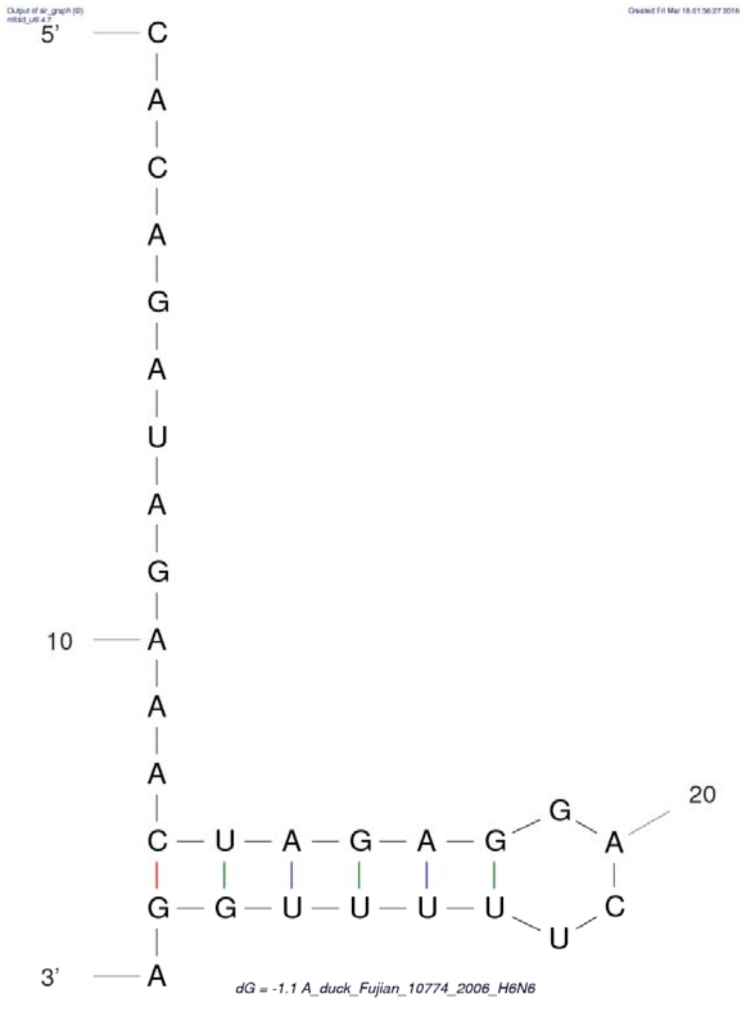

5'
3'
10
20
dG = -1.1 A_duck_Fujian_10774_2006_H6N6

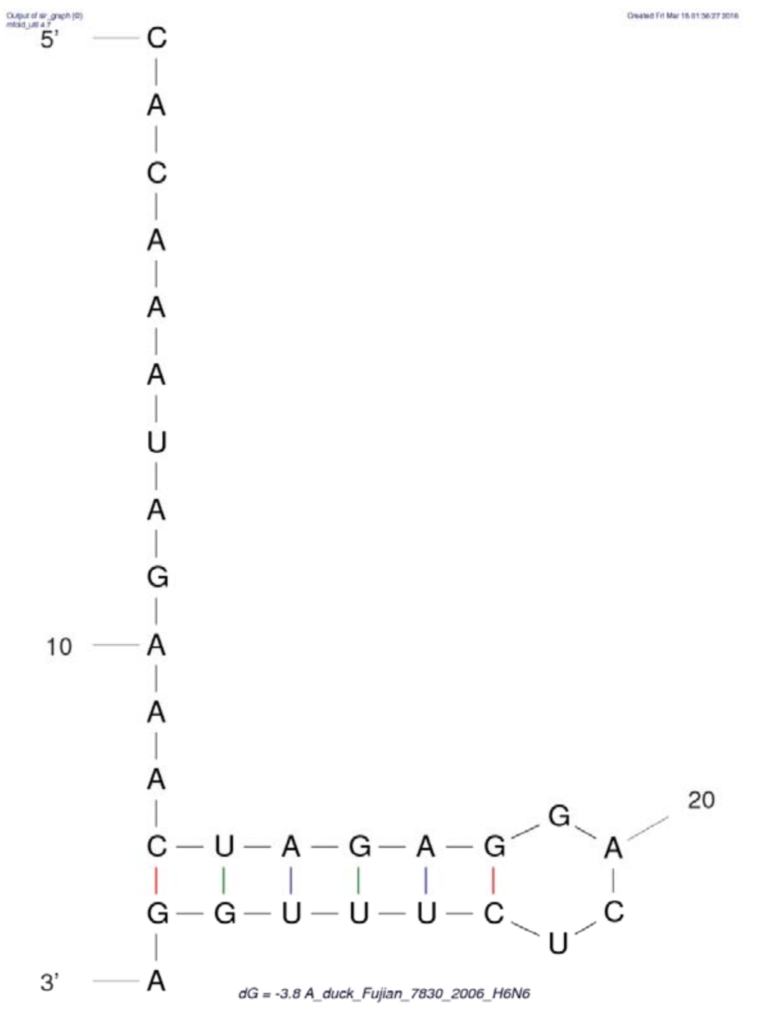
Output of sir_graph (©)
mfold_util 4.7
Created Fri Mar 18 01:56:27 2016
dG = -3.8 A_duck_Fujian_7830_2006_H6N6

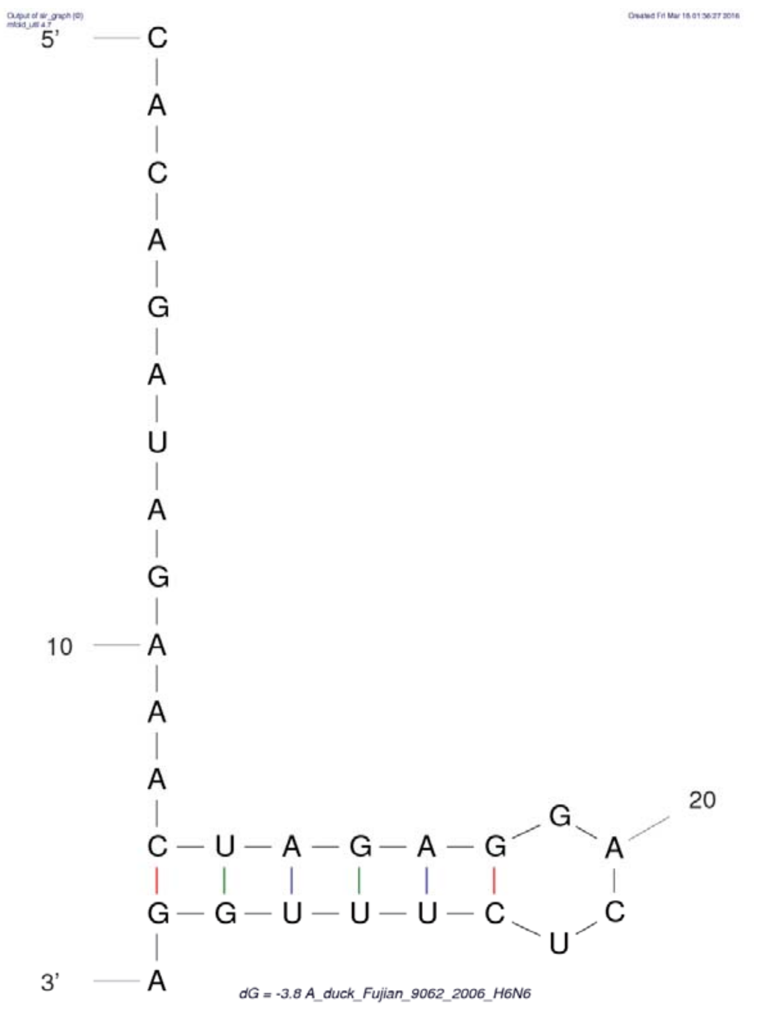
Output of sir_graph (©)
mfold_util 4.7
Created Fri Mar 18 01:56:27 2016
dG = -3.8 A_duck_Fujian_9062_2006_H6N6

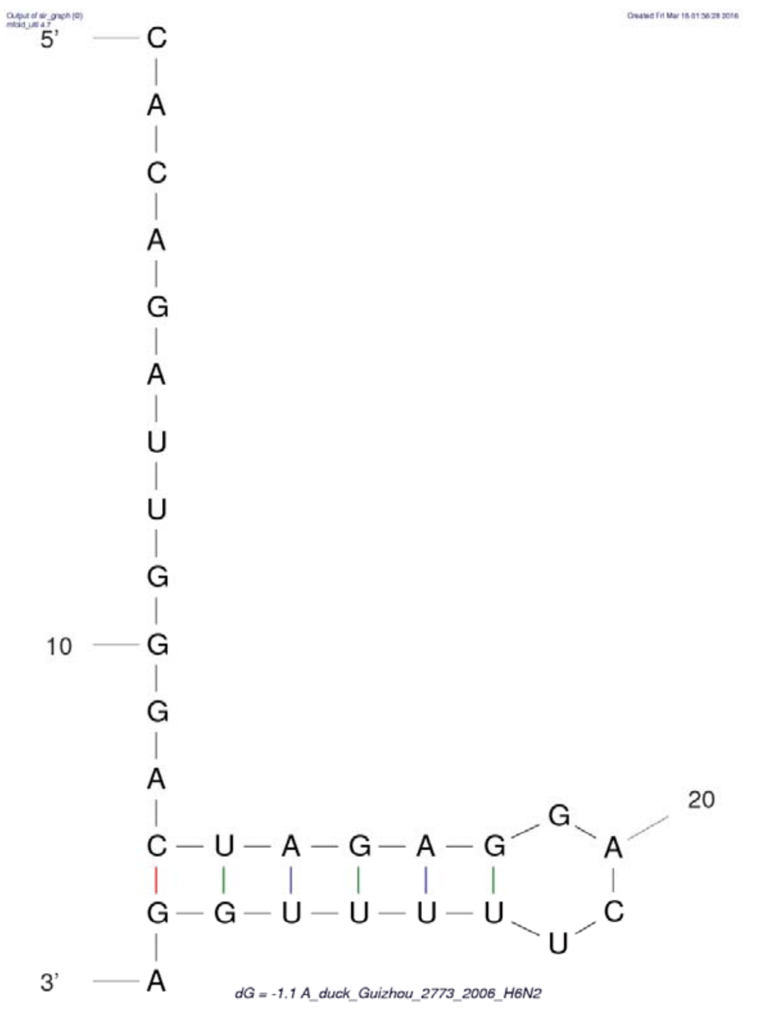
Output of sir_graph (©)
mfold_util 4.7
Created Fri Mar 18 01:56:28 2016
dG = -1.1 A_duck_Guizhou_2773_2006_H6N2

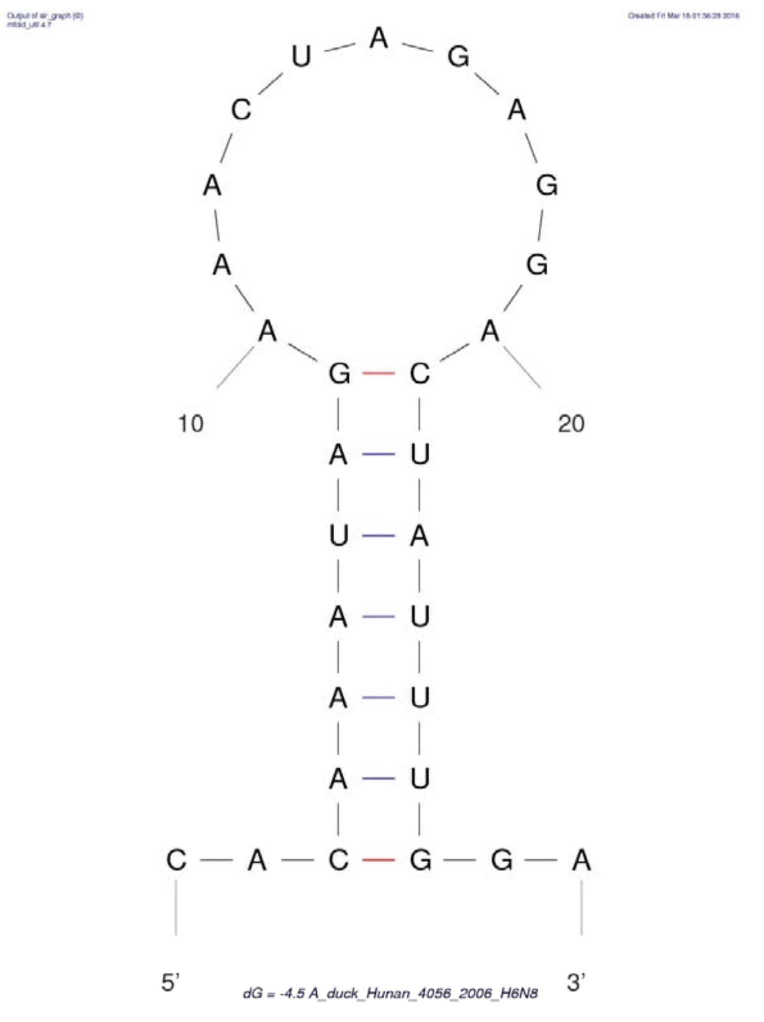
Output of sir_graph (©)
mfold_util 4.7
Created Fri Mar 18 01:56:28 2016
dG = -4.5 A_duck_Hunan_4056_2006_H6N8

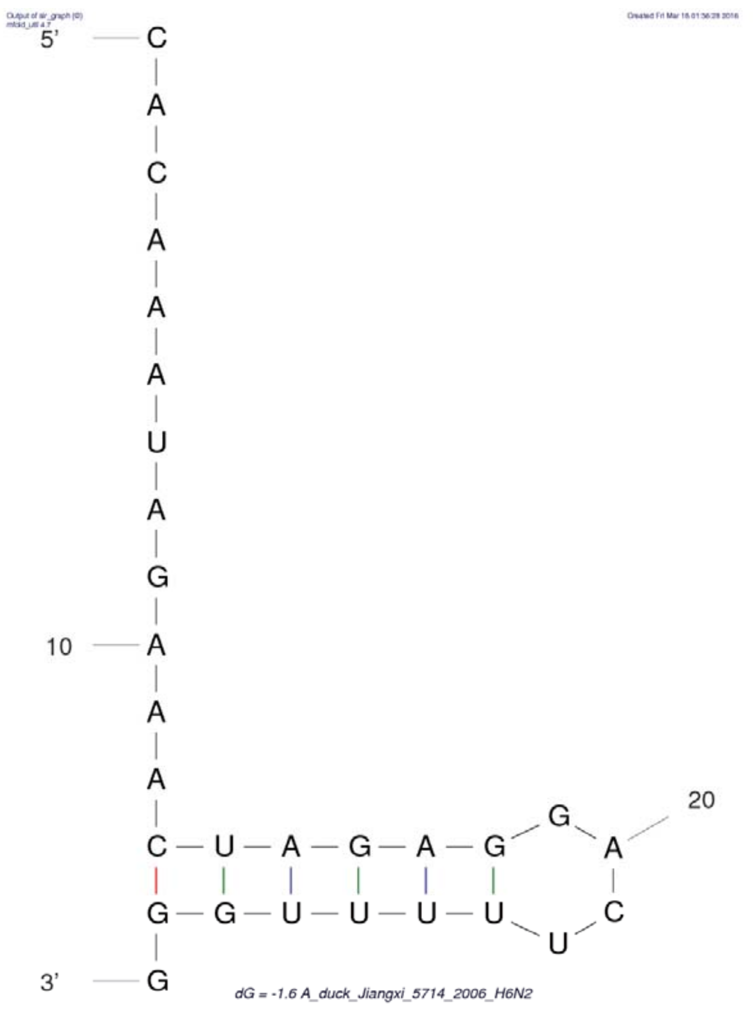
5'
10
20
3'
dG = -1.6 A_duck_Jiangxi_5714_2006_H6N2

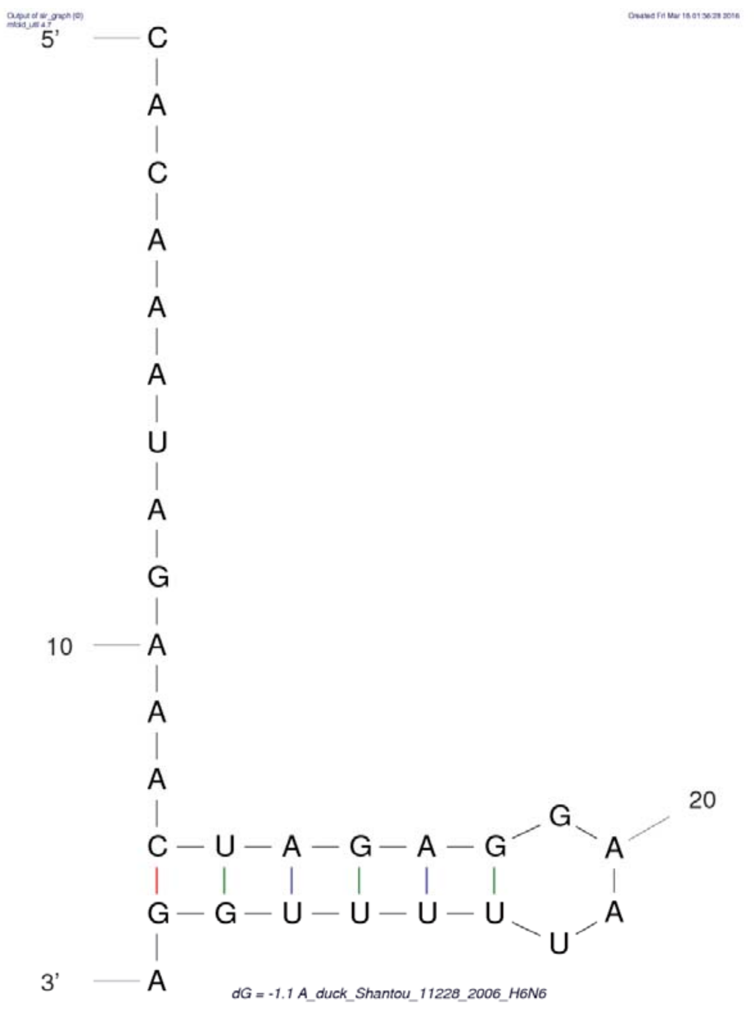
5'
10
20
3'
dG = -1.1 A_duck_Shantou_11228_2006_H6N6

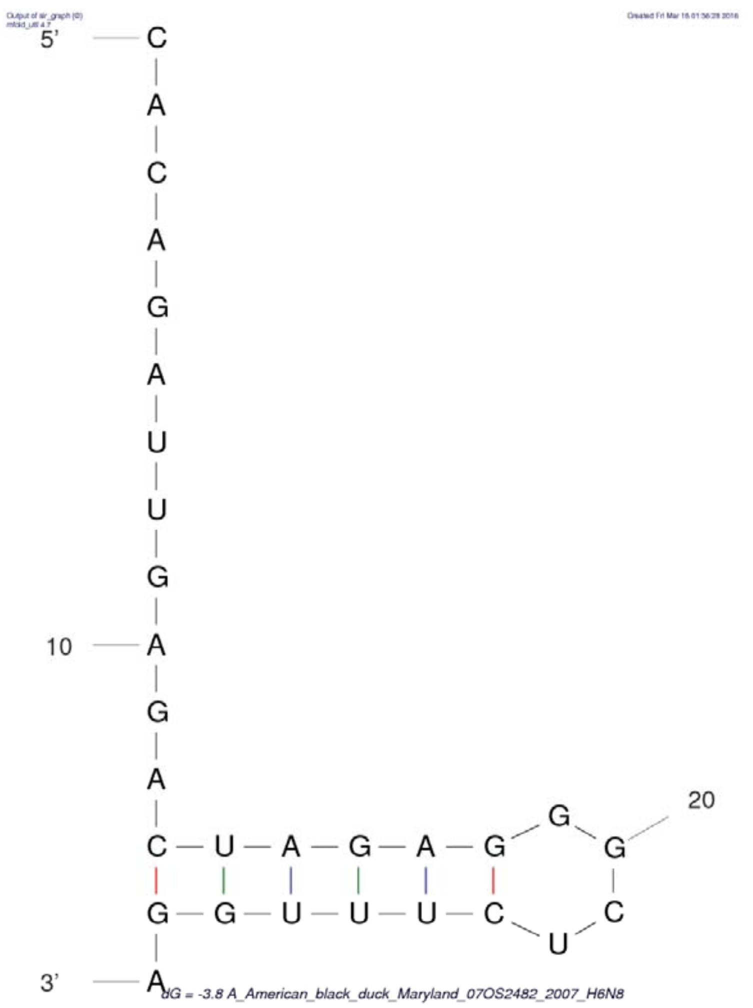
5'
10
20
3'
dG = -3.8 A_American_black_duck_Maryland_07OS2482_2007_H6N8

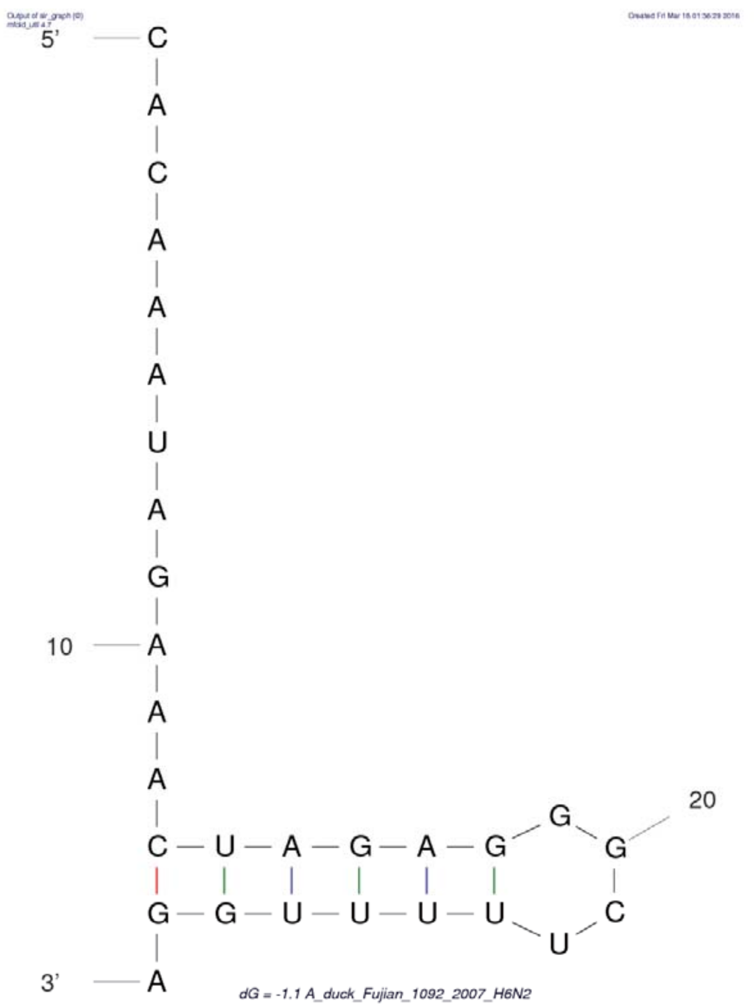
5'
10
20
3'
dG = -1.1 A_duck_Fujian_1092_2007_H6N2

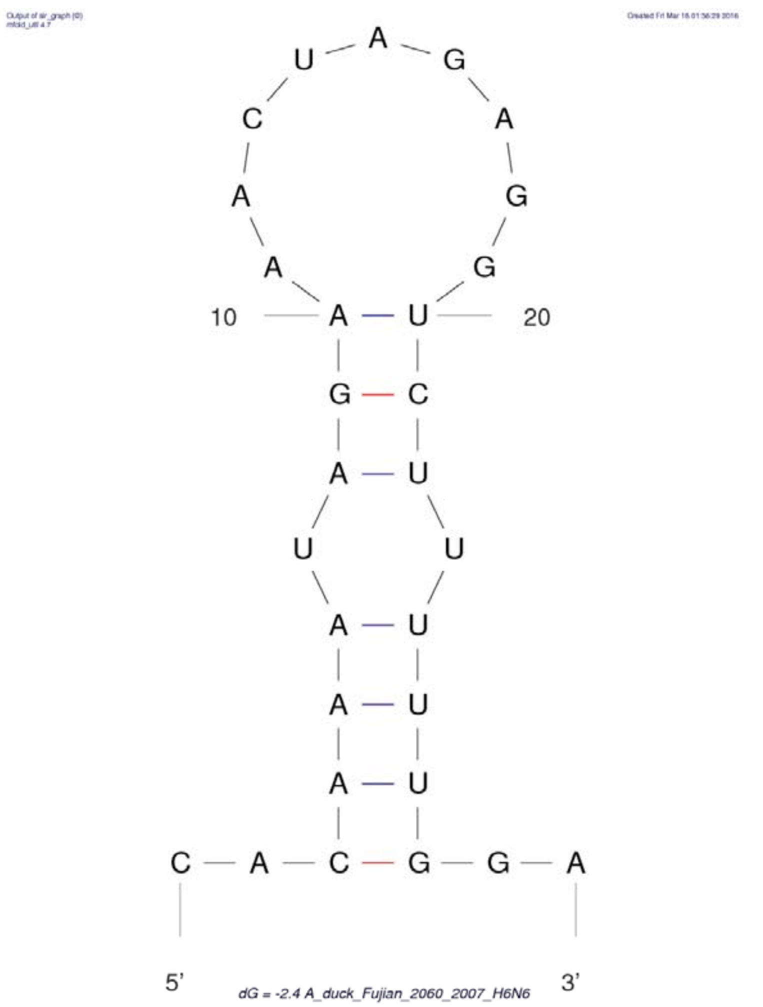

dG = -2.4 A_duck_Fujian_2060_2007_H6N6

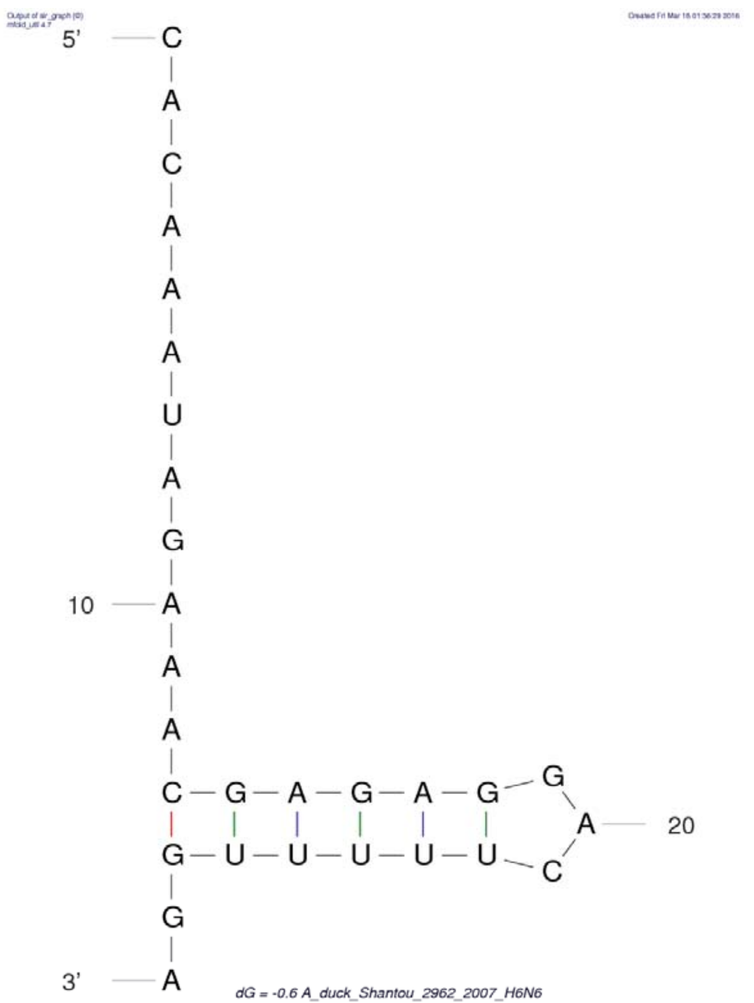

dG = -0.6 A_duck_Shantou_2962_2007_H6N6

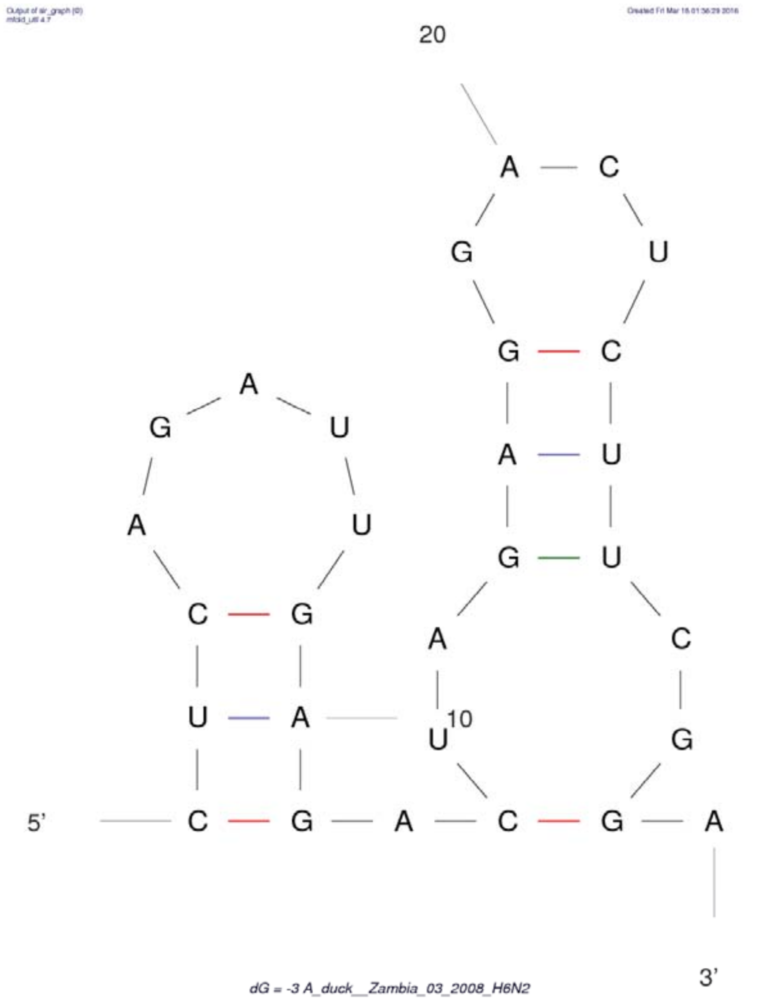

dG = -3 A_duck__Zambia_03_2008_H6N2

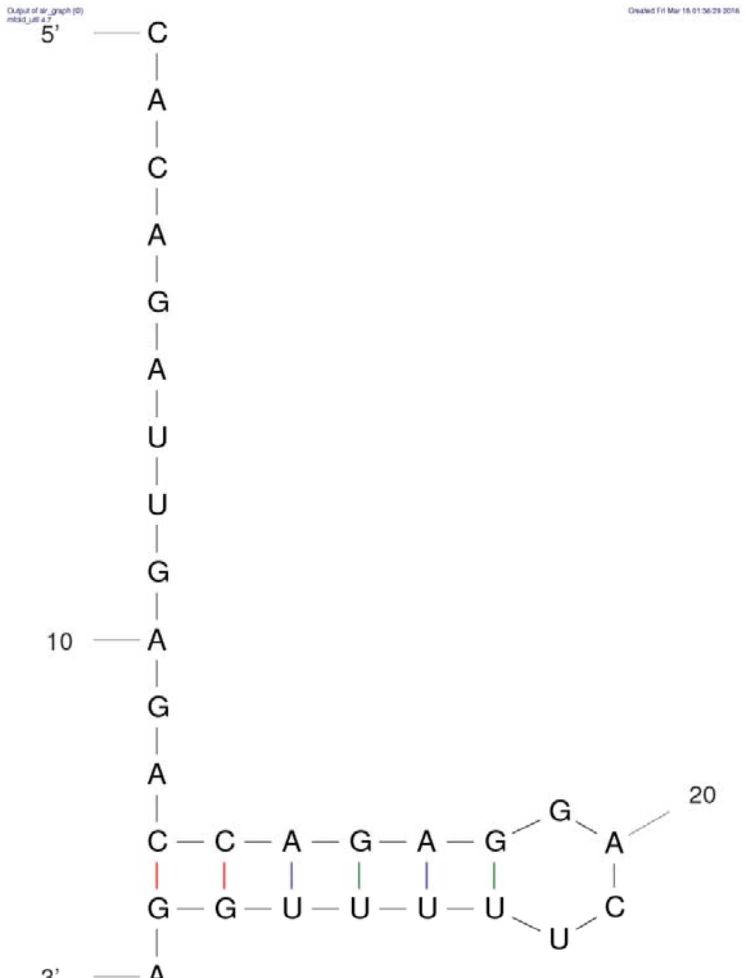

dG = -3.4 A_spot-billed_duck_Korea_528_2008_H6N8

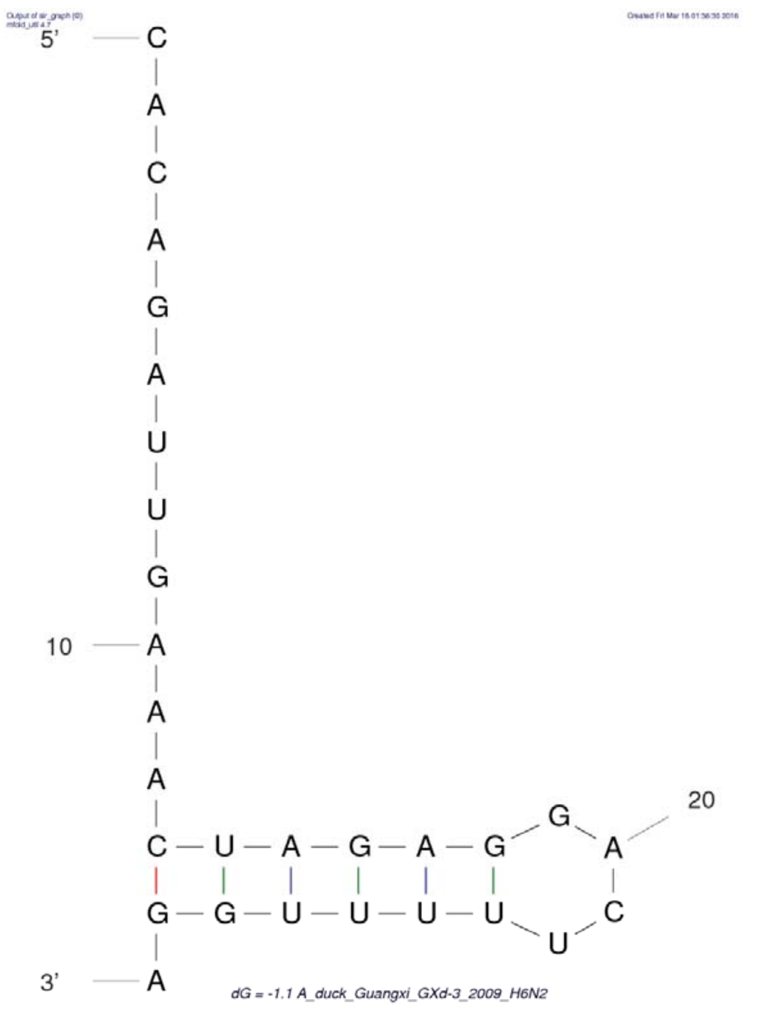
dG = -1.1 A_duck_Guangxi_GXd-3_2009_H6N2

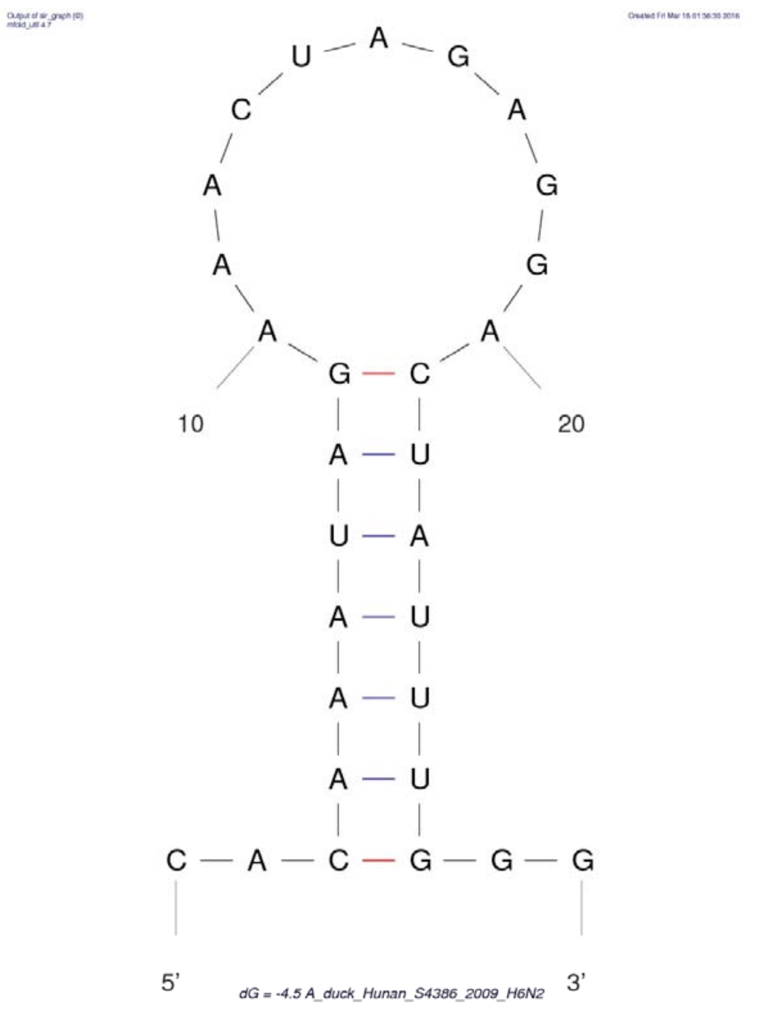
dG = -4.5 A_duck_Hunan_S4386_2009_H6N2

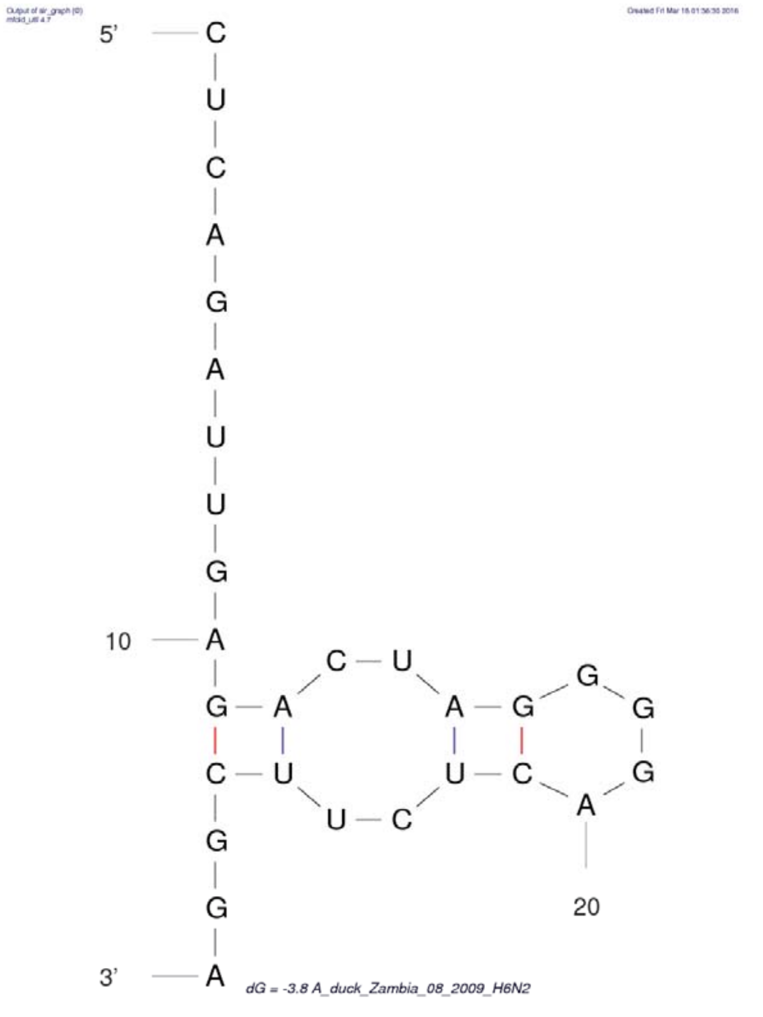
dG = -3.8 A_duck_Zambia_08_2009_H6N2

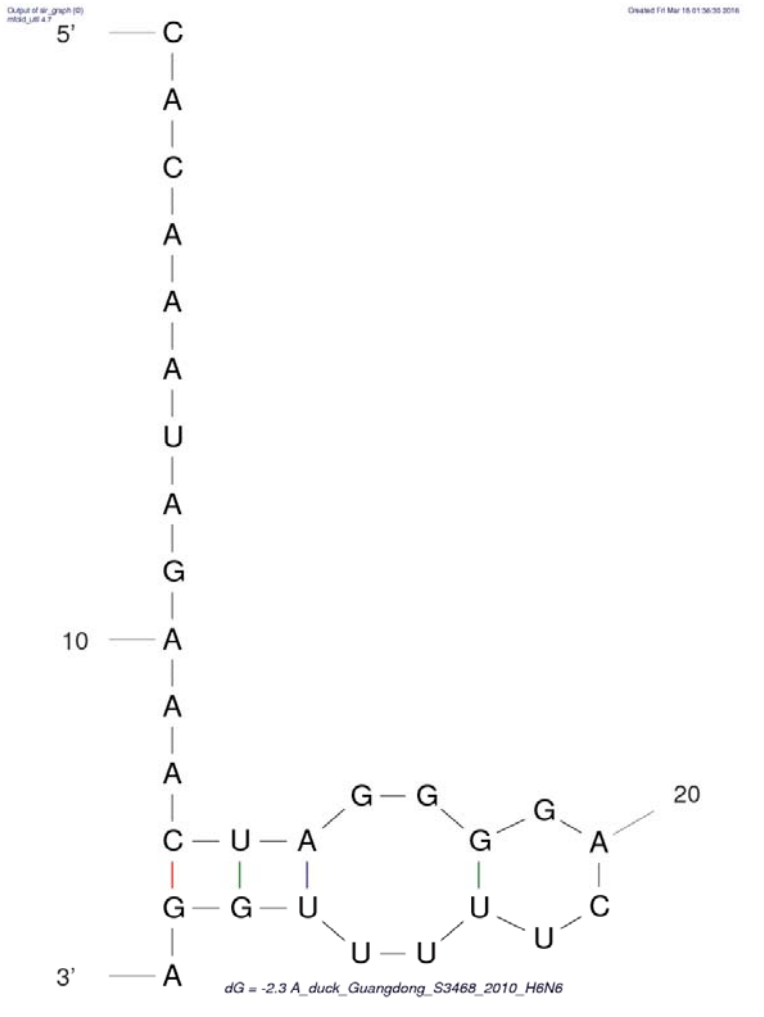
dG = -2.3 A_duck_Guangdong_S3468_2010_H6N6

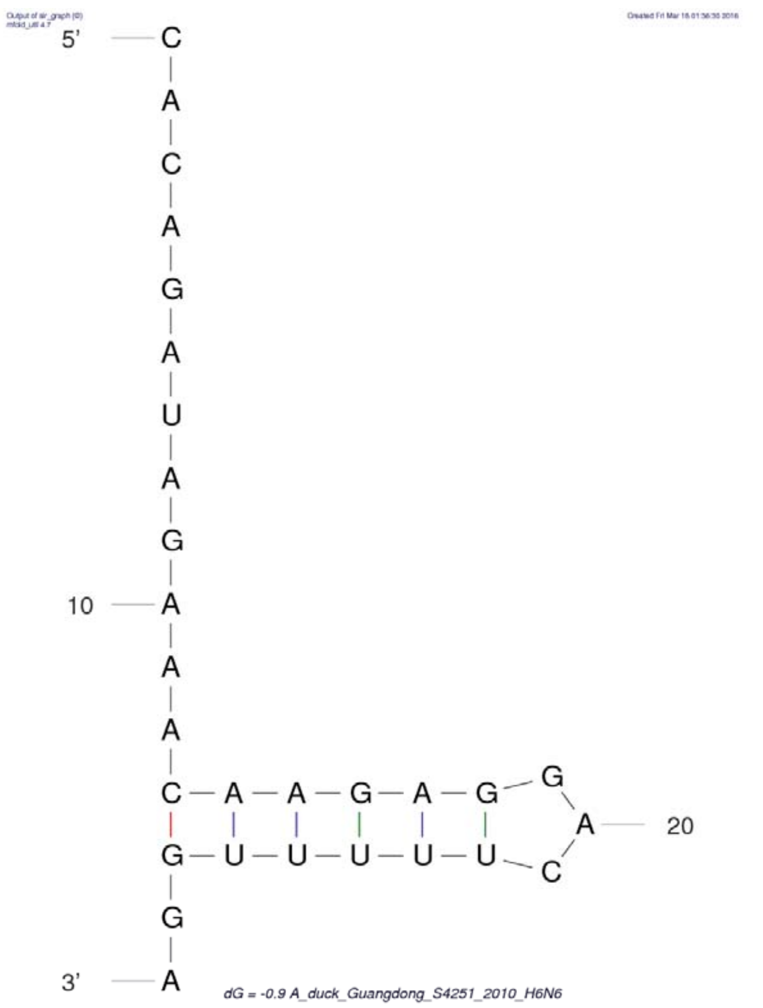
Output of sir_graph (©)
mfold_util 4.7
Created Fri Mar 18 01:36:30 2016
5'
10
20
3'
dG = -0.9 A_duck_Guangdong_S4251_2010_H6N6

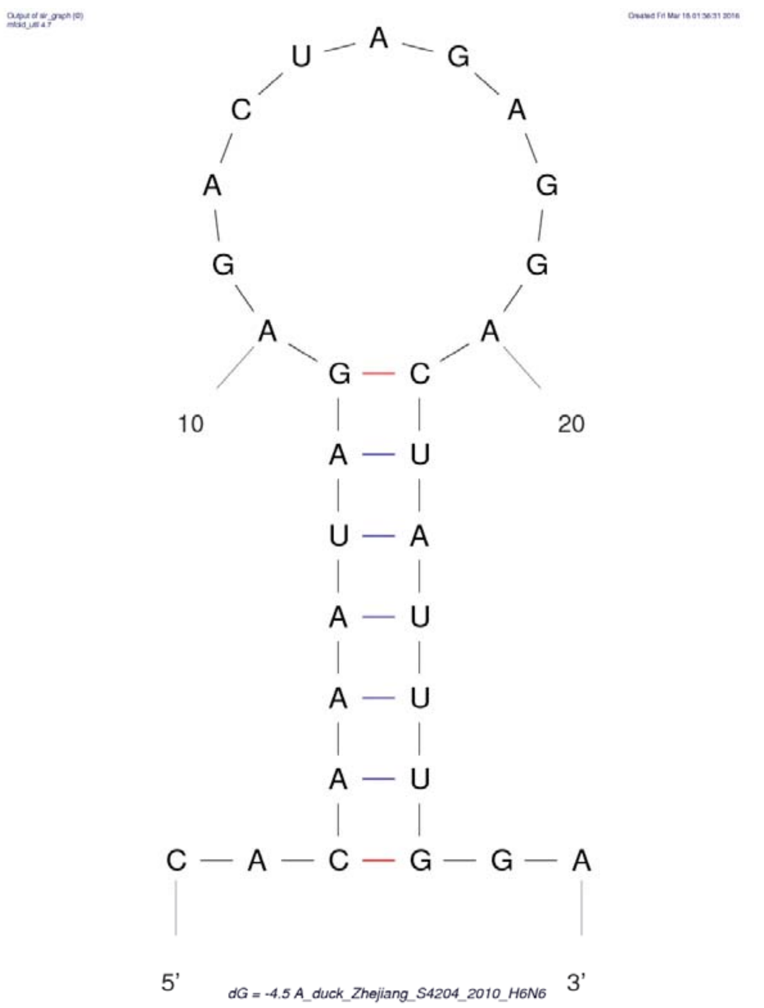
Output of sir_graph (©)
mfold_util 4.7
Created Fri Mar 18 01:36:31 2016
10
20
5'
3'
dG = -4.5 A_duck_Zhejiang_S4204_2010_H6N6

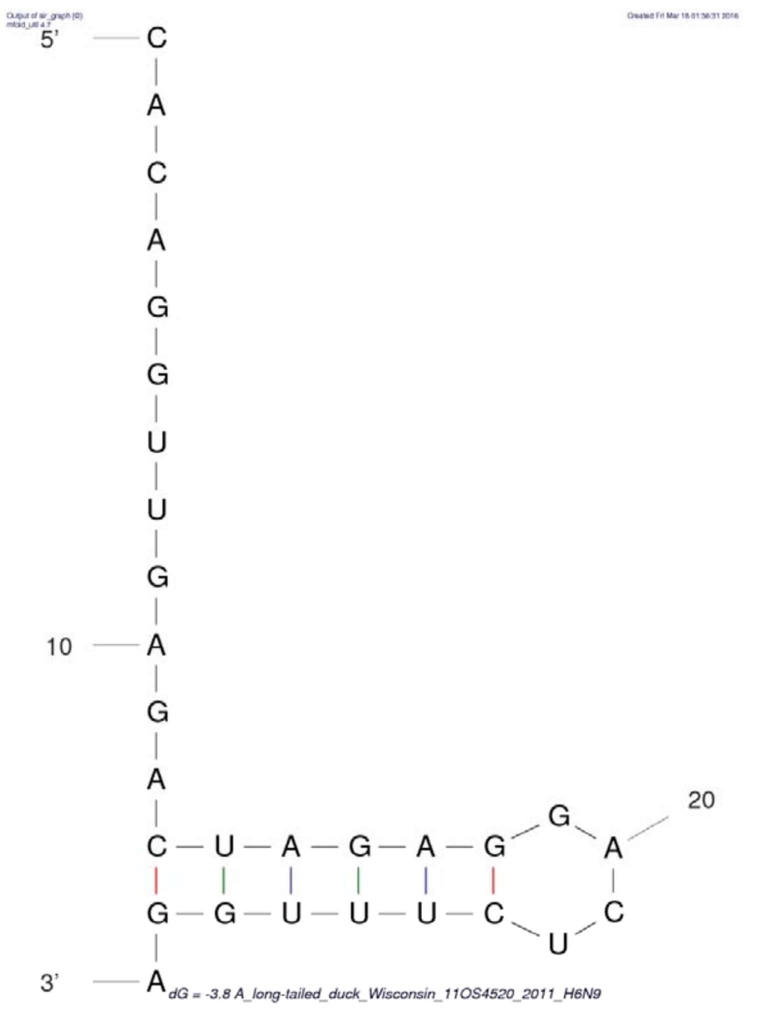
Output of sir_graph (©)
mfold_util 4.7
Created Fri Mar 18 01:36:31 2016
5'
10
20
3'
dG = -3.8 A_long-tailed_duck_Wisconsin_11OS4520_2011_H6N9

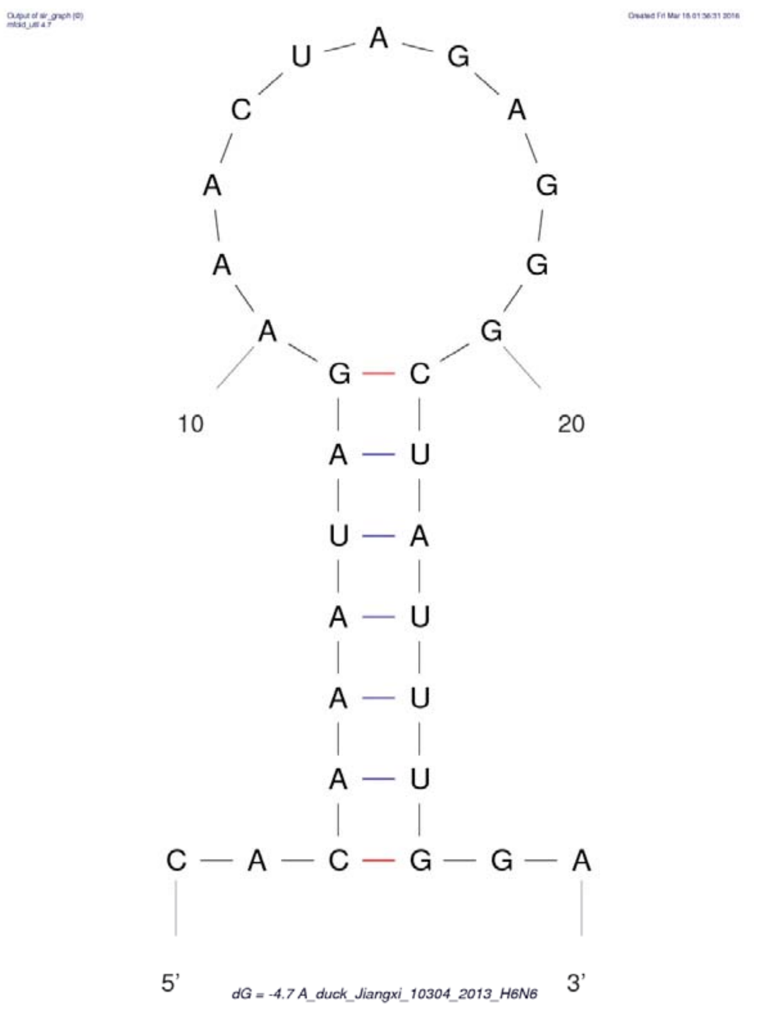
Output of sir_graph (©)
mfold_util 4.7
Created Fri Mar 18 01:36:31 2016
10
20
5'
3'
dG = -4.7 A_duck_Jiangxi_10304_2013_H6N6

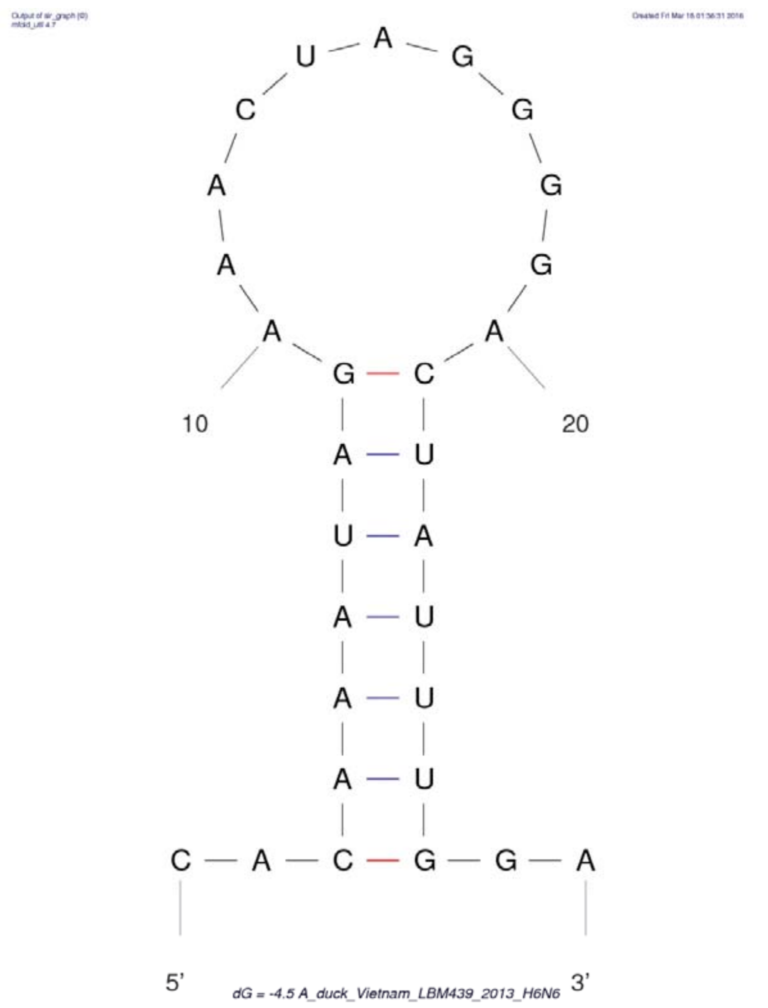
Output of sir_graph (©)
mfold_util 4.7
Created Fri Mar 18 01:36:31 2016
10
20
5'
3'
dG = -4.5 A_duck_Vietnam_LBM439_2013_H6N6

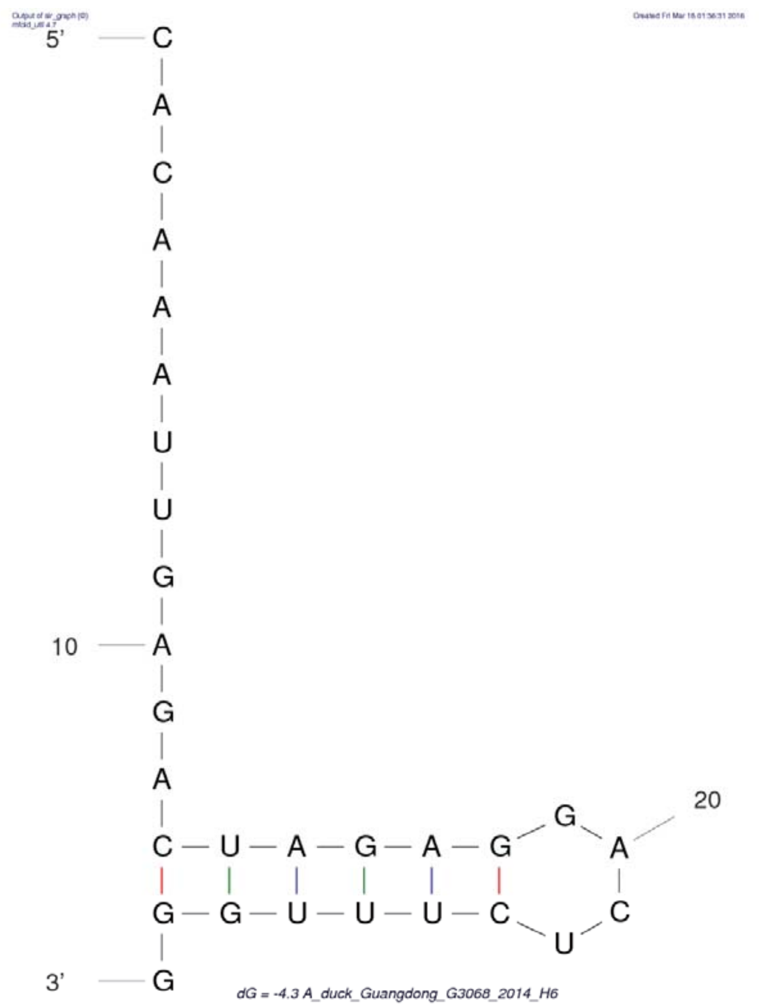
Output of sir_graph (©)
mfold_util 4.7
Created Fri Mar 18 01:36:31 2016
5'
10
20
3'
dG = -4.3 A_duck_Guangdong_G3068_2014_H6

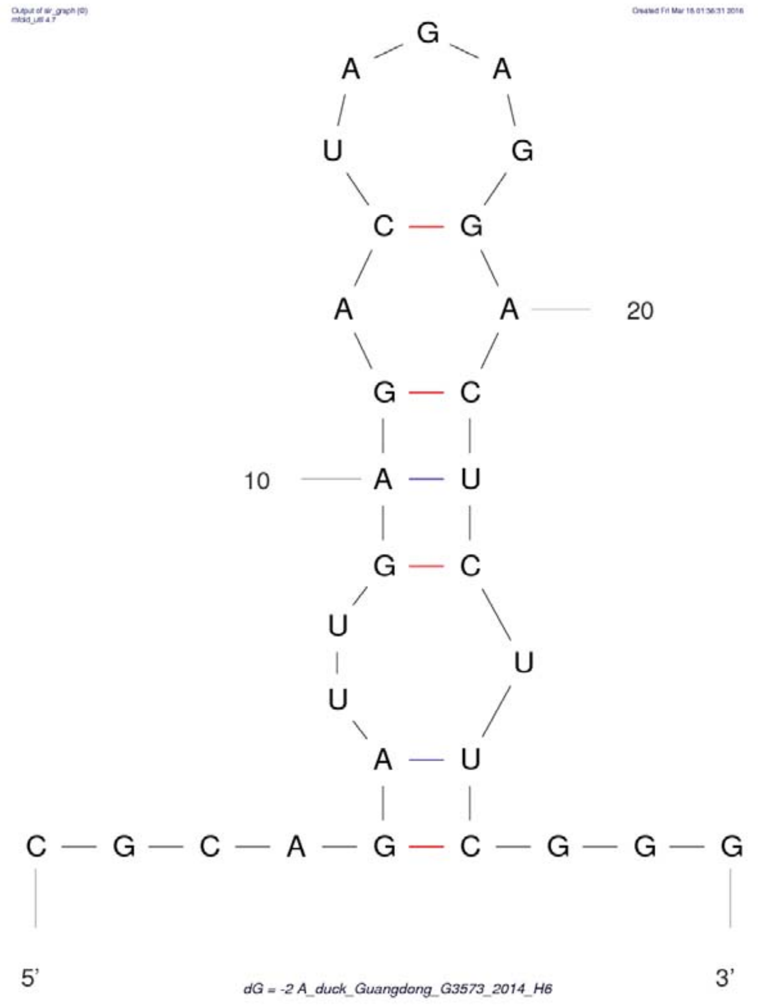
Output of sir_graph (©)
mfold_util 4.7
Created Fri Mar 18 01:36:31 2016
20
10
5'
3'
dG = -2 A_duck_Guangdong_G3573_2014_H6

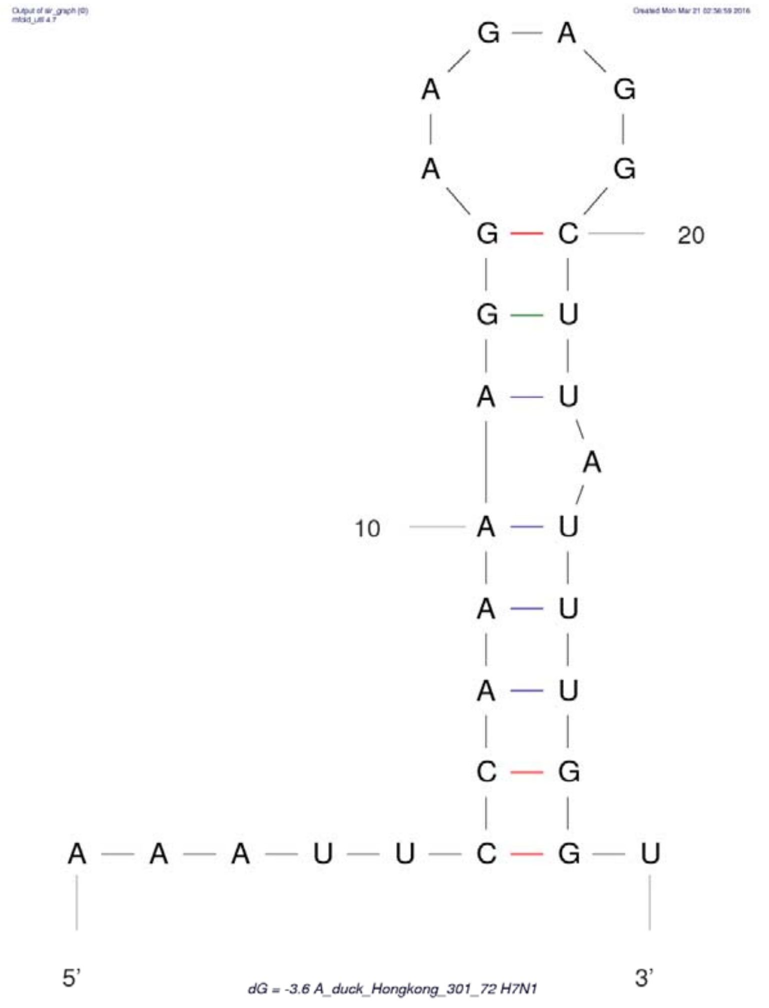
Output of sir_graph (©)
mfold_util 4.7
Created Mon Mar 21 02:56:59 2016
20
10
5'
3'
dG = -3.6 A_duck_Hongkong_301_72 H7N1

Output of sir_graph (©)
mfold_util 4.7

Created Mon Mar 21 02:56:59 2016

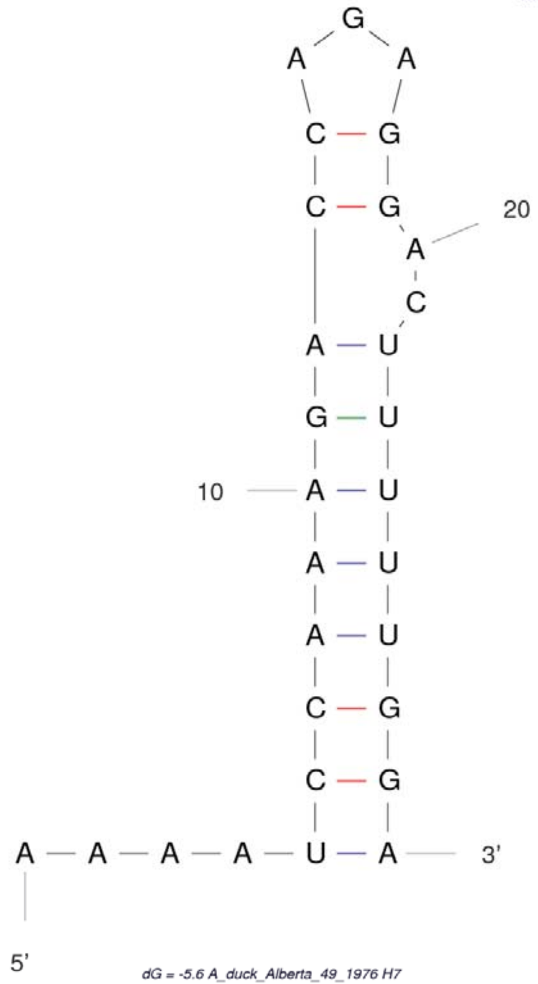

dG = -5.6 A_duck_Alberta_49_1976 H7

Output of sir_graph (©)
mfold_util 4.7

Created Mon Mar 21 02:56:59 2016

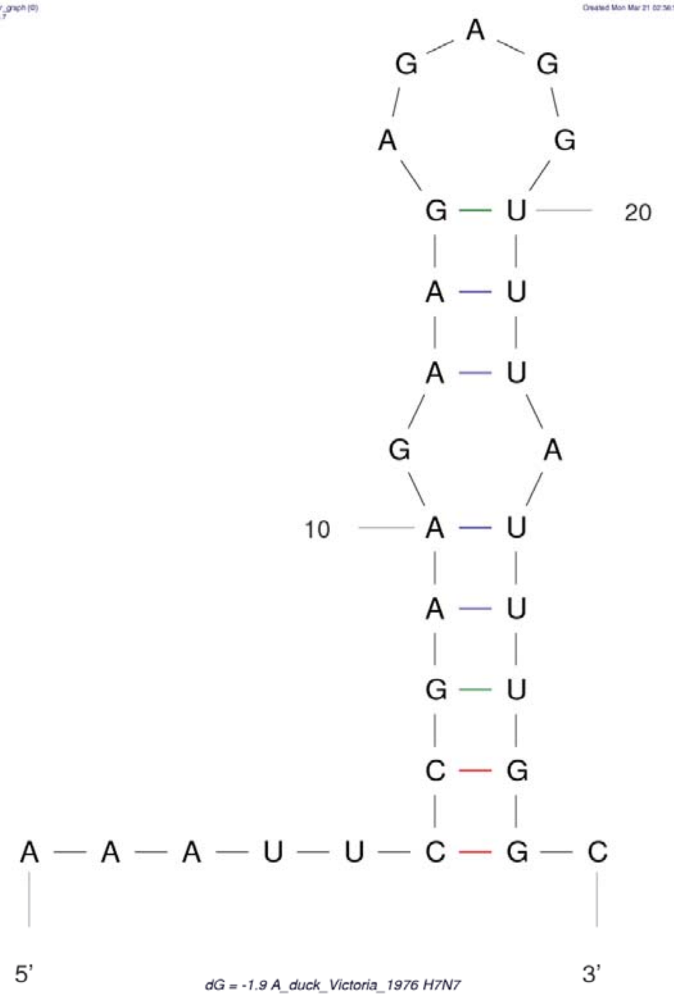

dG = -1.9 A_duck_Victoria_1976 H7N7

Output of sir_graph (©)
mfold_util 4.7

Created Mon Mar 21 02:57:00 2016

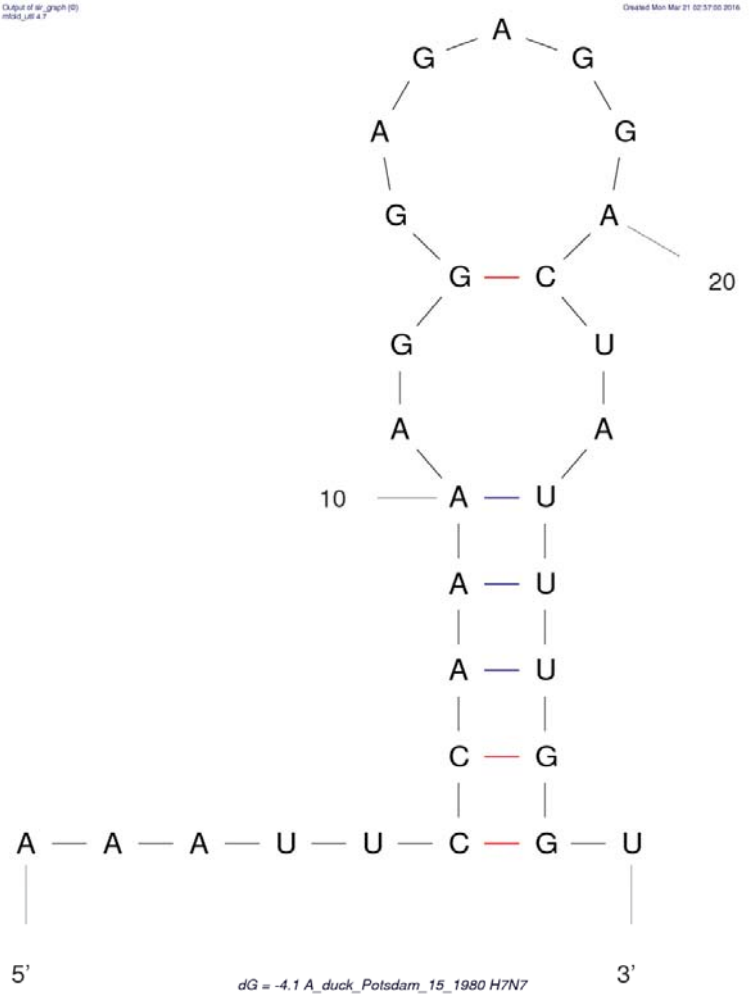

dG = -4.1 A_duck_Potsdam_15_1980 H7N7

Output of sir_graph (©)
mfold_util 4.7

Created Mon Mar 21 02:57:00 2016

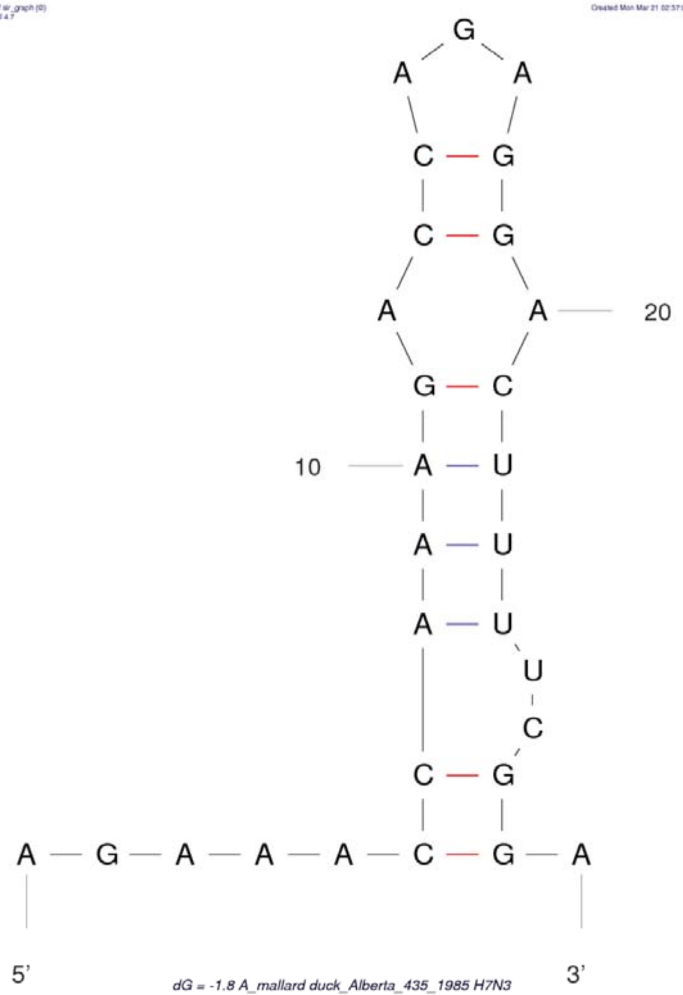

dG = -1.8 A_mallard duck_Alberta_435_1985 H7N3

Output of sir_graph (©)
mfold_util 4.7

Created Mon Mar 21 02:57:00 2016

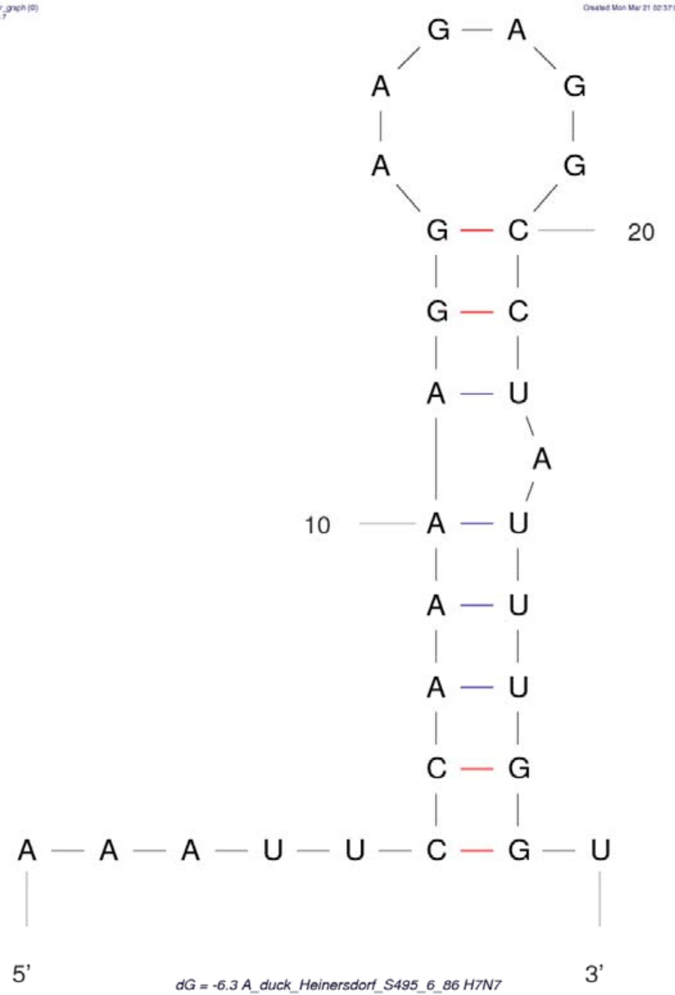

dG = -6.3 A_duck_Heinersdorf_S495_6_86 H7N7

Output of sir_graph (©)
mfold_util 4.7

Created Mon Mar 21 02:57:00 2016

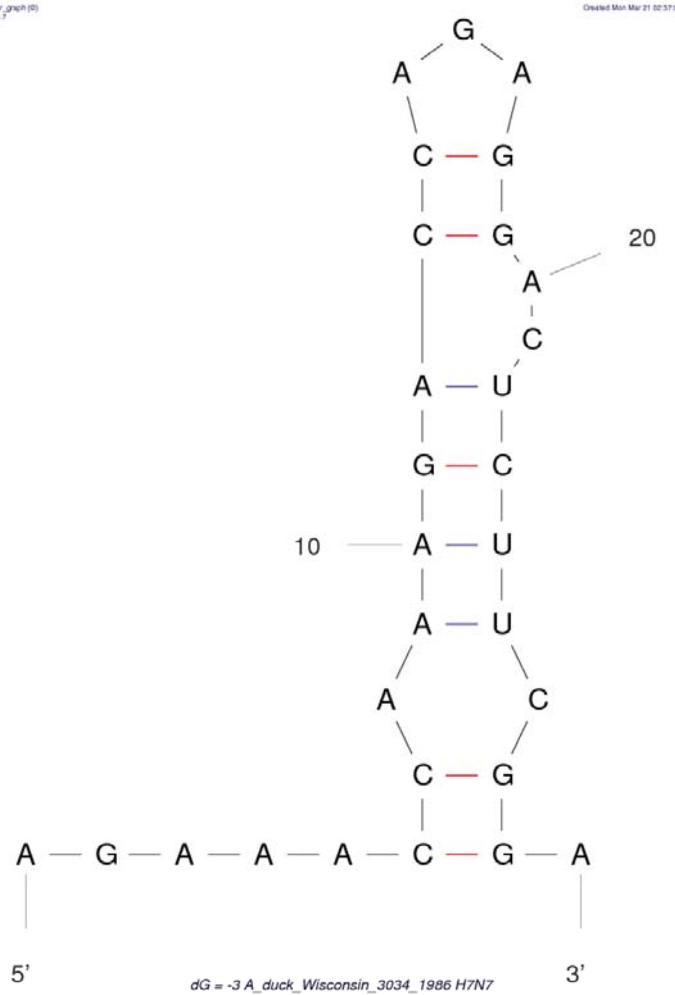

dG = -3 A_duck_Wisconsin_3034_1986 H7N7

Output of sir_graph (©)
mfold_util 4.7

Created Mon Mar 21 02:57:00 2016

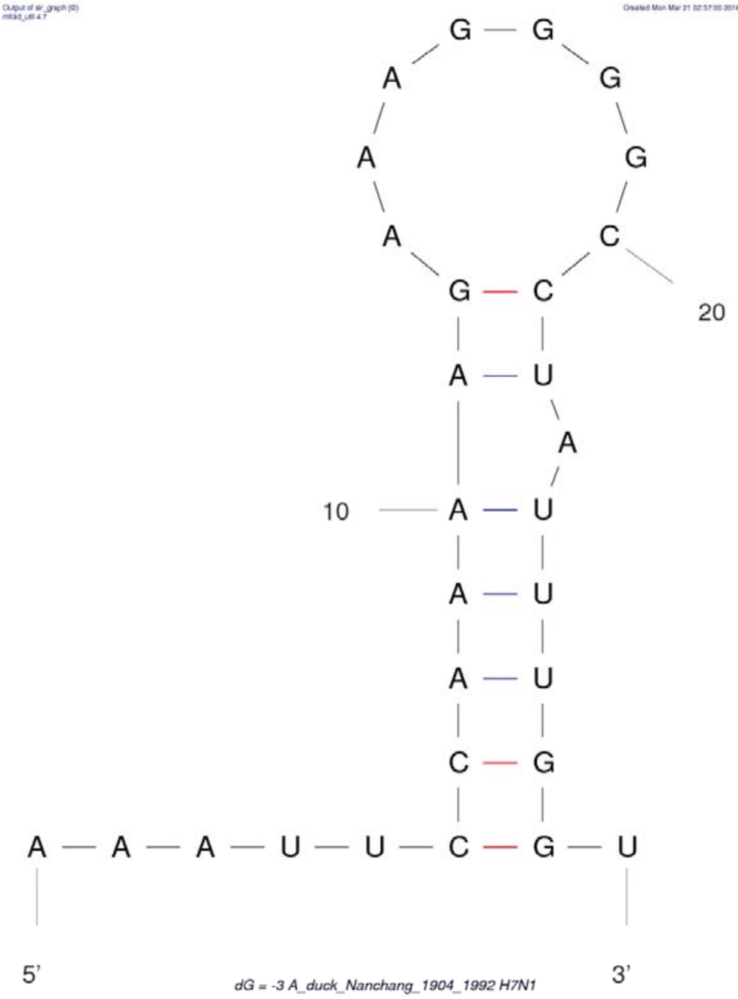

dG = -3 A_duck_Nanchang_1904_1992 H7N1

Output of sir_graph (©)
mfold_util 4.7

Created Mon Mar 21 02:57:01 2016

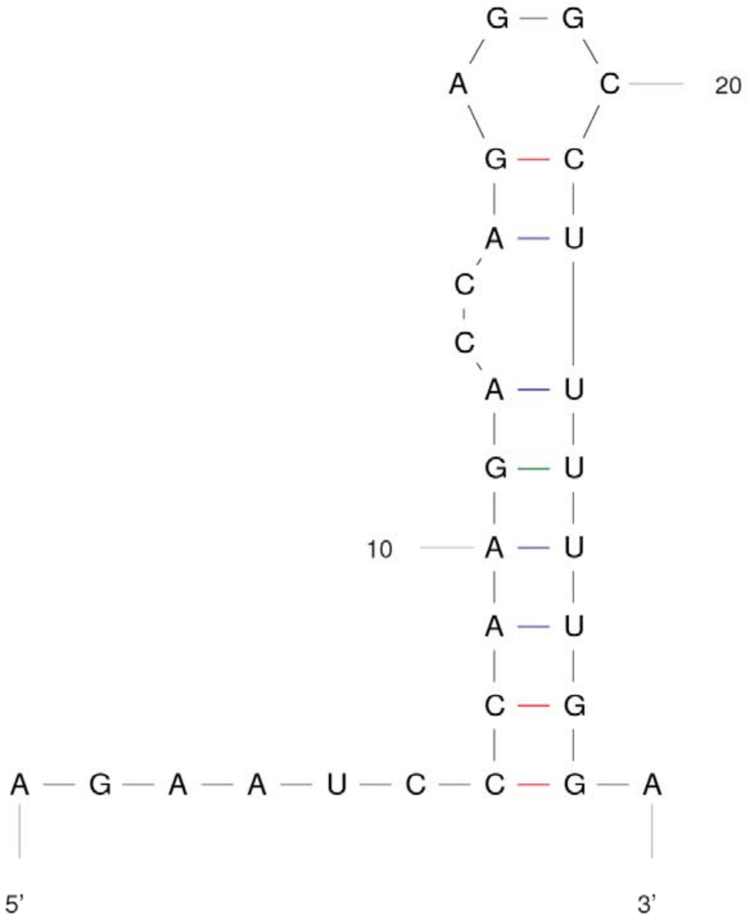

dG = -3.9 A_duck_Alaska_3111_1993 H7N3

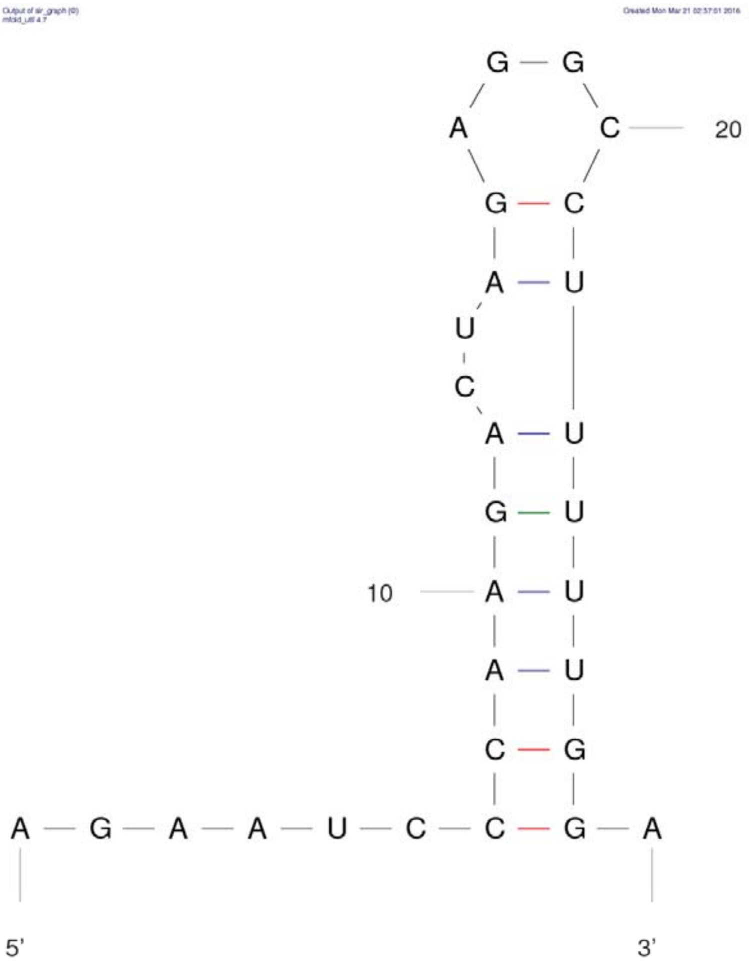

dG = -3.9 A_duck_Guangdong_1_1996 H7N3

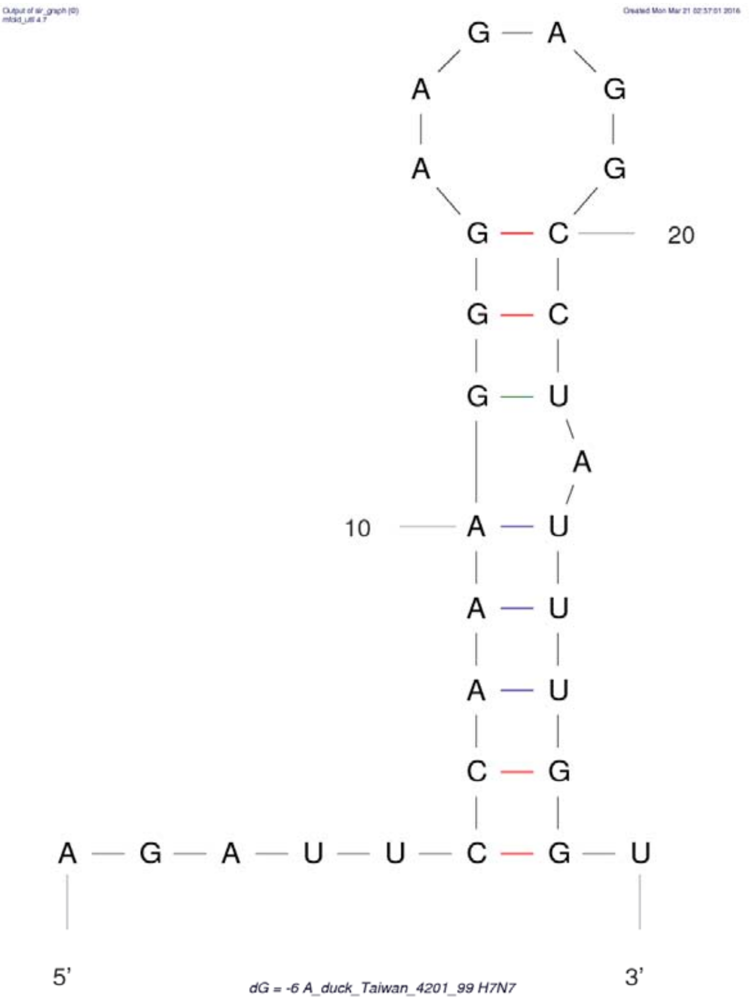

dG = -6 A_duck_Taiwan_4201_99 H7N7

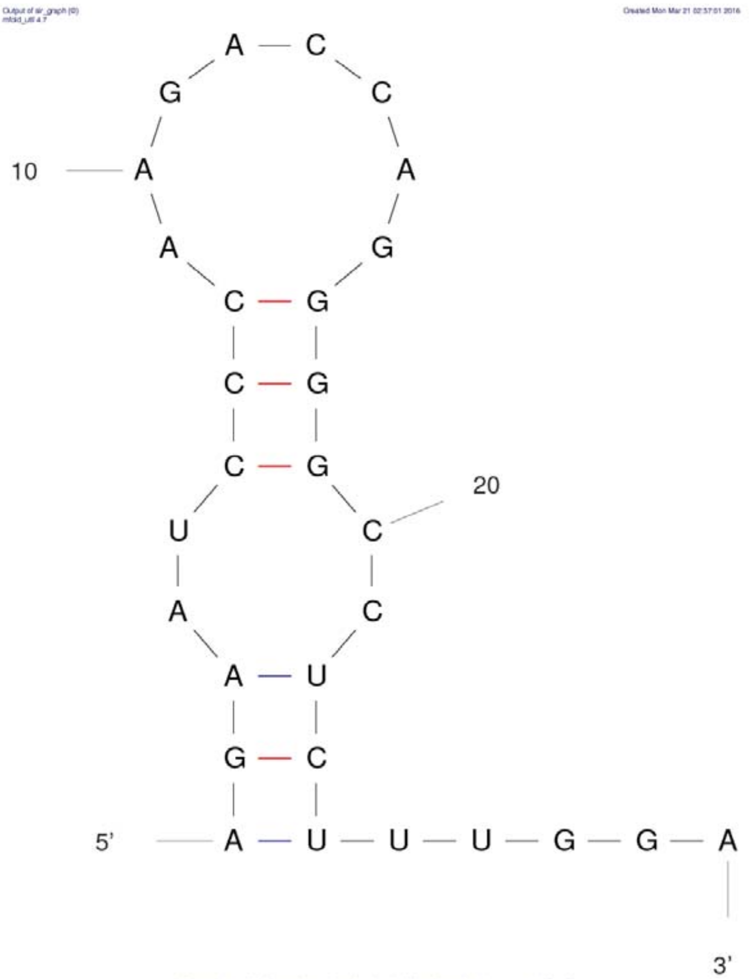

dG = -4.7 A_American black duck_Maryland_415_2001 H7N3

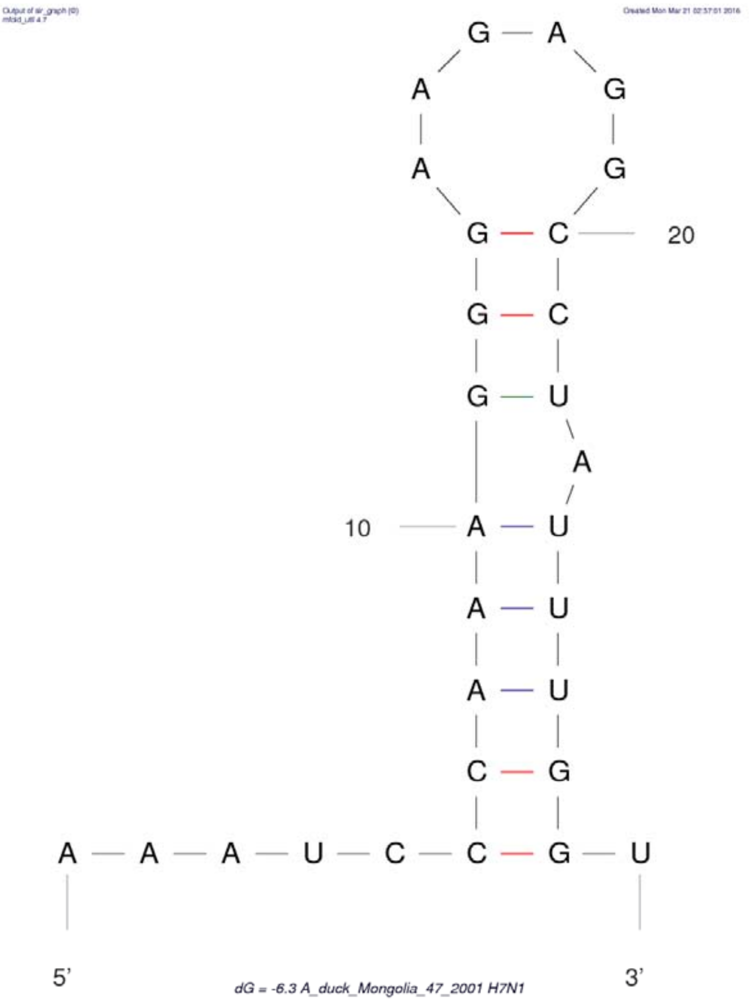

dG = -6.3 A_duck_Mongolia_47_2001 H7N1

Output of sir_graph (©)
mfold_util 4.7

Created Mon Mar 21 02:57:02 2016

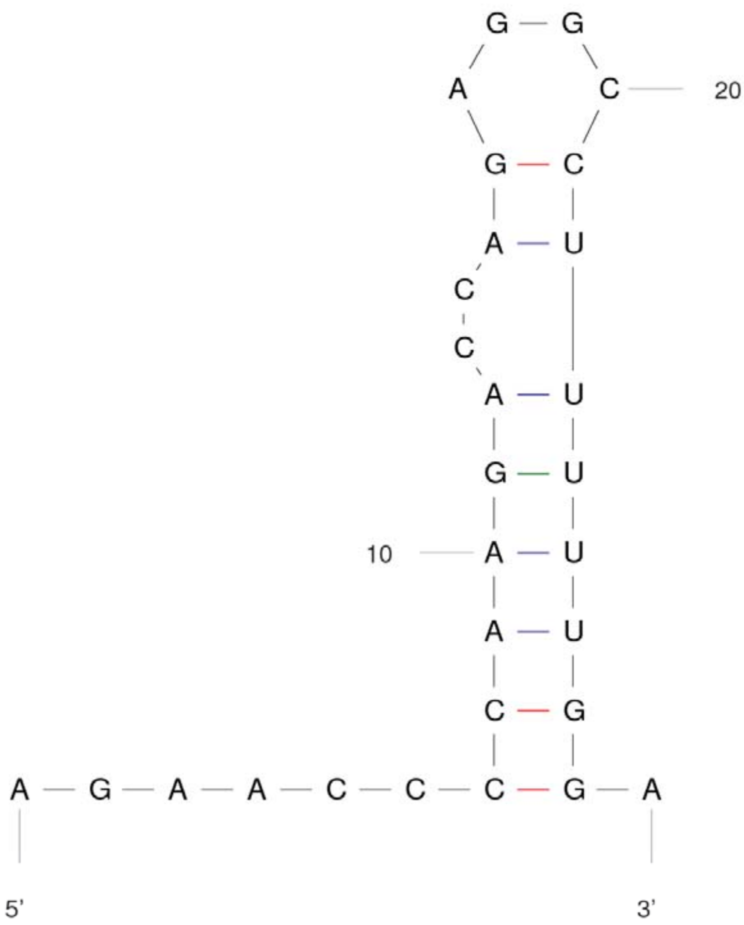

dG = -3.9 A_duck_PA_143585_2001 H7N3

Output of sir_graph (©)
mfold_util 4.7

Created Mon Mar 21 02:57:02 2016

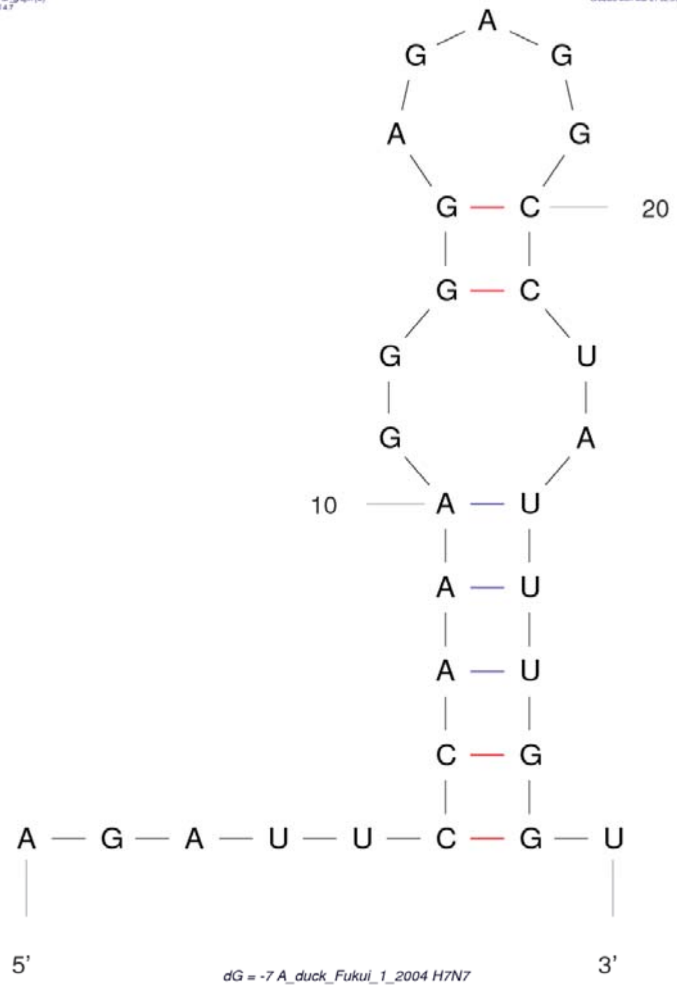

dG = -7 A_duck_Fukui_1_2004 H7N7

Output of sir_graph (©)
mfold_util 4.7

Created Mon Mar 21 02:57:02 2016

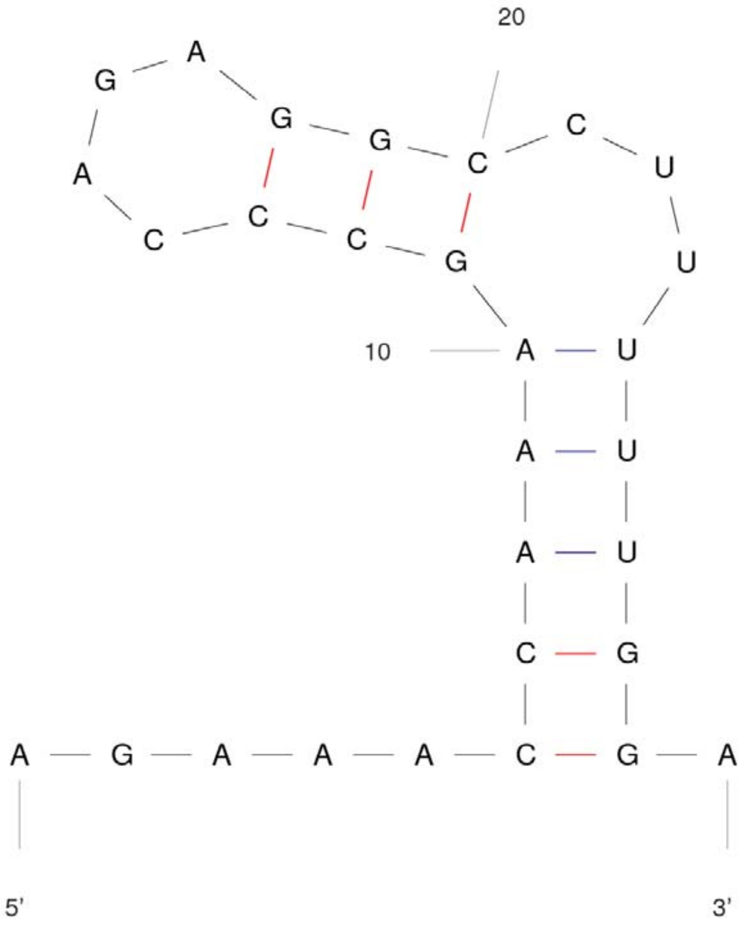

dG = -6.7 A_Muscovy duck_New York_11646-4_2005 H7N2

Output of sir_graph (©)
mfold_util 4.7

Created Mon Mar 21 02:57:02 2016

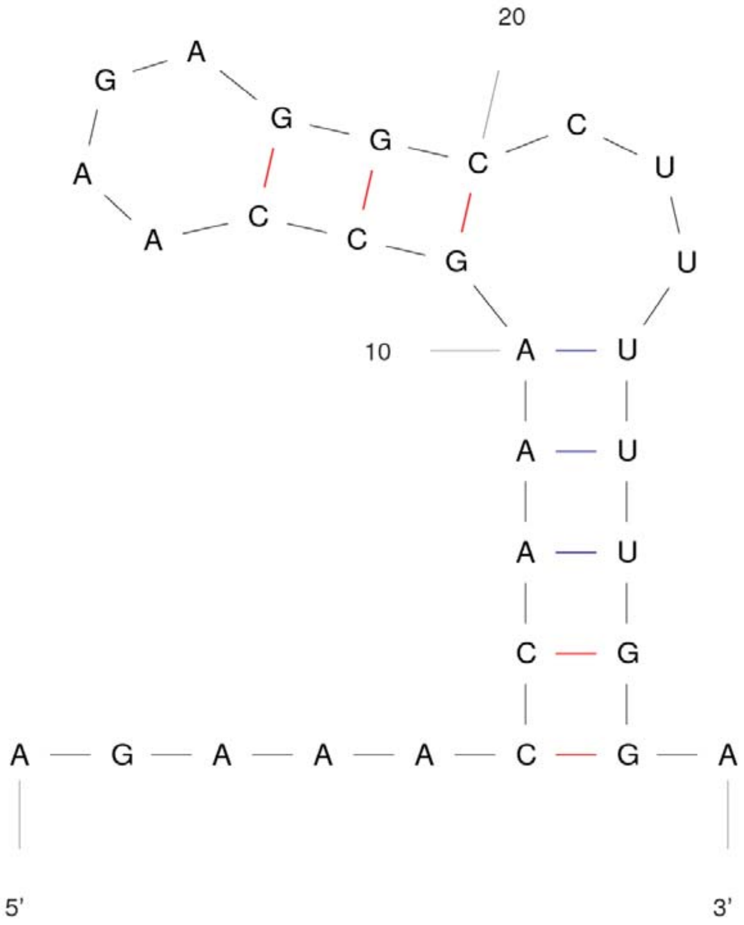

dG = -7.2 A_Muscovy duck_New York_23164-10_2005 H7N2

Output of sir_graph (©)
mfold_util 4.7

Created Mon Mar 21 02:57:02 2016

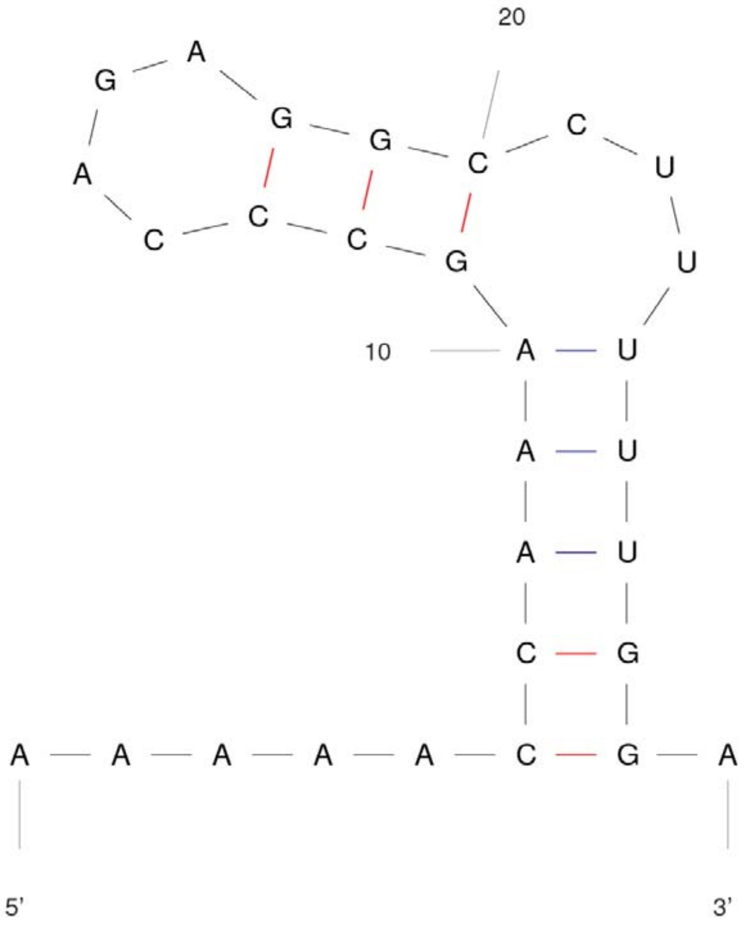

dG = -6.7 A_Muscovy duck_New York_23165-13_2005 H7N2

Output of sir_graph (©)
mfold_util 4.7

Created Mon Mar 21 02:57:03 2016

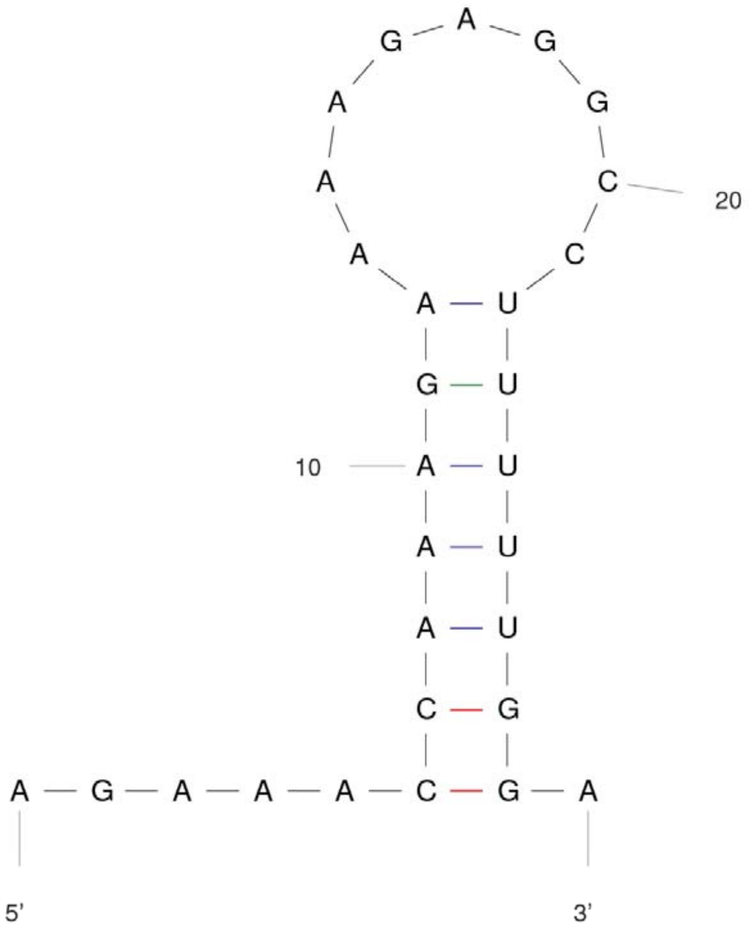

dG = -4.3 A_duck_New York_21211-6_2005 H7N2

Output of sir_graph (©)
mfold_util 4.7

Created Mon Mar 21 02:57:03 2016

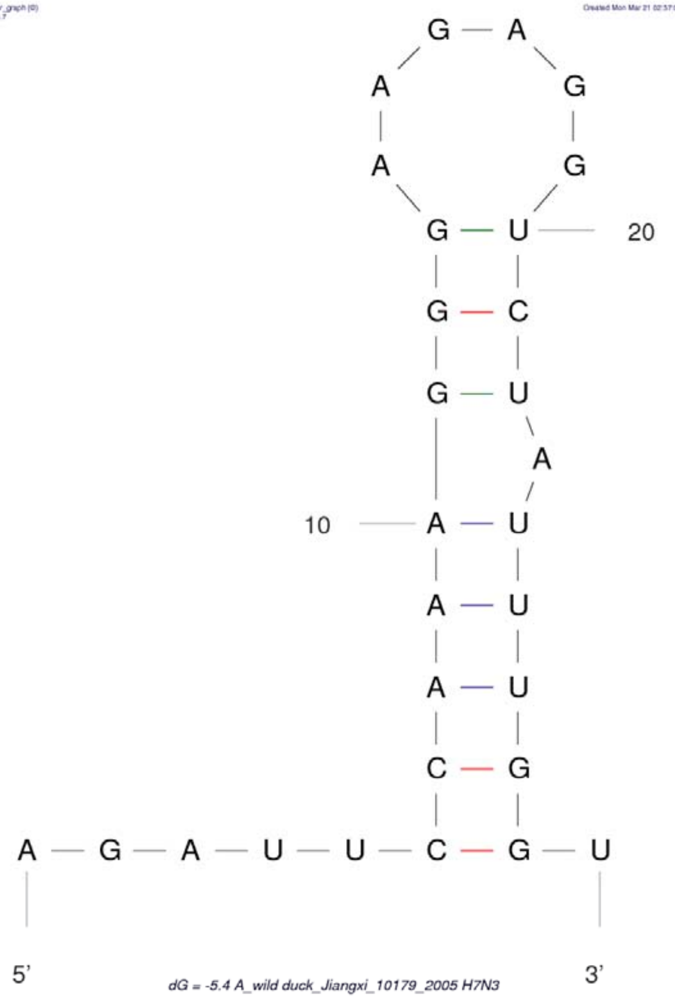

dG = -5.4 A_wild duck_Jiangxi_10179_2005 H7N3

Output of sir_graph (©)
mfold_util 4.7

Created Mon Mar 21 02:57:03 2016

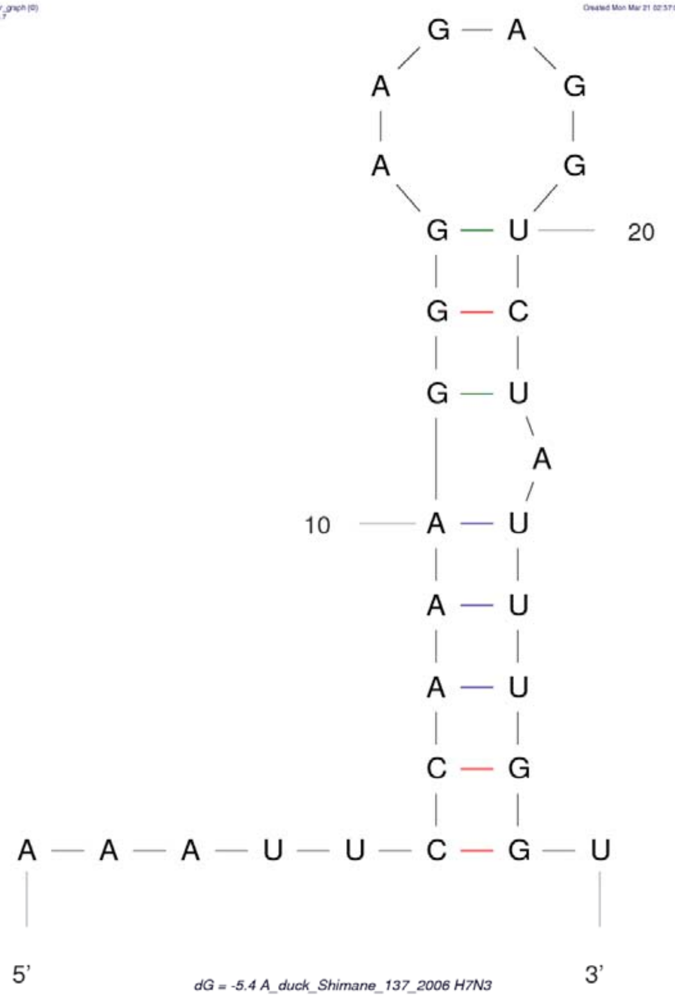

dG = -5.4 A_duck_Shimane_137_2006 H7N3

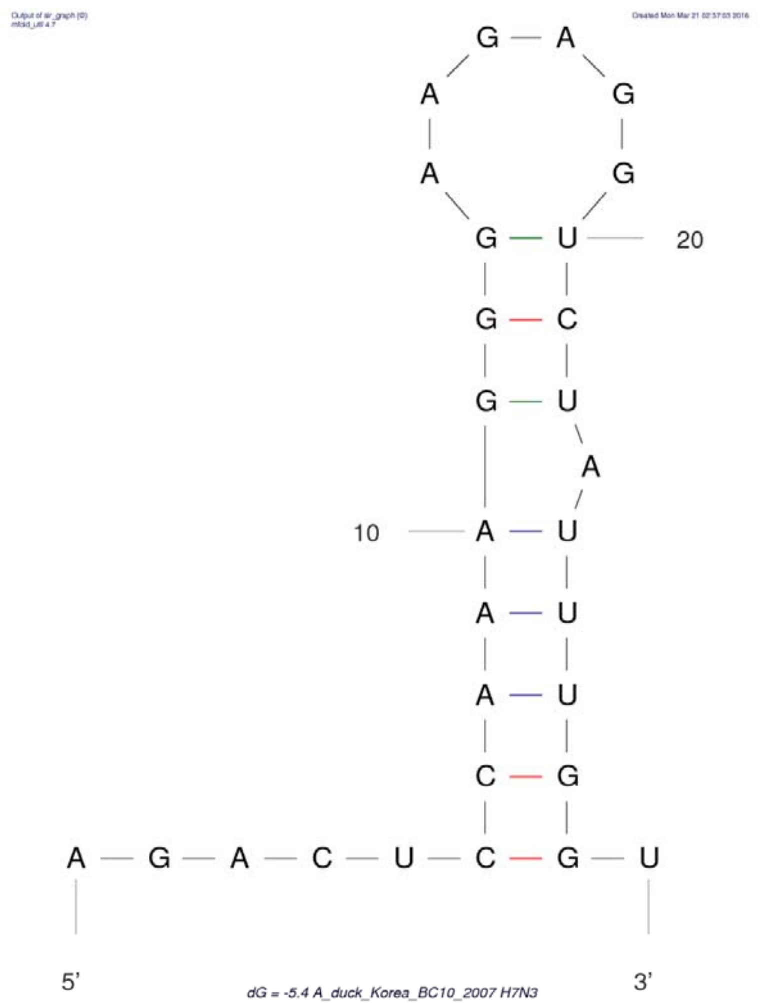

dG = -5.4 A_duck_Korea_BC10_2007 H7N3

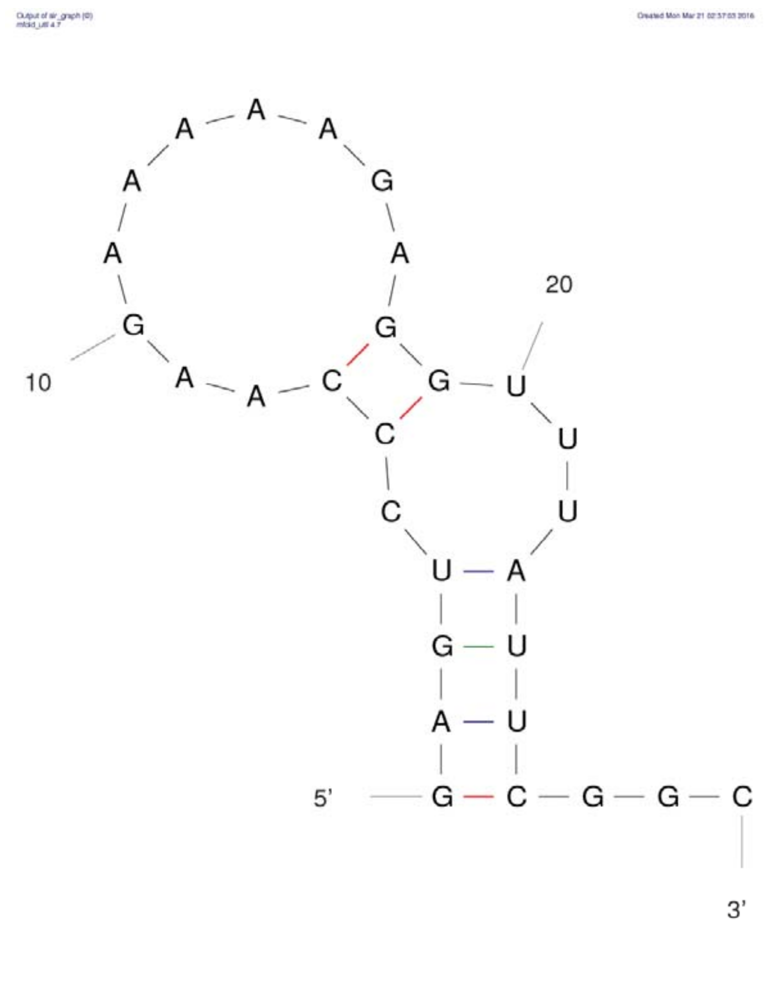

dG = -1 A_duck_Tasmania_277_2007 H7N2

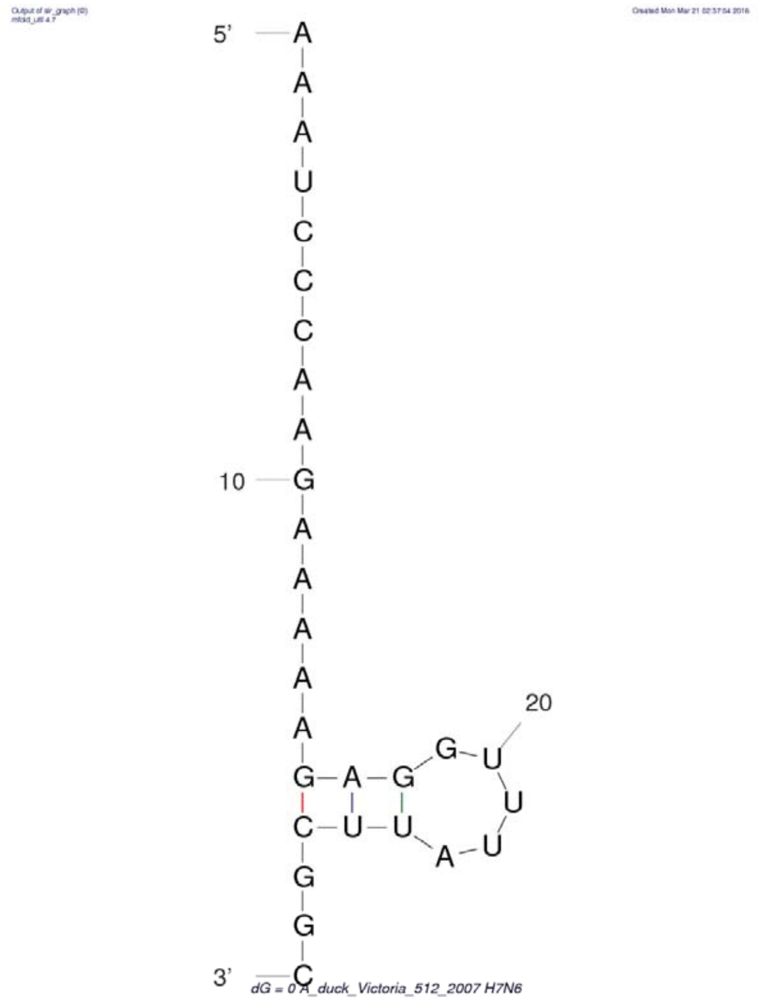

dG = 0 A_duck_Victoria_512_2007 H7N6

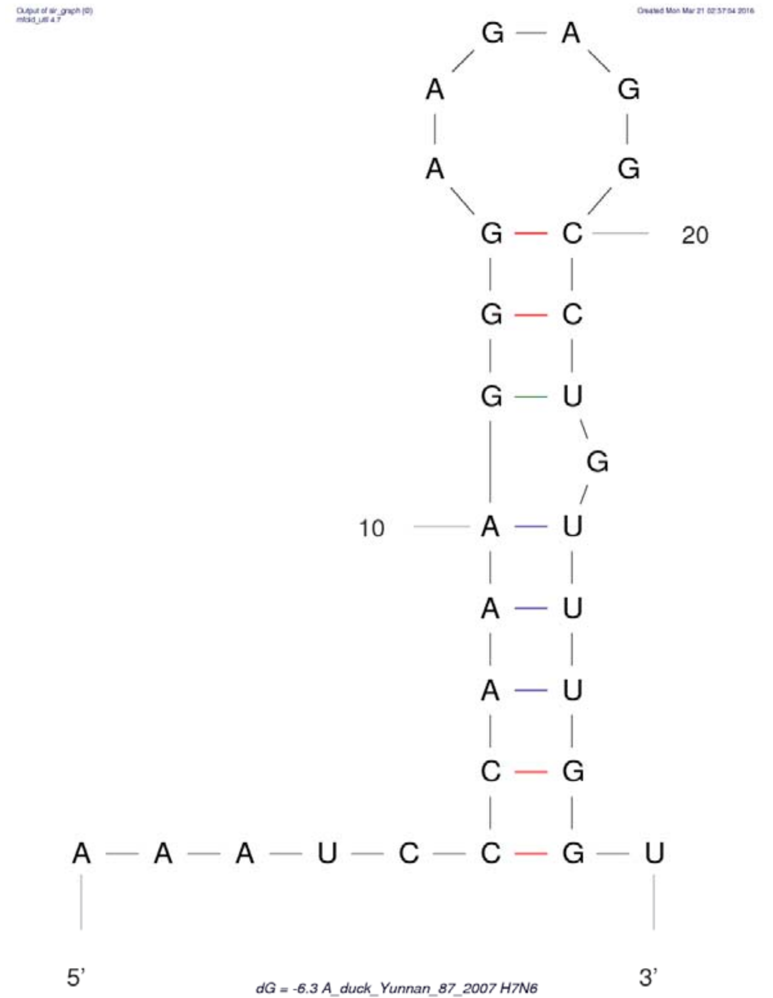

dG = -6.3 A_duck_Yunnan_87_2007 H7N6

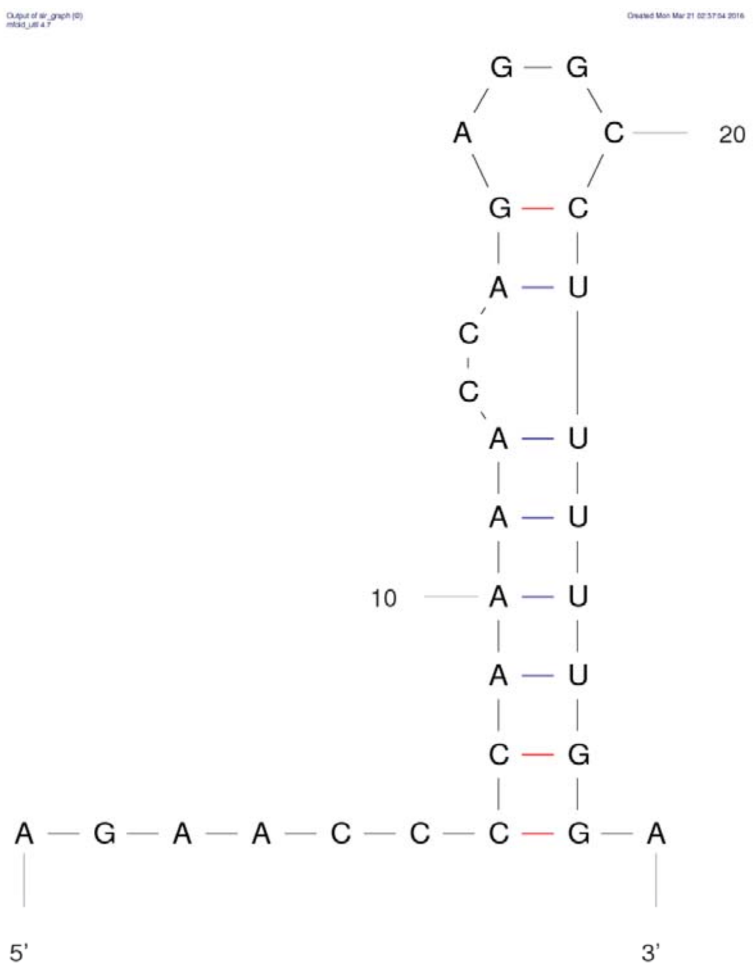

dG = -3.8 A_domestic duck_West Virginia_A00140912_2008 H7N3

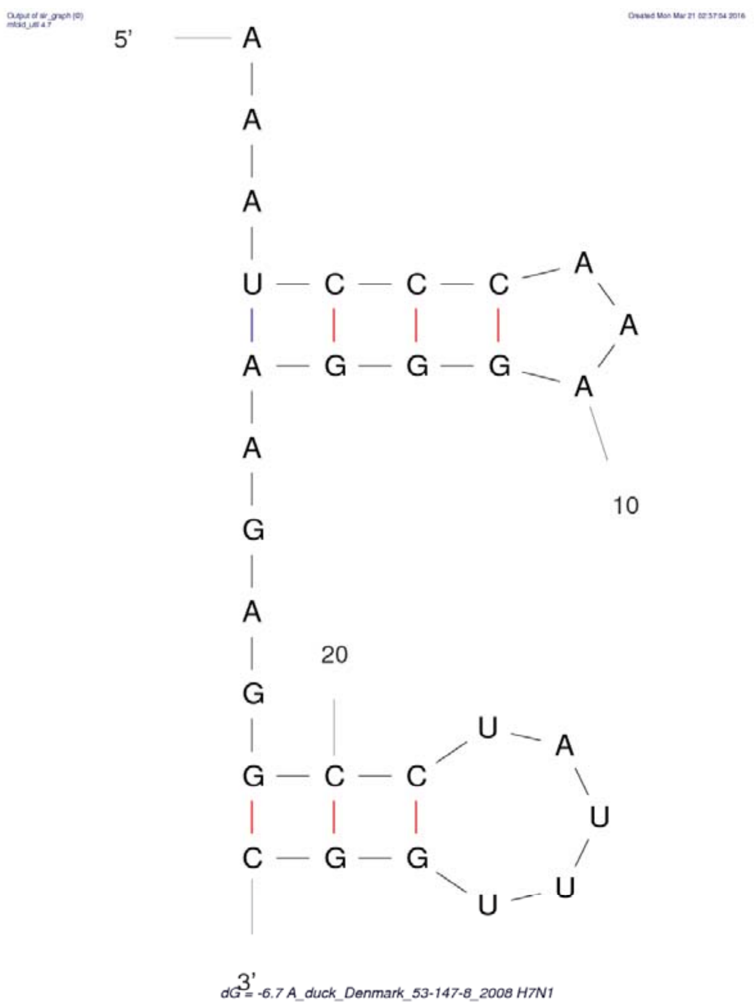

dG = -6.7 A_duck_Denmark_53-147-8_2008 H7N1

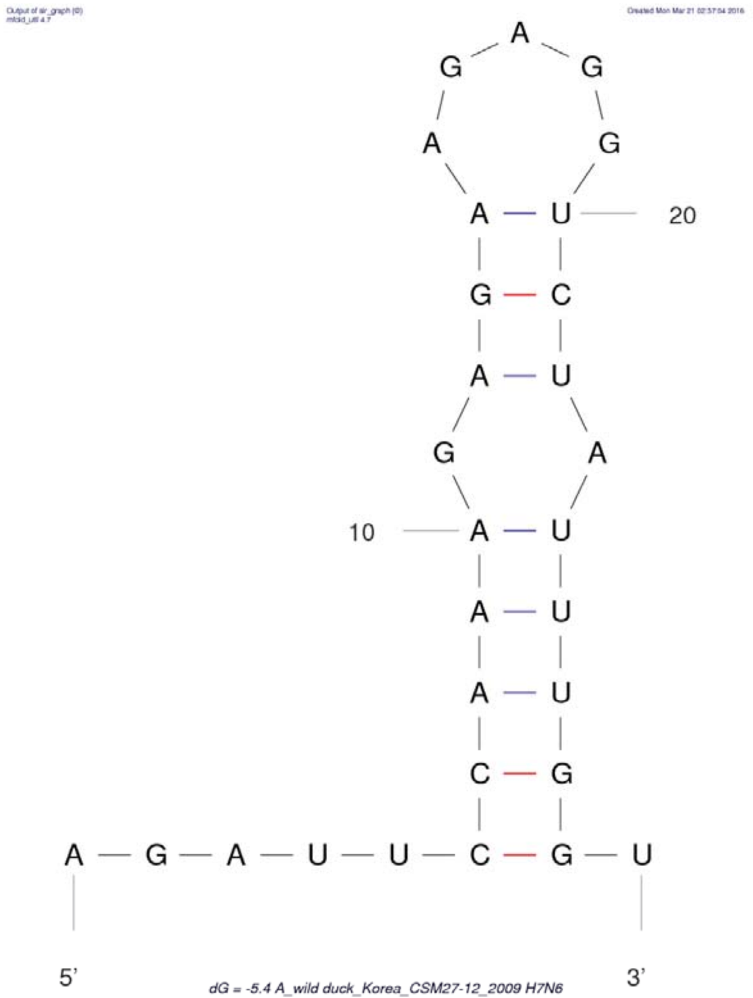

dG = -5.4 A_wild duck_Korea_CSM27-12_2009 H7N6

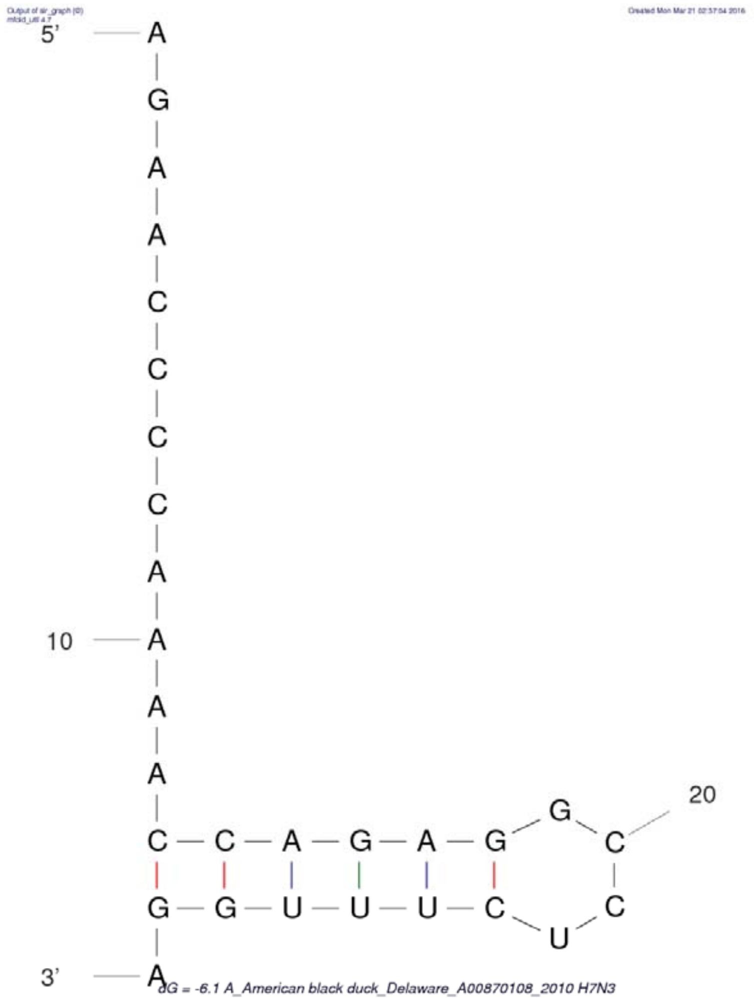

dG = -6.1 A_American black duck_Delaware_A00870108_2010 H7N3

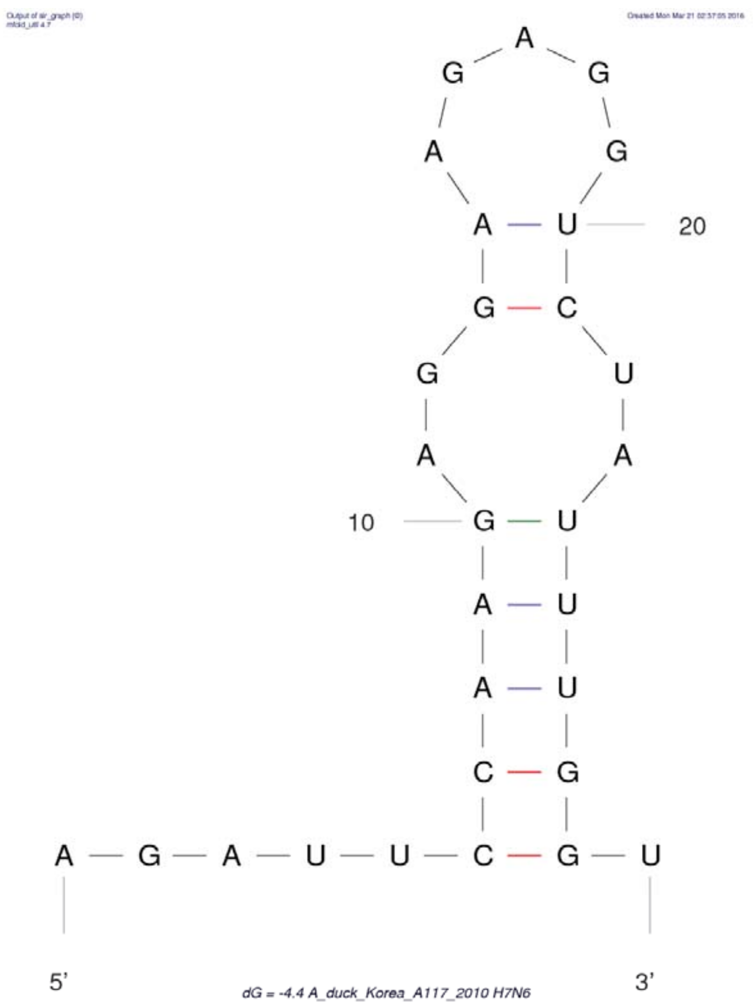

dG = -4.4 A_duck_Korea_A117_2010 H7N6

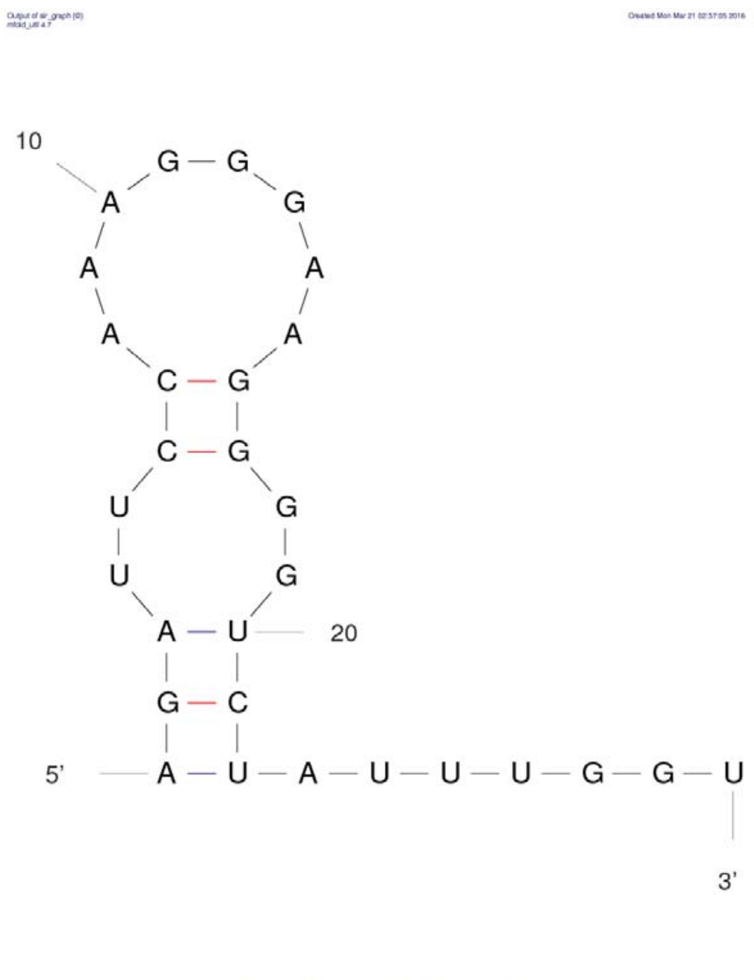

dG = -7 A_duck_Korea_A76_2010 H7N7

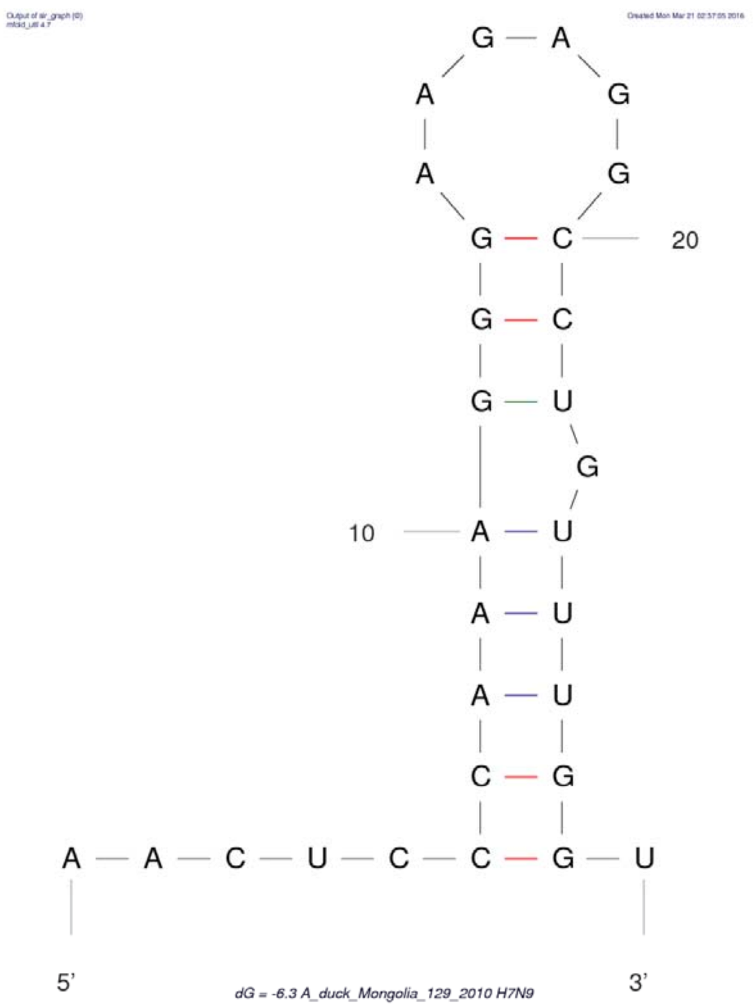

dG = -6.3 A_duck_Mongolia_129_2010 H7N9

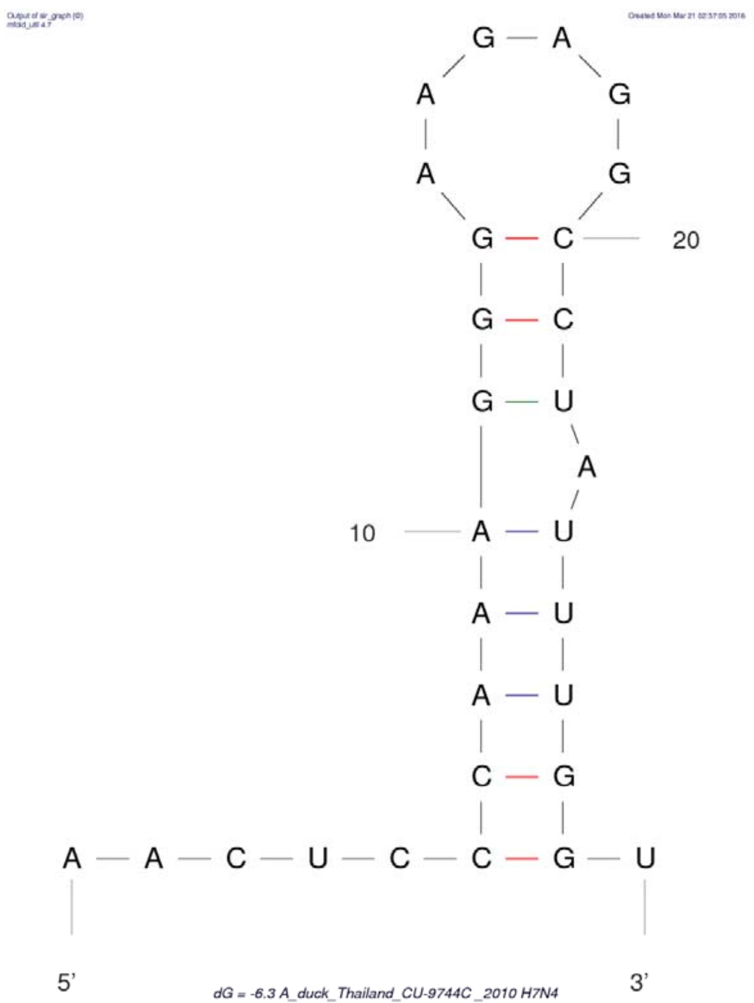

dG = -6.3 A_duck_Thailand_CU-9744C _2010 H7N4

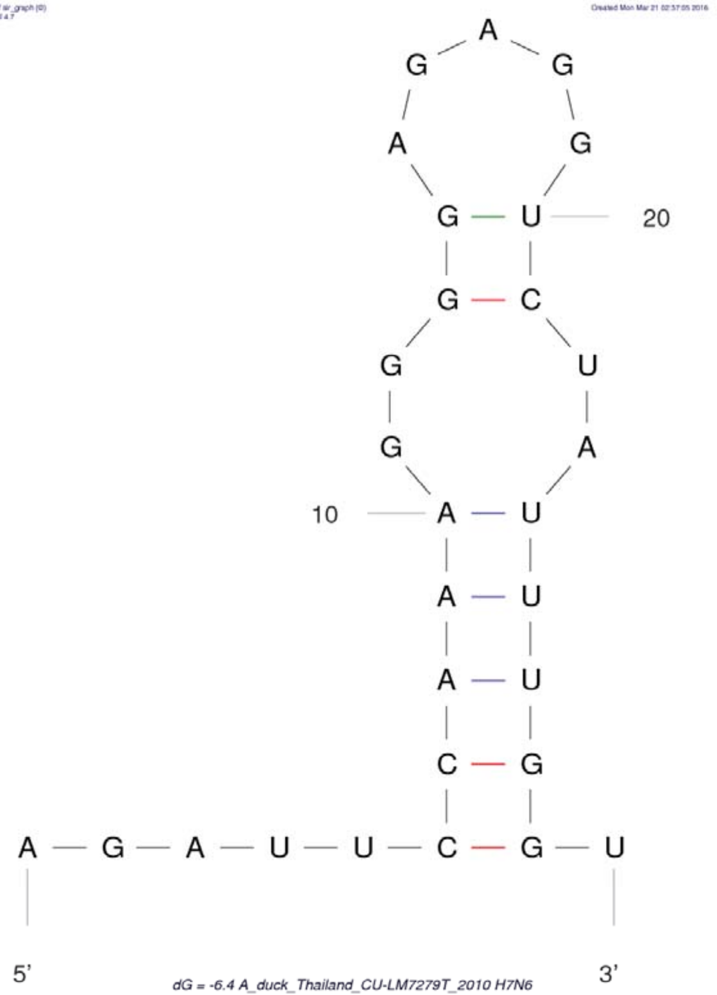

dG = -6.4 A_duck_Thailand_CU-LM7279T_2010 H7N6

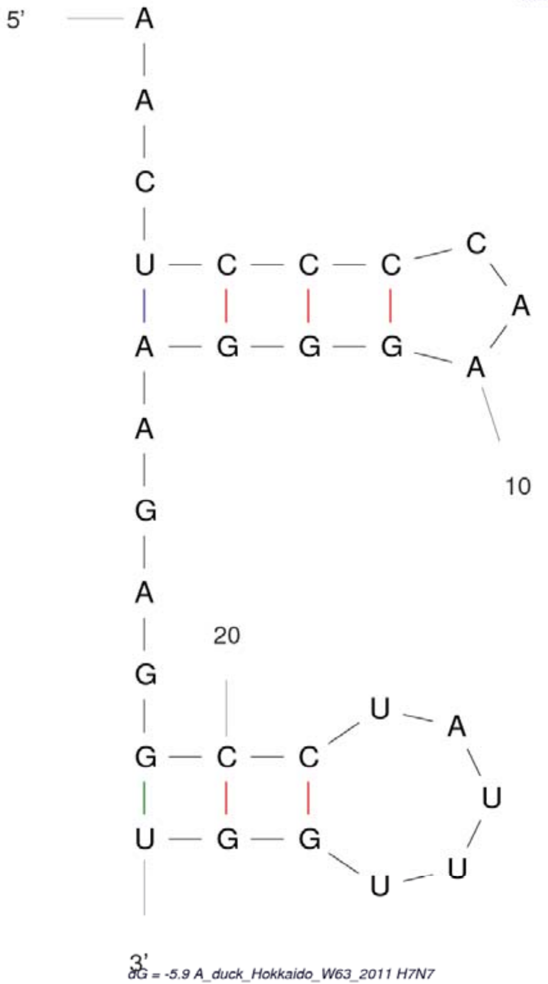

dG = -5.9 A_duck_Hokkaido_W63_2011 H7N7

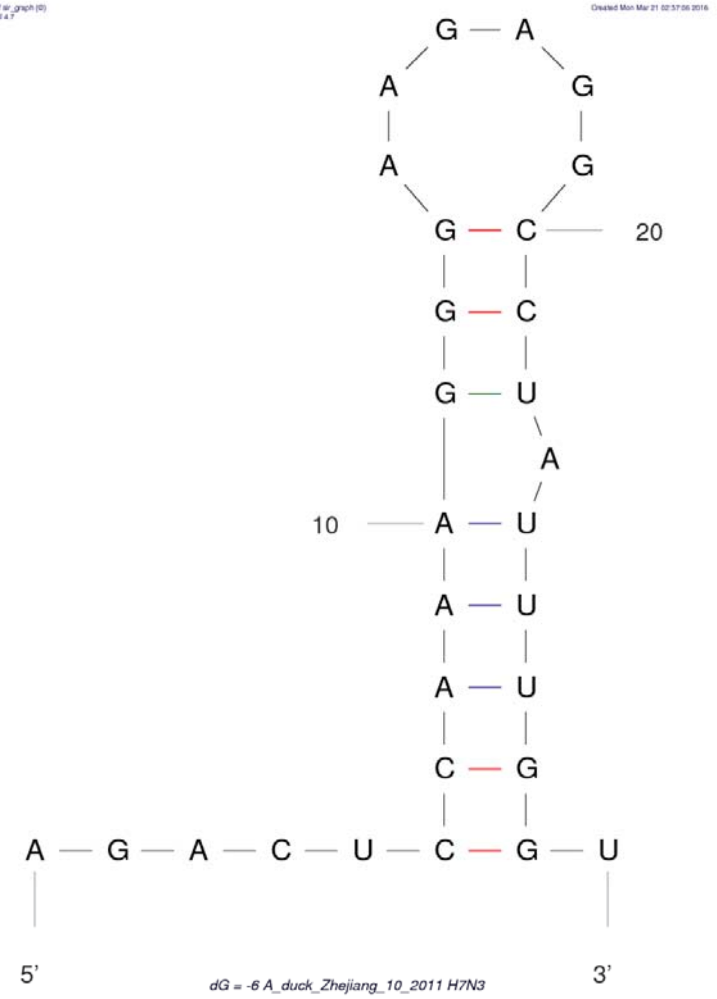

dG = -6 A_duck_Zhejiang_10_2011 H7N3

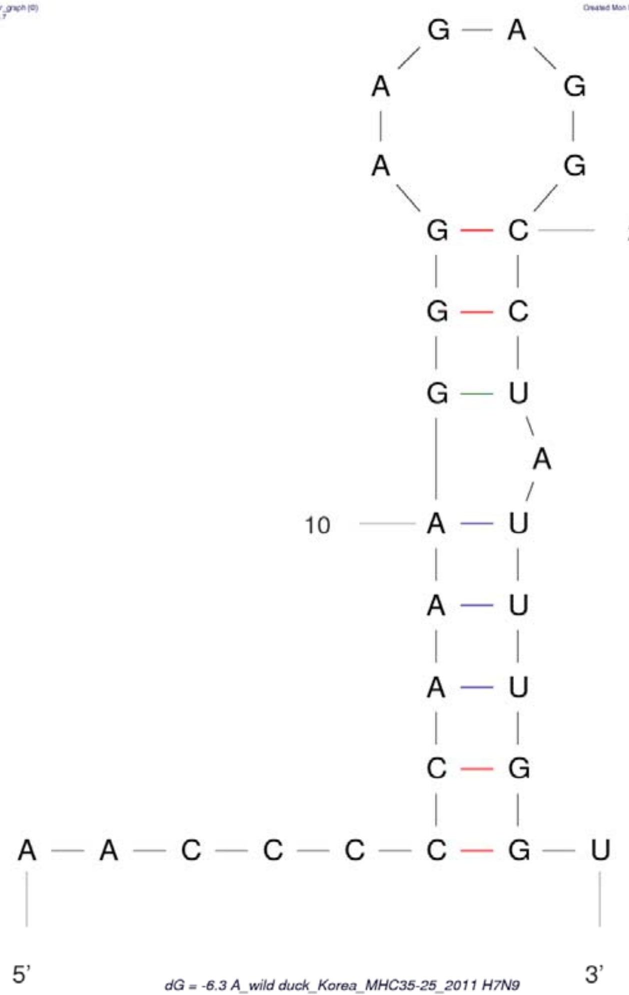

dG = -6.3 A_wild duck_Korea_MHC35-25_2011 H7N9

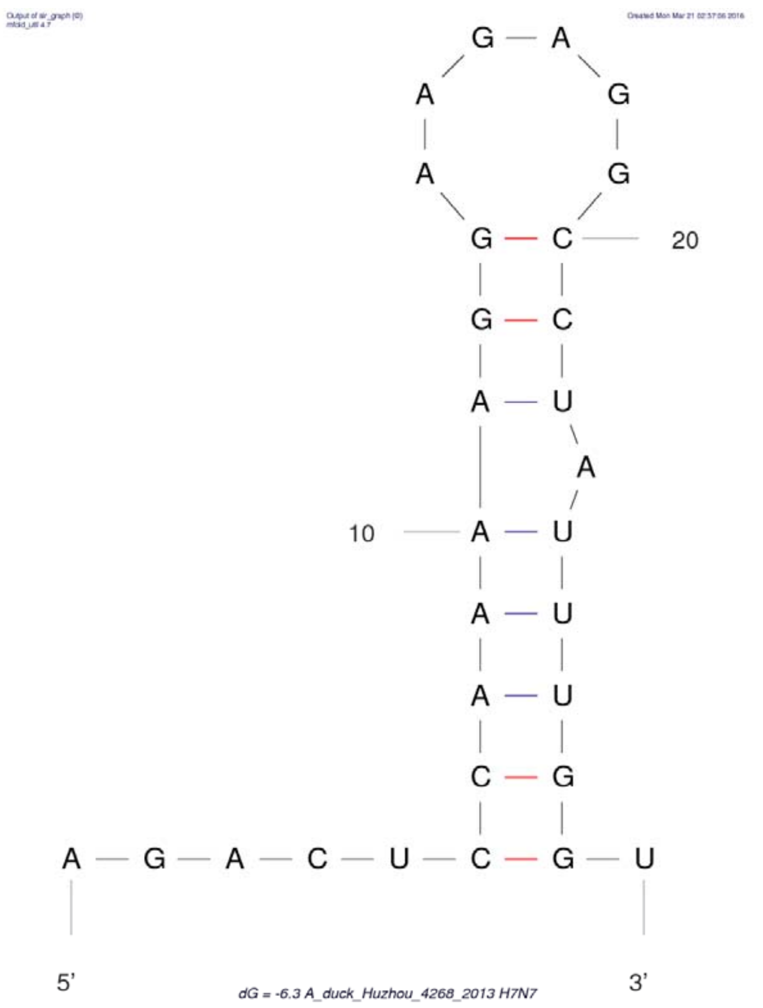

dG = -6.3 A_duck_Huzhou_4268_2013 H7N7

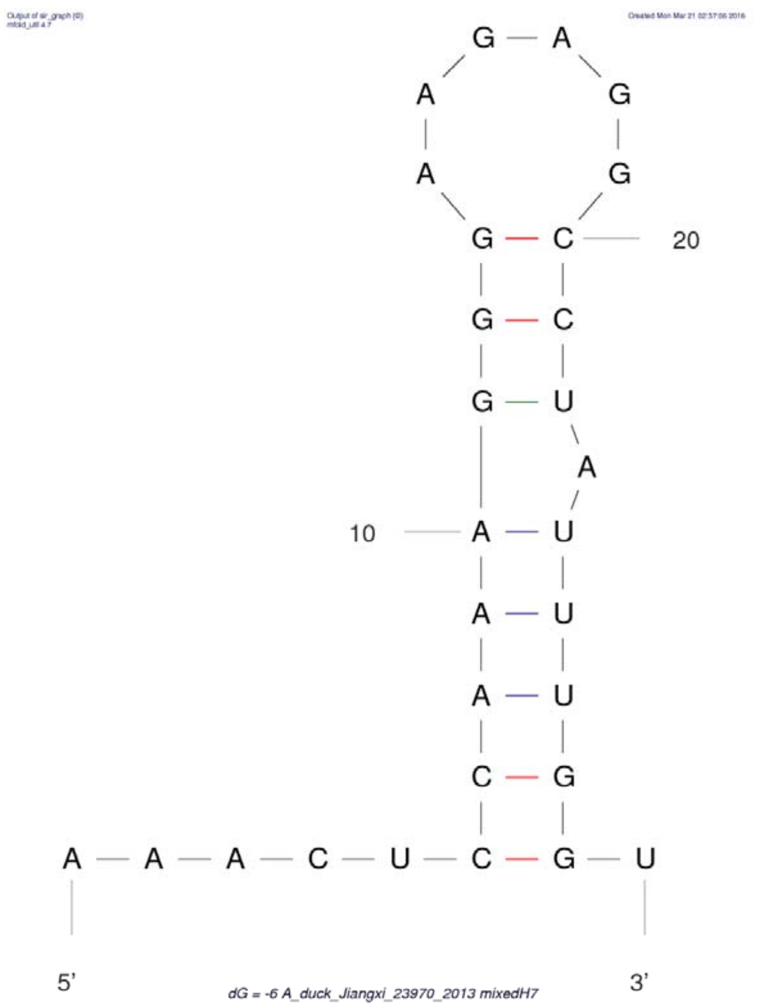

dG = -6 A_duck_Jiangxi_23970_2013 mixedH7

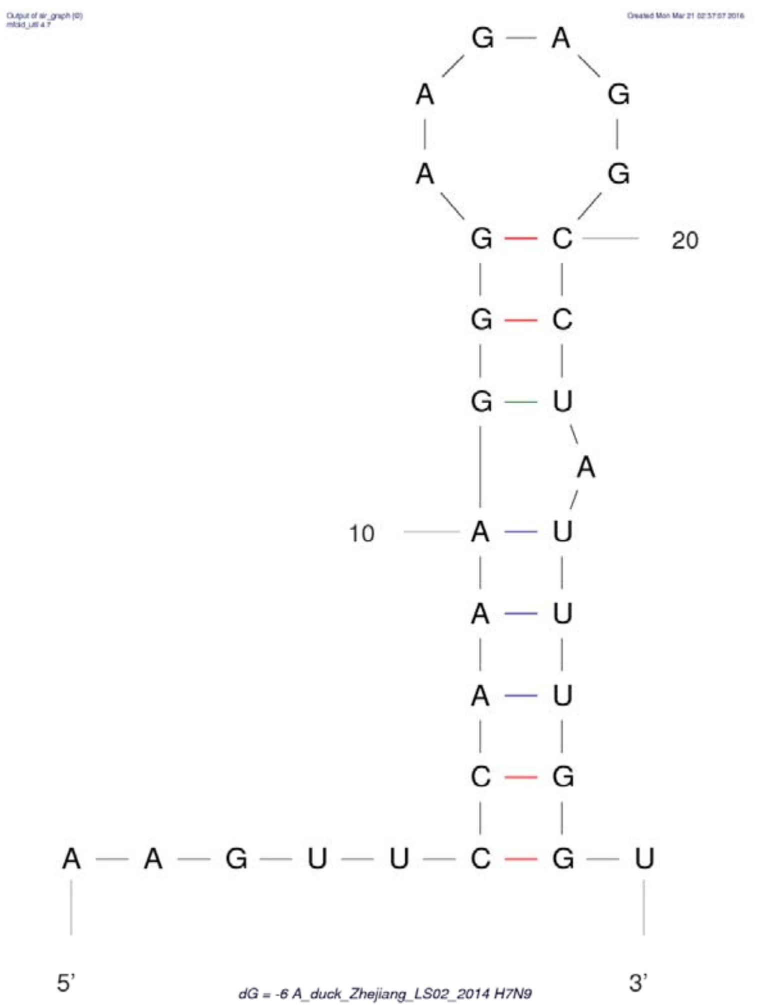

dG = -6 A_duck_Zhejiang_LS02_2014 H7N9

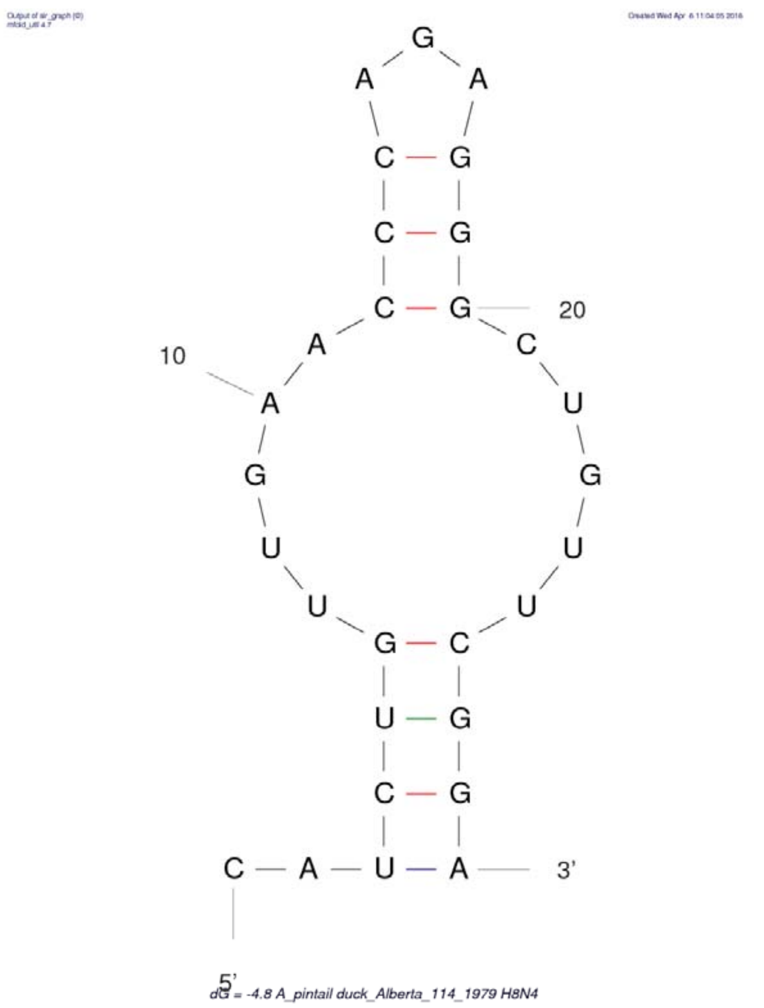

dG = -4.8 A_pintail duck_Alberta_114_1979 H8N4

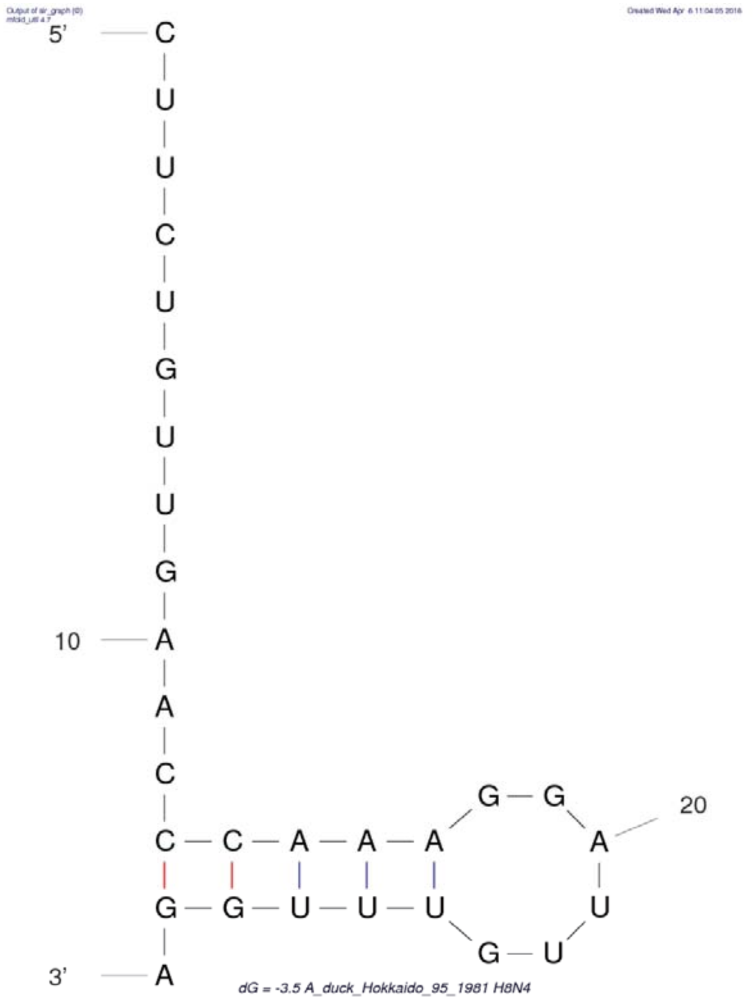

dG = -3.5 A_duck_Hokkaido_95_1981 H8N4

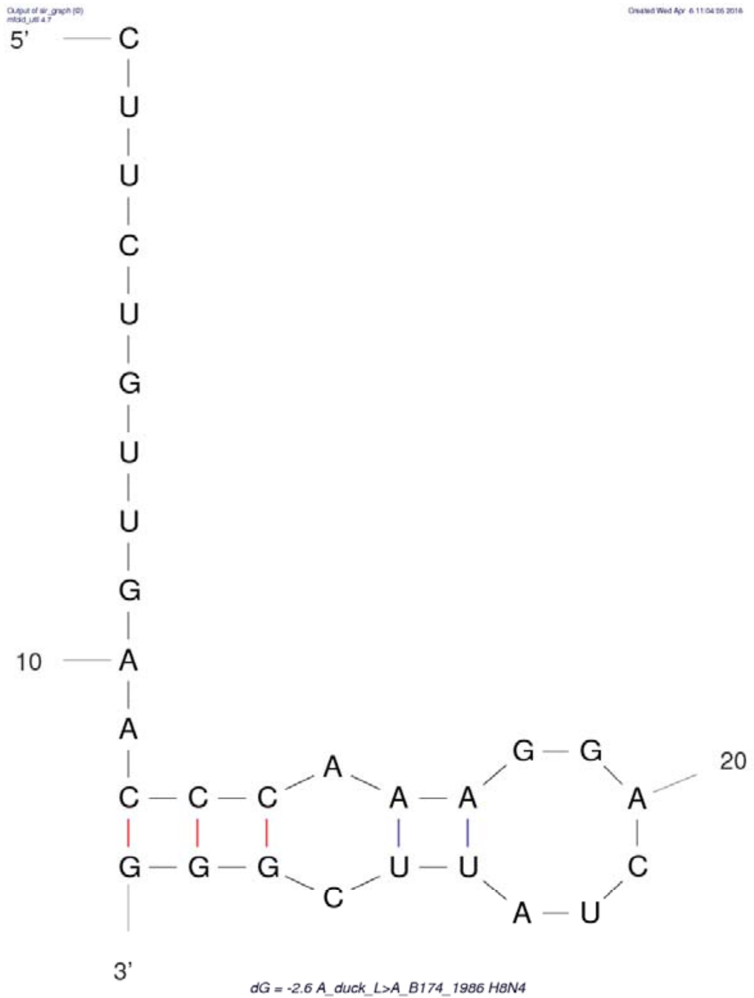

dG = -2.6 A_duck_L>A_B174_1986 H8N4

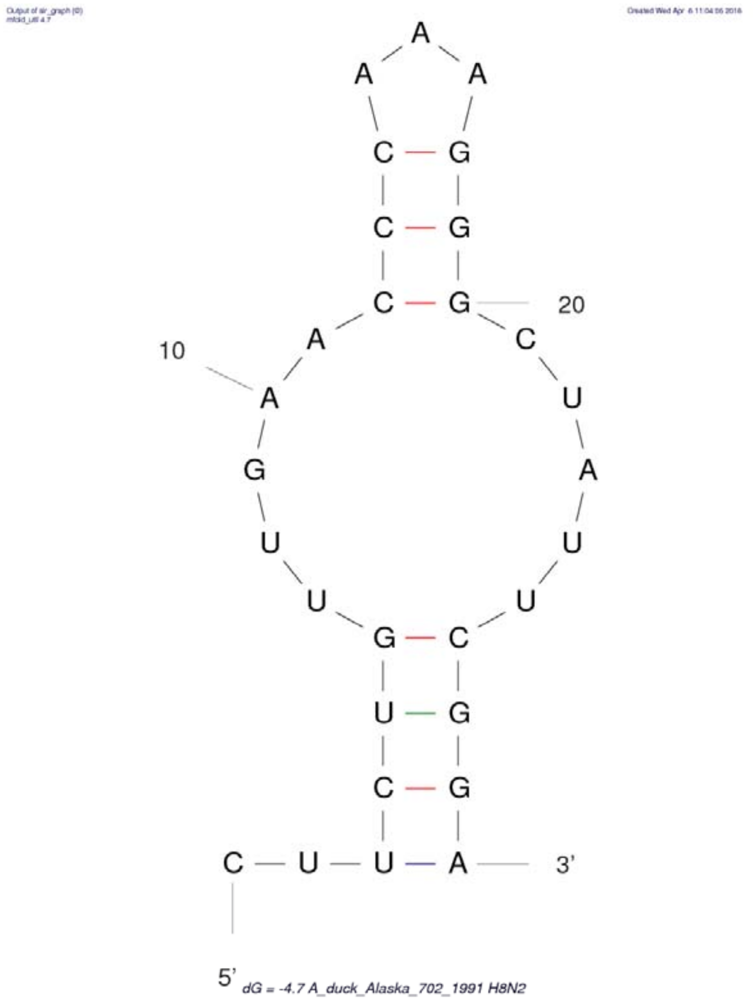

dG = -4.7 A_duck_Alaska_702_1991 H8N2

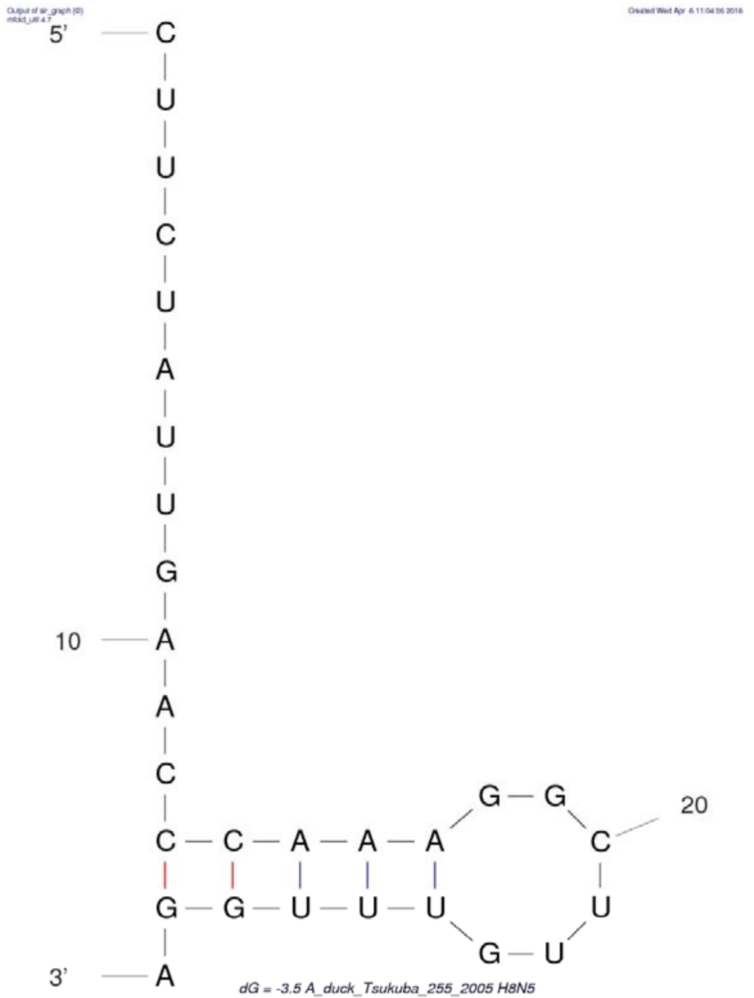

dG = -3.5 A_duck_Tsukuba_255_2005 H8N5

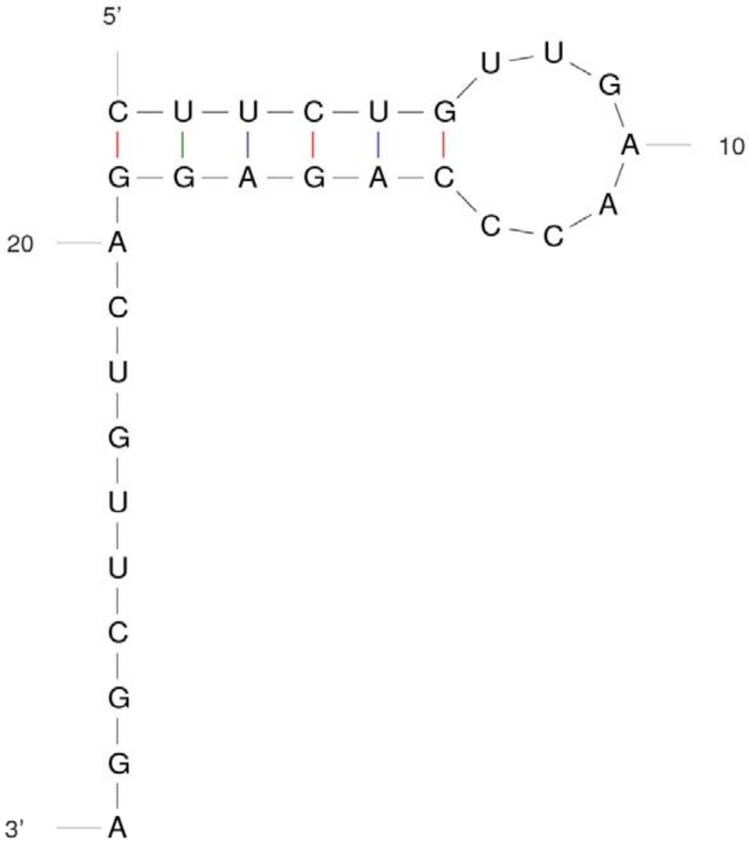

dG = -5.5 A_duck_Yangzhou_02_2005 H8N4

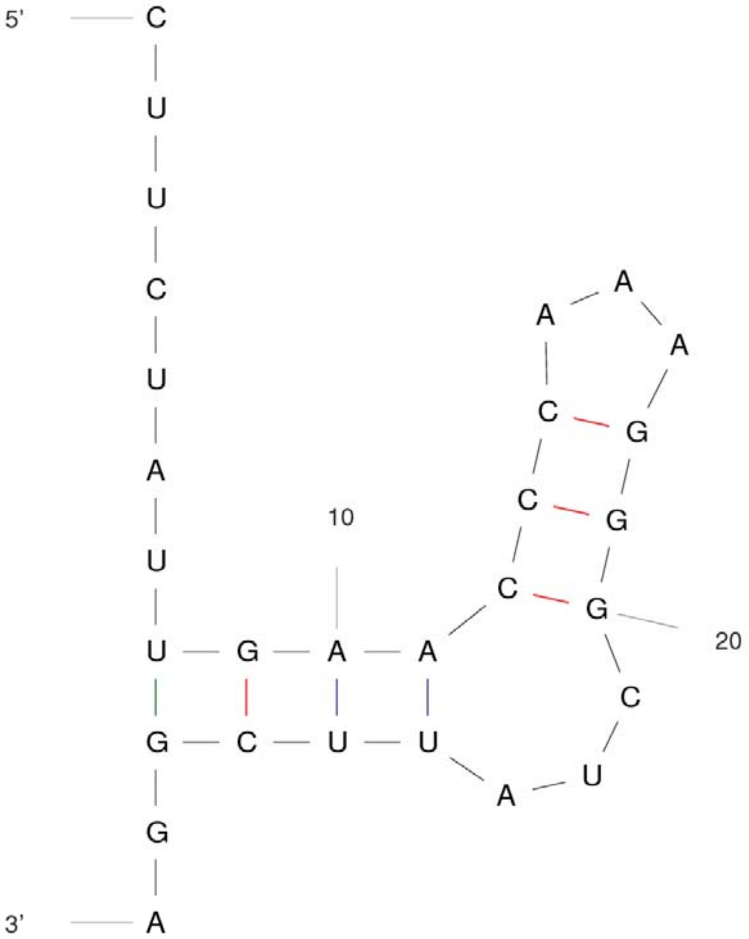

dG = -2.2 A_American black duck_Nova Scotia_02043_2007 H8N4

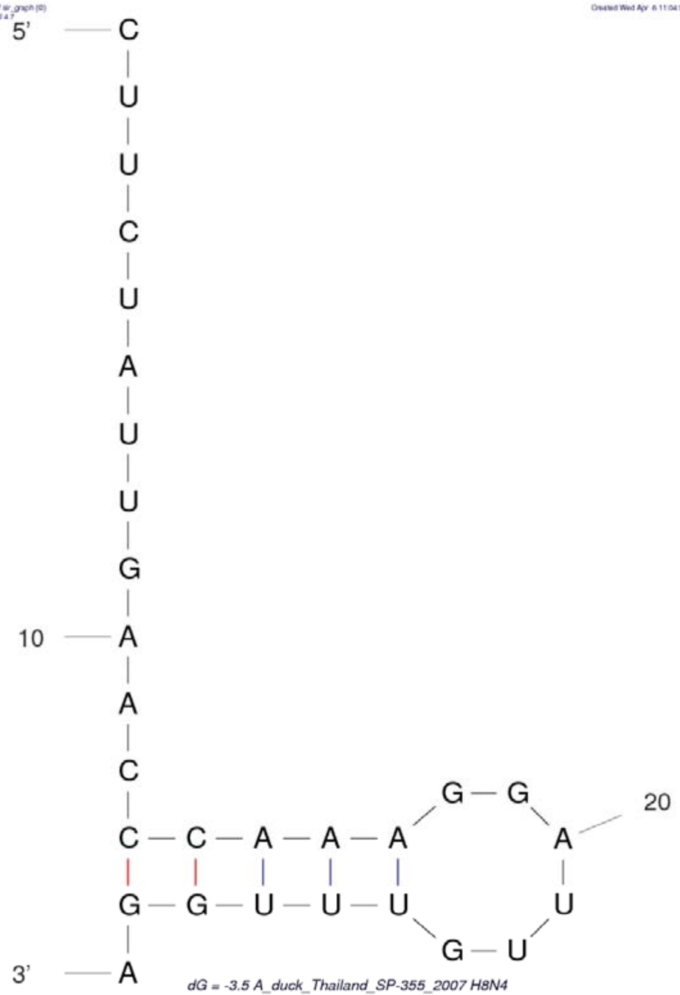

dG = -3.5 A_duck_Thailand_SP-355_2007 H8N4

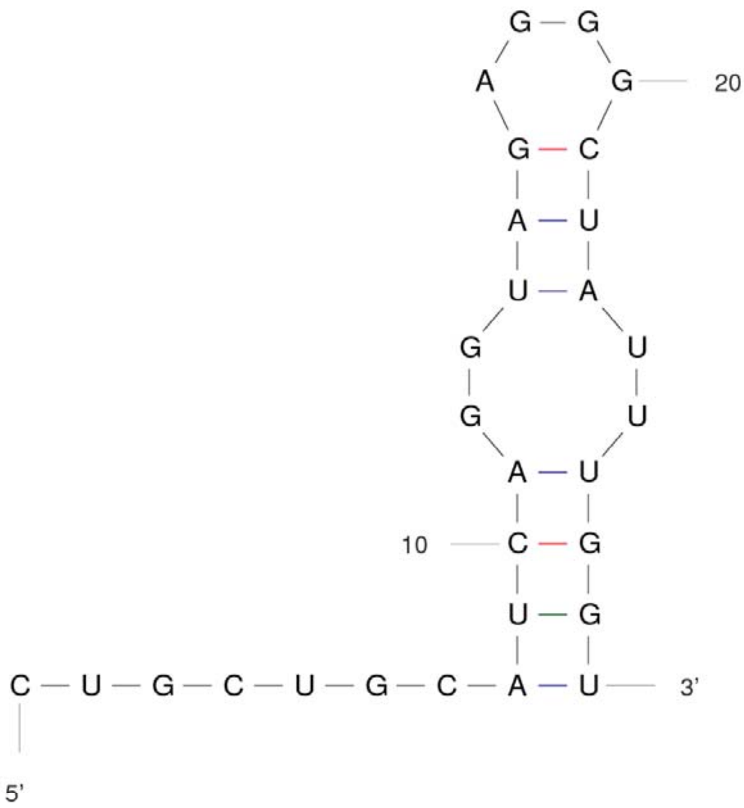

dG = -5 A_duck_Hong Kong_91_1976 H9N2

Output of sir_graph (©)
mfold_util 4.7

Created Mon Mar 21 03:50:49 2016

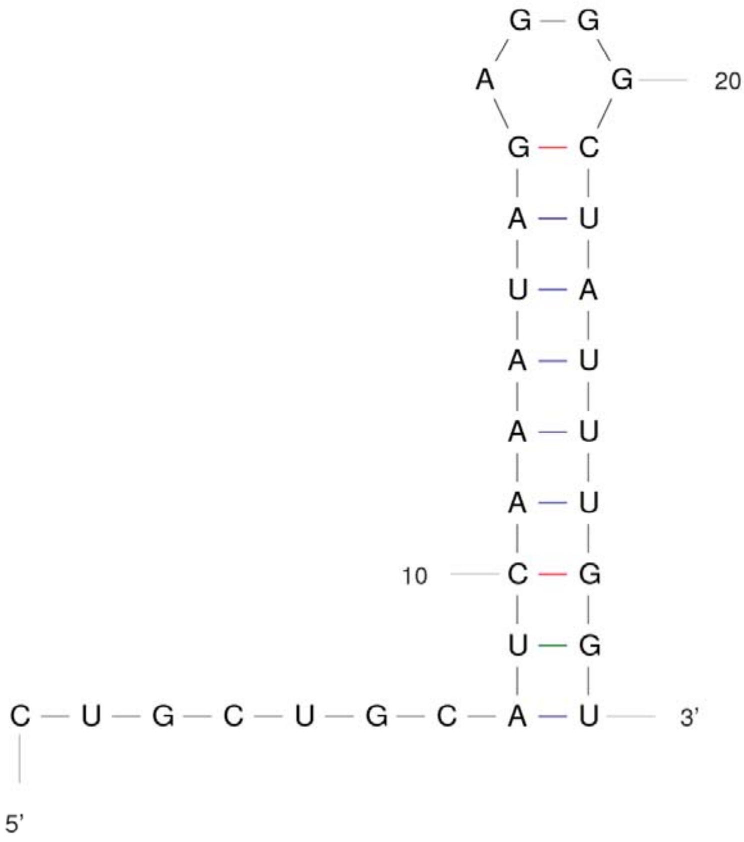

dG = -6.8 A_duck_Hong Kong_147_1977 H9N6

Output of sir_graph (©)
mfold_util 4.7

Created Mon Mar 21 03:50:49 2016

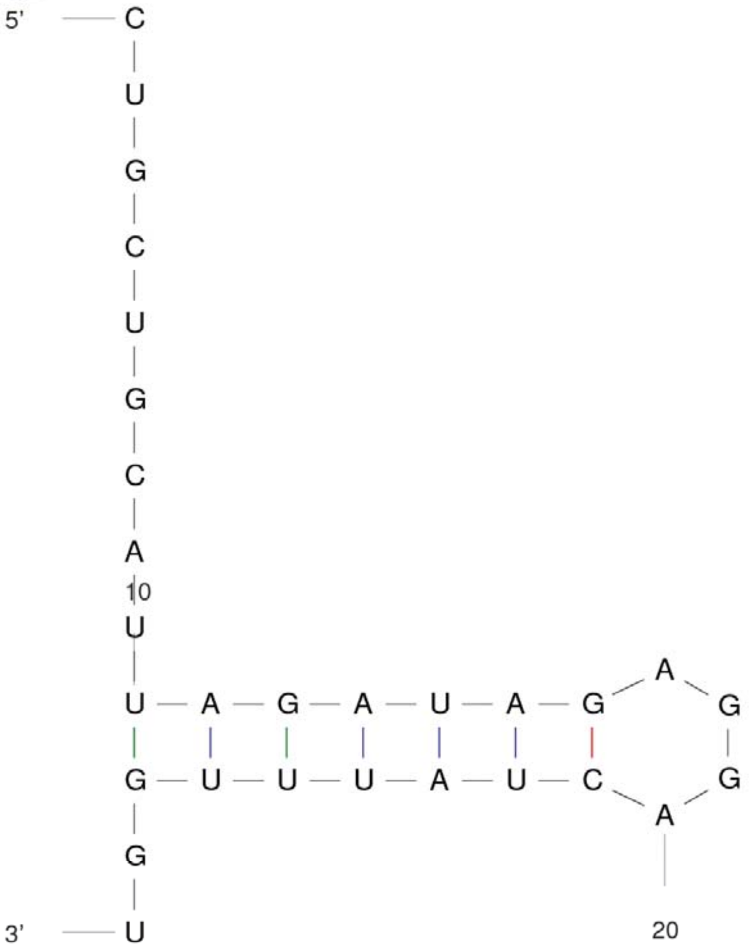

dG = -3.2 A_duck_Hong Kong_366_78 H9N2

Output of sir_graph (©)
mfold_util 4.7

Created Mon Mar 21 03:50:49 2016

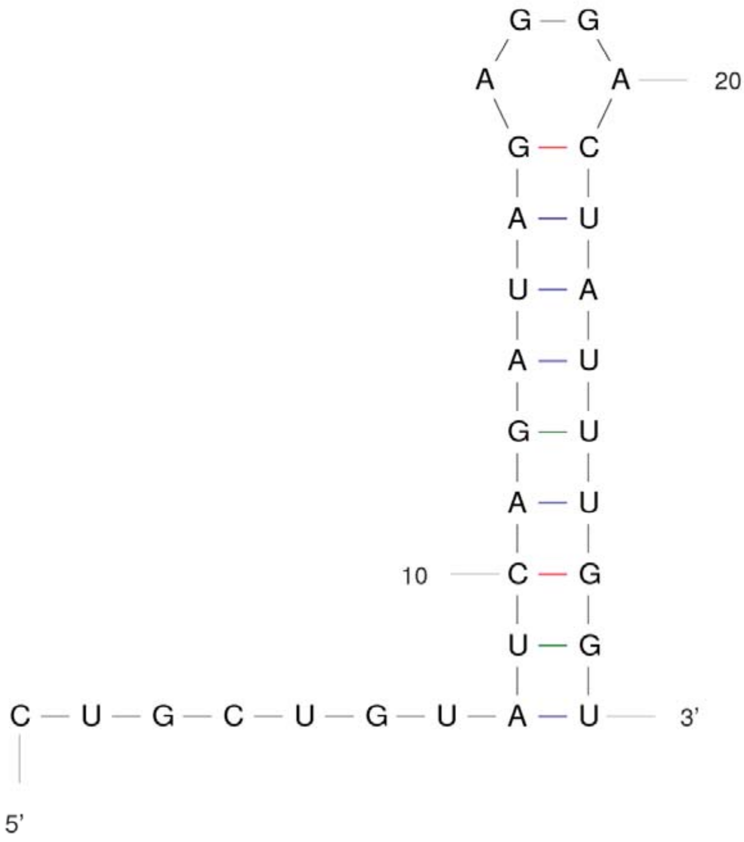

dG = -6.6 A_duck_Hong Kong_448_78 H9N2

Output of sir_graph (©)
mfold_util 4.7

Created Mon Mar 21 03:50:49 2016

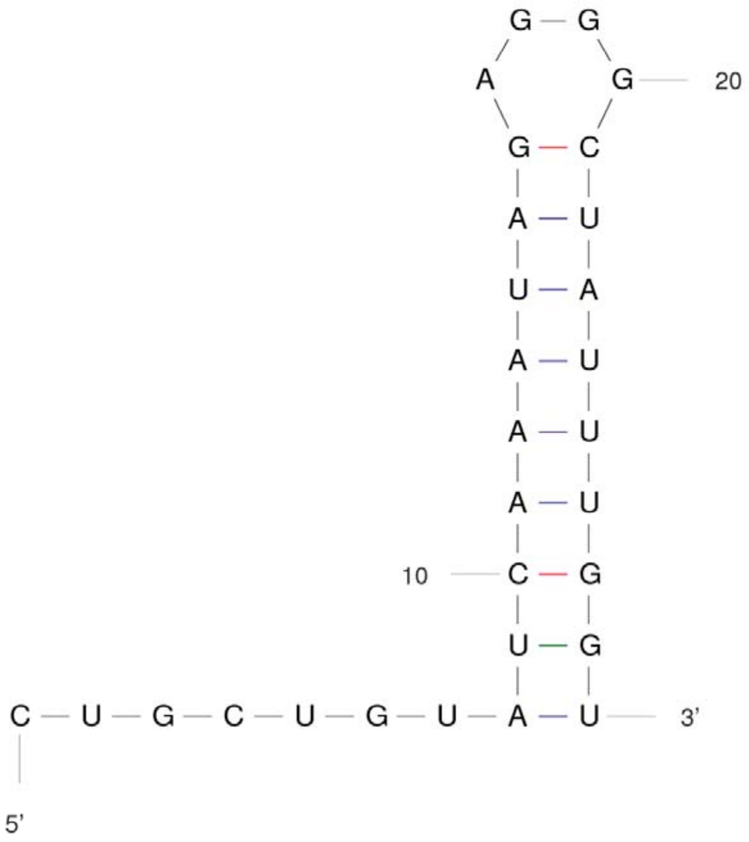

dG = -6.7 A_duck_Hong Kong_552_79 H9N2

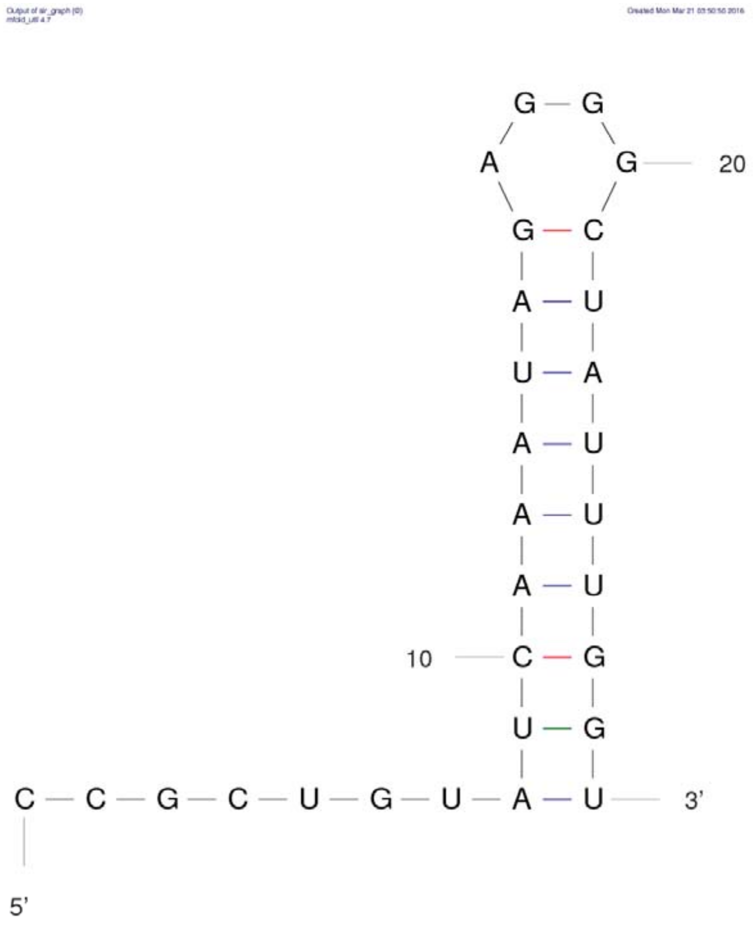

dG = -6.7 A_duck_Hong Kong_610_79 H9N2

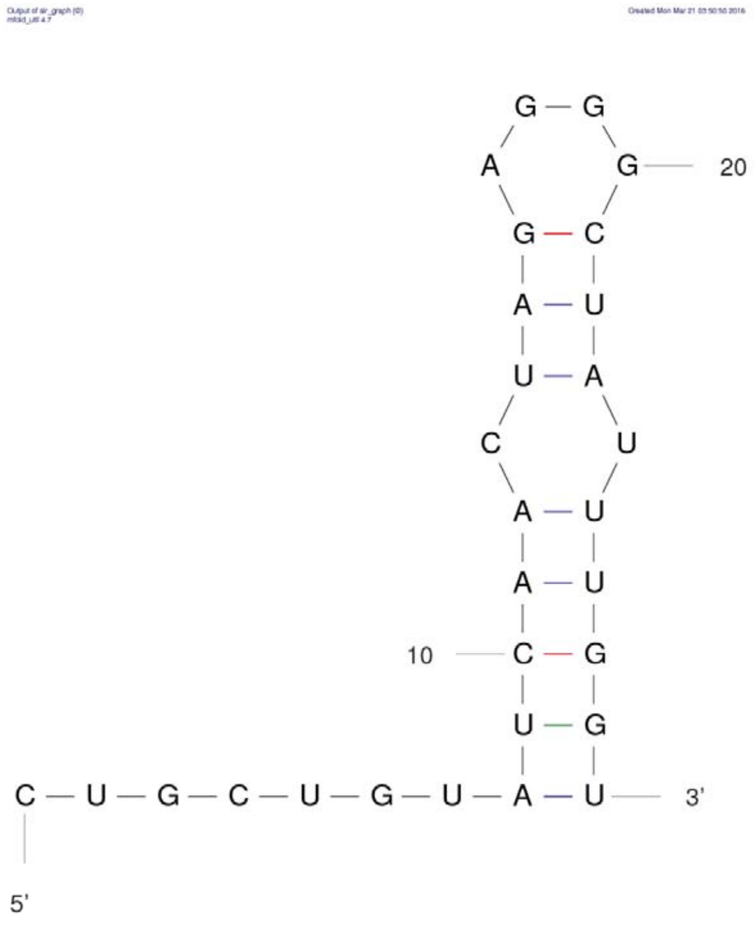

dG = -3 A_duck_Hong Kong_702_1979-chicken adapted H9N2

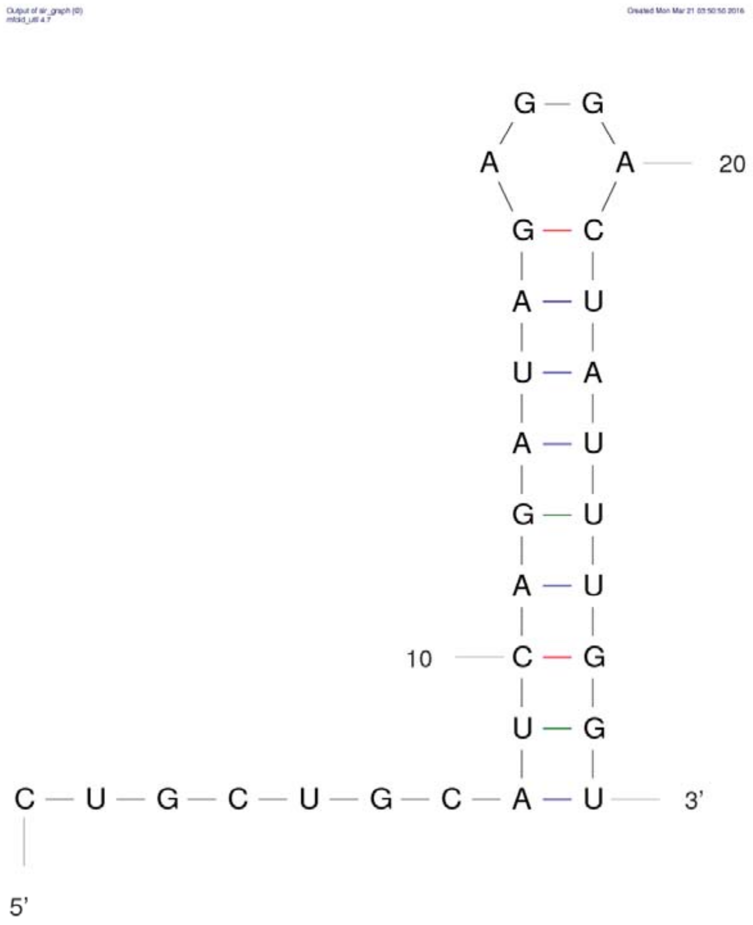

dG = -6.7 A_duck_Hong Kong_784_1979 H9N2

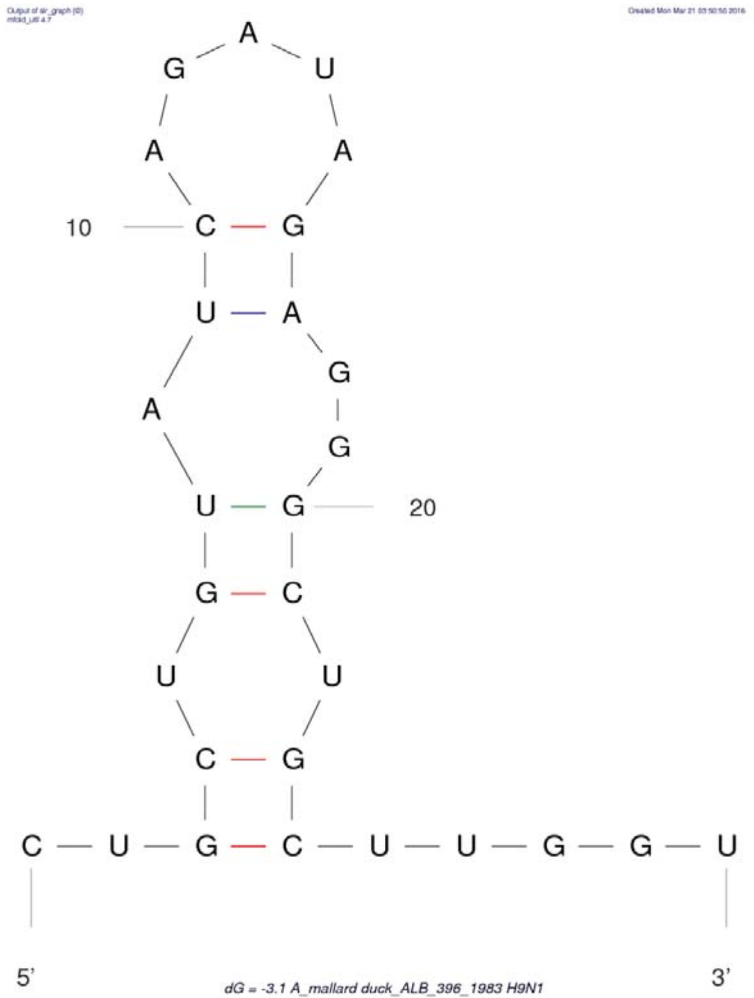

dG = -3.1 A_mallard duck_ALB_396_1983 H9N1

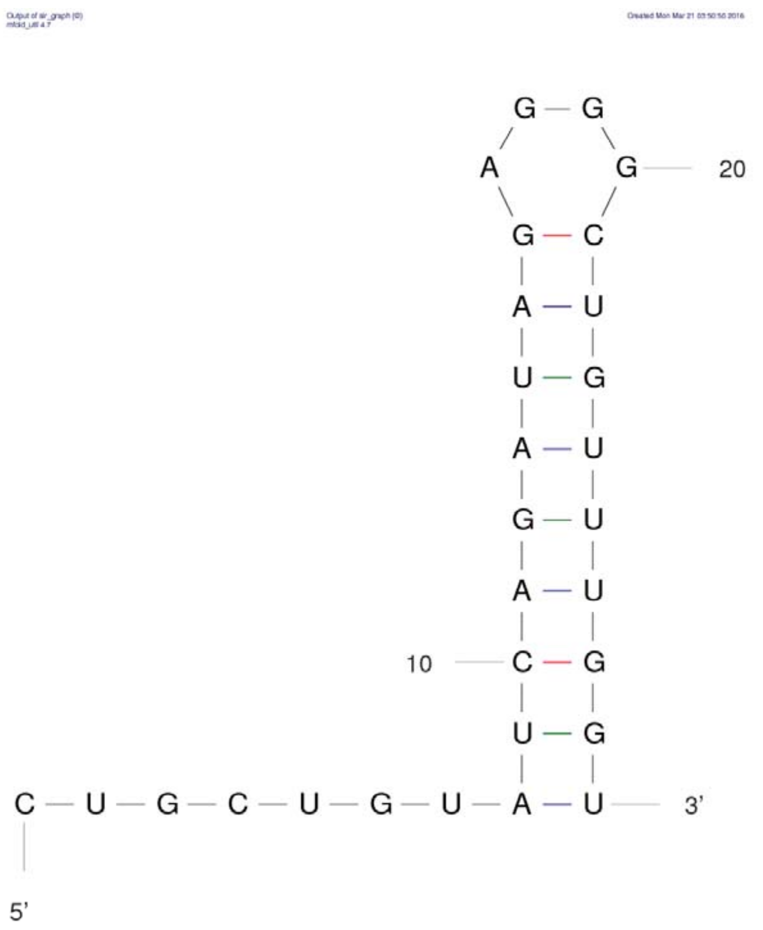

dG = -6.8 A_mallard duck_ALB_506_1983 H9N1

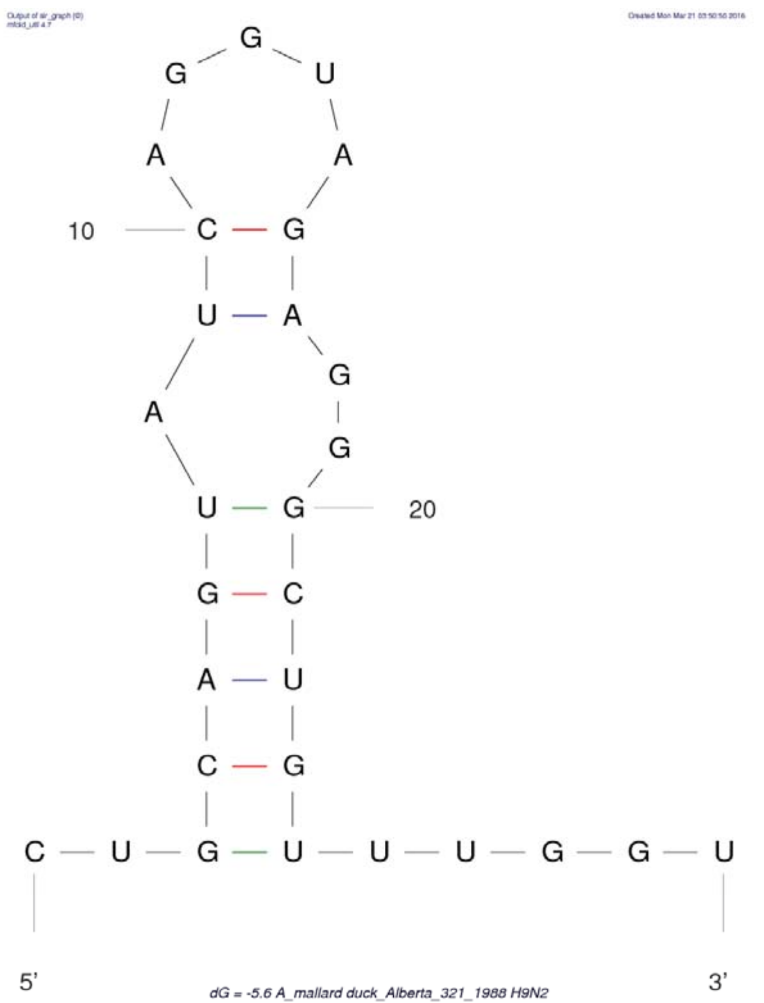

dG = -5.6 A_mallard duck_Alberta_321_1988 H9N2

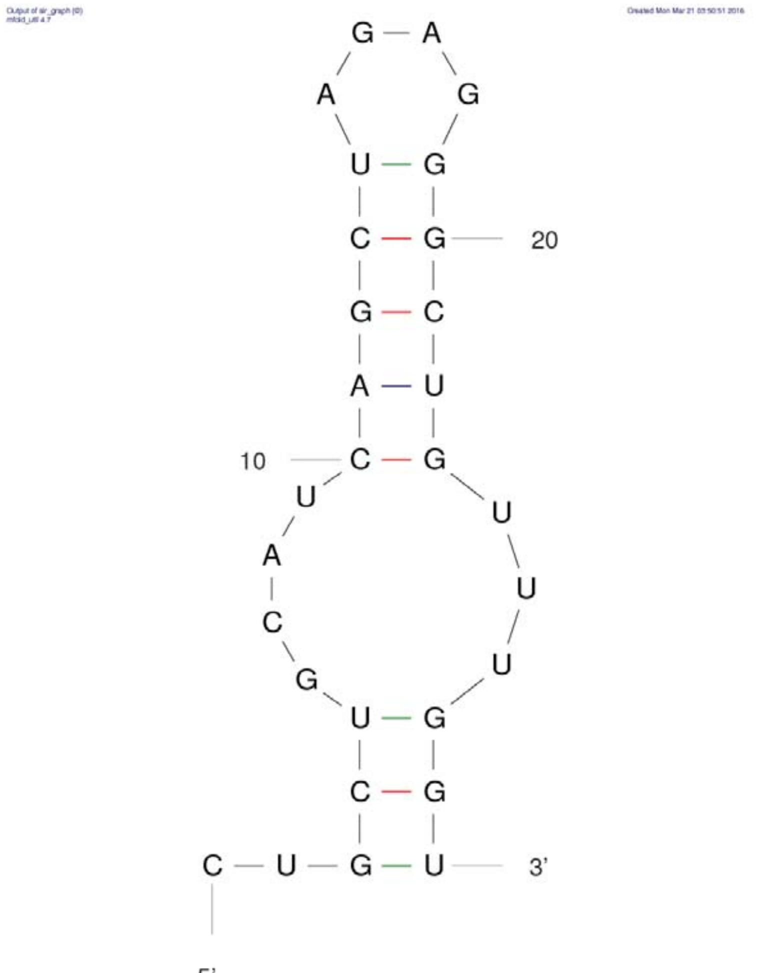

dG = -6.3 A_duck_Germany_113_1995 H9N2

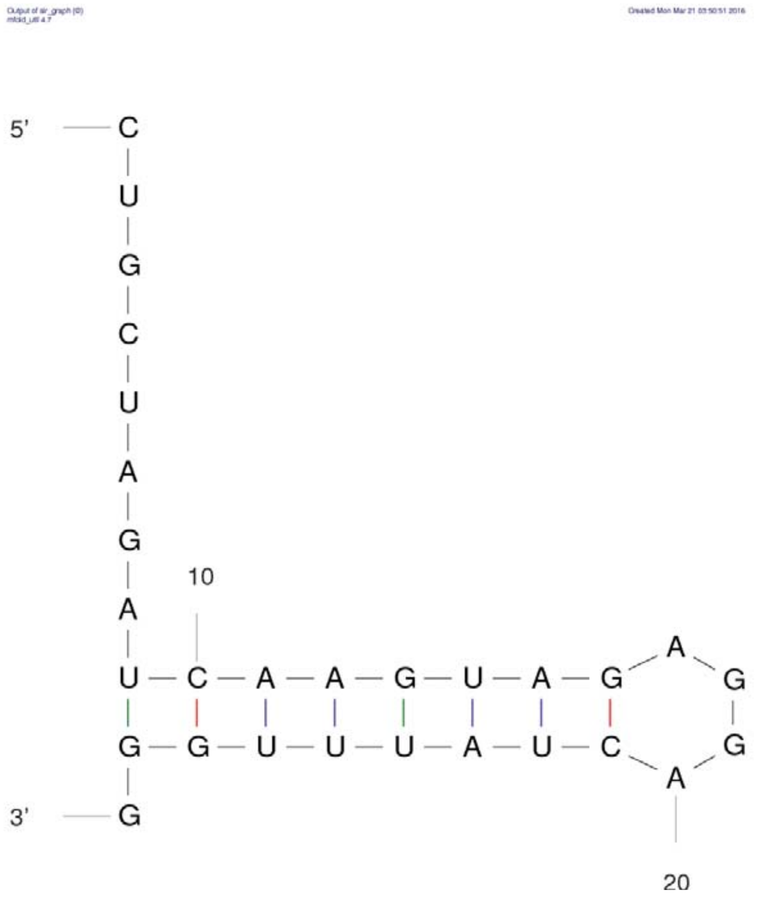

dG = -5.7 A_Muscovy duck_Fujian_CL_1997 H9N2

Output of sir_graph (©)
mfold_util 4.7

Created Mon Mar 21 03:50:51 2016

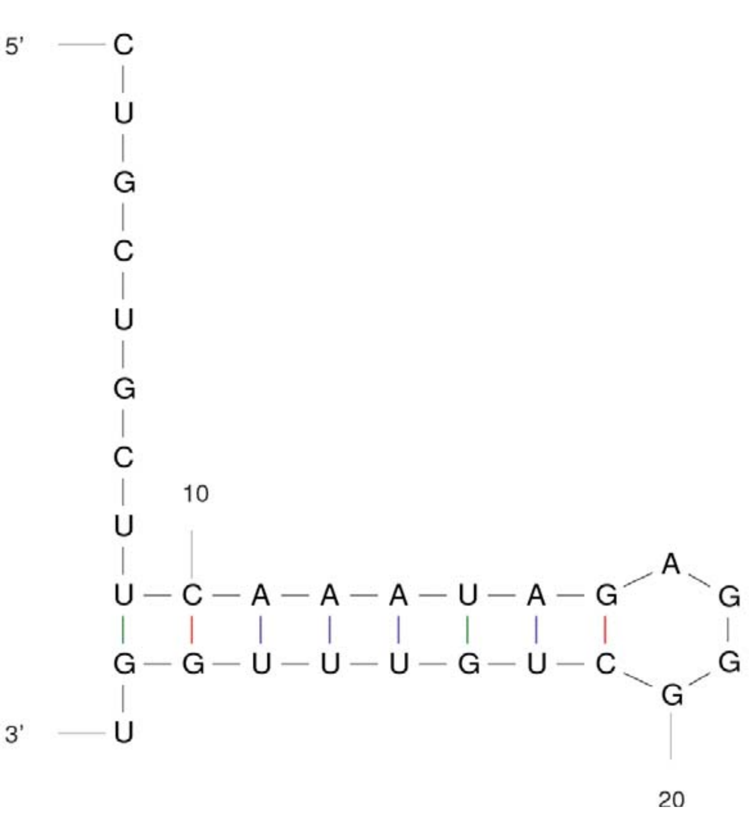

dG = -5.9 A_duck_Hong Kong_Y439_1997 H9N2

Output of sir_graph (©)
mfold_util 4.7

Created Mon Mar 21 03:50:51 2016

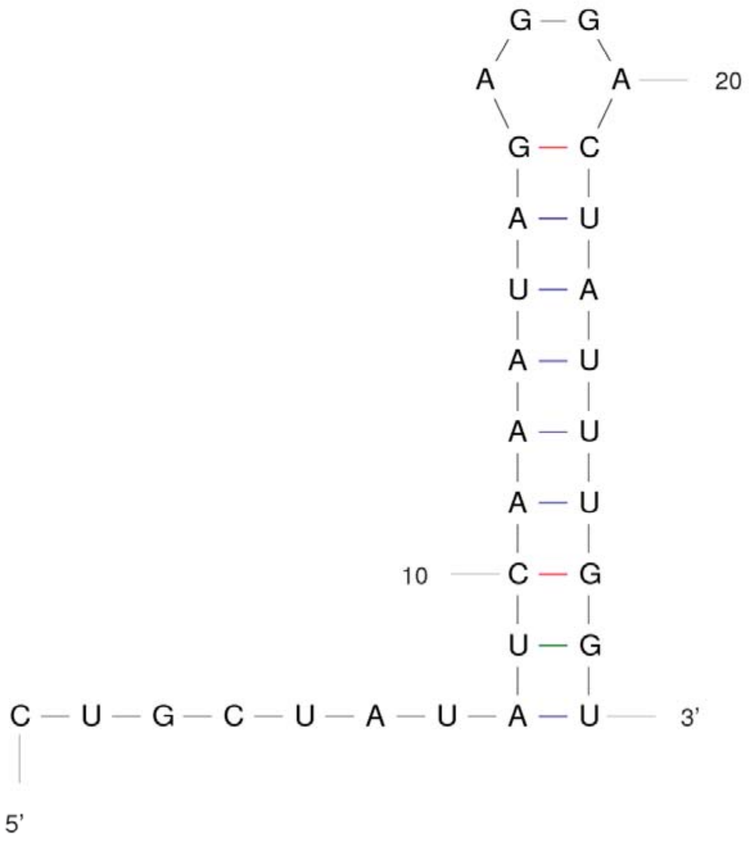

dG = -6.5 A_duck_Malaysia_91_1997 H9N2

Output of sir_graph (©)
mfold_util 4.7

Created Mon Mar 21 03:50:51 2016

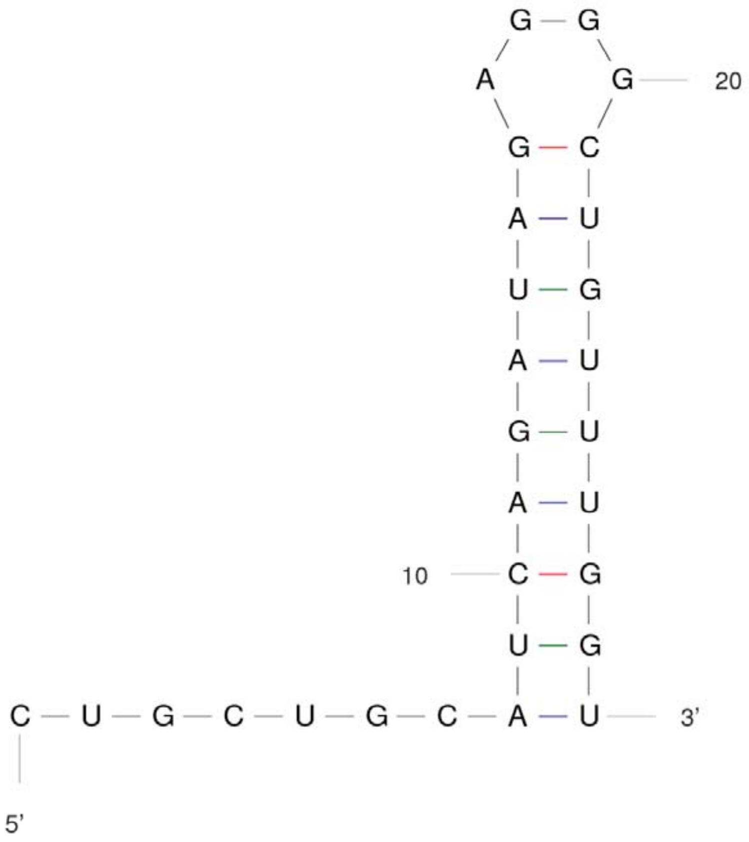

dG = -6.9 A_duck_Hokkaido_49_98 H9N2

Output of sir_graph (©)
mfold_util 4.7

Created Mon Mar 21 03:50:52 2016

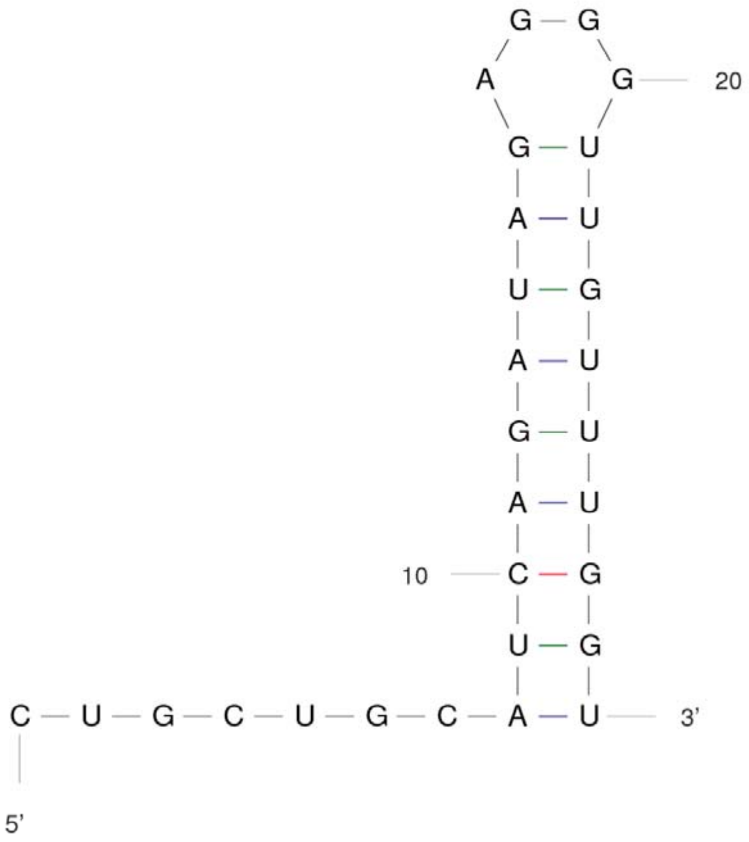

dG = -4.4 A_duck_Hokkaido_9_99 H9N2

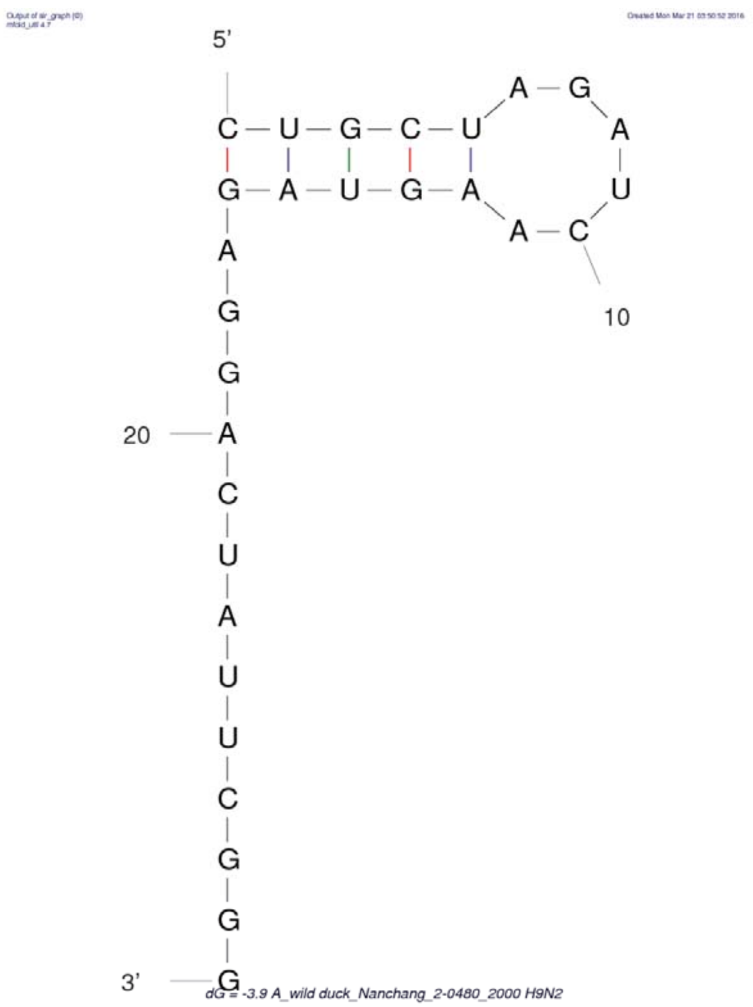

dG = -3.9 A_wild duck_Nanchang_2-0480_2000 H9N2

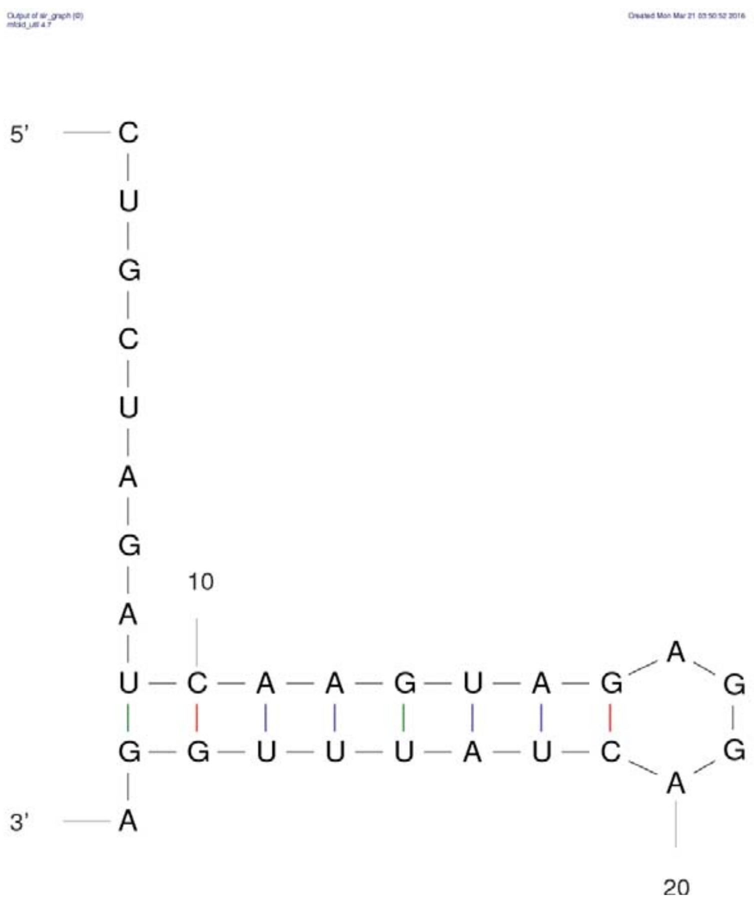

dG = -5.7 A_duck_Guangdong_GZ01_2001 H9N2

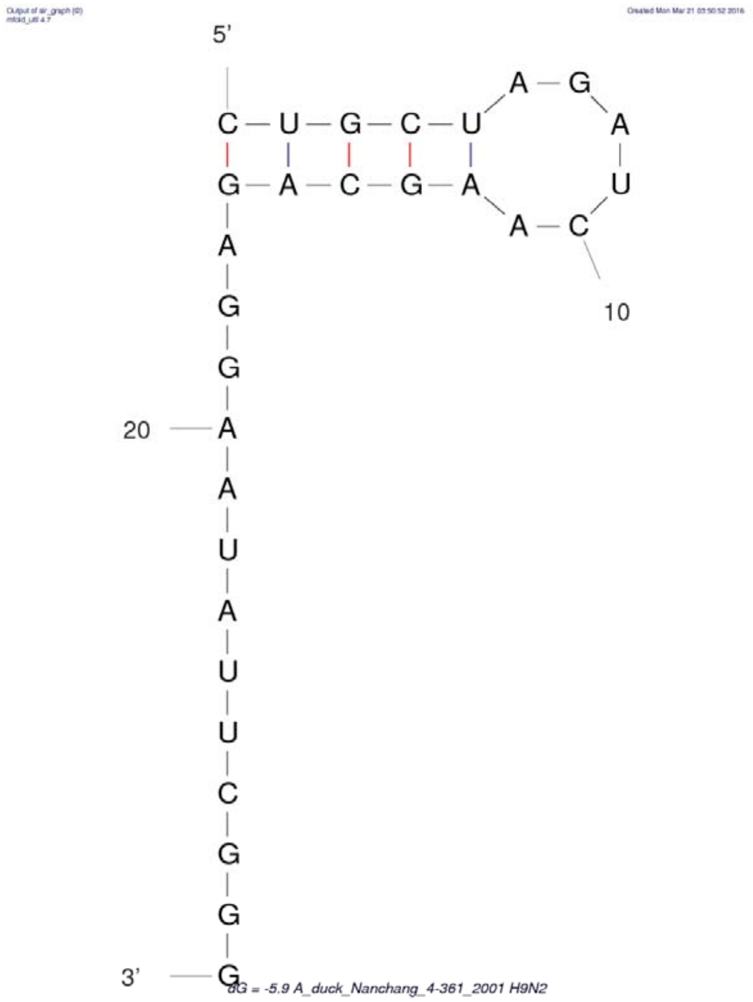

dG = -5.9 A_duck_Nanchang_4-361_2001 H9N2

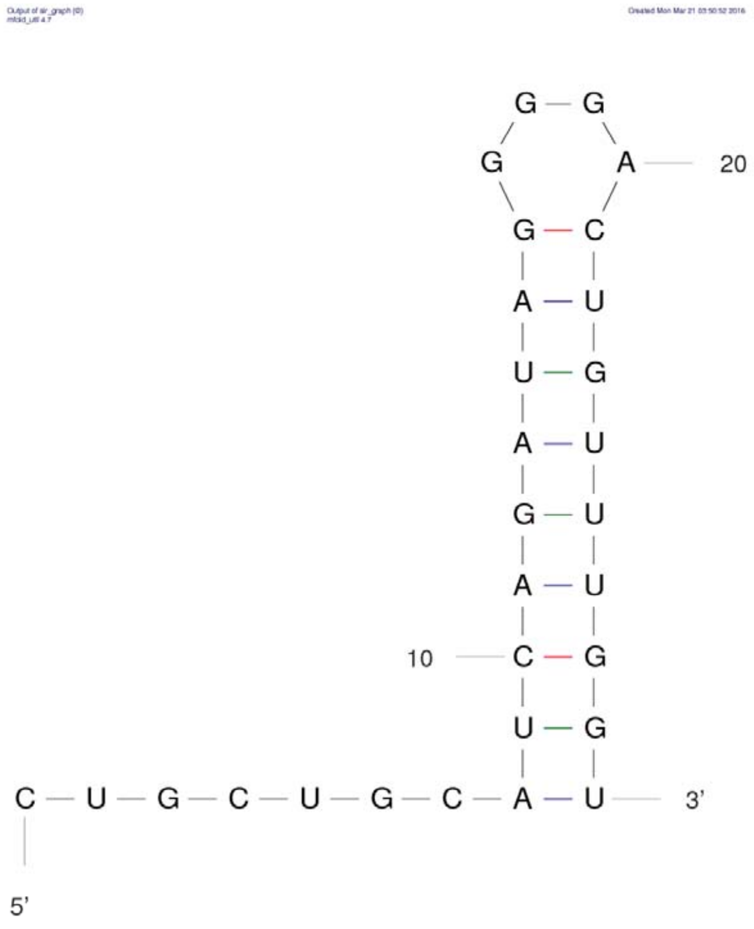

dG = -11 A_duck_Italy_260_2004 H9N8

Output of sir_graph (©)
mfold_util 4.7

Created Mon Mar 21 03:50:53 2016

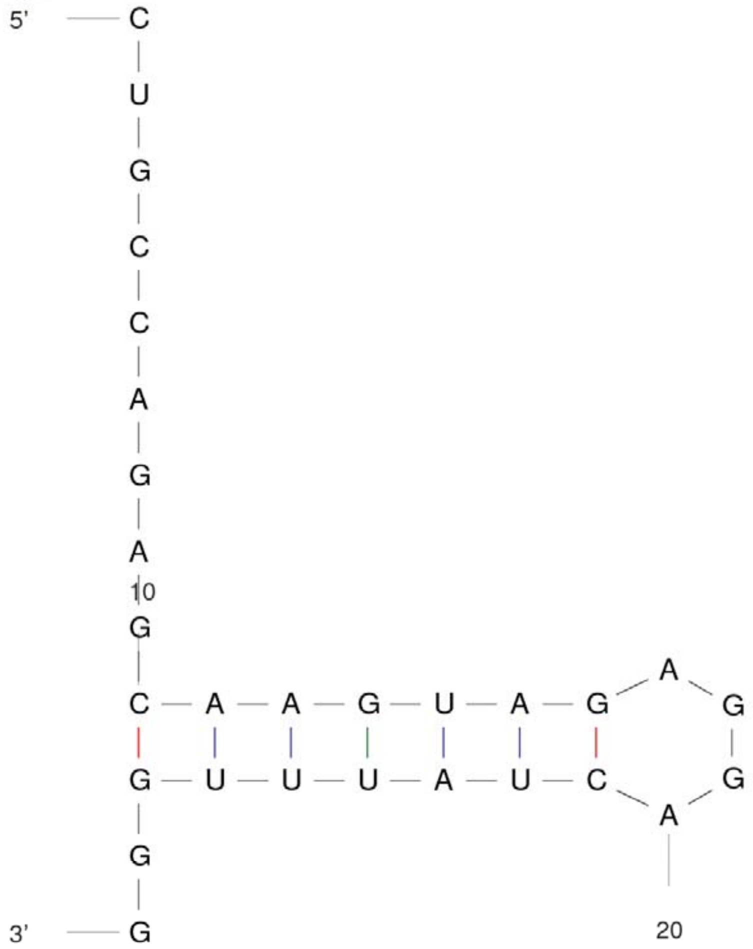

dG = -5.3 A_duck_Guangxi_51_2005 H9N2

Output of sir_graph (©)
mfold_util 4.7

Created Mon Mar 21 03:50:53 2016

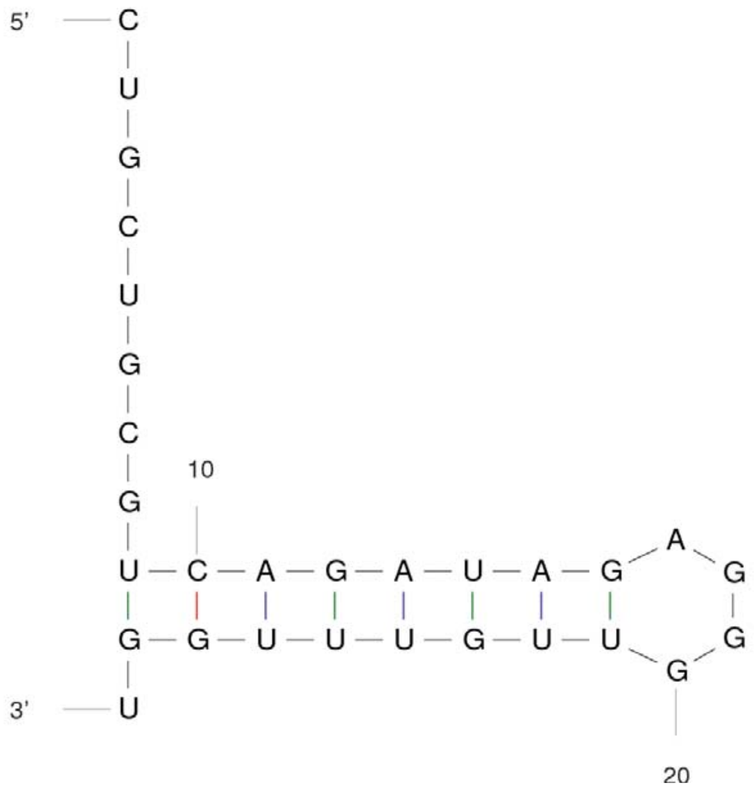

dG = -3.3 A_duck_Hokkaido_HY57_2005 H9N4

Output of sir_graph (©)
mfold_util 4.7

Created Mon Mar 21 03:50:53 2016

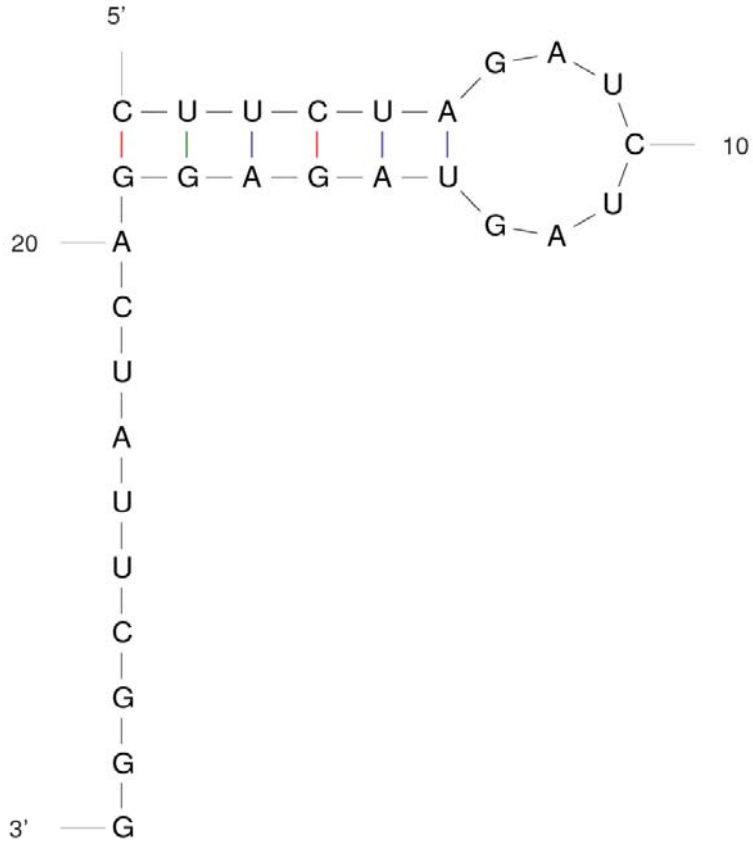

dG = -3.9 A_duck_Guangxi_NN_2006 H9N2

Output of sir_graph (©)
mfold_util 4.7

Created Mon Mar 21 03:50:53 2016

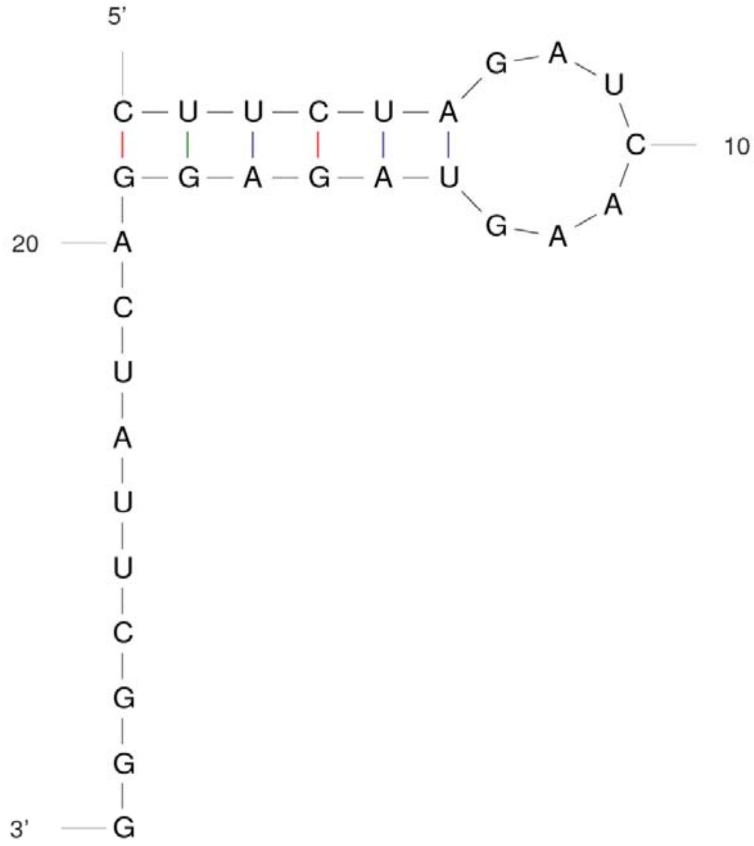

dG = -3.9 A_duck_Hunan_1_2006 H9N2

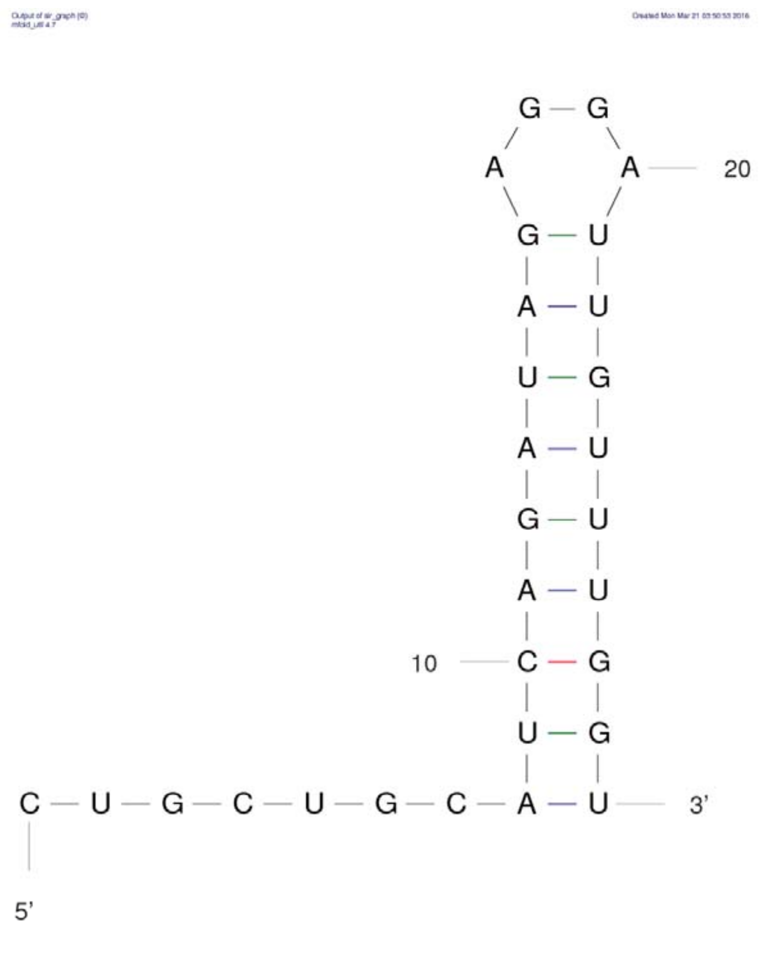

dG = -3.9 A_duck_Chiba_1_2007 H9N2

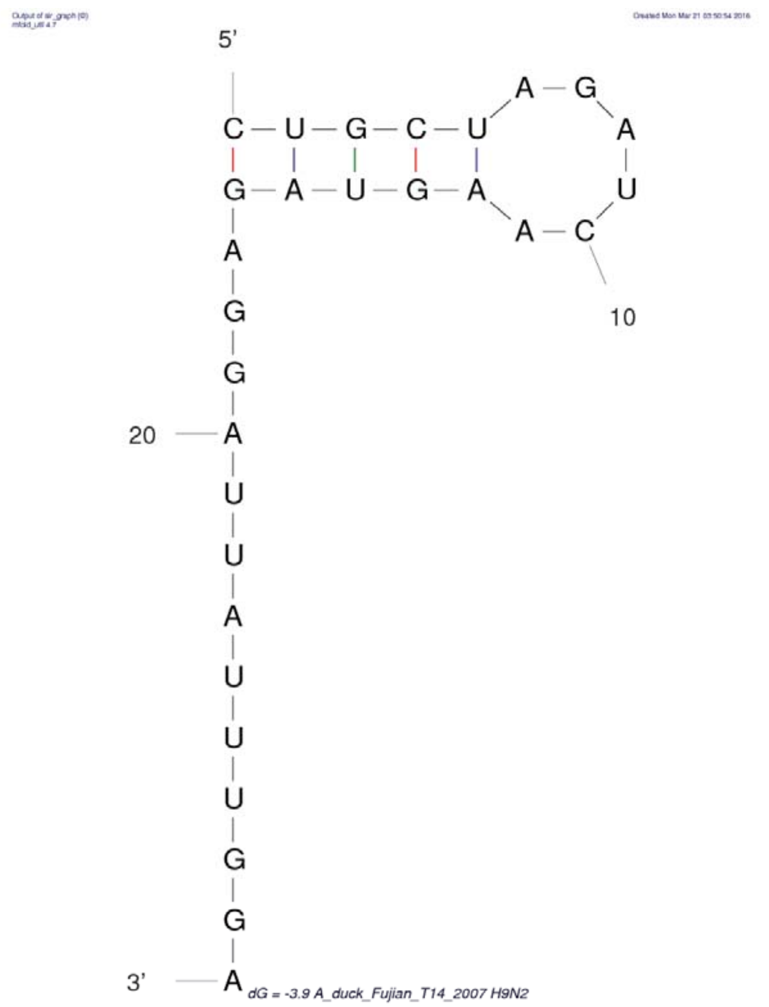

dG = -3.9 A_duck_Fujian_T14_2007 H9N2

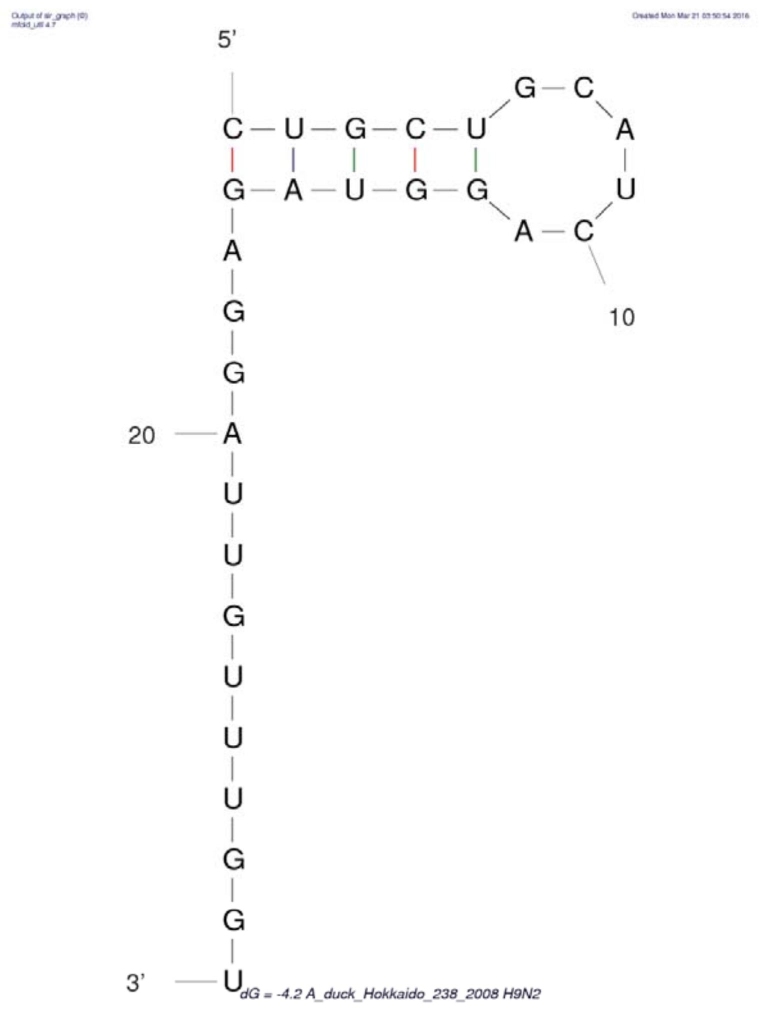

dG = -4.2 A_duck_Hokkaido_238_2008 H9N2

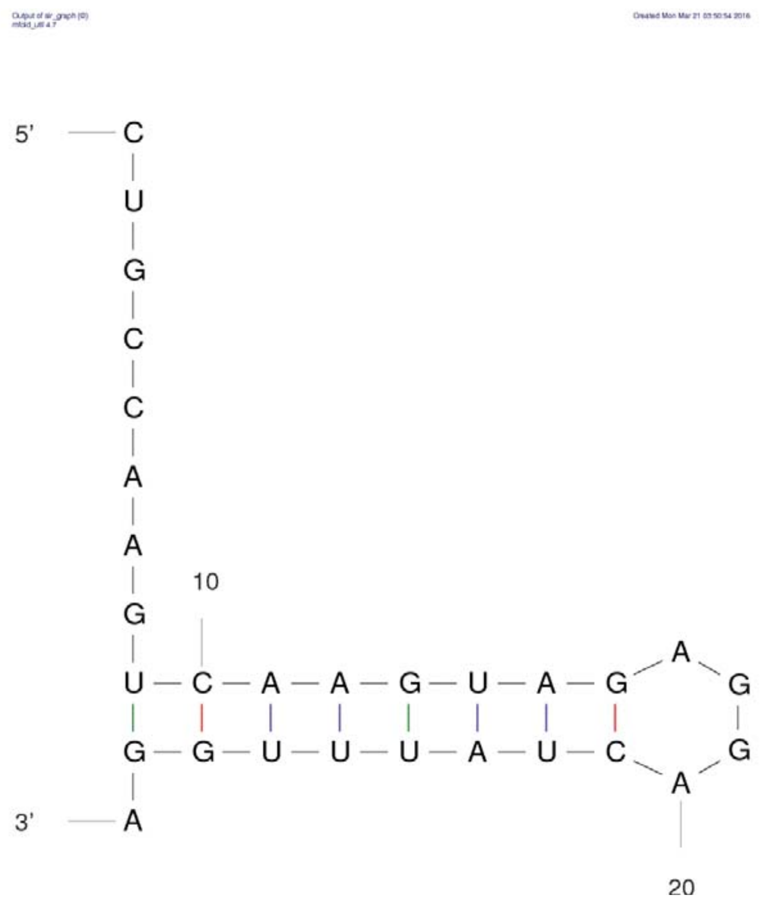

dG = -5.7 A_duck_Bangladesh_1231_2009 H9N2

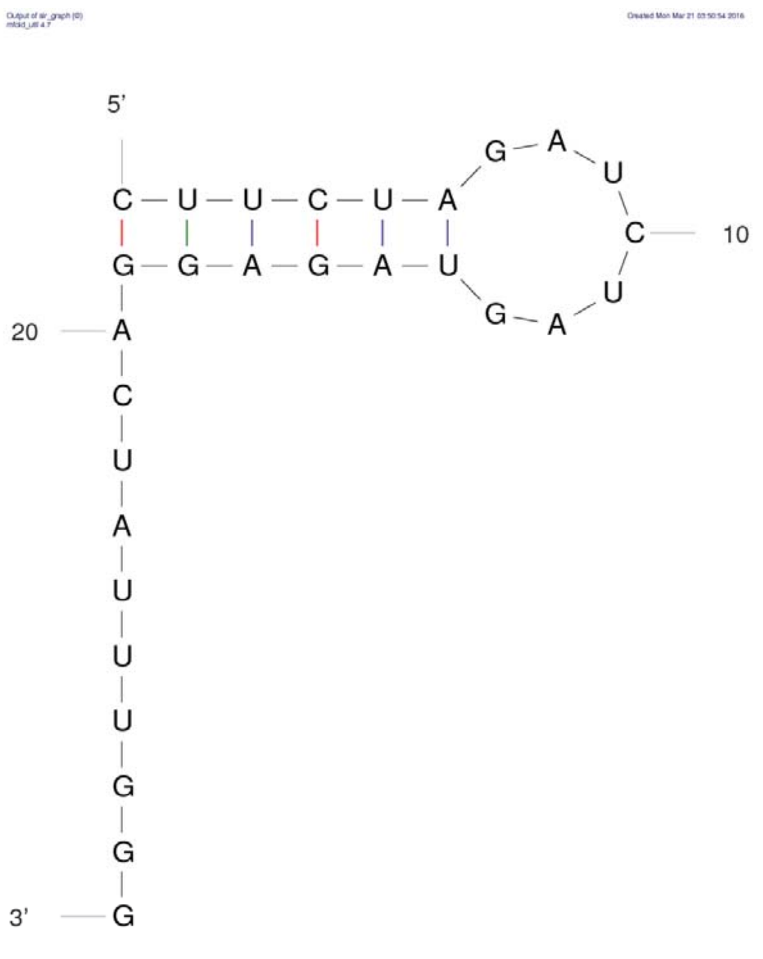

dG = -3.9 A_duck_Guangdong_810_2009 H9N2

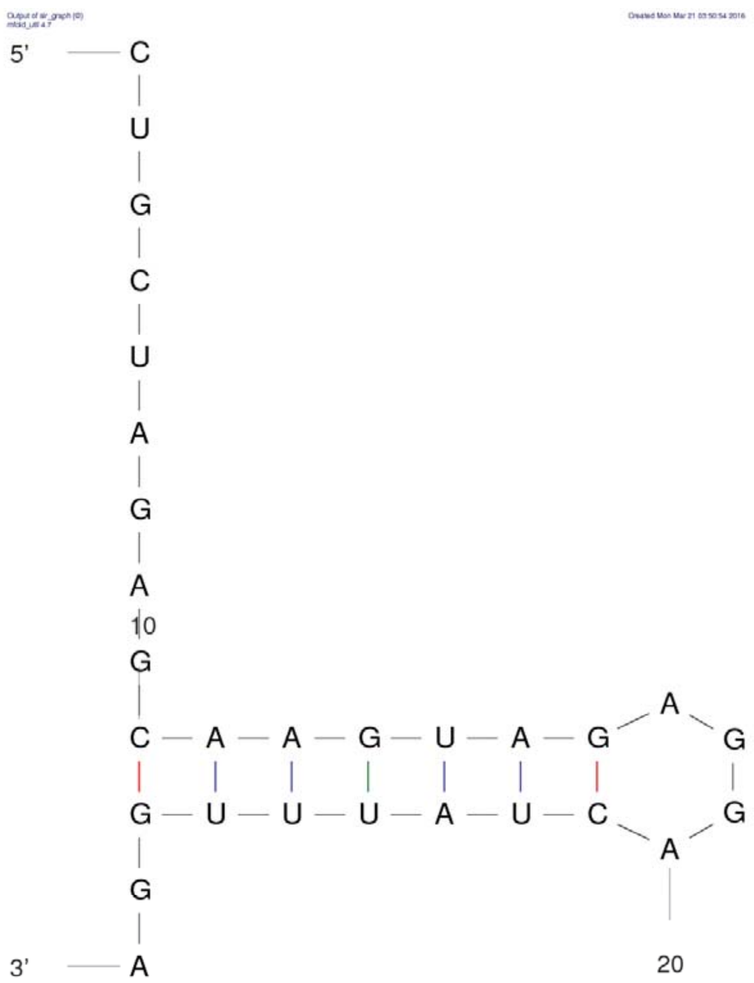

dG = -5.3 A_duck_Guangxi_LAD9_2009 H9N8

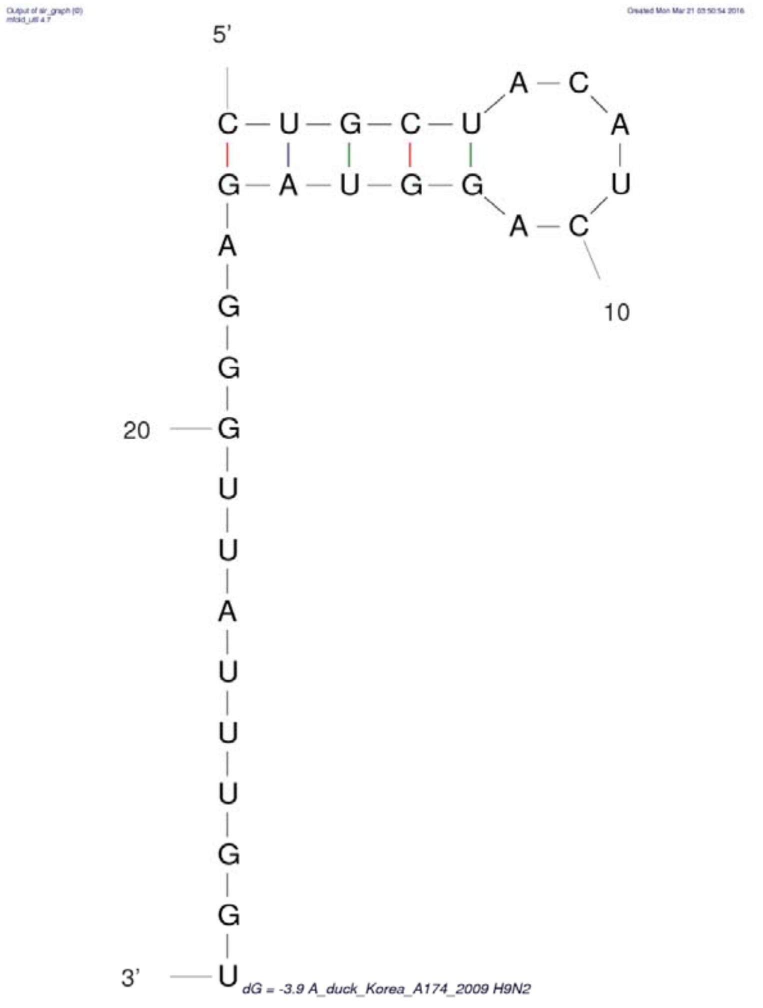

dG = -3.9 A_duck_Korea_A174_2009 H9N2

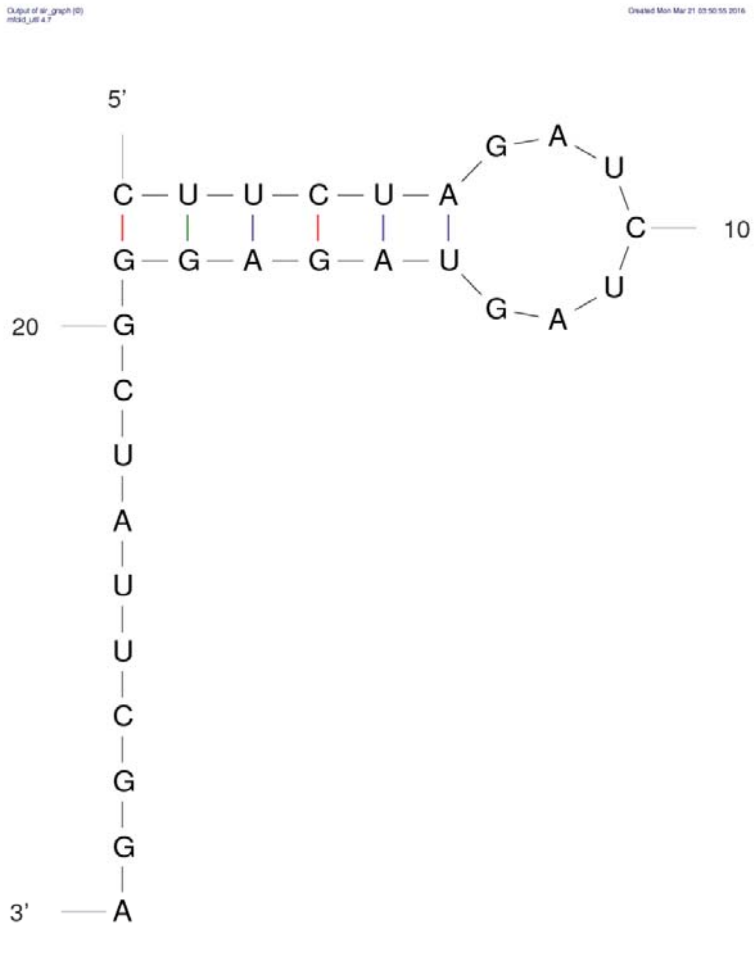

dG = -4.1 A_duck_Shanghai_C163_2009 H9N2

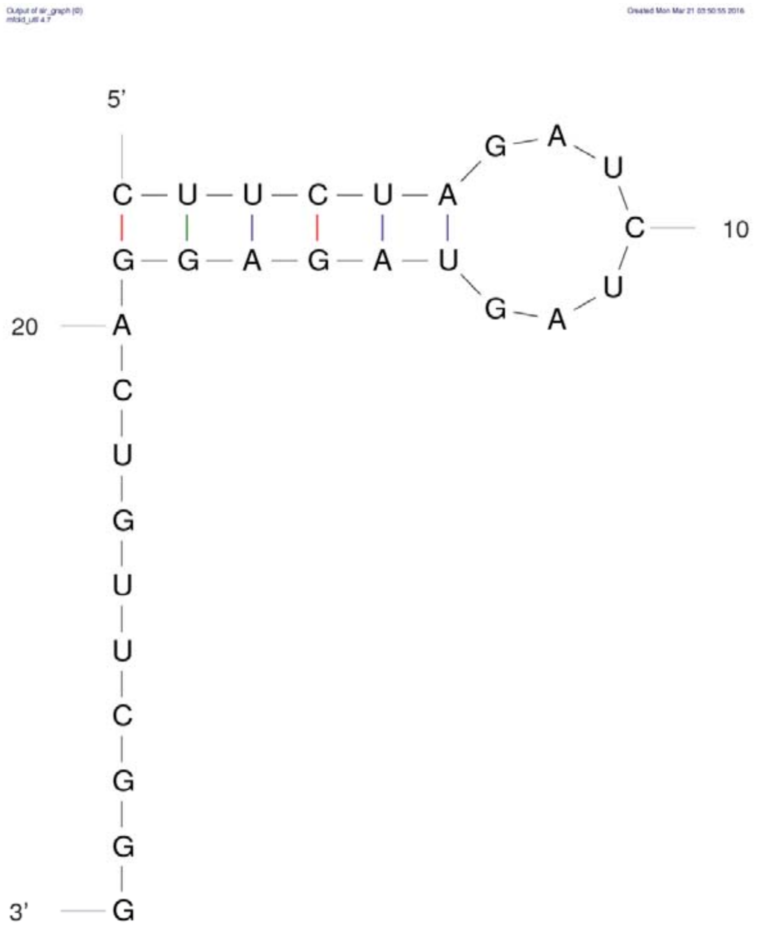

dG = -3.9 A_duck_Tibet_S2_2009 H9N2

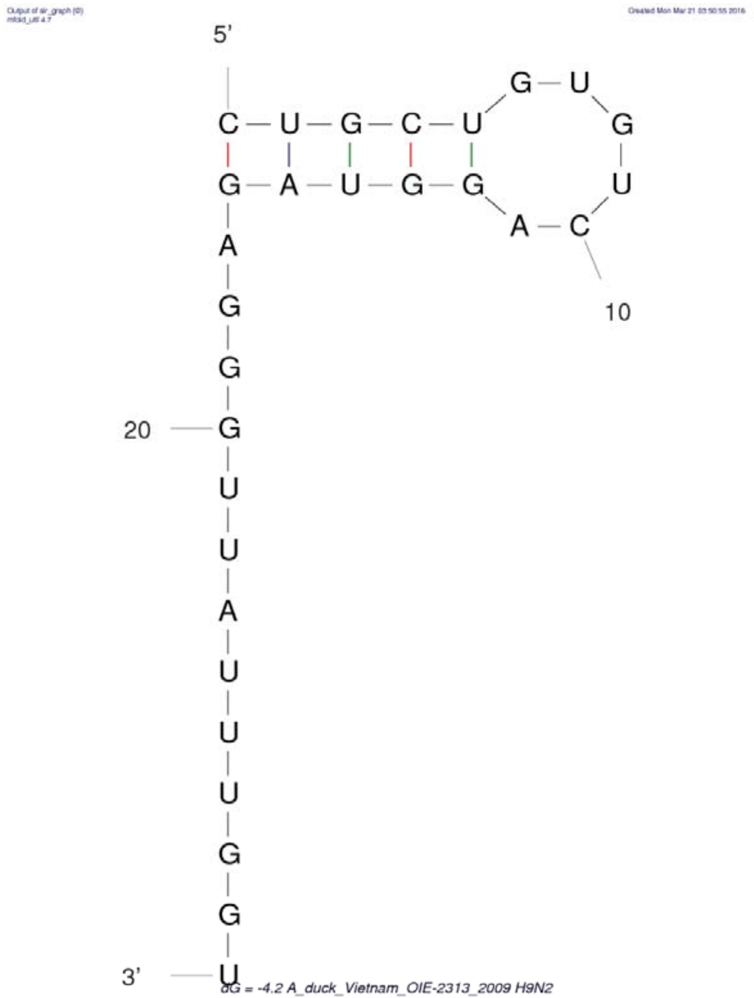

dG = -4.2 A_duck_Vietnam_OIE-2313_2009 H9N2

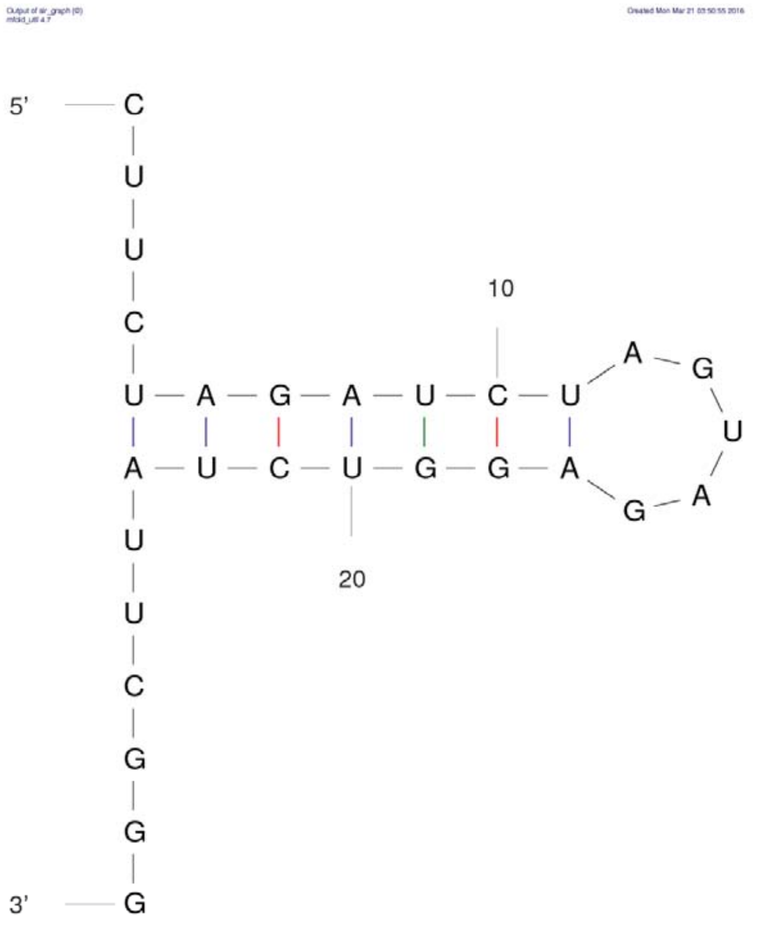

dG = -6.1 A_duck_Guangdong_G17_2010 H9N2

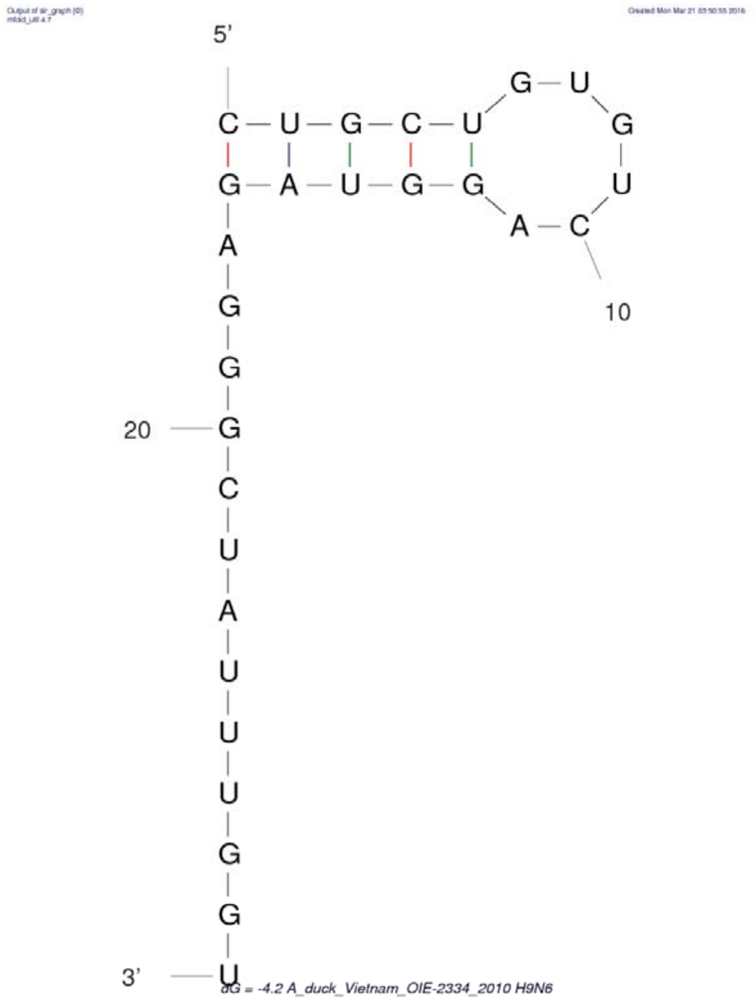

dG = -4.2 A_duck_Vietnam_OIE-2334_2010 H9N6

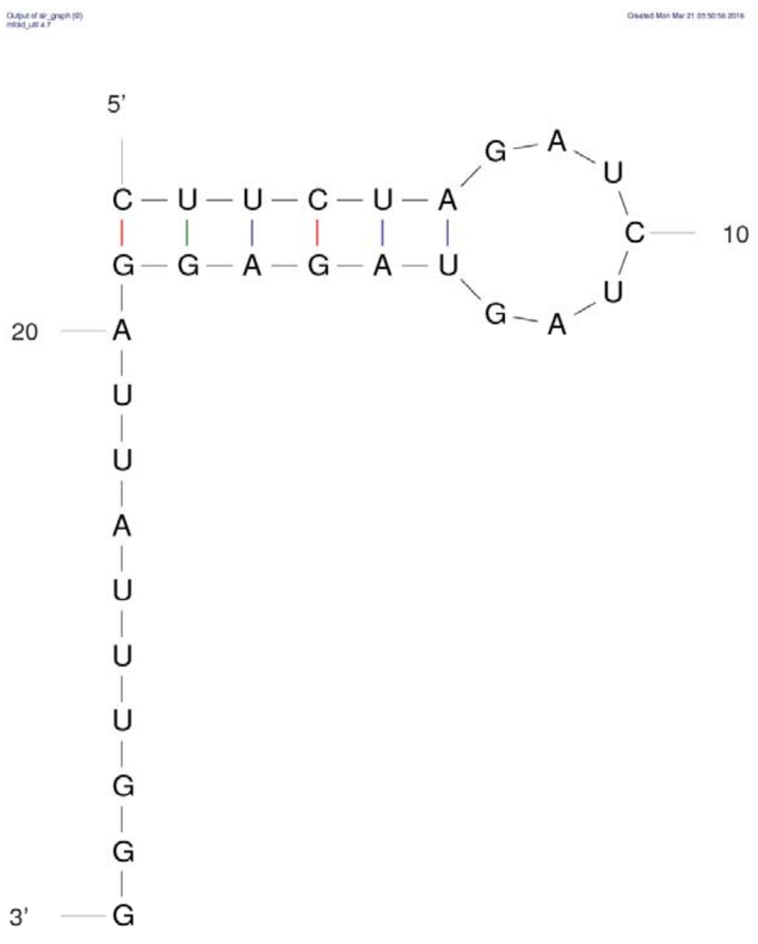

dG = -3.9 A_duck_Hubei_C1146_2011 H9N2

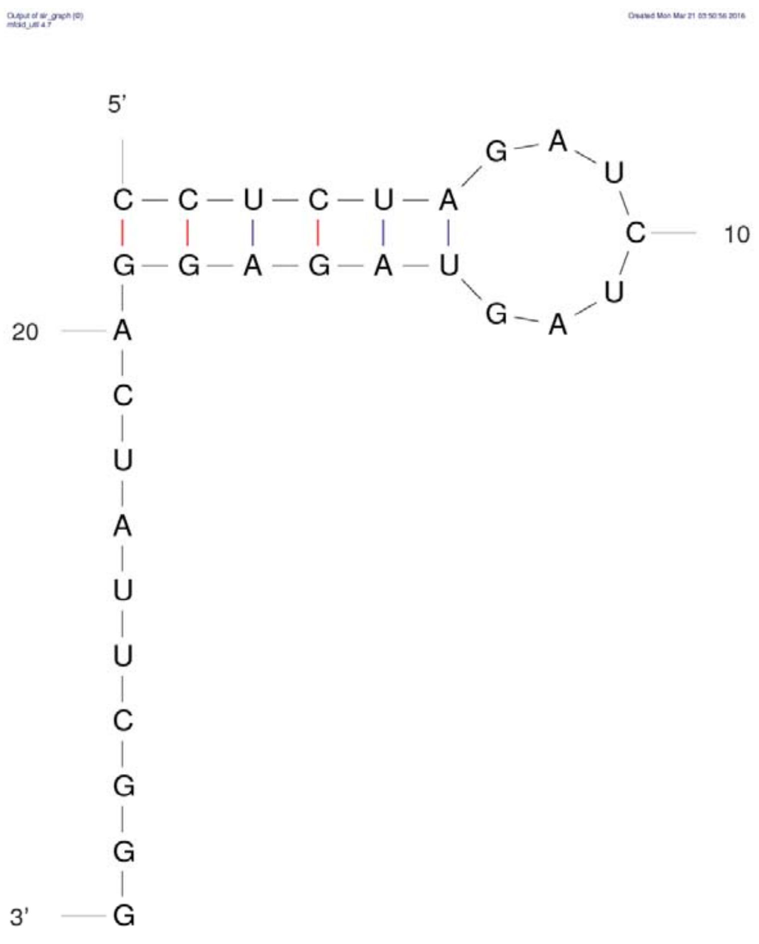

dG = -6.6 A_duck_Shandong_TA01_2012 H9N2

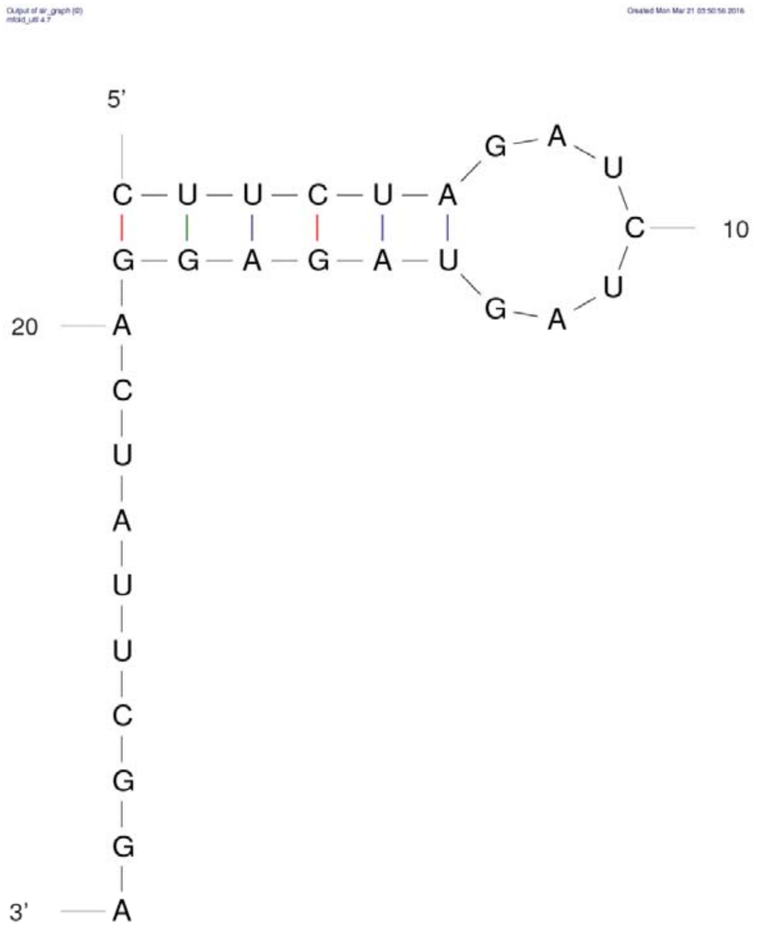

dG = -3.9 A_duck_Zhejiang_Z42_2012 H9

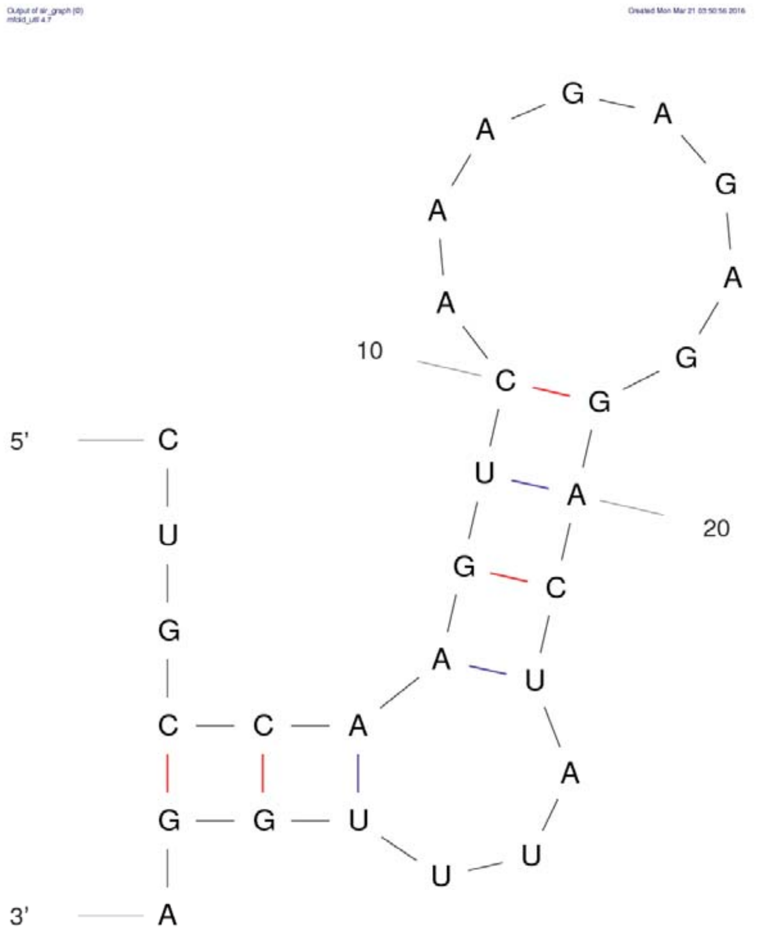

dG = -5 A_duck_Bangladesh_21126_2013 H9N2

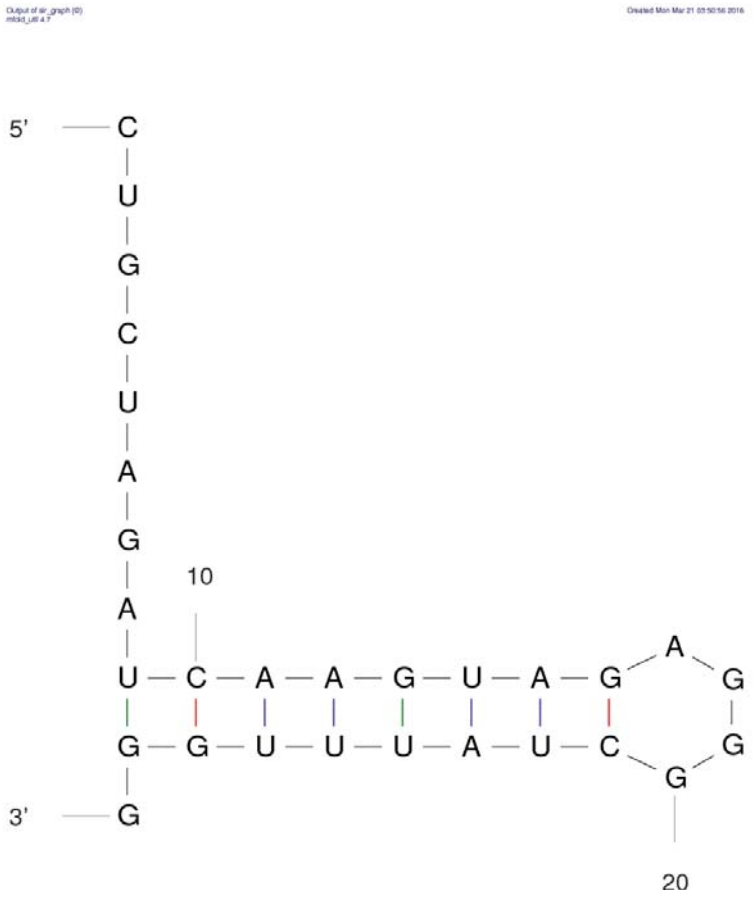

dG = -5.9 A_duck_Jiangsu_KS0802_2013 H9N2

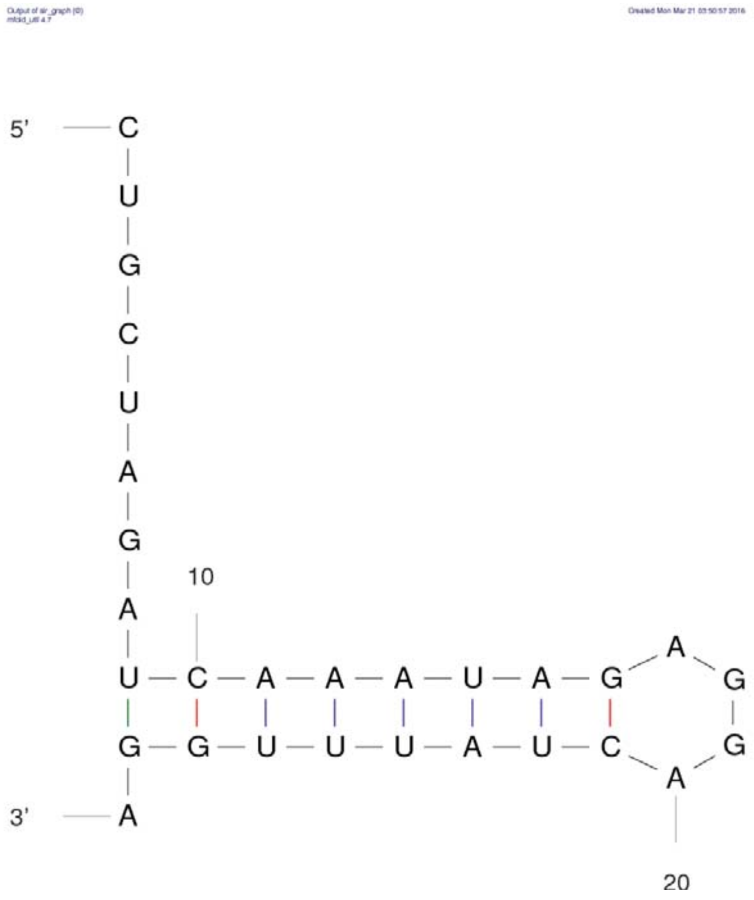

dG = -5.7 A_muscovy duck_Vietnam_LBM417_2013 H9N2

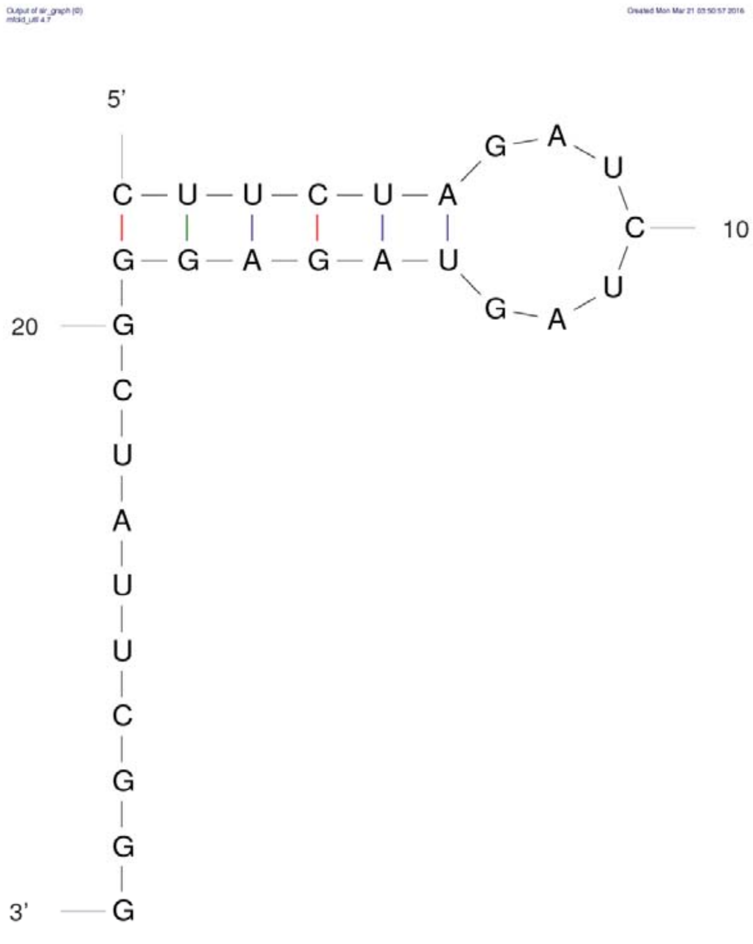

dG = -4.1 A_duck_Guangdong_G2259_2014 H9

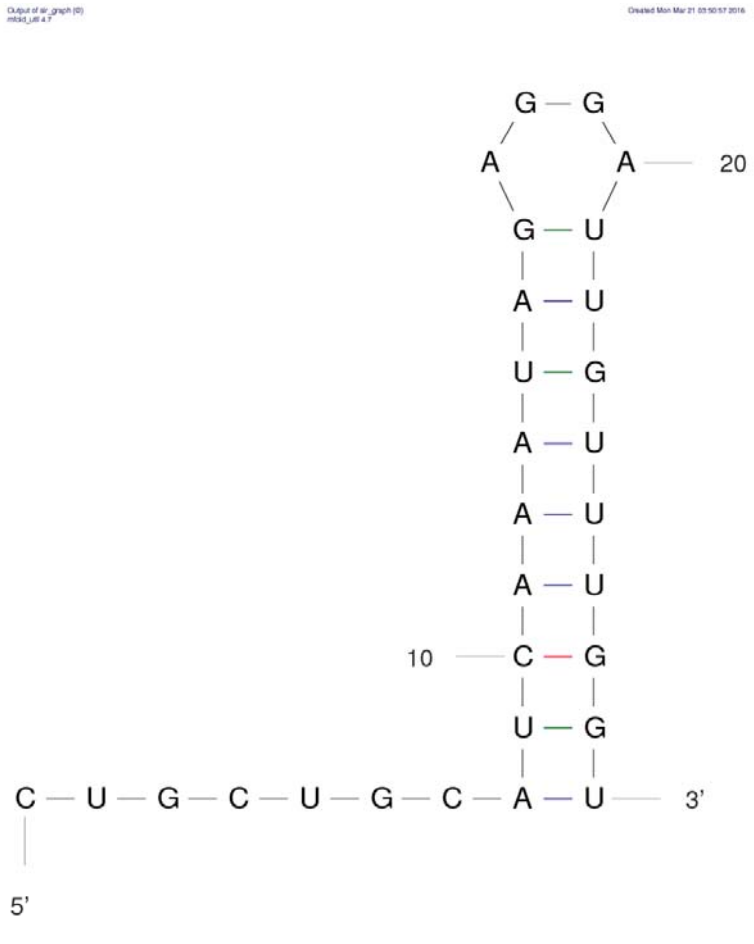

dG = -3.8 A_duck_Wuhan_WHYF05_2014 H9N2

Output of sir_graph (©)
mfold_util 4.7

Created Mon Mar 21 04:17:07 2016

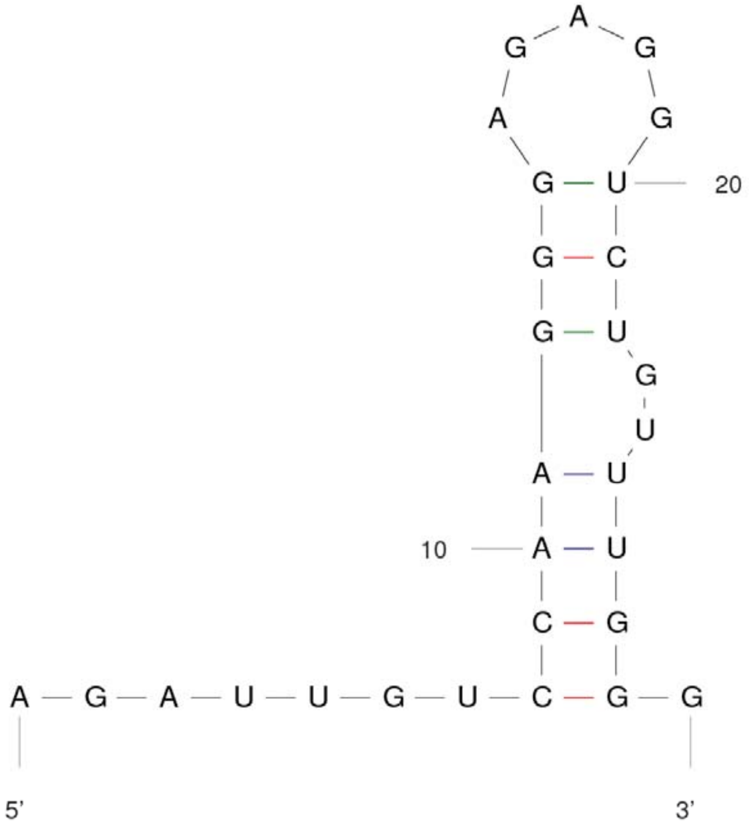

dG = -4.3 A_duck_Manitoba_1953 H10N7

Output of sir_graph (©)
mfold_util 4.7

Created Mon Mar 21 04:17:07 2016

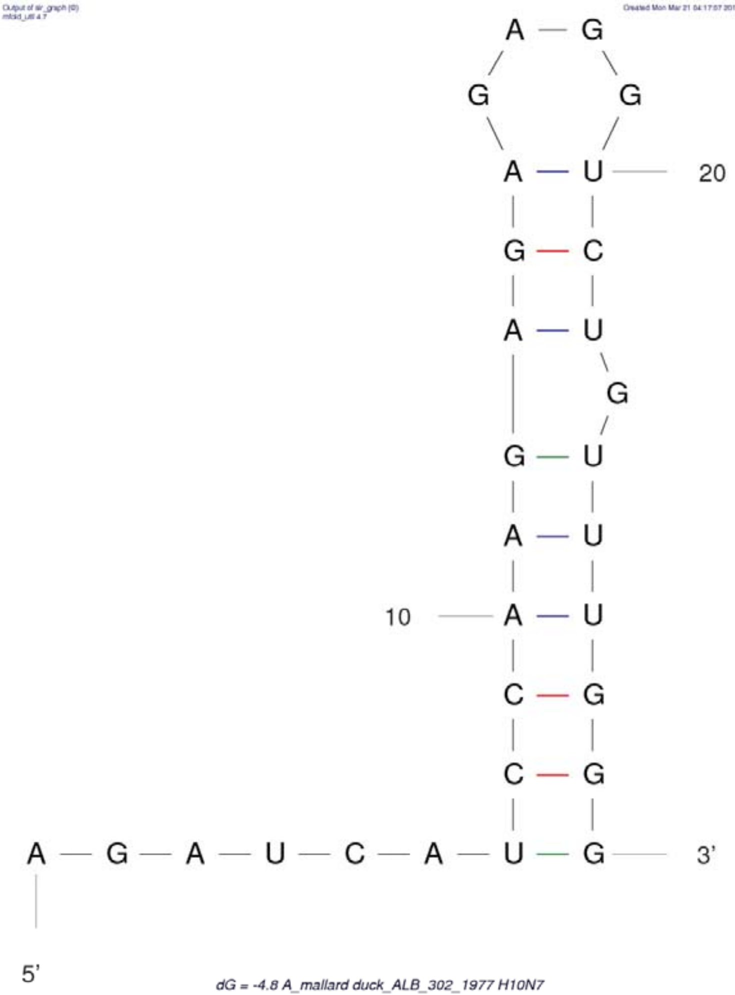

dG = -4.8 A_mallard duck_ALB_302_1977 H10N7

Output of sir_graph (©)
mfold_util 4.7

Created Mon Mar 21 04:17:07 2016

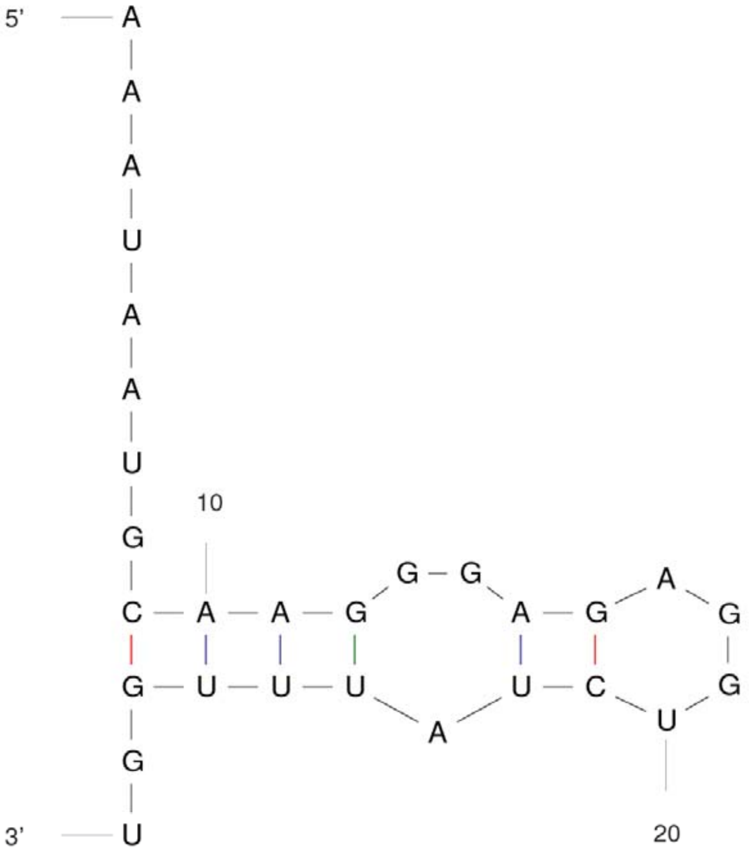

dG = -1.7 A_duck_Hong Kong_562_1979 H10N9

Output of sir_graph (©)
mfold_util 4.7

Created Mon Mar 21 04:17:08 2016

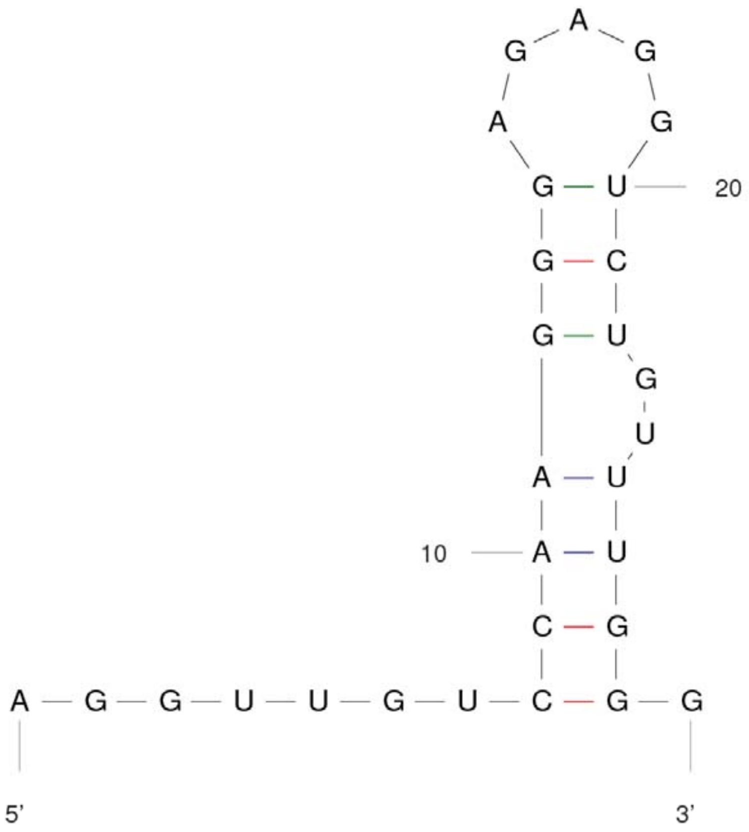

dG = -4.3 A_mallard duck_Minnesota_19_1979 H10N7

Output of sir_graph (©)
mfold_util 4.7

Created Mon Mar 21 04:17:08 2016

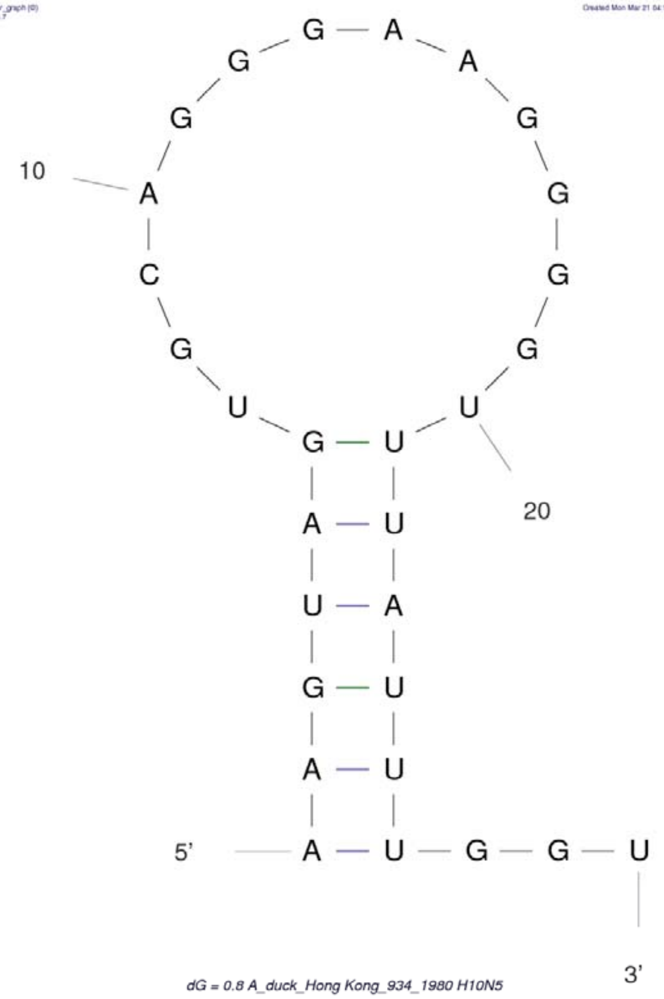

dG = 0.8 A_duck_Hong Kong_934_1980 H10N5

Output of sir_graph (©)
mfold_util 4.7

Created Mon Mar 21 04:17:08 2016

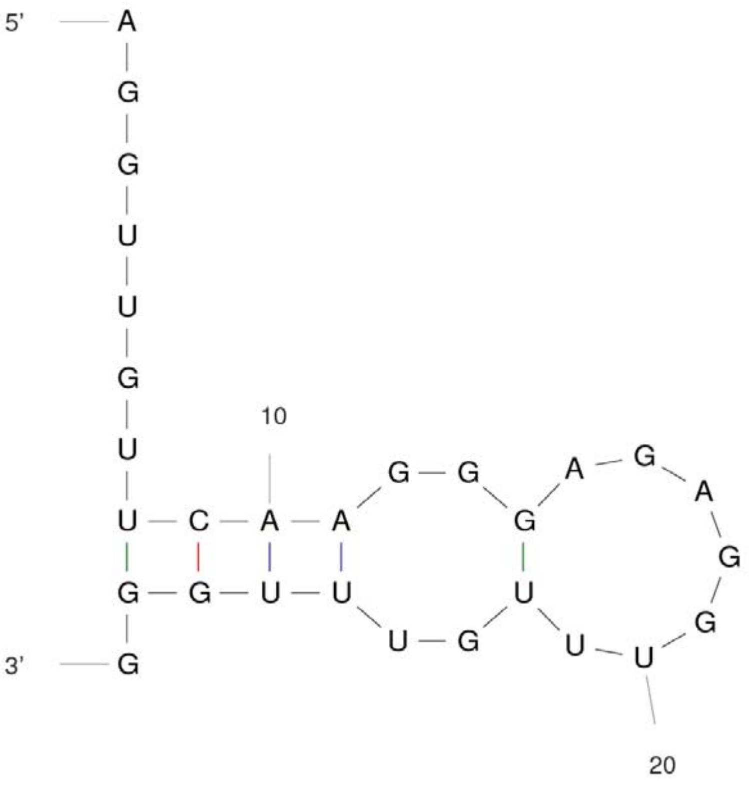

dG = 0.3 A_duck_Wisconsin_2368_1985 H10N3

Output of sir_graph (©)
mfold_util 4.7

Created Mon Mar 21 04:17:08 2016

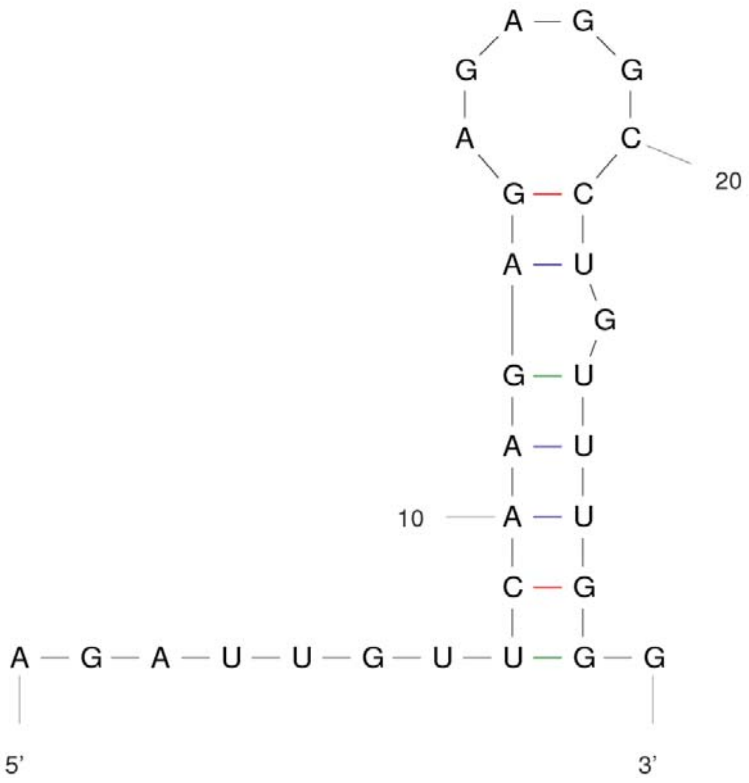

dG = -1.1 A_duck_Alaska_658_1991 H10N7

Output of sir_graph (©)
mfold_util 4.7

Created Mon Mar 21 04:17:08 2016

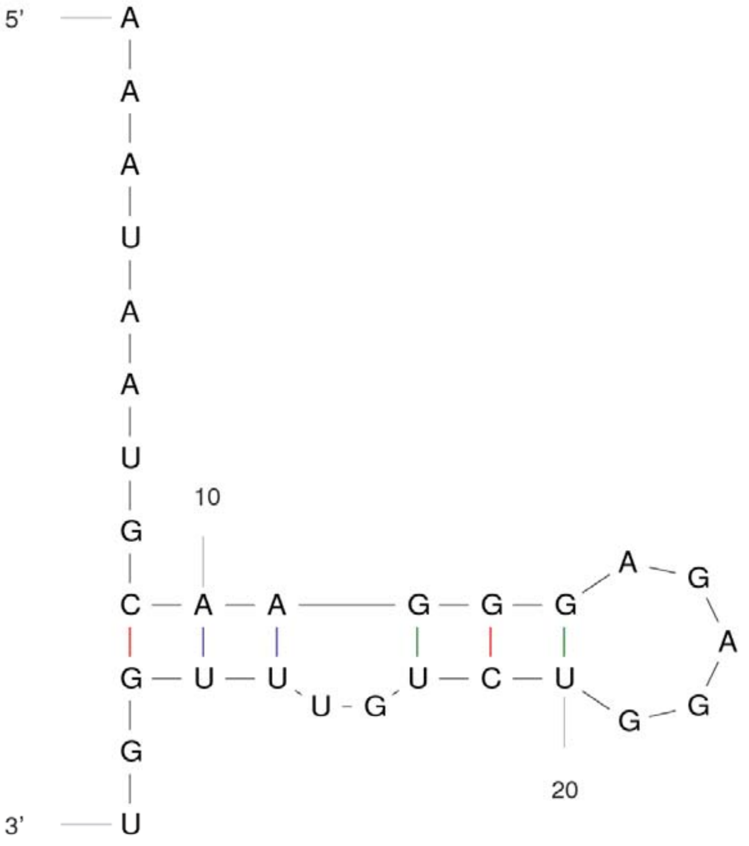

dG = -1.1 A_duck_Hokkaido_18_00 H10N4

Output of sir_graph (©)
mfold_util 4.7

Created Mon Mar 21 04:17:09 2016

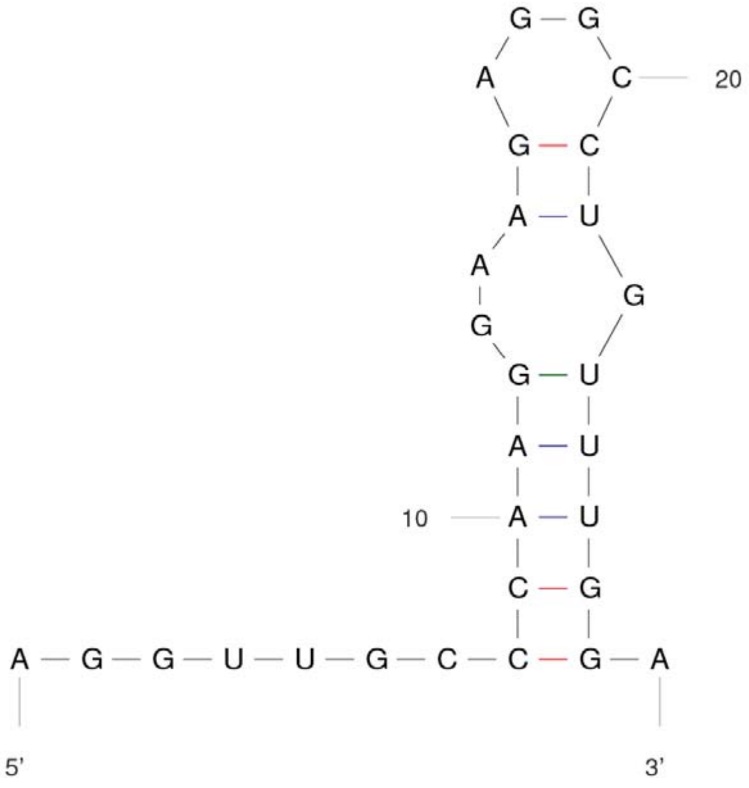

dG = -4.1 A_longtail duck_Maryland_295_2005 H10N8

Output of sir_graph (©)
mfold_util 4.7

Created Mon Mar 21 04:17:09 2016

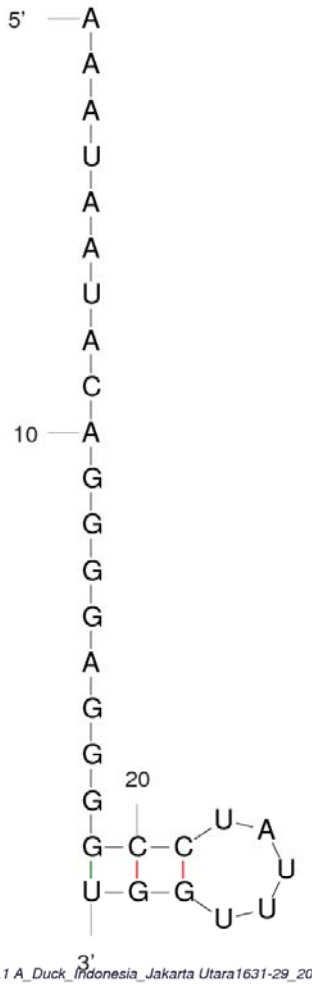

dG = -2.1 A_Duck_Indonesia_Jakarta Utara1631-29_2006 H10

Output of sir_graph (©)
mfold_util 4.7

Created Mon Mar 21 04:17:09 2016

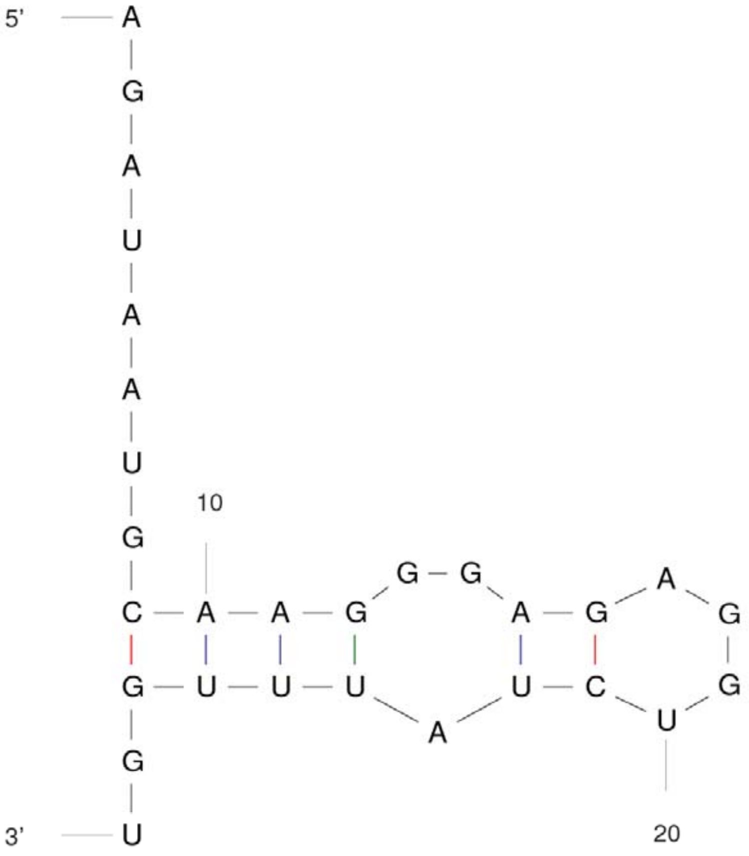

dG = -1.7 A_duck_Tsukuba_574_2006 H10N1

Output of sir_graph (©)
mfold_util 4.7

Created Mon Mar 21 04:17:09 2016

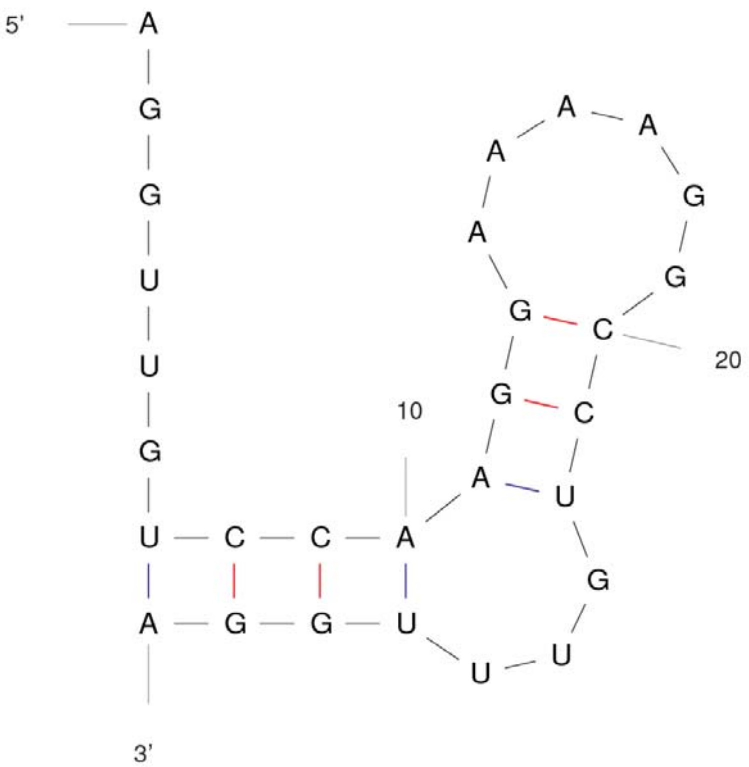

dG = -4.6 A_ring-necked duck_Michigan_588_2006 mixedH10

Output of sir_graph (©)
mfold_util 4.7

Created Mon Mar 21 04:17:09 2016

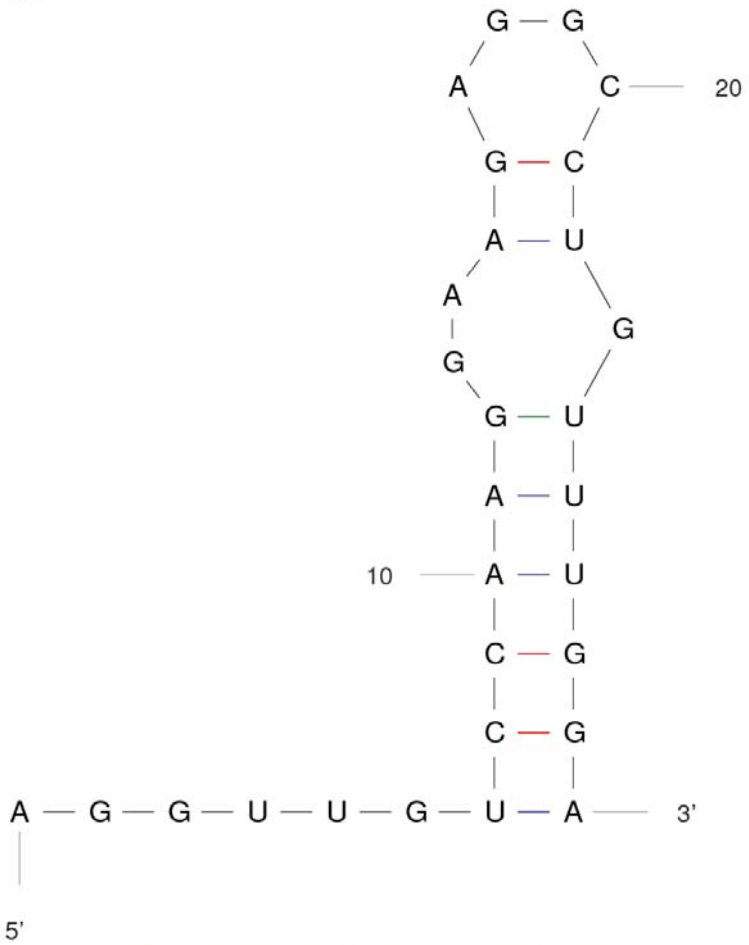

dG = -4.7 A_American black duck_New Brunswick_04395_2007 H10N7

Output of sir_graph (©)
mfold_util 4.7

Created Mon Mar 21 04:17:10 2016

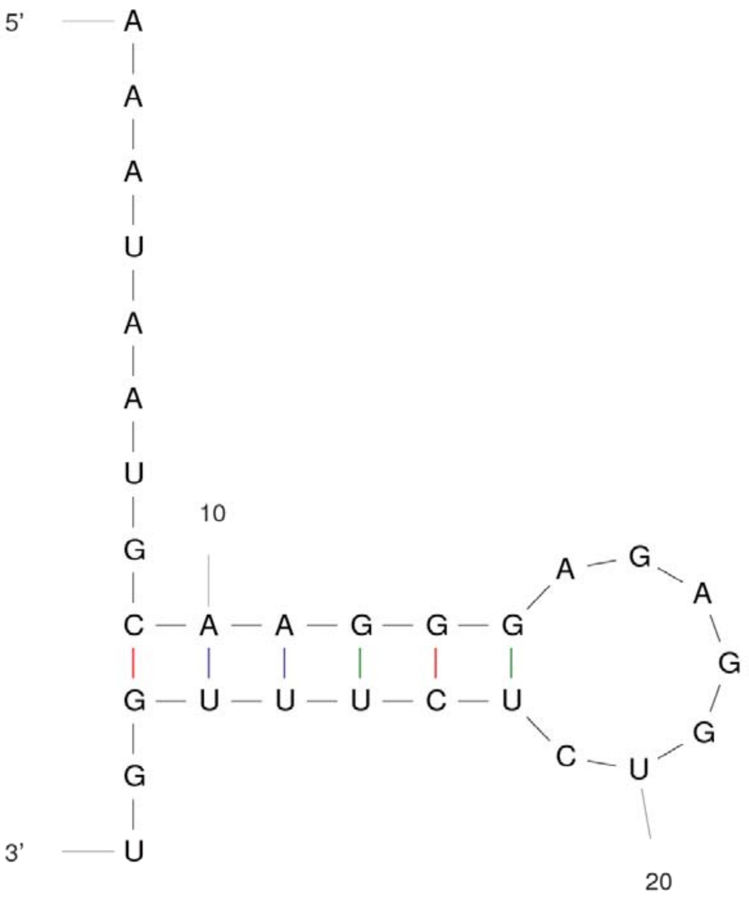

dG = -5.4 A_duck_Hokkaido_W87_2007 H10N2

Output of sir_graph (©)
mfold_util 4.7

Created Mon Mar 21 04:17:10 2016

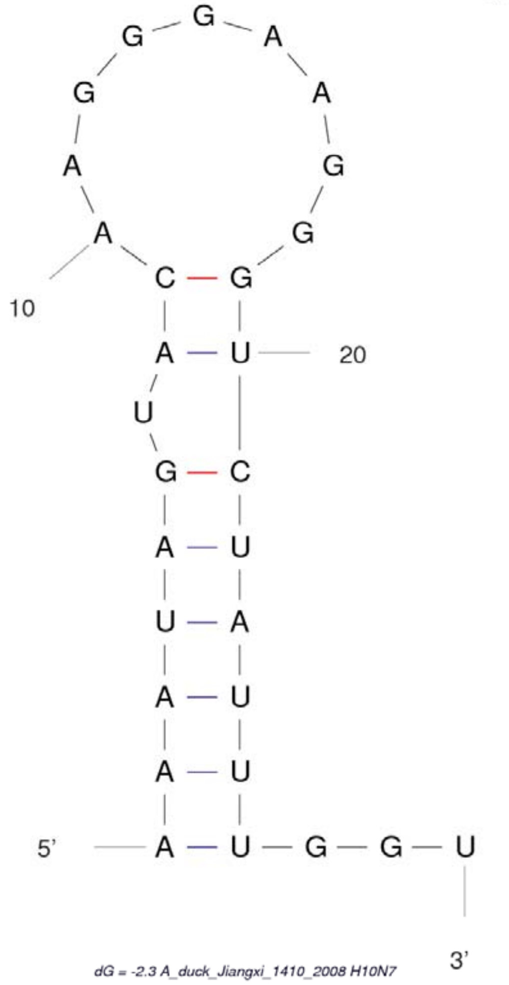

dG = -2.3 A_duck_Jiangxi_1410_2008 H10N7

Output of sir_graph (©)
mfold_util 4.7

Created Mon Mar 21 04:17:10 2016

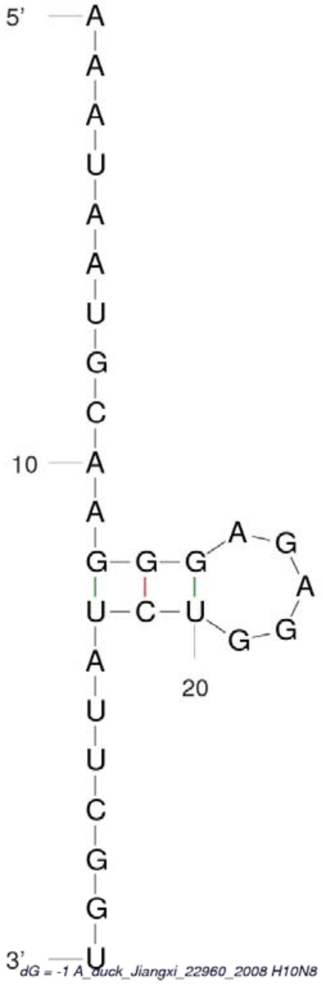

dG = -1 A_duck_Jiangxi_22960_2008 H10N8

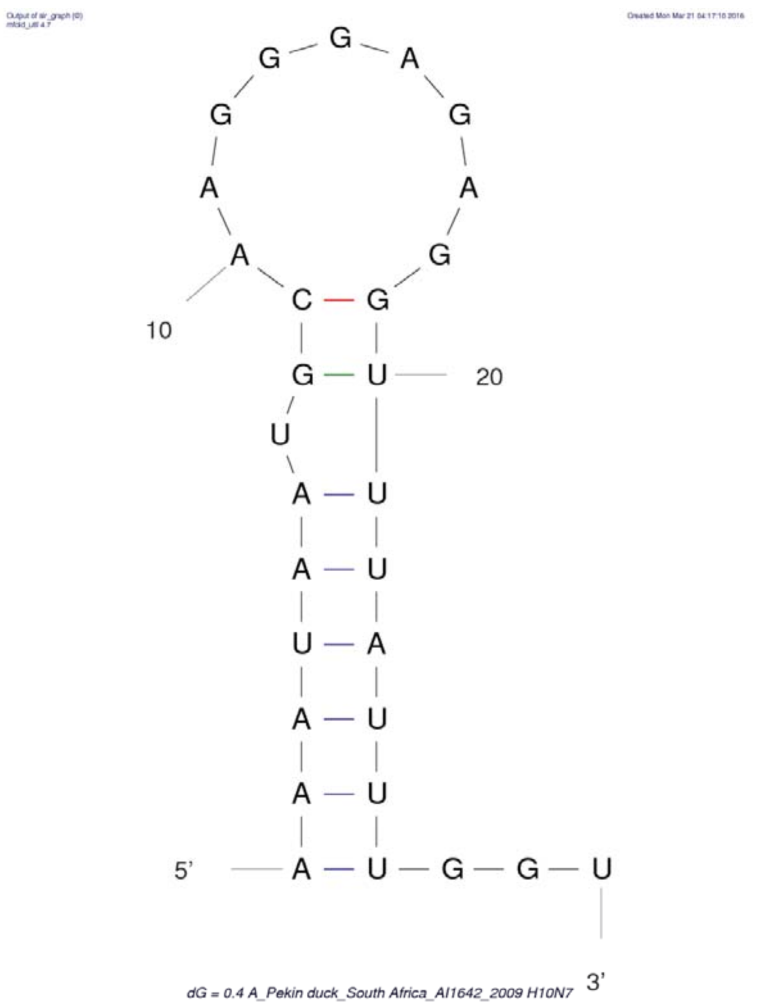

dG = 0.4 A_Pekin duck_South Africa_AI1642_2009 H10N7

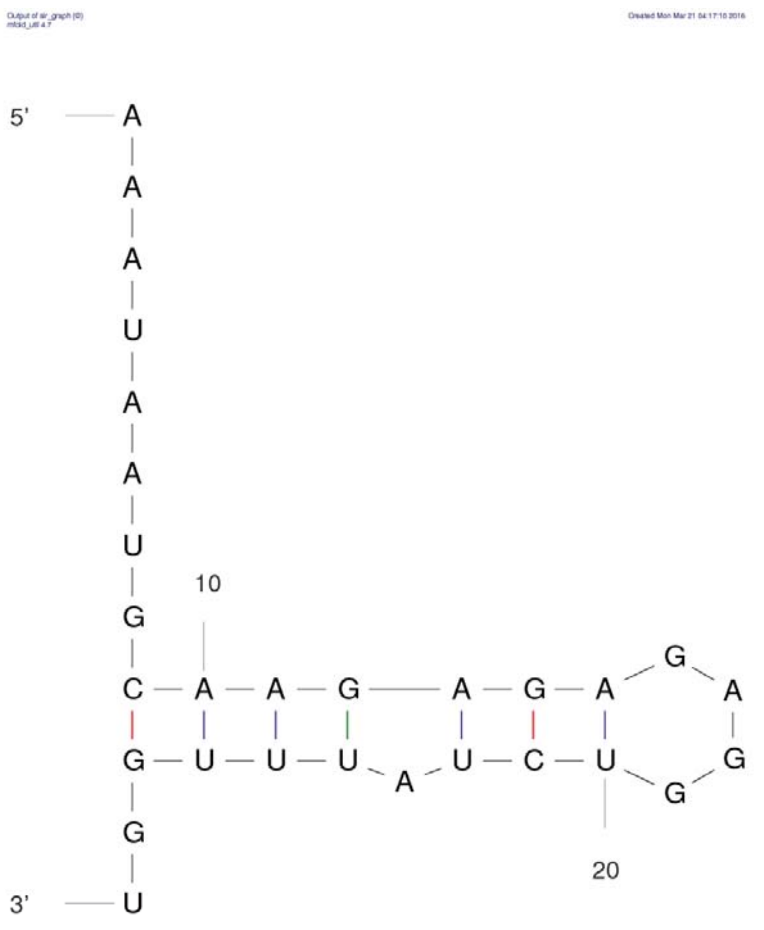

dG = -1.6 A_duck_Jiangxi_26141_2009 H10N7

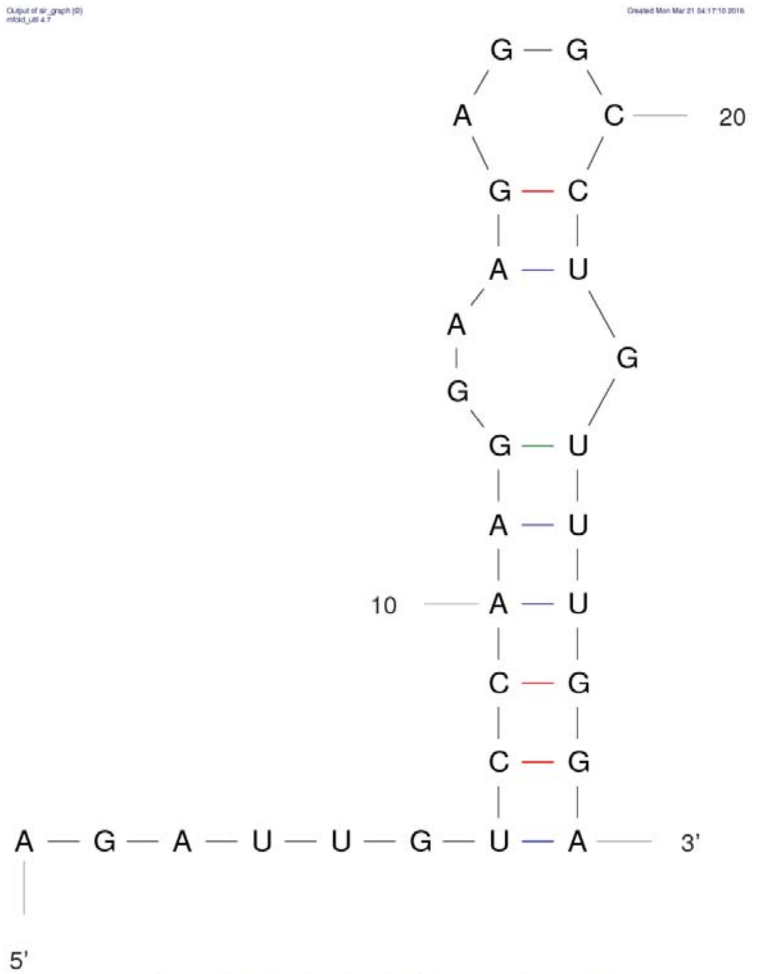

dG = -4.7 A_American black duck_New Brunswick_00471_2010 H10N6

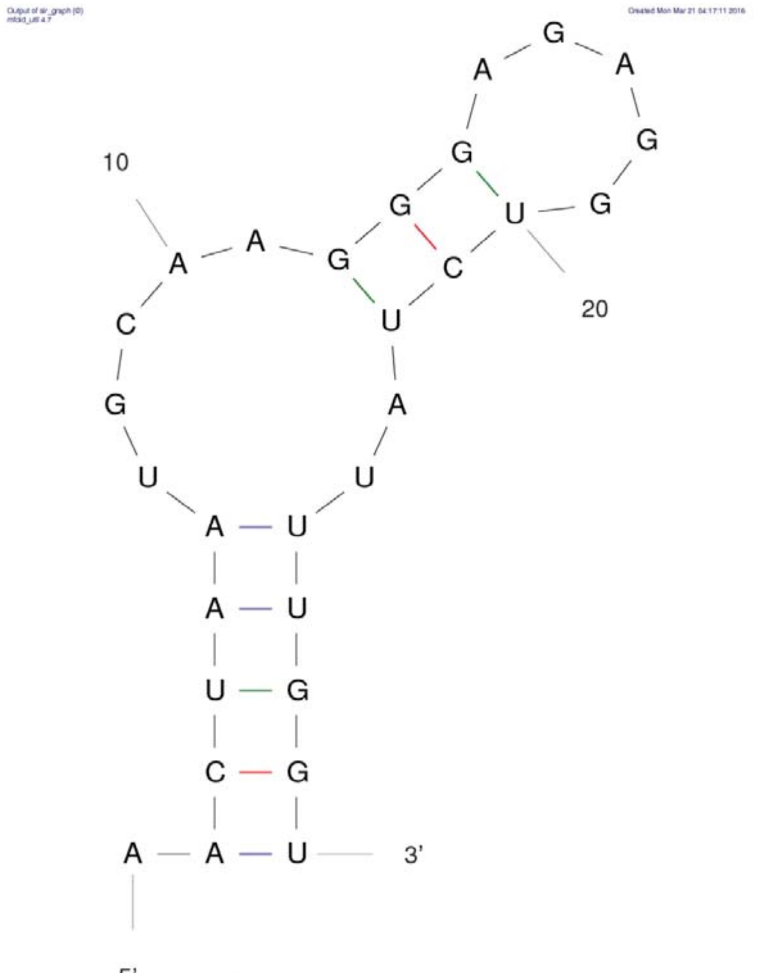

dG = -2.1 A_domestic duck_Republic of Georgia_1_2010 H10N7

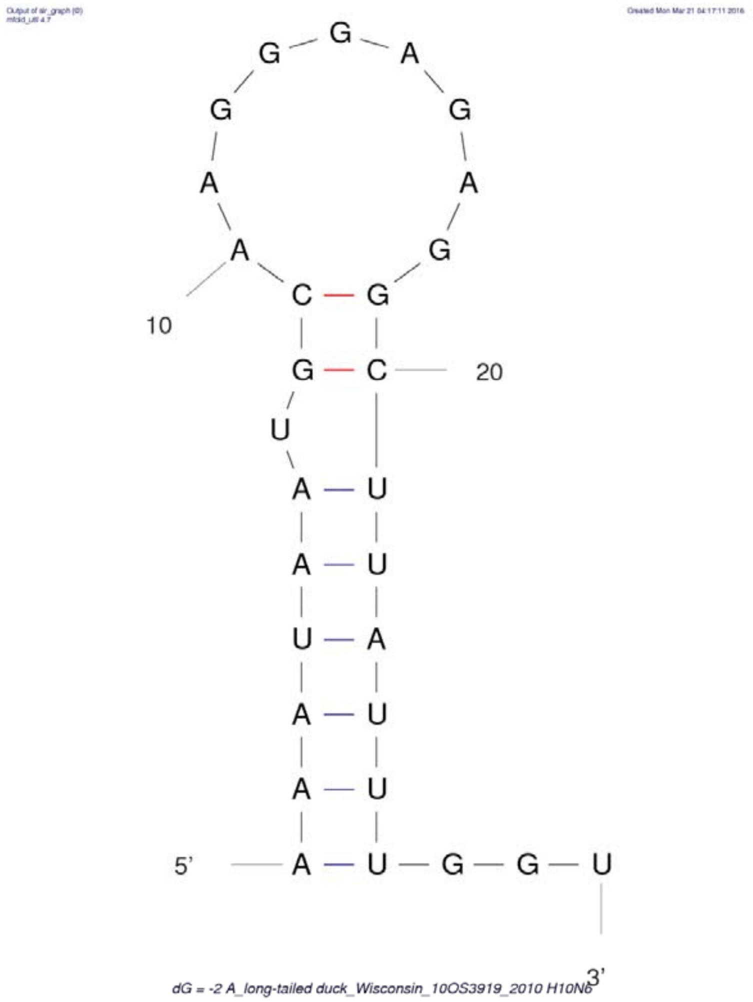

dG = -2 A_long-tailed duck_Wisconsin_10OS3919_2010 H10N6

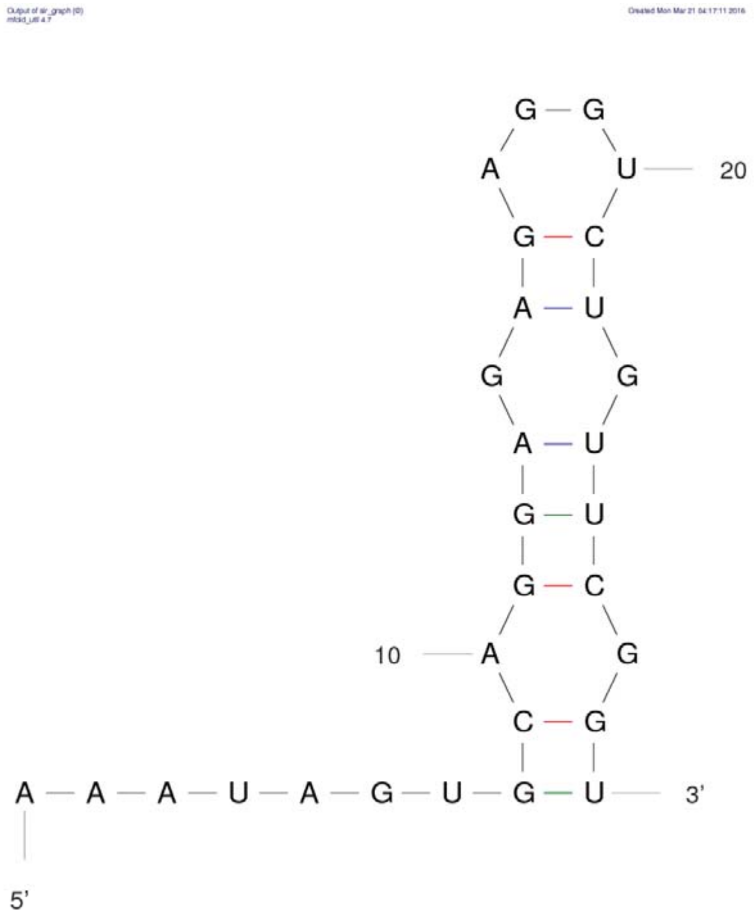

dG = -3.6 A_duck_Guangdong_E1_2012 H10N8

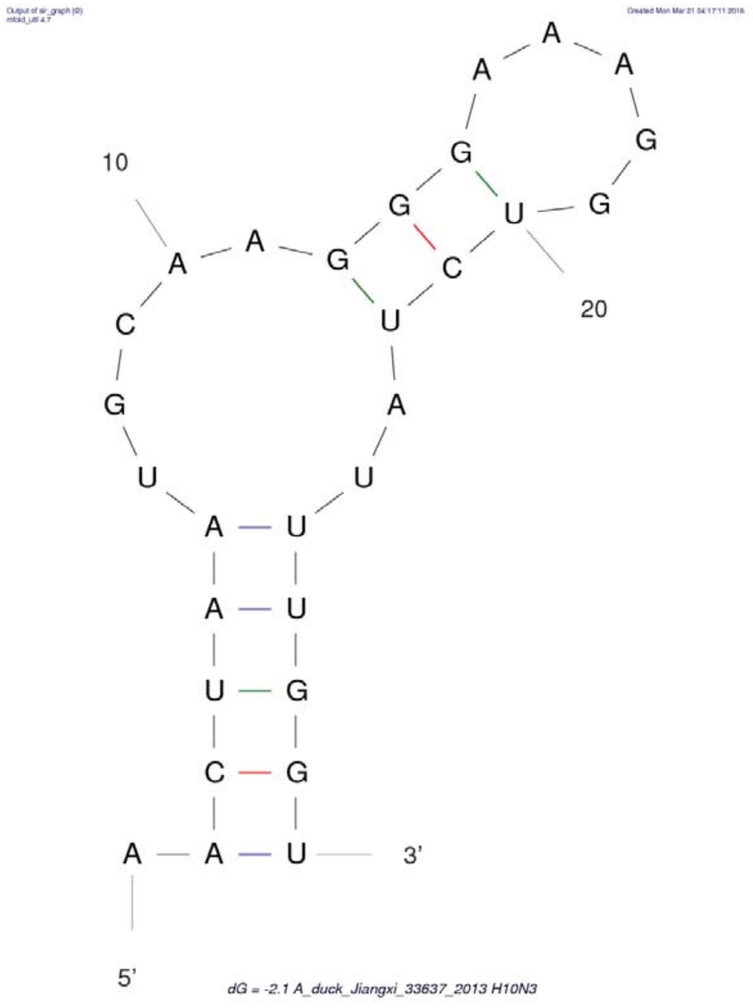

dG = -2.1 A_duck_Jiangxi_33637_2013 H10N3

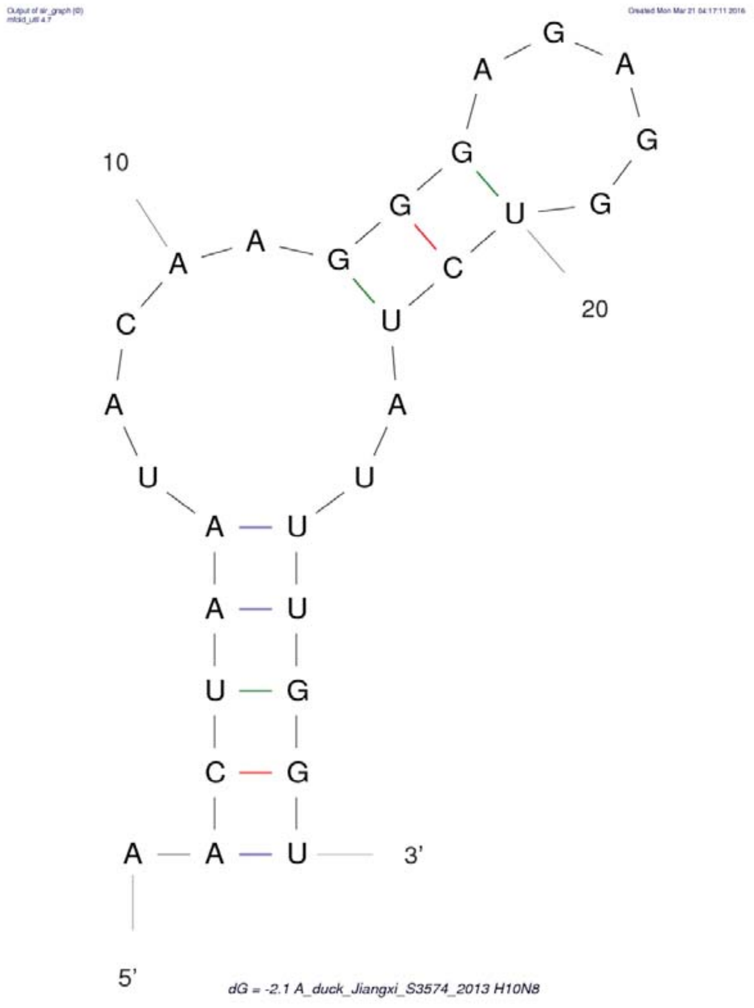

dG = -2.1 A_duck_Jiangxi_S3574_2013 H10N8

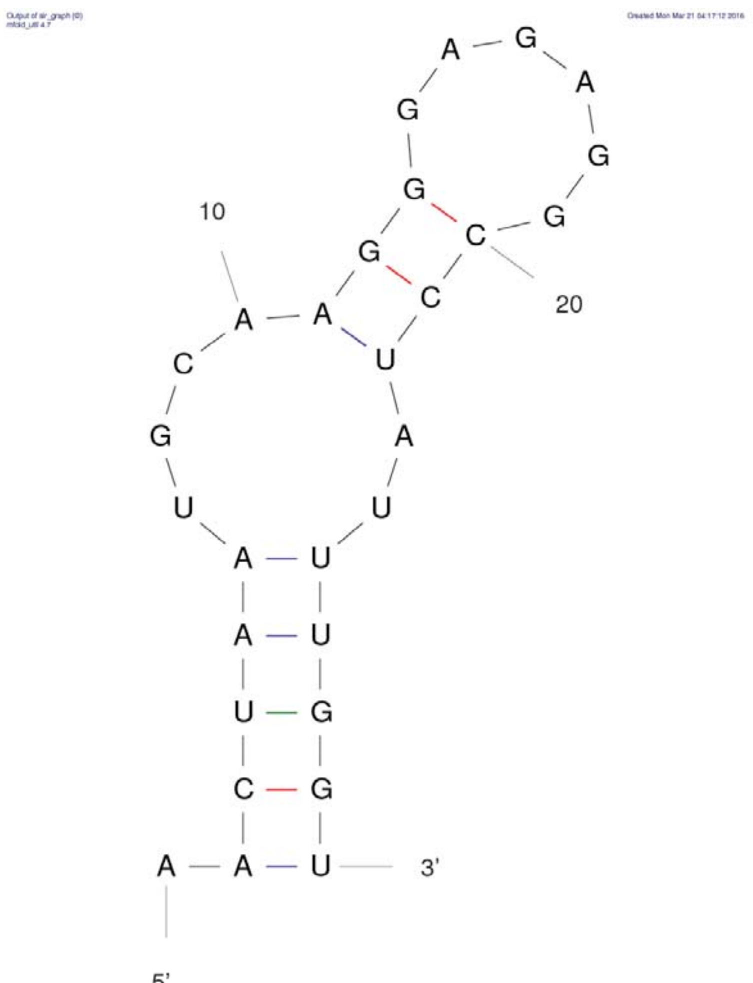

dG = -3.7 A_migratory duck_Jiangxi_33038_2013 H10N7

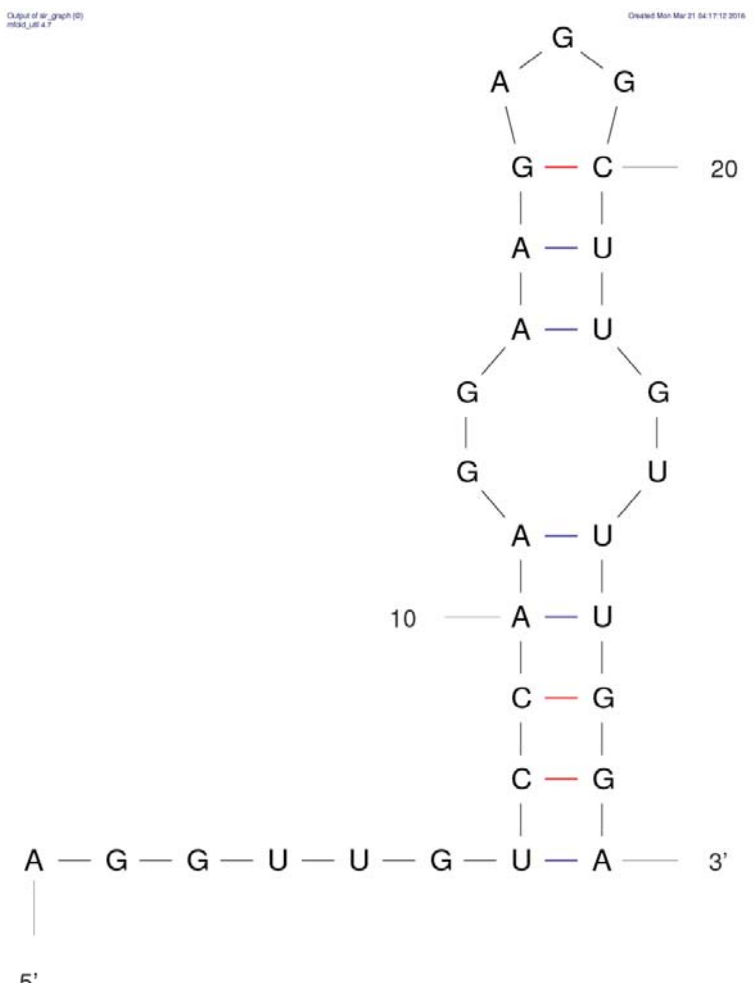

dG = -4 A_harlequin duck_Alaska_250_2014 H10N7

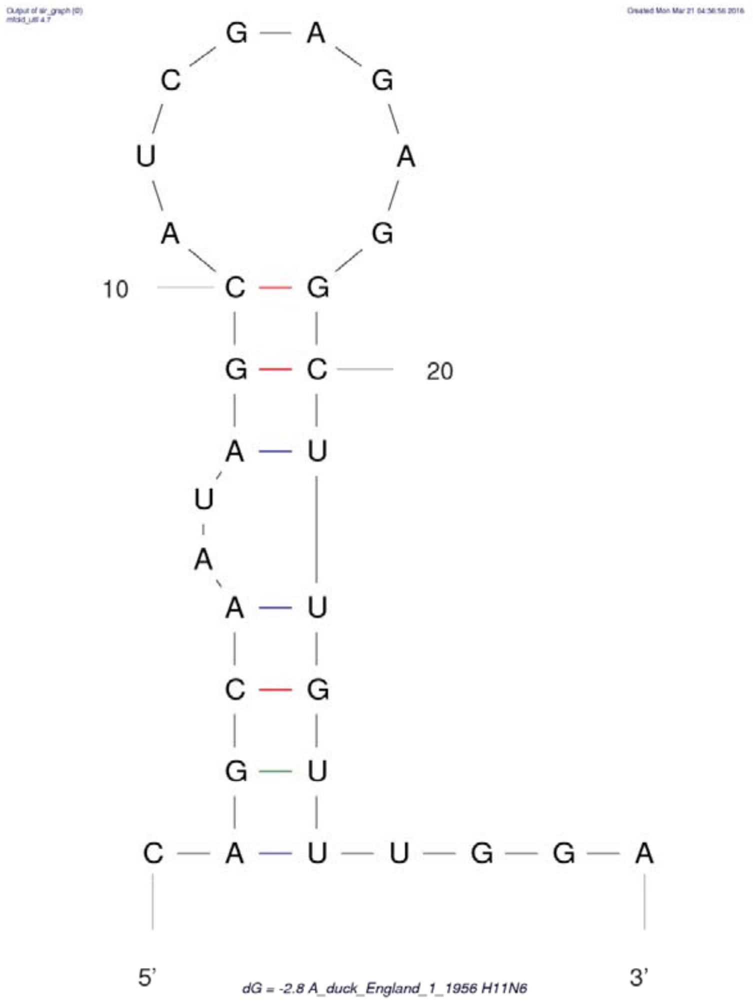

dG = -2.8 A_duck_England_1_1956 H11N6

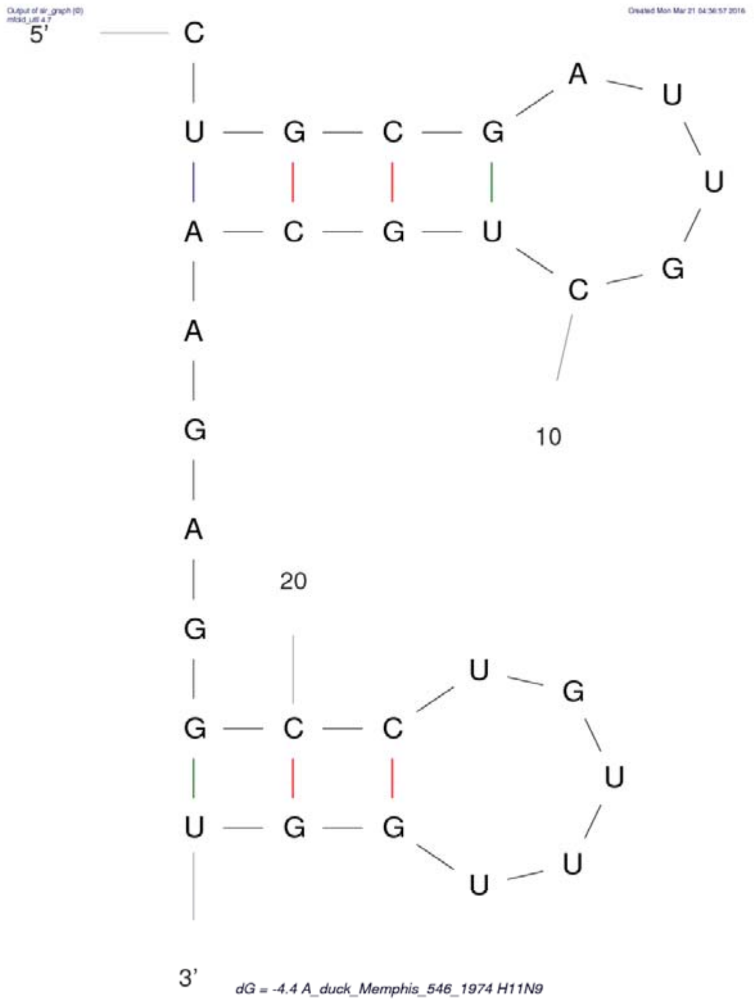

dG = -4.4 A_duck_Memphis_546_1974 H11N9

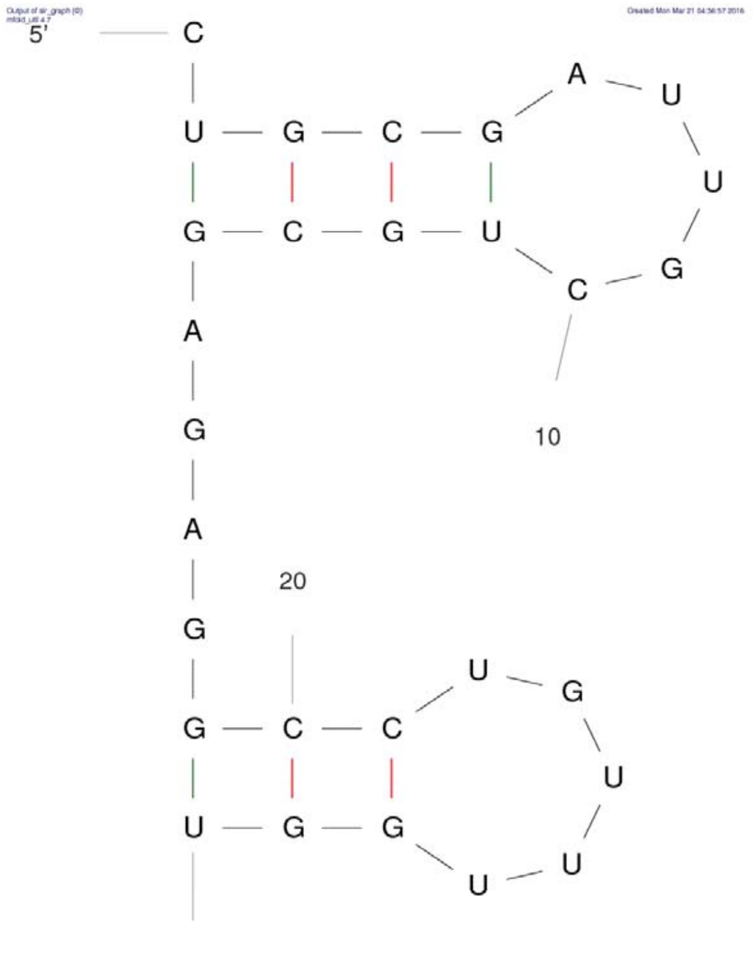

dG = -3.7 A_duck_Minnesota_83_1974 H11N9

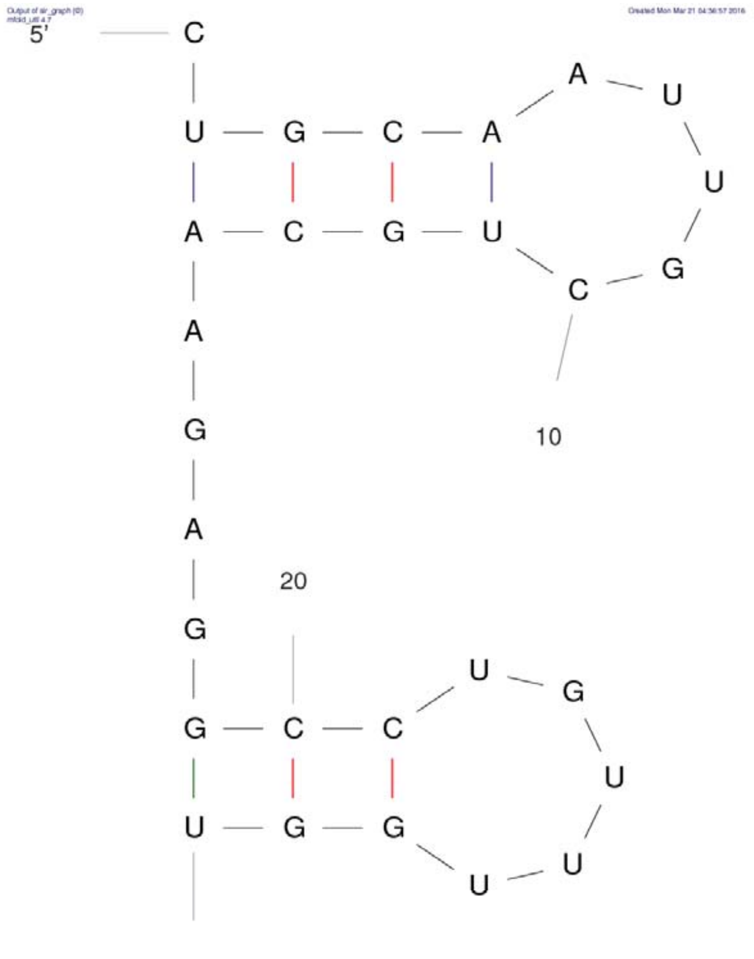

dG = -5.1 A_redhead duck_Alberta_357_1983 H11N9

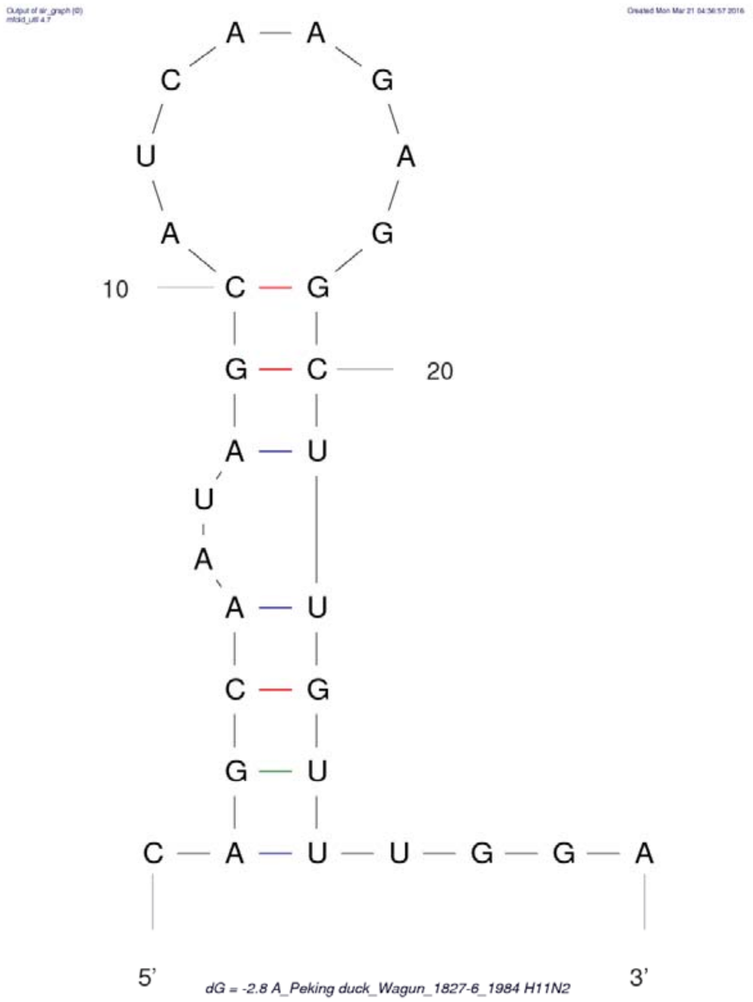

dG = -2.8 A_Peking duck_Wagun_1827-6_1984 H11N2

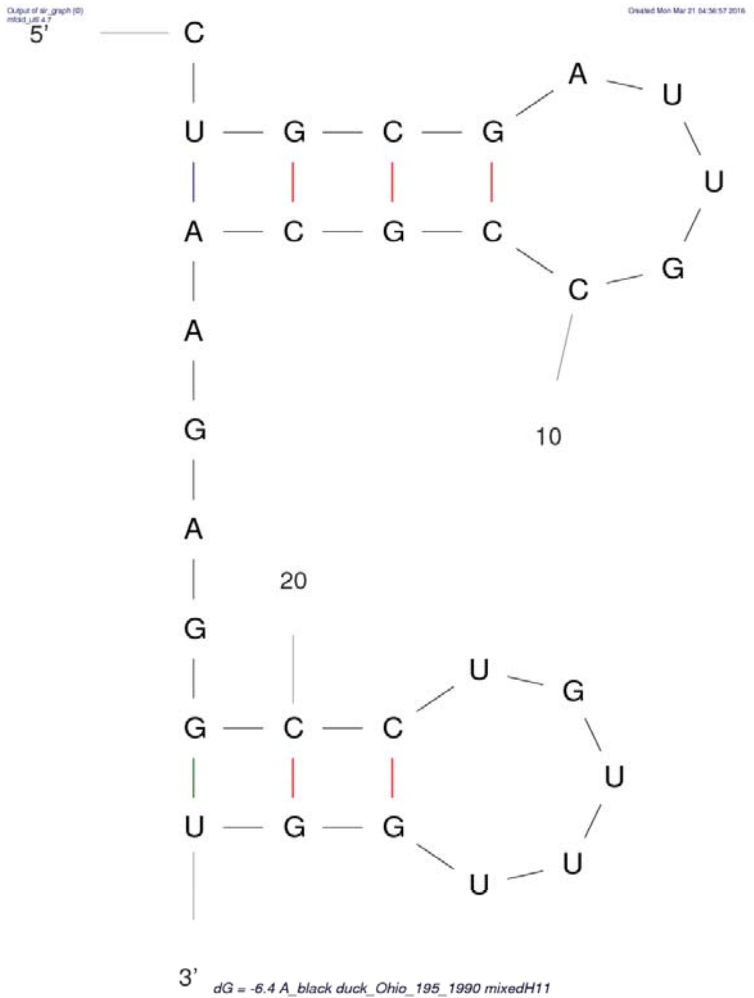

dG = -6.4 A_black duck_Ohio_195_1990 mixedH11

Output of sir_graph (©)
mfold_util 4.7

Created Mon Mar 21 04:36:57 2016

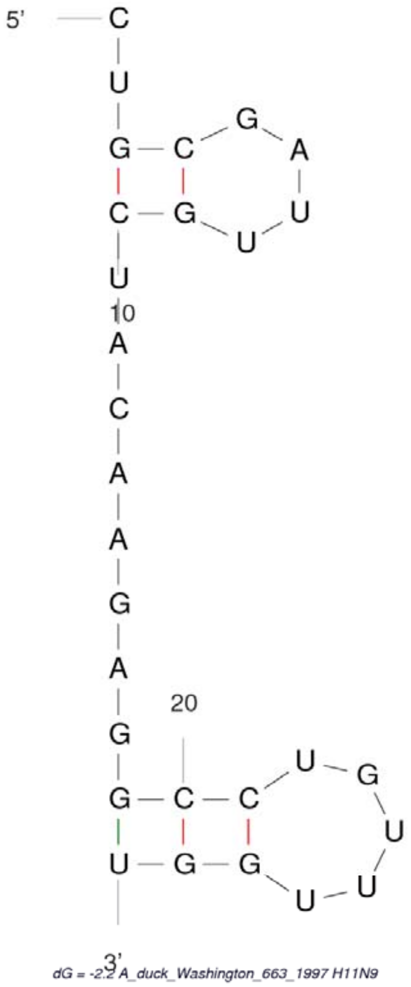

dG = -2.2 A_duck_Washington_663_1997 H11N9

Output of sir_graph (©)
mfold_util 4.7

Created Mon Mar 21 04:36:58 2016

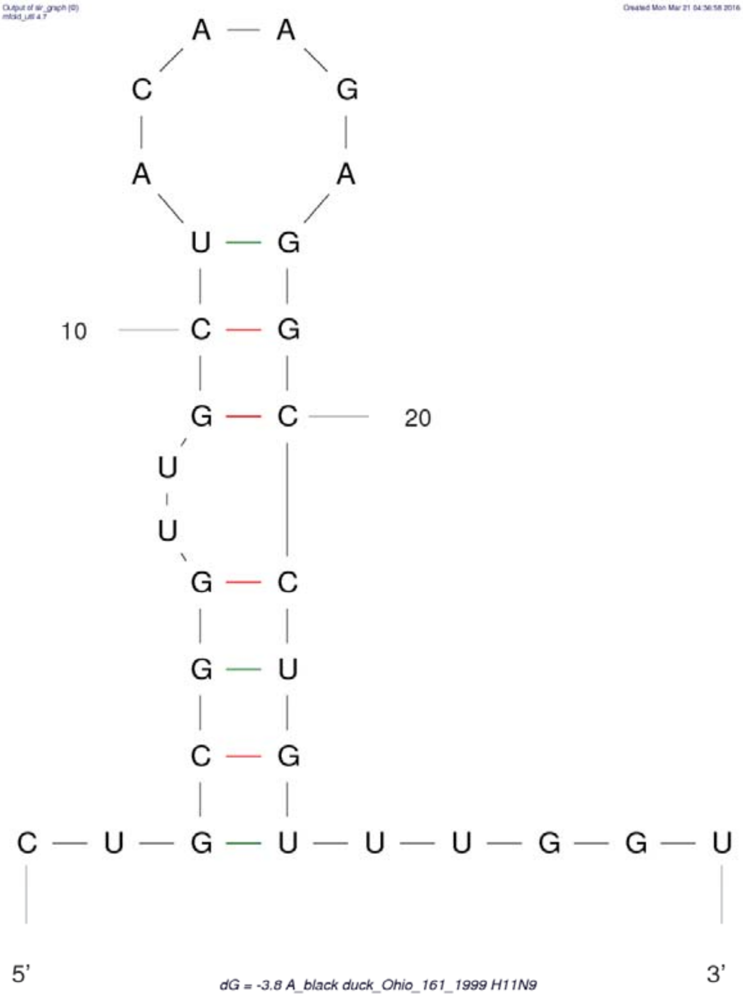

dG = -3.8 A_black duck_Ohio_161_1999 H11N9

Output of sir_graph (©)
mfold_util 4.7

Created Mon Mar 21 04:36:58 2016

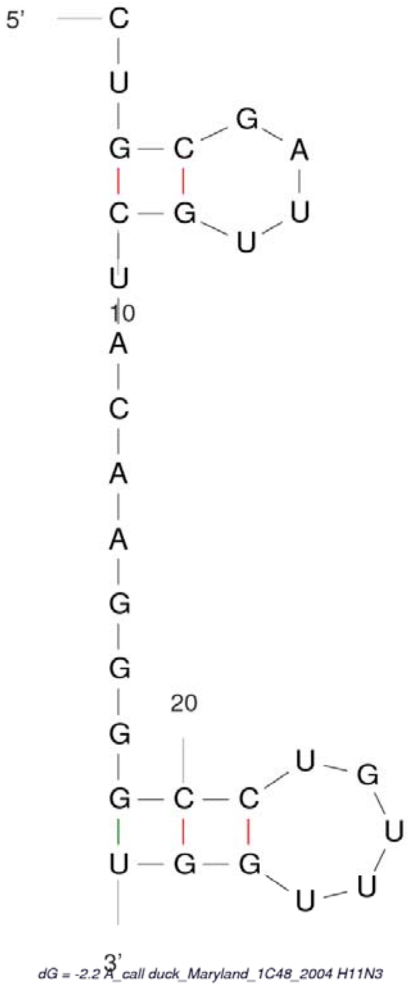

dG = -2.2 A_call duck_Maryland_1C48_2004 H11N3

Output of sir_graph (©)
mfold_util 4.7

Created Mon Mar 21 04:36:58 2016

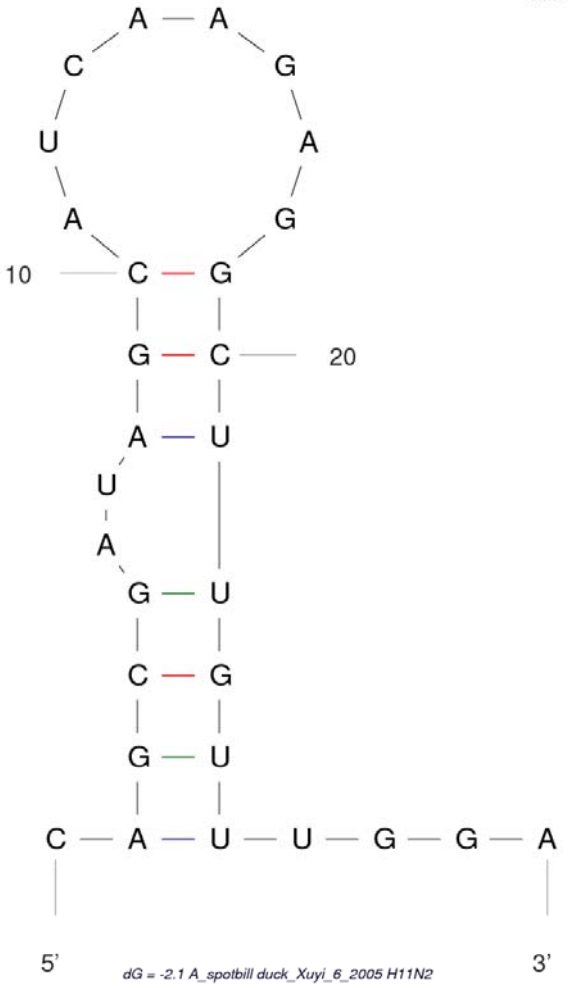

dG = -2.1 A_spotbill duck_Xuyi_6_2005 H11N2

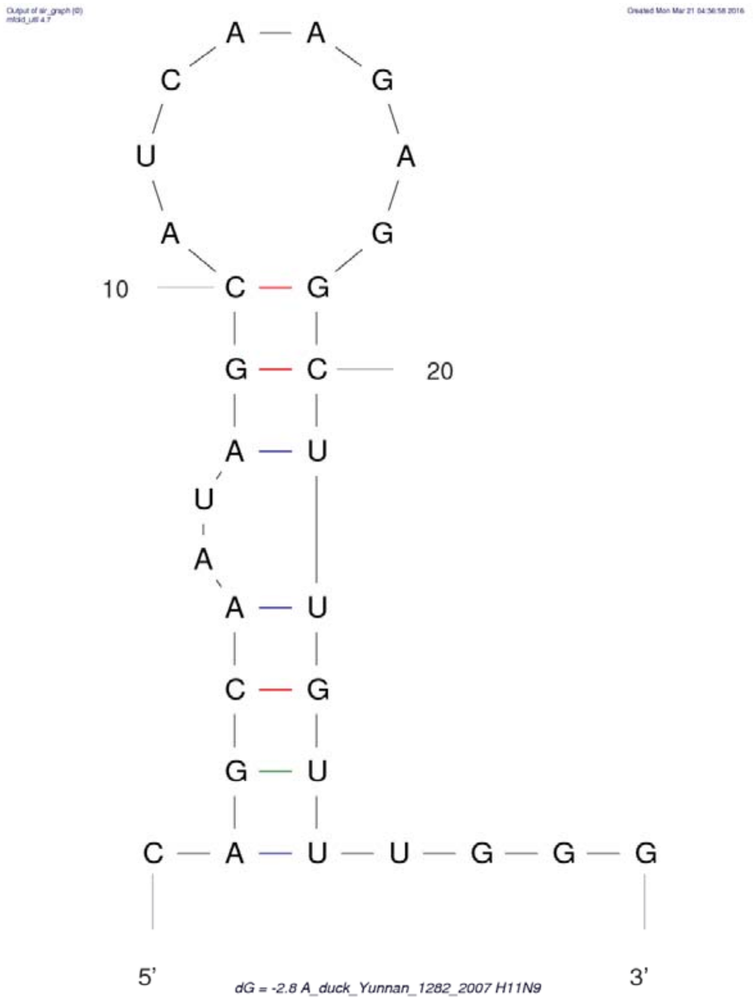

dG = -2.8 A_duck_Yunnan_1282_2007 H11N9

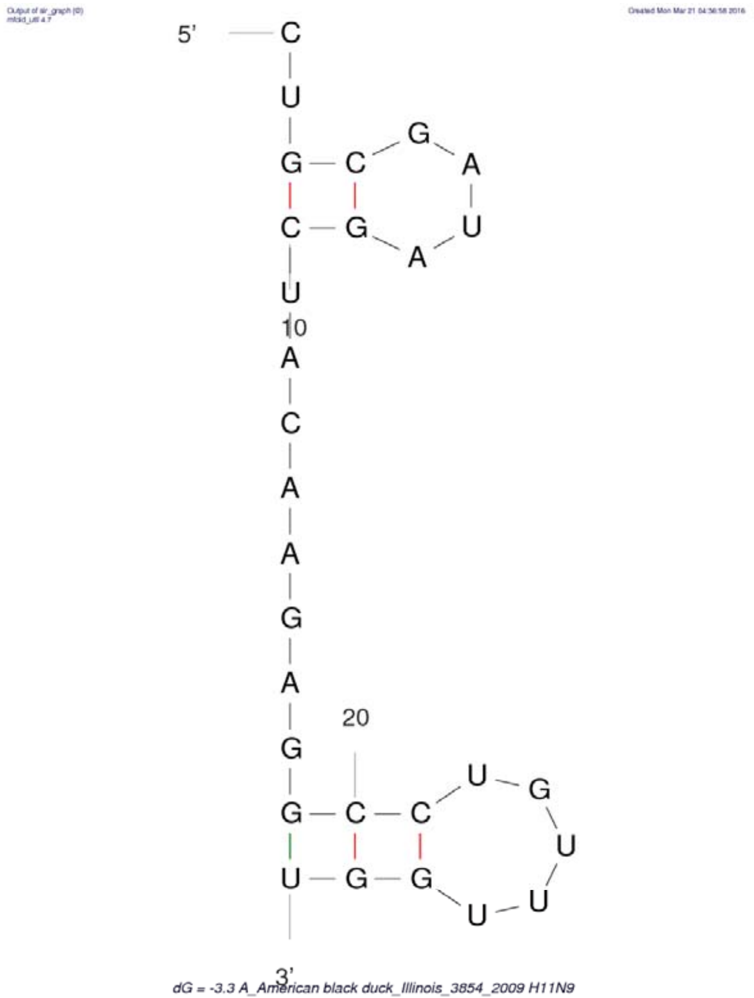

dG = -3.3 A_American black duck_Illinois_3854_2009 H11N9

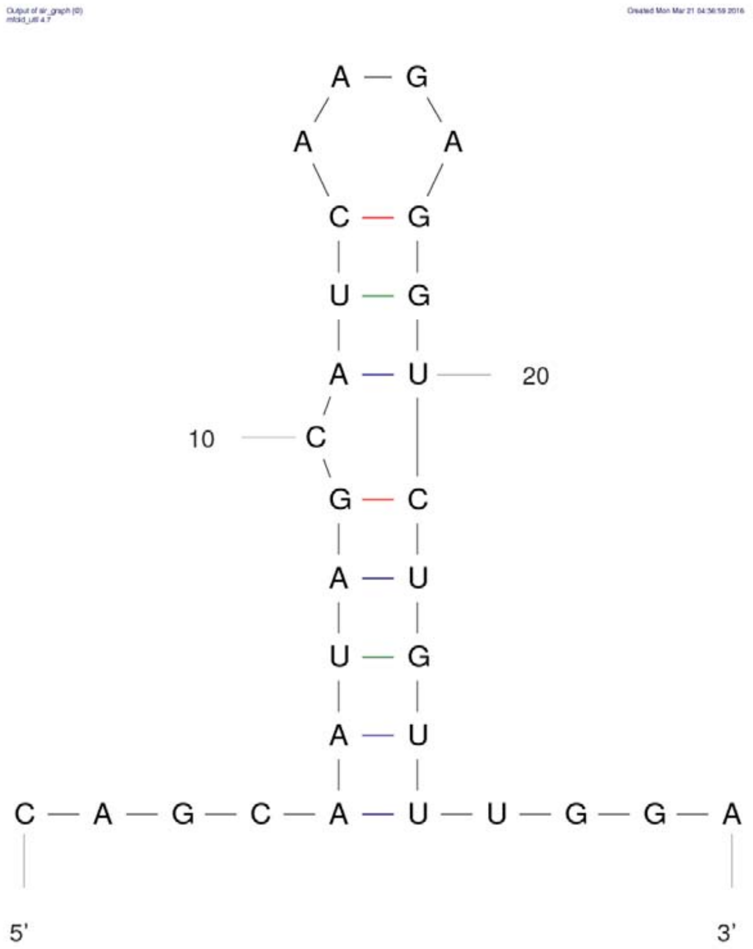

dG = -2.9 A_duck_Thailand_CU5388_2009 H11N3

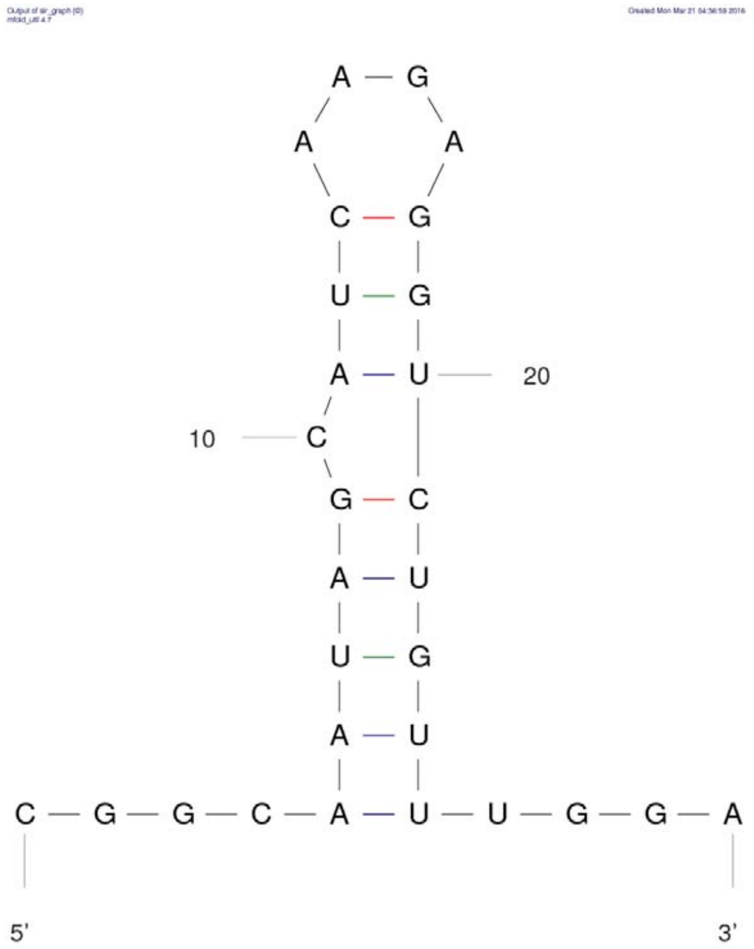

dG = -2.9 A_duck_Vietnam_OIE-2329_2009 H11N3

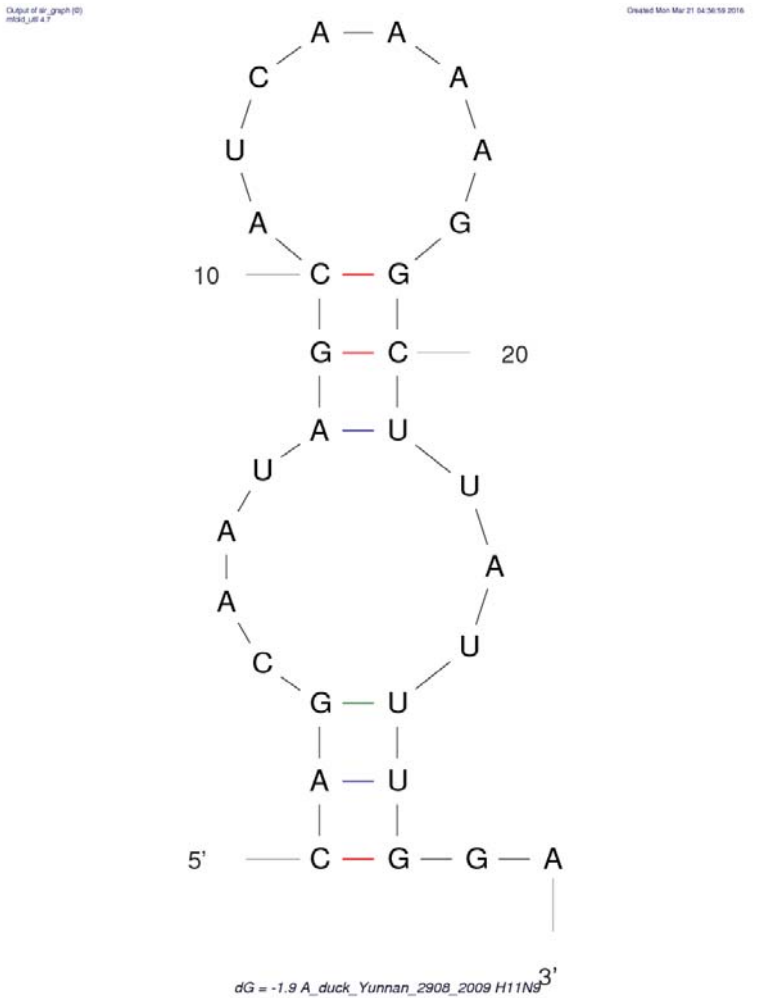

dG = -1.9 A_duck_Yunnan_2908_2009 H11N9

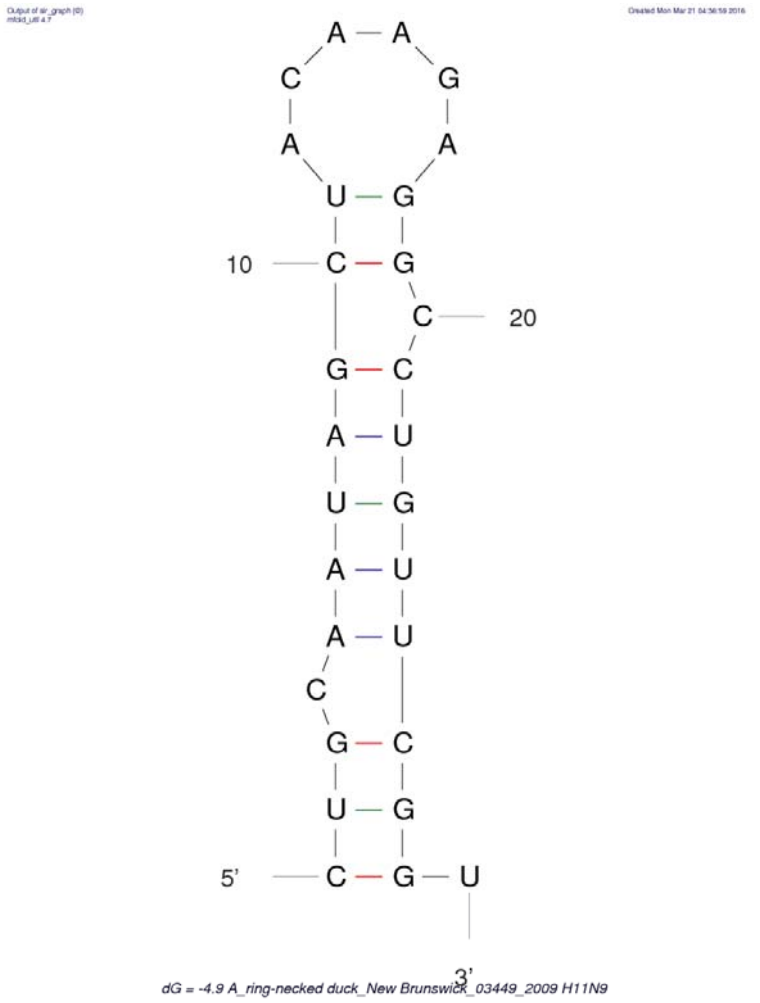

dG = -4.9 A_ring-necked duck_New Brunswick_03449_2009 H11N9

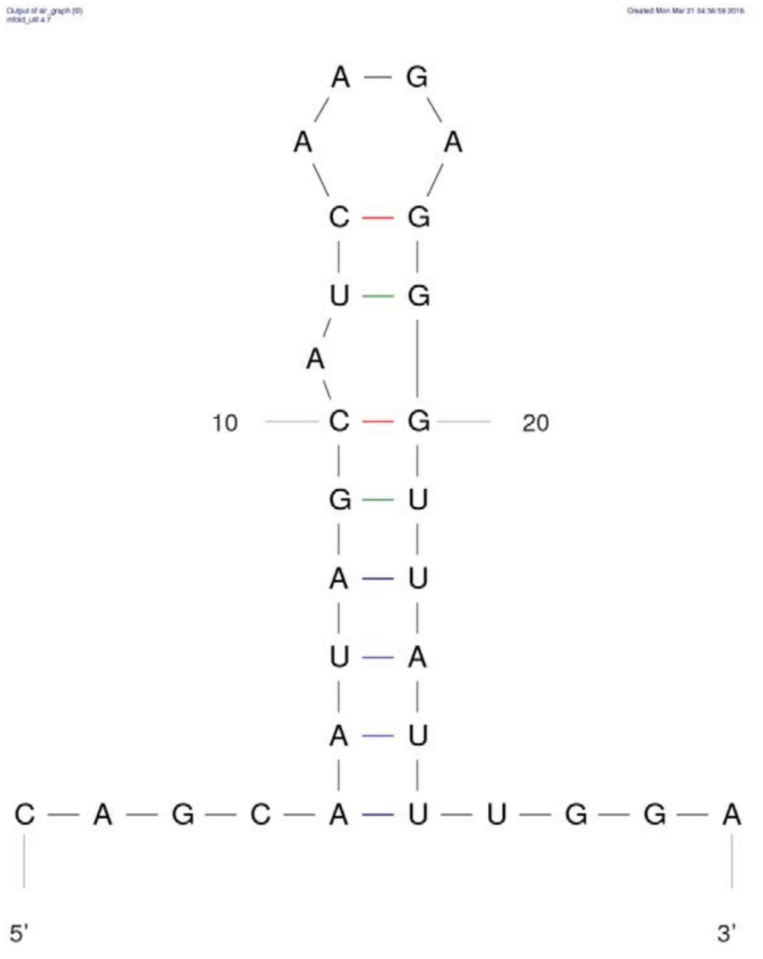

dG = -2.2 A_duck_Guizhou_1078_2011 H11N9

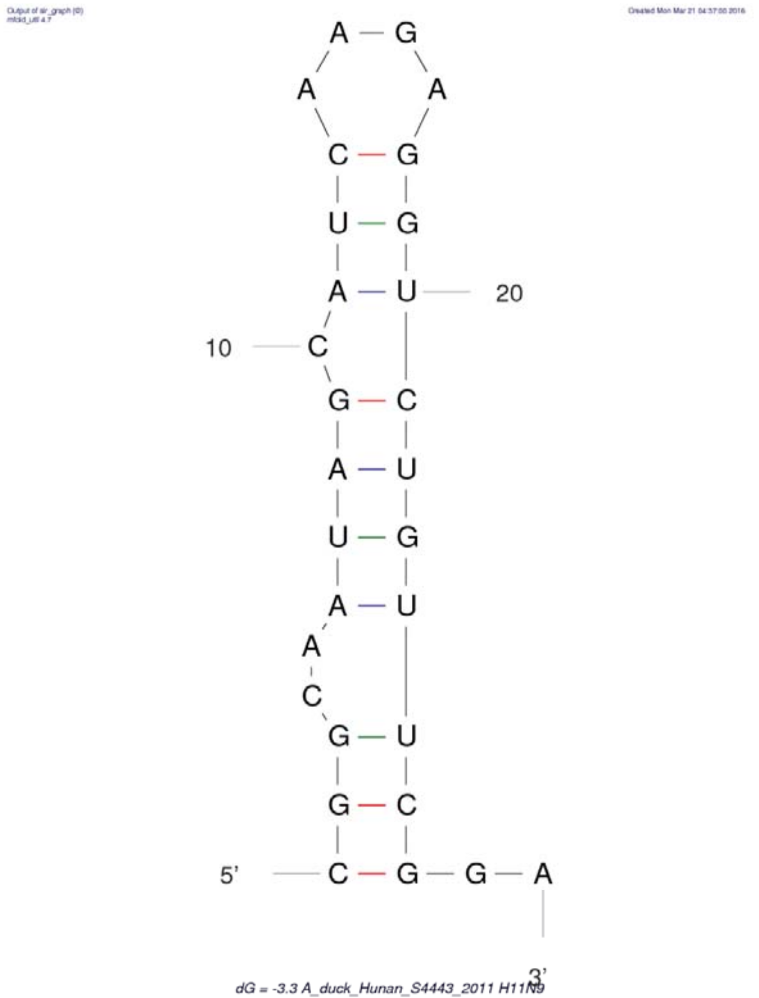

dG = -3.3 A_duck_Hunan_S4443_2011 H11N9

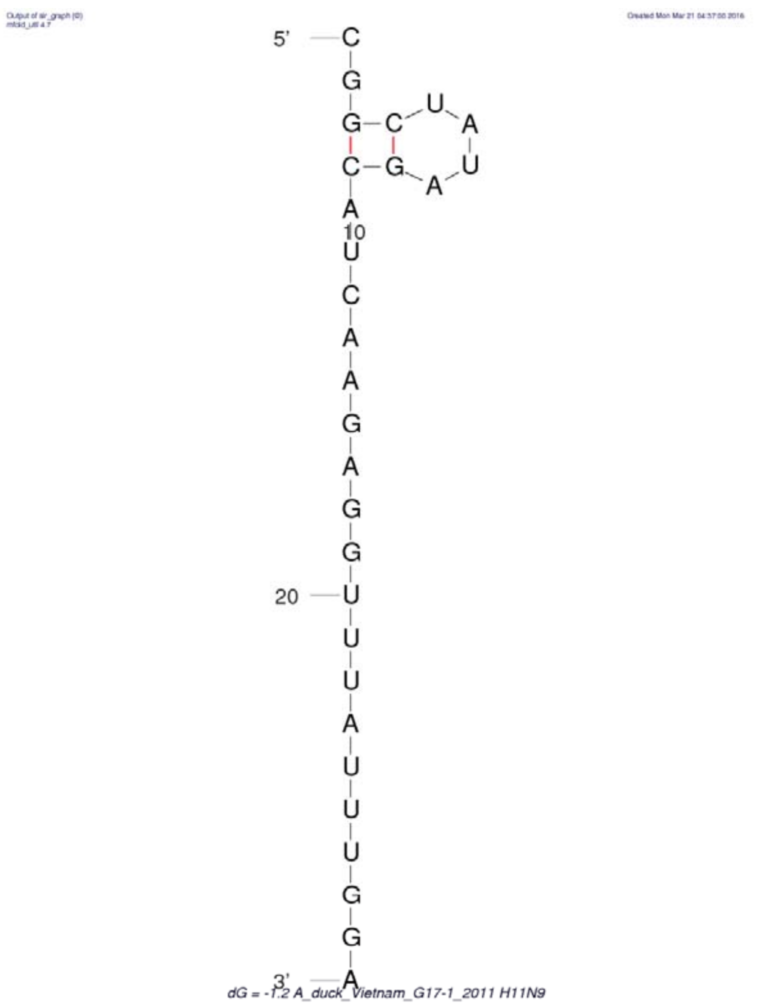

dG = -1.2 A_duck_Vietnam_G17-1_2011 H11N9

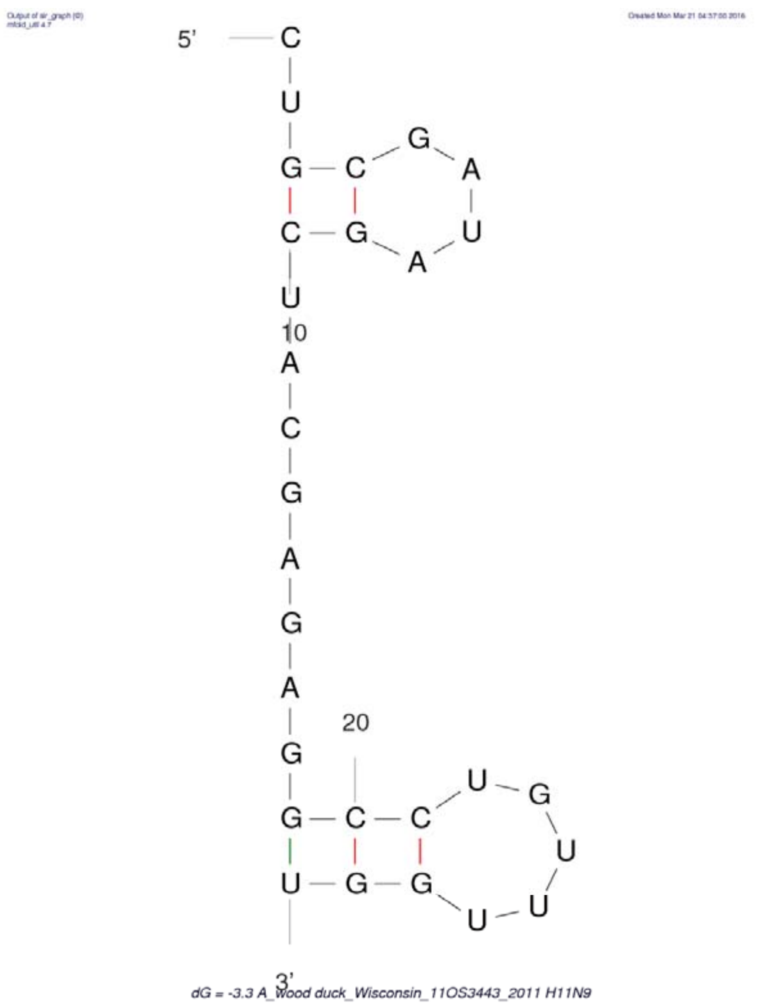

dG = -3.3 A_wood duck_Wisconsin_11OS3443_2011 H11N9

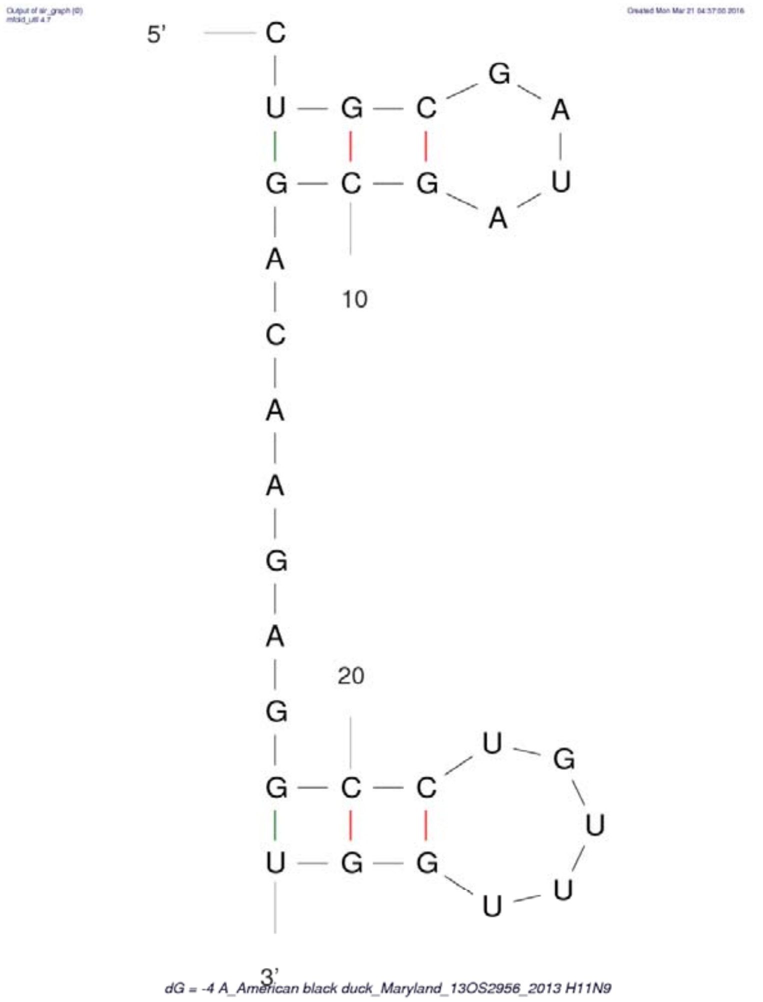

dG = -4 A_American black duck_Maryland_13OS2956_2013 H11N9

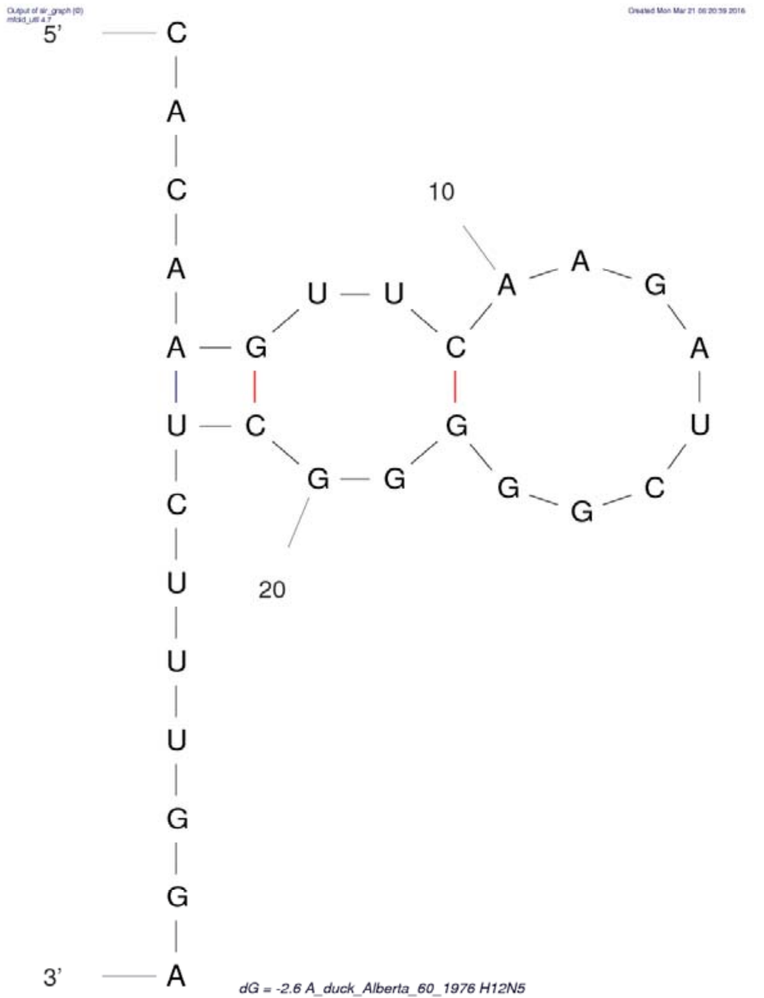

dG = -2.6 A_duck_Alberta_60_1976 H12N5

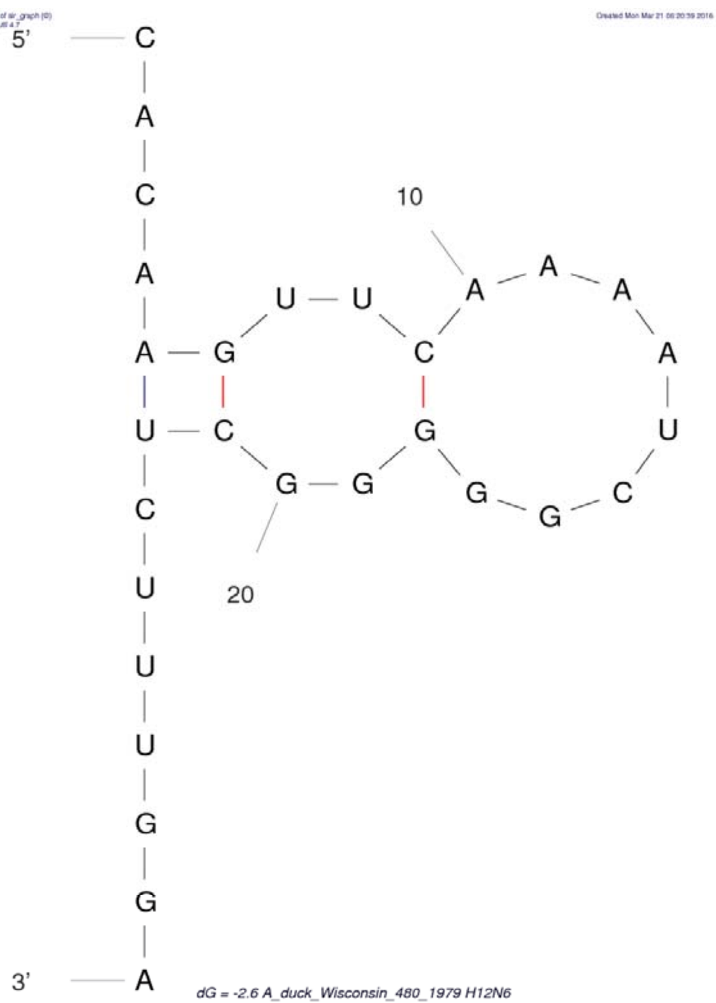

dG = -2.6 A_duck_Wisconsin_480_1979 H12N6

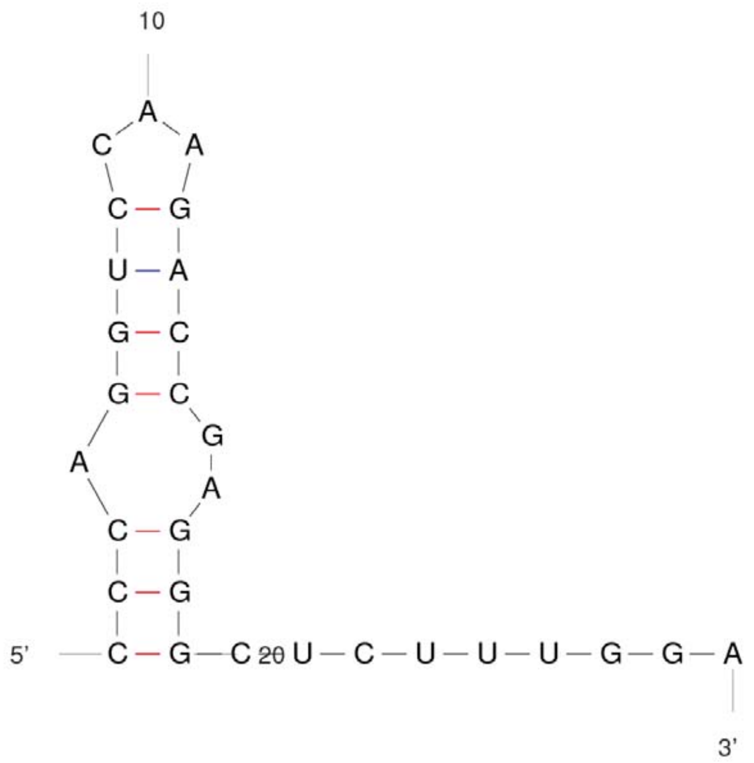

dG = -8.2 A_duck_Hokkaido_66_2001 H12N5

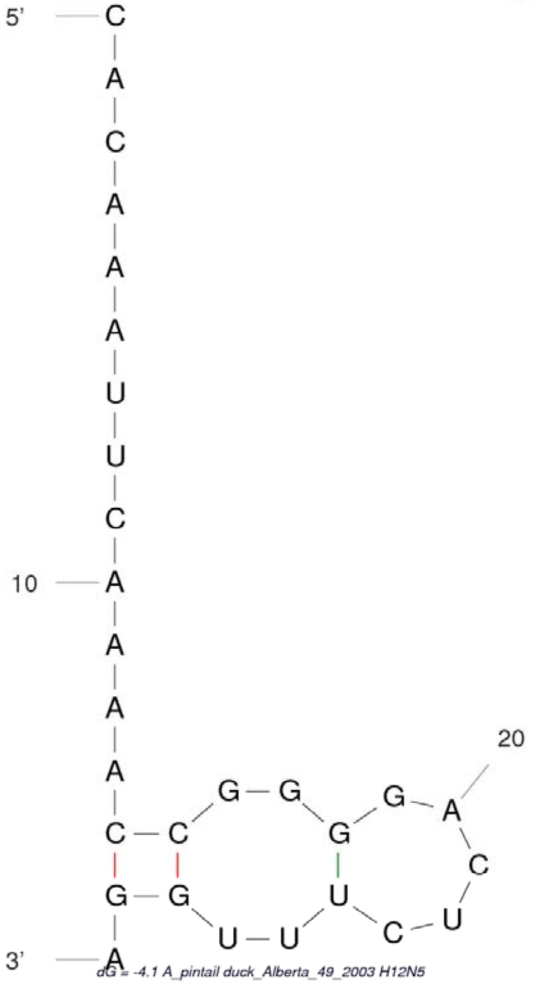

dG = -4.1 A_pintail duck_Alberta_49_2003 H12N5

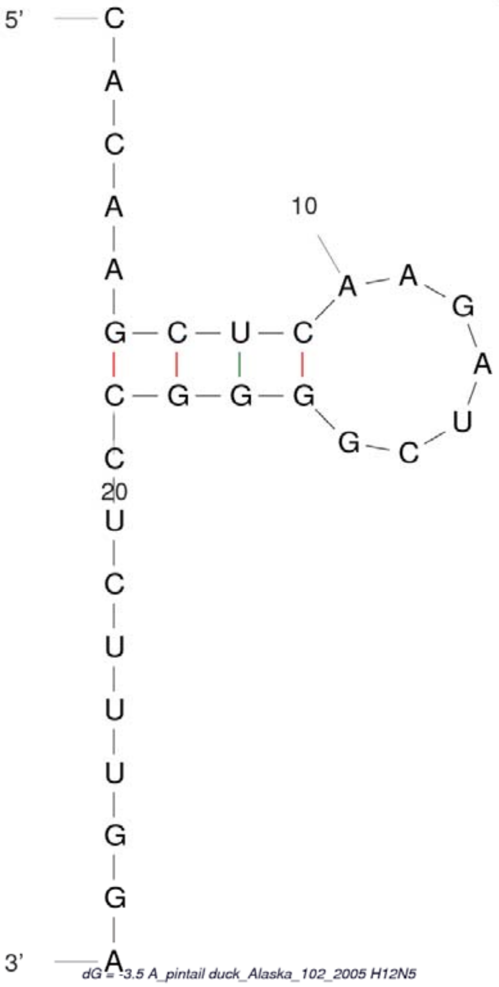

dG = -3.5 A_pintail duck_Alaska_102_2005 H12N5

Output of sir_graph (©)
mfold_util 4.7

Created Mon Mar 21 06:20:40 2016

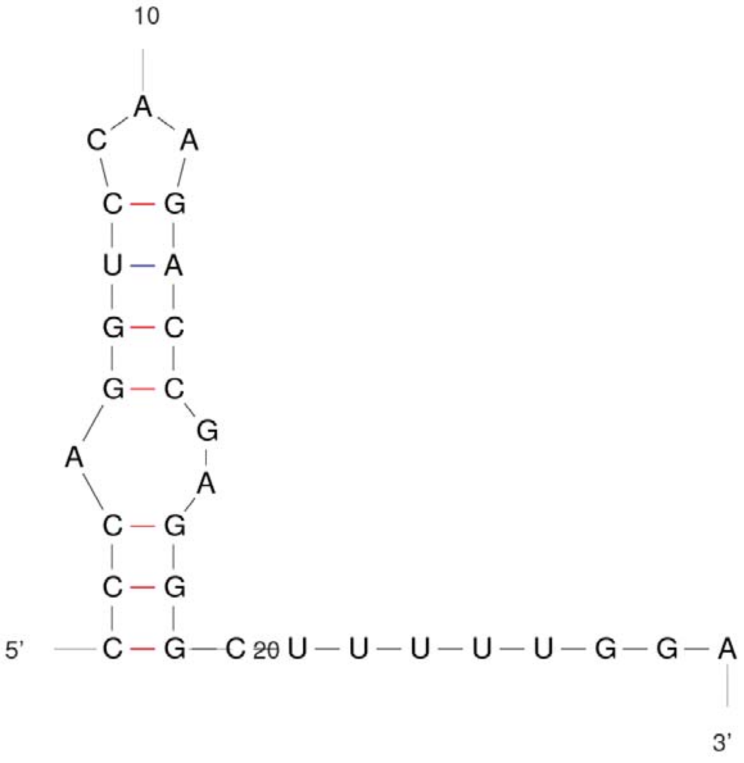

dG = -8.2 A_duck_Tsukuba_212_2006 H12N5

Output of sir_graph (©)
mfold_util 4.7

Created Mon Mar 21 06:20:40 2016

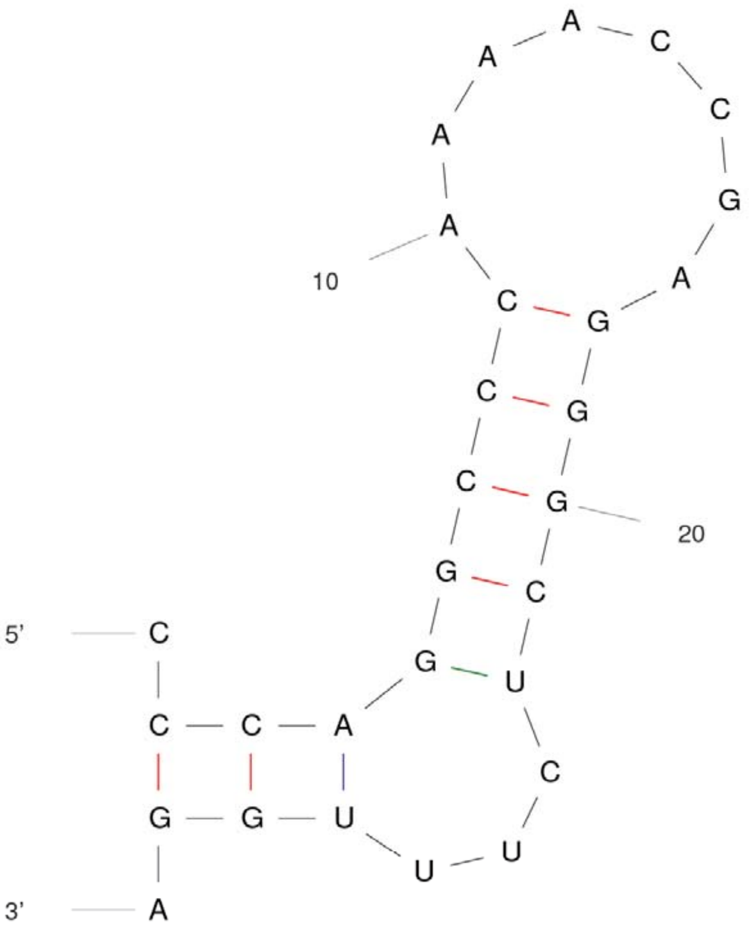

dG = -10.7 A_duck_Vietnam_G18_2009 H12N5

Output of sir_graph (©)
mfold_util 4.7

Created Mon Mar 21 06:20:40 2016

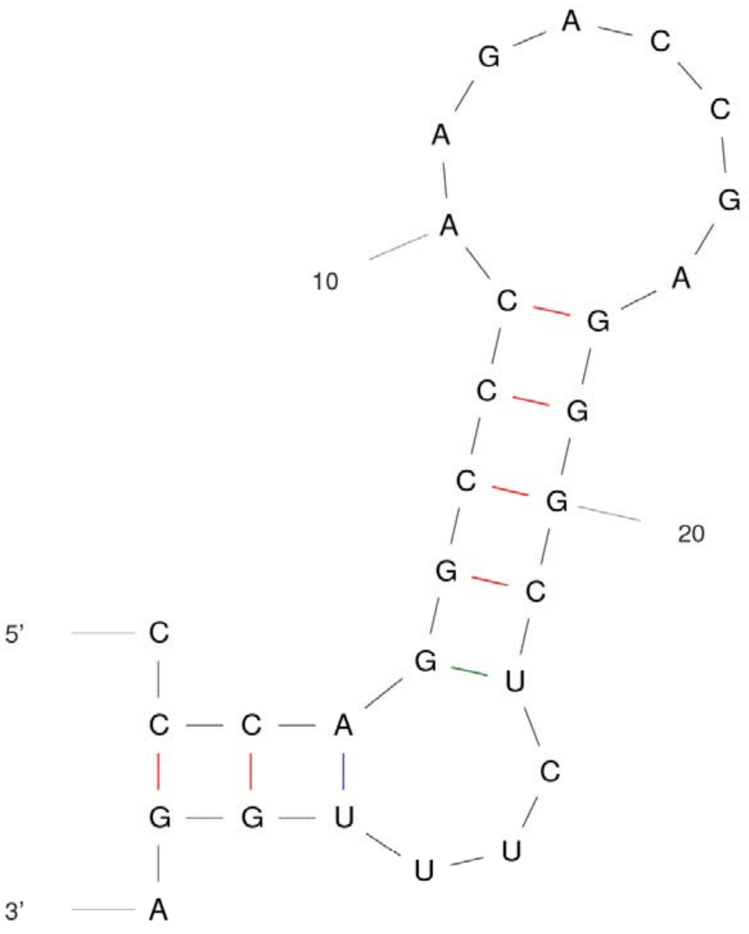

dG = -10.7 A_duck_Hokkaido_W26_2012 H12N1

Output of sir_graph (©)
mfold_util 4.7

Created Mon Mar 21 06:32:59 2016

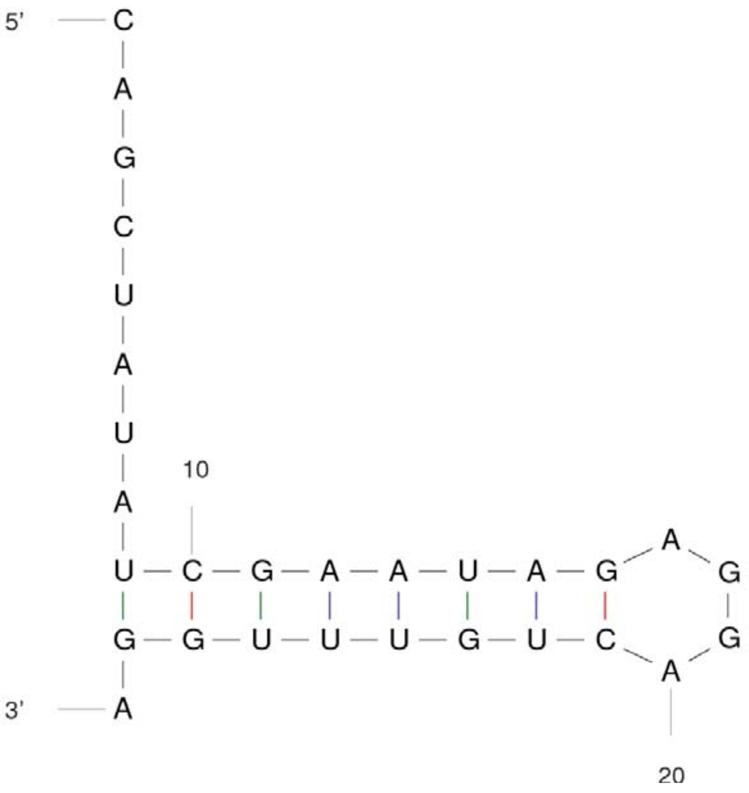

dG = -5.4 A_duck_Siberia_272_1998 H13N6

Output of sir_graph (©)
mfold_util 4.7

Created Mon Mar 21 06:32:59 2016

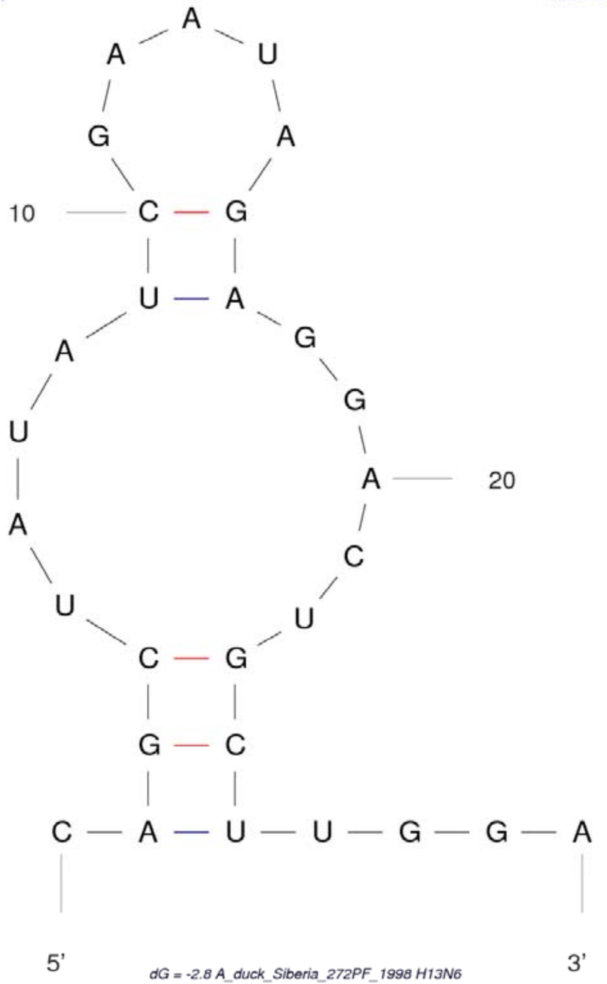

dG = -2.8 A_duck_Siberia_272PF_1998 H13N6

Output of sir_graph (©)
mfold_util 4.7

Created Mon Mar 21 06:32:59 2016

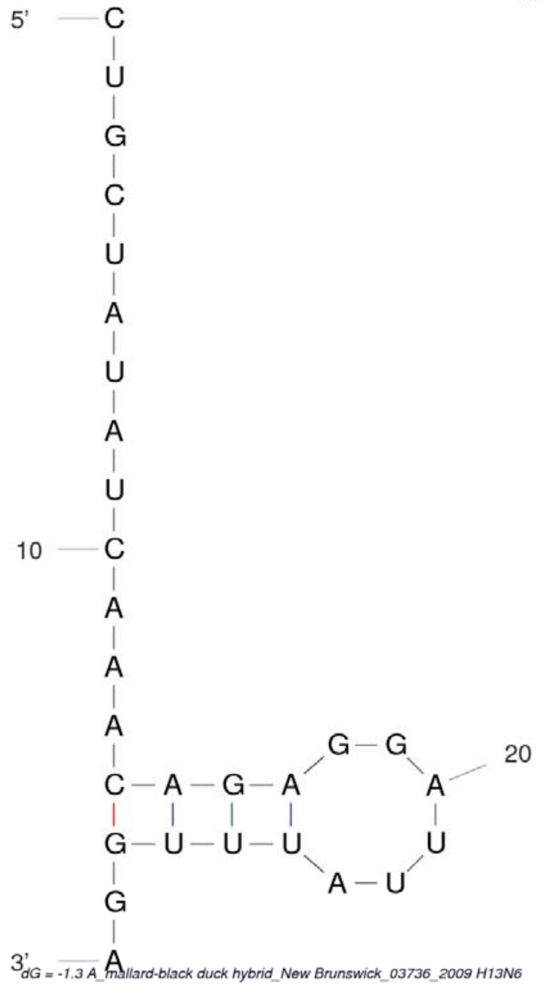

dG = -1.3 A_mallard-black duck hybrid_New Brunswick_03736_2009 H13N6

Output of sir_graph (©)
mfold_util 4.7

Created Mon Mar 21 06:33:00 2016

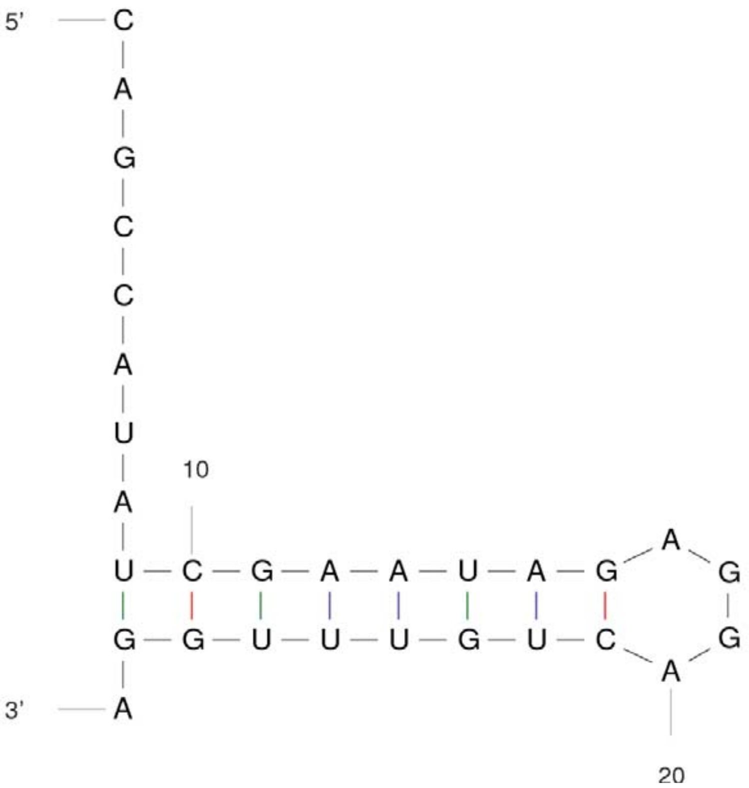

dG = -5.4 A_duck_Interior Alaska_11PG00703_2011 mixedH13

Output of sir_graph (©)
mfold_util 4.7

Created Mon Mar 21 06:33:00 2016

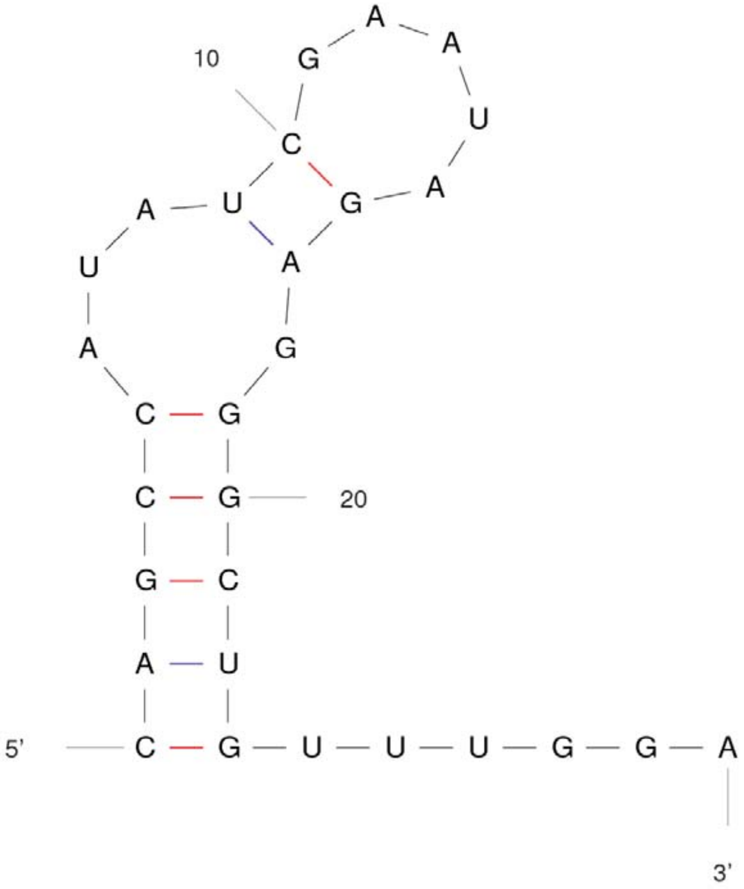

dG = -7.1 A_duck_Hokkaido_WZ68_2012 H13N2

Output of sir_graph (©)
mfold_util 4.7

Created Sat Apr 9 05:47:56 2016

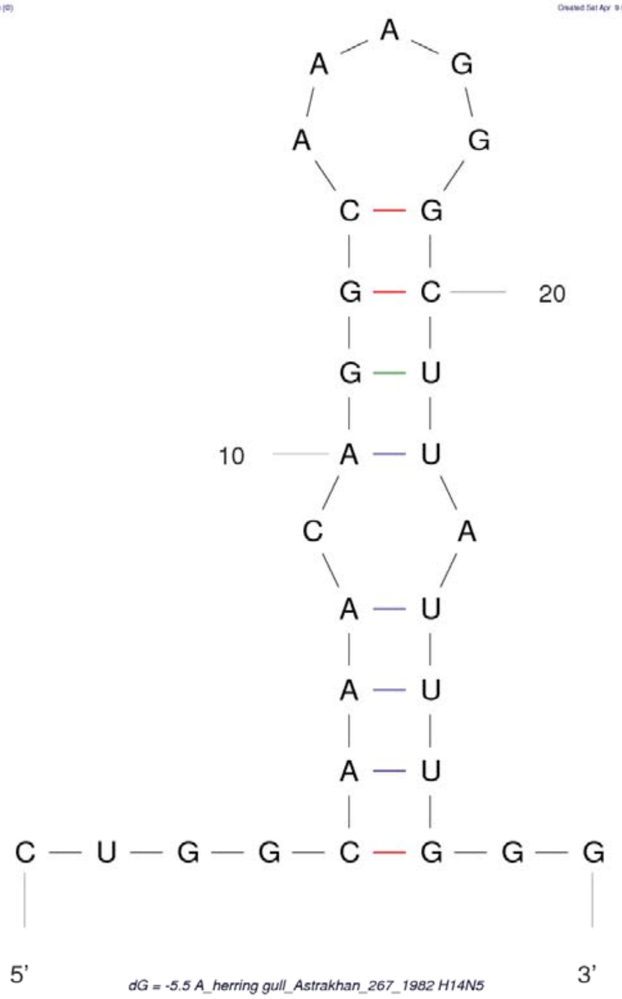

dG = -5.5 A_herring gull_Astrakhan_267_1982 H14N5

Output of sir_graph (©)
mfold_util 4.7

Created Sat Apr 9 05:47:56 2016

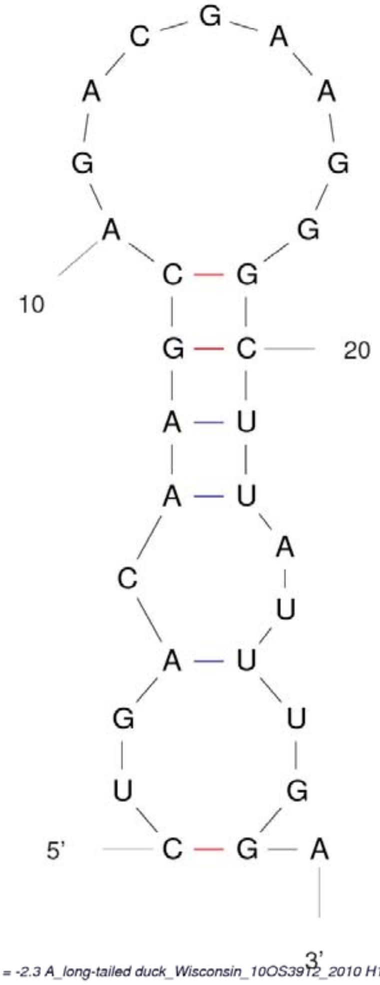

dG = -2.3 A_long-tailed duck_Wisconsin_10OS3912_2010 H14N6

Output of sir_graph (©)
mfold_util 4.7

Created Sat Apr 9 05:47:57 2016

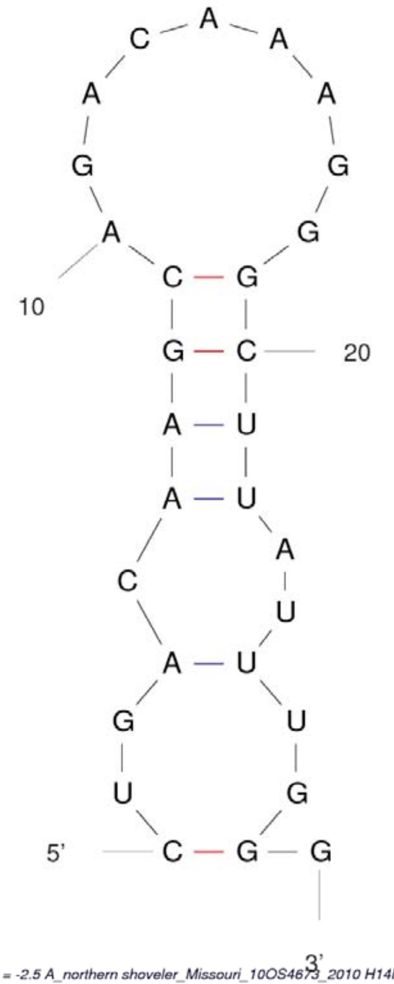

dG = -2.5 A_northern shoveler_Missouri_10OS4673_2010 H14N6

Output of sir_graph (©)
mfold_util 4.7

Created Sat Apr 9 05:47:57 2016

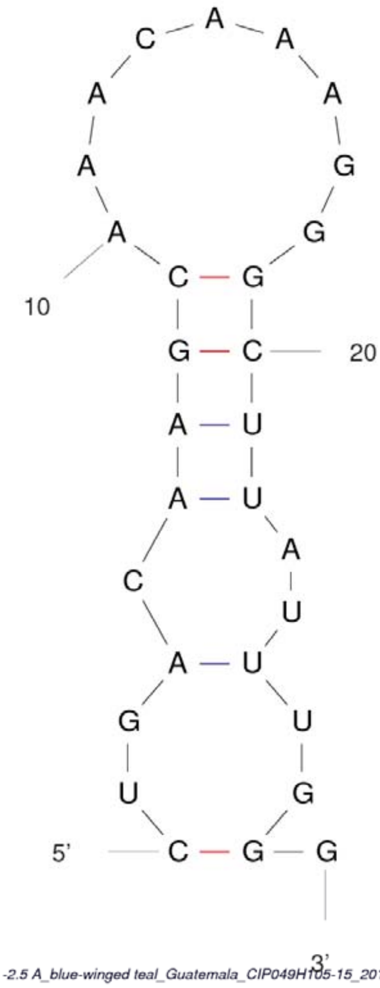

dG = -2.5 A_blue-winged teal_Guatemala_CIP049H105-15_2011 H14N3

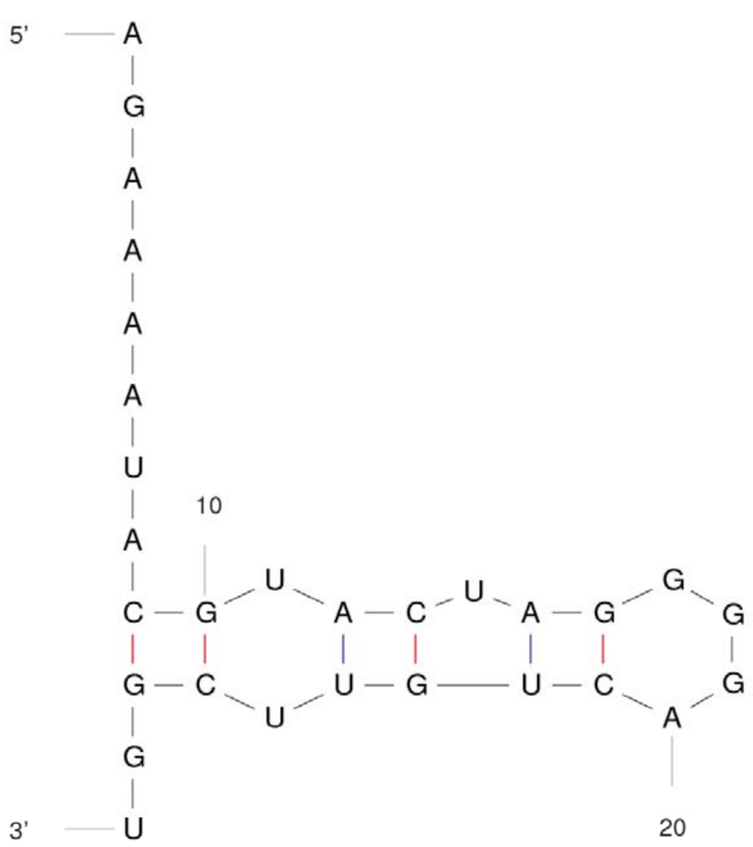

dG = -5.3 A_Australian shelduck_Western Australia_1762_1979 H15N9

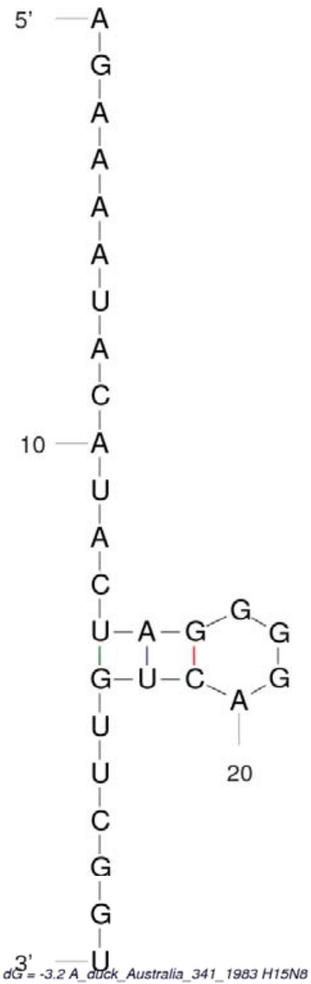

dG = -3.2 A_duck_Australia_341_1983 H15N8

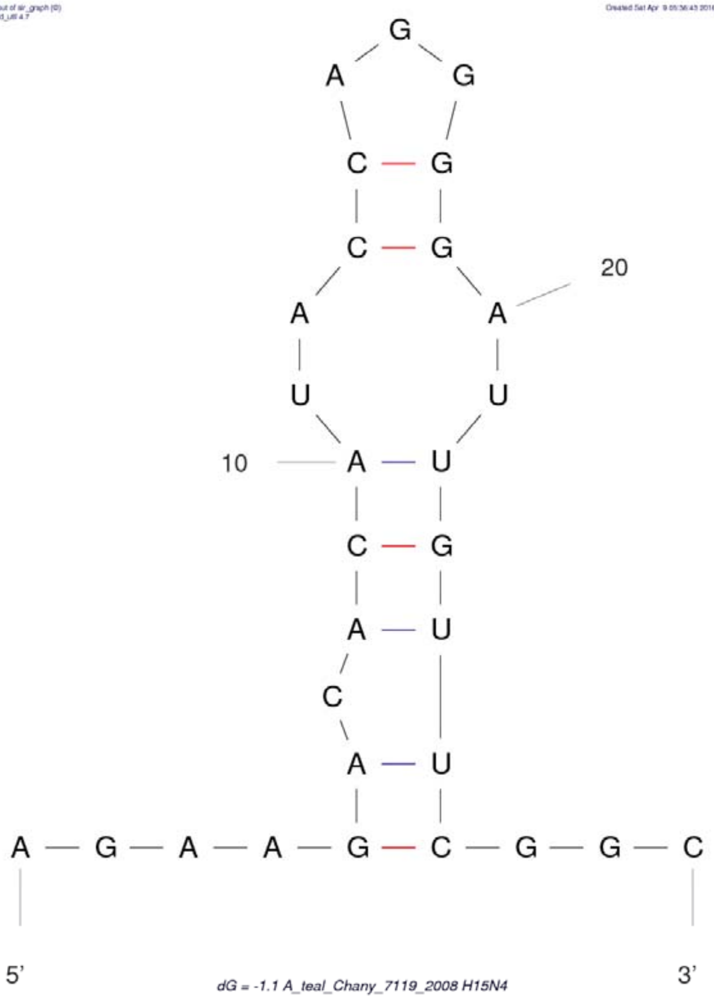

dG = -1.1 A_teal_Chany_7119_2008 H15N4

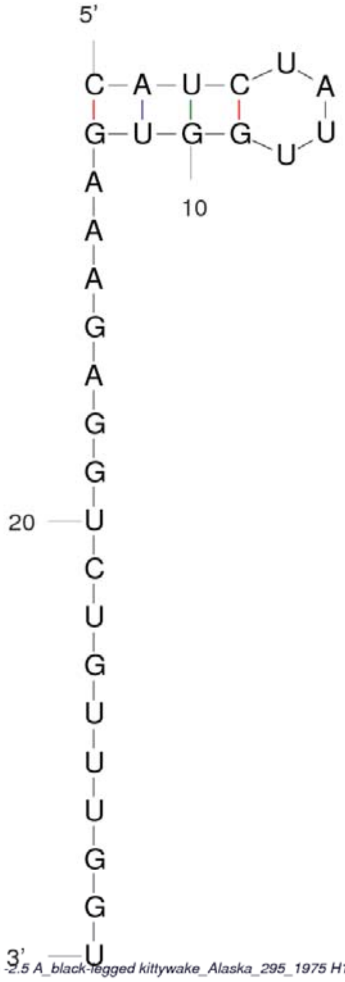

dG = -2.5 A_black-legged kittywake_Alaska_295_1975 H16N3

Output of sir_graph (©)
mfold_util 4.7

Created Sat Apr 9 05:19:07 2016

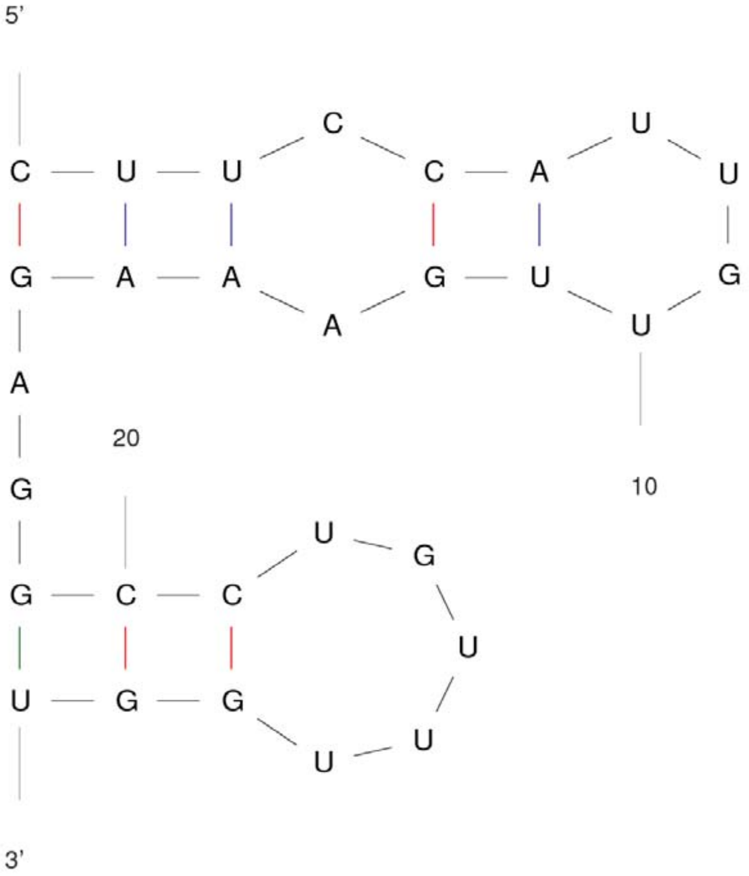

dG = -2.7 A_little tern_Gurjev_779_83 H16N3

Output of sir_graph (©)
mfold_util 4.7

Created Sat Apr 9 05:19:07 2016

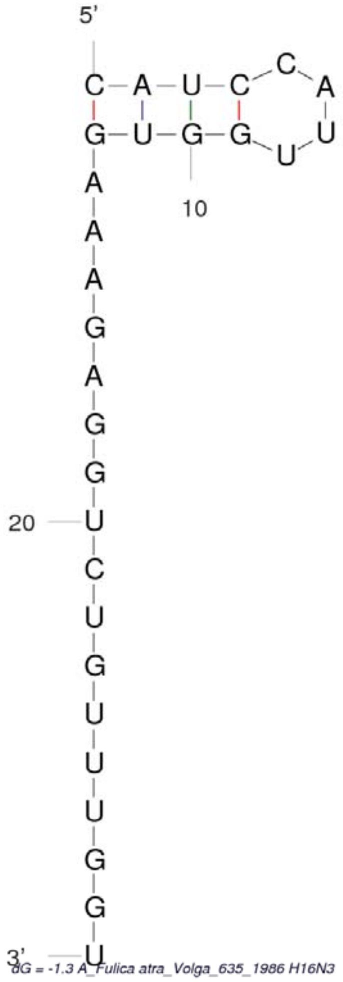

dG = -1.3 A_Fulica atra_Volga_635_1986 H16N3

Output of sir_graph (©)
mfold_util 4.7

Created Sat Apr 9 05:19:07 2016

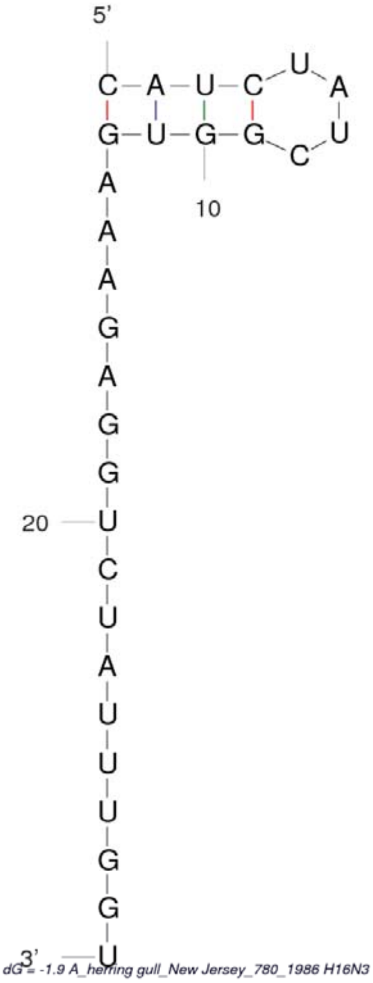

dG = -1.9 A_herring gull_New Jersey_780_1986 H16N3

Output of sir_graph (©)
mfold_util 4.7

Created Sat Apr 9 05:19:08 2016

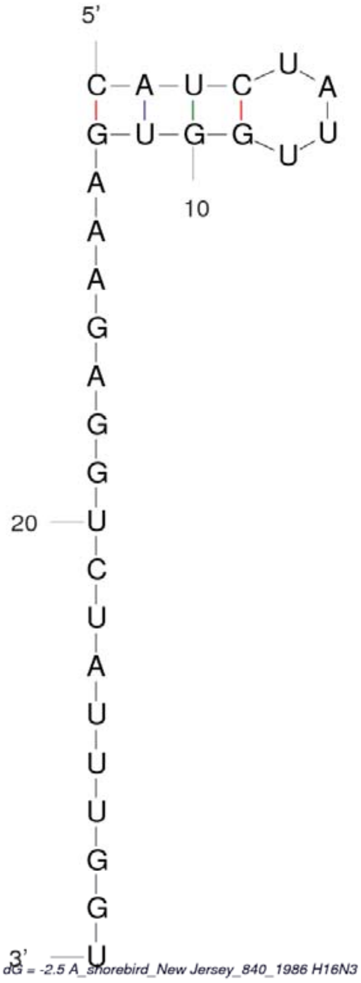

dG = -2.5 A_shorebird_New Jersey_840_1986 H16N3

Output of sir_graph (©)
mfold_util 4.7

Created Sat Apr 9 05:19:08 2016

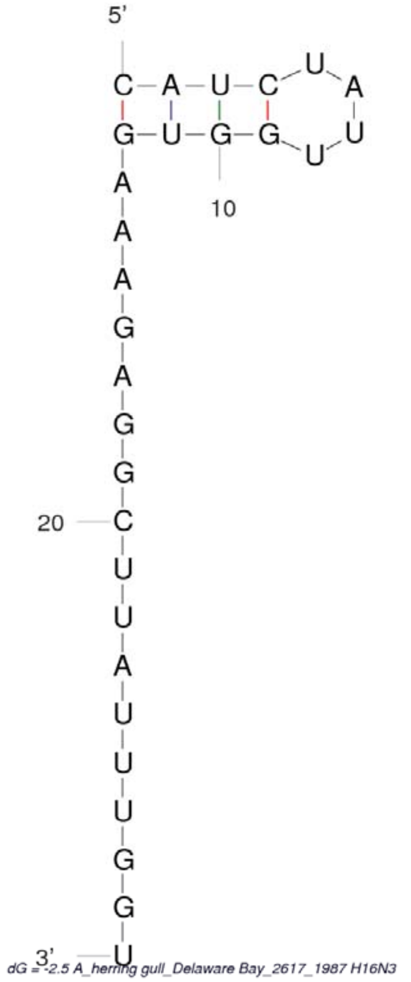

dG = -2.5 A_herring gull_Delaware Bay_2617_1987 H16N3

Output of sir_graph (©)
mfold_util 4.7

Created Sat Apr 9 05:19:08 2016

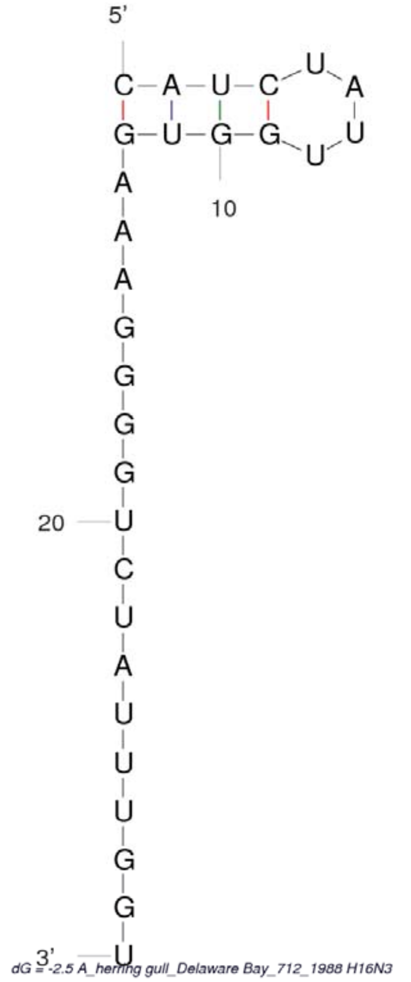

dG = -2.5 A_herring gull_Delaware Bay_712_1988 H16N3

Output of sir_graph (©)
mfold_util 4.7

Created Sat Apr 9 05:19:08 2016

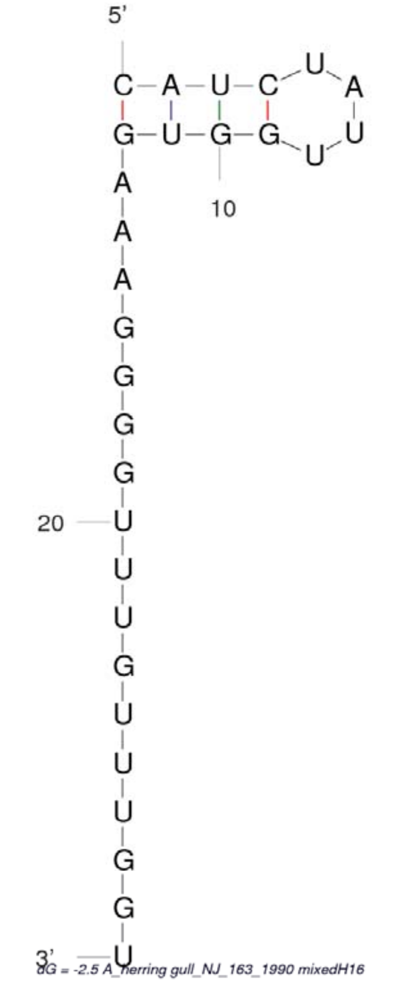

dG = -2.5 A_herring gull_NJ_163_1990 mixedH16

Output of sir_graph (©)
mfold_util 4.7

Created Sat Apr 9 05:19:08 2016

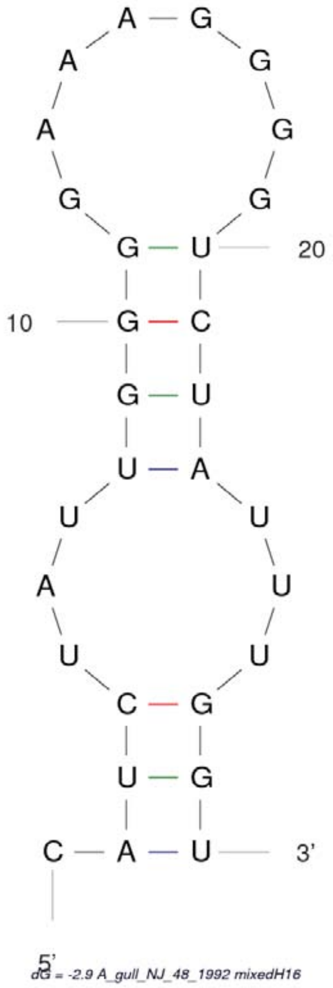

dG = -2.9 A_gull_NJ_48_1992 mixedH16

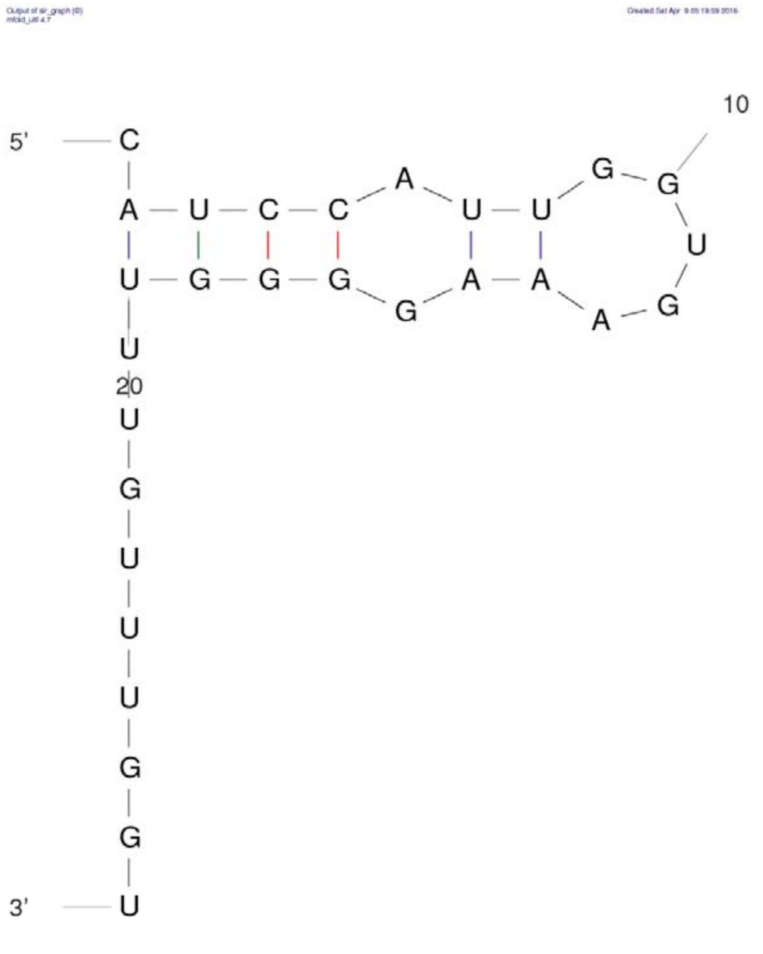

dG = -1.9 A_laughing gull_Delaware Bay_296_1998 H16N3

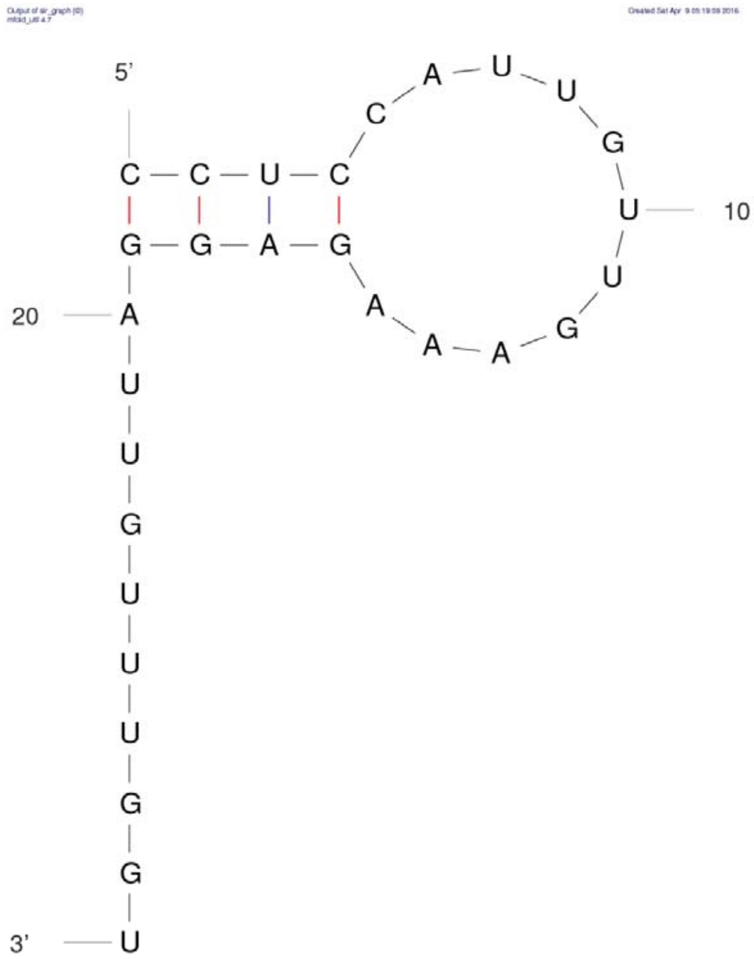

dG = -3.3 A_black-headed gull_Sweden_2_99 H16N3

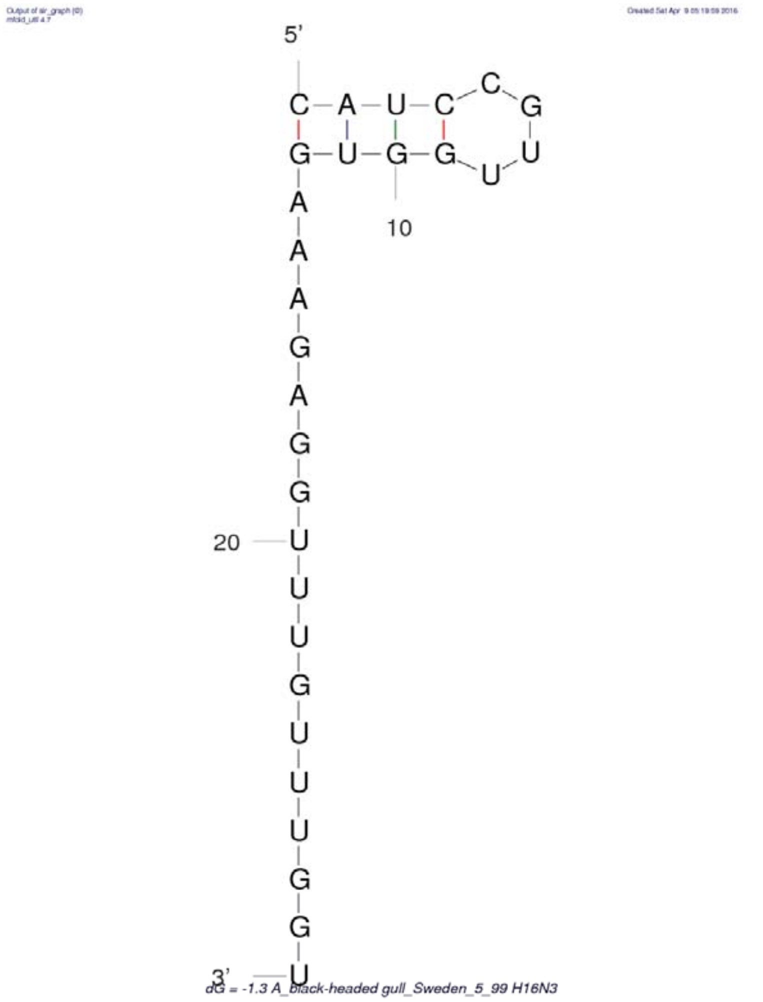

dG = -1.3 A_black-headed gull_Sweden_5_99 H16N3

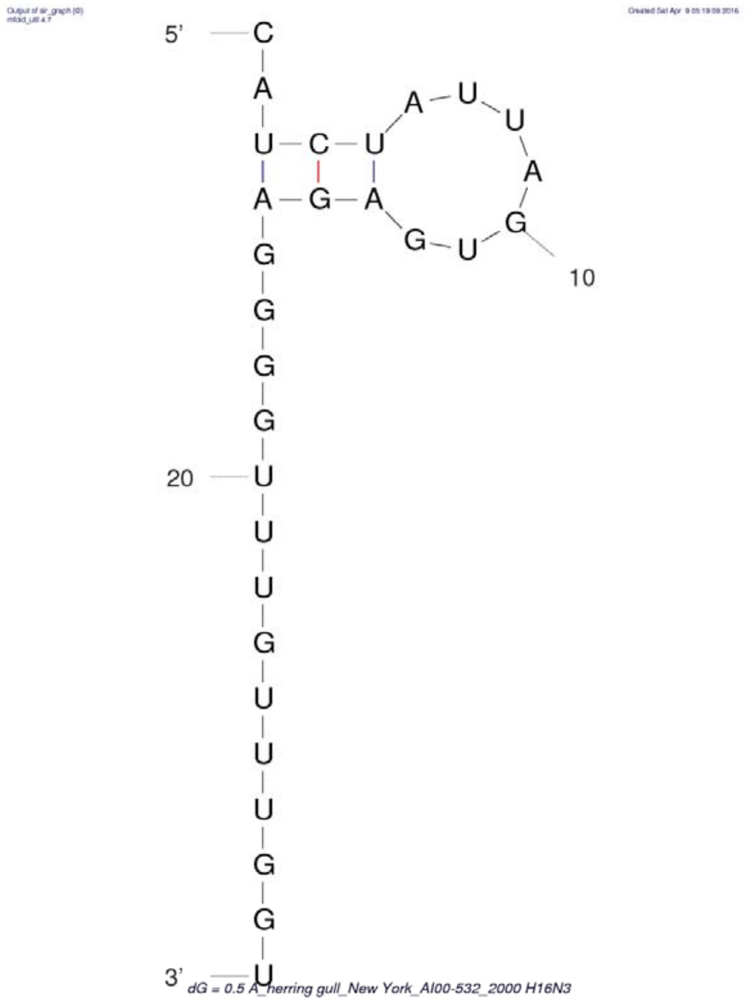

dG = 0.5 A_herring gull_New York_AI00-532_2000 H16N3

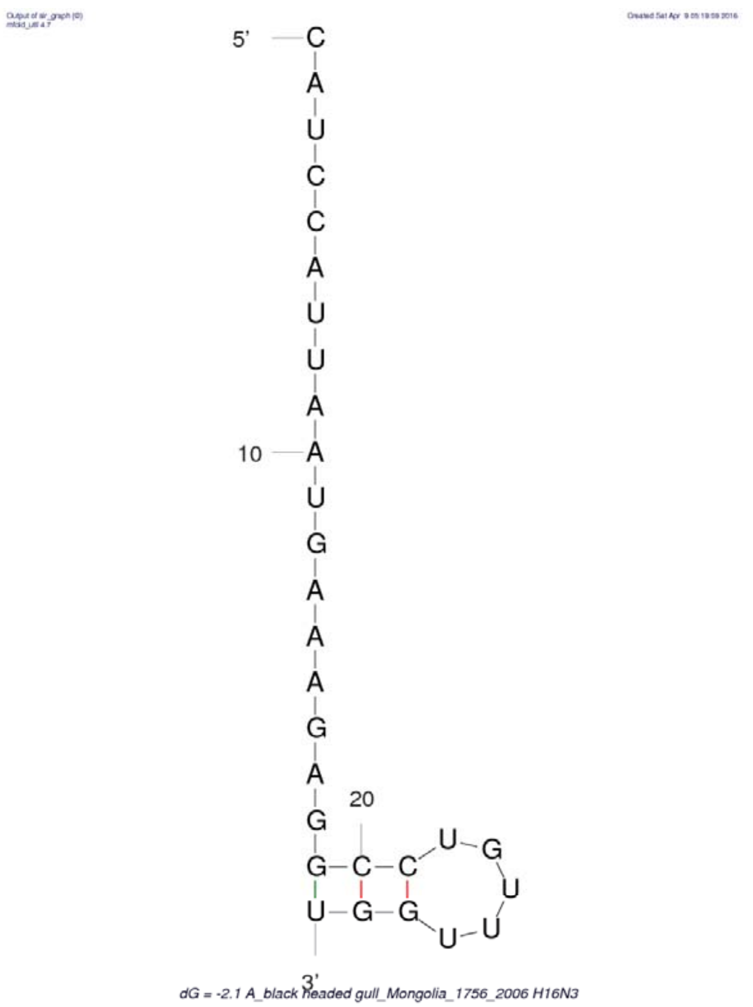

dG = -2.1 A_black headed gull_Mongolia_1756_2006 H16N3

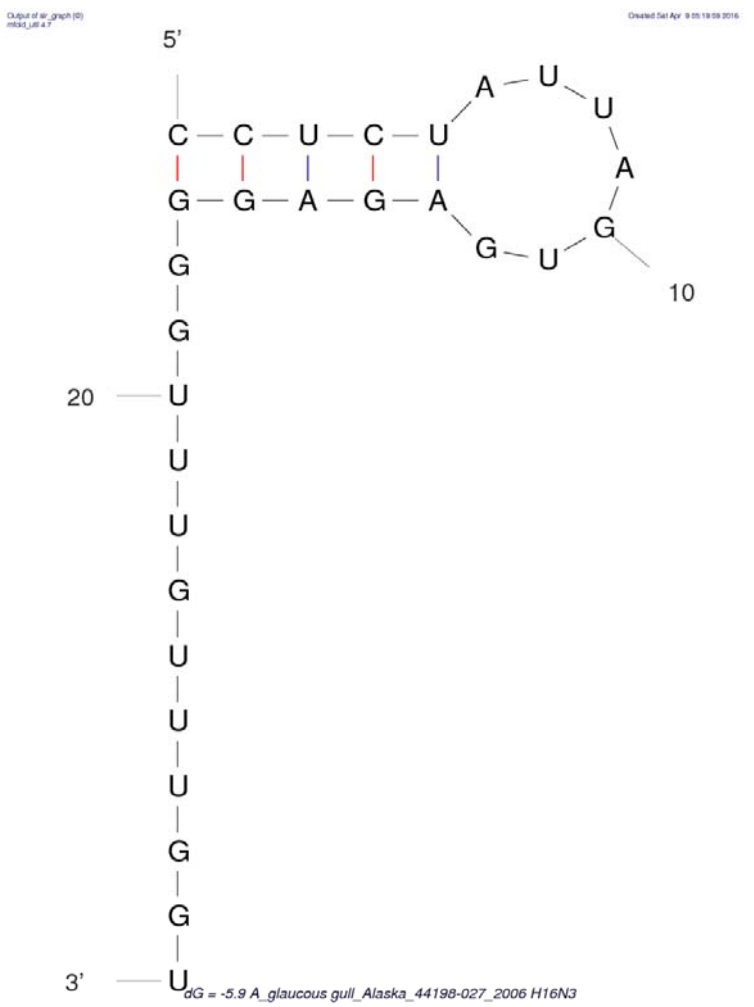

dG = -5.9 A_glaucous gull_Alaska_44198-027_2006 H16N3

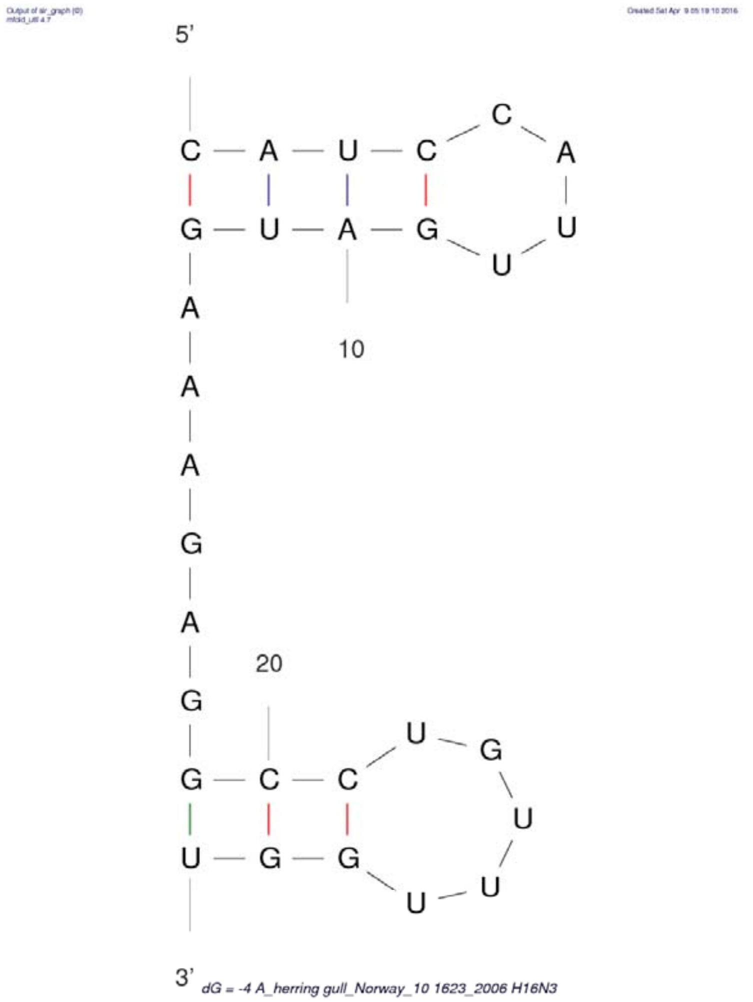

dG = -4 A_herring gull_Norway_10 1623_2006 H16N3

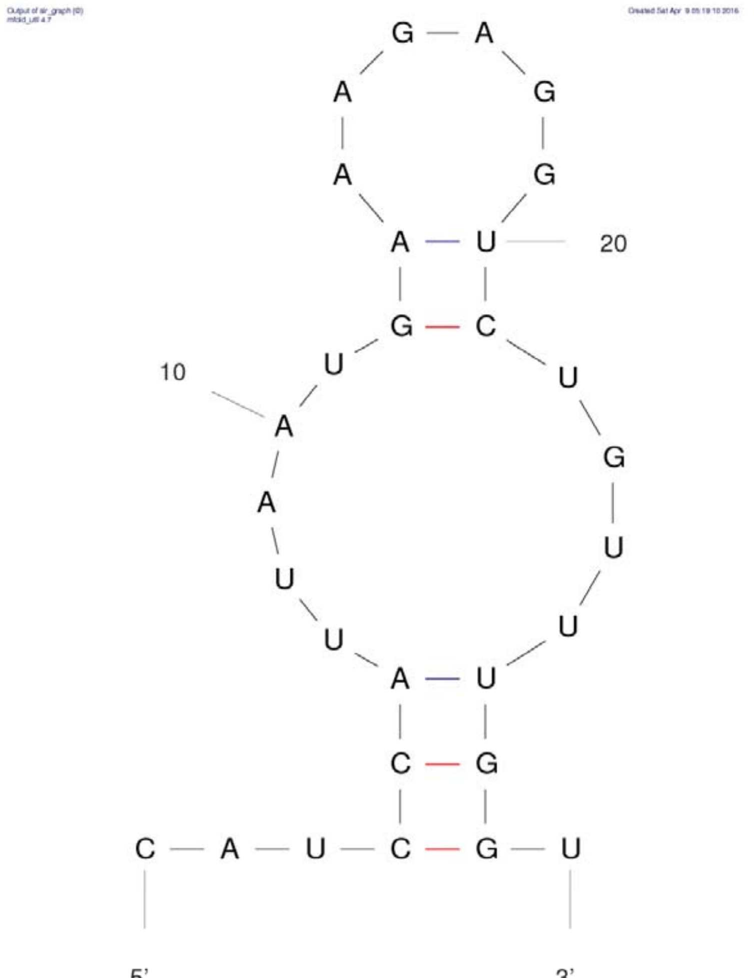

dG = -1.2 A_glaucous-winged gull_Southcentral Alaska_12NH01265_2008 H16N3

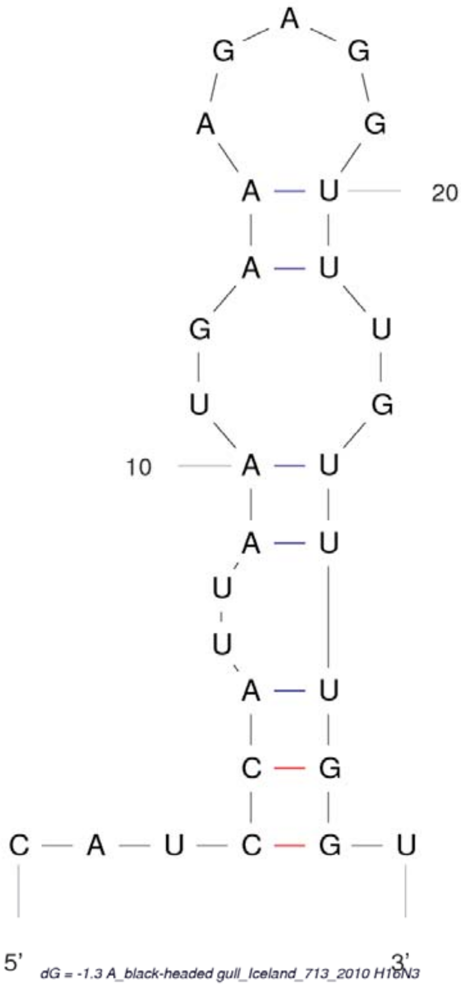

dG = -1.3 A_black-headed gull_Iceland_713_2010 H16N3

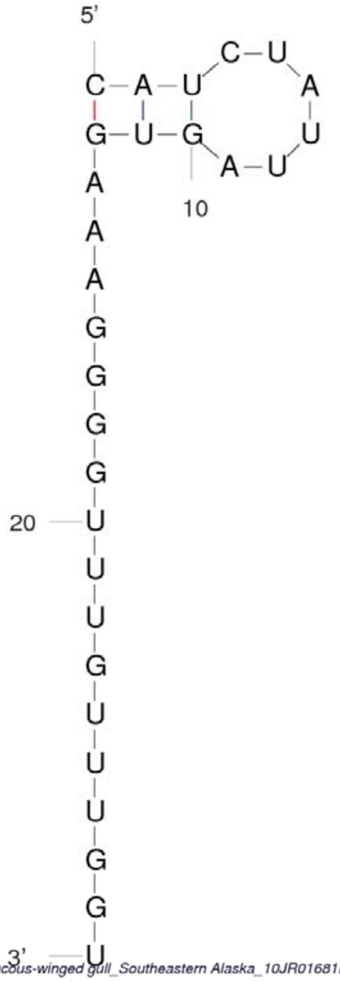

dG = 0.6 A_glaucous-winged gull_Southeastern Alaska_10JR01681R0_2010 H16N3

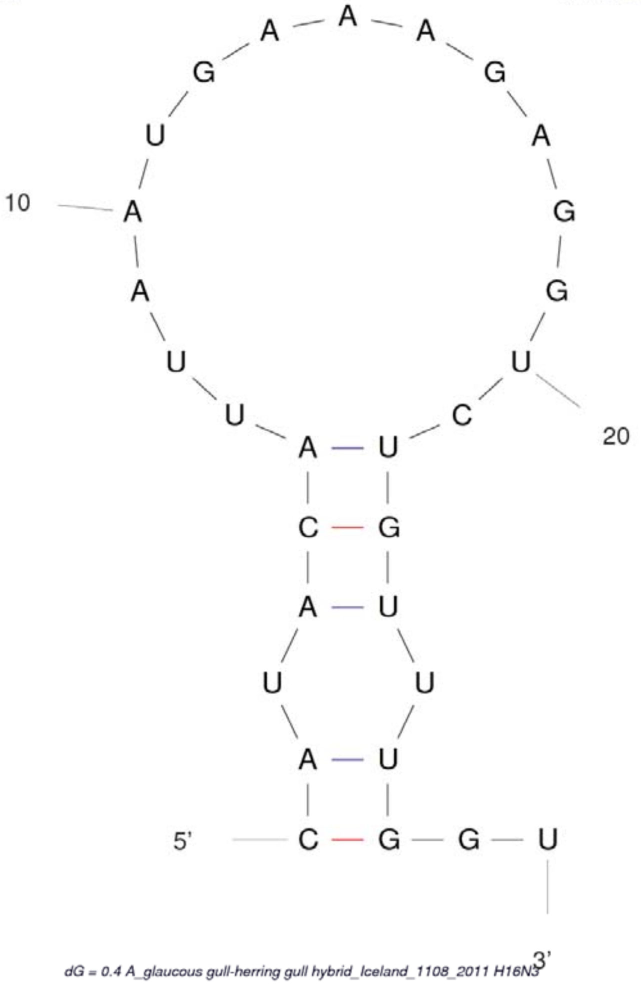

dG = 0.4 A_glaucous gull-herring gull hybrid_Iceland_1108_2011 H16N3

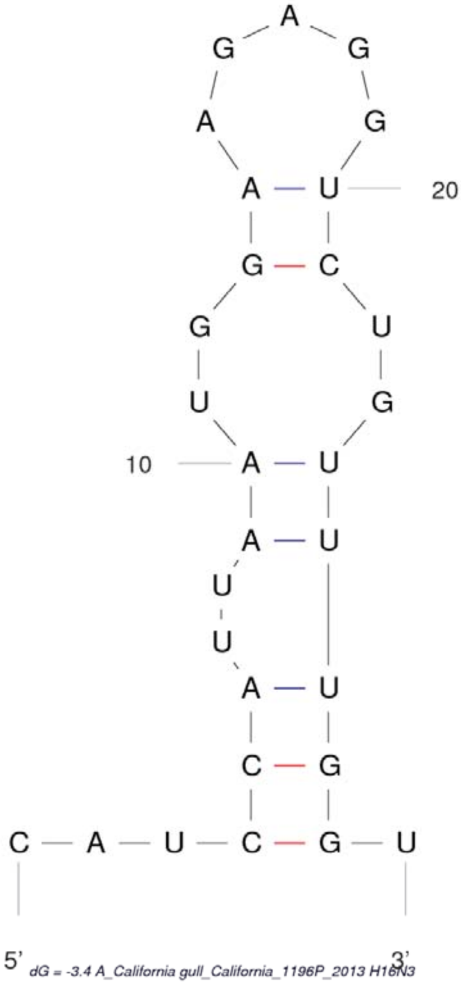

dG = -3.4 A_California gull_California_1196P_2013 H16N3

Output of sir_graph (©)
mfold_util 4.7

Created Sat Apr 9 05:19:11 2016

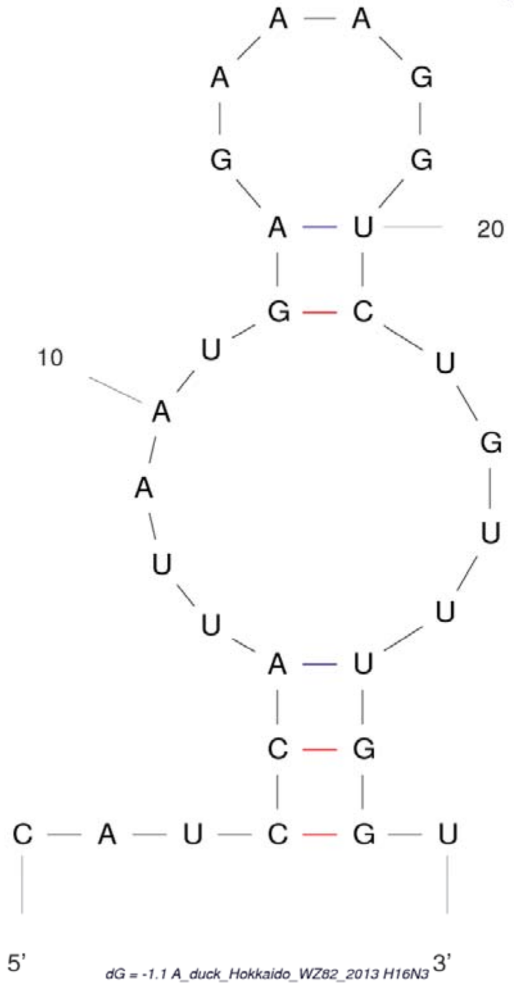

dG = -1.1 A_duck_Hokkaido_WZ82_2013 H16N3
